# Merging directed sp^3^ and nondirected sp^2^ C–H functionalization for Pd-catalyzed polydeuteration of (hetero)arenes†

**DOI:** 10.1039/d5sc01407g

**Published:** 2025-05-08

**Authors:** Soo Eun Park, Sungjun Choi, Chaewon Lim, Sang Hak Lee, Siyeon Jeong, Jung Min Joo

**Affiliations:** a Department of Chemistry, College of Sciences, Kyung Hee University Seoul 02447 Republic of Korea jmjoo@khu.ac.kr; b Department of Chemistry, Pusan National University Busan 46241 Republic of Korea

## Abstract

Polydeuteration has emerged as a key strategy in the development of pharmaceuticals and functional organic materials, advancing beyond monodeuteration and trideuteromethylation. We have developed methods for the polydeuteration of a wide range of organic compounds through Pd-catalyzed directed sp^3^ C–H activation and nondirected sp^2^ C–H activation, using readily available deuterium source, AcOH-*d*_4_. This approach addresses the challenge of facilitating both directed and nondirected C–H functionalization of electronically and sterically diverse (hetero)aromatic compounds through the use of a versatile [2,2′-bipyridin]-6(1*H*)-one (BpyOH) ligand. This method demonstrates high functional group compatibility, readily applicable in the presence of directing functional groups such as carboxylic acids, amides, and azoles, as well as nondirecting electron-withdrawing groups such as nitro, sulfonamide, and ester groups. DFT calculations reveal that ligands influence intermediates and transition states by providing bidentate chelation, internal base, and hydrogen bonding. The Pd(BpyOH) complex exhibits well-balanced reactivity for C–H cleavage while readily forming complexes with substrates, which is relevant to other Pd-catalyzed C–H functionalization reactions. Our approach significantly broadens the scope of deuterated building blocks and late-stage deuteration, thereby facilitating evaluation of the deuterium effect in various applications across medicinal chemistry, materials science, and beyond.

## Abstract 2

A ligand-enabled strategy achieves deuteration with high deuterium enrichment through both directed sp^3^ and nondirected sp^2^ C–H deuteration.

## Graphical abstract

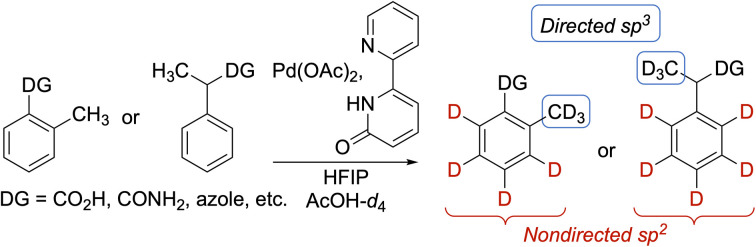

## Introduction

Deuterium labelling of organic compounds has been critical in drug discovery and materials science, providing both target compounds and analytical tools.^1^ The incorporation of deuterium improves the pharmacokinetic properties and safety of compounds, most notably by increasing their stability due to the stronger C–D bonds compared to C–H bonds.^2^ This isotopic substitution can lead to increased metabolic stability, prolonged biological half-life, and reduced toxicity, all of which are highly desirable attributes for potential therapeutic agents. Beyond pharmaceutical applications, deuterium labeling also finds utility in organic light-emitting devices and fluorophores, where it aids in enhancing efficiency and lifetime of organic functional materials.^3^ In addition, the preparation of deuterated analogs is frequently performed to conduct mechanistic studies, enabling researchers to elucidate reaction pathways and intermediates through kinetic isotope effect measurements and mass spectrometry analysis.^4^ Therefore, developing deuteration methods of various functional group-containing organic compounds is highly desirable to fully harness the benefits of deuterium labeling across varied applications.

**Fig. 1:**
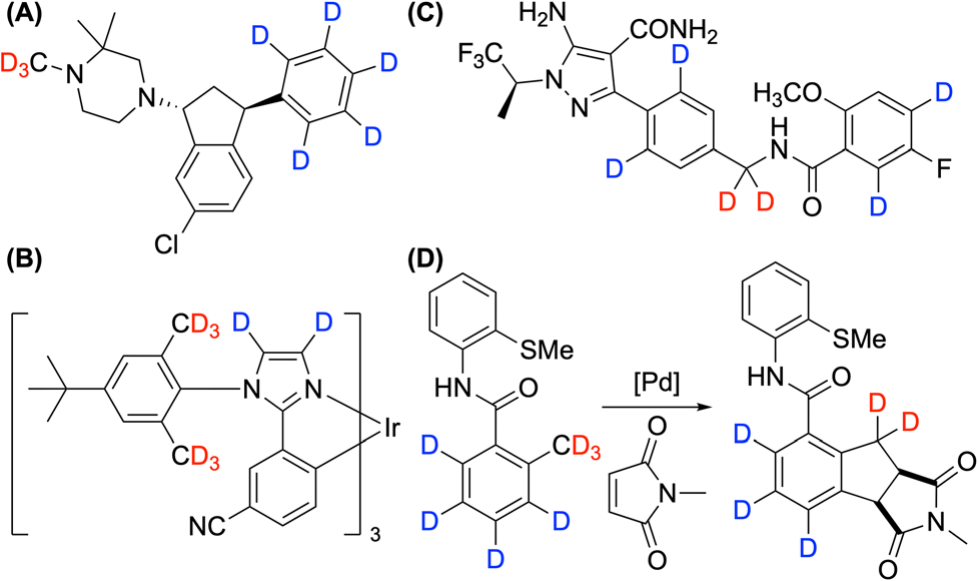
Examples of polydeuterated compounds: (A) Lu AF35700 used in drug development for enhanced metabolic stability. (B) An Ir-arylimidazole dopant employed in phosphorescent OLEDs for increased lifetime. (C) Polydeuterated pirtobrutinib as a mass spectrometry internal standard. (D) An essential substrate for mechanistic studies involving sp^2^ and sp^3^ C–H activation.

The advantage of deuteration can be precisely accessed when deuterated analogs are prepared with high selectivity and efficiency. Among various types of deuteration methods, monodeuteration and trideuteromethylation are frequently performed using commercially available, highly D-enriched donors, such as CH_3_I-*d*_3_ and CH_3_OH-*d*_4_. However, polydeuteration is required when multiple sites within a molecule influence its stability. For example, deuteration of the phenyl ring notably slowed the rate of *N*-demethylation of Lu AF35700 (Fig. 1A).^5^ In addition, the operational lifetime of OLED devices has been extended by deuterating vulnerable benzylic and heteroaromatic sites (Fig. 1B).^3*b*^ Polydeuteration is also critical in the synthesis of D-labelled internal mass standards to achieve the necessary mass difference for quantitative analysis, as illustrated by the case of pirtobrutinib (Fig. 1C).^6^ Furthermore, C–H activation reactions that combine sp^2^ and sp^3^ C–H activation requires the preparation of polydeuterated compounds for mechanistic studies (Fig. 1D).^7^

Although polydeuterated compounds are essential in various applications, they present substantial challenges in achieving high D incorporation. Although functional group transformations, such as reductive deuteration and dehalogenative deuteration, are useful for selective deuterium incorporation,^8^ hydrogen isotope exchange (HIE) offers an advantageous single-step approach to directly replace ubiquitous C–H bonds of organic compounds with multiple C–D bonds.^9^ This HIE strategy has been successfully achieved by acid/base and heterogeneous catalysis, significantly advancing the development of polydeuteration.^10^

**Fig. 2:**
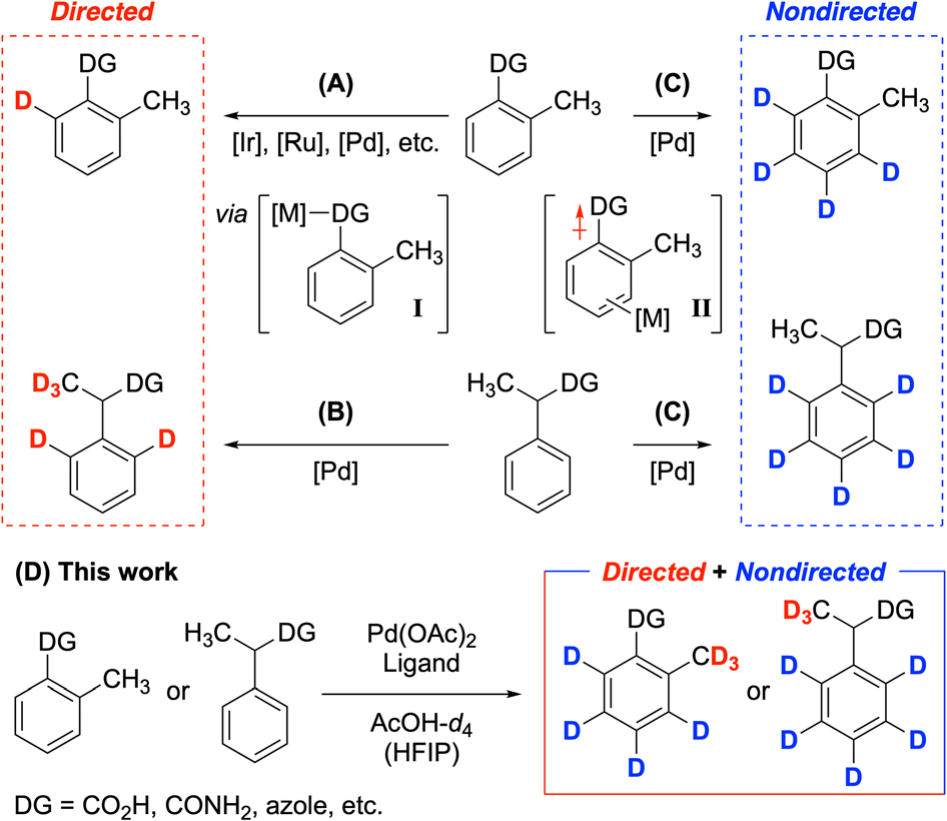
(A) Directed sp^2^ C–H deuterations by various transition metal catalysts. (B) Pd-catalyzed directed sp^2^ and sp^3^ C–H deuteration. (C) Nondirected sp^2^ C–H deuteration. (D) Merging directed sp^3^ and nondirected sp^2^ C–H deuteration.

Complementarily, transition-metal-catalyzed C–H functionalization has broadened the substrate scope of deuteration by enhancing functional group compatibility (Fig. 2).^11^ Particularly, directing groups improve both the efficiency and selectivity of HIE processes, enabling deuteration at *ortho*-sp^2^ C–H bonds (Fig. 2A).^1*a*,12^ However, in contrast to a wide range of directing groups and transition metal catalysts available for directed sp^2^ C–H deuteration, limited examples of directed sp^3^ C–H deuteration have been reported. A notable example is the Pd-catalyzed deuteration of carboxylic acids, which deuterates both aromatic and α-methyl C–H bonds adjacent to the directing carboxylic acid group (Fig. 2B).^13^ Conversely, nondirected sp^2^ C–H perdeuteration replaces hydrogen atoms at sp^2^ carbon atoms of (hetero)arenes with deuterium, while leaving other aliphatic positions, including those near directing groups, unchanged (Fig. 2C).^14^

Although it is conceivable that merging directed and nondirected C–H activation could enable polydeuteration, its success has been limited due to differing strategies needed for each process.^15^ While directing groups can facilitate directed C–H activation by coordinating with metal complexes (Fig. 2, complex I), they can contrarily hinder activation at remote positions. Once the metal complex dissociates from the directing group, the electron-withdrawing nature of many directing groups (such as carboxylic acids) decreases electron density, weakening the π-complexation step necessary for nondirected C–H activation (Fig. 2, complex II).^16^ During our studies on nondirected Pd-catalyzed sp^2^ C–H perdeuteration of (hetero)arenes, we found that carboxylic acid derivatives exhibited incomplete deuteration, both directed and nondirected.^17^ Consequently, the resulting deuterated analogs were unsuitable for use in medicinal chemistry and mechanistic studies.^18^ To achieve polydeuteration at aromatic and directed methyl C–H bonds while leaving other aliphatic positions unreacted, we sought to identify key factors that facilitate activation at both nondirected sp^2^ and directed sp^3^ positions. Our new ligand-enabled method is compatible with a variety of electron-withdrawing groups (EWGs), including carboxylic acids, amides, and azoles with directing effects, as well as nondirecting nitro, sulfonamide, and ester groups (Fig. 2D). Notably, *ortho*-tolyl groups were fully deuterated, which is rarely reported, addressing the challenges of activating *ortho*-methyl C–H bonds.^19^ This method provides versatile deuterated derivatives, ranging from simple building blocks to complex pharmaceuticals, with high levels of deuterium incorporation.

## Results and discussion

Preliminary ligand screening revealed the importance of incorporating a 2-pyridone unit as an internal base for efficient deuteration.^20^ To facilitate directed deuteration, several heterocycle-substituted bidentate pyridone ligands with varying electronic and steric characters were examined, ultimately identifying three key ligands, 6-(3-methyl-1*H*-pyrazol-1-yl)pyridin-2(1*H*)-one (3-Me-PzPyOH),^17^ [2,2′-bipyridin]-6(1*H*)-one (BpyOH),^21^ and [2,2′-bipyridine]-6,6′(1*H*,1′*H*)-dione (Bpy(OH)_2_) (Table 1).^22^ Compared to the pyrazole ligand 3-Me-PzPyOH,^23^ the more Lewis-basic pyridine-substituted ligands BpyOH and Bpy(OH)_2_ demonstrated superior results in directed deuteration at sp^3^ positions, while maintaining comparable D incorporation at nondirected sp^2^ positions ([D]1 and [D]2). Particularly, Bpy(OH)_2_ outperformed BpyOH in directed sp^3^ deuteration of five-membered heteroarenes, which are more challenging to deuterate than six-membered arenes because the larger acute bond angles in five-membered rings renders palladacycle formation more difficult ([D]3).^24^ However, BpyOH showed consistently high efficiency, even in substrates with nondirecting EWGs such as ester and nitro groups ([D]4, *vide infra*), thus selected as an optimal ligand.

**Table 1:** Ligand effect on directed and nondirected deuteration reactions

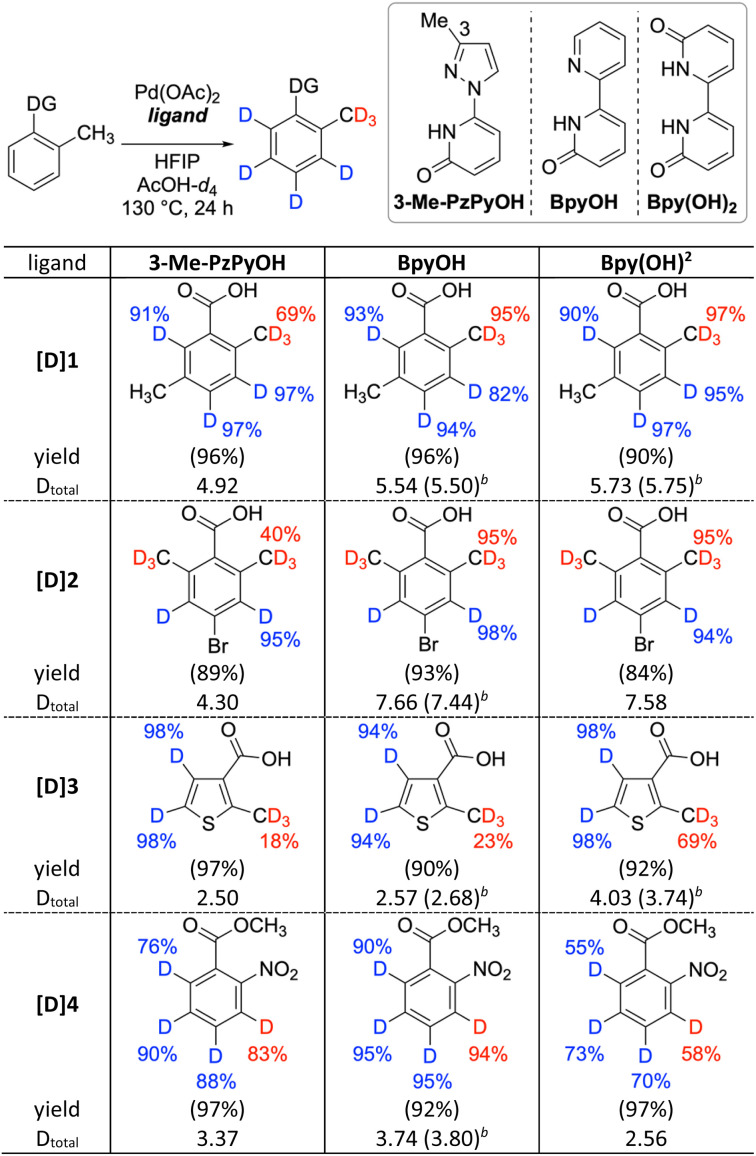

a Reaction conditions: substrate (0.2 mmol), Pd(OAc)_2_ (0.02 mmol), ligand (0.02 mmol), HFIP (0.2 mmol), AcOH-*d*_4_ (0.2 M), at 130 °C, and 24 h. D incorporation by ^1^H NMR. Isolated yields in parentheses. D incorporation at the positions lower than 10% is not listed.

b By mass analysis.

The D source can be either AcOH-*d*_4_ or D_2_O, with the latter being more sensitive to the solubility of substrates, thus limiting its scope (see the ESI†). The addition of 1,1,1,3,3,3-hexafluoroisopropanol (HFIP) was not critical for the degree of deuteration but sometimes slightly improved the yield.^25^ Extending the reaction time from 24 to 48 hours further increased deuteration, and a catalyst loading of 5 mol% was found to be feasible, resulting in only a slight decrease in deuterium incorporation. Several acid/base additives were examined; however, they did not improve the overall D incorporation. In the absence of Pd(OAc)_2_, deuterium incorporation was not observed at either the sp^2^ or sp^3^ positions. Furthermore, BpyOH itself undergoes deuteration but is expected to maintain similar catalytic activity (see the ESI†).

**Scheme 1:**
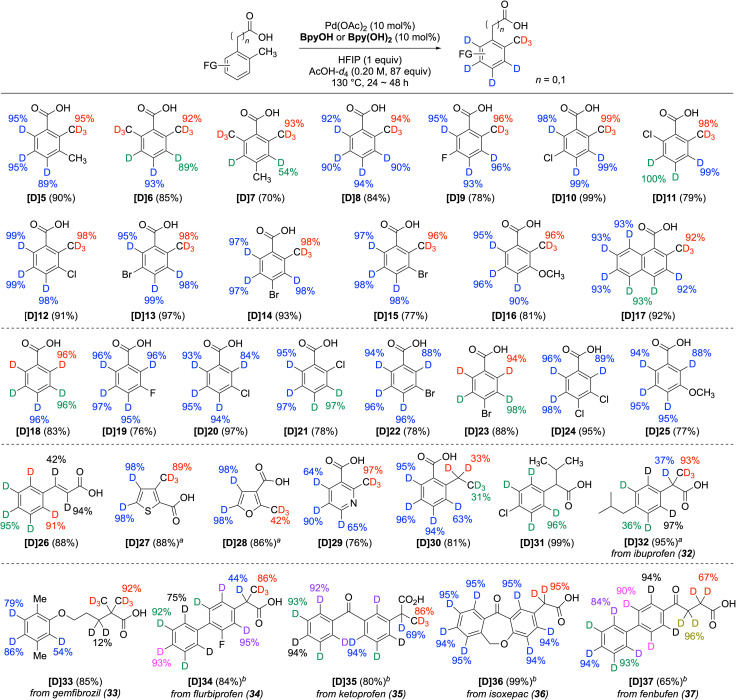
Directed and nondirected deuteration of carboxylic acids. Reaction conditions: substrate (0.2 mmol), Pd(OAc)_2_ (0.02 mmol), BpyOH or Bpy(OH)_2_ (0.02 mmol), HFIP (0.2 mmol), AcOH-*d*_4_ (0.2 M), 130 °C, for 24 h. D incorporation at the positions lower than 10% is not listed. The yield indicated in blue represents the D incorporation at a single C–H bond, whereas the yields indicated in other colors represent D incorporation at one or more C–H bonds with similar chemical shifts that have been replaced by C–D bonds. ^*a*^Bpy(OH)_2_ instead of BpyOH. ^*b*^For 48 h.

A representative method using BpyOH was applied to the polydeuteration of a variety of carboxylic acid derivatives (Scheme 1). The aromatic rings and *ortho*-methyl groups of benzoic acids were readily deuterated (5–17). Notably, for the sp^3^-methyl positions of dimethylbenzoic acid, selective deuteration occurred exclusively at the directed methyl group (5 and 7). *Ortho*-toluic acid (8) and its halogenated derivatives (9–15) underwent successful perdeuteration, demonstrating tolerance to various positions and types of halogens. The methoxy and naphthalene variants (16 and 17) were also suitable for polydeuteration. In addition to *ortho*-toluic acid derivatives, the BpyOH system enabled high deuterium incorporation for benzoic acid (18) and its derivatives. The efficient sp^2^ C–H deuteration of monohalogenated benzoic acids (19–23), *m*,*p*-dichloro benzoic acid (24), *m*-methoxy benzoic acid (25), and cinnamic acid (26) demonstrates the high functional group compatibility of the method. Consistent with the deuteration of 2-methylthiophene-3-carboxylic acid (3), the methyl groups of five-membered heteroaromatic carboxylic acids (27 and 28) were deuterated slightly more efficiently by Bpy(OH)_2_. However, although the methyl group of six-membered heteroaromatic carboxylic acids, such as 29, is activated and readily deuterated,^26^ their sp^2^ C–H deuteration is generally less efficient than that of five-membered heteroarenes, provided that decarboxylation is not significant (see the ESI†).^27^ Furthermore, while the aromatic ring of 2-ethylbenzoic acid (30) and 2-(4-chlorophenyl)-3-methylbutanoic acid (31) underwent efficient deuteration, the ethyl and propyl groups largely remained intact. In the case of 2-phenylbutanoic acid, a phenylacetic acid containing an ethyl group at the α-position, a considerable amount of dehydrogenation products was formed, as previously reported (see Scheme S3†).^21*e*^ Additionally, the scope was investigated using commercially available pharmaceuticals containing a carboxylic acid. For example, ibuprofen (32), gemfibrozil (33), flurbiprofen (34), ketoprofen (35), isoxepac (36), and fenbufen (37), which were only partially deuterated by known methods,^13*a*,14*c*^ were converted to the corresponding polydeuterated derivatives, clearly illustrating the advantage of the Pd/BpyOH system in this series of pharmaceuticals. The combined results from 30 to 35 demonstrate that this method is advantageous for the deuteration of sterically unhindered directed sp^3^ C–H bonds, including *ortho*- and α-methyl groups adjacent to directing groups, although it is not suitable for larger alkyl groups.

**Scheme 2:**
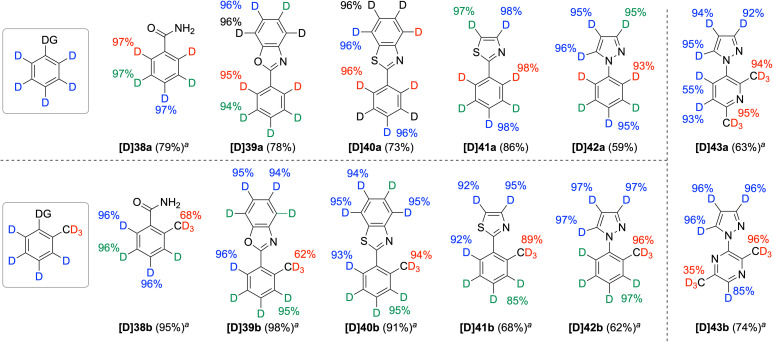
Directed and nondirected deuteration of amide and heteroaromatic compounds. For reaction conditions, see Scheme 1. ^*a*^For 48 h.

Although directed HIE has made significant advances,^1*a*^ catalytic systems that are universal to a wide range of directing groups remain uncommon. However, extending the Pd/BpyOH system to other directing groups, such as amide and azole, enabled perdeuteration of both parent compounds and *ortho*-methyl variants (Scheme 2). Despite the weaker directing effects of benzamides (38a and 38b) and benzoxazoles (39a and 39b) compared to benzoic acids, moderate deuteration occurred at the methyl groups, along with full deuteration at both aromatic and heteroaromatic C–H bonds. Both benzothiazoles (40a and 40b) and thiazoles (41a and 41b) serve as useful directing groups to promote D incorporation at the *ortho*-methyl group, while all sp^2^ C–H bonds were also efficiently deuterated. In addition, another class of azole, pyrazole, proved to be an excellent directing group, affording the corresponding deuterated analogs of *N*-phenylpyrazole and *N*-(*o*-tolyl)pyrazole (42a and 42b, respectively). Substituting aryl rings with six-membered heteroaryl rings, such as pyridine and pyrazine, did not hinder the deuteration of pyrazole rings (43a and 43b). Similar to 2-methylnicotinic acid (29), the methyl groups readily underwent deuteration, while the sp^2^ positions of the six-membered rings showed relatively low D incorporation.^26^ However, one methyl group of pyrazine 43b was underdeuterated, presumably due to the bidentate coordination involving the nitrogen atoms of both the pyrazole and pyrazine rings (for additional six-membered heteroarene substrates, see Scheme S3†).

**Scheme 3:**
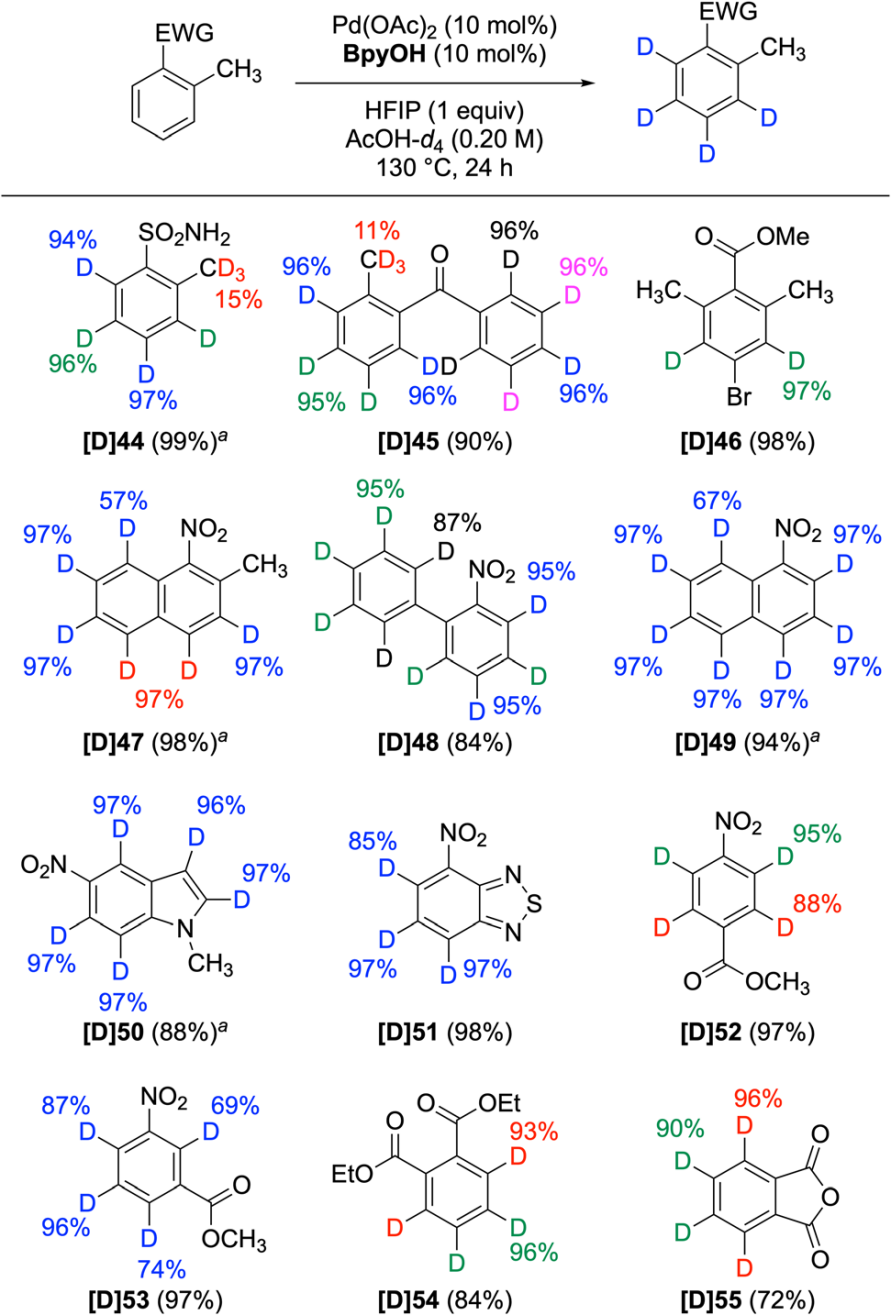
Nondirected deuteration of electron-deficient arenes. For reaction conditions, see Scheme 1. ^*a*^For 48 h.

Furthermore, the Pd/BpyOH method facilitated high deuterium incorporation at sp^2^ C–H positions in electron-deficient (hetero)aromatic compounds (Scheme 3). In contrast to carboxylic acids, amides, and azoles, other EWGs such as sulfonamide, ketone, ester, and nitro groups did not exert directing effects at benzylic methyl positions (44–47). However, substrates containing a single EWG (48–51), as well as those with two EWGs (52–55), underwent deuteration at all sp^2^ C–H bonds. The capability to deuterate electron-deficient (hetero)arenes significantly expands the range of deuterated building blocks, demonstrating its broad applicability.

**Fig. 3:**
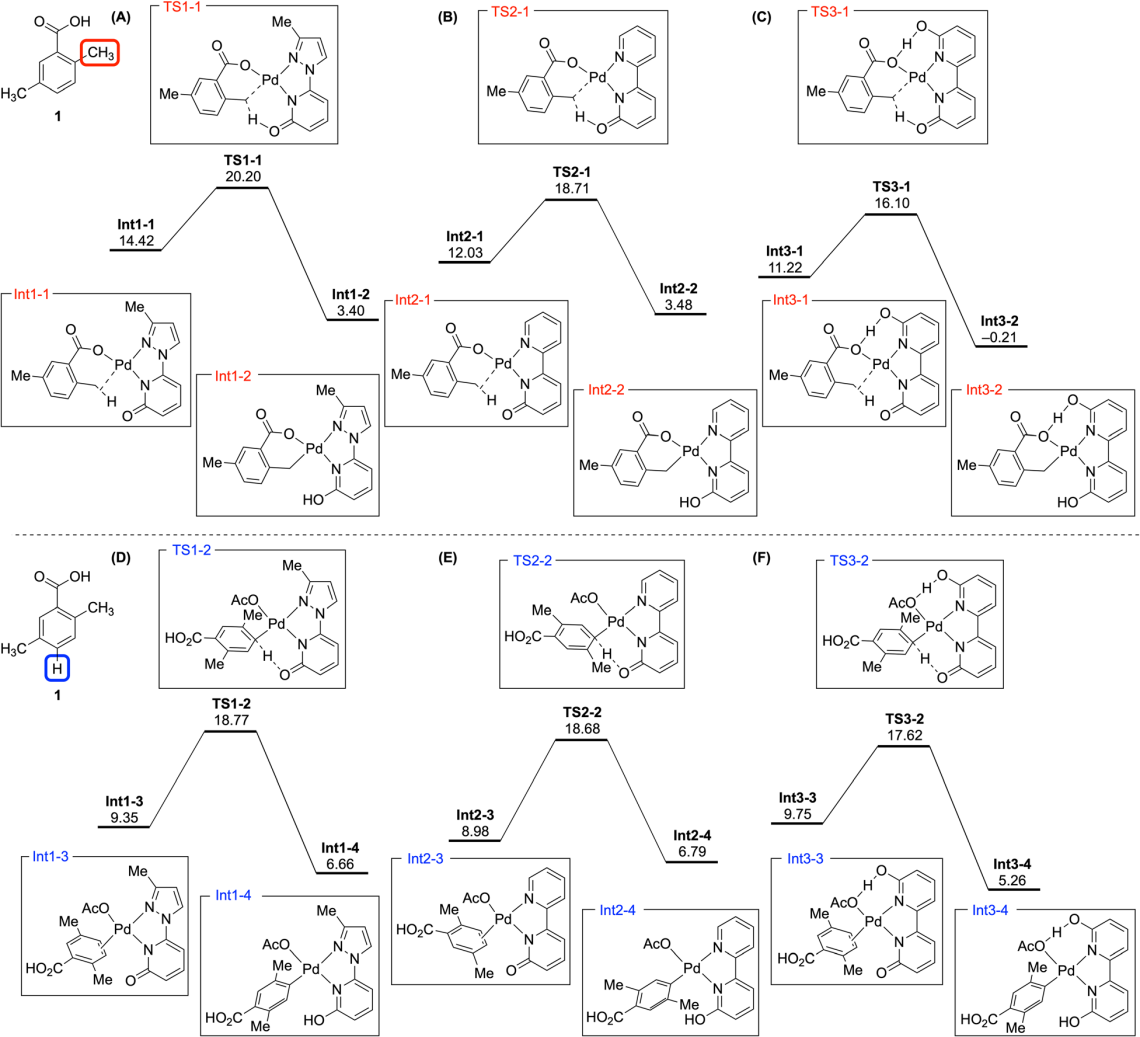
Calculated energy profiles showing deuteration pathways at two sites of 2,5-dimethylbenzoic acid (1): the *ortho*-methyl position (A–C) and *para*-position (D–F). Results are shown for catalysis with ligands 3-Me-PzPyOH, BpyOH, and Bpy(OH)_2_ (A/D, B/E, and C/F, respectively). DFT calculations were performed at the uB3LYP-D3/SDD-6-311+G**/SMD(AcOH)//uB3LYP-D3/LANL2DZ-6-31G** level of theory. Free energies (kcal mol^−1^) relative to 1/3 Pd_3_(OAc)_6_.

To elucidate the reactivity differences between directed sp^3^ C–H bonds and nondirected sp^2^ C–H bonds across three pyridone ligands, DFT calculations were conducted for Pd-catalyzed deuteration reactions of compound 1 (Fig. 3). Relative Gibbs free energies were calculated using the 1/3 Pd_3_(OAc)_6_ complex as a reference to compare energies, with representative calculations for the aromatic positions performed at the *para* position. C–H activation processes are reversible, requiring consideration of both intermediates and transition states.^28^ In addition, because each ligand was employed independently, we focused on identifying trends rather than comparing absolute values between ligands.

For the directed methyl position, both the activation barrier and intermediate energy were reduced by switching the ligand from 3-Me-PzPyOH to BpyOH, due to stable bidentate chelation and efficient internal base (Fig. 3A and B). With Bpy(OH)_2_, forming hydrogen bonds between the carboxylate of the substrate and the hydroxypyridine of the ligand was critical in reducing the activation barrier TS3-1 and stabilizing the intermediate Int3-2 (Fig. 3C). A similar trend was observed for the aromatic deuteration transition states, decreasing in the order of 3-Me-PzPyOH, BpyOH, and Bpy(OH)_2_ (Fig. 3D–F). Although not shown, the *ortho*-sp^2^ position, which is susceptible to directed deuteration, demonstrated that nondirected C–H activation has a lower energy barrier than the directed pathway (see the ESI†). This result suggests that all aromatic positions are likely to undergo deuteration in a nondirected fashion, similar to TS2-2. In addition, it is notable that the energy trend of the intermediates at the aromatic positions showed a slight deviation, where Bpy(OH)_2_ afforded a relatively high-energy Int3-3 compared to Int1-3 and Int2-3 derived from the other two ligands (Fig. 3F). This outcome indicates that substrate π-complexation to form an intermediate like Int3-3 could be more challenging than other ligands because of the steric effect of the hydroxy group in Bpy(OH)_2_. The consideration of intermediates may also have implications for the slow deuteration of aromatic positions in electron-deficient (hetero)arenes with Bpy(OH)_2_.^16^

The DFT calculations also facilitated the comparison of positional differences under a given ligand. With 3-Me-PzPyOH, the transition state energy for the aromatic position (TS1-2, Fig. 3D) was lower than that for the methyl position (TS1-1, Fig. 3A). In contrast, BpyOH resulted in similar activation barriers for both positions (Fig. 3B and E). However, calculations for Bpy(OH)_2_ indicated a reversed result, with the barrier at the methyl position being lower than that at the aromatic position (Fig. 3C and F). Therefore, the superiority of the Pd(BpyOH) complex in both directed and nondirected deuteration is attributed to its balanced reactivity for C–H cleavage and its ability to easily complex with substrates.

## Conclusions

Ligand-enabled strategies have been developed to achieve both directed sp^3^ and nondirected sp^2^ C–H deuteration using a single catalytic system. The combination of Pd(OAc)_2_ and BpyOH ligand demonstrated high compatibility with various directing groups such as carboxylic acid, amide, and azole, as well as nondirecting groups, including nitro, sulfonamide, and ester groups. This system tolerated a wide range of electronic and steric properties, enabling efficient perdeuteration of sp^2^ C–H bonds at both electron-rich and electron-deficient sites. DFT calculations revealed that BpyOH ligand facilitates both directed methyl and nondirected aromatic C–H deuteration in a well-balanced manner. The high efficiency, coupled with broad functional group tolerance, facilitate the preparation of deuterated building blocks and the late-stage deuteration of complex pharmaceuticals using convenient deuterium source, AcOH-*d*_4_. This approach significantly expands the pool of deuterated compounds available for drug development and materials applications. This result will further facilitate ligand design for versatile and efficient catalytic systems for hydrogen isotope exchange by homogeneous transition-metal-catalyzed C–H functionalization.

## Author contributions

S. E. Park, S. Choi, C. Lim performed all the reactions and characterization. S. H. Lee and S. Jeong performed the DFT calculations. J. M. J. supervised the project and wrote the manuscript with input from all the authors.

## Conflicts of interest

There are no conflicts of interest to declare.

## Supplementary Material

SC-016-D5SC01407G-s001

## Data Availability

The ESI† is available and contains experimental procedures, compounds characterization, and computational studies.
