# Supplementary material for: Merging directed sp3 and nondirected sp2 C–H functionalization for Pd-catalyzed polydeuteration of (hetero)arenes
Source: Chem Sci. 2025 May 8;16(23):10349–56. doi: 10.1039/d5sc01407g (PMC12059772; doi:10.1039/d5sc01407g)
Supplement: SC-016-D5SC01407G-s001 [file SC-016-D5SC01407G-s001.pdf]

Supplementary Information (SI) for Chemical Science.
This journal is © The Royal Society of Chemistry 2025

Supporting Information

Merging Directed sp^3 and Nondirected sp^2 C–H Functionalization for Pd-Catalyzed Polydeuteration of (Hetero)Arenes

Soo Eun Park,[1] Sungjun Choi,[1] Chaewon Lim,[1] Sang Hak Lee,[2] Siyeon Jeong,[1,*] and Jung Min Joo[1,*]

[1]Department of Chemistry, College of Sciences, Kyung Hee University, Seoul 02447, Republic of Korea;

[2]Department of Chemistry, Pusan National University, Busan 46241, Republic of Korea

I. Table of Contents……………………………………………………S1

II. General Information……………………………………………………S2

III. Experimental Procedures……………………………………………S3

IV. Optimization Studies……………………………………………………S5

V. H/D Exchange Experiments…………………………………………S13

VI. Compound Characterization Data……………………………………S15

VII. DFT Calculation……………………………………………………S254

VIII. References ……………………………………………………………S275

II. General Information

Flash chromatography was performed on 40-63 μm silica gel using indicated eluents. NMR spectra were recorded in $CDCl_3$, CD_2Cl_2, CD_3OD, $(CD_3)_2CO$, AcOH-d_4, and DMSO-d_6 at 300 K on a JEOL 500 MHz NMR spectrometer. Proton chemical shifts are expressed in parts per million (ppm, δ scale) and are referenced to residual protium in NMR solvents ($CDCl_3$, δ 7.26, CD_2Cl_2, δ 5.32, CD_3OD, δ 3.31, $(CD_3)_2CO$, δ 2.05, AcOH-d_4, δ 2.04, and DMSO-d_6, δ 2.50). Carbon chemical shifts are expressed in parts per million (ppm, δ scale) and are referenced to the carbon resonance of the NMR solvent ($CDCl_3$, δ 77.16, CD_2Cl_2, δ 53.50, CD_3OD, δ 49.00, $(CD_3)_2CO$, δ 29.84, AcOH-d_4, δ 20.00, and DMSO-d_6, δ 39.52). Deuteration yields were determined by ^{1}H NMR using the following internal NMR standard [dibromomethane (2H, 4.93 ppm)] and [trichloroethylene (1H, 6.46 ppm)], which was added to reaction mixtures after cooling to 25 °C or isolated products. The liquid chromatography-mass spectroscopy (LC-MS) were acquired on high-resolution mass spectrometers: Q-TOF (ionization mode: ESI). The gas chromatography-mass spectroscopy (GC-MS) are recorded by using an Agilent 5977B GC/MSD (ionization mode: EI). All commercial reagents and solvents were purchased from Sigma-Aldrich, Alfa Aesar, TCI, BLDpharm, or Combi-Blocks. They were used as received unless otherwise indicated. 4,5-Diphenylpyrazolo[1,5-*a*][1,8]naphthyridine (**PzNPy1**),[1] 6-(1*H*-pyrazol-1-yl)pyridin-2(1*H*)-one (**PzPyOH**),[2] 6-(3-methyl-1*H*-pyrazol-1-yl)pyridin-2(1*H*)-one (**3-Me-PzPyOH**),[2] and [2,2'-bipyridin]-6(1*H*)-one (**BpyOH**)[3] were prepared according to the procedures in the literature.

III. Experimental Procedures

General Procedures for the Deuteration Reactions

General Procedure A (BpyOH). $Pd(OAc)_2$ (4.5 mg, 0.020 mmol), **BpyOH** (3.4 mg, 0.020 mmol), and HFIP (21 μL, 0.20 mmol) were added to a solution of a substrate (0.20 mmol) and AcOH-d_4 (1.0 mL, 0.20 M) in an 8 mL-glass vial. The reaction mixture was stirred in a preheated reaction block at 130 °C. After stirring for 24 h, the reaction mixture was cooled to 25 °C and concentrated. The residue was purified by flash column chromatography to afford the desired product.

General Procedure B (BpyOH, 48 h). $Pd(OAc)_2$ (4.5 mg, 0.020 mmol), **BpyOH** (3.4 mg, 0.020 mmol), and HFIP (21 μL, 0.20 mmol) were added to a solution of a substrate (0.20 mmol) and AcOH-d_4 (1.0 mL, 0.20 M) in an 8 mL-glass vial. The reaction mixture was stirred in a preheated reaction block at 130 °C. After stirring for 48 h, the reaction mixture was cooled to 25 °C and concentrated. The residue was purified by flash column chromatography to afford the desired product.

General Procedure C (3-Me-PzPyOH). $Pd(OAc)_2$ (4.5 mg, 0.020 mmol), **3-Me-PzPyOH** (3.5 mg, 0.020 mmol), and HFIP (21 μL, 0.20 mmol) were added to a solution of a substrate (0.20 mmol) and AcOH-d_4 (1.0 mL, 0.20 M) in an 8 mL-glass vial. The reaction mixture was stirred in a preheated reaction block at 130 °C. After stirring for 24 h, the reaction mixture was cooled to 25 °C and concentrated. The residue was purified by flash column chromatography to afford the desired product.

General Procedure D ($Bpy(OH)_2$). $Pd(OAc)_2$ (4.5 mg, 0.020 mmol), **$Bpy(OH)_2$** (3.8 mg, 0.020 mmol), and HFIP (21 μL, 0.20 mmol) were added to a solution of a substrate (0.20 mmol) and AcOH-d_4 (1.0 mL, 0.20 M) in an 8 mL-glass vial. The reaction mixture was stirred in a preheated reaction block at 130 °C. After stirring for 24 h, the reaction mixture was cooled to 25 °C and concentrated. The residue was purified by flash column chromatography to afford the desired product.

Determination of Deuteration Degrees by ^1H NMR Spectroscopy

The NMR analysis was performed by measuring the decrease of the proton signal intensity, which was compared with ^1H NMR spectra of non-deuterated starting materials. Signal decrease was

calibrated based on the positions with internal NMR standard [dibromomethane (2H, 4.93 ppm)], which was added to reaction mixtures after cooling to 25 °C or products.

Determination of Deuteration Degrees by Mass Spectrometry

Liquid chromatography-mass spectroscopy (LC-MS) were acquired on high-resolution mass spectrometers: Q-TOF (ionization mode: ESI). With the obtained experimental data, the mass spectrometric analysis was performed by using the automated program, Universal Mass Calculator of the University of Münster (UMC Version 3.11.0.70, Dr. Matthias C. Letzel, WWU Münster, Org.-Chem. Institut, Germany. https://www.uni-muenster.de/Chemie.oc/ms/downloads.html). Relative m/z intensities are expressed as abundance and % abundance for ESI. The top row of the diagrams displays the measured m/z values and experimentally determined relative intensities of the isolated compounds, while the bottom row displays theoretically generated mass diagrams for selected degrees of deuteration considering the natural abundance of isotopes.

The gas chromatography-mass spectrometry (GC-MS) data were recorded using an Agilent 5977B GC/MSD (ionization mode: EI). With the obtained experimental data, the deuteration degrees were calculated using the reported methods by Gao et al.[4]

Determination of Yields

The yield was calculated by dividing the weight of the product by the molecular weight of the deuterated product containing deuterium at the positions where H/D exchange occurs more than 50%.

IV. Optimization Studies
IV. A. Ligand Effects

Table S1. Deuteration of **35**[a]

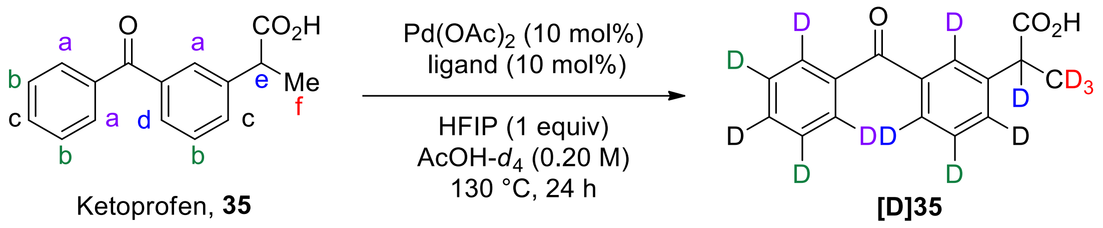

entry	ligand	D incorporation (%)[b]						yield (%)[c]
		a	b	c	d	e	f	
1	–	27	27	41	20	28	18	–
2	**PzNPy1**	37	18	30	34	49	19	–
3	**PyOH**	39	39	55	28	42	29	
4	**Ac-Gly-OH**	26	30	42	21	28	19	–
5	**PzPyOH**	60	86	80	74	43	28	–
6	**3-Me-PzPyOH**	77	94	90	89	76	75	42
7	**BpyOH**	88	91	93	93	57	82	94
8	**Bpy(OH)₂**	68	76	81	73	60	93	86
9[d]	**BpyOH**	82	97	96	88	27	87	77
10[e]	**BpyOH**	88	92	92	92	50	76	79
11[d,e]	**BpyOH**	91	98	98	95	53	95	65
12[f]	**BpyOH**	92	93	94	94	69	86	80
13[g]	**BpyOH**	81	89	90	89	40	75	–
14[h]	**BpyOH**	0	0	0	0	0	0	–

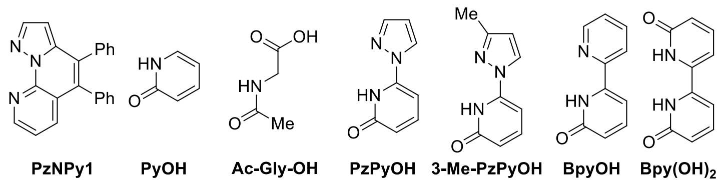

[a] Reaction conditions: **33** (0.2 mmol), $Pd(OAc)_2$ (0.02 mmol), ligand (0.02 mmol), HFIP (0.2 mmol), AcOH-d_4 (0.2 M), and 130 °C. [b] By ^{1}H NMR. [c] Isolated yields. [d] Without HFIP. [e] With D_2O instead of AcOH-d_4. [f] For 48 h. [g] 5 mol% of $Pd(OAc)_2$ and **BpyOH** were used. [h] Without $Pd(OAc)_2$.

IV. B. Additional Deuteration Methods

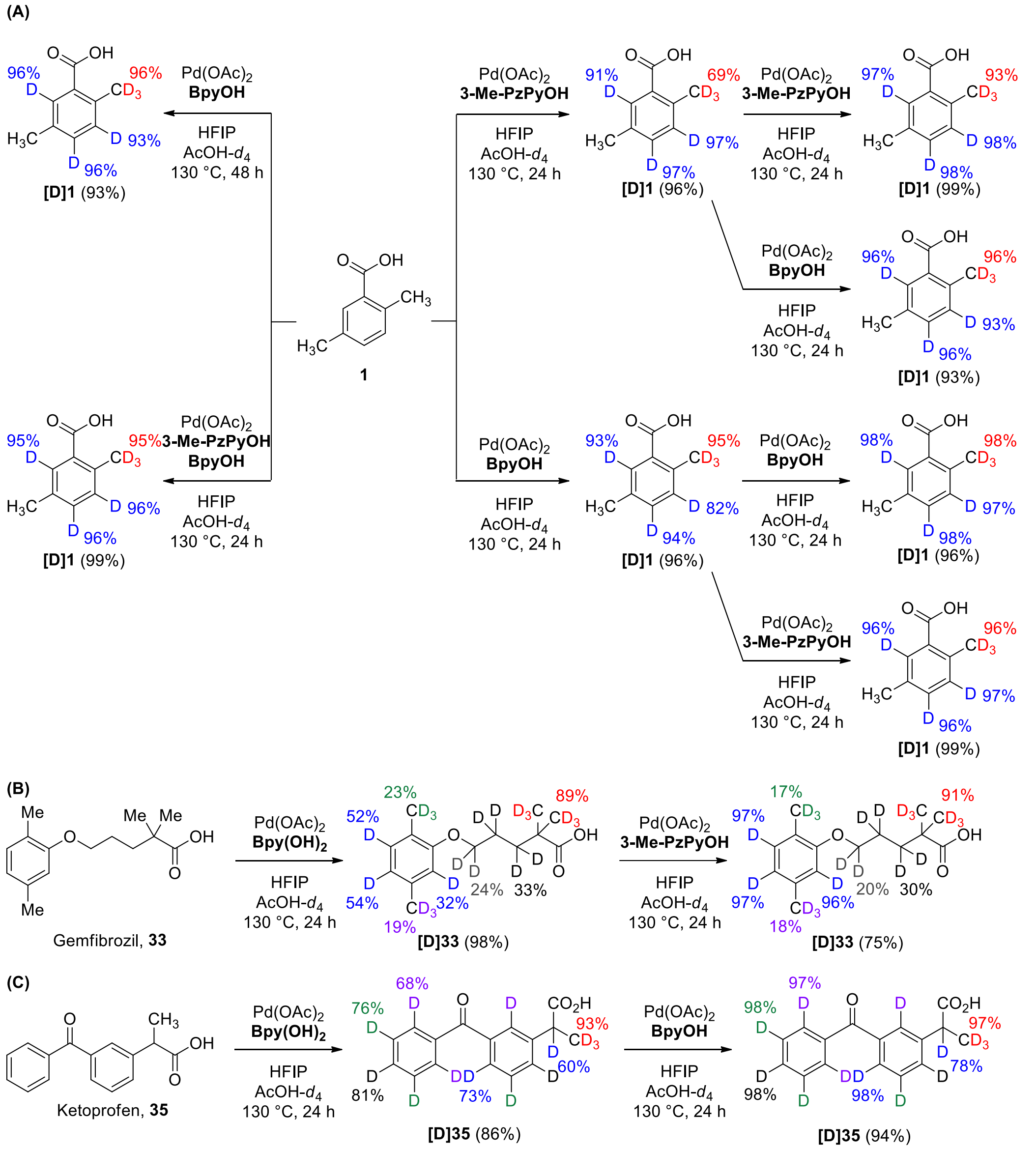

Scheme S1. Approaches for the enhancement of deuterium enrichment of (A) **1**, (B) **33**, and (C) **35**

IV. C. Comparison of Deuteration Methods

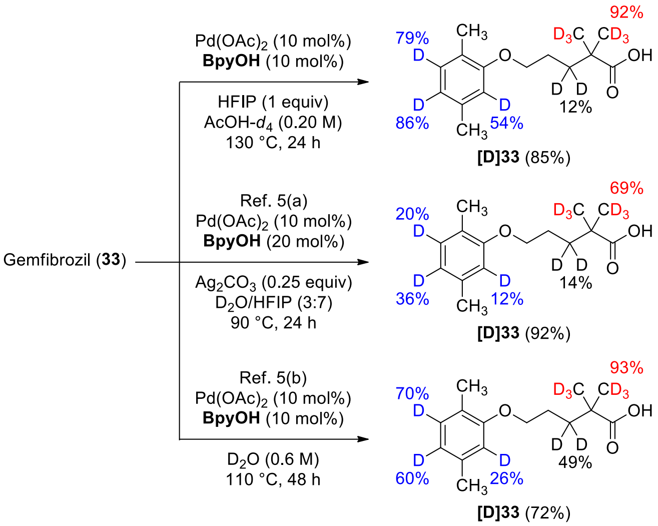

Scheme S2. Deuteration of **33** using **BpyOH** under previously reported conditions[5]

IV. D. Additional Substrate Scopes

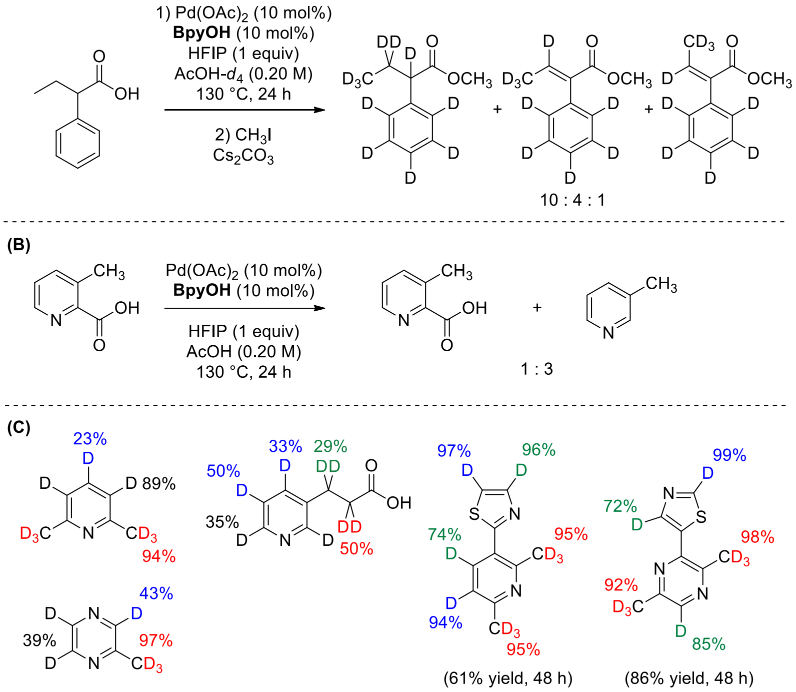

Scheme S3. Deuteration of (A) 2-phenylbutanoic acid, (B) 3-methylpicolinic acid, and (C) 6-membered heteroarene derivatives

When 2-phenylbutanoic acid, a phenylacetic acid containing an ethyl group at the α-position, was subjected to the standard reaction conditions, dehydrogenation occurred as reported (Scheme S3A).[6] In addition, decarboxylation of picolinic acid derivatives has been reported,[7] making these derivatives unsuitable for Pd-catalyzed deuteration (Scheme S3B). Decarboxylation of other heteroaromatic acids was negligible, as demonstrated by high isolated yields and separate experiments performed in the absence of D source. Methyl groups of six-membered heteroarenes, including pyridine and pyrazine, are activated and readily undergo deuteration without the requirement of directing groups (Scheme S3C).[8] These results also suggest that six-membered heteroarene substrates do not hinder the reaction through potential coordination via the Lewis basic nitrogen atom, while their sp^2 positions are less reactive in deuteration compared to those of five-membered heteroarenes.

IV. E. D Incorporation of BpyOH

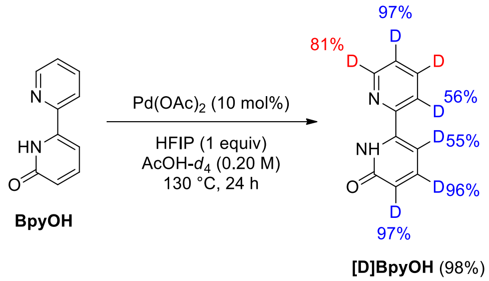

Scheme S4. Deuteration of **BpyOH**

$Pd(OAc)_2$ (4.5 mg, 0.020 mmol), and HFIP (21 µL, 0.20 mmol) were added to a solution of a [2,2'-bipyridin]-6(1*H*)-one (34.4 mg, 0.20 mmol) and AcOH-d_4 (1.0 mL, 0.20 M) in an 8 mL-glass vial. The reaction mixture was stirred in a preheated reaction block at 130 °C. After stirring for 24 h, the reaction mixture was cooled to 25 °C and concentrated. Purification by flash column chromatography (EtOAc/HCO_2H = 1:0.01) provided product **[D]BpyOH** as a yellow solid (35 mg, 98% yield).

Deuterium Incorporation

[GCMS (EI)] calcd for $C_{10}HD_7N_2O$ $[M]^+$ 5.28 D/molecule, [^{1}H NMR] 5.63 D/molecule.

NMR Data of the Starting Material

^{1}H NMR (500 MHz, $CDCl_3$) δ 10.71 (s, 1H), 8.65 (d, J = 4.7 Hz, 1H), 7.82 (d, J = 4.2 Hz, 2H), 7.53 -7.46 (m, 1H), 7.36 (q, J = 4.5 Hz, 1H), 6.80 (d, J = 6.9 Hz, 1H), 6.64 (d, J = 9.2 Hz, 1H).

NMR Data of the Product

^{1}H NMR (500 MHz, $CDCl_3$) δ 8.67-8.63 (m, 0.44H, 56% D), 7.82 (s, 0.38H, 81% D), 7.49 (s, 0.45H, 55% D), 7.39-7.34 (m, 0.04H, 96% D), 6.83-6.79 (m, 0.03H, 97% D), 6.66-6.62 (m, 0.03H, 97% D); ^{13}C NMR (126 MHz, $CDCl_3$) δ 163.0, 149.3, 147.9, 141.8, 140.5, 137.3, 124.6-124.2 (1C), 122.2-121.8 (1C), 119.7-119.3 (1C), 102.8-102.4 (1C).

Mass Data

	M+2	M+3	M+4	M+5	M+6	M+7	M+8
m/z	174	175	176	177	178	179	180
Abound	18608	127639	5601	863962	553085	151015	16934
Theoretical exact mass of start material: 172.0637							
Weighted average of deuterated product: 177.3392							
Average %D: 75%							

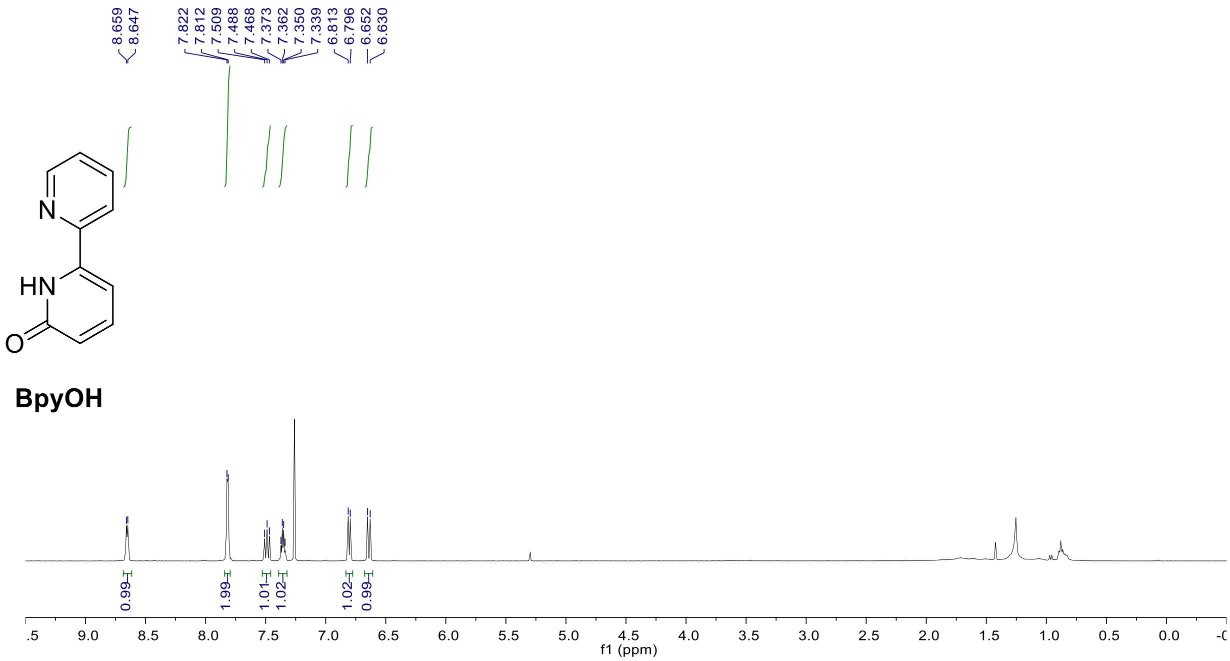

^{1}H NMR spectrum of compound **BpyOH** ($CDCl_3$, 500 MHz)

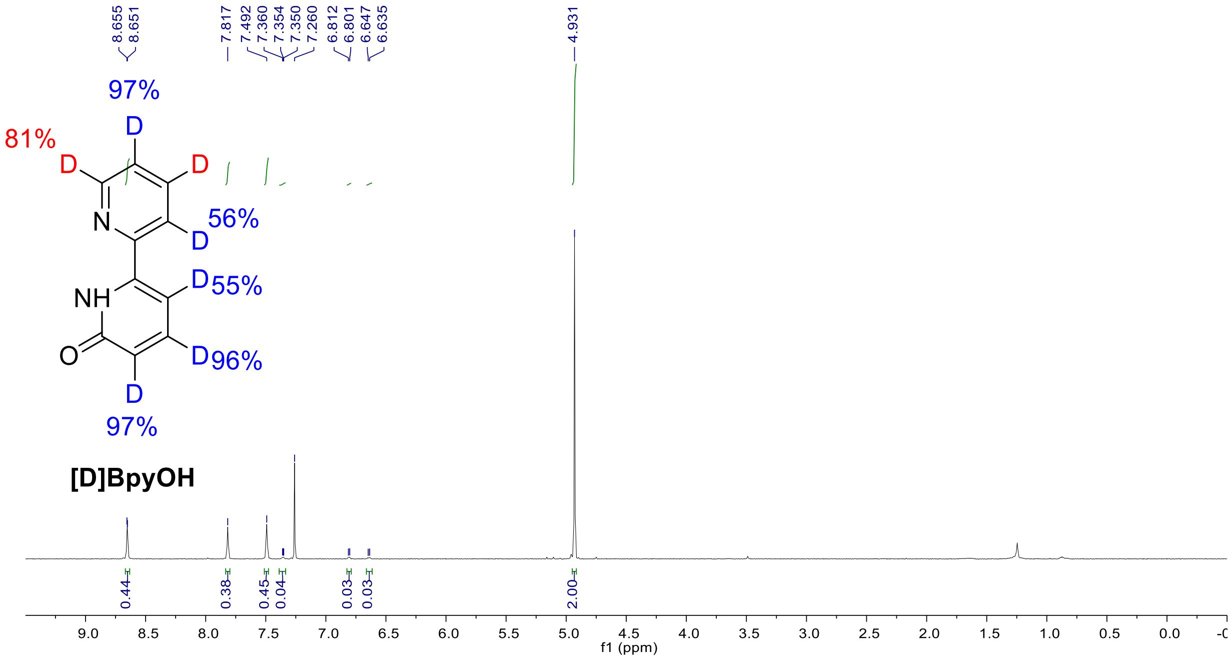

^{1}H NMR spectrum of compound **[D]BpyOH** ($CDCl_3$, 500 MHz)

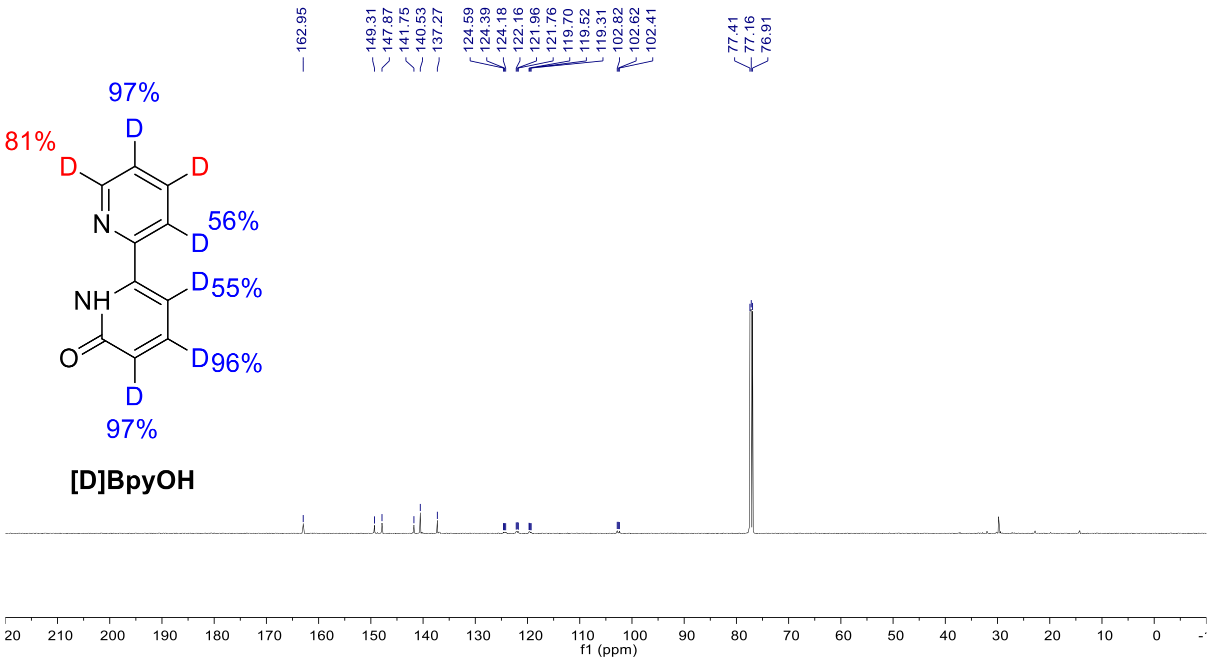

^{13}C NMR spectrum of compound **[D]BpyOH** ($CDCl_3$, 126 MHz)

V. H/D Exchange Experiments

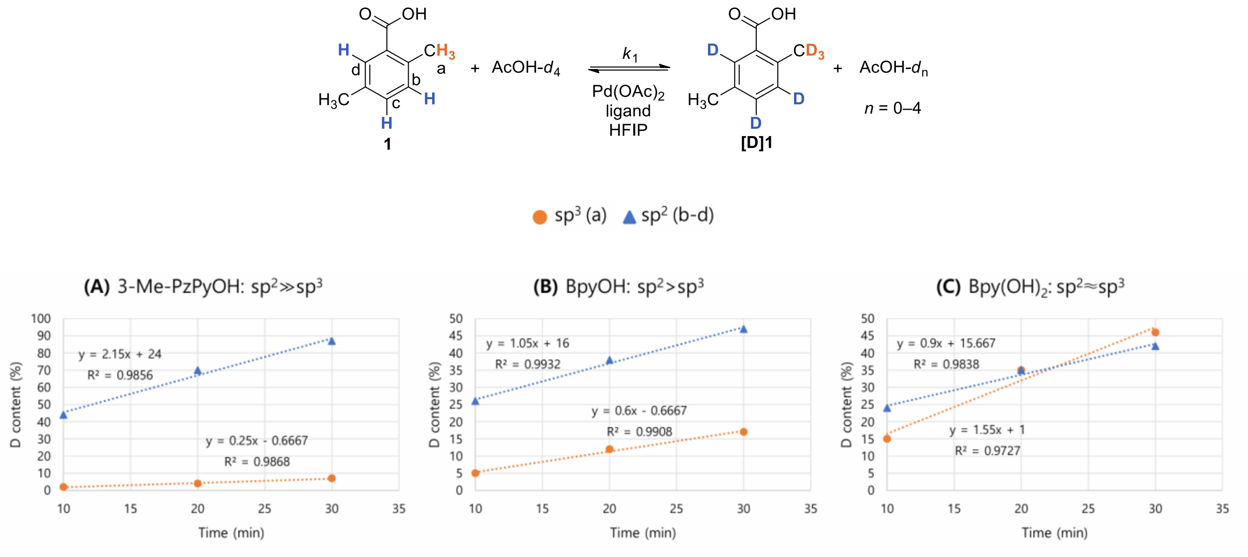

Fig. S1. H/D exchange of the directed sp^3 and nondirected sp^2 positions of **1** with ligands (A) **3-Me-PzPyOH**, (B) **BpyOH**, and (C) **Bpy(OH)$_2$**.

To elucidate the ligand effect on deuteration, H/D exchange at the *ortho*-methyl and aromatic positions of 2,5-dimethylbenzoic acid (**1**) was monitored (Fig. S1). C–H activation processes are reversible, requiring consideration of both intermediates and transition states.[9] In terms of intermediates, directed sp^3 and nondirected sp^2 pathways involve two distinct types: one involving a directing group-bound Pd complex and the other lacking such an interaction. Therefore, nondirected aromatic positions that share the latter type of intermediate were considered collectively. Conversion was measured over the same time period for all positions to enable a clear comparison. When **3-Me-PzPyOH** was used, aromatic positions were deuterated much faster than the methyl position (Fig. S1A). However, the difference in reaction rates was reduced when **BpyOH** was used (Fig. S1B), whereas similar reaction progresses were observed for both positions with **Bpy(OH)$_2$** (Fig. S1C).

Table S2. D incorporation into **1** (average of two runs)

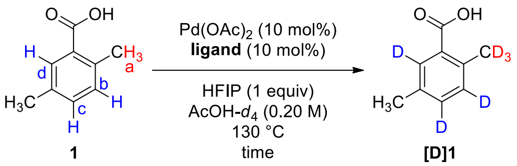

entry	time (min.)	D content (%)											
		**3-Me-PzPyOH**				**BpyOH**				**Bpy(OH)₂**			
		a	b	c	d	a	b	c	d	a	b	c	d
1	10	2	13	16	15	5	6	10	10	15	5	8	11
2	20	4	23	27	20	12	8	14	16	35	10	13	12
3	30	7	29	33	25	17	10	19	18	46	12	17	13

VI. Compound Characterization Data

2,5-dimethylbenzoic acid (1)

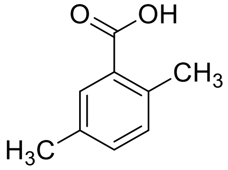

Following the general procedure A, the reaction was set up with 2,5-dimethylbenzoic acid (30.0 mg, 0.20 mmol). Purification by flash column chromatography (hexanes/EtOAc/HCO_2H = 10:1:0.1) provided product **[D]1** as a white solid (30 mg, 96% yield).

Following the general procedure C, **[D]1** was provided as a yellow solid (30 mg, 96% yield).

Following the general procedure D, **[D]1** was provided as a white solid (28 mg, 90% yield).

Deuterium Incorporation

General procedure A: [LCMS (ESI)] calcd for $C_9H_3D_6O_2$ $[M-H]^-$ 5.50 D/molecule, [^{1}H NMR] 5.54 D/molecule.

General procedure D: [GCMS (EI)] calcd for $C_9H_4D_6O_2$ $[M]^+$ 5.75 D/molecule, [^{1}H NMR] 5.73 D/molecule.

NMR Data of the Starting Material

^{1}H NMR (500 MHz, CD_2Cl_2) δ 7.86 (s, 1H), 7.29 (dd, *J* = 7.8, 2.0 Hz, 1H), 7.18 (d, *J* = 7.7 Hz, 1H), 2.59 (s, 3H), 2.36 (s, 3H).

NMR Data of the Product

General procedure A: ^{1}H NMR (500 MHz, CD_2Cl_2) δ 7.87 (s, 0.07H, 93% D), 7.29 (s, 0.06H, 94% D), 7.19 (s, 0.18H, 82% D), 2.56 (s, 0.15H, 95% D), 2.36 (s, 2.95H, 2% D); ^{13}C NMR (126 MHz, $CDCl_3$) δ 176.1, 140.3-140.1 (1C), 136.36-136.26 (1C), 129.3, 129.0, 128.8-128.5 (2C), 21.3-21.2 (1C), 20.2-19.4 (1C).

General procedure C: ^{1}H NMR (500 MHz, CD_2Cl_2) δ 7.86 (s, 0.09H, 91% D), 7.29 (s, 0.03H, 97% D), 7.18 (s, 0.03H, 97% D), 2.59-2.54 (m, 0.92H, 69% D), 2.36 (s, 3H).

General procedure D: ^{1}H NMR (500 MHz, CD_2Cl_2) δ 7.85 (s, 0.10H, 90% D), 7.29 (s, 0.03H, 97% D), 7.18 (s, 0.05H, 95% D), 2.59-2.53 (m, 0.10H, 97% D), 2.36 (s, 3.31H, 0% D).

Mass Data

General procedure A

LabelChecker Results

Formula: C9 H9 O2
Mass (monoisotopic): 149.06

Difference Value: 0.000029
Error Sum: 0.005
Error (%): 0.073

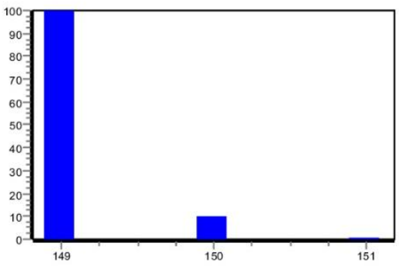

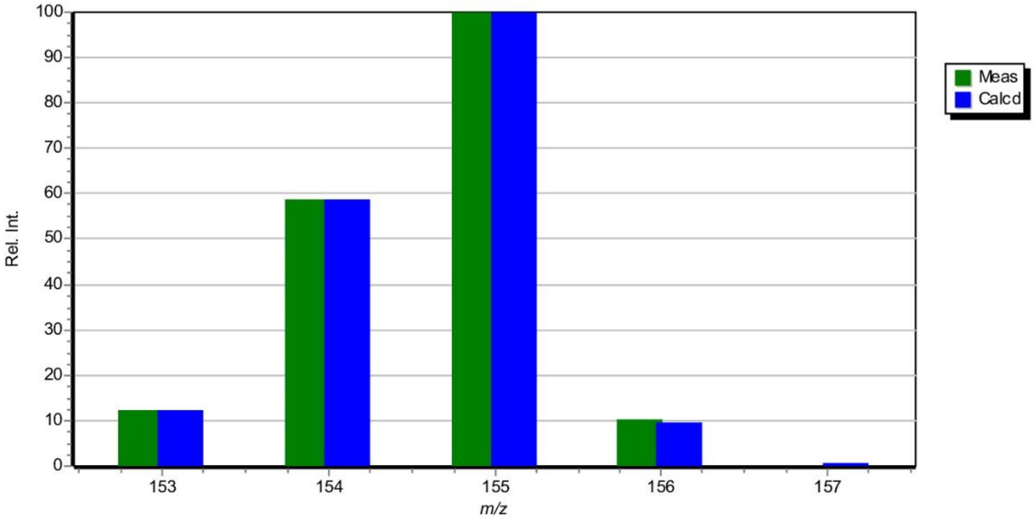

Deuterium: 0-fold (%): 0.00 0.00
Deuterium: 1-fold (%): 0.07 0.04
Deuterium: 2-fold (%): 0.00 0.00
Deuterium: 3-fold (%): 0.01 0.00
Deuterium: 4-fold (%): 13.10 7.52
Deuterium: 5-fold (%): 61.13 35.07
Deuterium: 6-fold (%): 100.00 57.37
Label Atom Sum: 5.50 (61.08%)

Isotope List used for fitting data:

m/z intensity
153.08 1273801
154.09 6068319
155.10 10318724
156.10 1049926
157.10 49753

General procedure D

	M+4	M+5	M+6	M+7
m/z	154	155	156	157
Abound	10599	50973	178452	19115
Theoretical exact mass of start material: 150.0681				
Weighted average of deuterated product: 155.7953				
Average %D: 96%				

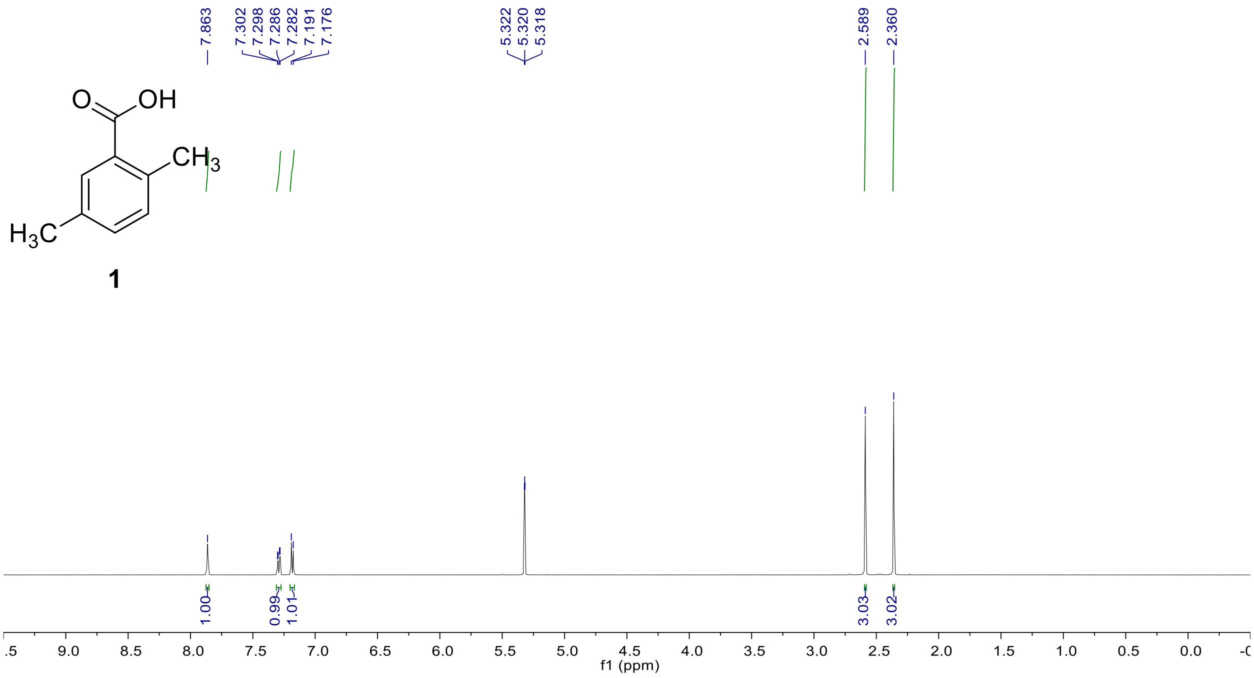

^{1}H NMR spectrum of compound **1** (CD_2Cl_2, 500 MHz)

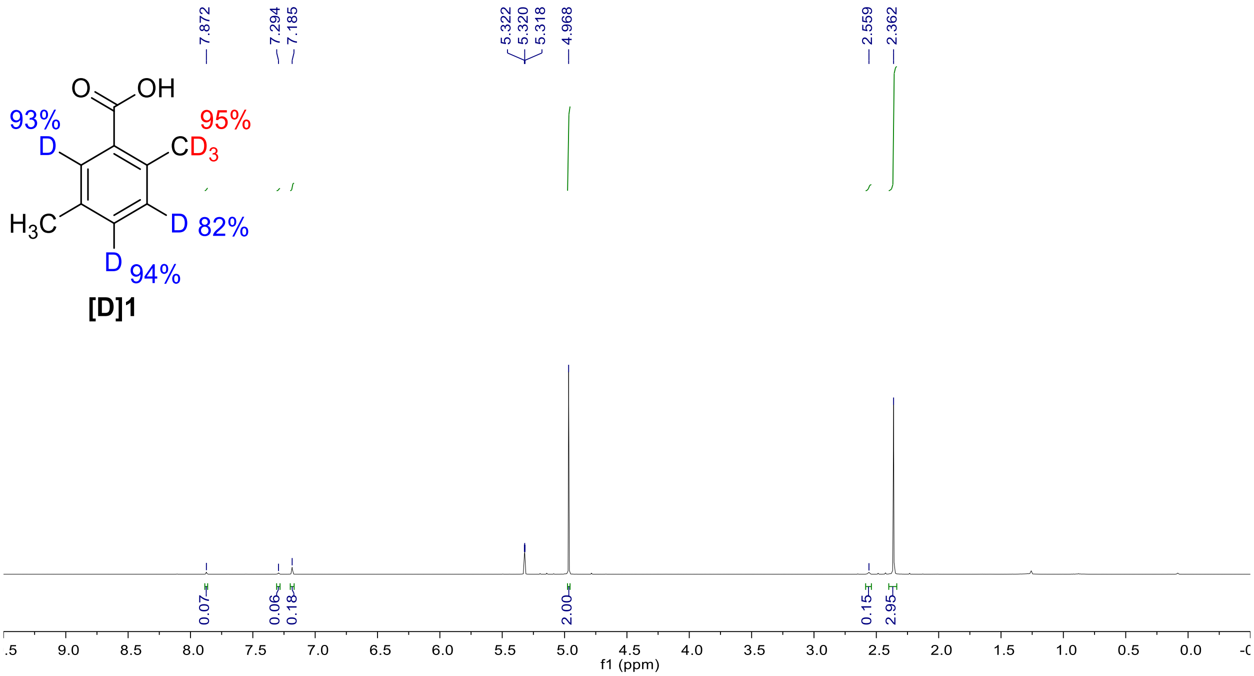

^{1}H NMR spectrum of compound **[D]1** (Procedure A, CD_2Cl_2, 500 MHz)

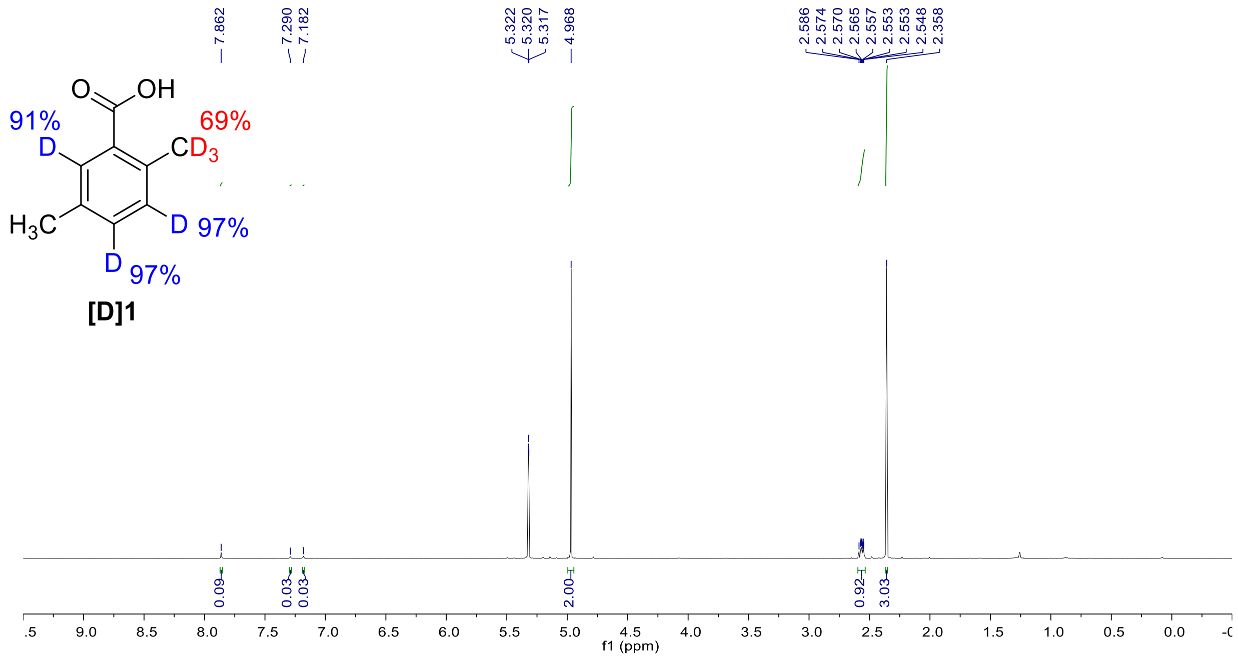

^{1}H NMR spectrum of compound **[D]1** (Procedure C, CD_2Cl_2, 500 MHz)

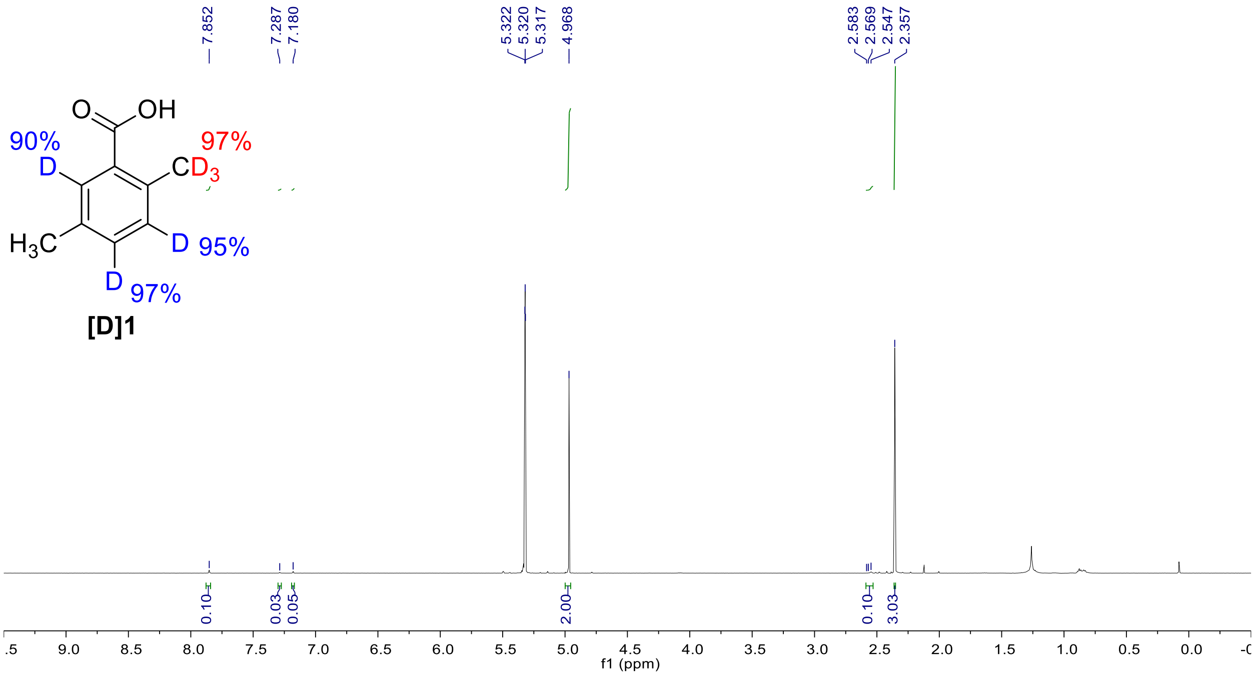

^{1}H NMR spectrum of compound **[D]1** (Procedure D, CD_2Cl_2, 500 MHz)

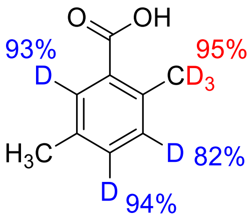

[D]1

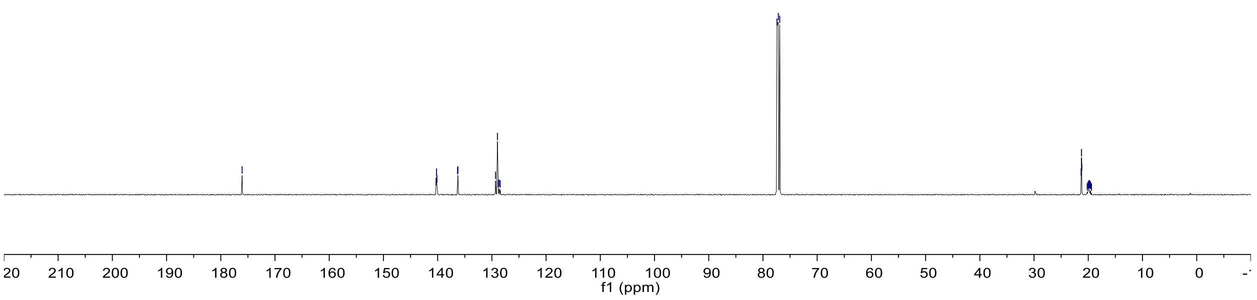

^{13}C NMR spectrum of compound **[D]1** (Procedure A, $CDCl_3$, 126 MHz)

4-bromo-2,6-dimethylbenzoic acid (2)

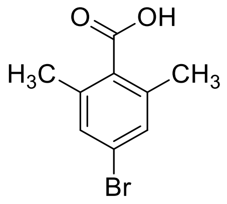

Following the general procedure A, the reaction was set up with 4-bromo-2,6-dimethylbenzoic acid (45.6 mg, 0.20 mmol). Purification by flash column chromatography hexanes/EtOAc/HCO_2H = 8:1:0.1) provided product **[D]2** as a yellow solid (44 mg, 93% yield).

Following the general procedure C, **[D]2** was provided as a yellow solid (41 mg, 89% yield).

Following the general procedure D, **[D]2** was provided as a white solid (40 mg, 84% yield).

Deuterium Incorporation

General procedure A: [LCMS (ESI)] calcd for $C_9D_8BrO_2$ $[M-H]^-$ 7.44 D/molecule, [^{1}H NMR] 7.66 D/molecule.

NMR Data of the Starting Material

^{1}H NMR (500 MHz, $CDCl_3$) δ 7.24 (s, 2H), 2.40 (s, 6H).

NMR Data of the Product

General procedure A: ^{1}H NMR (500 MHz, $CDCl_3$) δ 7.22 (s, 0.05H, 98% D), 2.40-2.32 (m, 0.30H, 95% D); ^{13}C NMR (126 MHz, $CDCl_3$) δ 174.4, 137.9, 131.5 (2C), 131.0-130.4 (1C), 124.0 (2C), 20.0-19.3 (2C).

General procedure C: ^{1}H NMR (500 MHz, $CDCl_3$) δ 7.25 (s, 0.11H, 95% D), 2.44-2.36 (m, 3.62H, 40% D).

General procedure D: ^{1}H NMR (500 MHz, $CDCl_3$) δ 7.24 (s, 0.13H, 94% D), 2.40-2.35 (m, 0.31H, 95% D);

Mass Data

LabelChecker Results

Formula:	C9 H8 O2 Br
Mass (monoisotopic):	226.97
Difference Value:	0.004663
Error Sum:	0.068
Error (%):	0.483

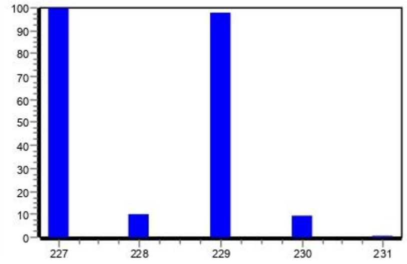

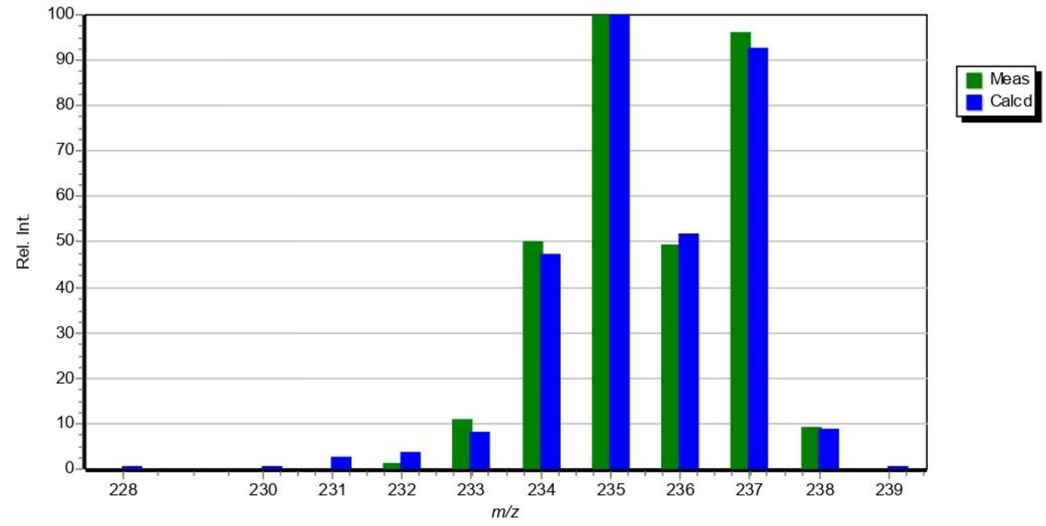

Deuterium: 0-fold (%): 0.21 0.13
Deuterium: 1-fold (%): 0.72 0.44
Deuterium: 2-fold (%): 0.00 0.00
Deuterium: 3-fold (%): 0.00 0.00
Deuterium: 4-fold (%): 3.10 1.92
Deuterium: 5-fold (%): 3.78 2.34
Deuterium: 6-fold (%): 5.78 3.57
Deuterium: 7-fold (%): 48.04 29.72
Deuterium: 8-fold (%): 100.00 61.87
Label Atom Sum: 7.44 (93.04%)

Isotope List used for fitting data:

m/z	intensity
232.00	326697
233.01	2536648
234.01	11684142
235.02	23296528
236.01	11501330
237.02	22391956
238.02	2192303

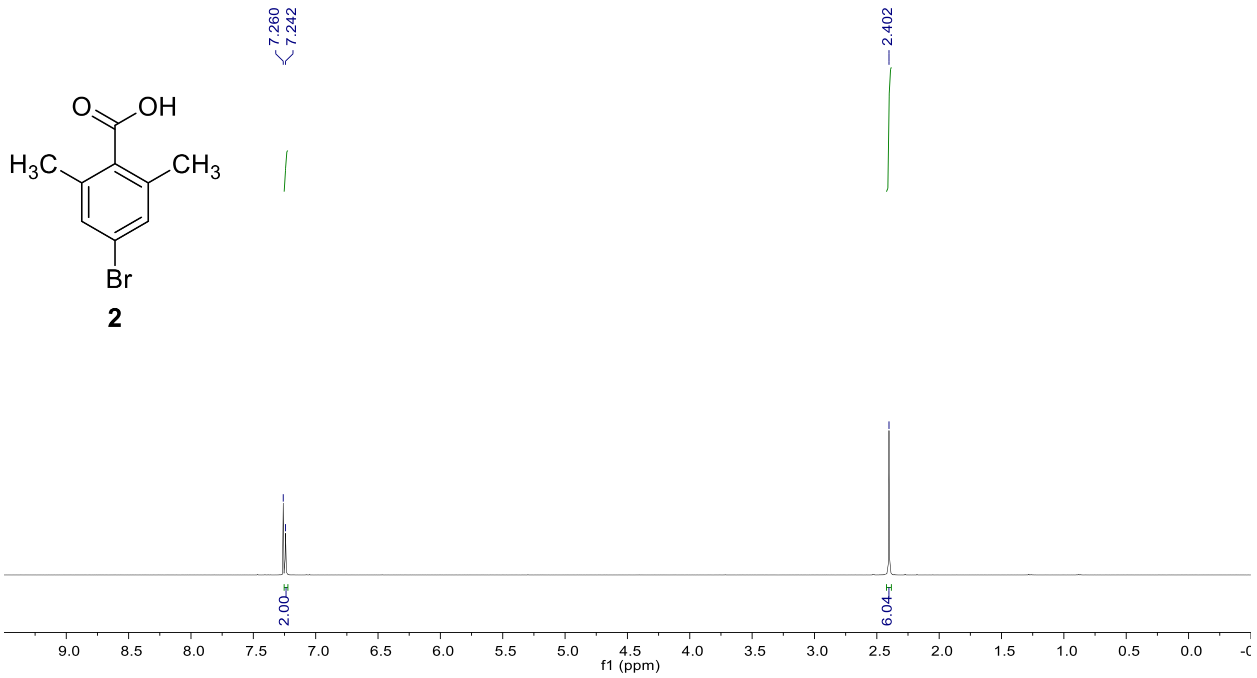

^{1}H NMR spectrum of compound **2** ($CDCl_3$, 500 MHz)

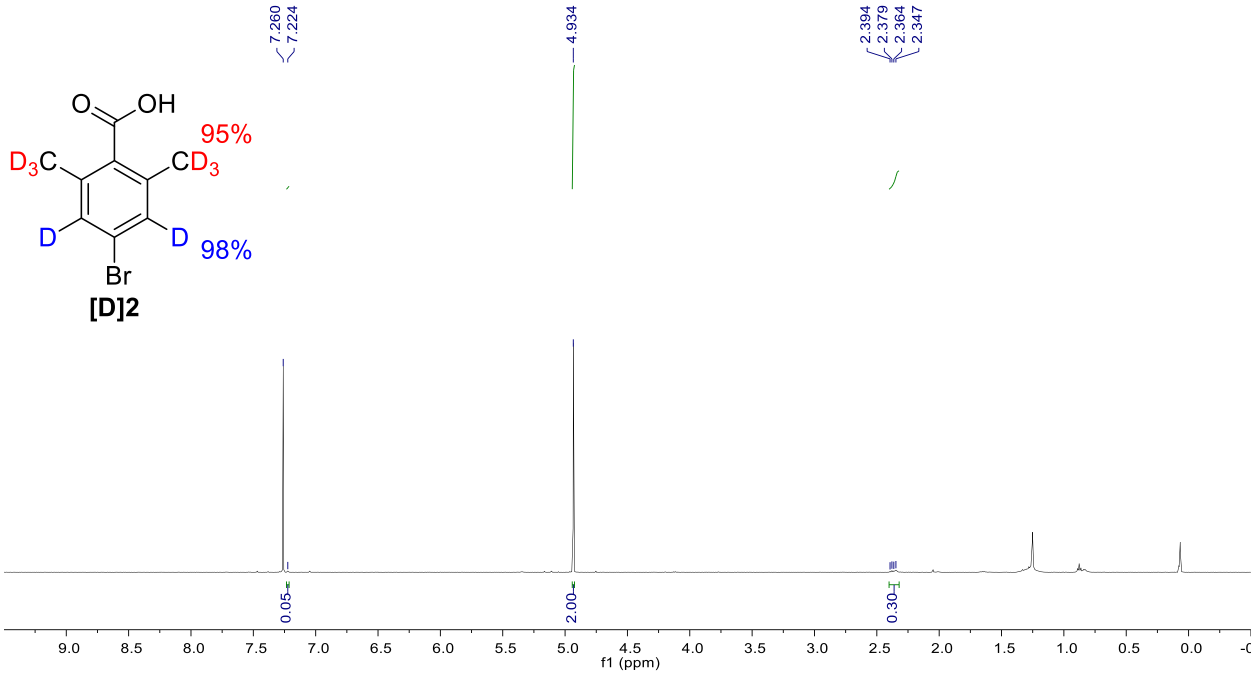

^{1}H NMR spectrum of compound **[D]2** (Procedure A, $CDCl_3$, 500 MHz)

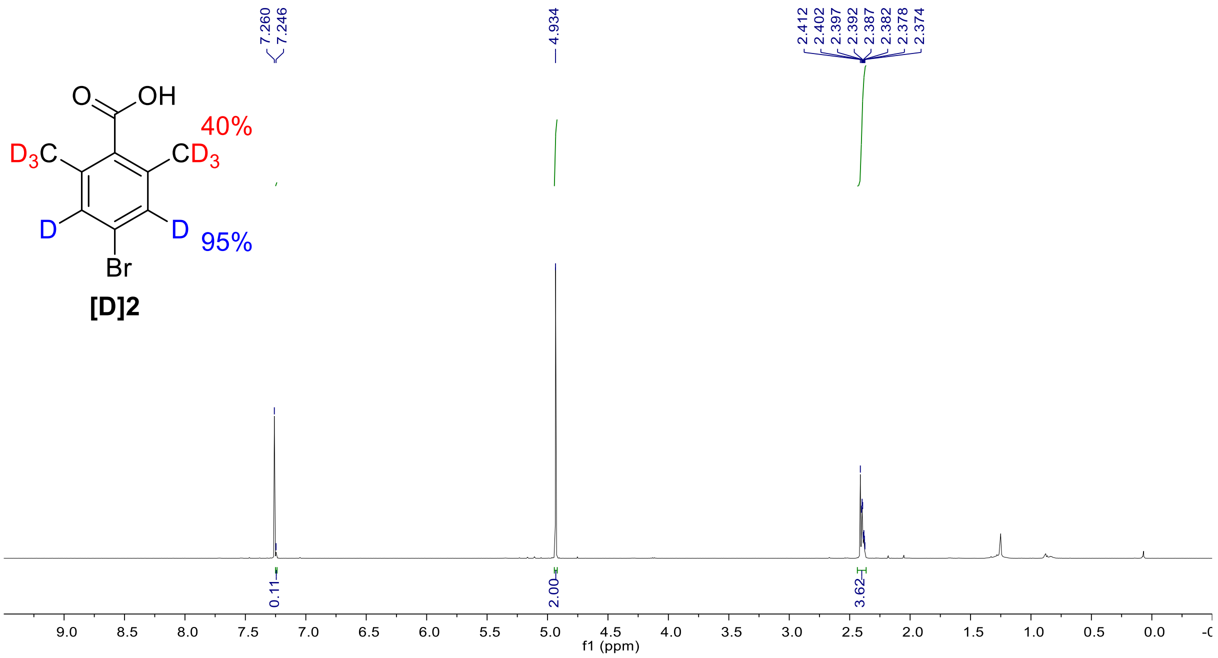

^{1}H NMR spectrum of compound **[D]2** (Procedure C, $CDCl_3$, 500 MHz)

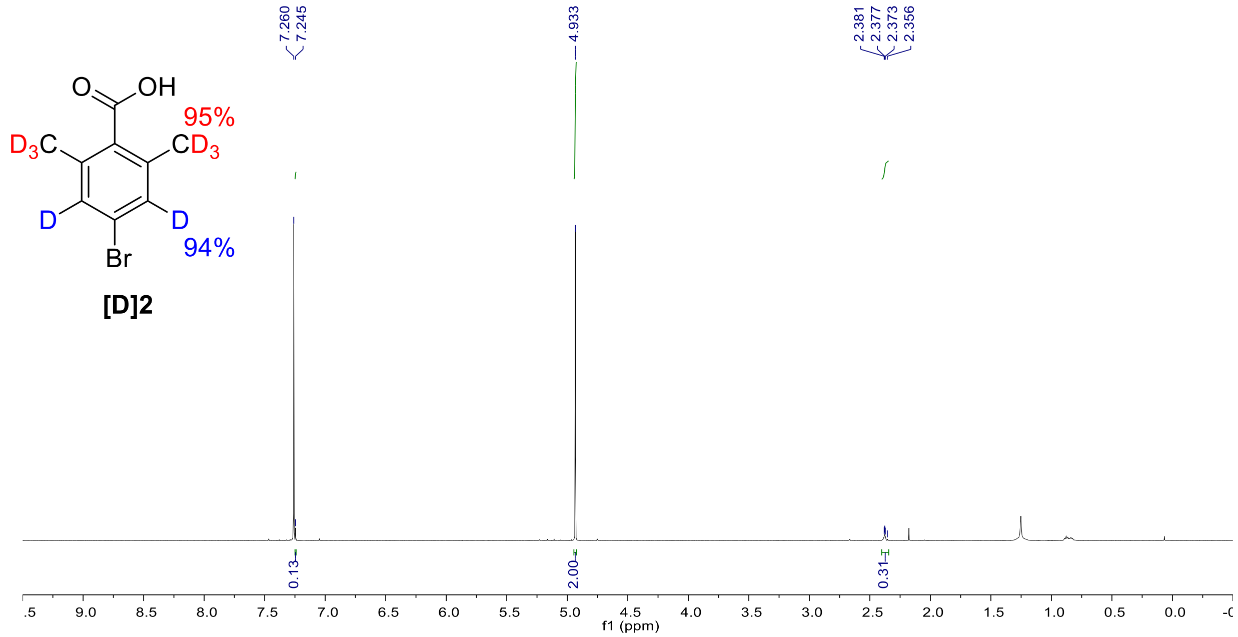

^{1}H NMR spectrum of compound **[D]2** (Procedure D, $CDCl_3$, 500 MHz)

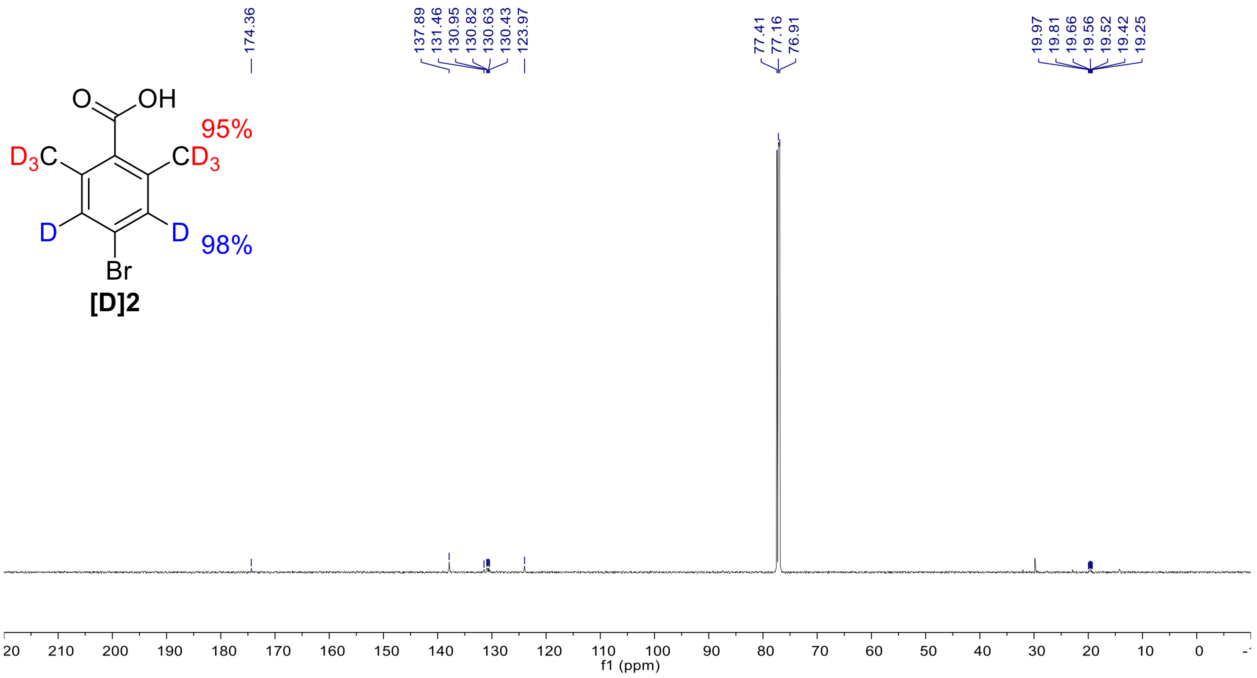

^{13}C NMR spectrum of compound **[D]2** (Procedure A, $CDCl_3$, 126 MHz)

2-methylthiophene-3-carboxylic acid (3)

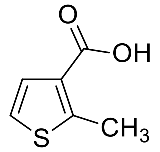

Following the general procedure A, the reaction was set up with 2-methylthiophene-3-carboxylic acid (28.4 mg, 0.20 mmol). Purification by flash column chromatography (hexanes/EtOAc/HCO_2H = 5:1:0.08) provided product **[D]3** as a white solid (26 mg, 90% yield).

Following the general procedure C, **[D]3** was provided as a yellow solid (28 mg, 97% yield).

Following the general procedure D, **[D]3** was provided as a white solid (27 mg, 92% yield).

Deuterium Incorporation

General procedure A: [LCMS (ESI)] calcd for $C_6D_5O_2S$ $[M-H]^-$ 2.68 D/molecule, [^{1}H NMR] 2.57 D/molecule.

General procedure D: [GCMS (EI)] calcd for $C_6D_5O_2S$ $[M]^+$ 3.74 D/molecule, [^{1}H NMR] 4.03 D/molecule.

NMR Data of the Starting Material

^{1}H NMR (500 MHz, $CDCl_3$) δ 7.45 (d, J = 5.4 Hz, 1H), 7.01 (d, J = 5.3 Hz, 1H), 2.77 (s, 3H).

NMR Data of the Product

General procedure A: ^{1}H NMR (500 MHz, $CDCl_3$) δ 7.45 (s, 0.06H, 94% D), 7.01 (s, 0.06H, 94% D), 2.79-2.72 (m, 2.32H, 23% D); ^{13}C NMR (126 MHz, $CDCl_3$) δ 169.4, 151.7-151.6 (1C), 129.7-129.3 (1C), 127.6, 121.3-120.8 (1C), 15.8-15.4 (1C).

General procedure C: ^{1}H NMR (500 MHz, $CDCl_3$) δ 7.44 (s, 0.02H, 98% D), 7.01 (s, 0.02H, 98% D), 2.79-2.74 (m, 2.45H, 18% D).

General procedure D: ^{1}H NMR (500 MHz, $CDCl_3$) δ 7.44 (s, 0.02H, 98% D), 7.01 (s, 0.02H, 98% D), 2.80-2.70 (m, 0.94H, 69% D).

Mass Data

General procedure A

LabelChecker Results

Formula: C6 H5 O2 S
Mass (monoisotopic): 141.00

Difference Value: 0.000498
Error Sum: 0.022
Error (%): 0.560

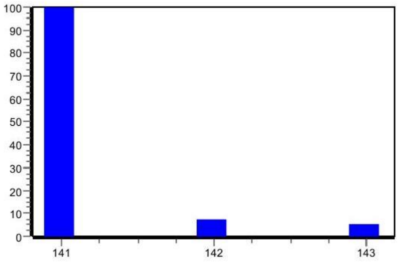

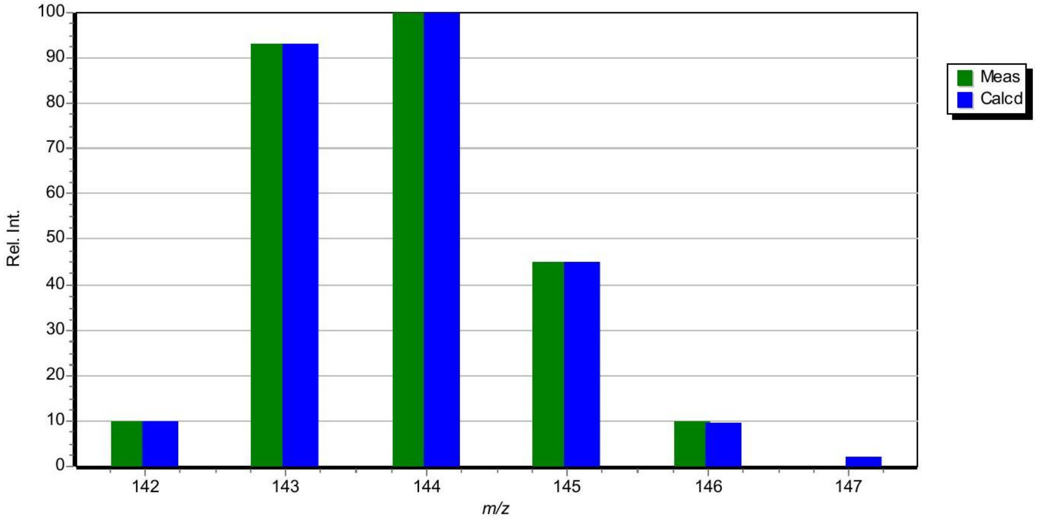

Deuterium: 0-fold (%): 0.00 0.00
Deuterium: 1-fold (%): 10.60 4.26
Deuterium: 2-fold (%): 99.88 40.13
Deuterium: 3-fold (%): 100.00 40.18
Deuterium: 4-fold (%): 36.11 14.51
Deuterium: 5-fold (%): 2.28 0.91
Label Atom Sum: 2.68 (53.54%)

Isotope List used for fitting data:

m/z intensity
142.01 1174204
143.01 11154319
144.02 11960606
145.03 5402470
146.03 1172436

General procedure D

	M+2	M+3	M+4	M+5	M+6
m/z	144	145	146	147	148
Abound	32408	77074	95816	55924	8545
Theoretical exact mass of start material: 142.0089					
Weighted average of deuterated product: 145.7447					
Average %D: 75%					

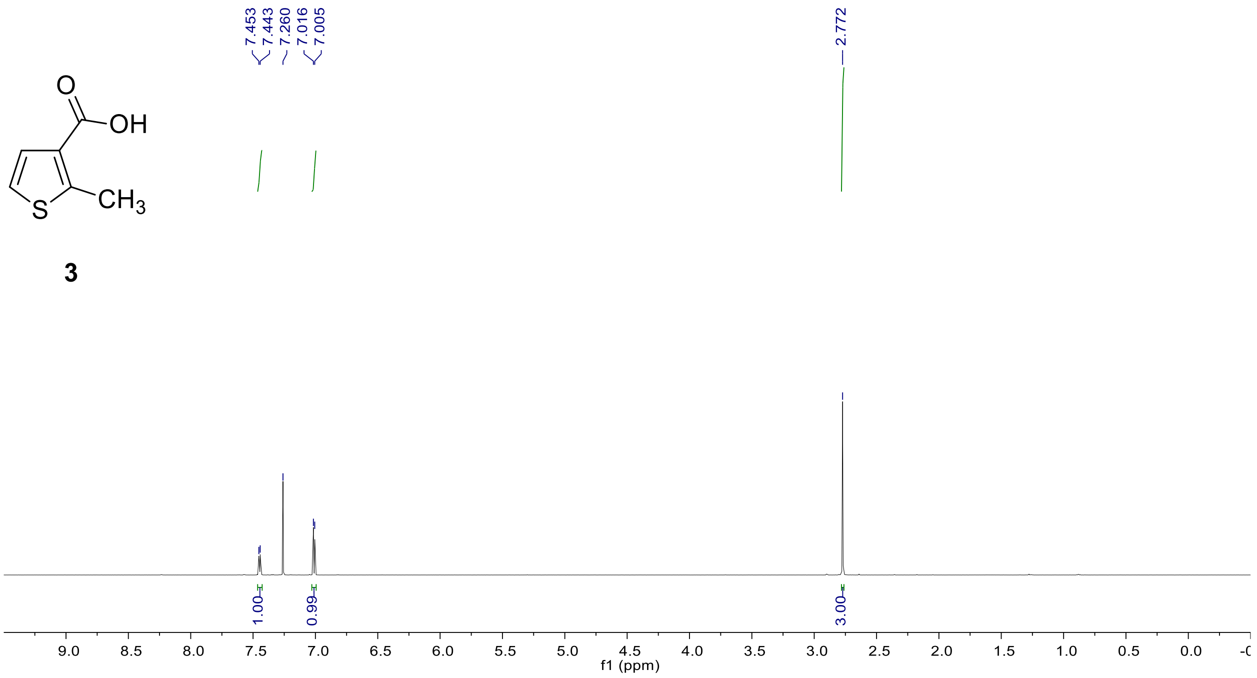

^{1}H NMR spectrum of compound **3** ($CDCl_3$, 500 MHz)

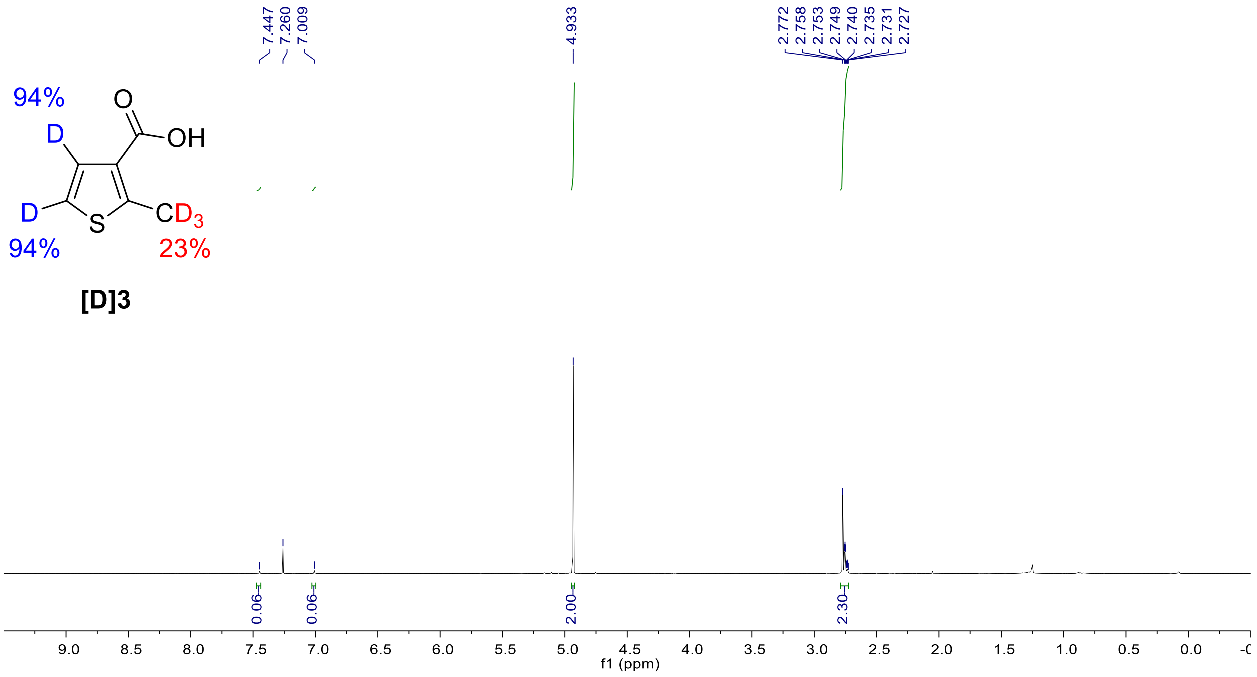

^{1}H NMR spectrum of compound **[D]3** (Procedure A, $CDCl_3$, 500 MHz)

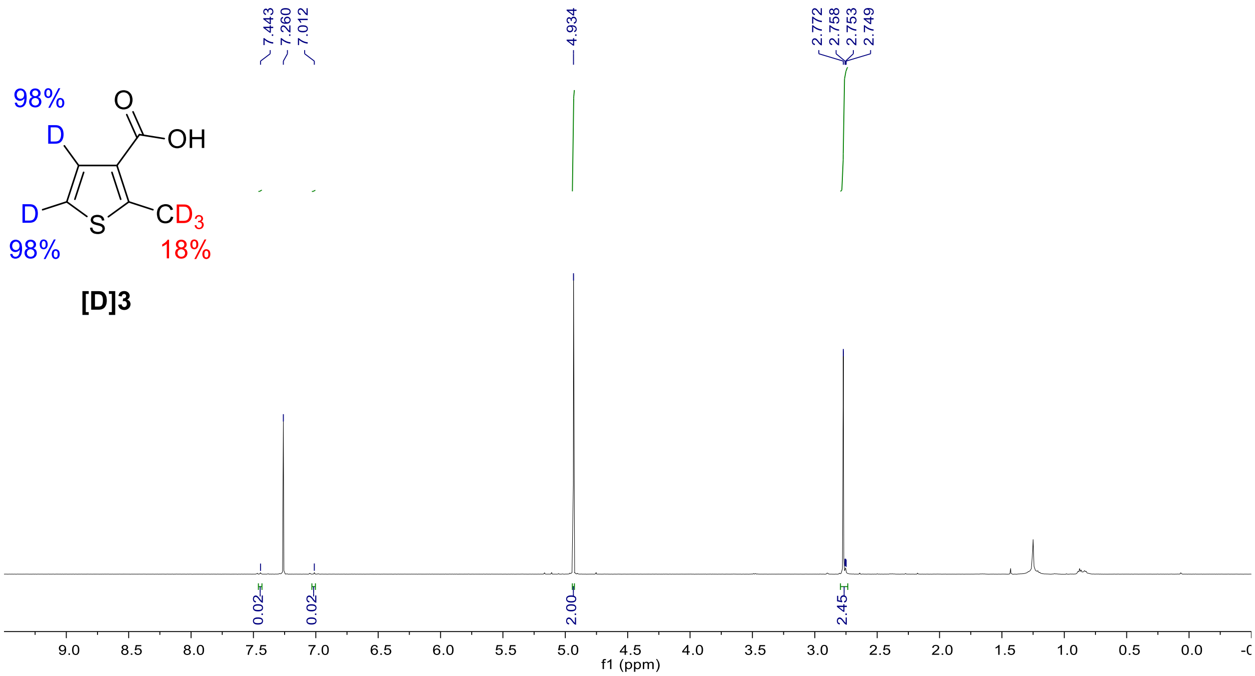

^{1}H NMR spectrum of compound **[D]3** (Procedure C, $CDCl_3$, 500 MHz)

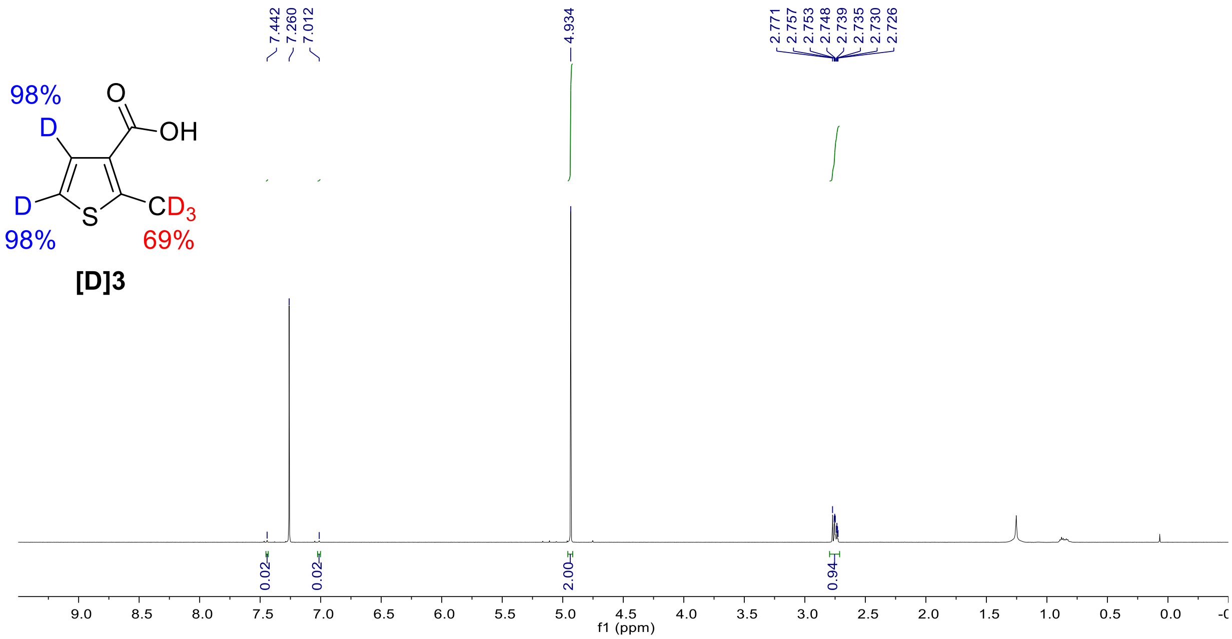

^{1}H NMR spectrum of compound **[D]3** (Procedure D, $CDCl_3$, 500 MHz)

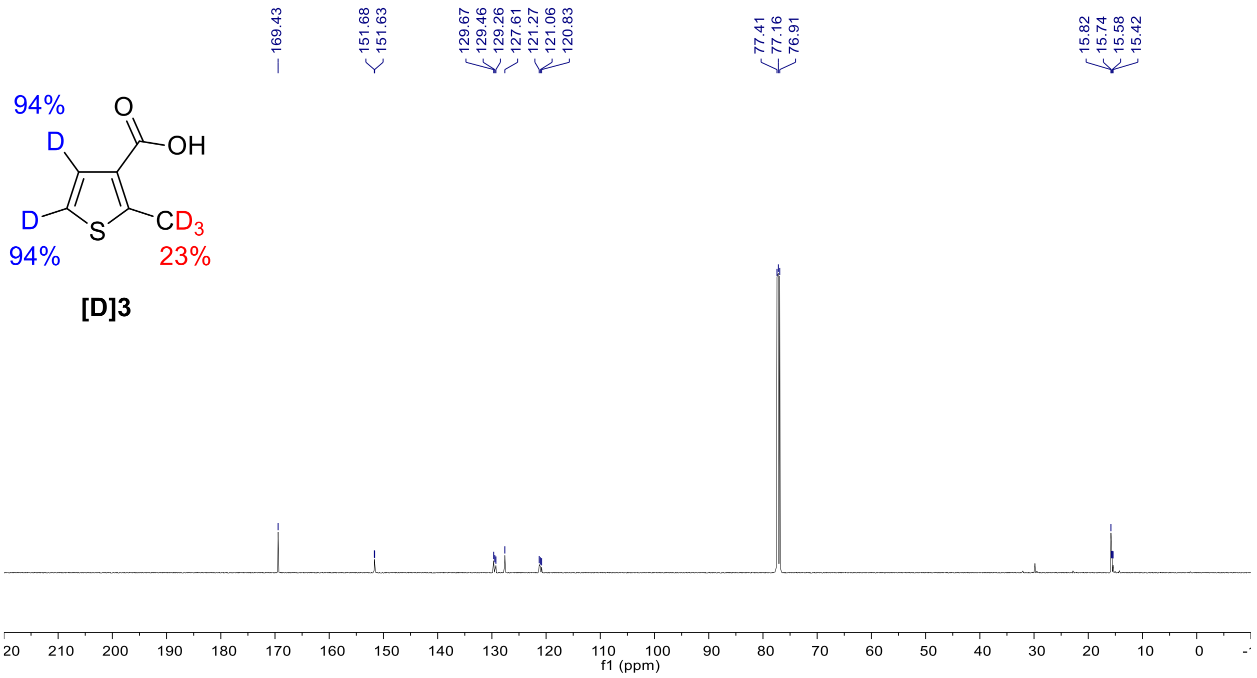

^{13}C NMR spectrum of compound **[D]3** (Procedure A, $CDCl_3$, 126 MHz)

methyl 2-nitrobenzoate (4)

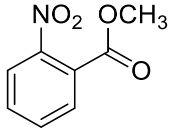

Following the general procedure A, the reaction was set up with methyl 2-nitrobenzoate (36.2 mg, 0.20 mmol). Purification by flash column chromatography (hexanes/EtOAc = 5:1) provided product **[D]4** as a yellow liquid (34 mg, 92% yield).

Following the general procedure C, **[D]4** was provided as a yellow liquid (36 mg, 97% yield).

Following the general procedure D, **[D]4** was provided as a yellow liquid (36 mg, 97% yield).

Deuterium Incorporation

General procedure A: [GCMS (EI)] calcd for $C_8H_3D_4NO_4$ $[M]^+$ 3.80 D/molecule, [^{1}H NMR] 3.74 D/molecule.

NMR Data of the Starting Material

^{1}H NMR (500 MHz, $CDCl_3$) δ 7.91 (dd, J = 7.9, 1.4 Hz, 1H), 7.74 (dd, J = 7.5, 1.6 Hz, 1H), 7.68 (td, J = 7.5, 1.4 Hz, 1H), 7.63 (td, J = 7.7, 1.6 Hz, 1H), 3.92 (s, 3H).

NMR Data of the Product

General procedure A: ^{1}H NMR (500 MHz, $CDCl_3$) δ 7.92 (s, 0.06H, 94% D), 7.75 (s, 0.10H, 90% D), 7.68 (s, 0.05H, 95% D), 7.64 (s, 0.05H, 95% D), 3.93 (s, 2.85H, 5% D); ^{13}C NMR (126 MHz, $CDCl_3$) δ 166.0, 148.3, 133.0-132.4 (1C), 131.8-131.2 (1C), 130.0-129.4 (1C), 127.7-127.6 (1C), 124.0-123.5 (1C), 53.4.

General procedure C: ^{1}H NMR (500 MHz, $CDCl_3$) δ 7.92 (s, 0.17H, 83% D), 7.75 (s, 0.24H, 76% D), 7.68 (s, 0.12H, 88% D), 7.64 (s, 0.10H, 90% D), 3.93 (s, 2.94H, 2% D).

General procedure D: ^{1}H NMR (500 MHz, $CDCl_3$) δ 7.93-7.92 (m, 0.42H, 58% D), 7.76-7.74 (m, 0.45H, 55% D), 7.70-7.67 (m, 0.30H, 70% D), 7.66-7.62 (s, 0.27H, 73% D), 3.93 (s, 3.00H, 0% D).

Mass Data

	M+3	M+4	M+5
m/z	184	185	186
Abound	84963	258467	24636
Theoretical exact mass of start material: 181.0375			
Weighted average of deuterated product: 184.8361			
Average %D: 95%			

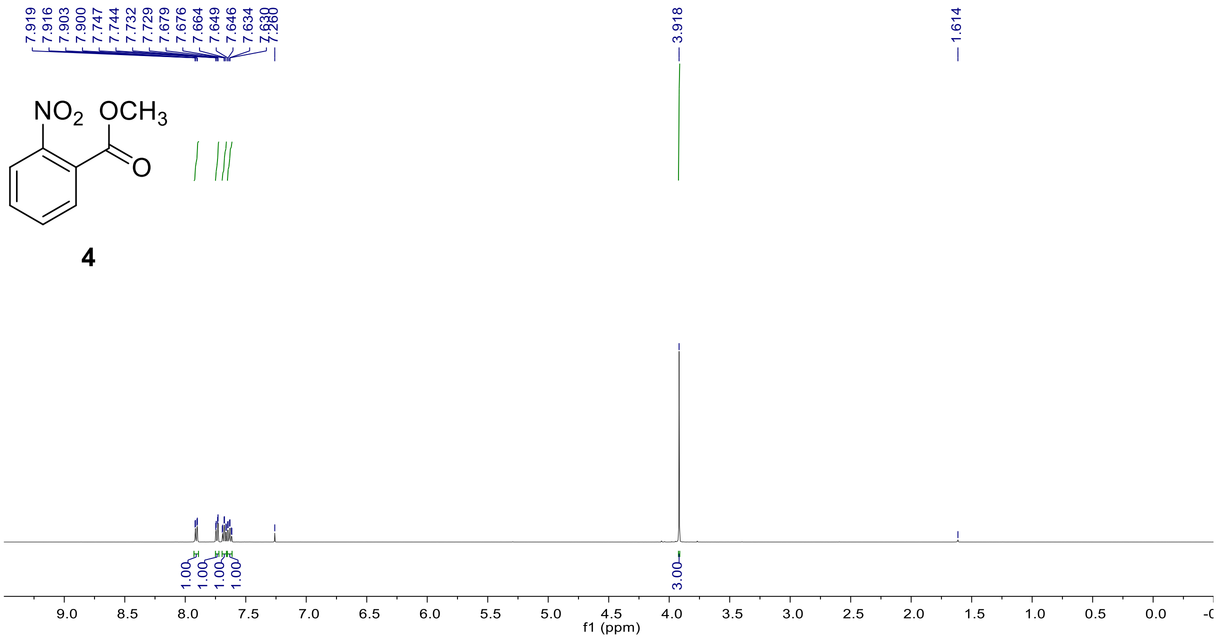

^{1}H NMR spectrum of compound **4** ($CDCl_3$, 500 MHz)

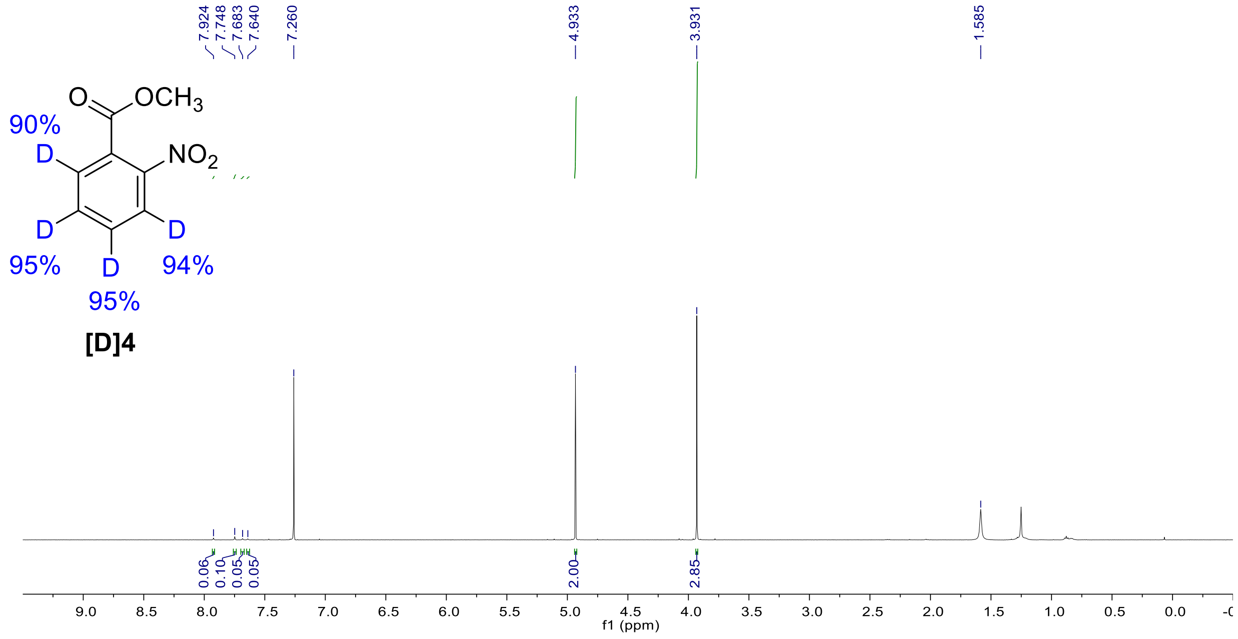

^{1}H NMR spectrum of compound **[D]4** (Procedure A, $CDCl_3$, 500 MHz)

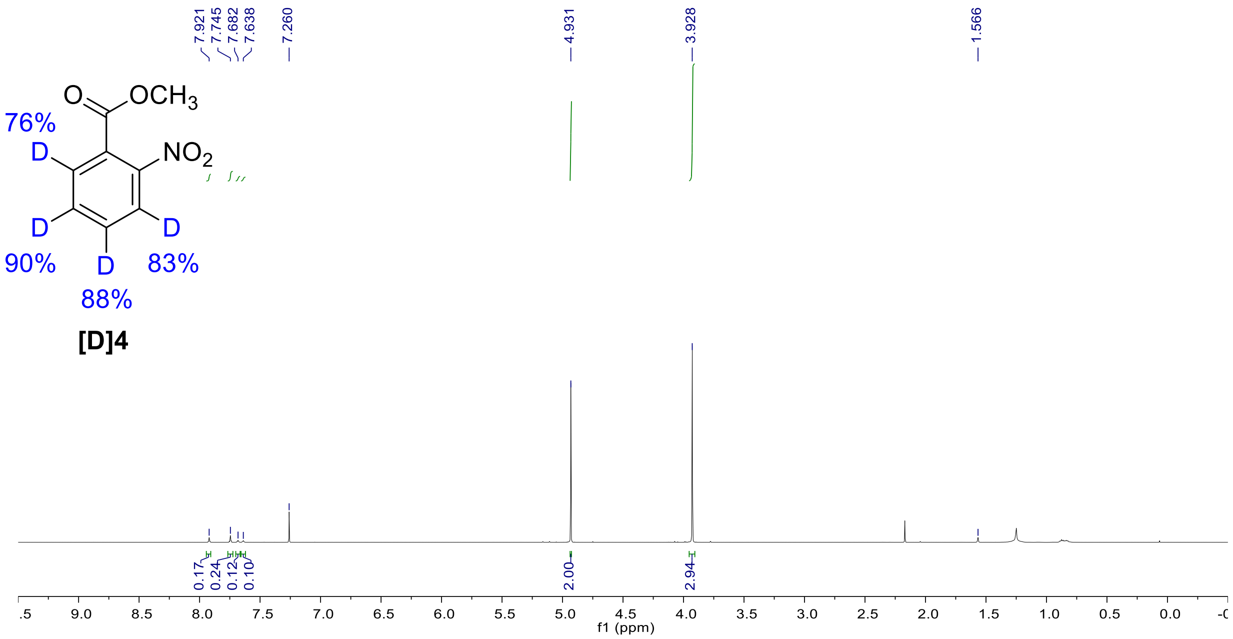

^{1}H NMR spectrum of compound **[D]4** (Procedure C, $CDCl_3$, 500 MHz)

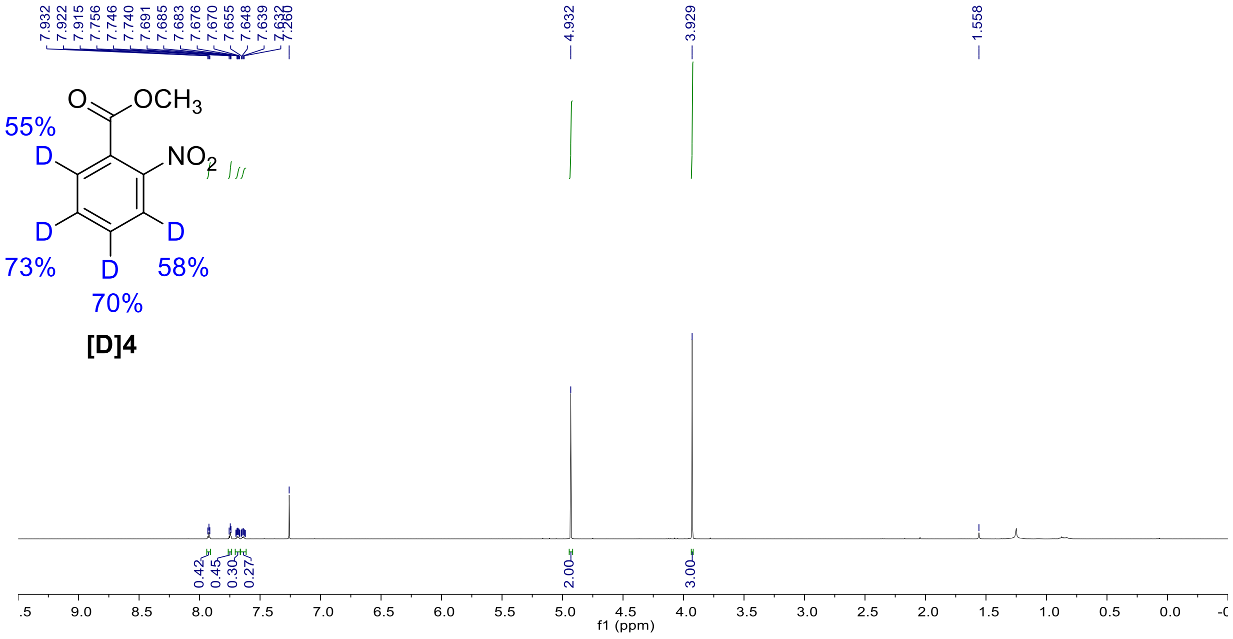

^{1}H NMR spectrum of compound **[D]4** (Procedure D, $CDCl_3$, 500 MHz)

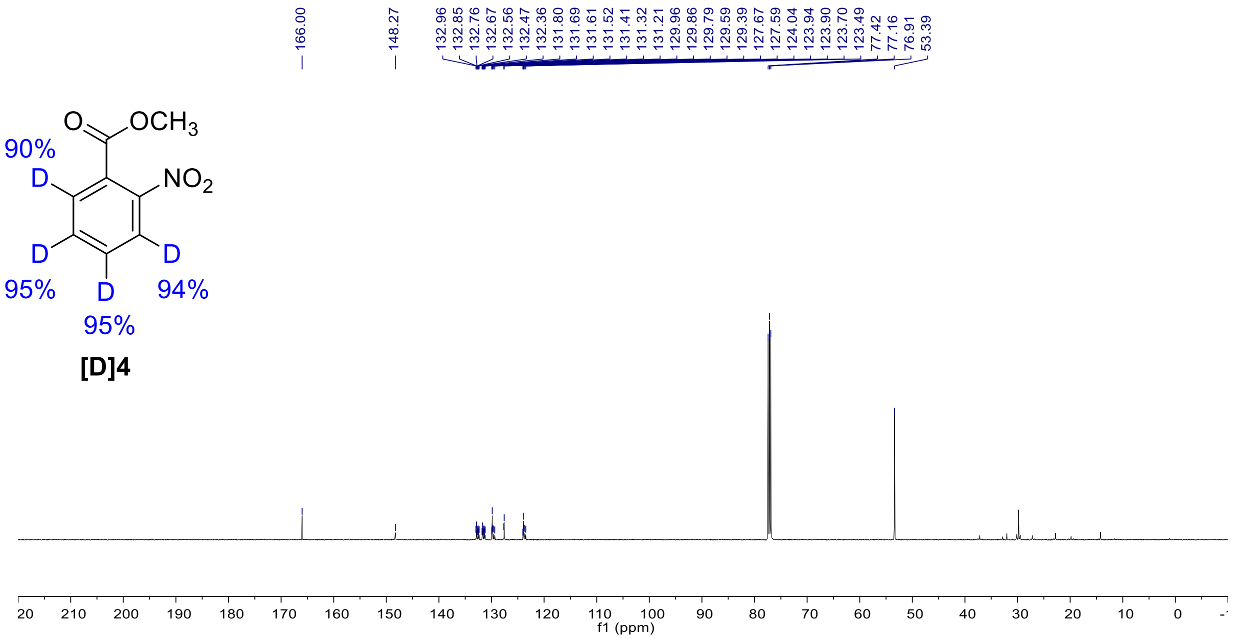

^{13}C NMR spectrum of compound **[D]4** (Procedure A, $CDCl_3$, 126 MHz)

2,3-dimethylbenzoic acid (5)

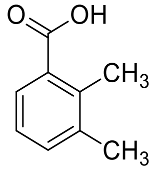

Following the general procedure A, the reaction was set up with 2,3-dimethylbenzoic acid (30.0 mg, 0.20 mmol). Purification by flash column chromatography (hexanes/EtOAc/HCO_2H = 6:1:0.07) provided product **[D]5** as a yellow solid (28 mg, 90% yield).

Deuterium Incorporation

General procedure A: [LCMS (ESI)] calcd for $C_9H_3D_6O_2$ $[M-H]^-$ 5.64 D/molecule, [^{1}H NMR] 5.64 D/molecule.

NMR Data of the Starting Material

^{1}H NMR (500 MHz, $CDCl_3$) δ 7.82 (d, *J* = 7.7 Hz, 1H), 7.35 (d, *J* = 7.4 Hz, 1H), 7.17 (t, *J* = 7.7 Hz, 1H), 2.55 (s, 3H), 2.35 (s, 3H).

NMR Data of the Product

General procedure A: ^{1}H NMR (500 MHz, $CDCl_3$) δ 7.83 (s, 0.05H, 95% D), 7.35 (s, 0.11H, 89% D), 7.17 (s, 0.05H, 95% D), 2.50 (s, 0.14H, 95% D), 2.35 (s, 3H); ^{13}C NMR (126 MHz, $CDCl_3$) δ 174.6, 139.0, 138.3, 134.3-133.7 (1C), 129.5, 128.9-128.4 (1C), 125.2-124.7 (1C), 20.82-20.76 (1C), 18.4-15.9 (1C).

Mass Data

LabelChecker Results

Formula: C9 H9 O2

Mass (monoisotopic): 149.06

Difference Value: 0.000084

Error Sum: 0.009

Error (%): 0.217

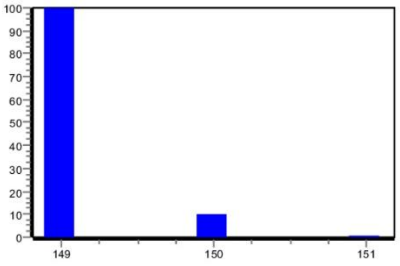

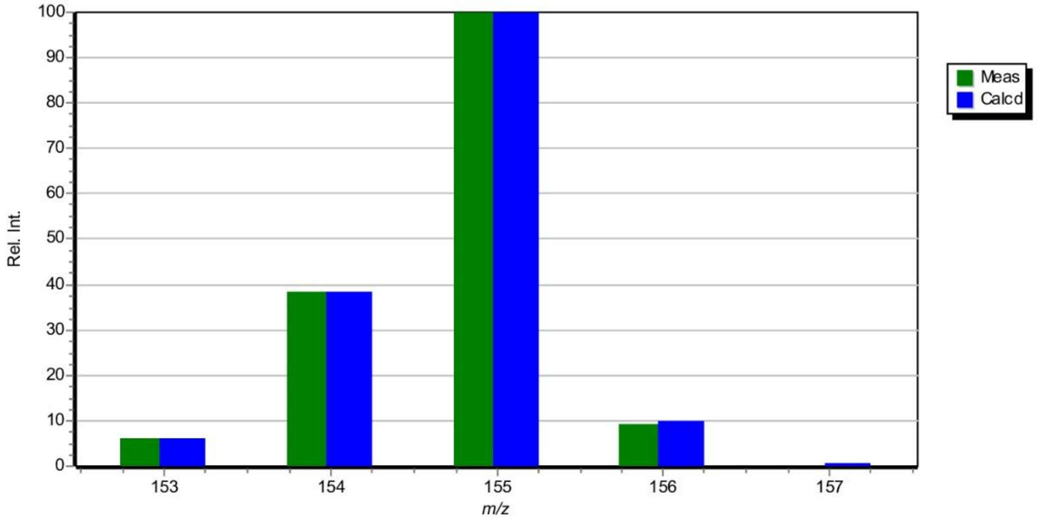

Deuterium: 0-fold (%): 0.00 0.00
Deuterium: 1-fold (%): 0.00 0.00
Deuterium: 2-fold (%): 0.00 0.00
Deuterium: 3-fold (%): 0.00 0.00
Deuterium: 4-fold (%): 6.39 4.38
Deuterium: 5-fold (%): 39.25 26.95
Deuterium: 6-fold (%): 100.00 68.66
Label Atom Sum: 5.64 (62.70%)

Isotope List used for fitting data:

m/z	intensity
153.08	1186441
154.09	7411855
155.10	19315192
156.10	1817962

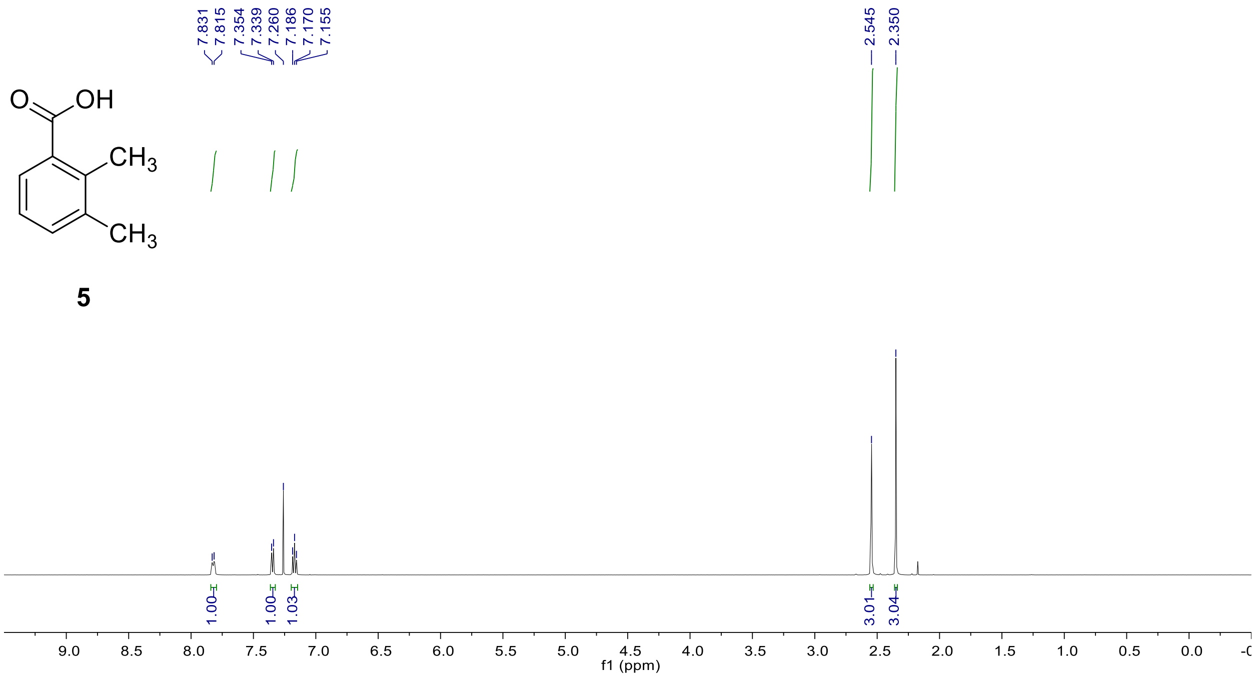

^{1}H NMR spectrum of compound **5** ($CDCl_3$, 500 MHz)

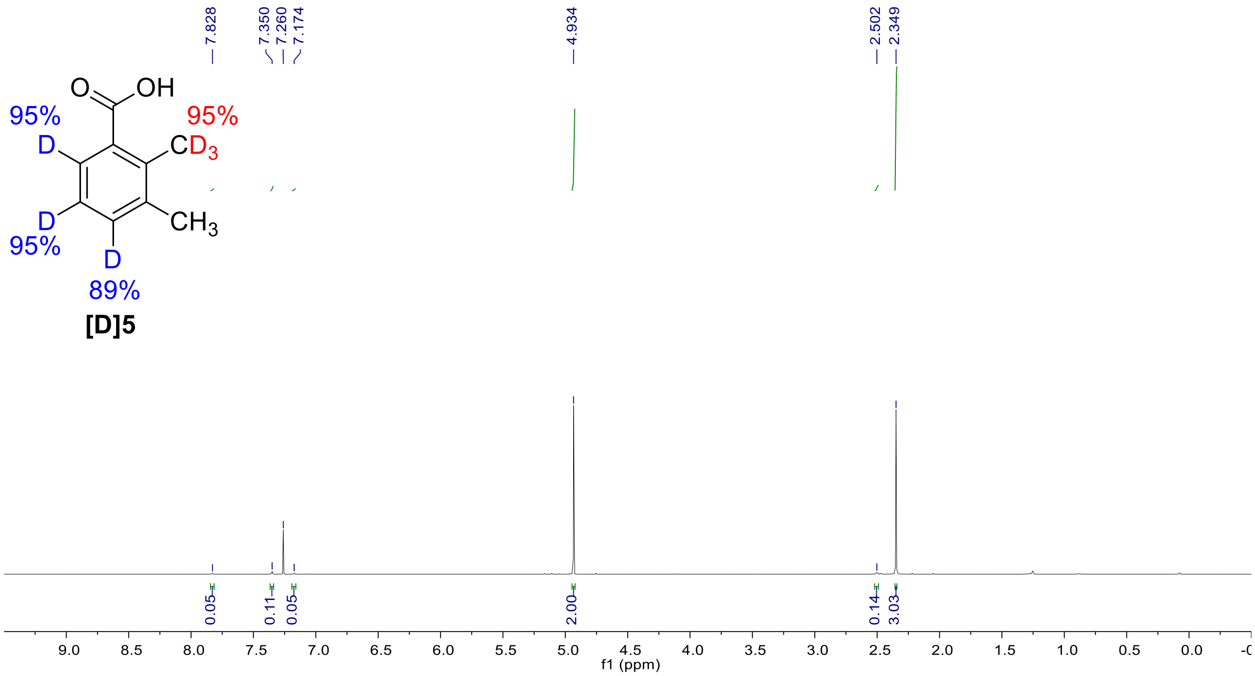

^{1}H NMR spectrum of compound **[D]5** (Procedure A, $CDCl_3$, 500 MHz)

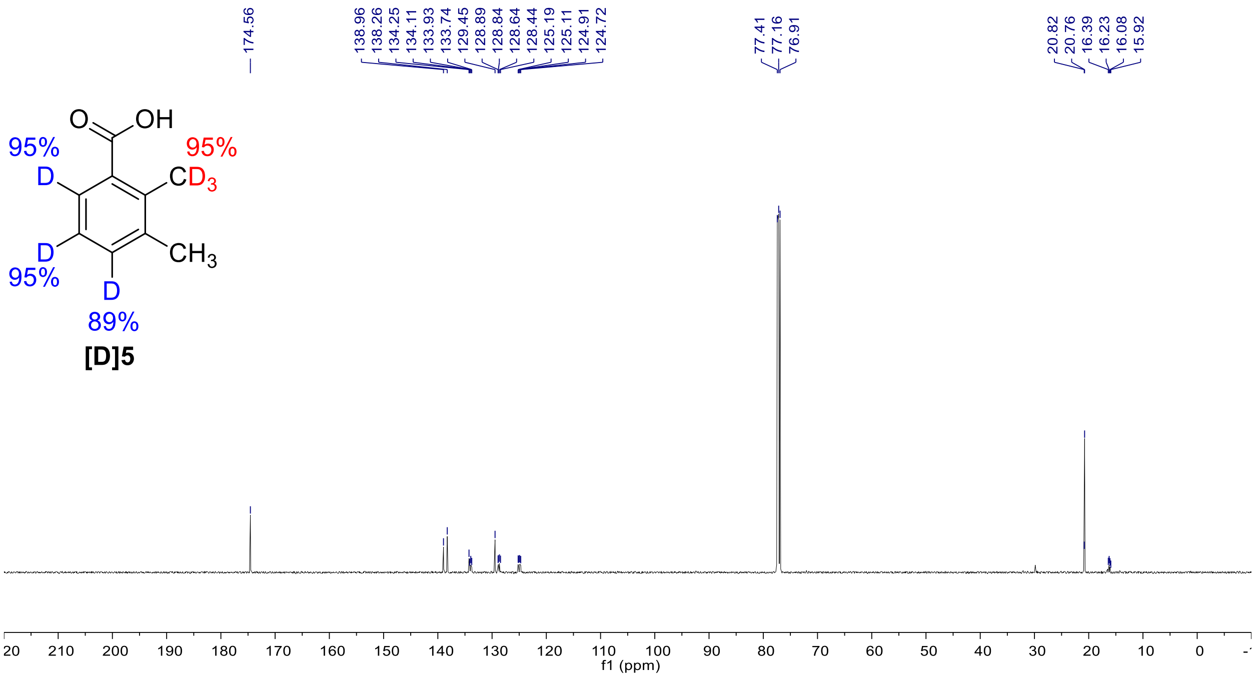

^{13}C NMR spectrum of compound **[D]5** (Procedure A, $CDCl_3$, 126 MHz)

2,6-dimethylbenzoic acid (6)

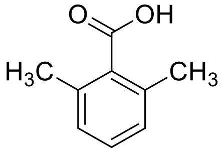

Following the general procedure A, the reaction was set up with 2,6-dimethylbenzoic acid (30.0 mg, 0.20 mmol). Purification by flash column chromatography (hexanes/EtOAc/HCO_2H = 10:1:0.1) provided product **[D]6** as a white solid (27 mg, 85% yield).

Deuterium Incorporation

General procedure A: [LCMS (ESI)] calcd for $C_9D_9O_2$ $[M-H]^-$ 8.40 D/molecule, [^{1}H NMR] 8.23 D/molecule.

NMR Data of the Starting Material

^{1}H NMR (500 MHz, $CDCl_3$) δ 7.23 (t, J = 7.6 Hz, 1H), 7.07 (d, J = 7.6 Hz, 2H), 2.45 (s, 6H).

NMR Data of the Product

General procedure A: ^{1}H NMR (500 MHz, $CDCl_3$) δ 7.23 (s, 0.07H, 93% D), 7.07 (s, 0.22H, 89% D), 2.46-2.37 (m, 0.47H, 92% D); ^{13}C NMR (126 MHz, $CDCl_3$) δ 176.2, 135.7 (2C), 132.5, 130.0-129.4 (1C), 128.1-127.4 (2C), 20.1-19.1 (2C).

Mass Data

LabelChecker Results

Formula: C9 H9 O2
Mass (monoisotopic): 149.06

Difference Value: 0.000070
Error Sum: 0.008
Error (%): 0.162

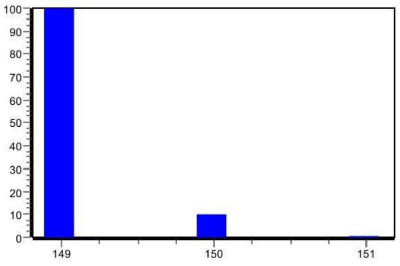

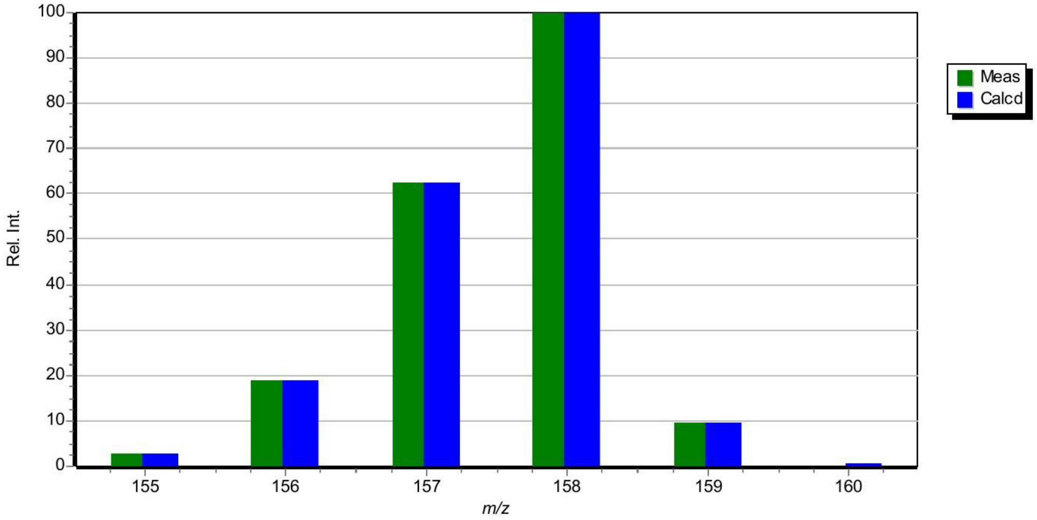

Deuterium: 0-fold (%): 0.00 0.00
Deuterium: 1-fold (%): 0.00 0.00
Deuterium: 2-fold (%): 0.00 0.00
Deuterium: 3-fold (%): 0.00 0.00
Deuterium: 4-fold (%): 0.00 0.00
Deuterium: 5-fold (%): 0.00 0.00
Deuterium: 6-fold (%): 2.99 1.60
Deuterium: 7-fold (%): 19.67 10.49
Deuterium: 8-fold (%): 64.82 34.58
Deuterium: 9-fold (%): 100.00 53.34
Label Atom Sum: 8.40 (93.30%)

Isotope List used for fitting data:

m/z intensity
155.10 15070257
156.10 100545912
157.11 336445888
158.12 536771776
159.12 51300448

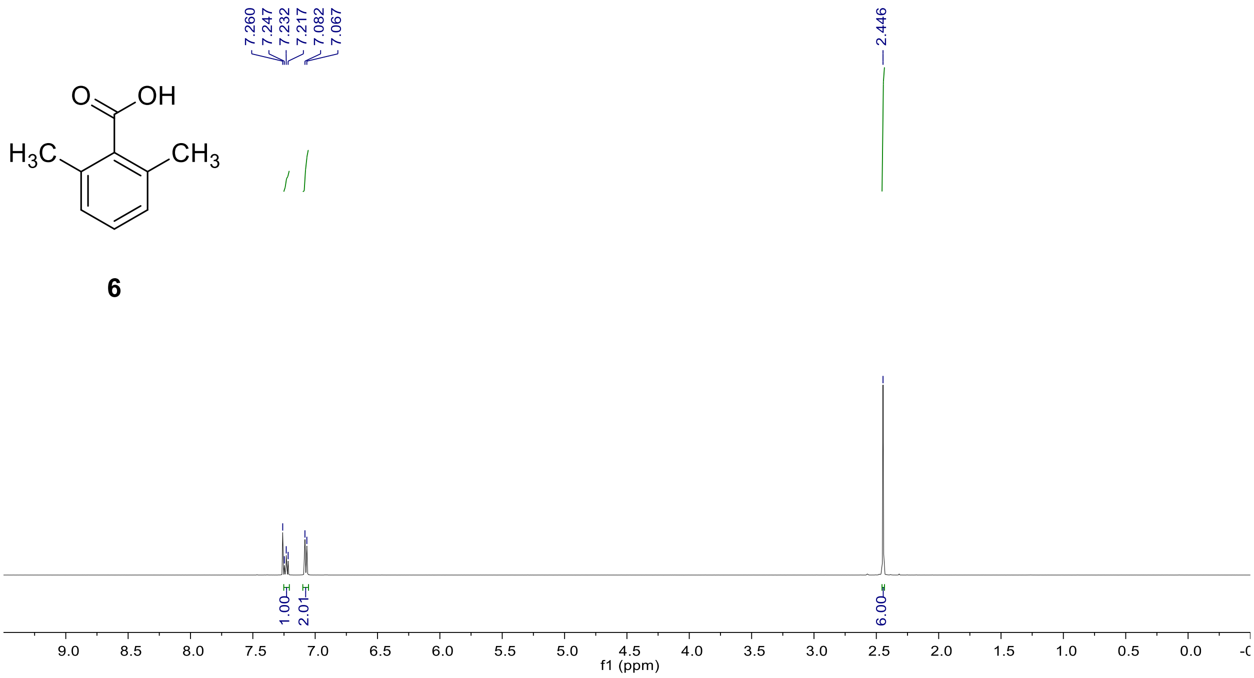

^{1}H NMR spectrum of compound **6** ($CDCl_3$, 500 MHz)

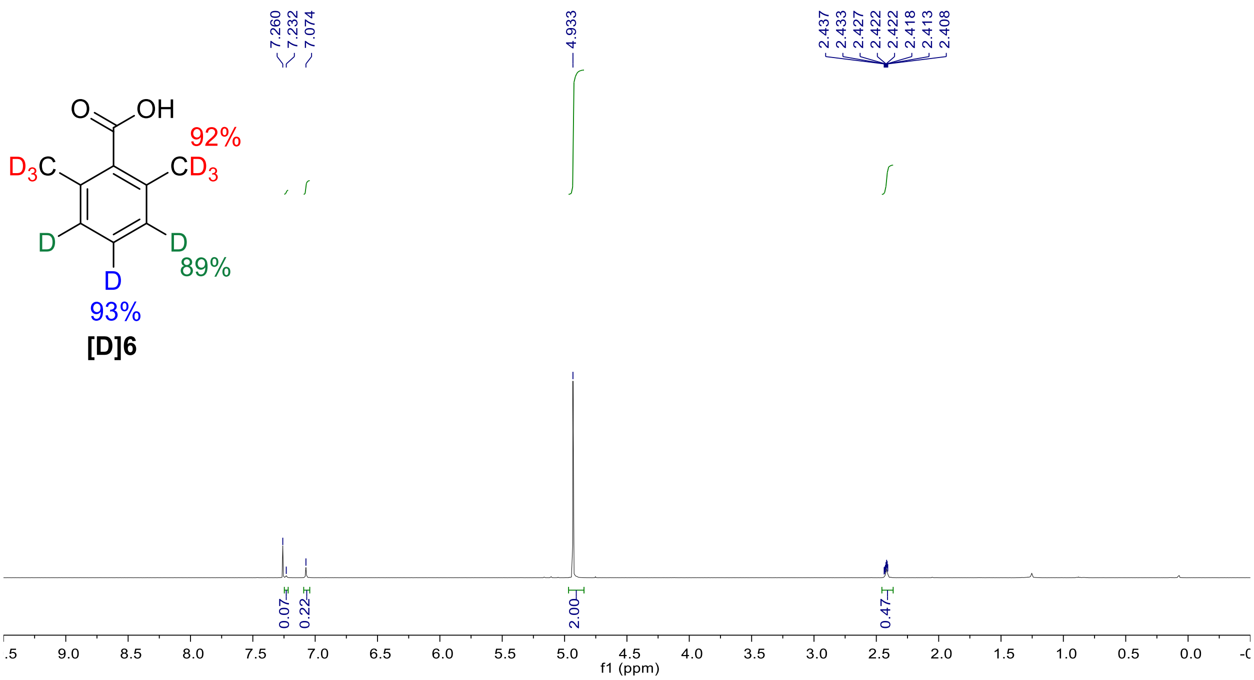

^{1}H NMR spectrum of compound **[D]6** (Procedure A, $CDCl_3$, 500 MHz)

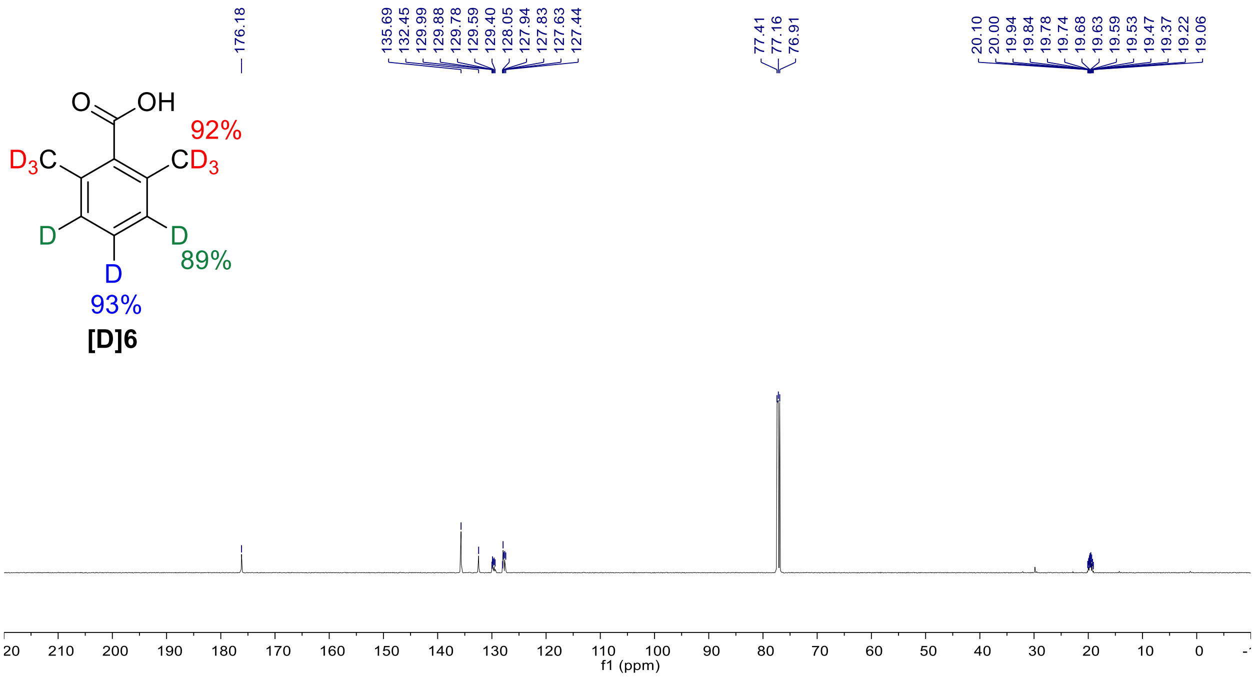

^{13}C NMR spectrum of compound **[D]6** (Procedure A, $CDCl_3$, 126 MHz)

2,4,6-trimethylbenzoic acid (7)

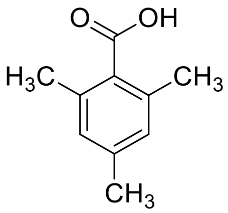

Following the general procedure A, the reaction was set up with 2,4,6-trimethylbenzoic acid (32.8 mg, 0.20 mmol). Purification by flash column chromatography (hexanes/EtOAc/HCO_2H = 10:1:0.1) provided product **[D]7** as a white solid (24 mg, 70% yield).

Deuterium Incorporation

General procedure A: [LCMS (ESI)] calcd for $C_{10}H_3D_8O_2$ $[M\text{-}H]^-$ 6.76 D/molecule, [^1H NMR] 6.66 D/molecule.

NMR Data of the Starting Material

^1H NMR (500 MHz, $CDCl_3$) δ 6.89 (s, 2H), 2.42 (s, 6H), 2.30 (s, 3H).

NMR Data of the Product

General procedure A: ^1H NMR (500 MHz, $CDCl_3$) δ 6.89 (s, 0.92H, 54% D), 2.42-2.37 (m, 0.40H, 93% D), 2.30 (s, 3H); ^{13}C NMR (126 MHz, $CDCl_3$) δ 173.7, 138.2, 135.4 (2C), 132.2-131.4 (1C), 128.1 (2C), 20.9, 20.8 (2C).

Mass Data

LabelChecker Results

Formula: C10 H11 O2
Mass (monoisotopic): 163.08

Difference Value: 0.000043
Error Sum: 0.007
Error (%): 0.083

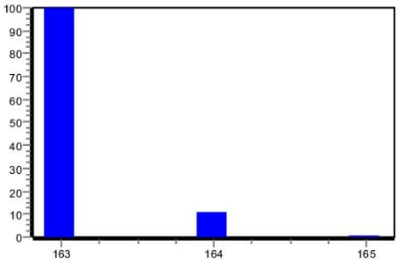

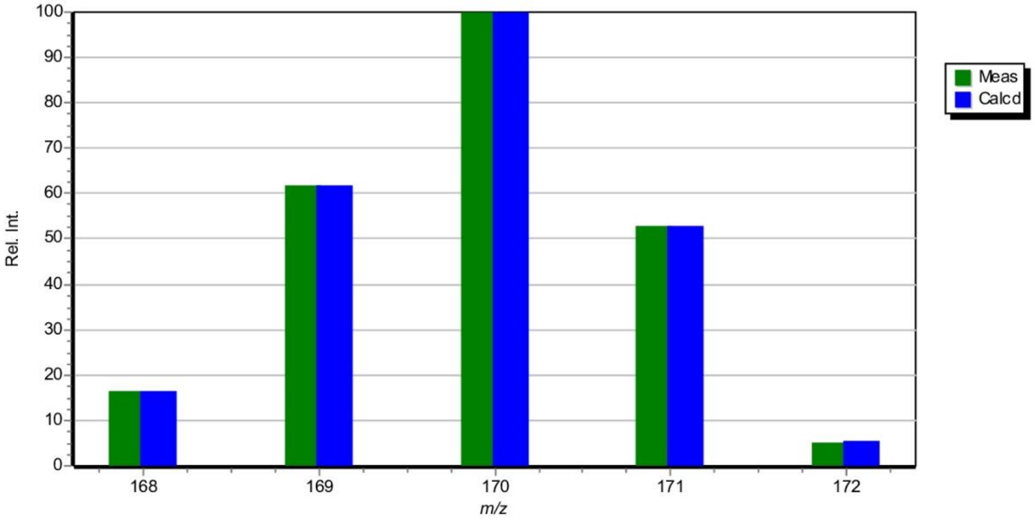

Deuterium: 0-fold (%): 0.00 0.00
Deuterium: 1-fold (%): 0.00 0.00
Deuterium: 2-fold (%): 0.02 0.01
Deuterium: 3-fold (%): 0.00 0.00
Deuterium: 4-fold (%): 0.00 0.00
Deuterium: 5-fold (%): 17.57 7.74
Deuterium: 6-fold (%): 64.43 28.36
Deuterium: 7-fold (%): 100.00 44.03
Deuterium: 8-fold (%): 45.11 19.86
Label Atom Sum: 6.76 (61.45%)

Isotope List used for fitting data:

m/z	intensity
168.11	1412835
169.11	5335630
170.12	8623080
171.12	4563199
172.13	435667

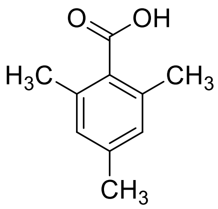

7

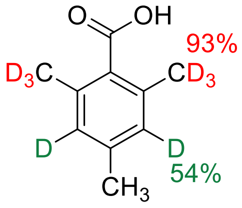

[D]7

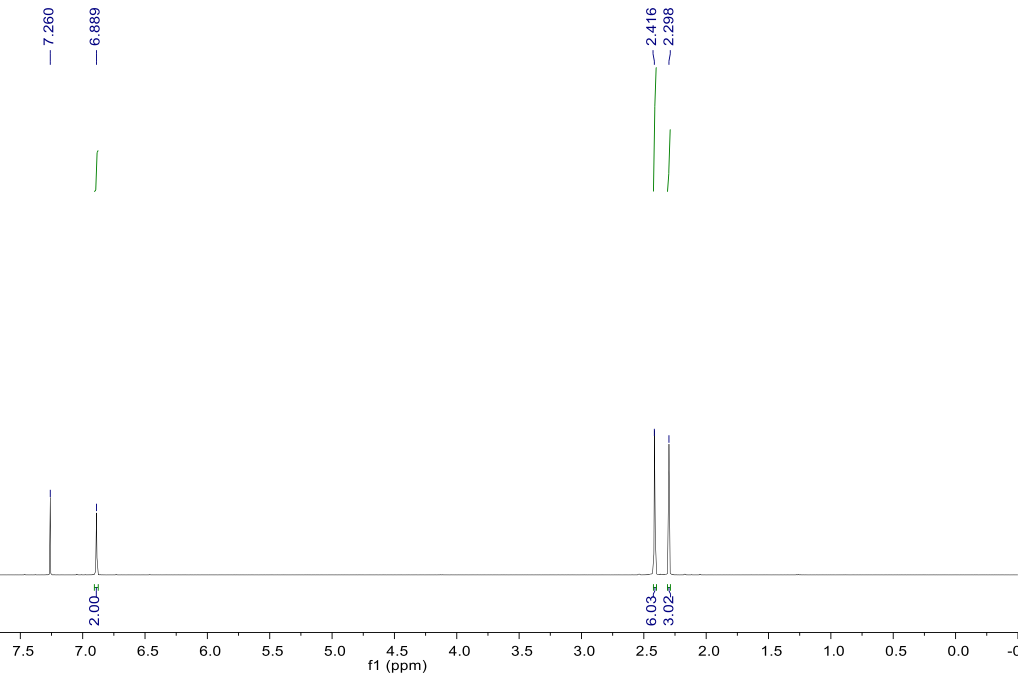

^{1}H NMR spectrum of compound **7** ($CDCl_3$, 500 MHz)

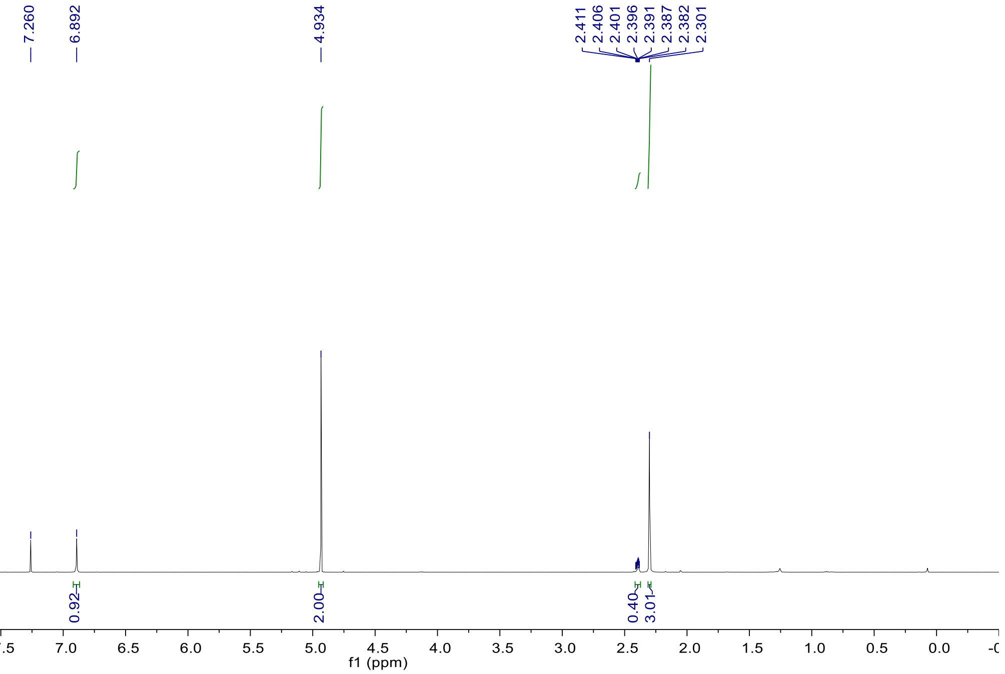

^{1}H NMR spectrum of compound **[D]7** (Procedure A, $CDCl_3$, 500 MHz)

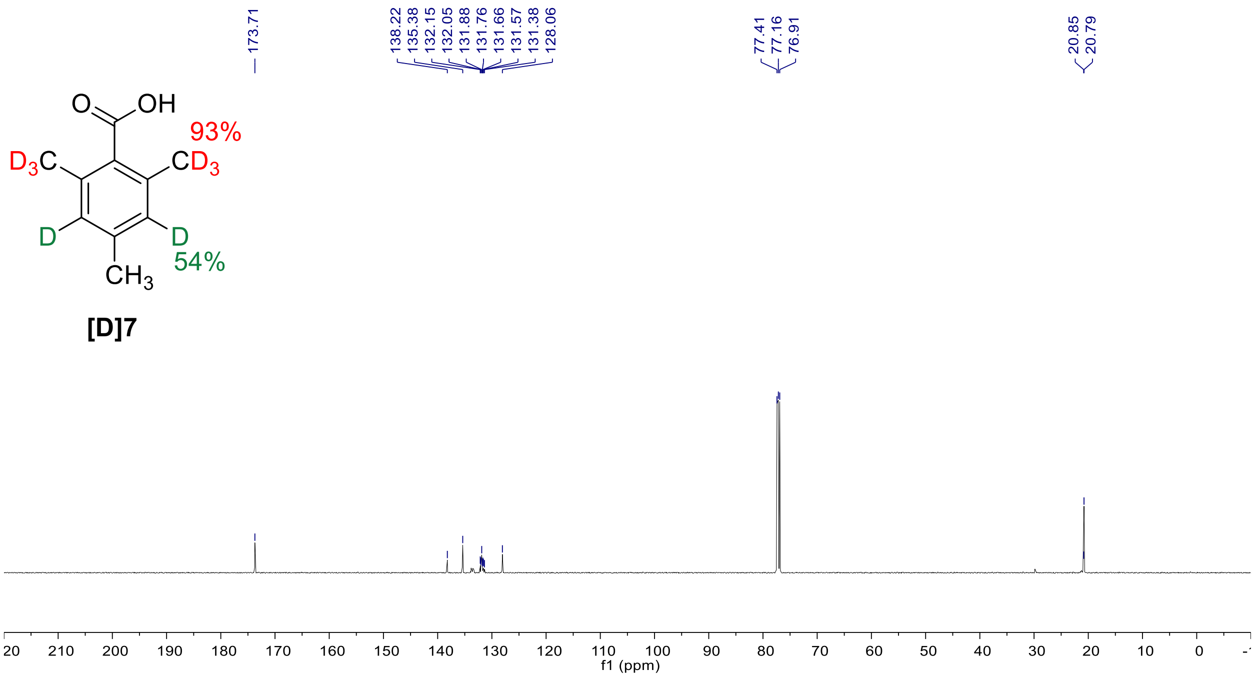

^{13}C NMR spectrum of compound **[D]7** (Procedure A, $CDCl_3$, 126 MHz)

2-methylbenzoic acid (8)

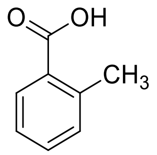

Following the general procedure A, the reaction was set up with 2-methylbenzoic acid (27.2 mg, 0.20 mmol). Purification by flash column chromatography (hexanes/EtOAc/HCO_2H = 5:1:0.05) provided product **[D]8** as a white solid (24 mg, 84% yield).

Deuterium Incorporation

General procedure A: [LCMS (ESI)] calcd for $C_8D_7O_2$ $[M-H]^-$ 6.48 D/molecule, [^{1}H NMR] 6.48 D/molecule.

NMR Data of the Starting Material

^{1}H NMR (500 MHz, $CDCl_3$) δ 8.07 (d, J = 8.4 Hz, 1H), 7.46 (td, J = 7.5, 1.5 Hz, 1H), 7.31-7.27 (m, 2H), 2.67 (s, 3H).

NMR Data of the Product

General procedure A: ^{1}H NMR (500 MHz, $CDCl_3$) δ 8.08 (s, 0.08H, 92% D), 7.46 (s, 0.06H, 94% D), 7.31-7.27 (m, 0.21H, 90% D), 2.67-2.60 (m, 0.19H, 94% D); ^{13}C NMR (126 MHz, $CDCl_3$) δ 173.8, 141.4, 132.9-132.4 (1C), 132.0-131.2 (2C), 128.4, 125.8-125.3 (1C), 21.9-21.2 (1C).

Mass Data

LabelChecker Results

Formula: C8 H7 O2
Mass (monoisotopic): 135.04

Difference Value: 0.000058
Error Sum: 0.008
Error (%): 0.070

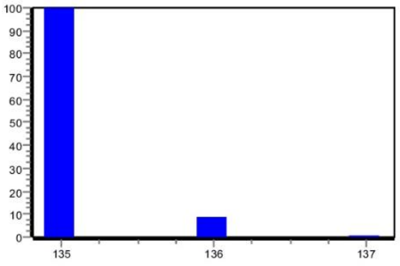

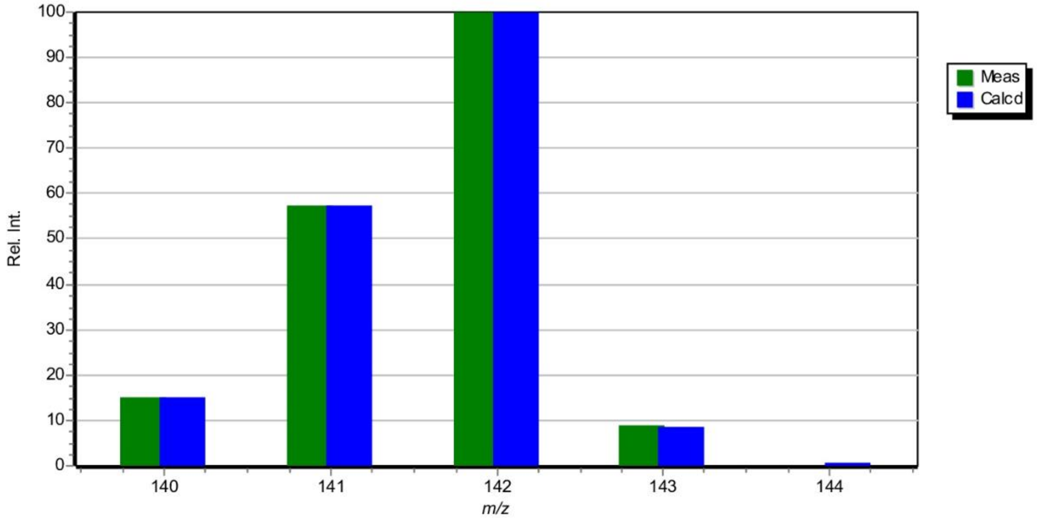

Deuterium: 0-fold (%): 0.00 0.00
Deuterium: 1-fold (%): 0.00 0.00
Deuterium: 2-fold (%): 0.00 0.00
Deuterium: 3-fold (%): 0.00 0.00
Deuterium: 4-fold (%): 0.00 0.00
Deuterium: 5-fold (%): 15.79 9.03
Deuterium: 6-fold (%): 59.16 33.81
Deuterium: 7-fold (%): 100.00 57.16
Label Atom Sum: 6.48 (92.59%)

Isotope List used for fitting data:

m/z	intensity
140.08	3497143
141.08	13405351
142.09	23317630
143.09	2078753

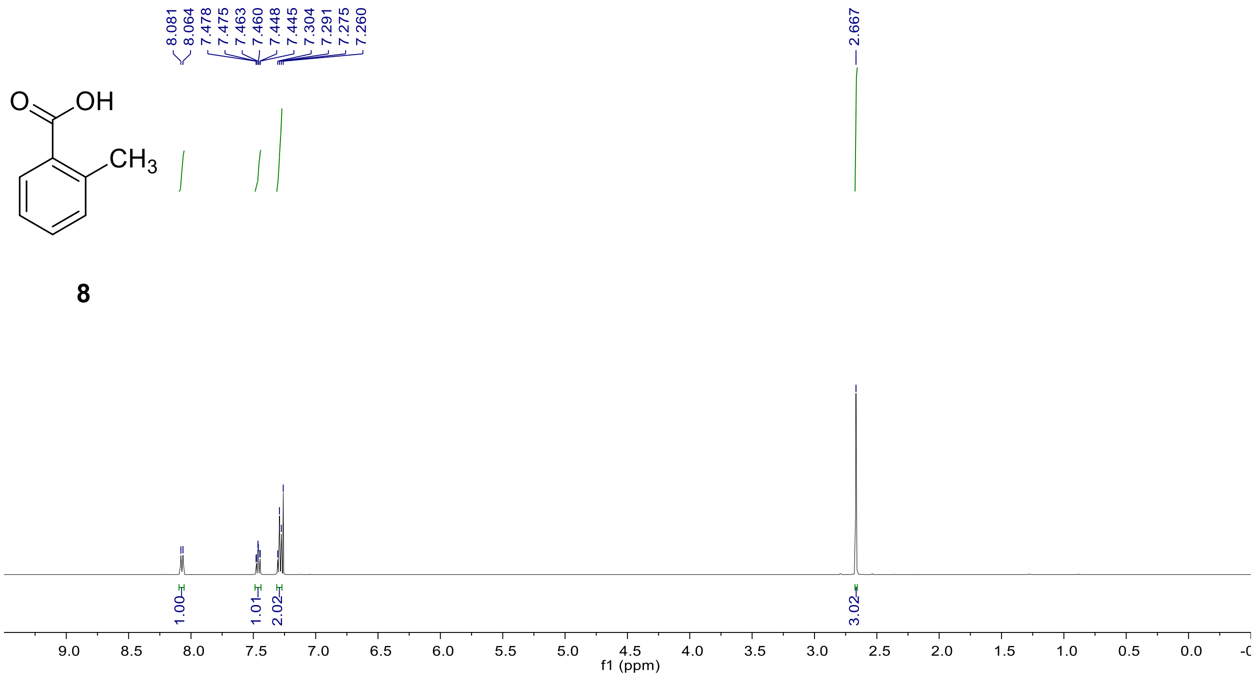

^1H NMR spectrum of compound **8** ($CDCl_3$, 500 MHz)

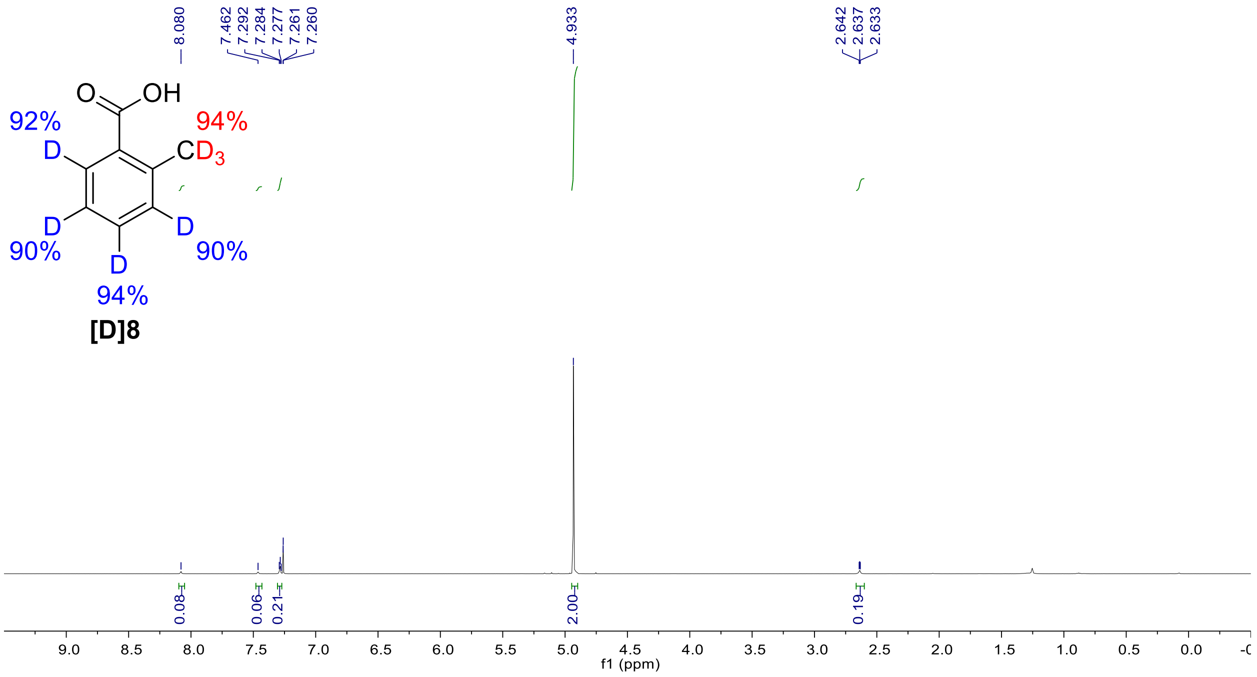

^1H NMR spectrum of compound **[D]8** (Procedure A, $CDCl_3$, 500 MHz)

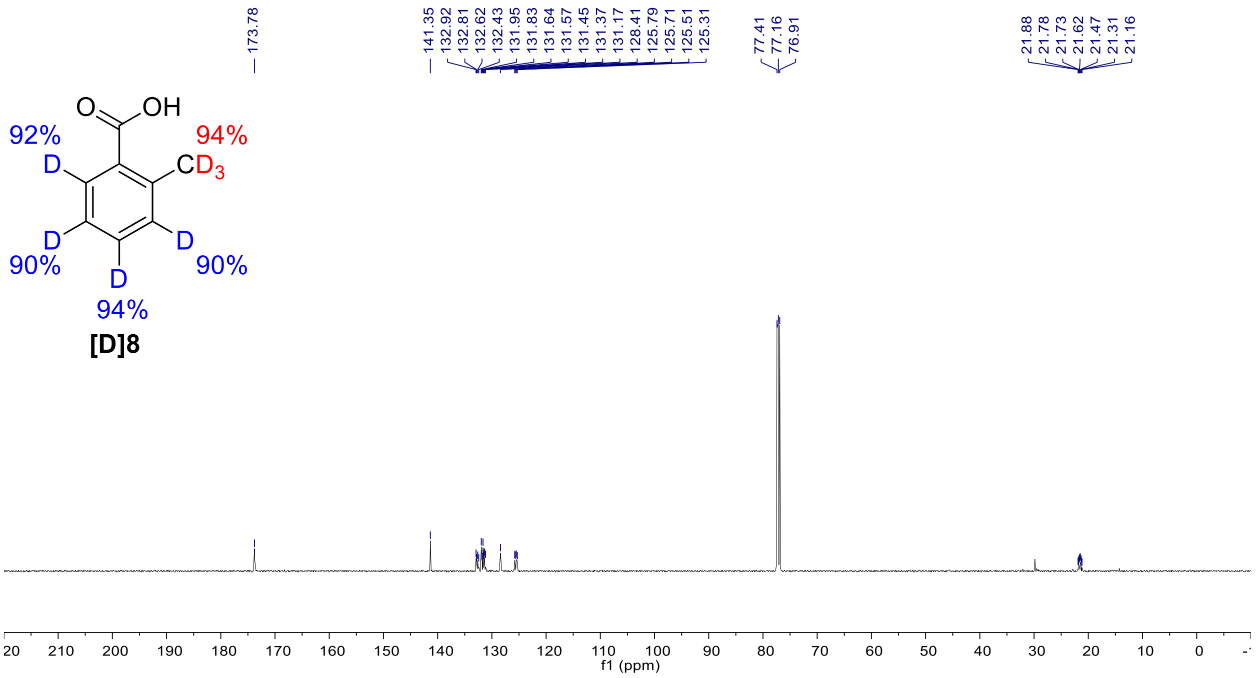

^{13}C NMR spectrum of compound **[D]8** (Procedure A, $CDCl_3$, 126 MHz)

5-fluoro-2-methylbenzoic acid (9)

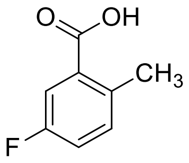

Following the general procedure A, the reaction was set up with 5-fluoro-2-methylbenzoic acid (30.8 mg, 0.20 mmol). Purification by flash column chromatography (hexanes/EtOAc = 15:1) provided product **[D]9** as a white solid (25 mg, 78% yield).

Deuterium Incorporation

General procedure A: [LCMS (ESI)] calcd for $C_8D_6O_2F$ $[M-H]^-$ 5.68 D/molecule, [^{1}H NMR] 5.72 D/molecule.

NMR Data of the Starting Material

^{1}H NMR (500 MHz, CD_2Cl_2) δ 7.74 (dd, J = 9.5, 2.9 Hz, 1H), 7.30-7.26 (m, 1H), 7.20 (td, J = 8.3, 2.9 Hz, 1H), 2.60 (s, 3H).

NMR Data of the Product

General procedure A: ^{1}H NMR (500 MHz, CD_2Cl_2) δ 7.75 (d, J = 9.6 Hz, 0.05H, 95% D), 7.28 (d, J = 5.5 Hz, 0.07H, 93% D), 7.20 (d, J = 8.1 Hz, 0.04H, 96% D), 2.57 (s, 0.13H, 96% D); ^{13}C NMR (126 MHz, CD_2Cl_2) δ 172.2, 161.6-159.6 (1C), 137.1, 133.5-132.9 (1C), 129.6, 120.0-119.4 (1C), 118.0-117.4 (1C), 20.8-19.8 (1C).

Mass data

LabelChecker Results

Formula: C8 H6 O2 F
Mass (monoisotopic): 153.04

Difference Value: 0.000061
Error Sum: 0.008
Error (%): 0.076

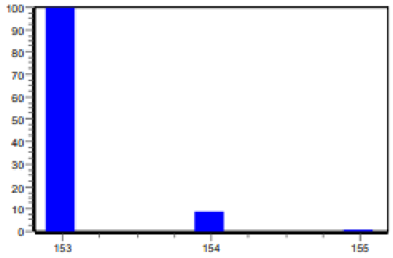

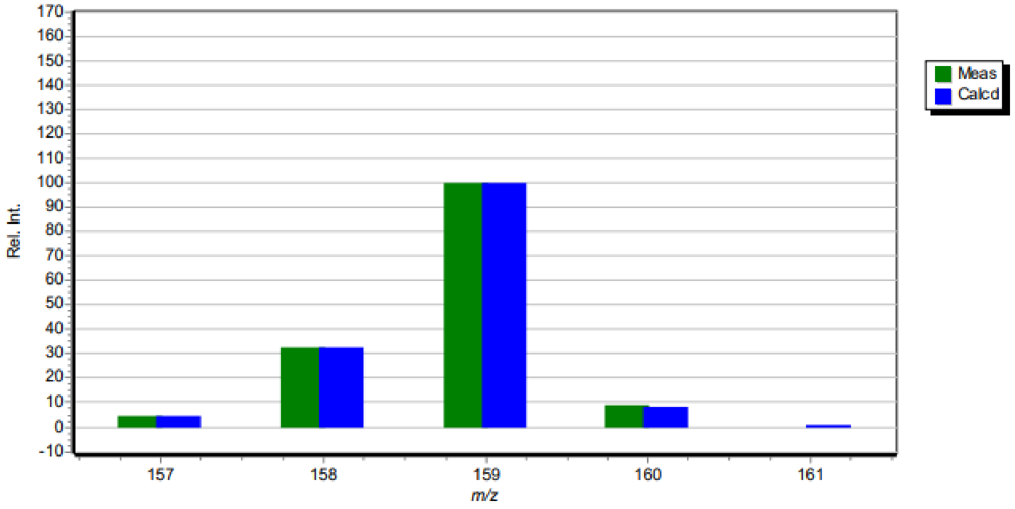

Deuterium: 0-fold (%): 0.00 0.00
Deuterium: 1-fold (%): 0.00 0.00
Deuterium: 2-fold (%): 0.04 0.03
Deuterium: 3-fold (%): 0.47 0.34
Deuterium: 4-fold (%): 4.51 3.25
Deuterium: 5-fold (%): 33.57 24.22
Deuterium: 6-fold (%): 100.00 72.16
Label Atom Sum: 5.68 (94.69%)

Isotope List used for fitting data:

m/z	intensity
155.05	1667
156.05	20345
157.06	196939
158.07	1470671
159.07	4458957
160.08	399994

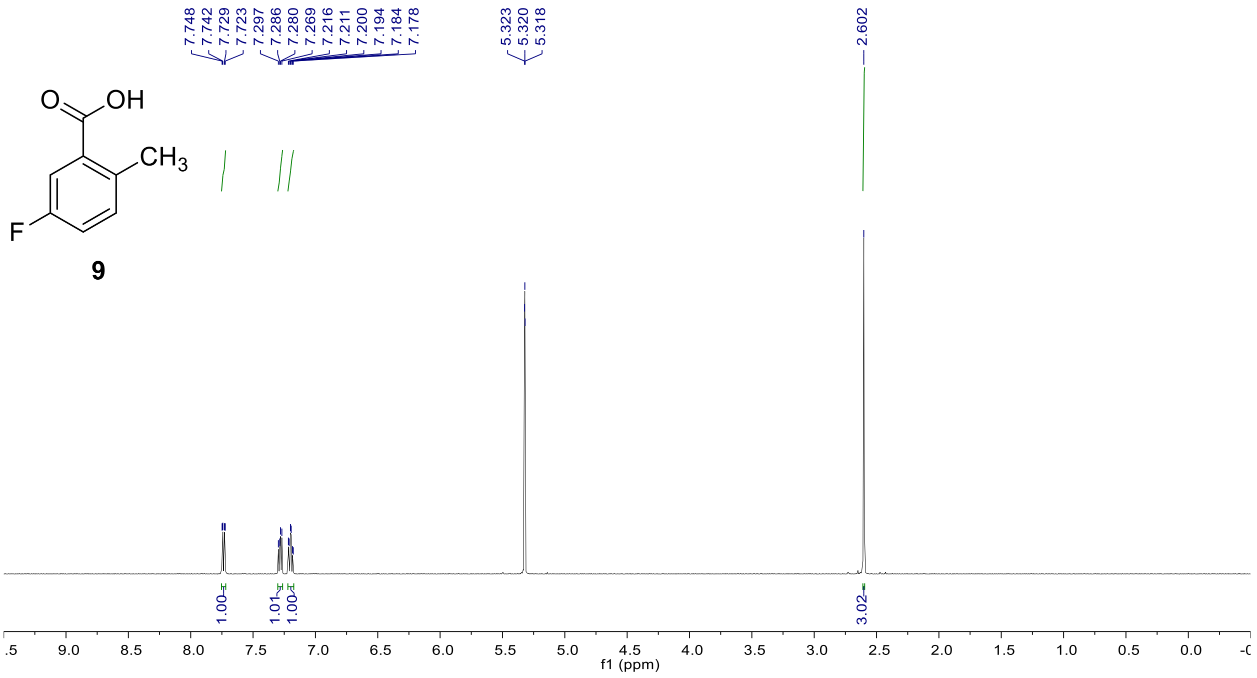

^{1}H NMR spectrum of compound **9** (CD_2Cl_2, 500 MHz)

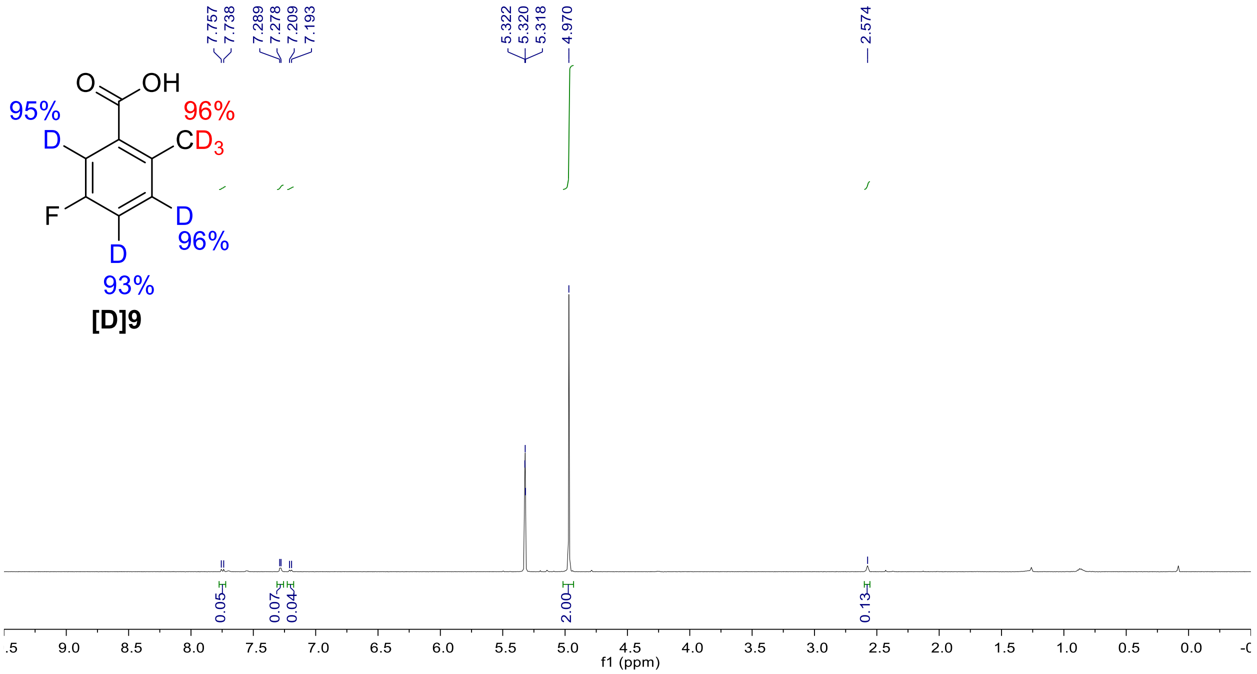

^{1}H NMR spectrum of compound **[D]9** (Procedure A, CD_2Cl_2, 500 MHz)

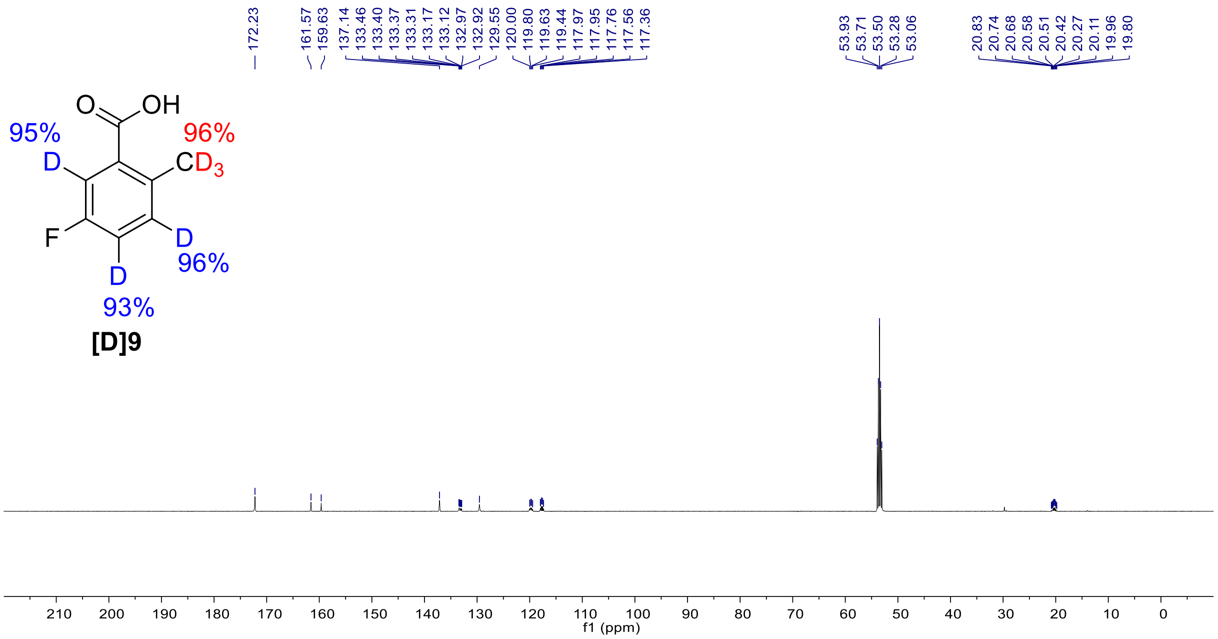

^{13}C NMR spectrum of compound **[D]9** (Procedure A, CD_2Cl_2, 126 MHz)

5-chloro-2-methylbenzoic acid (10)

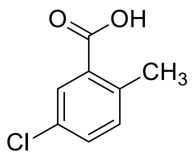

Following the general procedure A, the reaction was set up with 5-chloro-2-methylbenzoic acid (34.1 mg, 0.20 mmol). Purification by flash column chromatography (hexanes/EtOAc/AcOH= 20:1:0.2) provided product **[D]10** as a white solid (35 mg, 99% yield).

Deuterium Incorporation

General procedure A: [LCMS (ESI)] calcd for $C_8D_6ClO_2$ $[M-H]^-$ 5.62 D/molecule, [^{1}H NMR] 5.93 D/molecule.

NMR Data of the Starting Material

^{1}H NMR (500 MHz, $CDCl_3$) δ 8.05 (d, *J* = 2.4 Hz, 1H), 7.43 (dd, *J* = 8.2, 2.4 Hz, 1H), 7.23 (d, *J* = 8.2 Hz, 1H), 2.62 (s, 3H).

NMR Data of the Product

General procedure A: ^{1}H NMR (500 MHz, $CDCl_3$) δ 8.04 (d, *J* = 1.6 Hz, 0.02H, 98% D), 7.42 (s, 0.01H, 99% D), 7.22 (s, 0.01 H, 99% D), 2.59 (s, 0.03H, 99% D); ^{13}C NMR (126 MHz, $CDCl_3$) δ 172.0, 139.8, 133.2-132.5 (2C), 131.6-131.1 (2C), 129.8, 19.9.

Mass data

LabelChecker Results

Formula:	C8 H6 O2 Cl
Mass (monoisotopic):	169.01
Difference Value:	0.109808
Error Sum:	0.331
Error (%):	0.000

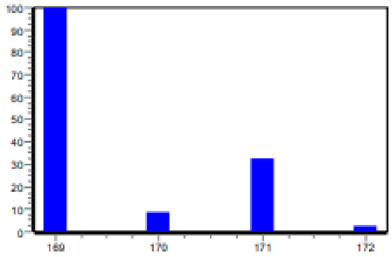

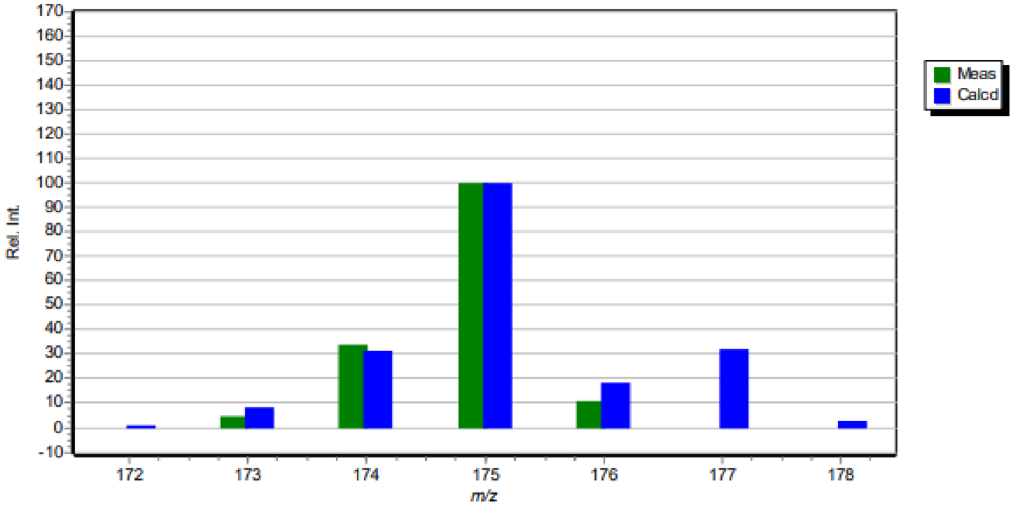

No Convergence! - Max Iterations (3000) reached!
Deuterium: 0-fold (%): 0.00 0.00
Deuterium: 1-fold (%): 0.00 0.00
Deuterium: 2-fold (%): 0.00 0.00
Deuterium: 3-fold (%): 1.21 0.85
Deuterium: 4-fold (%): 8.90 6.26
Deuterium: 5-fold (%): 32.21 22.63
Deuterium: 6-fold (%): 100.00 70.27
Label Atom Sum: 5.62 (93.72%)

Isotope List used for fitting data:

m/z	intensity
172.02	159795
173.03	2128777
174.04	14637720
175.04	43087964
176.03	4693202

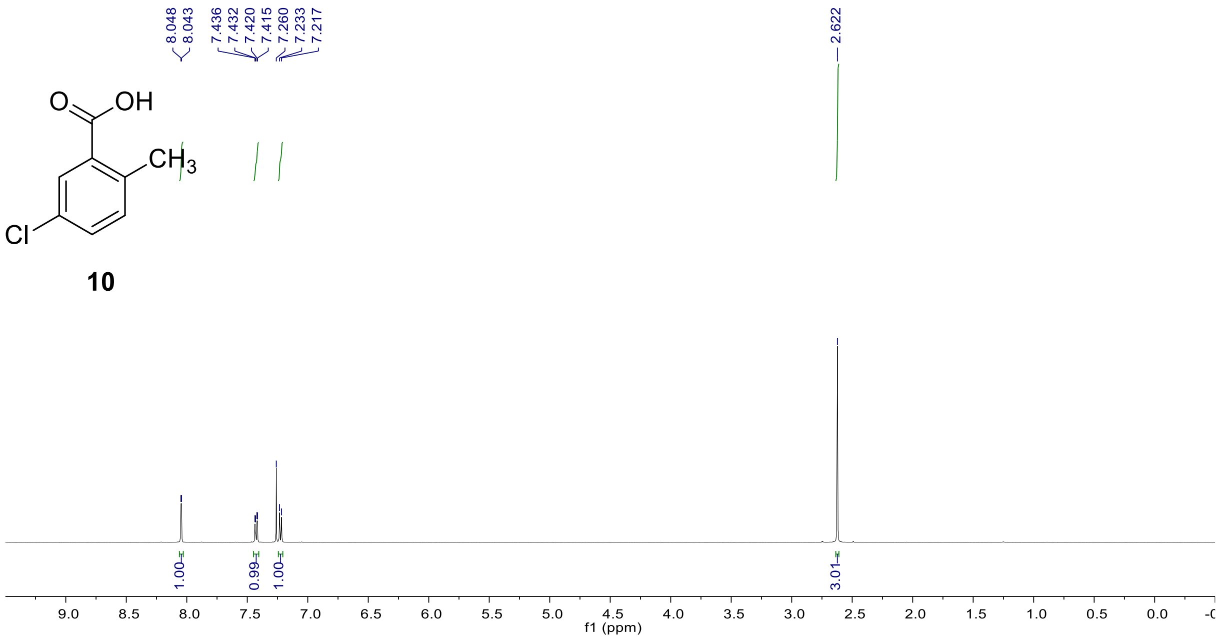

^{1}H NMR spectrum of compound **10** ($CDCl_3$, 500 MHz)

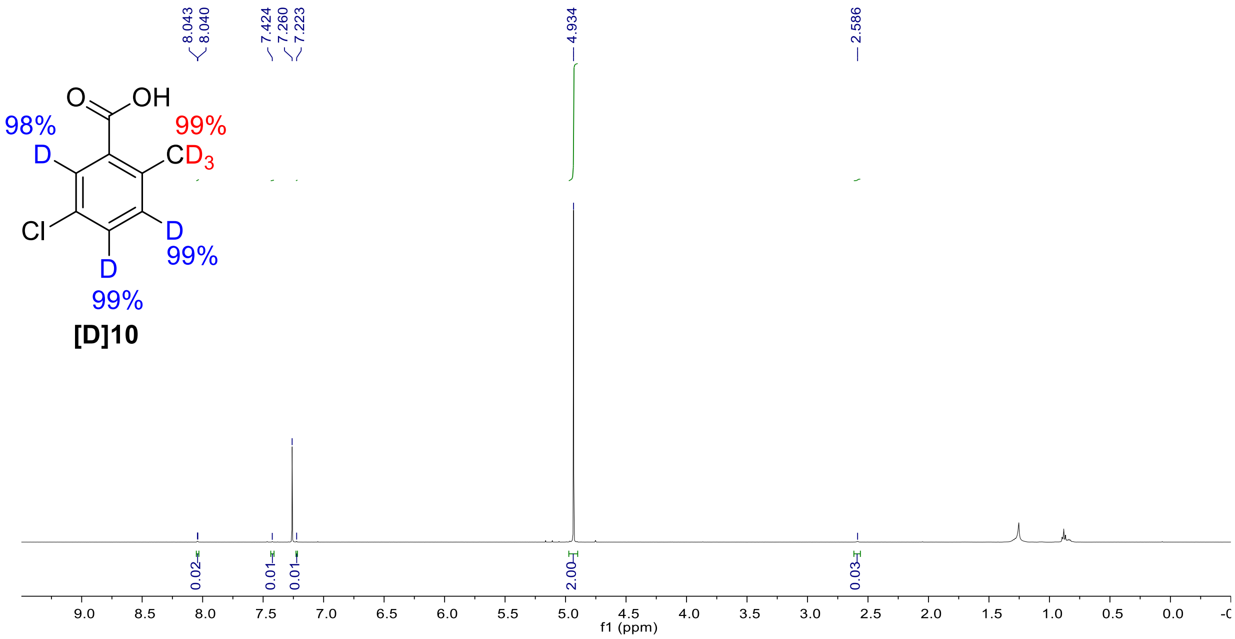

^{1}H NMR spectrum of compound **[D]10** (Procedure A, $CDCl_3$, 500 MHz)

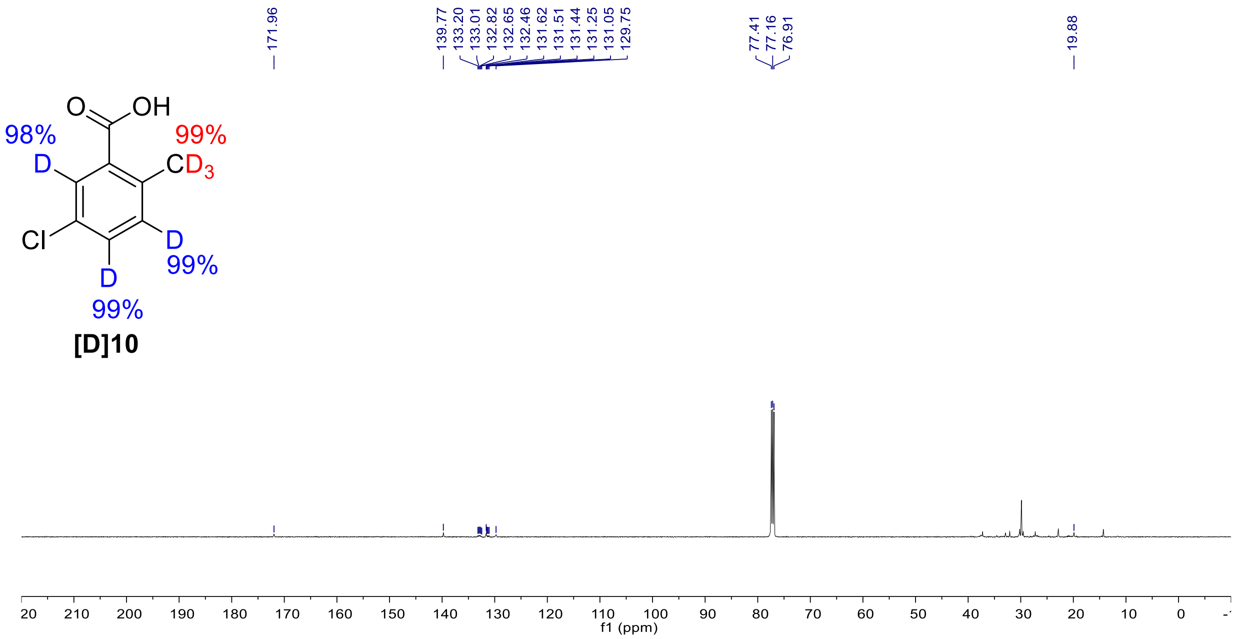

^{13}C NMR spectrum of compound **[D]10** (Procedure A, $CDCl_3$, 126 MHz)

2-chloro-6-methylbenzoic acid (11)

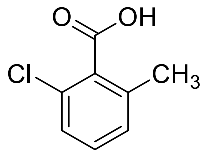

Following the general procedure A, the reaction was set up with 2-chloro-6-methylbenzoic acid (34.1 mg, 0.20 mmol). Purification by flash column chromatography (hexanes/EtOAc/AcOH = 20:1:0.2) provided product **[D]11** as a white solid (28 mg, 79% yield).

Deuterium Incorporation

General procedure A: [LCMS (ESI)] calcd for $C_8D_6O_2Cl$ $[M-H]^-$ 5.48 D/molecule, [^{1}H NMR] 5.93 D/molecule.

NMR Data of the Starting Material

^{1}H NMR (500 MHz, CD_2Cl_2) δ 7.31-7.27 (m, 2H), 7.21-7.17 (m, 1H), 2.41 (s, 3H).

NMR Data of the Product

General procedure A: ^{1}H NMR (500 MHz, CD_2Cl_2) δ 7.28 (d, *J* = 5.4 Hz, 0.01H, 100% D) 7.18 (s, 0.01H, 99% D), 2.37 (s, 0.06H, 98% D); ^{13}C NMR (126 MHz, CD_2Cl_2) δ 171.5, 137.0, 133.0, 131.0, 130.2, 128.8-128.0 (1C), 126.9-126.3 (1C), 19.5.

Mass Data

LabelChecker Results

Formula: C8 H6 O2 Cl
Mass (monoisotopic): 169.01

Difference Value: 0.006470
Error Sum: 0.080
Error (%): 0.219

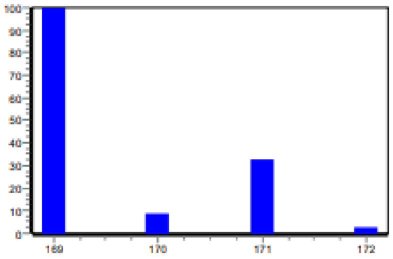

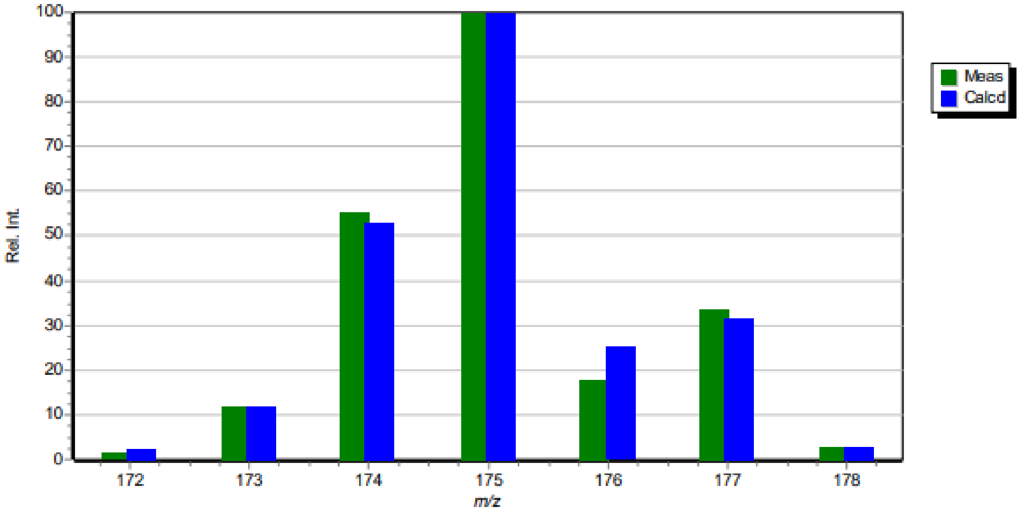

No Convergence! - Max Cycles (20) reached!
Deuterium: 0-fold (%): 0.00 0.00
Deuterium: 1-fold (%): 0.00 0.00
Deuterium: 2-fold (%): 0.01 0.01
Deuterium: 3-fold (%): 2.44 1.43
Deuterium: 4-fold (%): 12.74 7.45
Deuterium: 5-fold (%): 55.84 32.65
Deuterium: 6-fold (%): 100.00 58.47
Label Atom Sum: 5.48 (91.36%)

Isotope List used for fitting data:

m/z	intensity
172.02	3134200
173.03	25111850
174.04	116709160
175.04	211181680
176.03	37503204
177.04	71138928
178.04	5857466

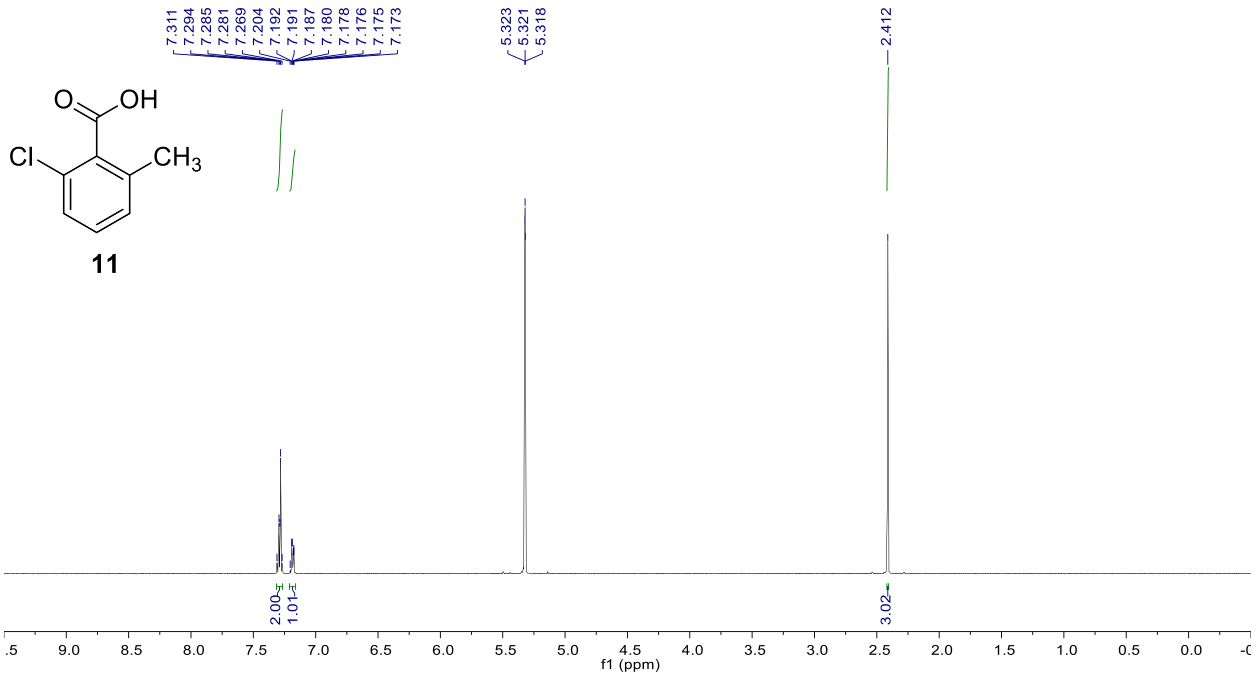

^{1}H NMR spectrum of compound **11** (CD_2Cl_2, 500 MHz)

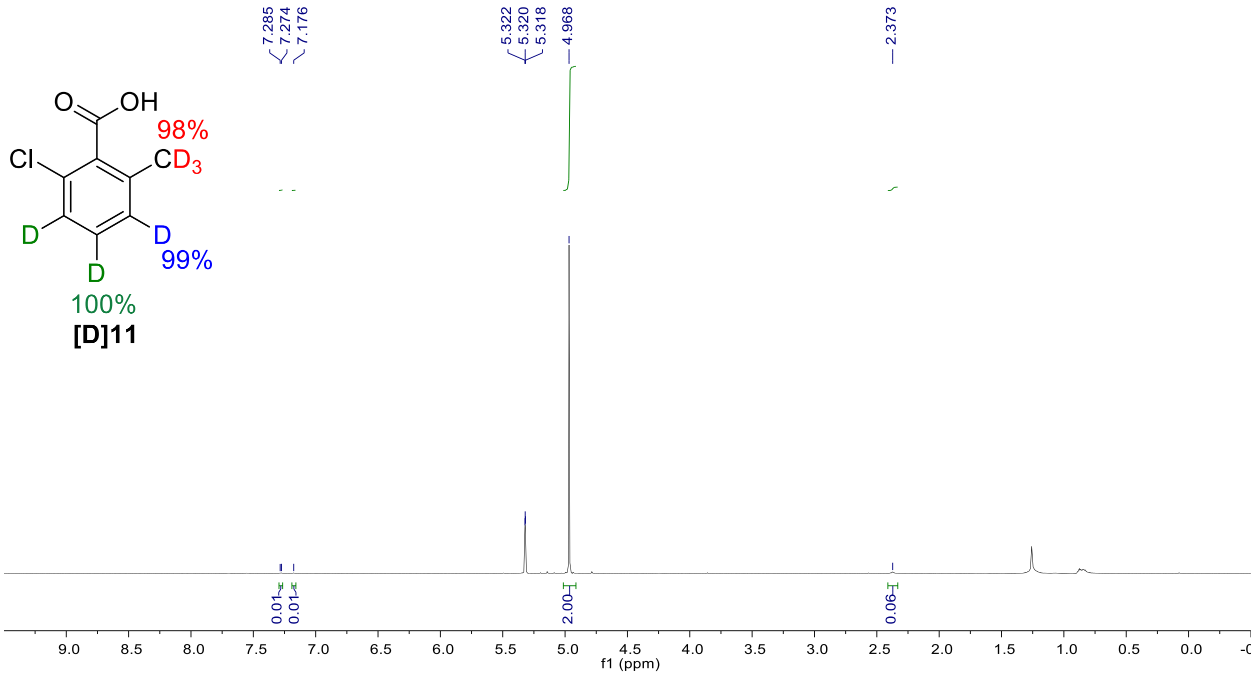

^{1}H NMR spectrum of compound **[D]11** (Procedure A, CD_2Cl_2, 500 MHz)

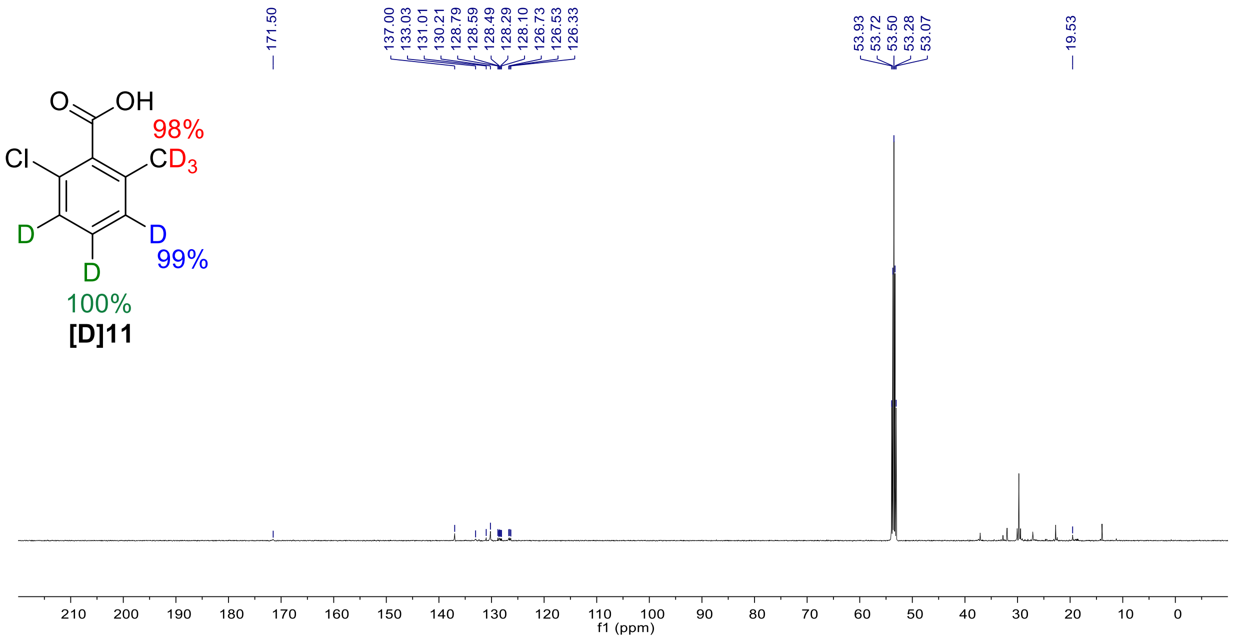

^{13}C NMR spectrum of compound **[D]11** (Procedure A, CD_2Cl_2, 126 MHz)

3-chloro-2-methylbenzoic acid (12)

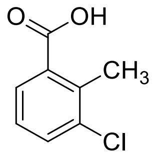

Following the general procedure A, the reaction was set up with 3-chloro-2-methylbenzoic acid (34.1 mg, 0.20 mmol). Purification by flash column chromatography (hexanes/EtOAc/AcOH = 20:1:0.2) provided product **[D]12** as a white solid (32 mg, 91% yield).

Deuterium Incorporation

General procedure A: [LCMS (ESI)] calcd for $C_8D_6ClO_2$ $[M-H]^-$ 5.59 D/molecule, [^{1}H NMR] 5.90 D/molecule.

NMR Data of the Starting Material

^{1}H NMR (500 MHz, CD_2Cl_2) δ 7.88 (dd, J = 7.9, 1.4 Hz, 1H), 7.59 (dd, J = 8.0, 1.3 Hz, 1H), 7.25 (td, J = 7.9, 0.7 Hz, 1H), 2.67 (s, 3H).

NMR Data of the Product

General procedure A: ^{1}H NMR (500 MHz, CD_2Cl_2) δ 7.88 (s, 0.02H, 98% D), 7.59 (s, 0.01H, 99% D), 7.24 (s, 0.01H, 99% D), 2.65-2.60 (m, 0.06H, 98% D); ^{13}C NMR (126 MHz, CD_2Cl_2) δ 172.2, 138.1, 136.2, 133.5-133.1 (1C), 131.2, 129.5-129.0 (1C), 126.4-125.9 (1C), 16.9-16.5 (1C).

Mass Data

LabelChecker Results

Formula: C8 H6 O2 Cl
Mass (monoisotopic): 169.01

Difference Value: 0.007156
Error Sum: 0.085
Error (%): 0.812

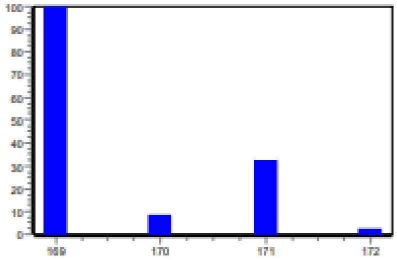

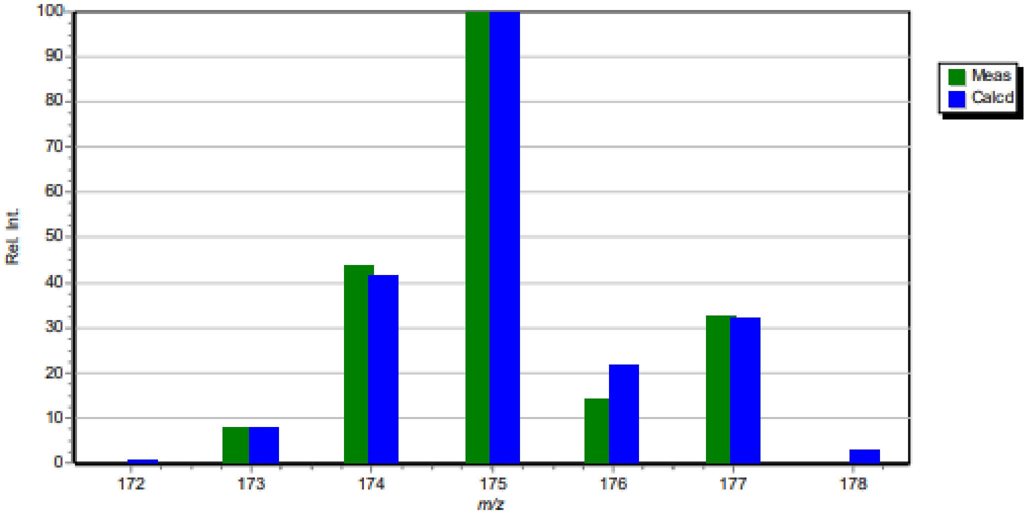

No Convergence! - Max Cycles (20) reached!
Deuterium: 0-fold (%): 0.00 0.00
Deuterium: 1-fold (%): 0.00 0.00
Deuterium: 2-fold (%): 0.00 0.00
Deuterium: 3-fold (%): 0.83 0.54
Deuterium: 4-fold (%): 8.45 5.54
Deuterium: 5-fold (%): 43.21 28.34
Deuterium: 6-fold (%): 100.00 65.58
Label Atom Sum: 5.59 (93.16%)

Isotope List used for fitting data:

m/z	intensity
173.03	15337545
174.04	86532288
175.04	197071712
176.03	27827862
177.04	63328764

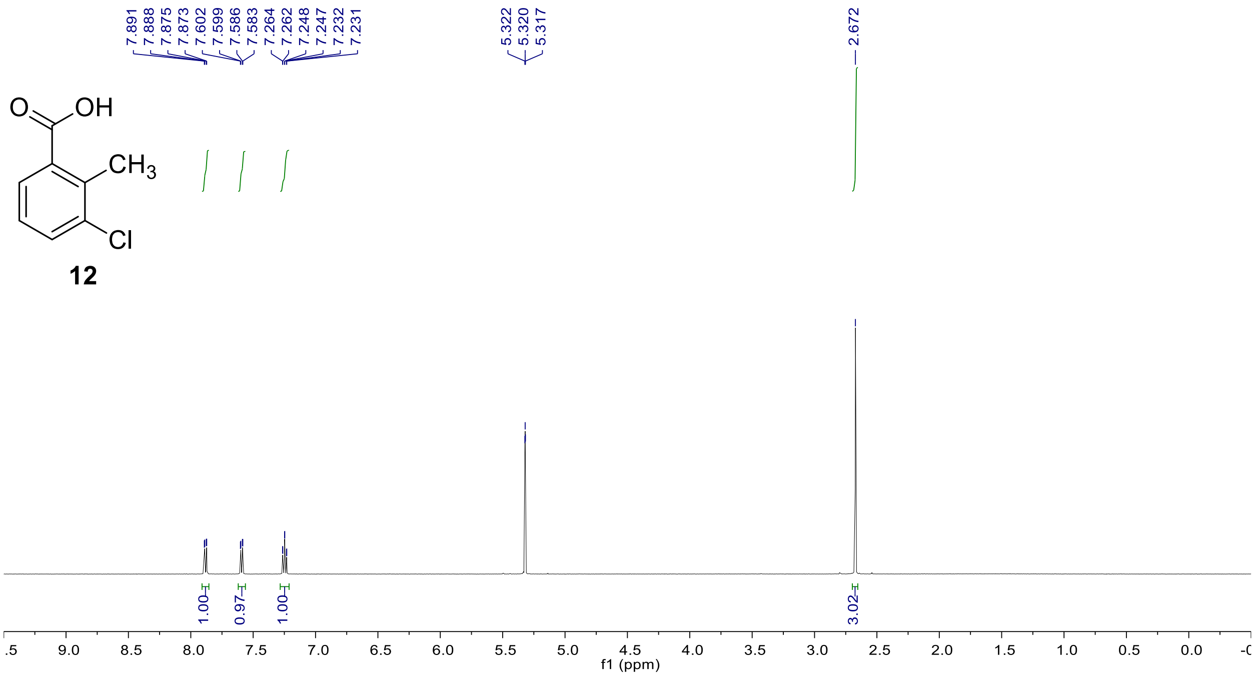

^{1}H NMR spectrum of compound **12** (CD_2Cl_2, 500 MHz)

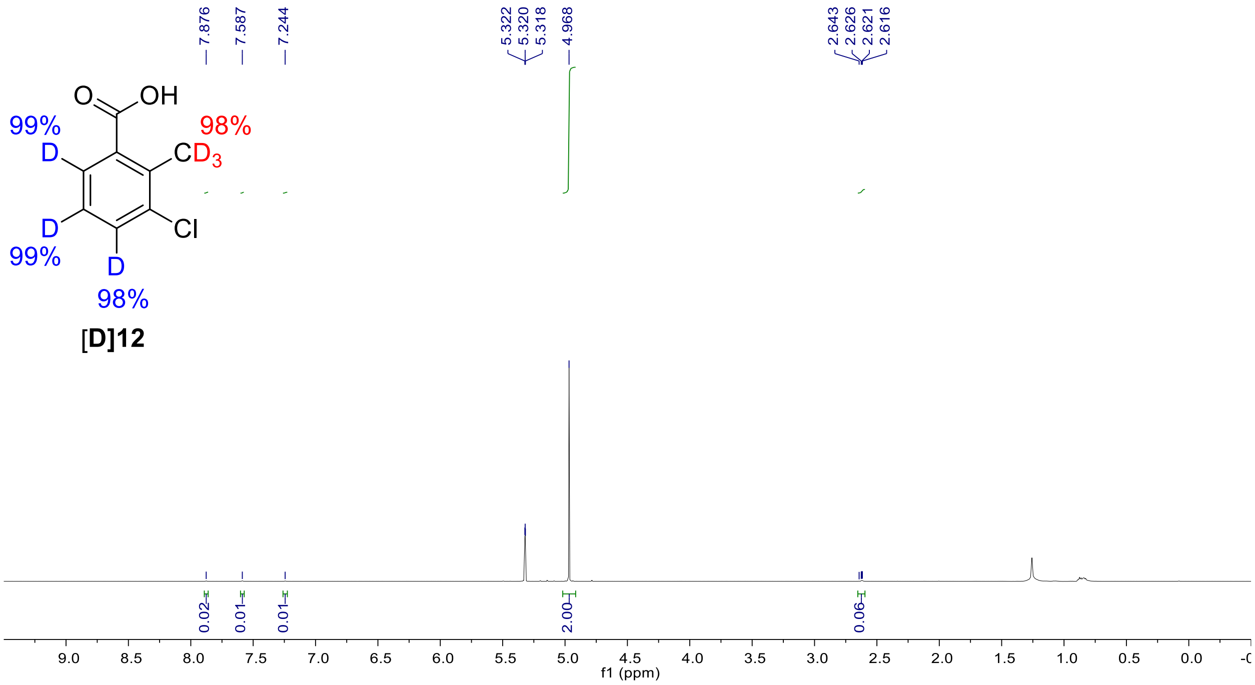

^{1}H NMR spectrum of compound **[D]12** (Procedure A, CD_2Cl_2, 500 MHz)

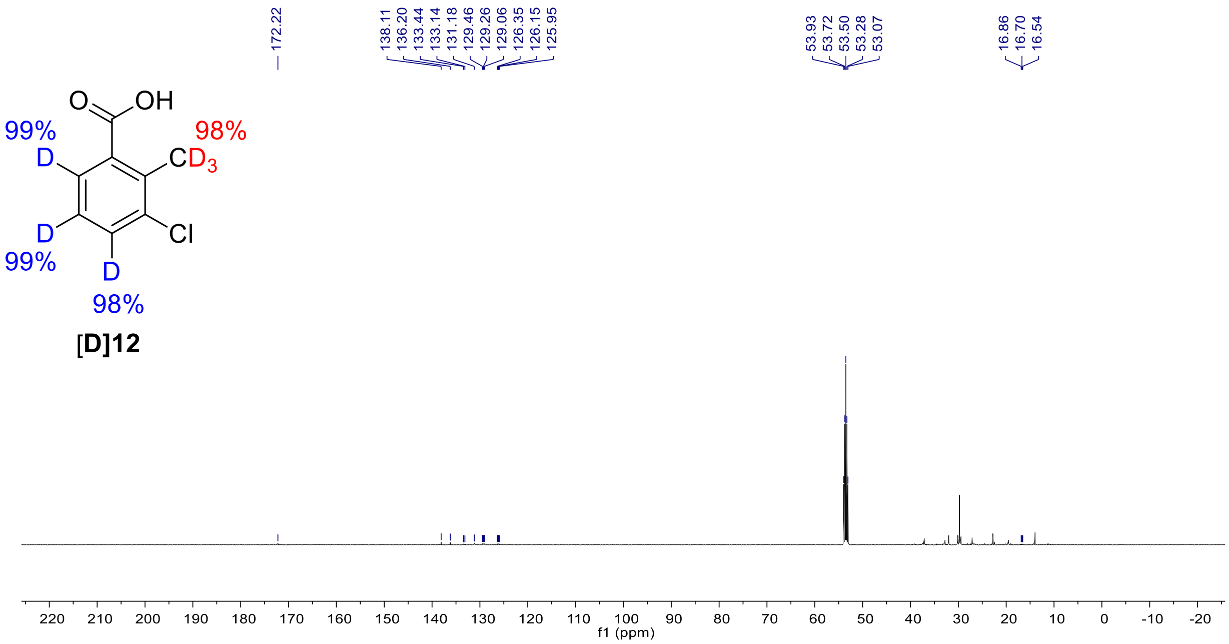

^{13}C NMR spectrum of compound **[D]12** (Procedure A, CD_2Cl_2, 126 MHz)

5-bromo-2-methylbenzoic acid (13)

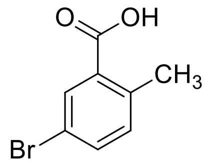

Following the general procedure A, the reaction was set up with 5-bromo-2-methylbenzoic acid (43.0 mg, 0.20 mmol). Purification by flash column chromatography (hexanes/EtOAc/AcOH = 20:1:0.2) provided product **[D]13** as a white solid (43 mg, 97% yield).

Deuterium Incorporation

General procedure A: [LCMS (ESI)] calcd for $C_8D_6O_2Br$ $[M-H]^-$ 5.49 D/molecule, [^{1}H NMR] 5.86 D/molecule.

NMR Data of the Starting Material

^{1}H NMR (500 MHz, $CDCl_3$) δ 8.19 (d, *J* = 2.3 Hz, 1H), 7.57 (dd, *J* = 8.2, 2.2 Hz, 1H), 7.16 (d, *J* = 8.2, 1H), 2.60 (s, 3H).

NMR Data of the Product

General procedure A: ^{1}H NMR (500 MHz, $CDCl_3$) δ 8.19 (s, 0.05H, 95% D), 7.57 (s, 0.01H, 99% D), 7.16 (s, 0.02H, 98% D), 2.59-2.55 (m, 0.05H, 98% D); ^{13}C NMR (126 MHz, $CDCl_3$) δ 171.9, 140.3, 135.9-135.4 (1C), 134.4-133.9 (1C), 133.6-133.1 (1C), 131.1, 119.3-119.2 (1C), 21.2-20.8 (1C).

Mass Data

LabelChecker Results

Formula: C8 H6 O2 Br
Mass (monoisotopic): 212.96

Difference Value: 0.009576
Error Sum: 0.098
Error (%): 0.000

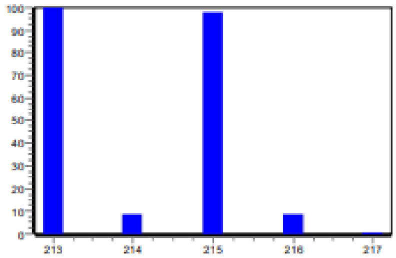

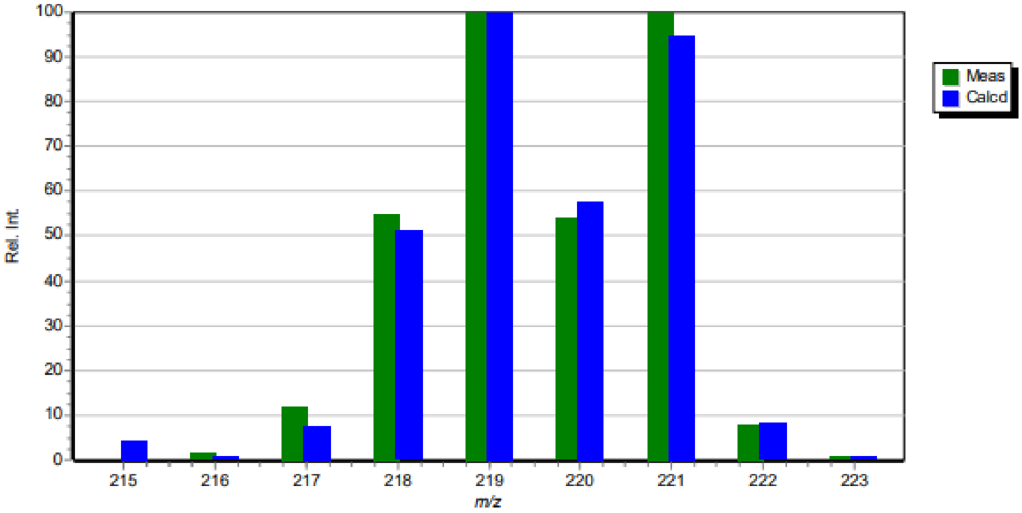

No Convergence! - Max Iterations (3000) reached!
Deuterium: 0-fold (%): 0.00 0.00
Deuterium: 1-fold (%): 0.46 0.28
Deuterium: 2-fold (%): 4.90 3.00
Deuterium: 3-fold (%): 0.00 0.00
Deuterium: 4-fold (%): 3.41 2.09
Deuterium: 5-fold (%): 54.64 33.44
Deuterium: 6-fold (%): 100.00 61.20
Label Atom Sum: 5.49 (91.50%)

Isotope List used for fitting data:

m/z	intensity
215.97	7712837
216.98	73374880
217.99	339104928
218.99	615141120
219.99	331012800
220.99	614365632
221.99	49069932
223.00	3802952

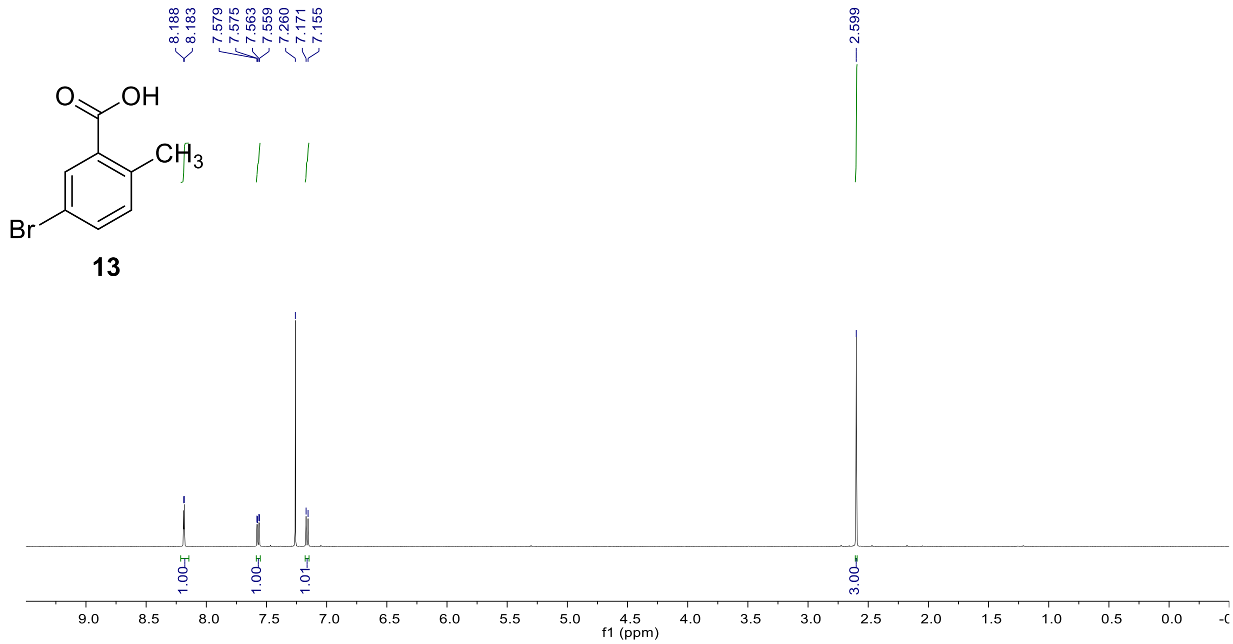

^{1}H NMR spectrum of compound **13** ($CDCl_3$, 500 MHz)

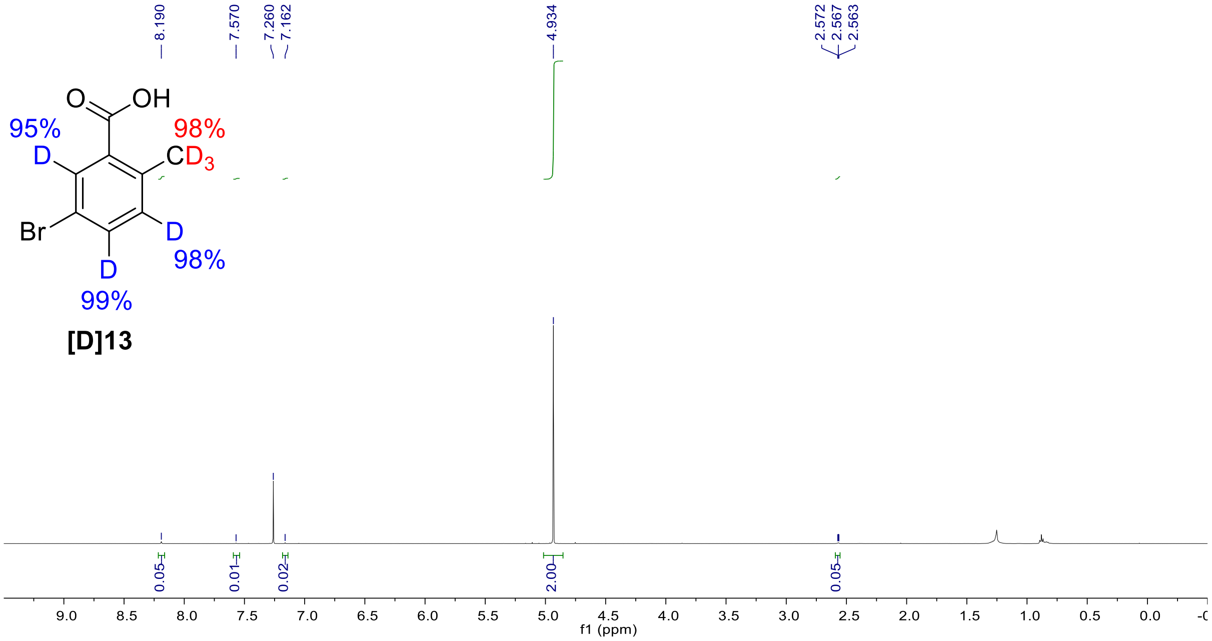

^{1}H NMR spectrum of compound **[D]13** (Procedure A, $CDCl_3$, 500 MHz)

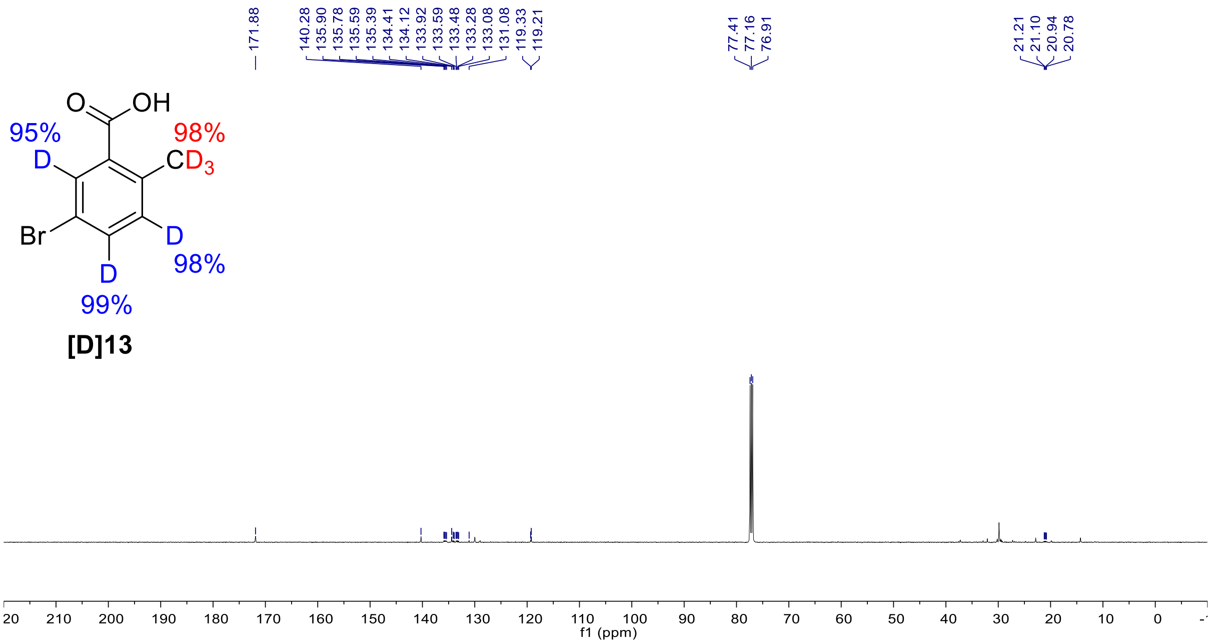

^{13}C NMR spectrum of compound **[D]13** (Procedure A, $CDCl_3$, 126 MHz)

4-bromo-2-methylbenzoic acid (14)

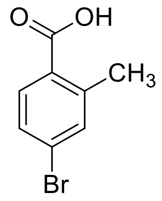

Following the general procedure A, the reaction was set up with 4-bromo-2-methylbenzoic acid (42.8 mg, 0.20 mmol). Purification by flash column chromatography (hexanes/EtOAc/AcOH = 20:1:0.15) provided product **[D]14** as a white solid (41 mg, 93% yield)

Deuterium Incorporation

General procedure A: [LCMS (ESI)] calcd for $C_8D_6O_2Br$ $[M\text{-}H]^-$ 5.67 D/molecule, [^{1}H NMR] 5.86 D/molecule.

NMR Data of the Starting Material

^{1}H NMR (500 MHz, $CDCl_3$) δ 7.93 (d, *J* = 8.4 Hz, 1H), 7.46 (s, 1H), 7.43 (dd, *J* = 8.3, 2.1 Hz, 1H), 2.63 (s, 3H).

NMR Data of the Product

General procedure A: ^{1}H NMR (500 MHz, $CDCl_3$) δ 7.93 (s, 0.03H, 97% D), 7.46 (s, 0.03H, 97% D), 7.43 (s, 0.02H, 98% D), 2.62-2.59 (m, 0.06H, 98% D); ^{13}C NMR (126 MHz, $CDCl_3$) δ 172.3, 143.5, 135.0-134.5 (1C), 133.1-132.7 (1C), 129.2-128.8 (1C), 127.9, 127.2, 21.4-21.0 (1C).

Mass Data

LabelChecker Results

Formula: C8 H6 O2 Br
Mass (monoisotopic): 212.96

Difference Value: 0.011590
Error Sum: 0.108
Error (%): 0.000

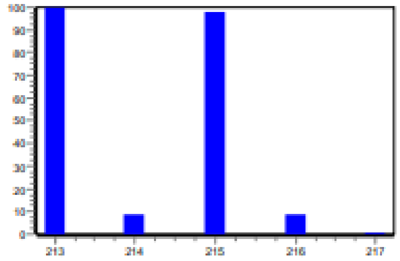

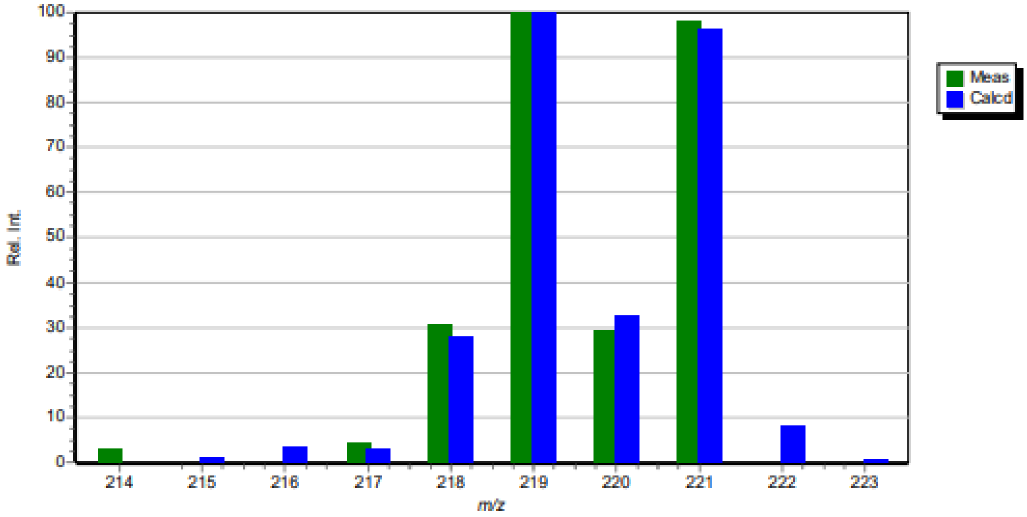

No Convergence! - Max Iterations (3000) reached!
Deuterium: 0-fold (%): 0.00 0.00
Deuterium: 1-fold (%): 0.14 0.10
Deuterium: 2-fold (%): 1.20 0.91
Deuterium: 3-fold (%): 3.20 2.43
Deuterium: 4-fold (%): 1.56 1.19
Deuterium: 5-fold (%): 25.71 19.50
Deuterium: 6-fold (%): 100.00 75.86
Label Atom Sum: 5.67 (94.44%)

Isotope List used for fitting data:

m/z	intensity
213.96	203826
214.97	10163
215.96	16204
216.98	259486
217.99	2038854
218.99	6557539
219.98	1935668
220.99	6454920

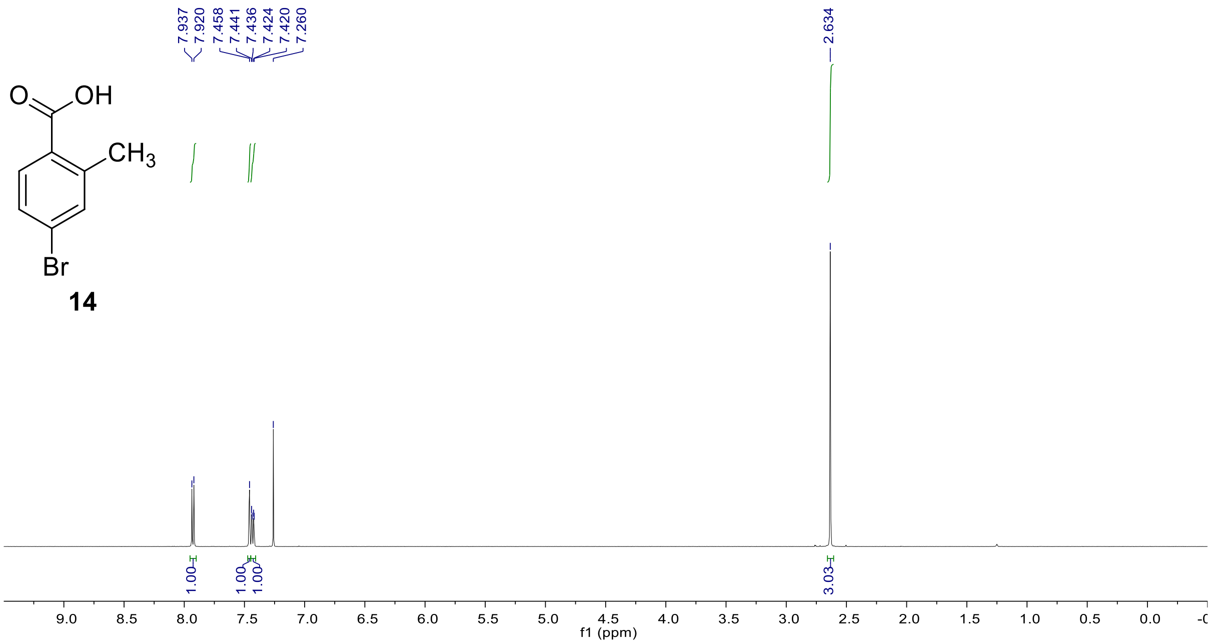

^{1}H NMR spectrum of compound **14** ($CDCl_3$, 500 MHz)

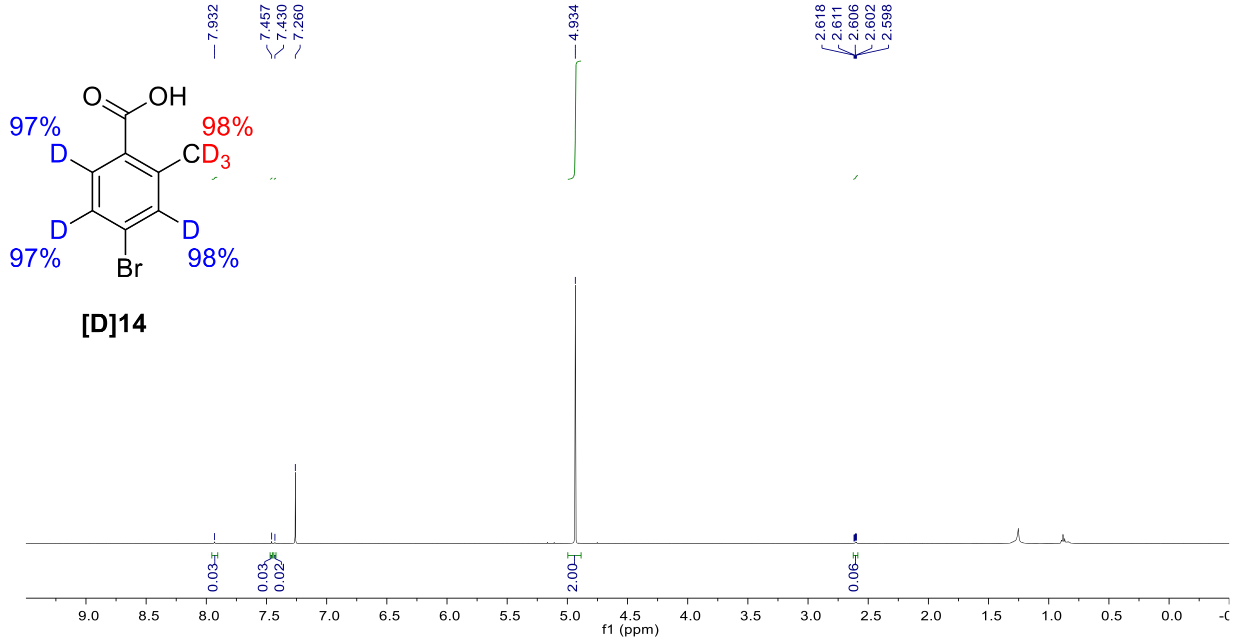

^{1}H NMR spectrum of compound **[D]14** (Procedure A, $CDCl_3$, 500 MHz)

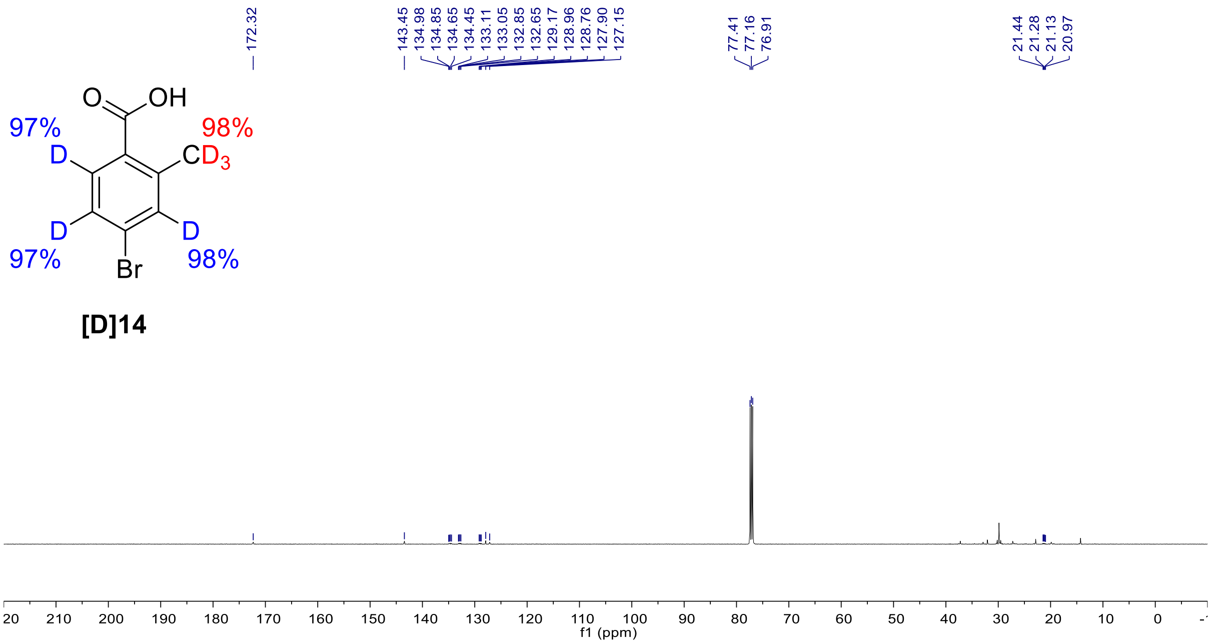

[D]14

^{13}C NMR spectrum of compound **[D]14** (Procedure A, $CDCl_3$, 126 MHz)

3-bromo-2-methylbenzoic acid (15)

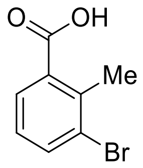

Following the general procedure A, the reaction was set up with 3-bromo-2-methylbenzoic acid (43.0 mg, 0.20 mmol). Purification by flash column chromatography (hexanes/EtOAc/AcOH = 20:1:0.2) provided product **[D]15** as a white solid (34 mg, 77% yield).

Deuterium Incorporation

General procedure A: [LCMS (ESI)] calcd for $C_8D_6O_2Br$ $[M-H]^-$ 5.54 D/molecule, [^{1}H NMR] 5.81 D/molecule.

NMR Data of the Starting Material

^{1}H NMR (500 MHz, $CDCl_3$) δ 7.93 (d, *J* = 7.9 Hz, 1H), 7.77 (dd, *J* = 8.0, 1.3 Hz, 1H), 7.15 (t, *J* = 7.9 Hz, 1H), 2.73 (s, 3H).

NMR Data of the Product

General procedure A: ^{1}H NMR (500 MHz, $CDCl_3$) δ 7.93 (s, 0.03H, 97% D), 7.77 (s, 0.02H, 98% D), 7.15 (s, 0.02H, 98% D), 2.71-2.66 (m, 0.11H, 96% D); ^{13}C NMR (126 MHz, CD_2Cl_2) δ 173.1, 139.9, 137.1-136.6 (1C), 131.1, 130.3-129.9 (1C), 127.4, 126.8-126.3 (1C), 20.7-19.9 (1C).

Mass Data

LabelChecker Results

Formula:	C8 H6 O2 Br
Mass (monoisotopic):	212.96
Difference Value:	0.013398
Error Sum:	0.116
Error (%):	0.000

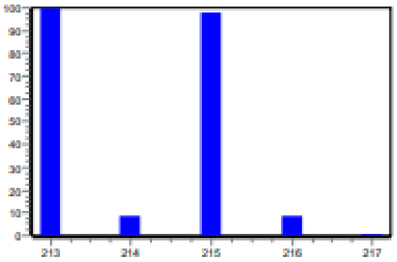

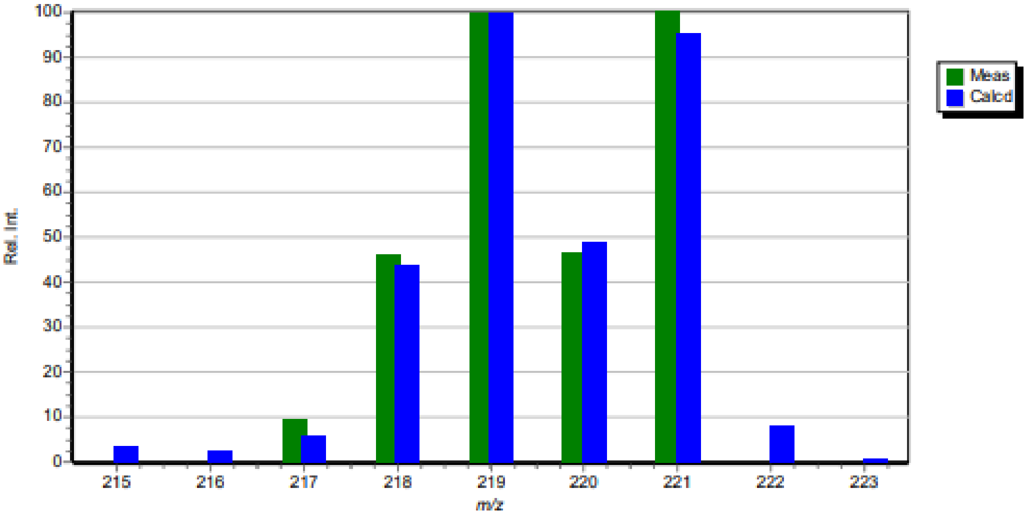

No Convergence! - Max Iterations (3000) reached!
Deuterium: 0-fold (%): 0.00 0.00
Deuterium: 1-fold (%): 0.00 0.00
Deuterium: 2-fold (%): 3.65 2.40
Deuterium: 3-fold (%): 2.22 1.46
Deuterium: 4-fold (%): 2.32 1.53
Deuterium: 5-fold (%): 43.98 28.90
Deuterium: 6-fold (%): 100.00 65.71
Label Atom Sum: 5.54 (92.34%)

Isotope List used for fitting data:

m/z	intensity
216.98	634284
217.99	3173064
218.99	6846488
219.98	3178111
220.99	6873465

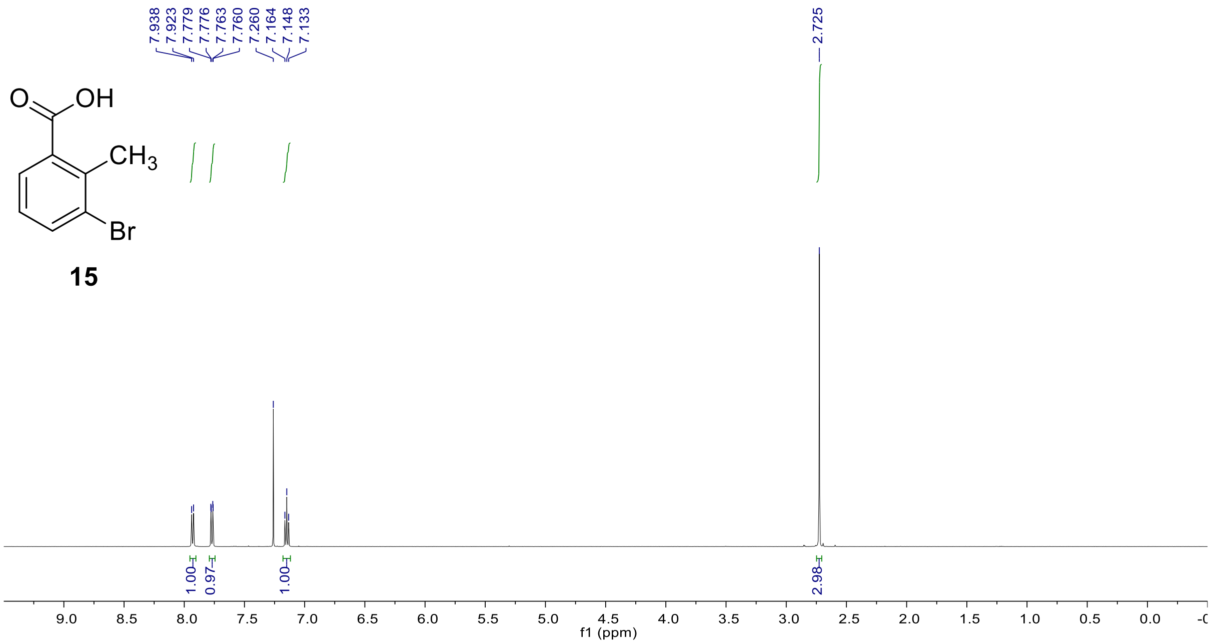

^{1}H NMR spectrum of compound **15** ($CDCl_3$, 126 MHz)

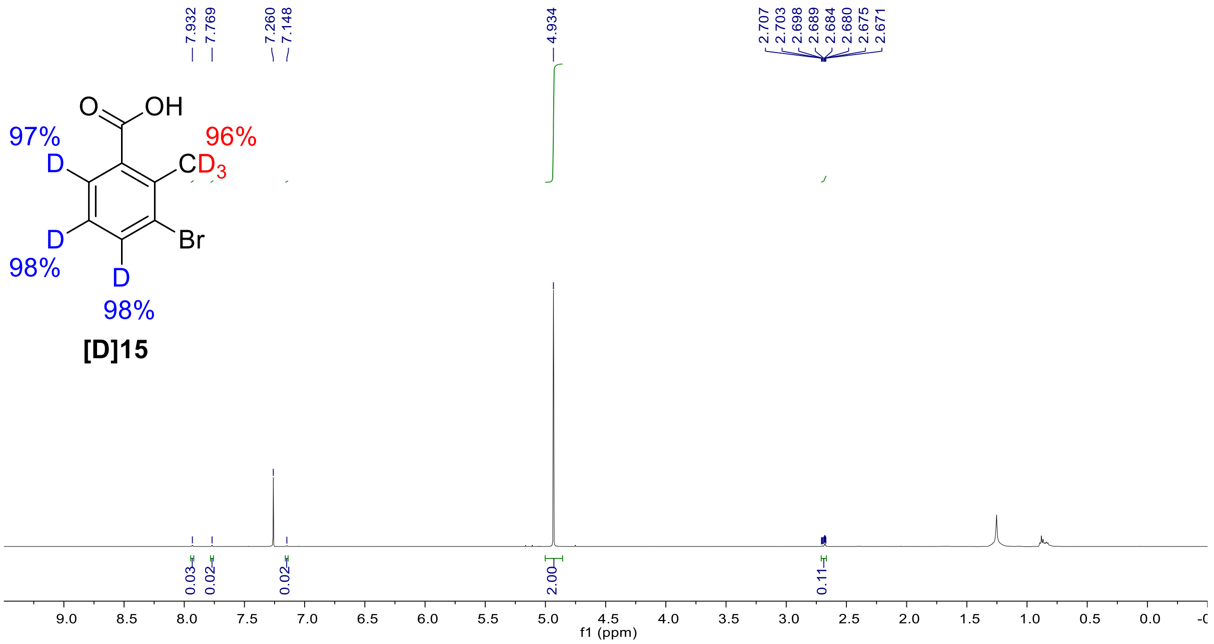

^{1}H NMR spectrum of compound **[D]15** (Procedure A, $CDCl_3$, 500 MHz)

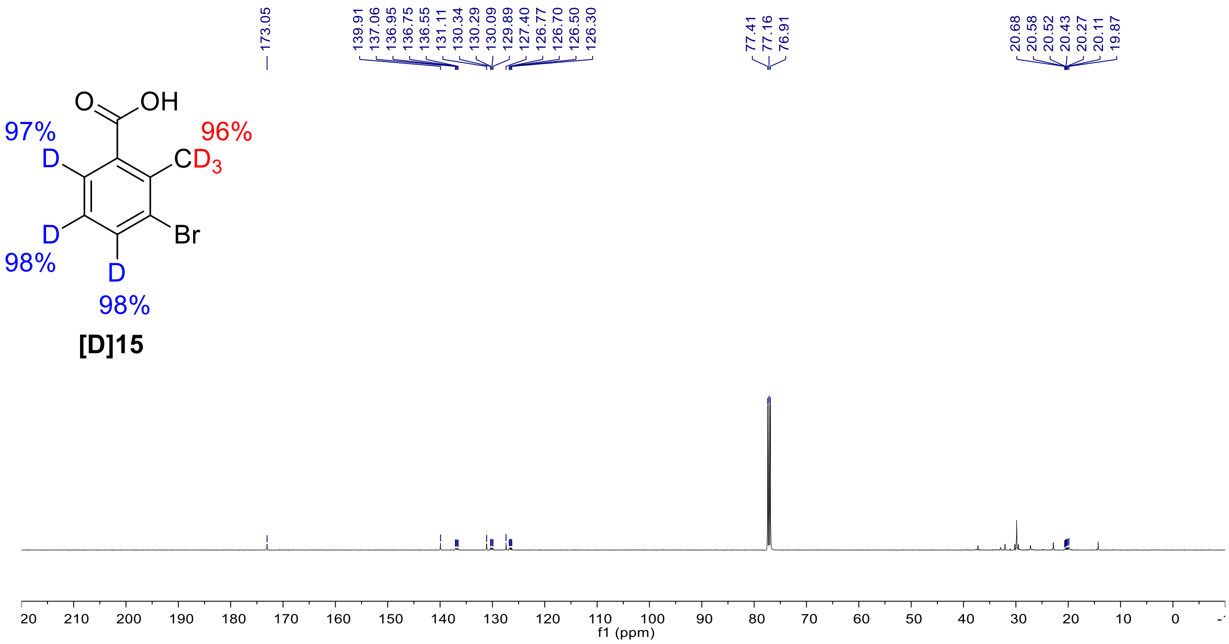

^{13}C NMR spectrum of compound **[D]15** (Procedure A, $CDCl_3$, 126 MHz)

3-methoxy-2-methylbenzoic acid (16)

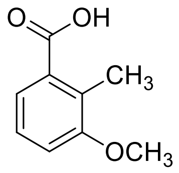

Following the general procedure A, the reaction was set up with 3-methoxy-2-methylbenzoic acid (33.2 mg, 0.20 mmol). Purification by flash column chromatography (hexanes/EtOAc = 15:1) provided product **[D]16** as a white solid (28 mg, 81% yield).

Deuterium Incorporation

General procedure A: [LCMS (ESI)] calcd for $C_9H_3D_6O_3$ $[M-H]^-$ 5.66 D/molecule, [^{1}H NMR] 5.69 D/molecule.

NMR Data of the Starting Material

^{1}H NMR (500 MHz, CD_2Cl_2) δ 7.55 (dd, *J* = 7.9, 1.2 Hz, 1H), 7.25 (t, *J* = 8.0 Hz, 1H), 7.08 (d, *J* = 8.2 Hz, 1H), 3.86 (s, 3H), 2.48 (s, 3H).

NMR Data of the Product

General procedure A: ^{1}H NMR (500 MHz, CD_2Cl_2) δ 7.56 (s, 0.04H, 96% D), 7.25 (s, 0.05H, 95% D), 7.08 (s, 0.10H, 90% D), 3.86 (s, 2.97H, 1% D), 2.45-2.42 (m, 0.12H, 96% D); ^{13}C NMR (126 MHz, CD_2Cl_2) δ 173.4, 158.3, 130.1, 129.4, 126.0-125.5 (1C), 122.6-122.1 (1C), 114.3-113.8 (1C), 29.8, 22.8.

Mass Data

LabelChecker Results

Formula: C9 H9 O3
Mass (monoisotopic): 165.06

Difference Value: 0.000118
Error Sum: 0.011
Error (%): 0.181

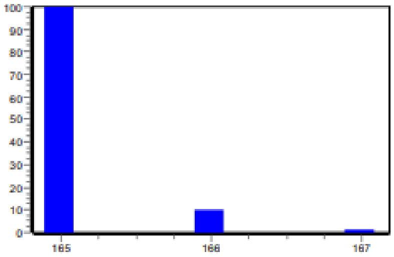

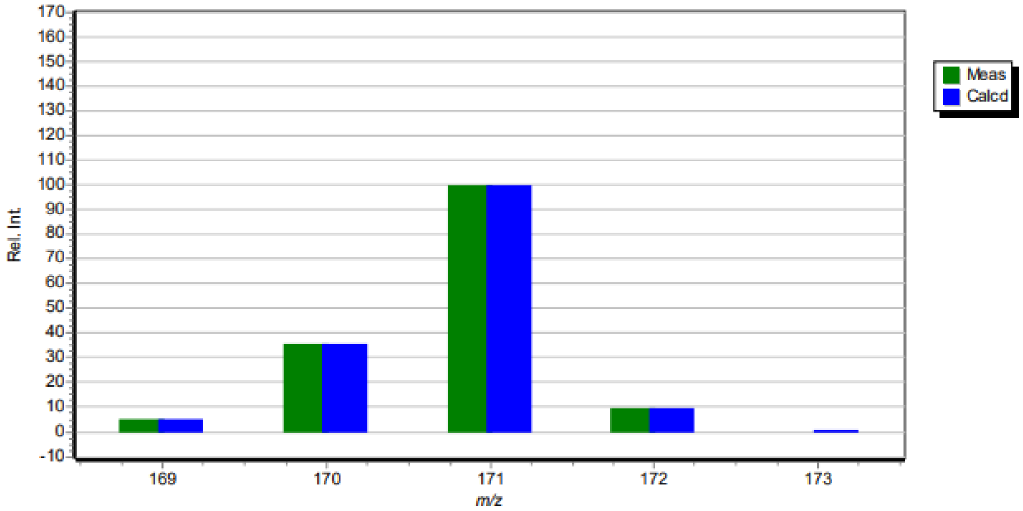

Deuterium: 0-fold (%): 0.00 0.00
Deuterium: 1-fold (%): 0.08 0.06
Deuterium: 2-fold (%): 0.01 0.01
Deuterium: 3-fold (%): 0.28 0.20
Deuterium: 4-fold (%): 5.20 3.65
Deuterium: 5-fold (%): 36.73 25.81
Deuterium: 6-fold (%): 100.00 70.27
Label Atom Sum: 5.66 (62.89%)

Isotope List used for fitting data:

m/z	intensity
167.07	25431
168.07	467039
169.08	8561891
170.09	61034224
171.09	169902960
172.10	16331521

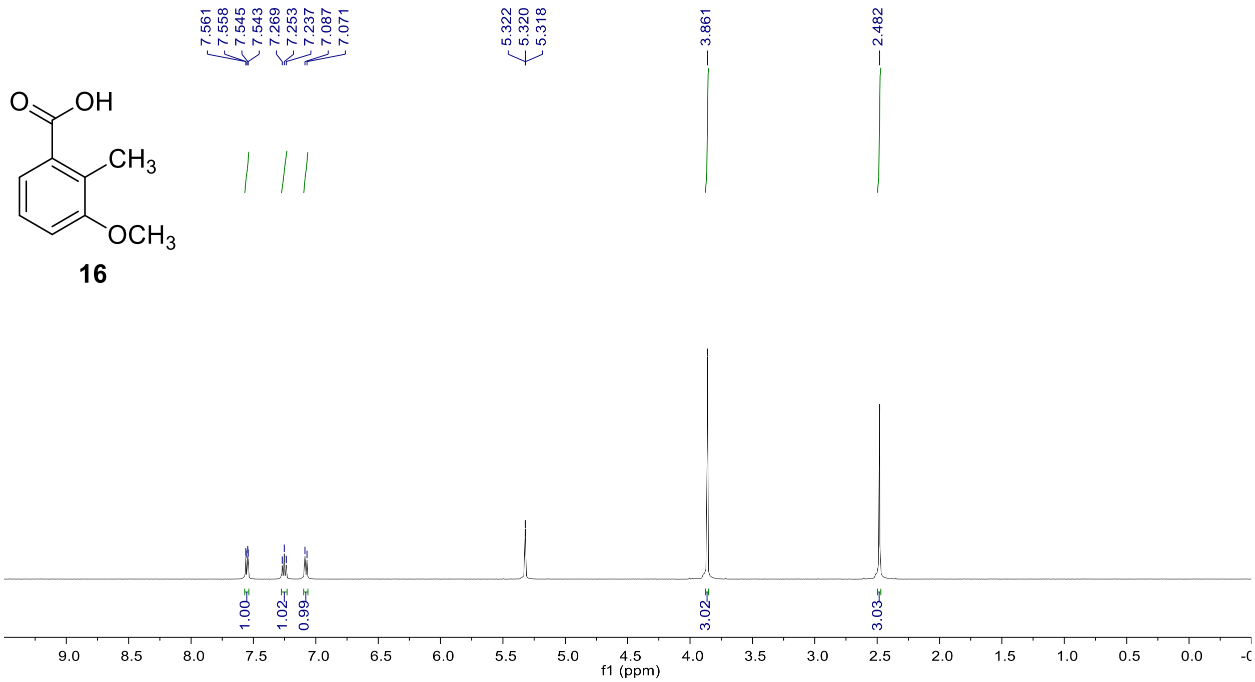

^{1}H NMR spectrum of compound **16** (CD_2Cl_2, 500 MHz)

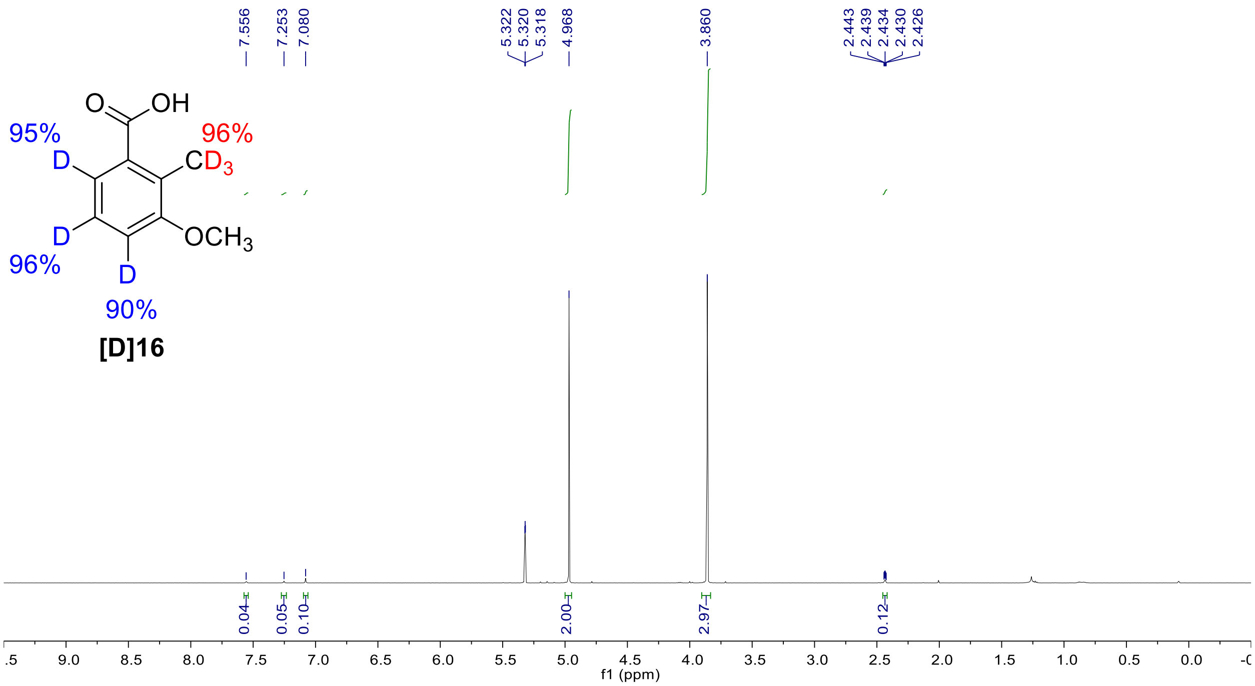

^{1}H NMR spectrum of compound **[D]16** (Procedure A, CD_2Cl_2, 500 MHz)

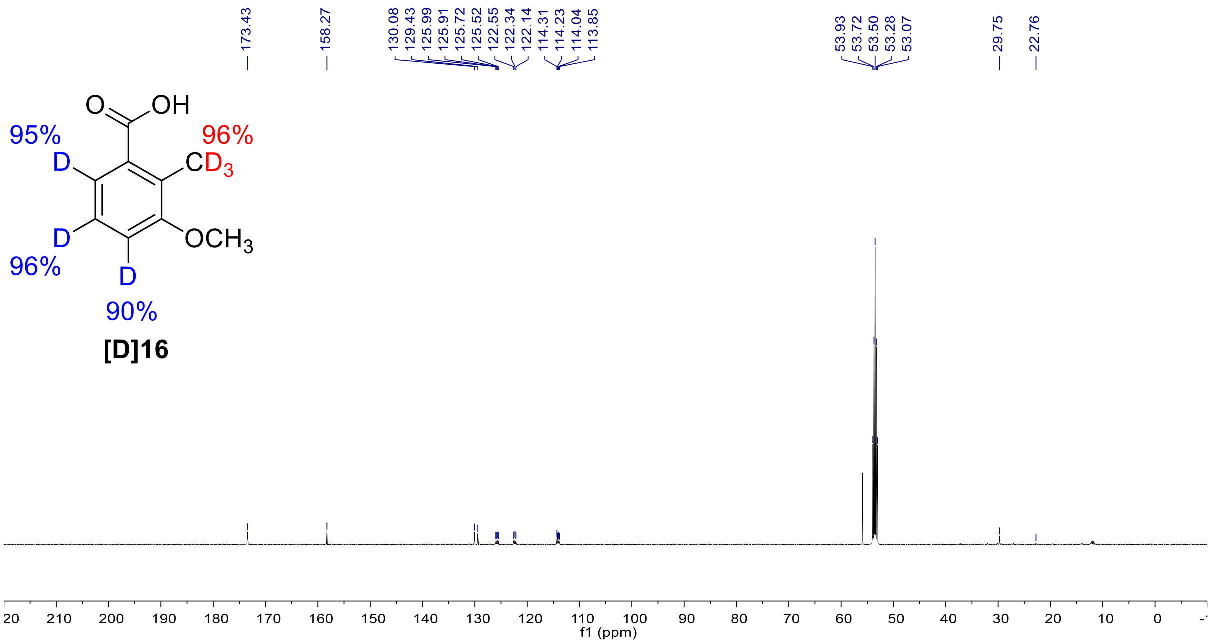

^{13}C NMR spectrum of compound **[D]16** (Procedure A, CD_2Cl_2, 126 MHz)

2-methyl-1-naphthoic acid (17)

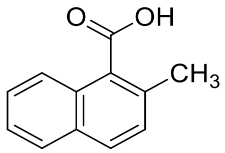

Following the general procedure A, the reaction was set up with 2-methyl-1-naphthoic acid (37.2 mg, 0.20 mmol). Purification by flash column chromatography (hexanes/EtOAc = 4:1) provided product **[D]17** as an orange solid (36 mg, 92% yield).

Deuterium Incorporation

General procedure A: [LCMS (ESI)] calcd for $C_{12}D_9O_2$ $[M-H]^-$ 8.34 D/molecule, [^1H NMR] 8.33 D/molecule.

NMR Data of the Starting Material

^1H NMR (500 MHz, $CDCl_3$) δ 8.11-8.07 (m, 1H), 7.85 (t, *J* = 7.6 Hz, 2H), 7.57 (d, *J* = 7.7 Hz, 1H), 7.49 (t, *J* = 7.5 Hz, 1H), 7.37 (d, *J* = 8.4 Hz, 1H), 2.66 (s, 3H).

NMR Data of the Product

General procedure A: ^1H NMR (500 MHz, $CDCl_3$) δ 8.15 (s, 0.07H, 93% D), 7.86 (d, *J* = 6.8 Hz, 0.15H, 93% D), 7.57 (s, 0.07H, 93% D), 7.49 (s, 0.07H, 93% D), 7.37 (s, 0.08H, 92% D), 2.65 (s, 0.23H, 92% D); ^{13}C NMR (126 MHz, $CDCl_3$) δ 175.6, 134.7, 131.8-131.7 (1C), 130.5-130.0 (2C), 128.6-127.6 (3C), 127.2-126.7 (1C), 125.5-125.0 (1C), 124.7-124.2 (1C), 20.4-19.7 (1C).

Mass Data

LabelChecker Results

Formula: C12 H9 O2
Mass (monoisotopic): 185.06

Difference Value: 0.000103
Error Sum: 0.010
Error (%): 0.067

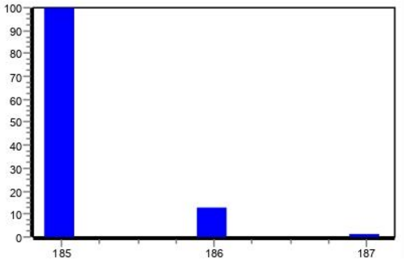

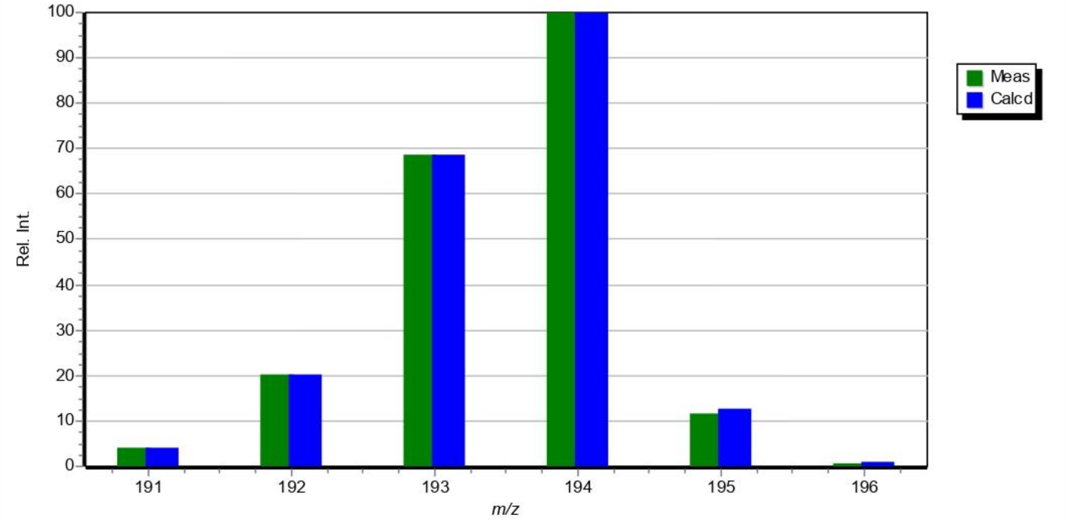

Deuterium: 0-fold (%): 0.06 0.03
Deuterium: 1-fold (%): 0.01 0.01
Deuterium: 2-fold (%): 0.00 0.00
Deuterium: 3-fold (%): 0.00 0.00
Deuterium: 4-fold (%): 0.00 0.00
Deuterium: 5-fold (%): 0.01 0.00
Deuterium: 6-fold (%): 4.49 2.26
Deuterium: 7-fold (%): 21.85 10.99
Deuterium: 8-fold (%): 72.45 36.43
Deuterium: 9-fold (%): 100.00 50.28
Label Atom Sum: 8.34 (92.72%)

Isotope List used for fitting data:

m/z	intensity
191.10	1763598
192.10	8824004
193.11	29639110
194.12	43166416
195.12	5112804
196.12	261918

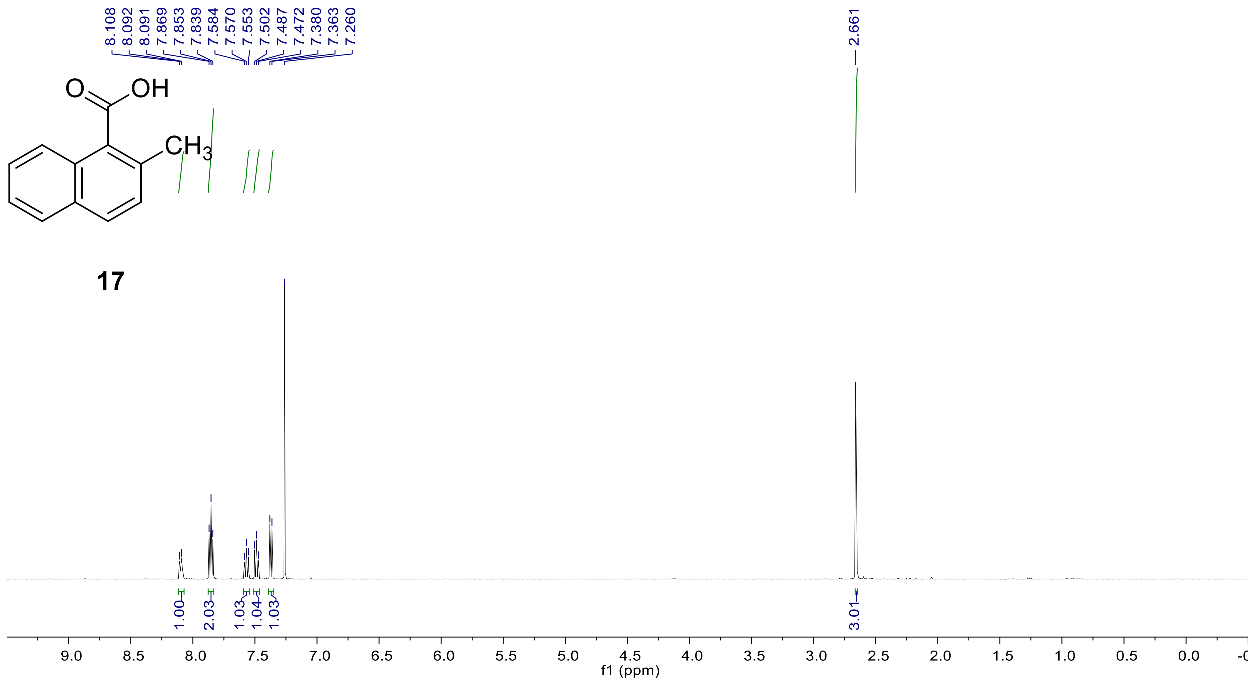

^{1}H NMR spectrum of compound **17** ($CDCl_3$, 500 MHz)

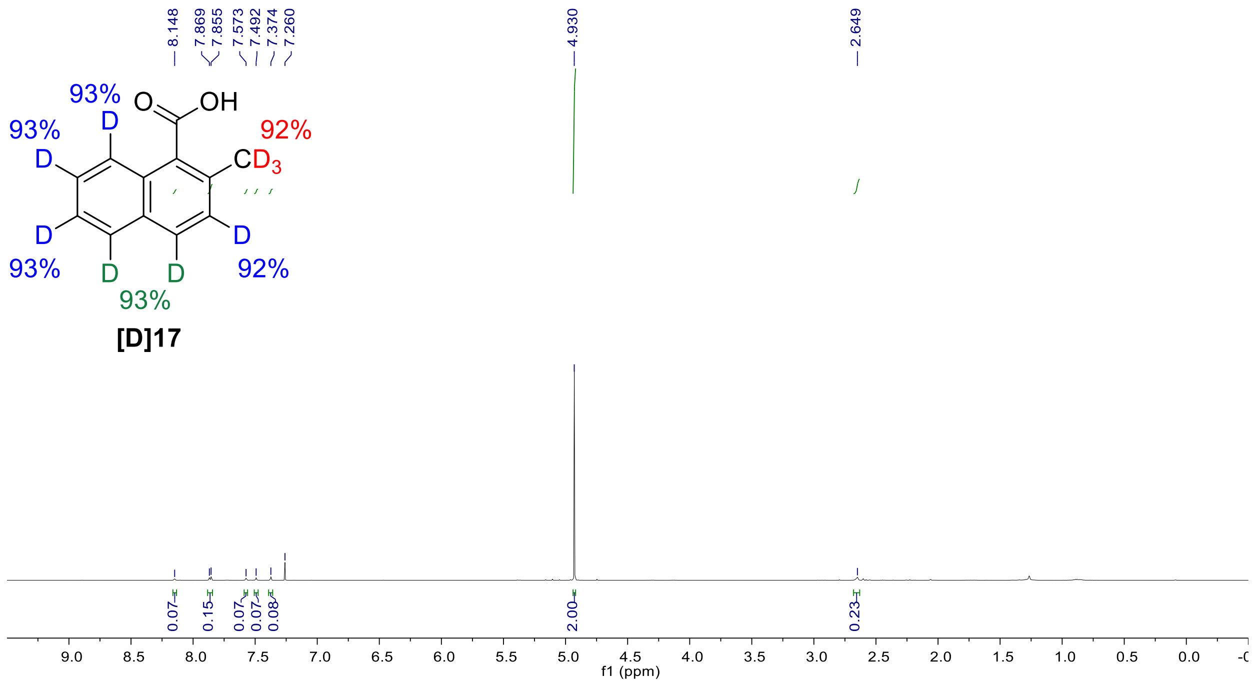

^{1}H NMR spectrum of compound **[D]17** (Procedure A, $CDCl_3$, 500 MHz)

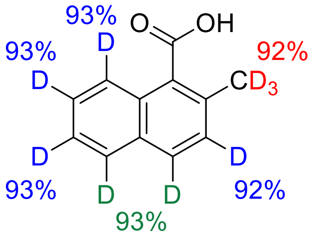

[D]17

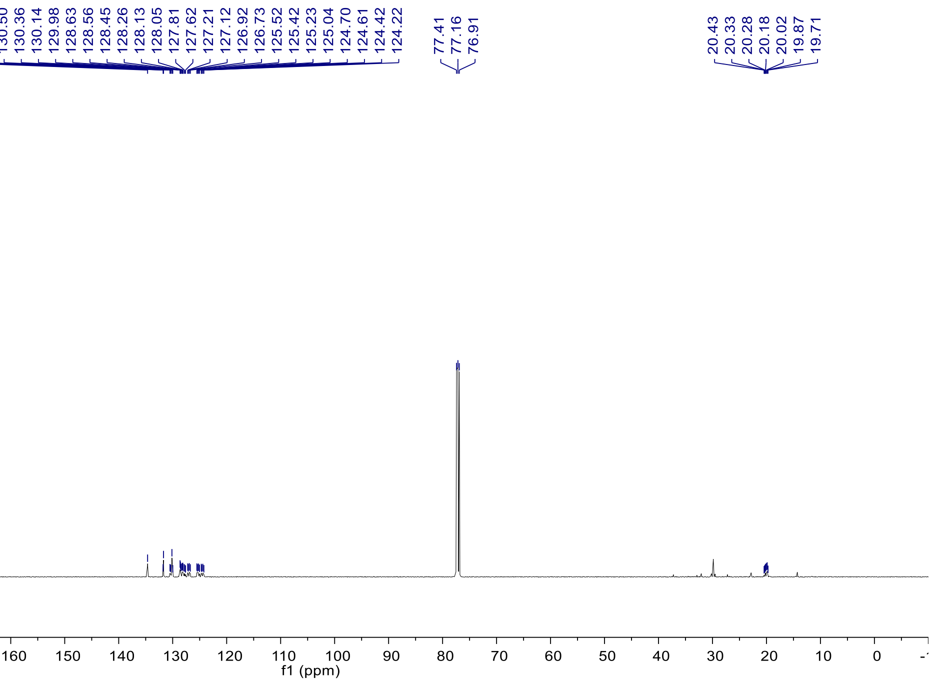

^{13}C NMR spectrum of compound **[D]17** (Procedure A, $CDCl_3$, 126 MHz)

benzoic acid (18)

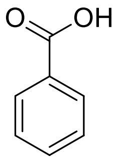

Following the general procedure A, the reaction was set up with benzoic acid (24.4 mg, 0.20 mmol). Purification by flash column chromatography (hexanes/EtOAc = 10:1) provided product **[D]18** as a white solid (21 mg, 83% yield).

Deuterium Incorporation

General procedure A: [LCMS (ESI)] calcd for $C_7D_5O_2$ $[M-H]^-$ 4.78 D/molecule, [^{1}H NMR] 4.80 D/molecule.

NMR Data of the Starting Material

^{1}H NMR (500 MHz, $CDCl_3$) δ 8.16-8.11 (m, 2H), 7.65-7.60 (m, 1H), 7.52-7.47 (m, 2H).

NMR Data of the Product

General procedure A: ^{1}H NMR (500 MHz, $CDCl_3$) δ 8.13 (s, 0.09H, 96% D), 7.63 (s, 0.04H, 96% D), 7.49 (s, 0.09H, 96% D); ^{13}C NMR (126 MHz, $CDCl_3$) δ 172.4, 133.8-133.3 (1C), 130.3-129.6 (2C), 129.3, 128.4-127.9 (2C).

Mass Data

LabelChecker Results

Formula: C7 H5 O2
Mass (monoisotopic): 121.03

Difference Value: 0.000044
Error Sum: 0.007
Error (%): 0.093

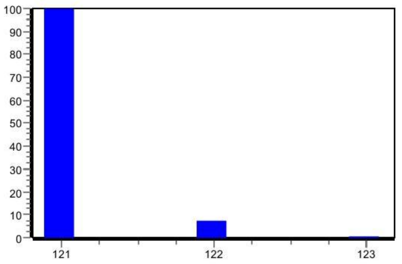

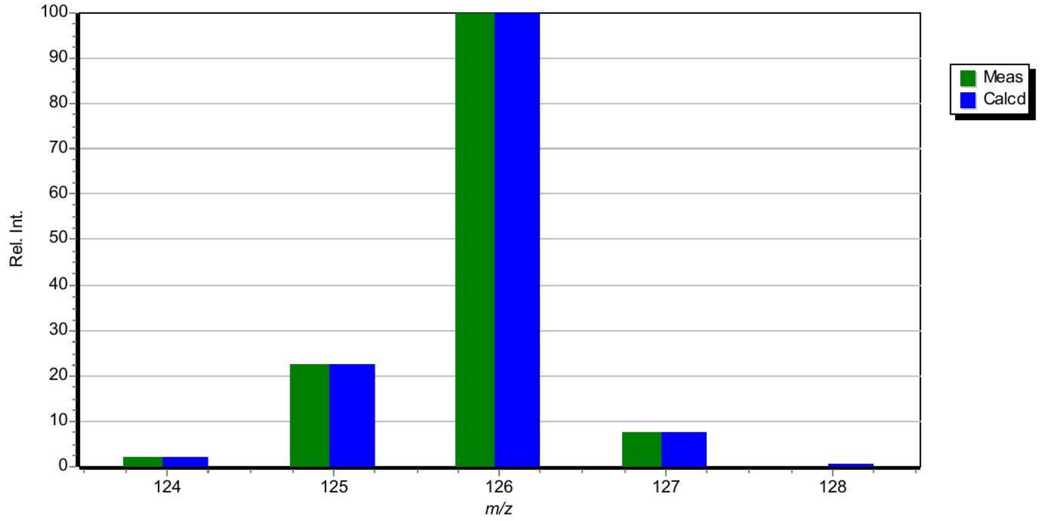

Deuterium: 0-fold (%): 0.00 0.00
Deuterium: 1-fold (%): 0.00 0.00
Deuterium: 2-fold (%): 0.00 0.00
Deuterium: 3-fold (%): 2.12 1.69
Deuterium: 4-fold (%): 23.07 18.43
Deuterium: 5-fold (%): 100.00 79.88
Label Atom Sum: 4.78 (95.64%)

Isotope List used for fitting data:

m/z intensity
124.05 250275
125.05 2742917
126.06 12020004
127.06 912112

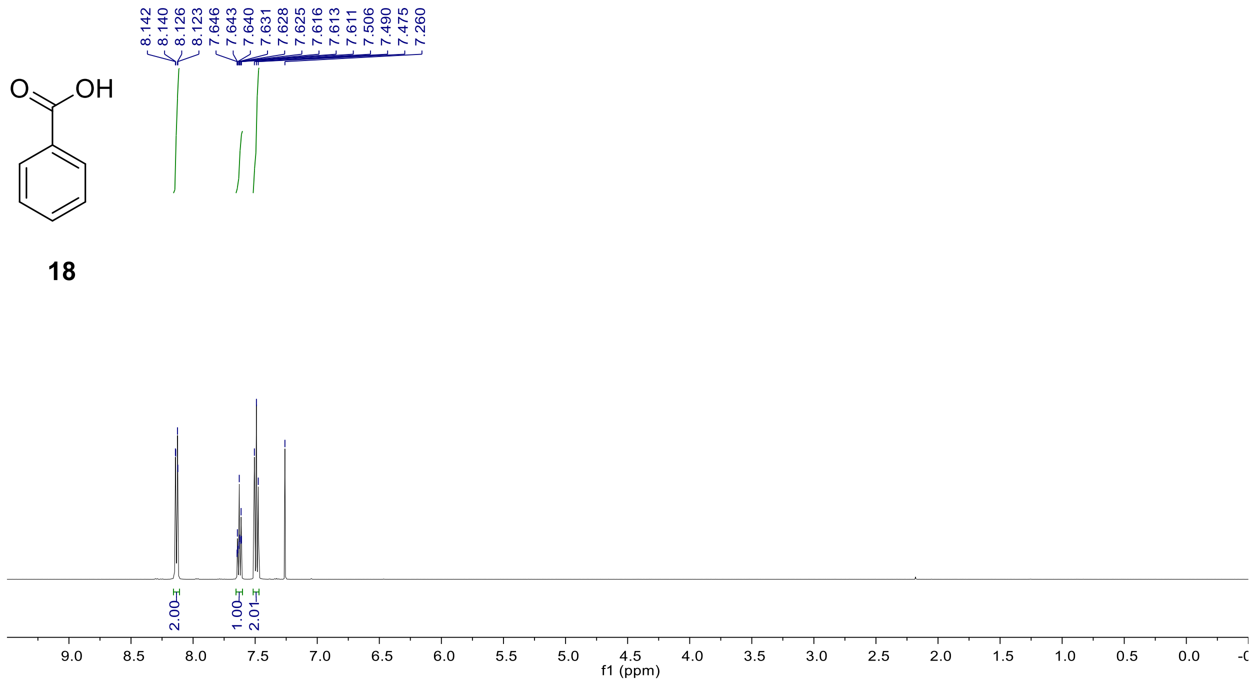

^{1}H NMR spectrum of compound **18** ($CDCl_3$, 500 MHz)

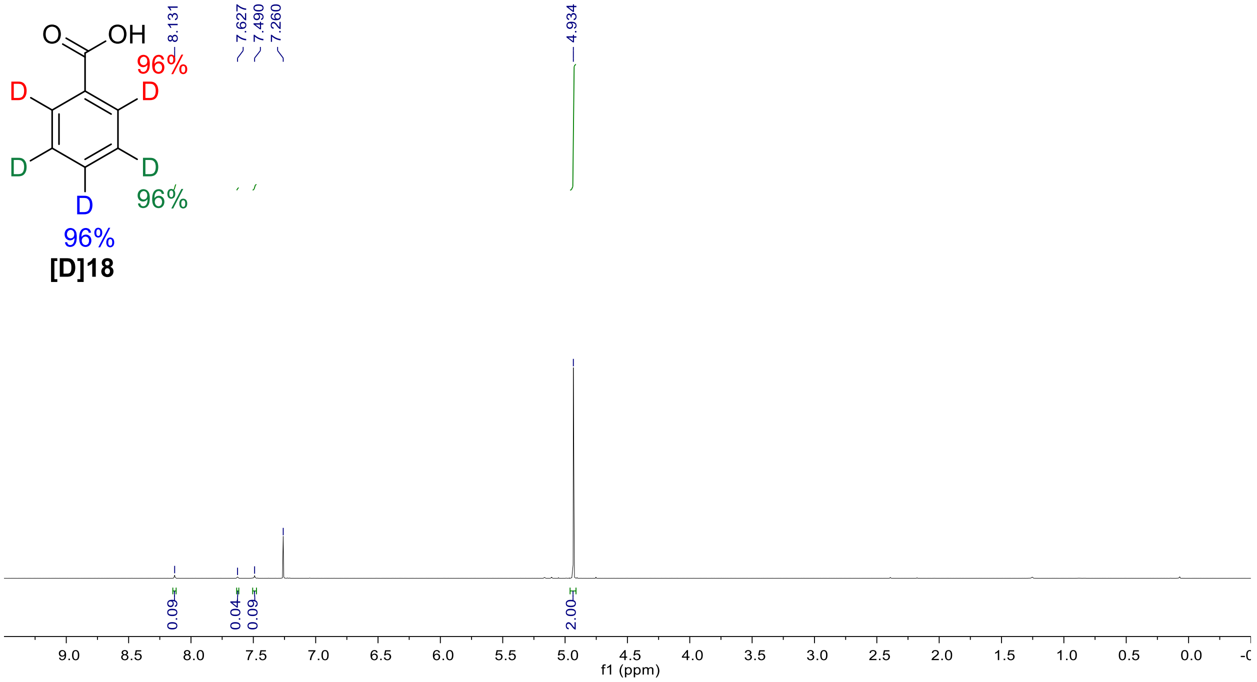

^{1}H NMR spectrum of compound **[D]18** (Procedure A, $CDCl_3$, 500 MHz)

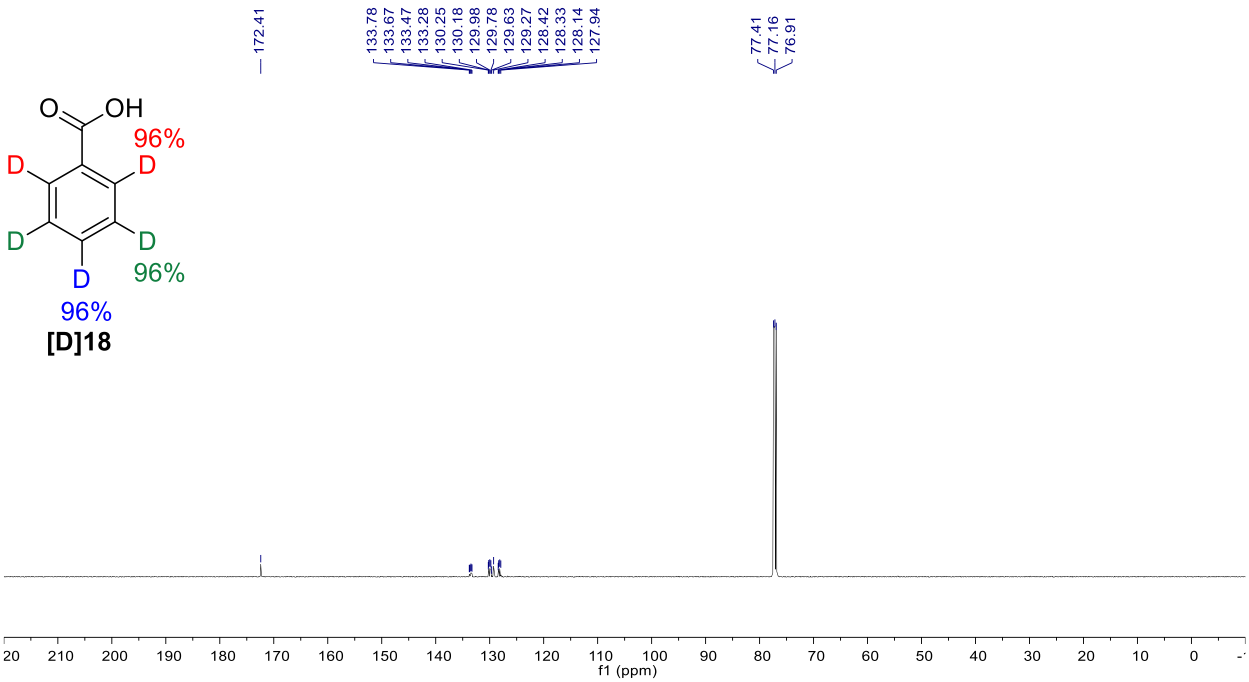

^{13}C NMR spectrum of compound **[D]18** (Procedure A, $CDCl_3$, 126 MHz)

3-fluorobenzoic acid (19)

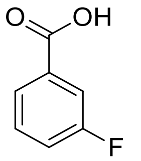

Following the general procedure A, the reaction was set up with 3-fluorobenzoic acid (28.0mg, 0.20 mmol). Purification by flash column chromatography (hexanes/EtOAc = 10:1) provided product **[D]19** as a white solid (22 mg, 76% yield)

Deuterium Incorporation

General procedure A: [LCMS (ESI)] calcd for $C_7D_4O_2F$ $[M-H]^-$ 3.78 D/molecule, [^{1}H NMR] 3.84 D/molecule.

NMR Data of the Starting Material

^{1}H NMR (500 MHz, $CDCl_3$) δ 7.92 (dt, J = 7.7, 1.3 Hz, 1H), 7.80 (ddd, J = 9.2, 2.7, 1.5 Hz, 1H), 7.47 (td, J = 8.0, 5.5 Hz, 1H), 7.33 (tdd, J = 8.3, 2.7, 1.0 Hz, 1H).

NMR Data of the Product

General procedure A: ^{1}H NMR (500 MHz, $CDCl_3$) δ 7.92 (s, 0.04H, 96% D), 7.80 (d, J = 9.2 Hz, 0.04H, 96% D), 7.47 (d, J = 5.4 Hz, 0.05H, 95% D), 7.33 (d, J = 8.4 Hz, 0.03H, 97% D); ^{13}C NMR (126 MHz, $CDCl_3$) δ 171.3, 162.6 (d, J = 247.6 Hz), 131.4, 130.1-129.6 (1C), 126.0-125.5 (1C), 121.2-120.5 (1C), 117.3-116.6 (1C).

Mass Data

LabelChecker Results

Formula: C7 H4 O2 F

Mass (monoisotopic): 139.02

Difference Value: 0.000045

Error Sum: 0.007

Error (%): 0.141

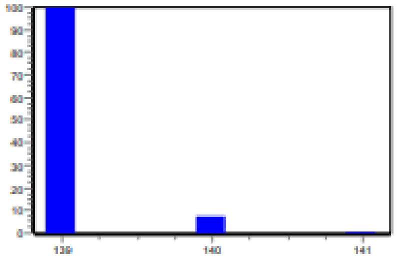

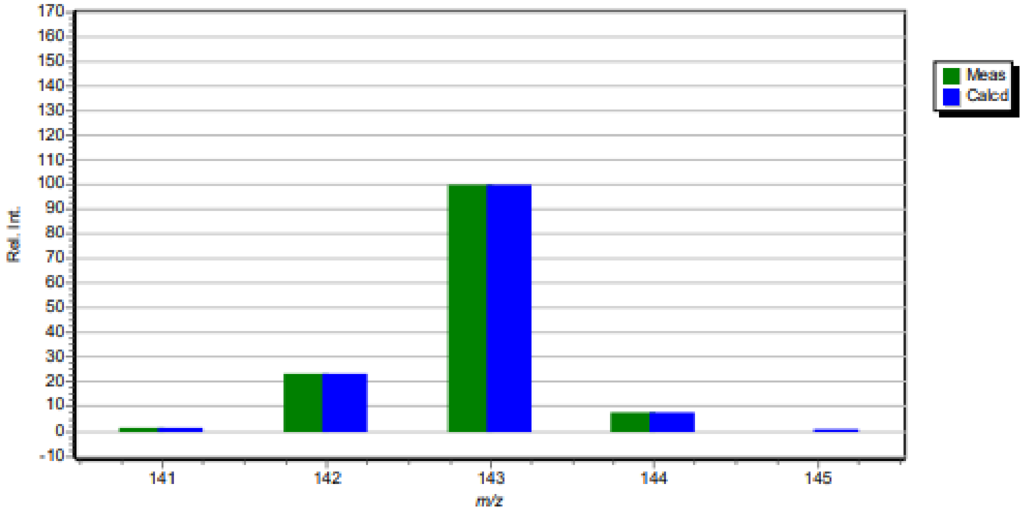

Deuterium: 0-fold (%): 0.00 0.00
Deuterium: 1-fold (%): 0.00 0.00
Deuterium: 2-fold (%): 1.86 1.48
Deuterium: 3-fold (%): 23.71 18.88
Deuterium: 4-fold (%): 100.00 79.64
Label Atom Sum: 3.78 (94.54%)

Isotope List used for fitting data:

m/z	intensity
141.03	55779
142.04	715326
143.04	3054044
144.05	230100

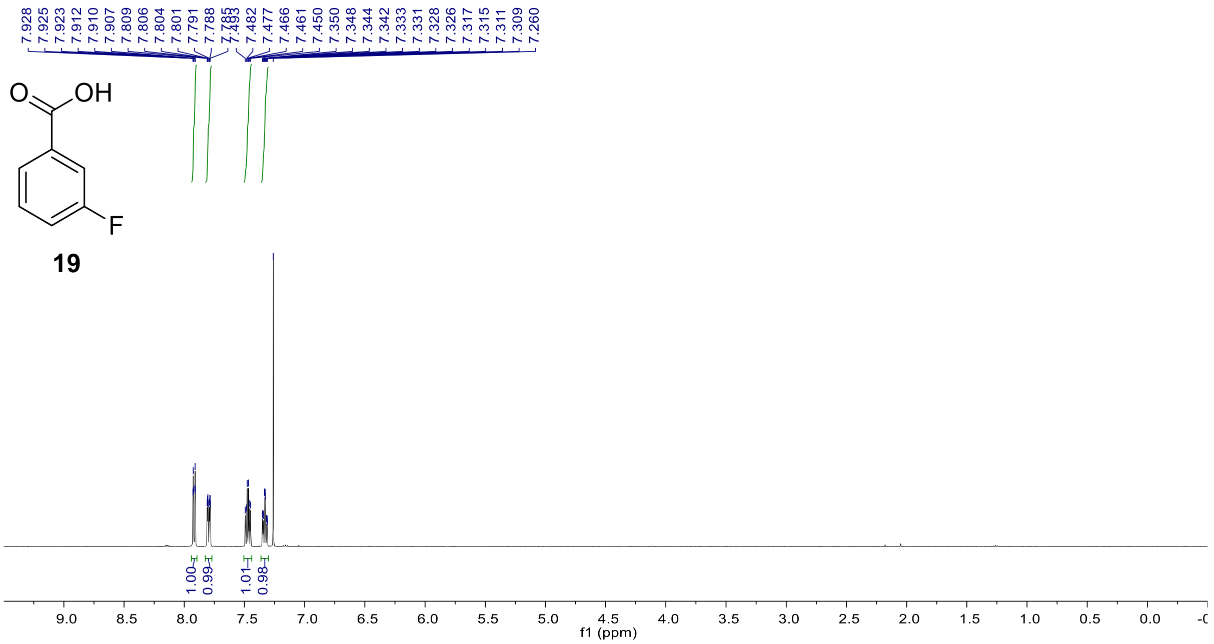

^{1}H NMR spectrum of compound **19** ($CDCl_3$, 500 MHz)

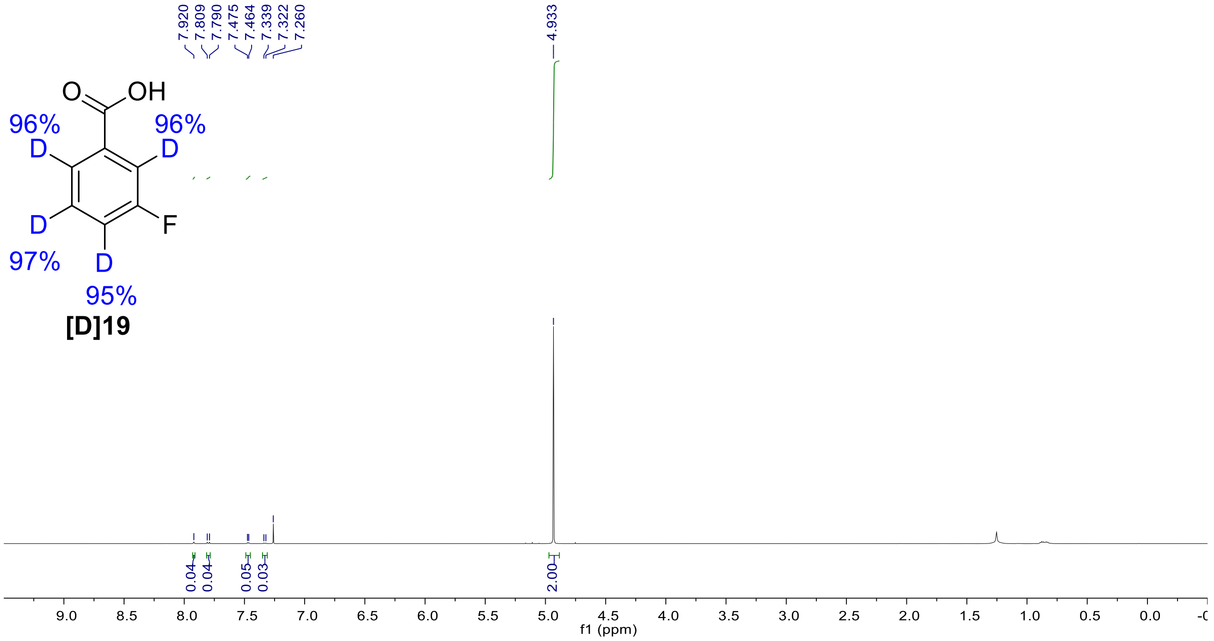

^{1}H NMR spectrum of compound **[D]19** (Procedure A, $CDCl_3$, 500 MHz)

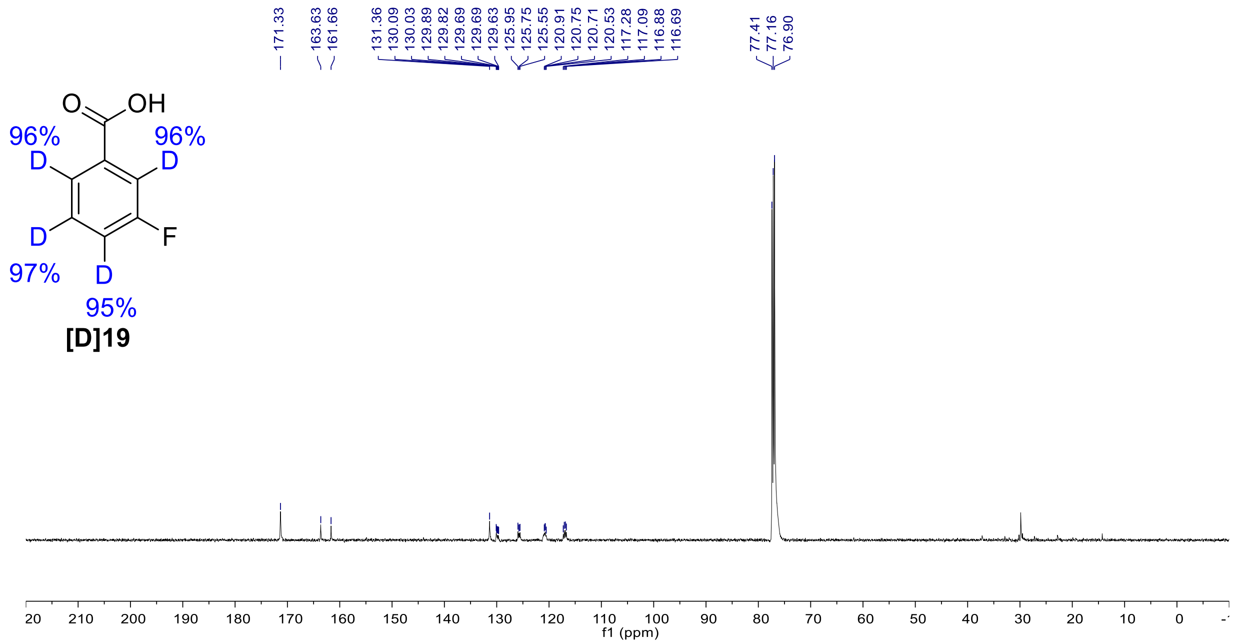

^{13}C NMR spectrum of compound **[D]19** (Procedure A, $CDCl_3$, 126 MHz)

3-chlorobenzoic acid (20)

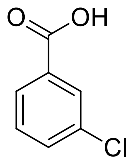

Following the general procedure A, the reaction was set up with 3-chlorobenzoic acid (31.3mg, 0.2 mmol). Purification by flash column chromatography (hexanes/EtOAc = 15:1) provided product **[D]20** as a white solid (31 mg, 97% yield)

Deuterium Incorporation

General procedure A: [LCMS (ESI)] calcd for $C_7D_4O_2Cl$ $[M-H]^-$ 4.00 D/molecule, [^1H NMR] 3.66 D/molecule.

NMR Data of the Starting Material

^1H NMR (500 MHz, $CDCl_3$) δ 8.10 (t, *J* = 1.9 Hz, 1H), 8.01 (dt, *J* = 7.8, 1.3 Hz, 1H), 7.60 (ddd, *J* = 8.0, 2.2, 1.1 Hz, 1H), 7.44 (t, *J* = 7.9 Hz, 1H).

NMR Data of the Product

General procedure A: ^1H NMR (500 MHz, $CDCl_3$) δ 8.10 (s, 0.16H, 84% D), 8.01 (s, 0.07H, 93% D), 7.60 (s, 0.06H, 94% D), 7.44 (s, 0.05H, 95% D).; ^{13}C NMR (100 MHz, $CDCl_3$) δ 171.3, 134.7, 134.0-133.5 (1C), 131.0, 130.4, 130.1-129.3 (1C), 128.4-127.9 (1C).

Mass Data

LabelChecker Results

Formula: C7 H4 O2 Cl
Mass (monoisotopic): 154.99

Difference Value: 0.007008
Error Sum: 0.084
Error (%): 0.029

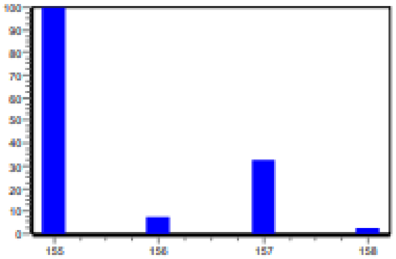

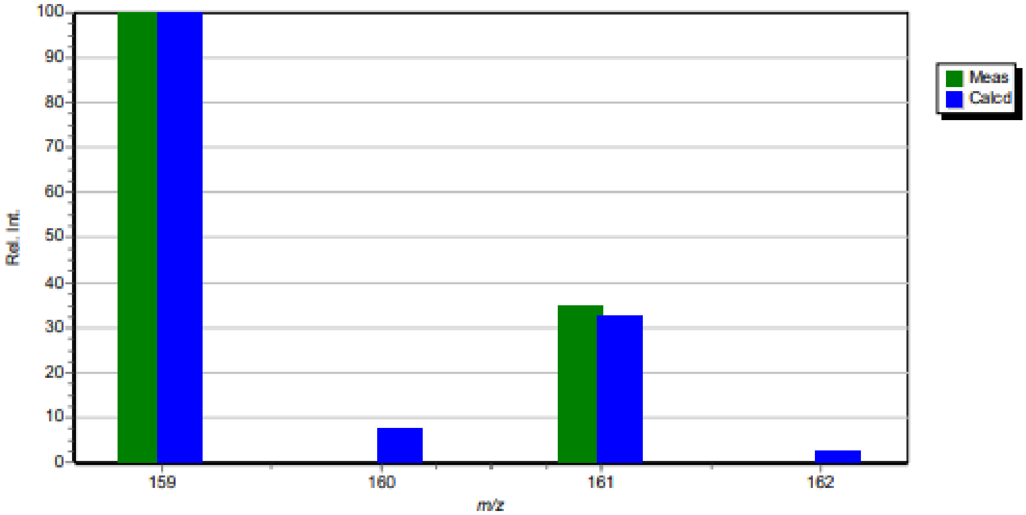

No Convergence! - Max Cycles (20) reached!
Deuterium: 0-fold (%): 0.00 0.00
Deuterium: 1-fold (%): 0.00 0.00
Deuterium: 2-fold (%): 0.00 0.00
Deuterium: 3-fold (%): 0.00 0.00
Deuterium: 4-fold (%): 100.00 100.00
Label Atom Sum: 4.00 (100.00%)

Isotope List used for fitting data:

m/z	intensity
159.01	3350861
161.01	1172446
163.02	4646

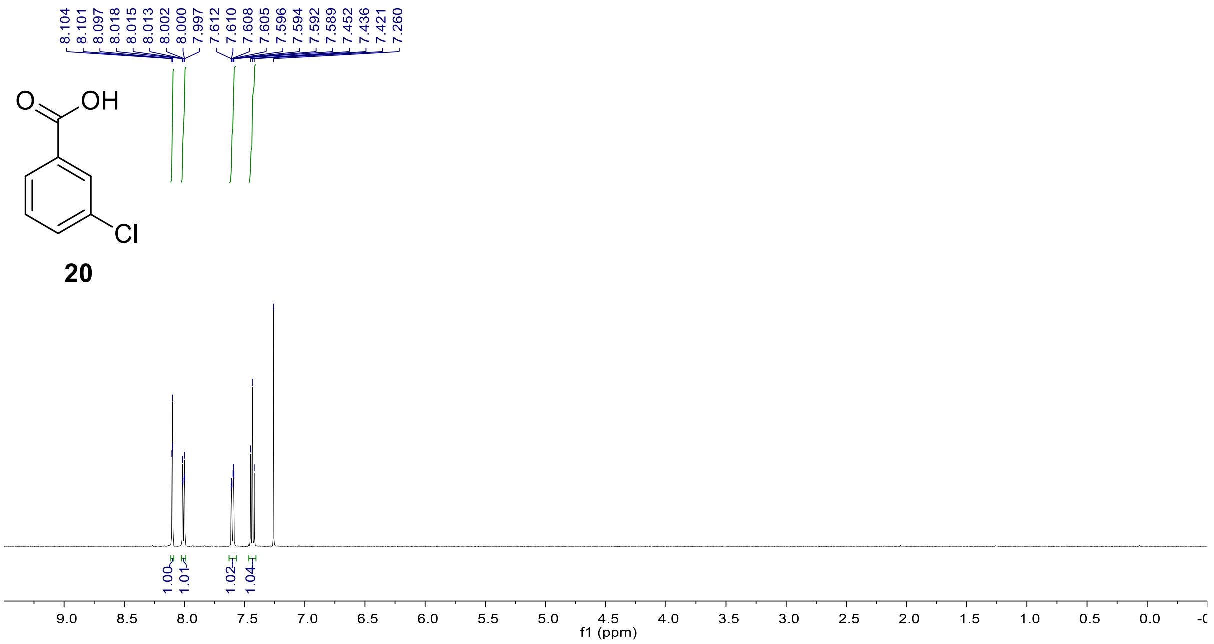

^{1}H NMR spectrum of compound **20** ($CDCl_3$, 500 MHz)

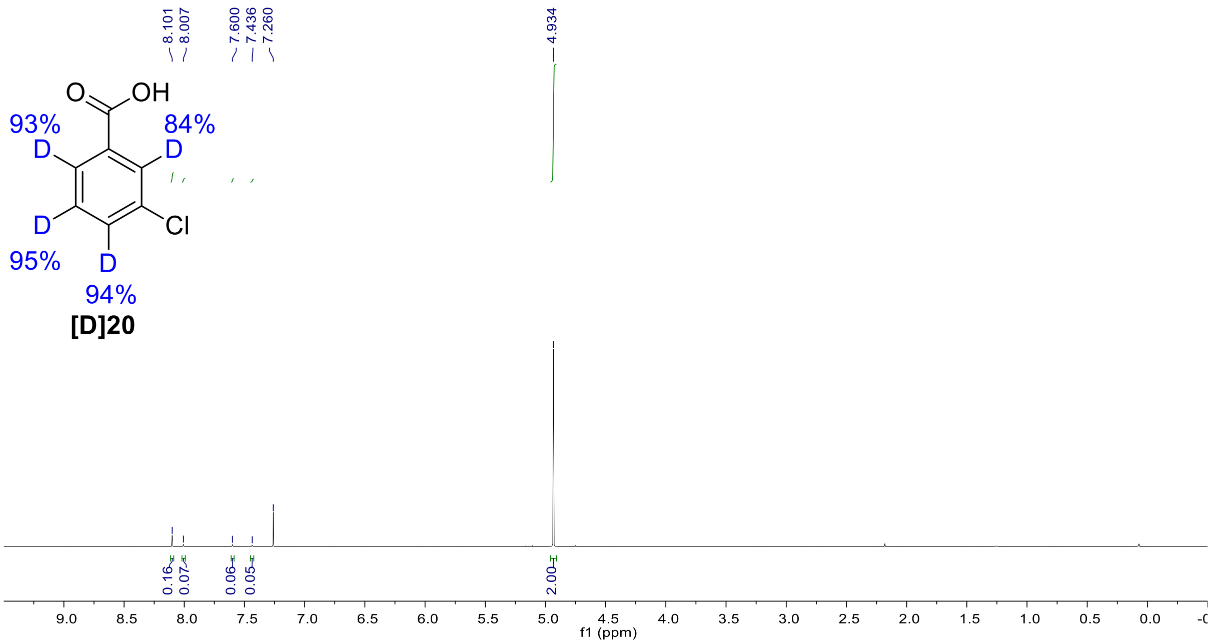

^{1}H NMR spectrum of compound **[D]20** (Procedure A, $CDCl_3$, 500 MHz)

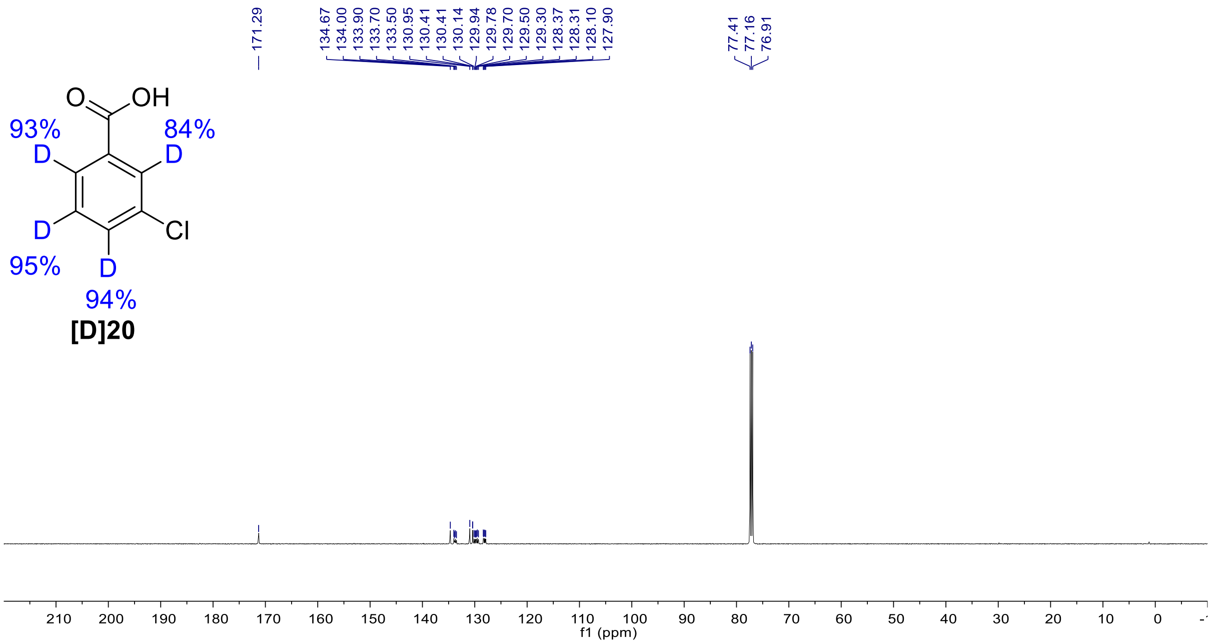

^{13}C NMR spectrum of compound **[D]20** (Procedure A, $CDCl_3$, 126 MHz)

2-chlorobenzoic acid (21)

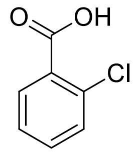

Following the general procedure A, the reaction was set up with 2-chlorobenzoic acid (31.2mg, 0.2 mmol). Purification by flash column chromatography (hexanes/EtOAc = 10:1) provided product **[D]21** as a white solid (25 mg, 78% yield)

Deuterium Incorporation

General procedure A: [LCMS (ESI)] calcd for $C_7D_4O_2Cl$ $[M-H]^-$ 4.00 D/molecule, [^{1}H NMR] 3.86 D/molecule.

NMR Data of the Starting Material

^{1}H NMR (500 MHz, $CDCl_3$) δ 8.03 (dd, J = 7.4, 1.4 Hz, 1H), 7.53-7.47 (m, 2H), 7.40-7.34 (m, 1H).

NMR Data of the Product

General procedure A: ^{1}H NMR (500 MHz, $CDCl_3$) δ 8.03 (s, 0.05H, 95% D), 7.50 (d, J = 8.5 Hz, 0.07H, 97%), 7.37 (s, 0.03H, 97% D); ^{13}C NMR (126 MHz, $CDCl_3$) δ 171.1, 134.9, 133.6-133.1 (1C), 132.5-132.1 (1C), 131.6-131.1 (1C), 128.4, 126.6-126.2 (1C).

Mass Data

LabelChecker Results

Formula: C7 H4 O2 Cl

Mass (monoisotopic): 154.99

Difference Value: 0.000032

Error Sum: 0.006

Error (%): 0.140

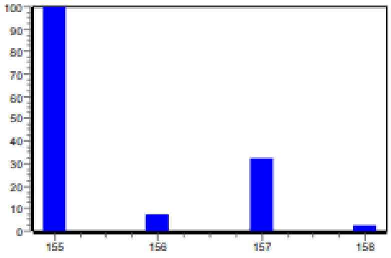

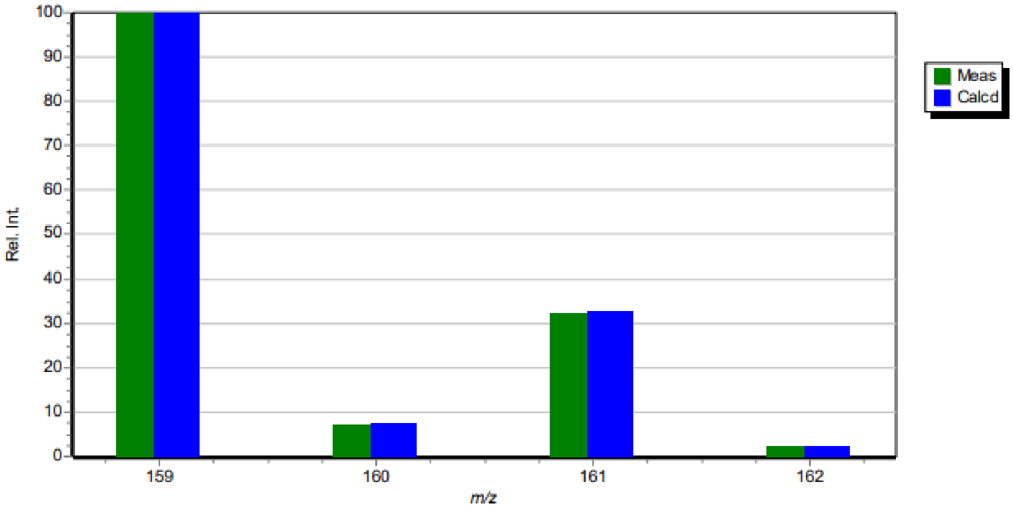

Deuterium: 0-fold (%): 0.00 0.00
Deuterium: 1-fold (%): 0.00 0.00
Deuterium: 2-fold (%): 0.06 0.06
Deuterium: 3-fold (%): 0.00 0.00
Deuterium: 4-fold (%): 100.00 99.94
Label Atom Sum: 4.00 (99.97%)

Isotope List used for fitting data:

m/z	intensity
159.01	20377916
160.02	1474919
161.01	6579979
162.01	486597
163.02	28910

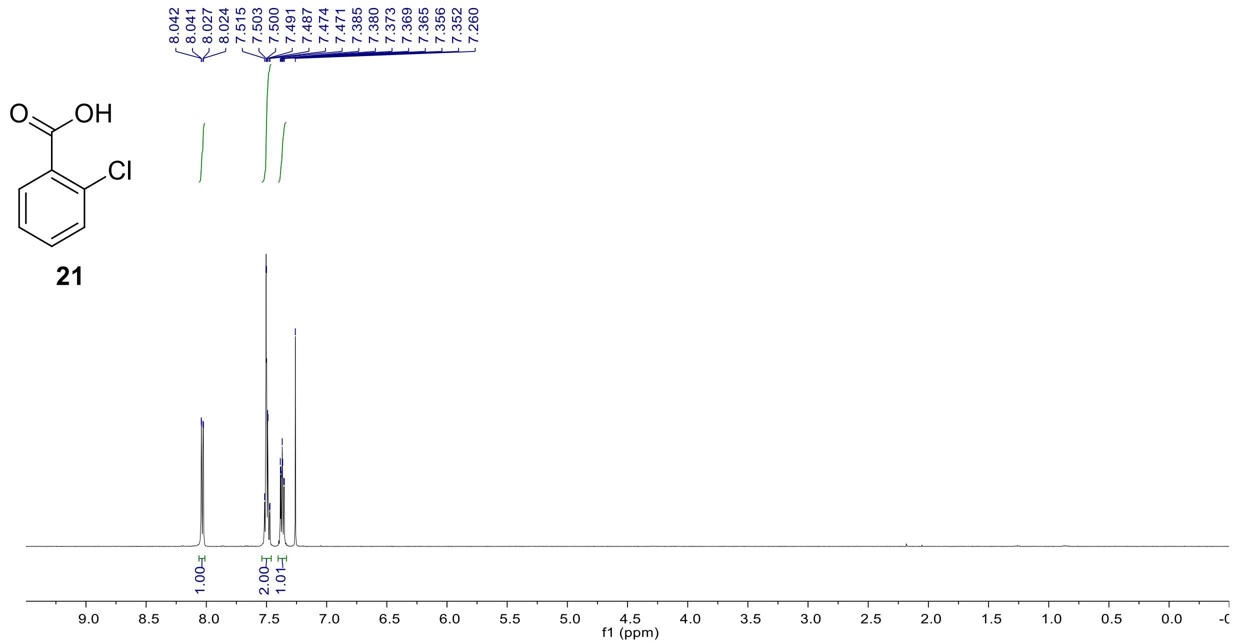

^{1}H NMR spectrum of compound **21** ($CDCl_3$, 500 MHz)

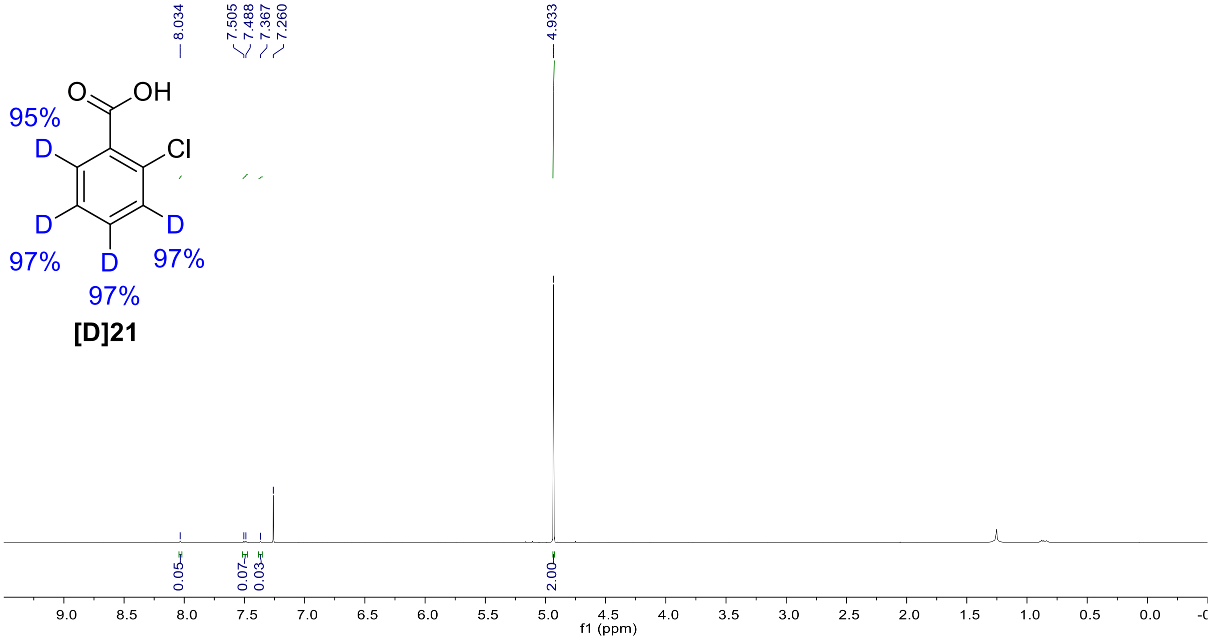

^{1}H NMR spectrum of compound **[D]21** (Procedure A, $CDCl_3$, 500 MHz)

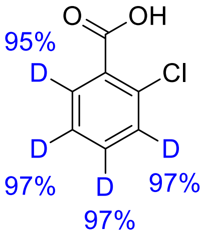

[D]21

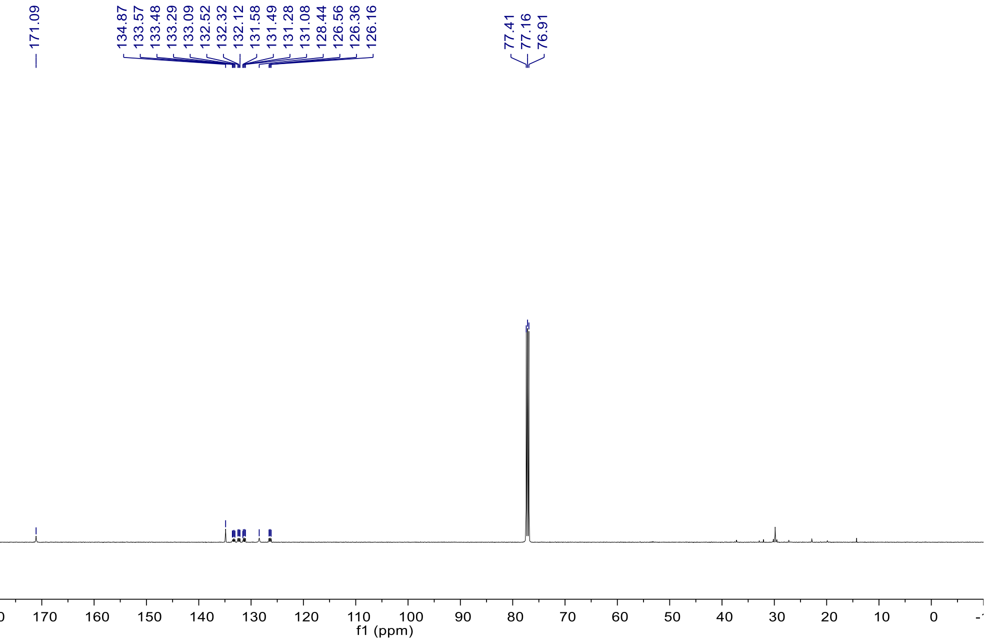

^{13}C NMR spectrum of compound **[D]21** (Procedure A, $CDCl_3$, 126 MHz)

3-bromobenzoic acid (22)

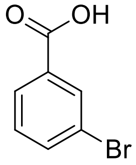

Following the general procedure A, the reaction was set up with 3-bromobenzoic acid (40.0 mg, 0.20 mmol). Purification by flash column chromatography (hexanes/EtOAc/AcOH = 20:1:0.15) provided product **[D]22** as a white solid (32 mg, 78% yield).

Deuterium Incorporation

General procedure A: [LCMS (ESI)] calcd for $C_7D_4O_2Br$ $[M-H]^-$ 3.66 D/molecule, [^{1}H NMR] 3.74 D/molecule.

NMR Data of the Starting Material

^{1}H NMR (500 MHz, $CDCl_3$) δ 8.25 (t, *J* = 1.8 Hz, 1H), 8.04 (dt, *J* = 7.8, 1.3 Hz, 1H), 7.75 (ddd, *J* = 8.0, 2.0, 1.0 Hz, 1H), 7.37 (t, *J* = 7.9 Hz, 1H).

NMR Data of the Product

General procedure A: ^{1}H NMR (500 MHz, $CDCl_3$) δ 8.26 (s, 0.12H, 88% D), 8.05 (s, 0.06H, 94% D), 7.75 (s, 0.04H, 96% D) 7.37 (s, 0.04H, 96% D); ^{13}C NMR (126 MHz, $CDCl_3$) δ 171.2, 136.9-136.4 (1C), 133.3-132.9 (1C), 131.1, 130.0-129.5 (1C), 128.8-128.4 (1C), 122.6-122.5 (1C).

Mass Data

LabelChecker Results

Formula:	C7 H4 O2 Br
Mass (monoisotopic):	198.94
Difference Value:	0.001867
Error Sum:	0.043
Error (%):	0.735

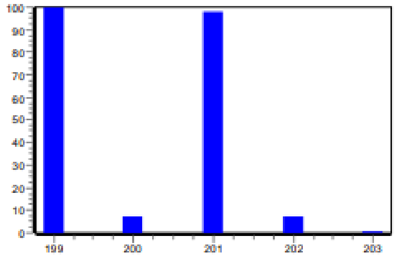

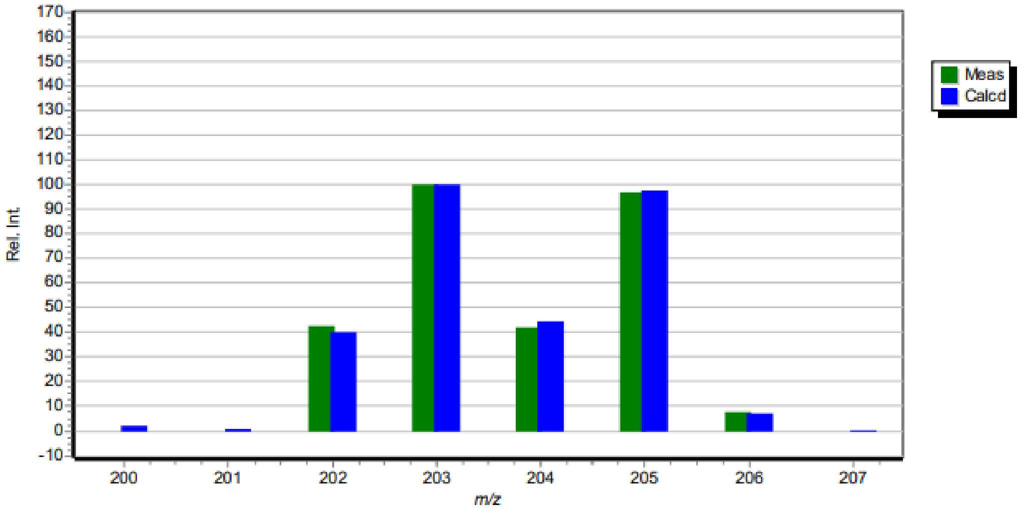

Deuterium: 0-fold (%): 0.00 0.00
Deuterium: 1-fold (%): 2.47 1.73
Deuterium: 2-fold (%): 0.49 0.34
Deuterium: 3-fold (%): 39.36 27.66
Deuterium: 4-fold (%): 100.00 70.27
Label Atom Sum: 3.66 (91.61%)

Isotope List used for fitting data:

m/z	intensity
201.96	5384558
202.96	12589341
203.96	5299922
204.96	12188366
205.97	956693
206.97	50604

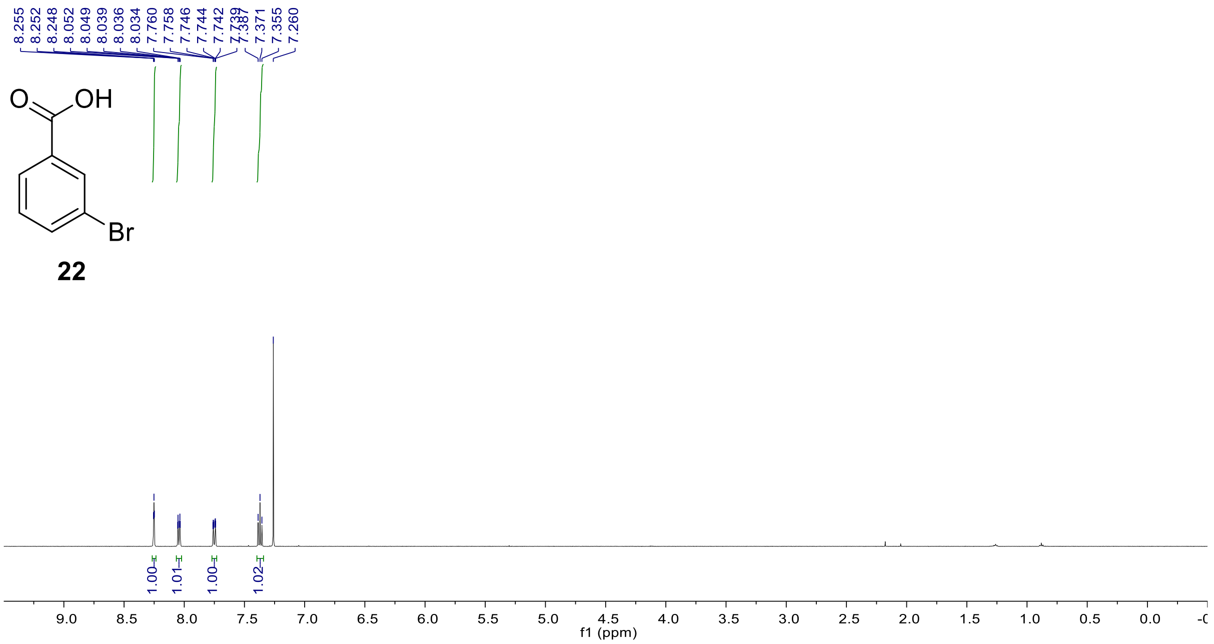

^{1}H NMR spectrum of compound **22** ($CDCl_3$, 500 MHz)

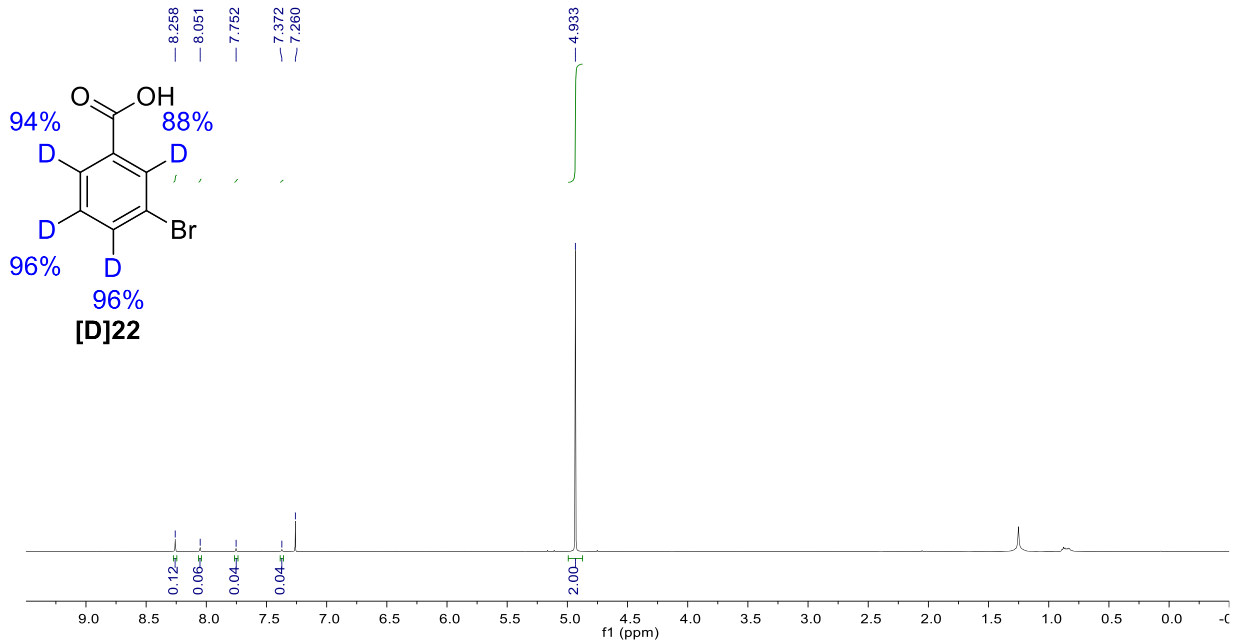

^{1}H NMR spectrum of compound **[D]22** (Procedure A, $CDCl_3$, 500 MHz)

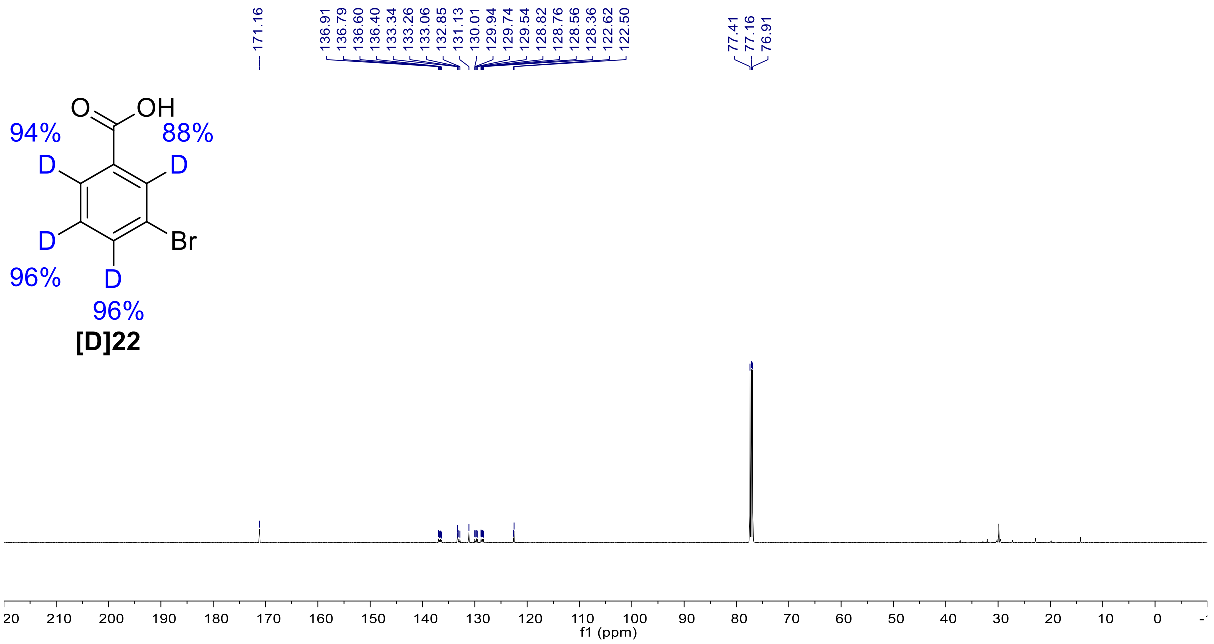

^{13}C NMR spectrum of compound **[D]22** (Procedure A, $CDCl_3$, 126 MHz)

4-bromobenzoic acid (23)

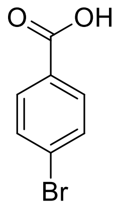

Following the general procedure A, the reaction was set up with 4-bromobenzoic acid (40.0 mg, 0.20 mmol). Purification by flash column chromatography (hexanes/EtOAc = 10:1) provided product **[D]23** as a white solid (36 mg, 88% yield)

Deuterium Incorporation

General procedure A: [LCMS (ESI)] calcd for $C_7D_4O_2Br$ $[M\text{-}H]^-$ 4.00 D/molecule, [^{1}H NMR] 3.84 D/molecule.

NMR Data of the Starting Material

^{1}H NMR (500 MHz, DMSO-d_6) δ 7.86 (d, J = 8.5 Hz, 2H), 7.71 (d, J = 8.5 Hz, 2H).

NMR Data of the Product

General procedure A: ^{1}H NMR (500 MHz, DMSO-d_6) δ 7.86 (s, 0.13H, 94% D), 7.71 (s, 0.05H, 98% D); ^{13}C NMR (126 MHz, DMSO-d_6) δ 166.7, 131.6-130.7 (4C), 129.9, 126.7.

Mass Data

LabelChecker Results

Formula: C7 H4 O2 Br
Mass (monoisotopic): 198.94

Difference Value: 0.011830
Error Sum: 0.109
Error (%): 0.018

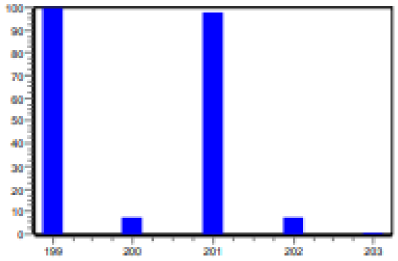

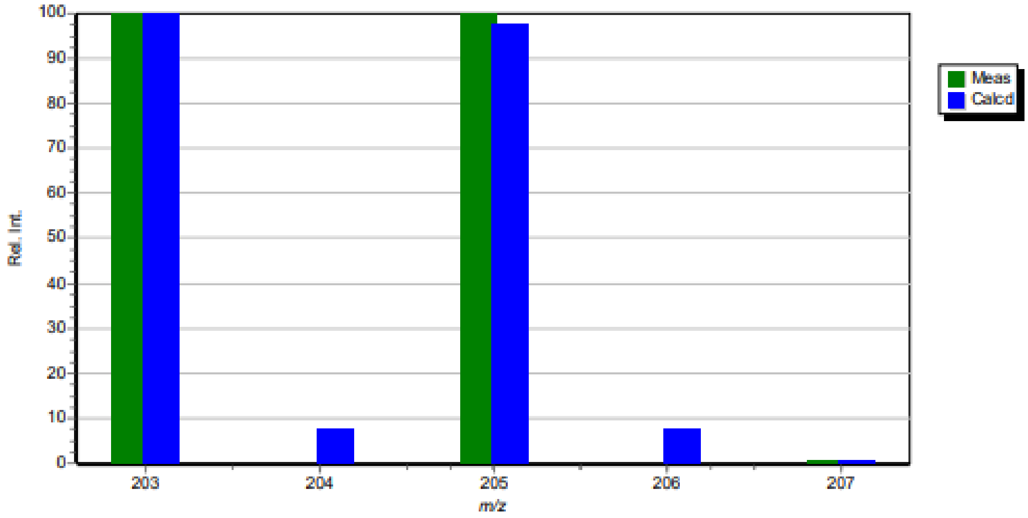

No Convergence! - Max Cycles (20) reached!
Deuterium: 0-fold (%): 0.00 0.00
Deuterium: 1-fold (%): 0.00 0.00
Deuterium: 2-fold (%): 0.00 0.00
Deuterium: 3-fold (%): 0.00 0.00
Deuterium: 4-fold (%): 100.00 100.00
Label Atom Sum: 4.00 (100.00%)

Isotope List used for fitting data:

m/z	intensity
202.96	2575967
204.96	2574030
206.97	13813

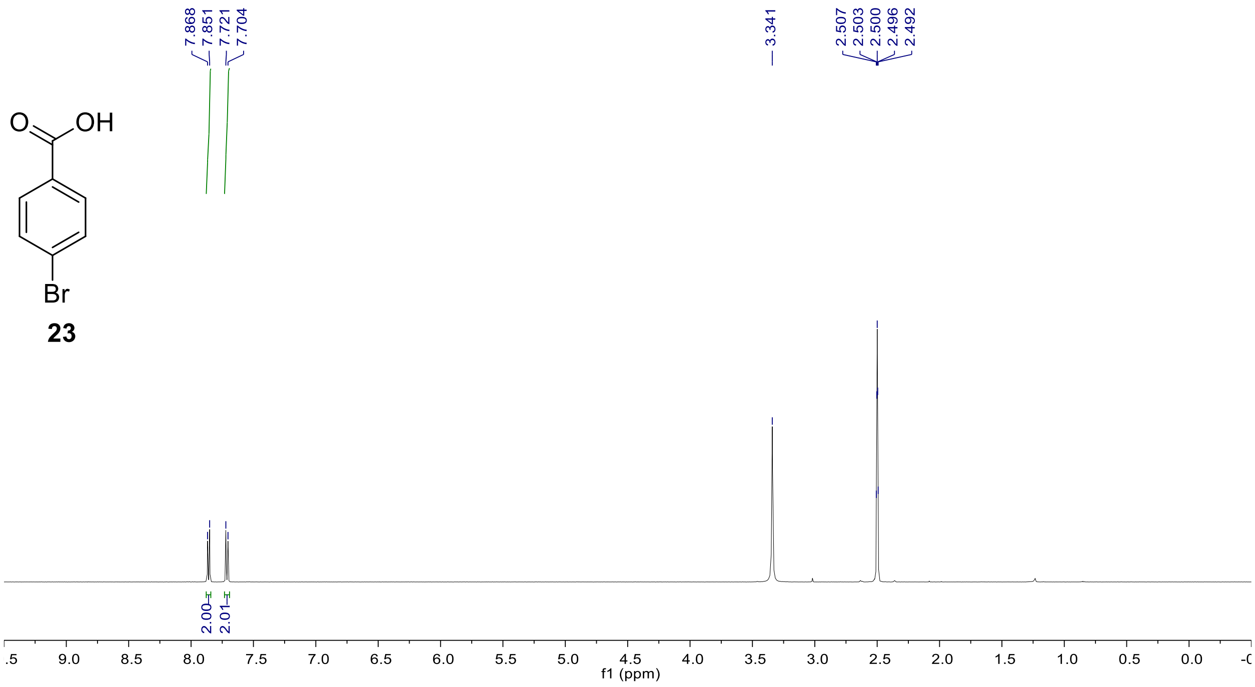

^{1}H NMR spectrum of compound **23** (DMSO-d_6, 500 MHz)

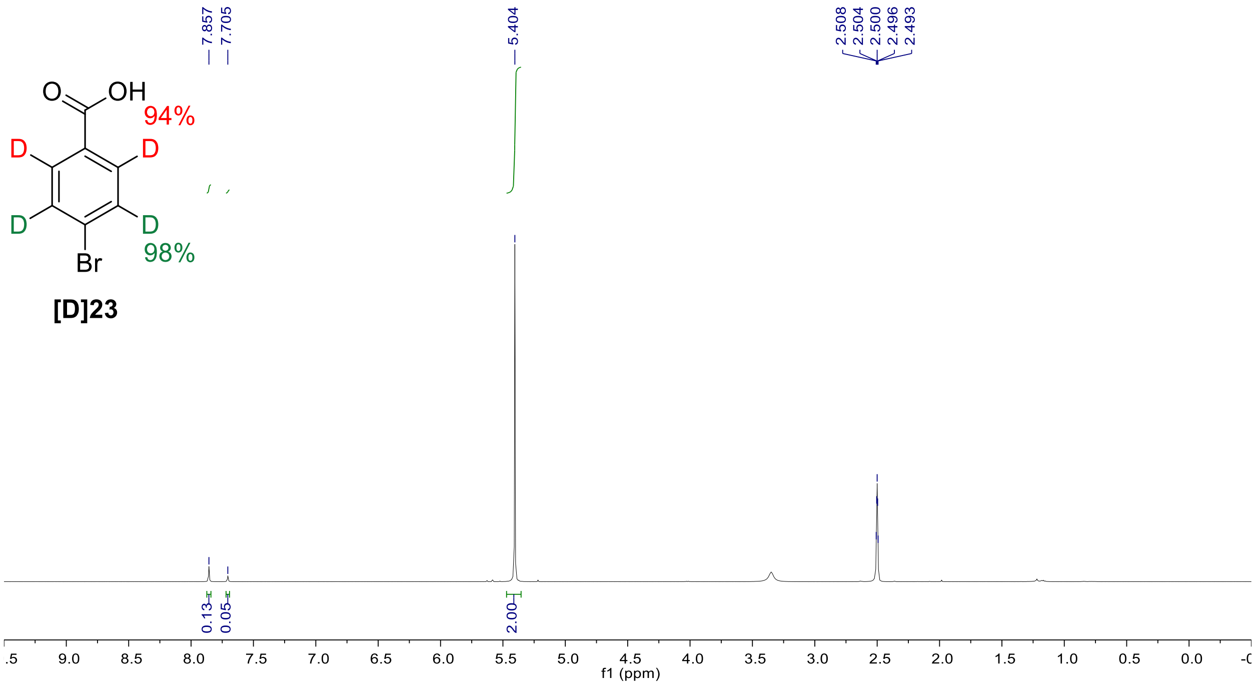

^{1}H NMR spectrum of compound **[D]23** (Procedure A, DMSO-d_6, 500 MHz)

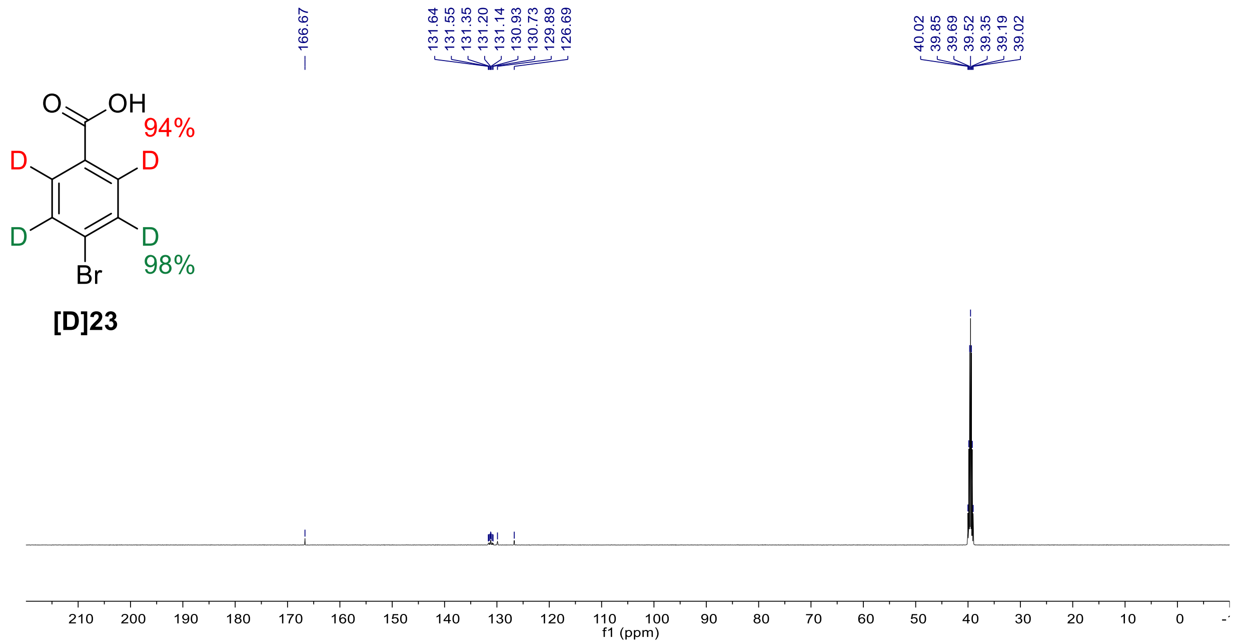

^{13}C NMR spectrum of compound **[D]23** (Procedure A, DMSO-d_6, 126 MHz)

3.4-dichlorobenzoic acid (24)

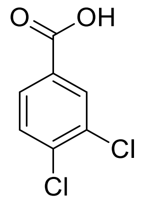

Following the general procedure A, the reaction was set up with 3.4-dichlorobenzoic acid (38.2 mg, 0.20 mmol). Purification by flash column chromatography (hexanes/EtOAc= 10:1) provided product **[D]24** as a white solid (37 mg, 95% yield).

Deuterium Incorporation

General procedure A: [LCMS (ESI)] calcd for $C_7D_3O_2Cl_2$ $[M-H]^-$ 3.00 D/molecule, [^{1}H NMR] 2.83 D/molecule.

NMR Data of the Starting Material

^{1}H NMR (500 MHz, $CDCl_3$) δ 8.19 (d, *J* = 2.0 Hz, 1H), 7.93 (dd, *J* = 8.4, 2.0 Hz, 1H), 7.57 (d, *J* = 8.3 Hz, 1H).

NMR Data of the Product

General procedure A: ^{1}H NMR (500 MHz, $CDCl_3$) δ 8.19 (s, 0.11H, 89% D), 7.93 (s, 0.04H, 96% D), 7.57 (s, 0.02H, 98% D); ^{13}C NMR (126 MHz, DMSO-*d*$_6$) δ 165.5 (1C), 135.7 (1C), 131.53-131.45 (1C), 131.0-130.9 (1C), 130.7-130.5 (1C), 129.22-129.17 (1C), 129.0-128.8 (1C).

Mass Data

LabelChecker Results

Formula: C7 H3 O2 Cl2
Mass (monoisotopic): 188.95

Difference Value: 0.008458
Error Sum: 0.092
Error (%): 0.001

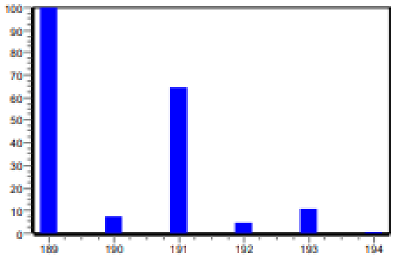

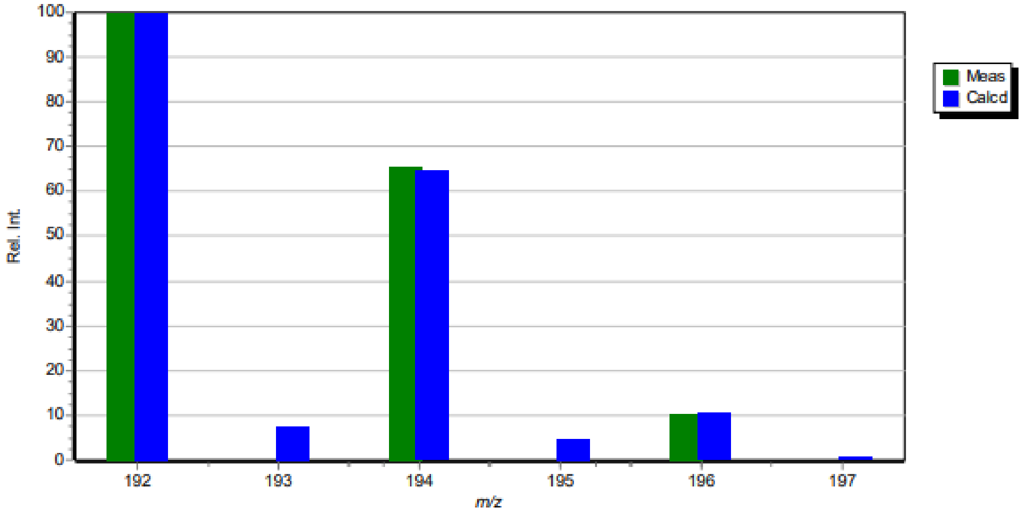

No Convergence! - Max Cycles (20) reached!
Deuterium: 0-fold (%): 0.00 0.00
Deuterium: 1-fold (%): 0.00 0.00
Deuterium: 2-fold (%): 0.00 0.00
Deuterium: 3-fold (%): 100.00 100.00
Label Atom Sum: 3.00 (100.00%)

Isotope List used for fitting data:

m/z intensity
191.97 23067438
193.97 15144362
195.96 2371001

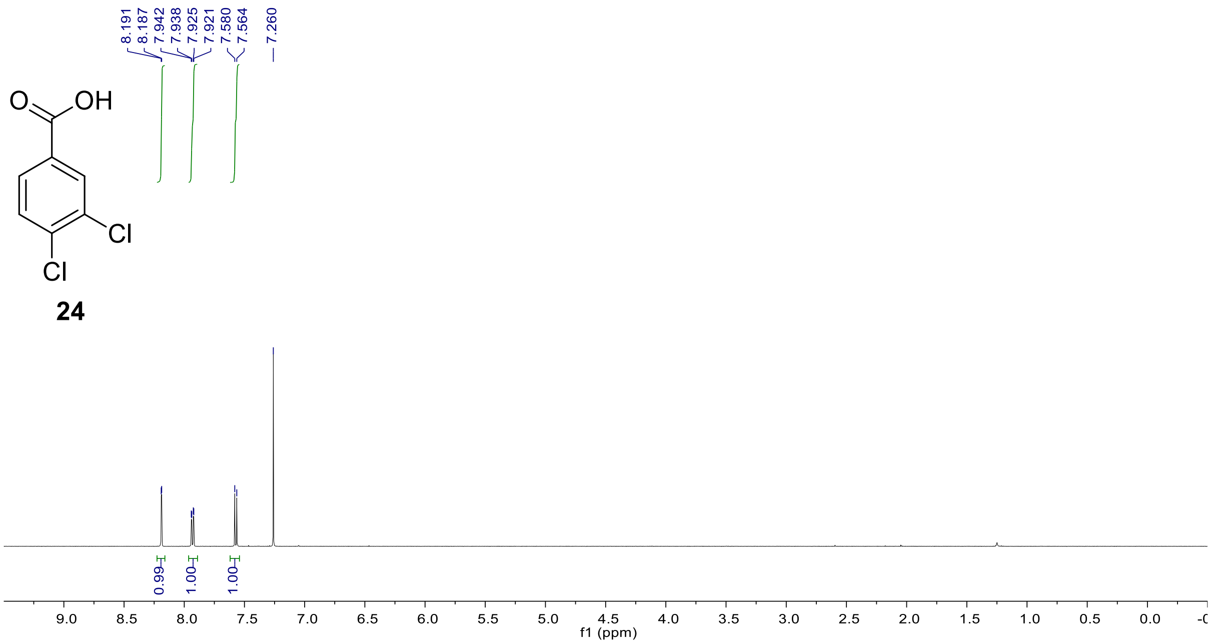

^{1}H NMR spectrum of compound **24** ($CDCl_3$, 500 MHz)

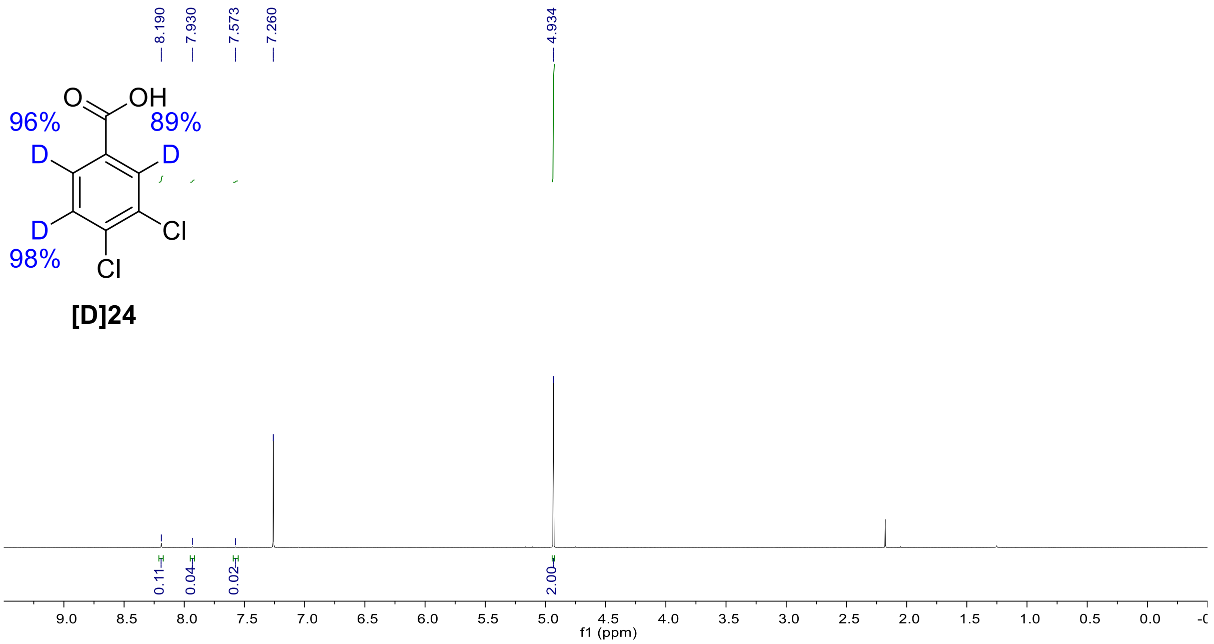

^{1}H NMR spectrum of compound **[D]24** (Procedure A, $CDCl_3$, 500 MHz)

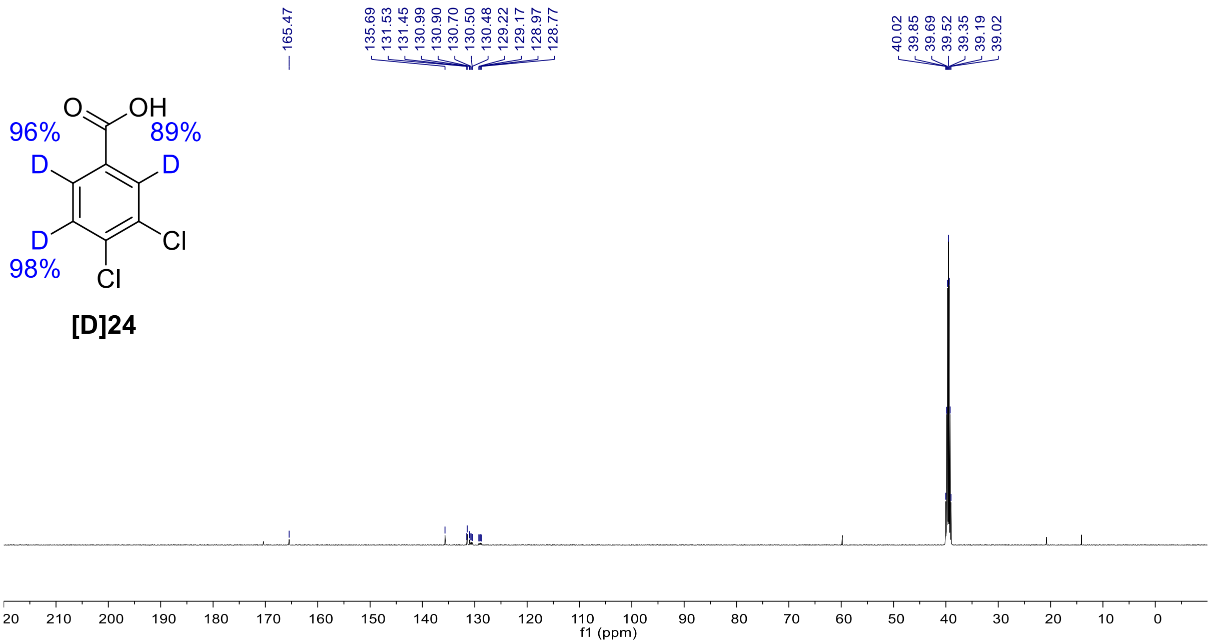

^{13}C NMR spectrum of compound **[D]24** (Procedure A, DMSO-d_6, 126 MHz)

3-methoxybenzoic acid (25)

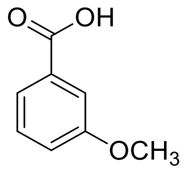

Following the general procedure A, the reaction was set up with 3-methoxybenzoic acid (30.4 mg, 0.20 mmol). Purification by flash column chromatography (hexanes/EtOAc= 5:1) provided product **[D]25** as a white solid (24 mg, 77% yield).

Deuterium Incorporation

General procedure A: [LCMS (ESI)] calcd for $C_8H_3D_4O_3$ $[M-H]^-$ 3.63 D/molecule, [^1H NMR] 3.72 D/molecule.

NMR Data of the Starting Material

^1H NMR (500 MHz, $CDCl_3$) δ 7.72 (dt, *J* = 7.6, 1.2 Hz, 1H), 7.62 (dd, *J* = 2.6, 1.5 Hz, 1H), 7.39 (t, *J* = 7.9 Hz, 1H), 7.16 (ddd, *J* = 8.3, 2.7, 1.0 Hz, 1H), 3.87 (s, 3H).

NMR Data of the Product

General procedure A: ^1H NMR (500 MHz, $CDCl_3$) δ 7.72 (s, 0.05H, 95% D), 7.63 (s, 0.06H, 94% D), 7.39 (s, 0.12H, 88% D), 7.17 (s, 0.05H, 95% D), 3.87 (s, 2.63H, 12% D); ^{13}C NMR (126 MHz, $CDCl_3$) δ 172.3, 159.7, 130.8, 129.6-128.9 (1C), 122.8-122.2 (1C), 120.6-120.0 (1C), 114.6-114.0 (1C), 55.6.

Mass Data

LabelChecker Results

Formula: C8 H7 O3
Mass (monoisotopic): 151.04

Difference Value: 0.000055
Error Sum: 0.007
Error (%): 0.041

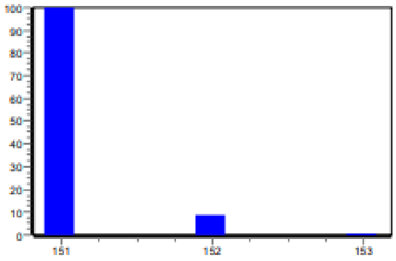

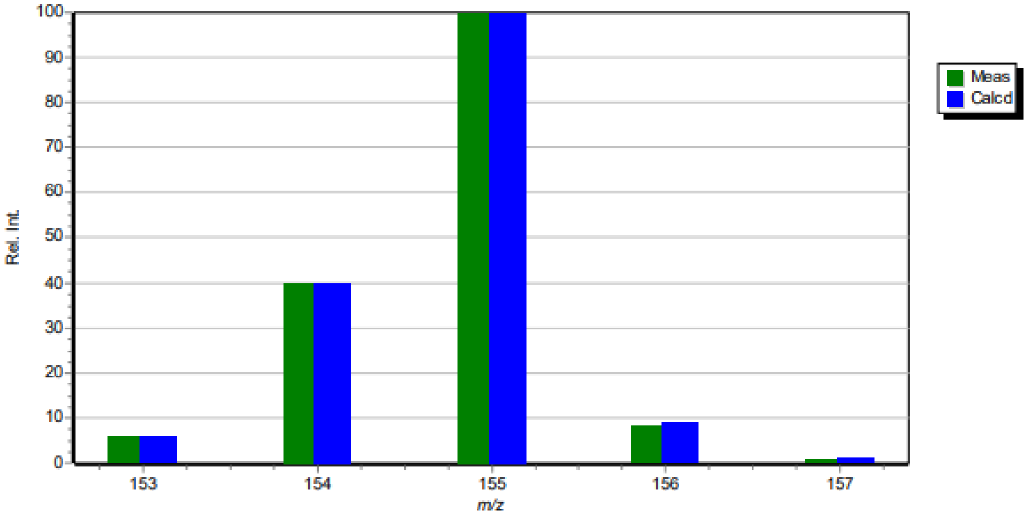

Deuterium: 0-fold (%): 0.04 0.03
Deuterium: 1-fold (%): 0.27 0.18
Deuterium: 2-fold (%): 6.10 4.15
Deuterium: 3-fold (%): 40.83 27.73
Deuterium: 4-fold (%): 100.00 67.91
Deuterium: 5-fold (%): 0.00 0.00
Deuterium: 6-fold (%): 0.01 0.01
Deuterium: 7-fold (%): 0.00 0.00
Label Atom Sum: 3.63 (51.91%)

Isotope List used for fitting data:

m/z	intensity
152.05	25027
153.05	564030
154.06	3808778
155.06	9543955
156.07	786102
157.07	55175

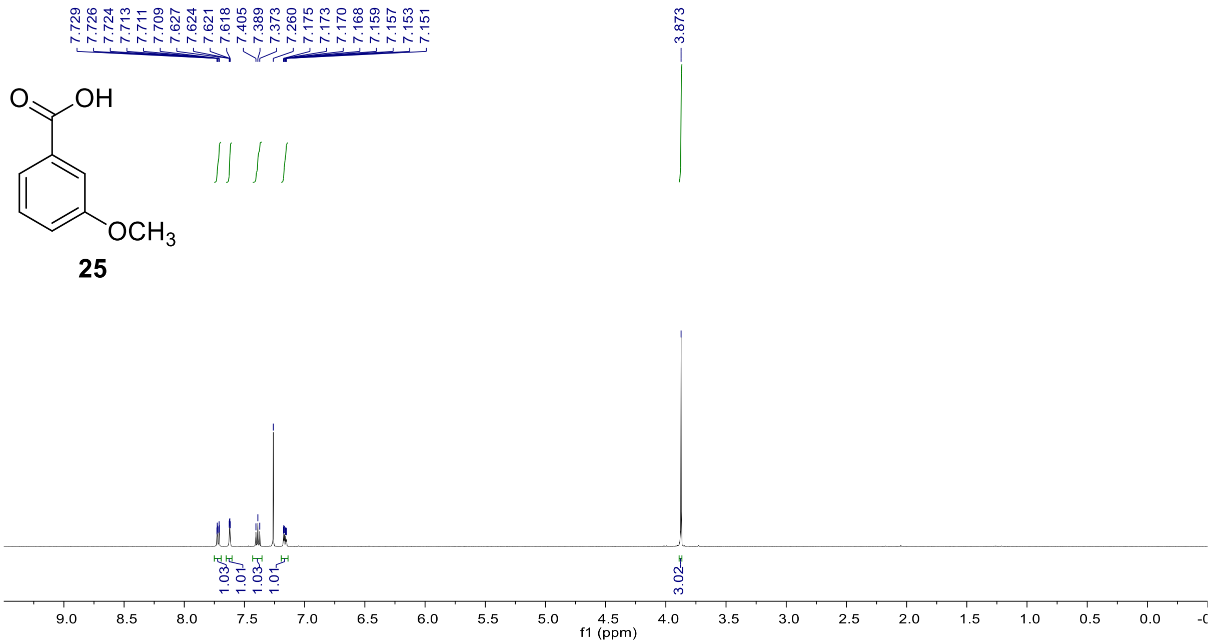

^{1}H NMR spectrum of compound **25** (CDCl$_3$, 500 MHz)

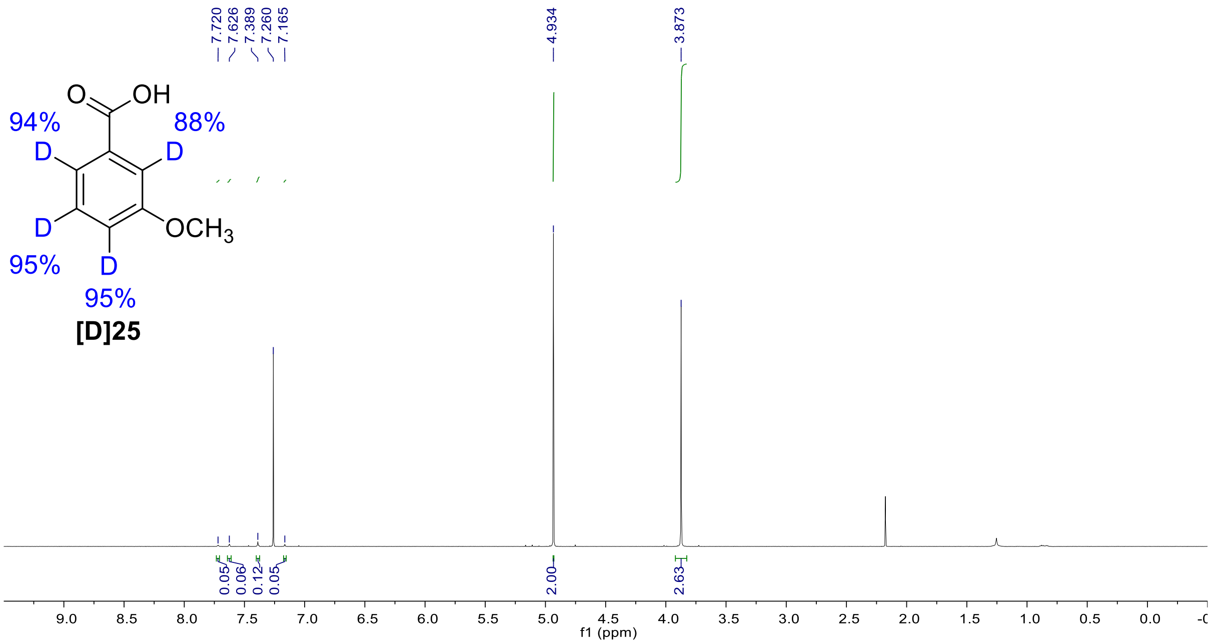

^{1}H NMR spectrum of compound **[D]25** (Procedure A, CDCl$_3$, 500 MHz)

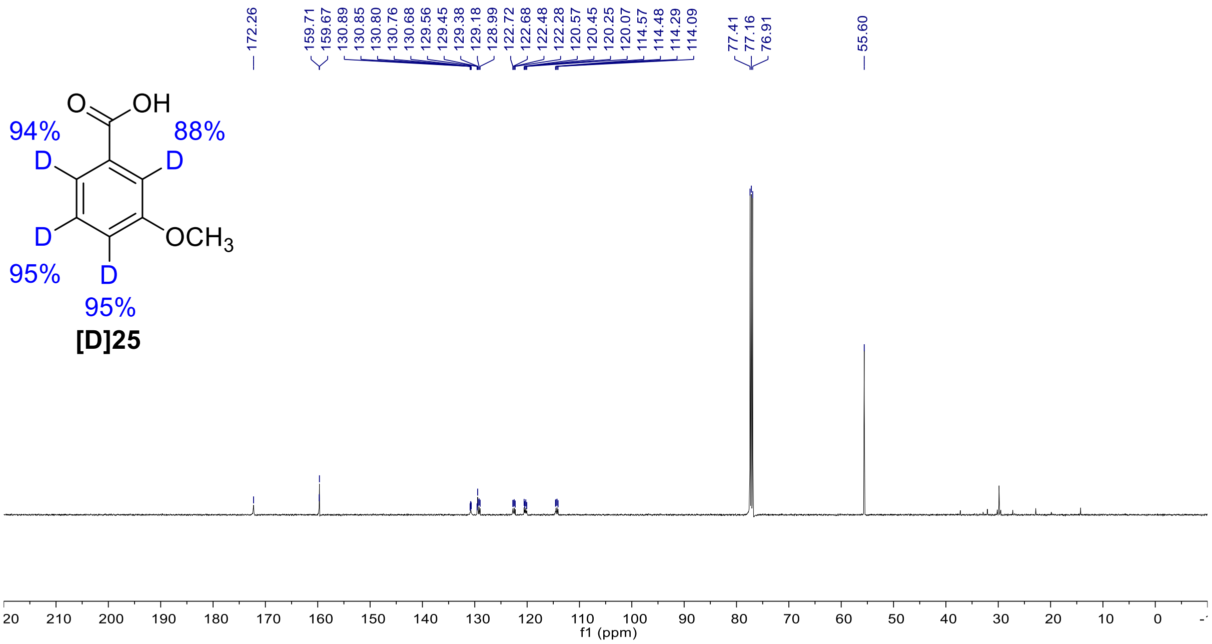

^{13}C NMR spectrum of compound **[D]25** (Procedure A, $CDCl_3$, 126 MHz)

cinnamic acid (26)

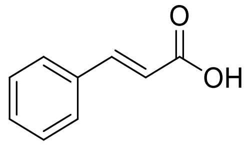

Following the general procedure A, the reaction was set up with cinnamic acid (29.6 mg, 0.20 mmol). Purification by flash column chromatography (hexanes/EtOAc= 10:1) provided product **[D]26** as a yellow solid (27 mg, 88% yield).

Deuterium Incorporation

General procedure A: [LCMS (ESI)] calcd for $C_9HD_6O_2$ $[M\text{-}H]^-$ 5.60 D/molecule, [^{1}H NMR] 6.03 D/molecule.

NMR Data of the Starting Material

^{1}H NMR (500 MHz, $CDCl_3$) δ 7.80 (d, J = 16.0 Hz, 1H), 7.60 -7.53 (m, 2H), 7.45-7.38 (m, 3H), 6.47 (d, J = 16.0 Hz, 1H).

NMR Data of the Product

General procedure A: ^{1}H NMR (500 MHz, $CDCl_3$) δ 7.84-7.77 (m, 0.58H, 42% D), 7.59-7.51 (m, 0.18H, 91% D), 7.41 (s, 0.16H, 95% D), 6.47 (d, J = 16.0 Hz, 0.06H, 94% D); ^{13}C NMR (126 MHz, $CDCl_3$) δ 172.6, 147.1, 134.0, 130.8-130.2 (2C), 129.0-128.6 (2C), 128.4-127.9 (1C), 117.5-117.0 (1C).

Mass Data

LabelChecker Results

Formula: C9 H7 O2
Mass (monoisotopic): 147.04

Difference Value: 0.000375
Error Sum: 0.019
Error (%): 0.173

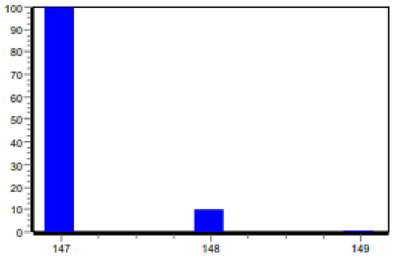

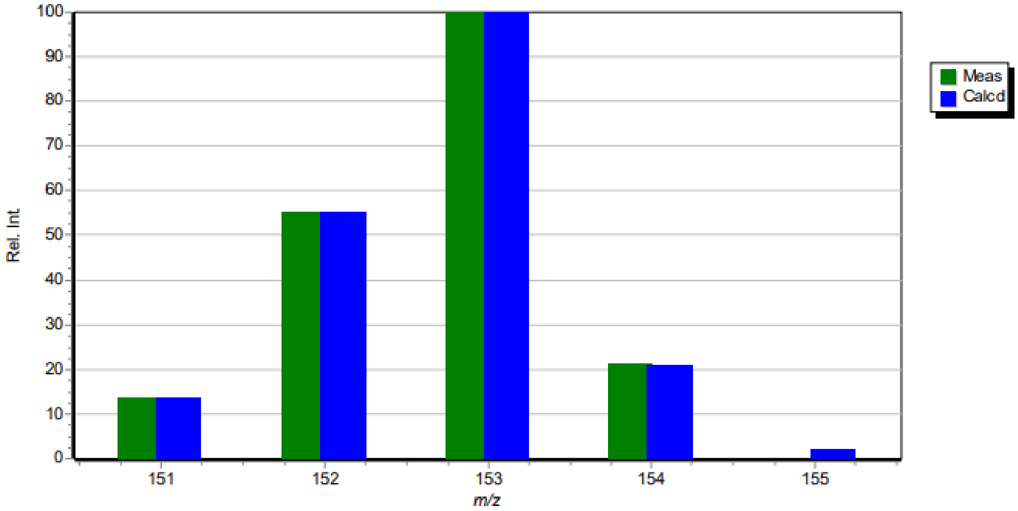

Deuterium: 0-fold (%): 0.00 0.00
Deuterium: 1-fold (%): 0.00 0.00
Deuterium: 2-fold (%): 0.00 0.00
Deuterium: 3-fold (%): 0.00 0.00
Deuterium: 4-fold (%): 14.39 7.86
Deuterium: 5-fold (%): 56.91 31.07
Deuterium: 6-fold (%): 100.00 54.60
Deuterium: 7-fold (%): 11.85 6.47
Label Atom Sum: 5.60 (79.95%)

Isotope List used for fitting data:

m/z intensity
151.07 253583
152.08 1028096
153.08 1863572
154.09 394092

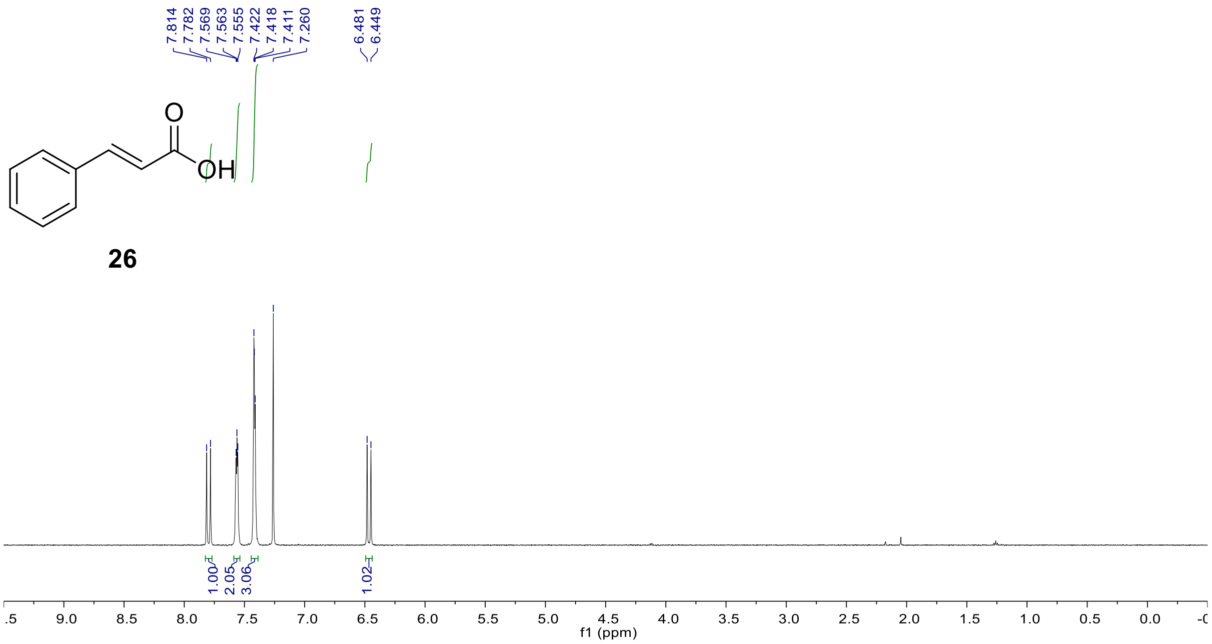

^{1}H NMR spectrum of compound **26** ($CDCl_3$, 500 MHz)

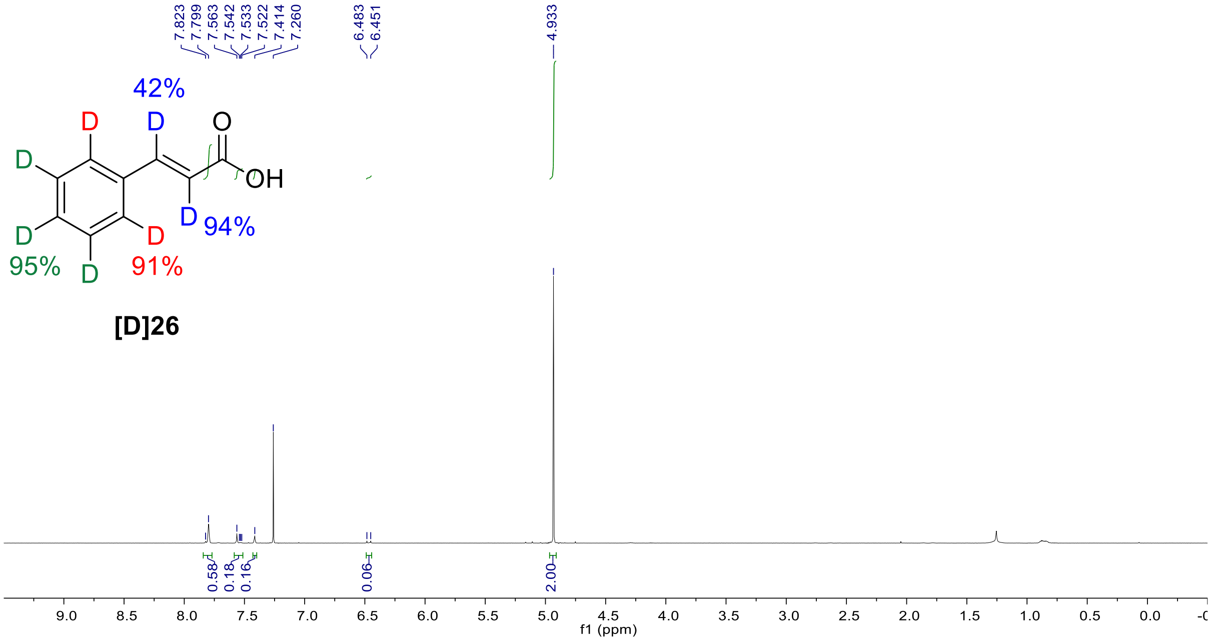

^{1}H NMR spectrum of compound **[D]26** (Procedure A, $CDCl_3$, 500 MHz)

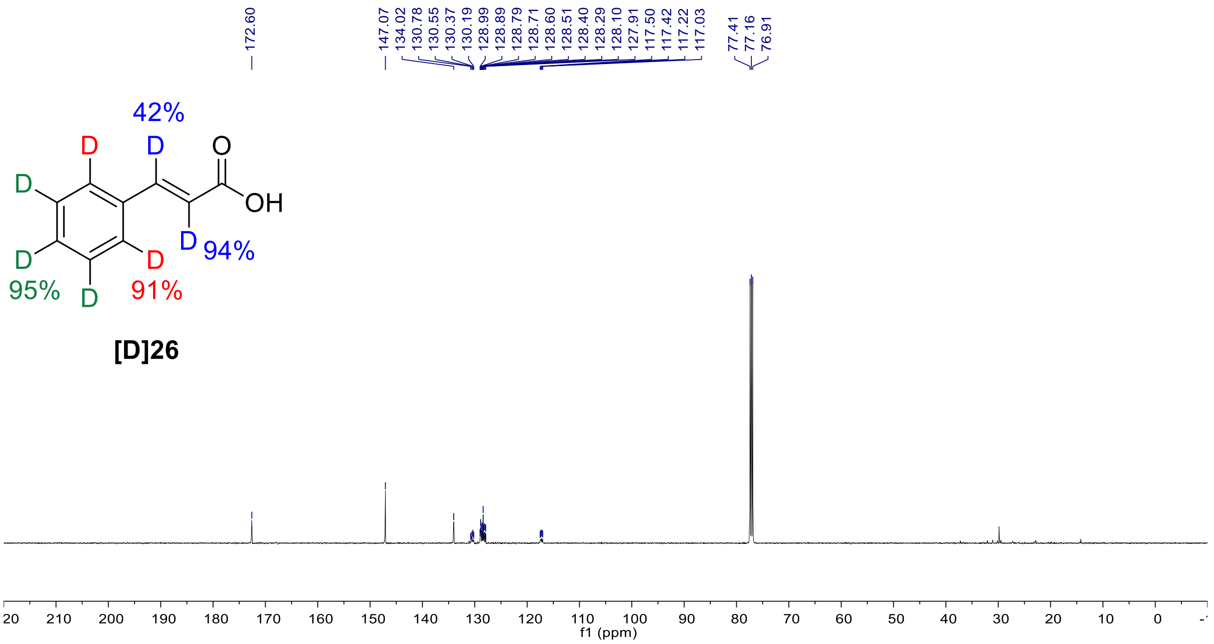

^{13}C NMR spectrum of compound **[D]26** (Procedure A, $CDCl_3$, 126 MHz)

3-methylthiophene-2-carboxylic acid (27)

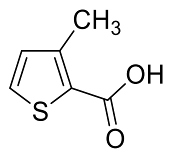

Following the general procedure A, the reaction was set up with 3-methylthiophene-2-carboxylic acid (28.4 mg, 0.20 mmol). Purification by flash column chromatography (hexanes/EtOAc = 9:1) provided product **[D]27** as a white solid (16 mg, 54% yield).

Following the general procedure D, **[D]27** was provided as a white solid (26 mg, 88% yield).

Deuterium Incorporation

General procedure A: [LCMS (ESI)] calcd for $C_6D_5O_2S$ $[M-H]^-$ 3.30 D/molecule, [^{1}H NMR] 3.43 D/molecule.

General procedure D: [GCMS (EI)] calcd for $C_6HD_5O_2S$ $[M]^+$ 4.49 D/molecule, [^{1}H NMR] 4.63 D/molecule.

NMR Data of the Starting Material

^{1}H NMR (500 MHz, $CDCl_3$) δ 7.48 (d, J = 5.0 Hz, 1H), 6.95 (d, J = 5.0 Hz, 1H), 2.58 (s, 3H).

NMR Data of the Product

General procedure A: ^{1}H NMR (500 MHz, $CDCl_3$) δ 7.51 (s, 0.04H, 96% D), 6.97 (s, 0.03H, 97% D), 2.60-2.51 (m, 1.50H, 50% D); ^{13}C NMR (126 MHz, $CDCl_3$) δ 168.9, 148.0, 132.1, 131.9-131.4 (1C), 126.3, 16.3-15.5 (1C).

General procedure D: ^{1}H NMR (500 MHz, $CDCl_3$) δ 7.48 (s, 0.02H, 98% D), 6.95 (s, 0.02H, 98% D), 2.58-2.52 (m, 0.34H, 89% D).

Mass Data

General procedure A

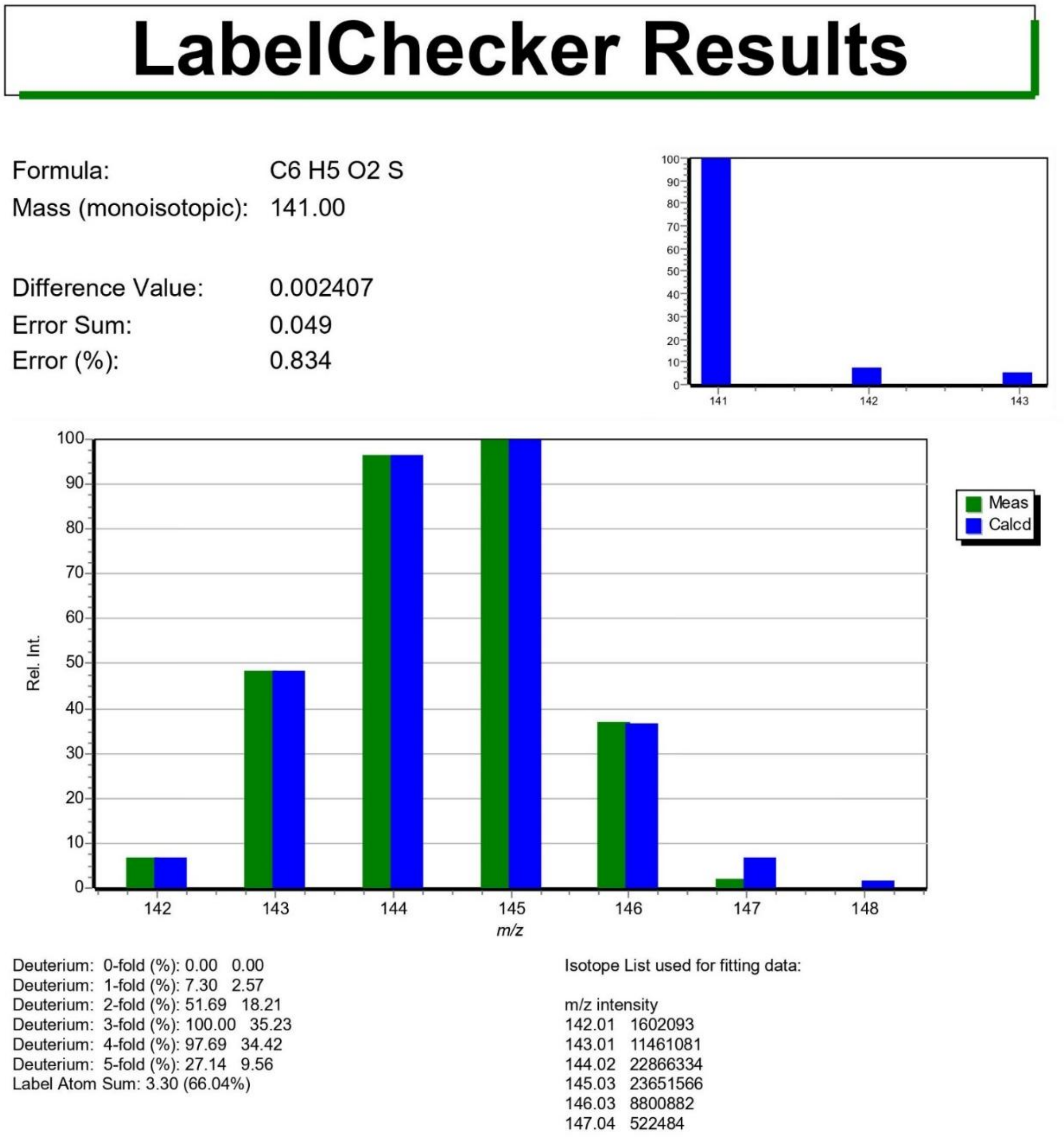

General procedure D

	M+2	M+3	M+4	M+5	M+6
m/z	144	145	146	147	148
Abound	4292	20591	57945	108240	11391
Theoretical exact mass of start material: 142.0089					
Weighted average of deuterated product: 146.5031					
Average %D: 90%					

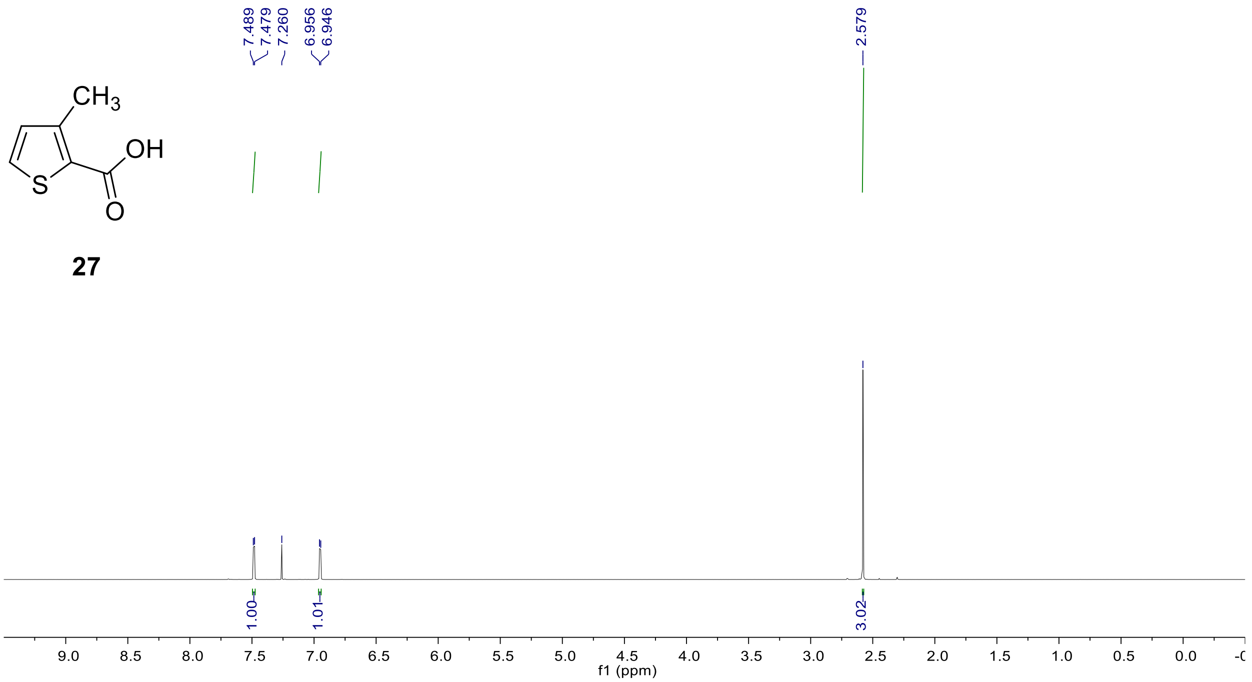

^{1}H NMR spectrum of compound **27** ($CDCl_3$, 500 MHz)

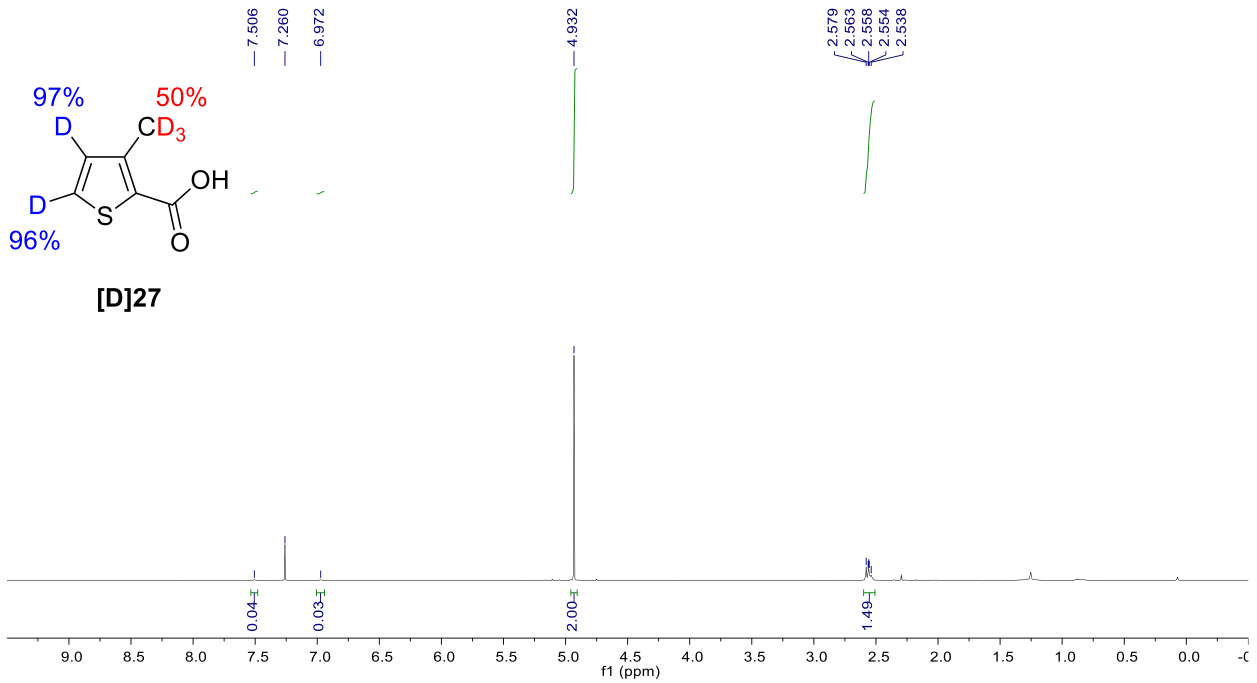

^{1}H NMR spectrum of compound **[D]27** (Procedure A, $CDCl_3$, 500 MHz)

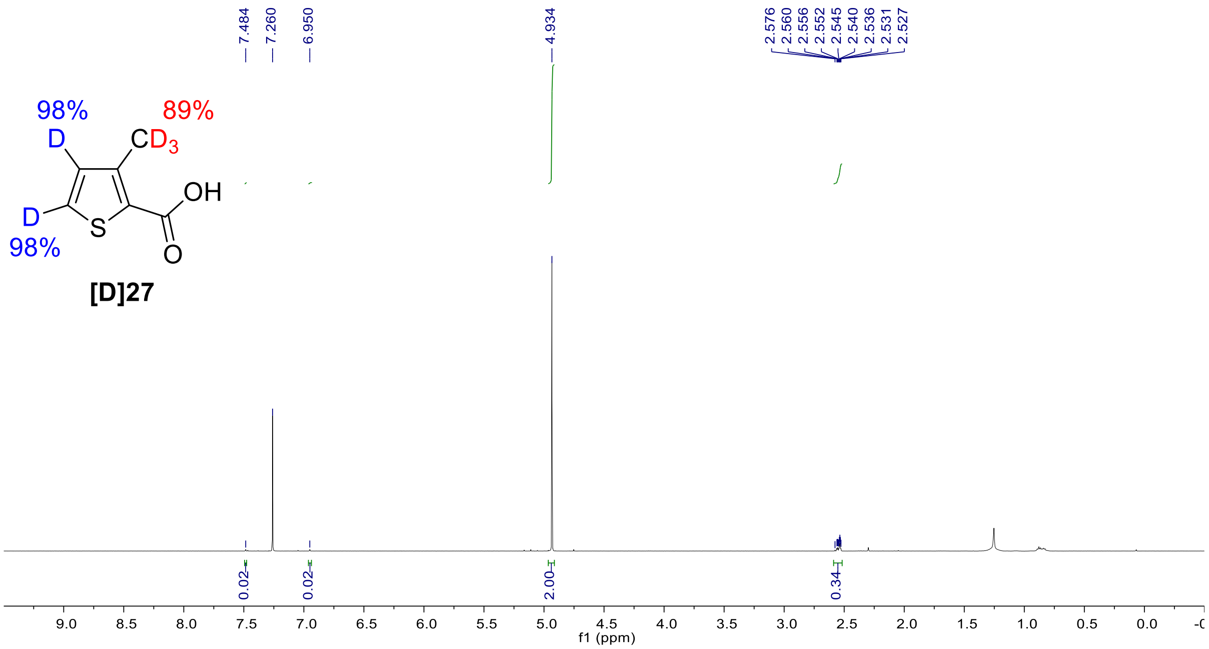

^{1}H NMR spectrum of compound **[D]27** (Procedure D, $CDCl_3$, 500 MHz)

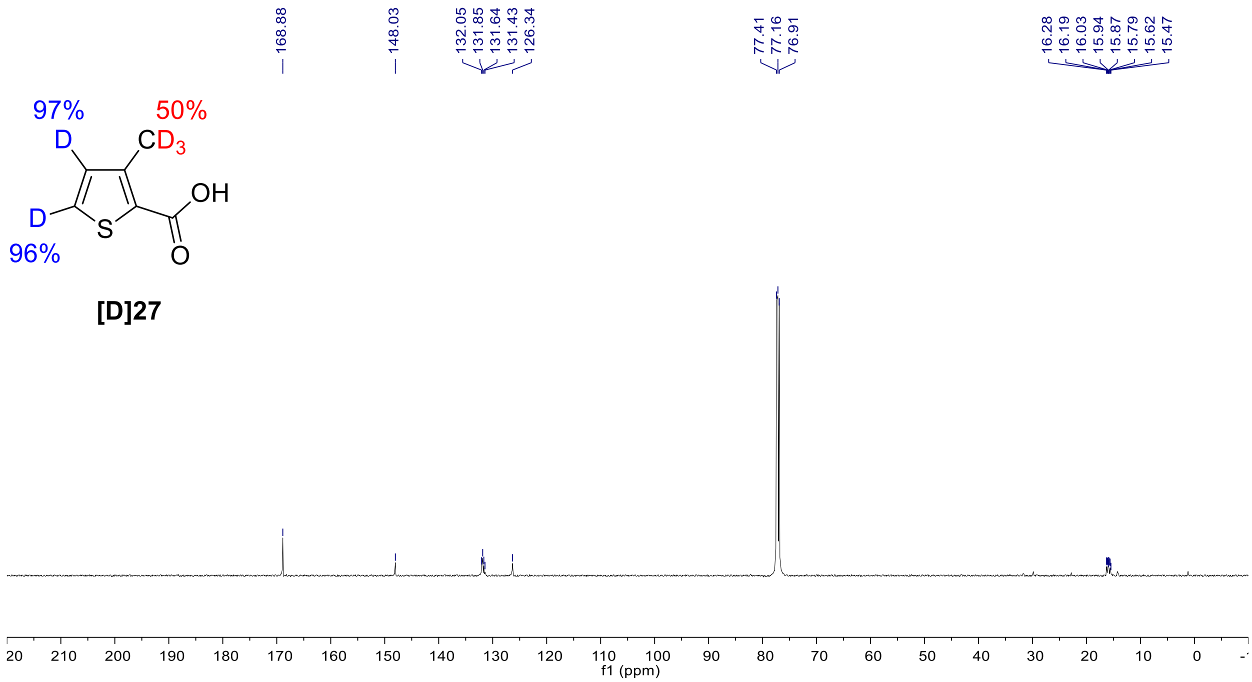

^{13}C NMR spectrum of compound **[D]27** (Procedure A, $CDCl_3$, 126 MHz)

2-methylfuran-3-carboxylic acid (28)

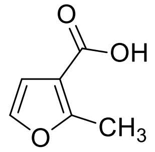

Following the general procedure A, the reaction was set up with 2-methylfuran-3-carboxylic acid (25.2 mg, 0.20 mmol). Purification by flash column chromatography (hexanes/EtOAc/HCO_2H = 5:1:0.05) provided product **[D]28** as a white solid (18 mg, 70% yield).

Following the general procedure D, **[D]28** was provided as a white solid (22 mg, 86% yield).

Deuterium Incorporation

General procedure A: [LCMS (ESI)] calcd for $C_6D_5O_3$ $[M-H]^-$ 2.16 D/molecule, [^1H NMR] 2.13 D/molecule.

General procedure D: [GCMS (EI)] calcd for $C_6HD_5O_3$ $[M]^+$ 3.20 D/molecule, [^1H NMR] 3.22 D/molecule.

NMR Data of the Starting Material

^1H NMR (500 MHz, CD_3OD) δ 7.35 (d, J = 2.0 Hz, 1H), 6.63 (d, J = 2.0 Hz, 1H), 2.54 (s, 3H).

NMR Data of the Product

General procedure A: ^1H NMR (500 MHz, CD_3OD) δ 7.36 (s, 0.04H, 96% D), 6.62 (s, 0.04H, 96% D), 2.54 (s, 2.78H, 7% D); ^{13}C NMR (126 MHz, CD_3OD) δ 167.4, 160.5, 141.9-141.4 (1C), 114.8, 111.7-111.3 (1C), 13.6.

General procedure D: ^1H NMR (500 MHz, CD_3OD) δ 7.36 (s, 0.02H, 98% D), 6.62 (s, 0.02H, 98% D), 2.54 (s, 1.74H, 42% D).

Mass Data

General procedure A

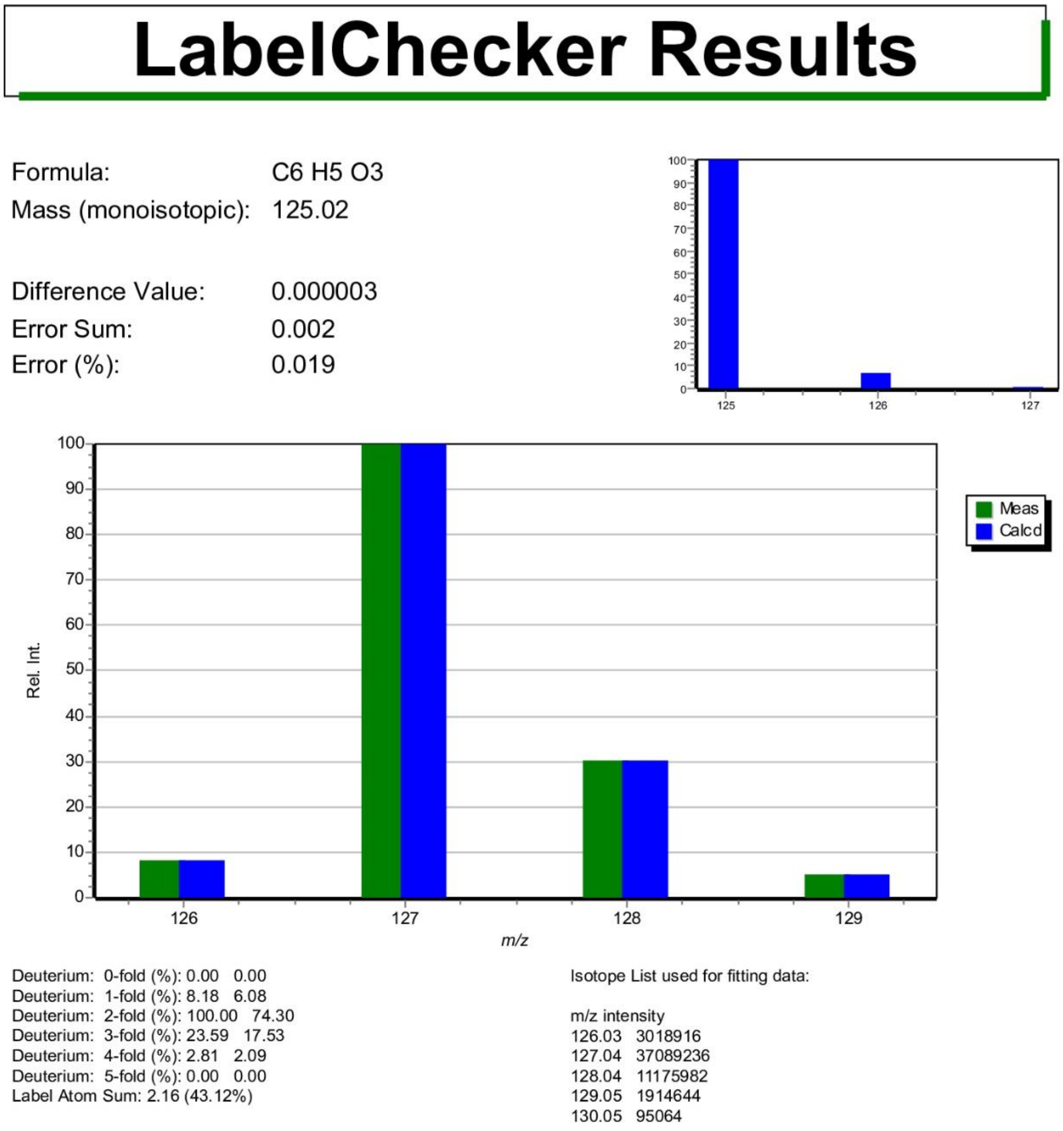

General procedure D

	M+3	M+4	M+5
m/z	129	130	131
Abound	38913	8505	1300
Theoretical exact mass of start material: 126.0317			
Weighted average of deuterated product: 129.2279			
Average %D: 64%			

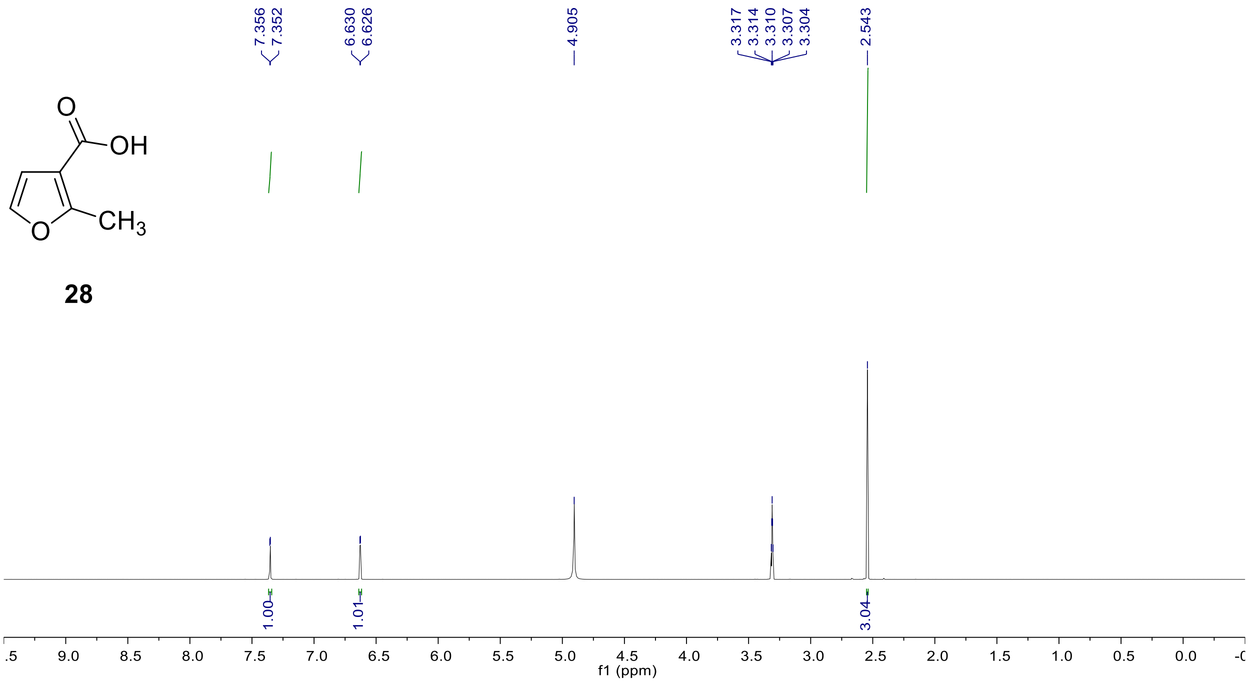

^{1}H NMR spectrum of compound **28** (CD_3OD, 500 MHz)

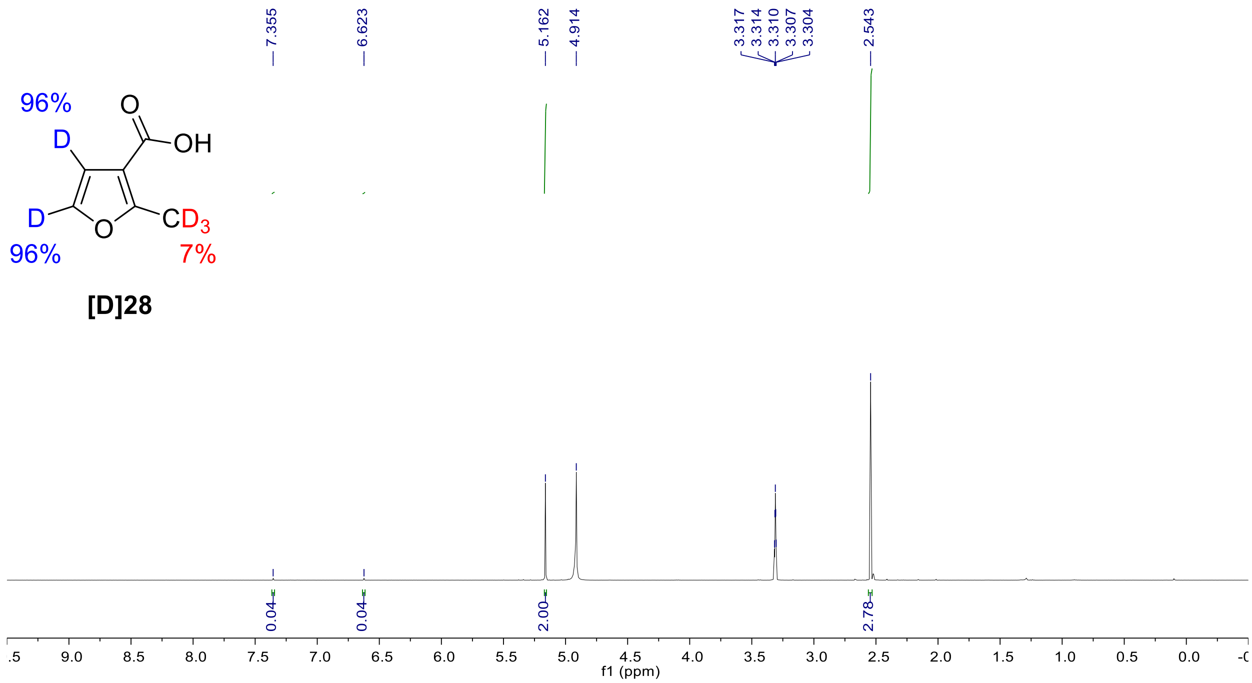

^{1}H NMR spectrum of compound **[D]28** (Procedure A, CD_3OD, 500 MHz)

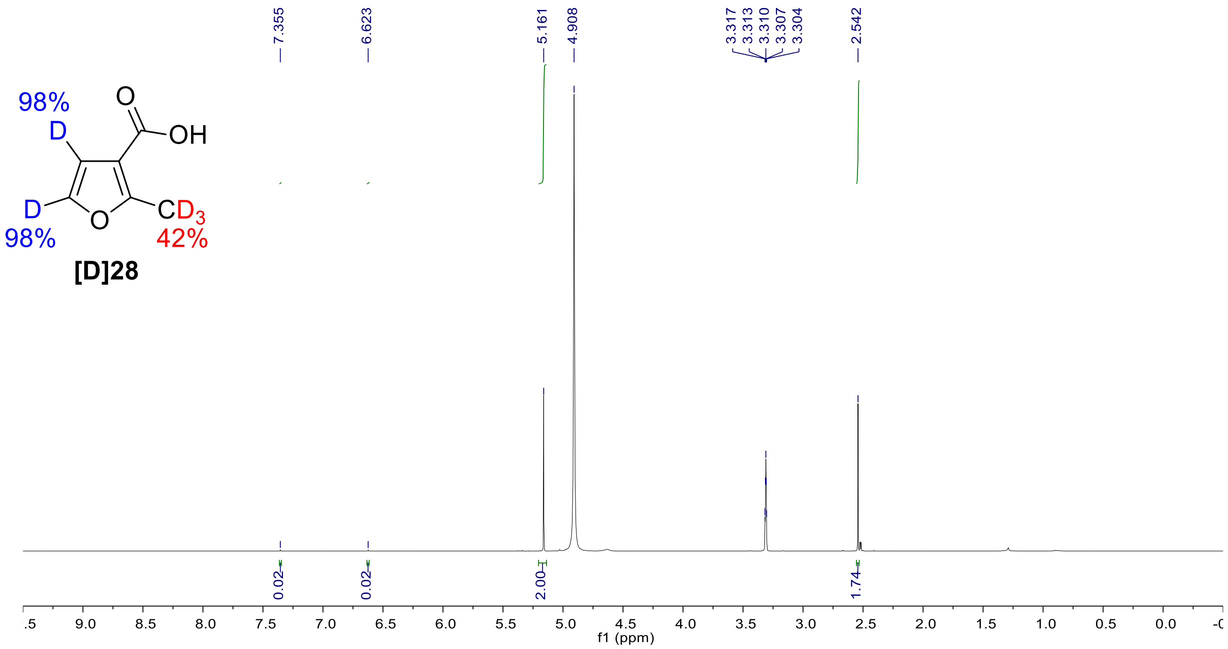

^{1}H NMR spectrum of compound **[D]28** (Procedure D, CD_3OD, 500 MHz)

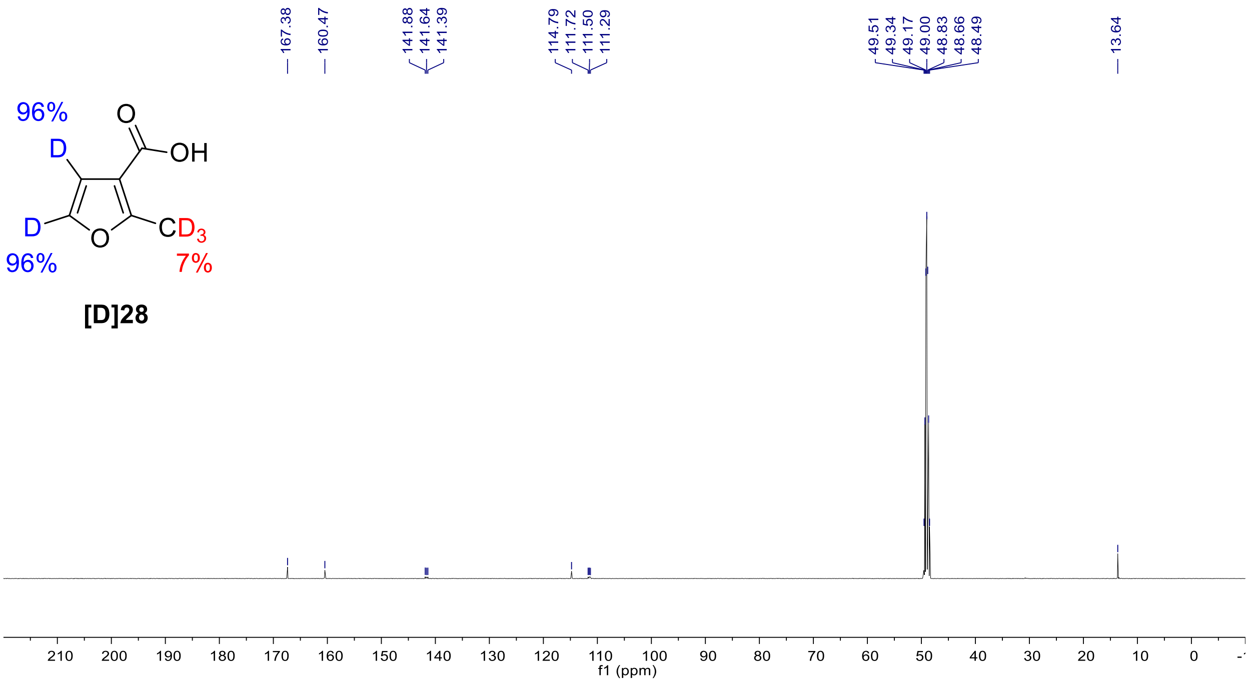

^{13}C NMR spectrum of compound **[D]28** (Procedure A, CD_3OD, 126 MHz)

2-methylnicotinic acid (29)

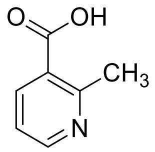

Following the general procedure A, the reaction was set up with 2-methylbenzoic acid (27.4 mg, 0.20 mmol). Purification by flash column chromatography (EtOAc/AcOH = 50:0.06) provided product **[D]29** as a white solid (22 mg, 77% yield).

Deuterium Incorporation

General procedure A: [HRMS (ESI)] calcd for $C_7H_2D_6NO_2$ $[M+H]^+$ 5.02 D/molecule, [^{1}H NMR] 5.10 D/molecule.

NMR Data of the Starting Material

^{1}H NMR (500 MHz, AcOH-*d*$_4$) δ 8.85 (d, *J* = 4.8 Hz, 1H), 8.66 (d, *J* = 7.9, 1H), 7.66 (t, *J* = 7.6, 5.3 Hz, 1H), 2.94 (s, 3H).

NMR Data of the Product

General procedure A: ^{1}H NMR (500 MHz, AcOH-*d*$_4$) δ 8.85 (d, 0.35H, 65% D), 8.65 (d, 0.36H, 64% D), 7.65 (dd, 0.10H, 90% D), 2.90 (s, 0.08H, 97% D); ^{13}C NMR (126 MHz, AcOH-*d*$_4$) δ 170.2, 159.7, 149.7-149.6 (1C), 144.6-144.5 (1C), 129.1, 124.4-123.9 (1C), 24.0.

Mass Data

LabelChecker Results

Formula: C7 H8 N O2
Mass (monoisotopic): 138.06

Difference Value: 0.000076
Error Sum: 0.009
Error (%): 0.094

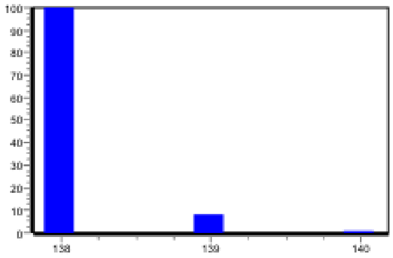

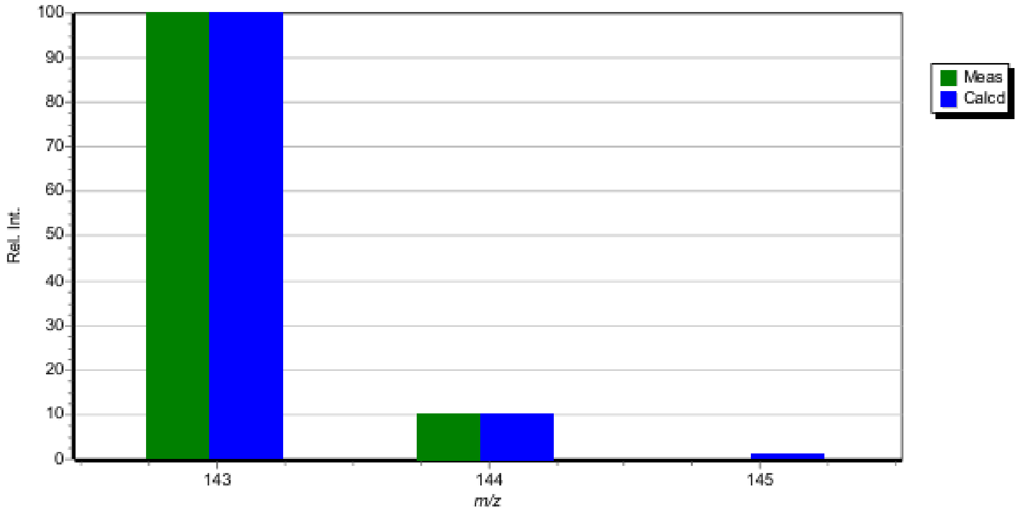

Deuterium: 0-fold (%): 0.00 0.00
Deuterium: 1-fold (%): 0.00 0.00
Deuterium: 2-fold (%): 0.01 0.01
Deuterium: 3-fold (%): 0.00 0.00
Deuterium: 4-fold (%): 0.00 0.00
Deuterium: 5-fold (%): 100.00 97.87
Deuterium: 6-fold (%): 2.16 2.11
Label Atom Sum: 5.02 (62.76%)

Isotope List used for fitting data:

m/z intensity
143.08 291403
144.09 29949

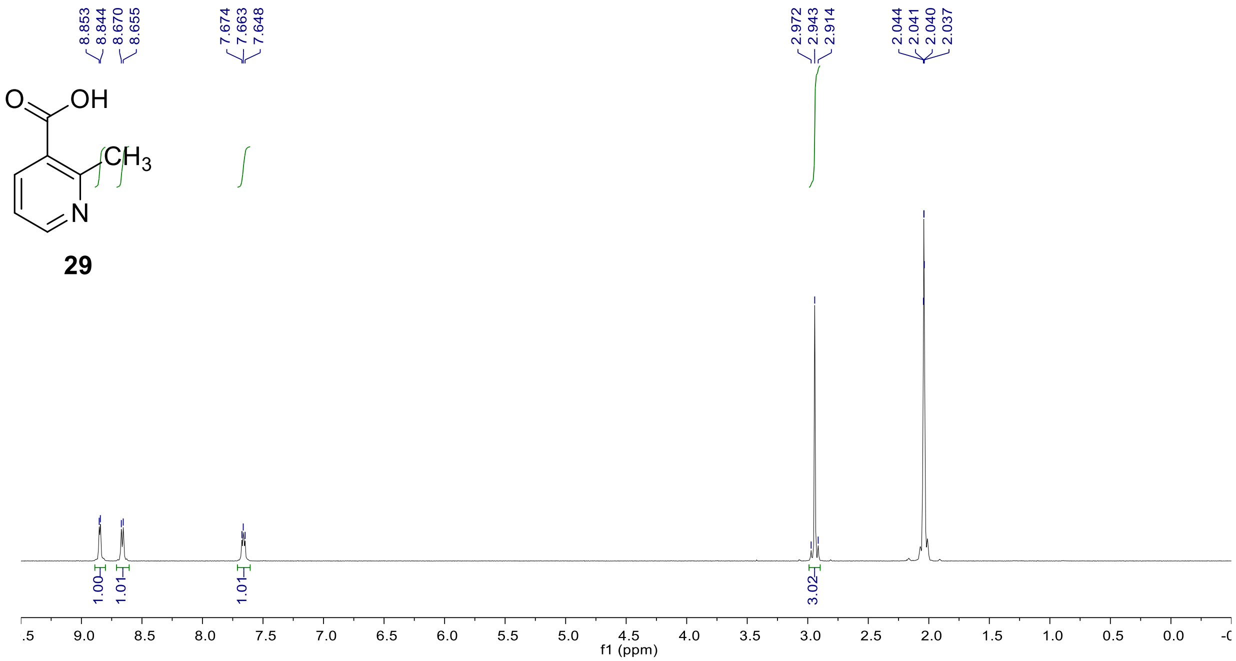

^{1}H NMR spectrum of compound **29** (AcOH-d_4, 500 MHz)

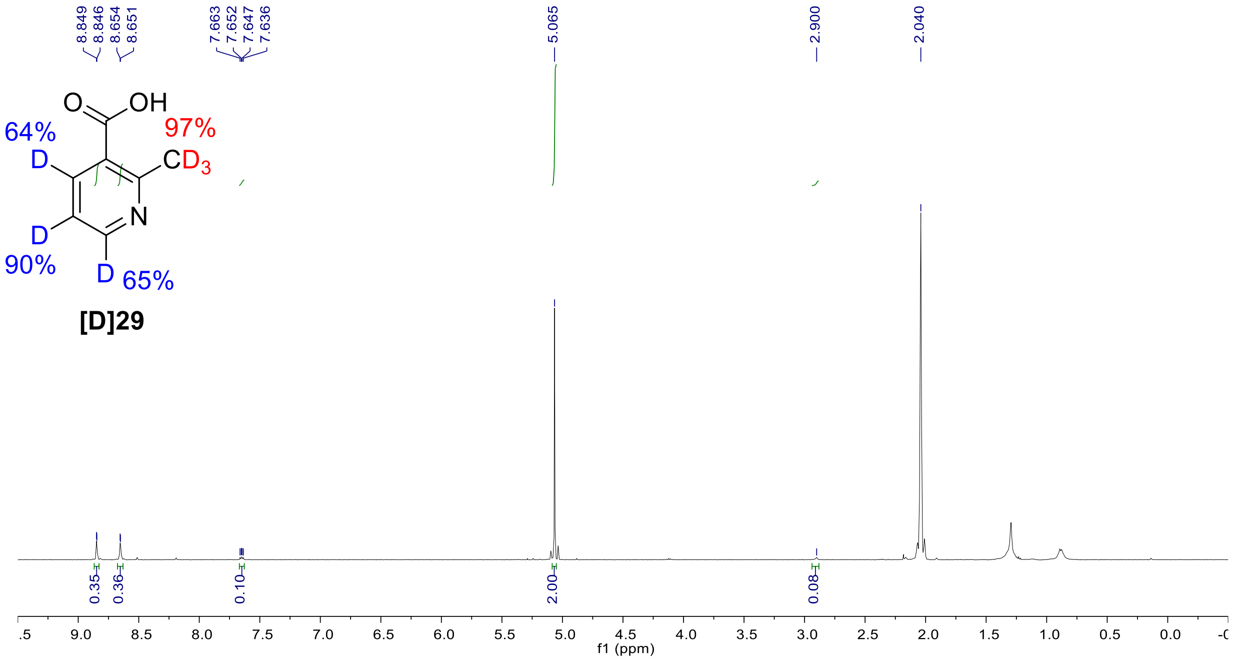

^{1}H NMR spectrum of compound **[D]29** (Procedure A, AcOH-d_4, 500 MHz)

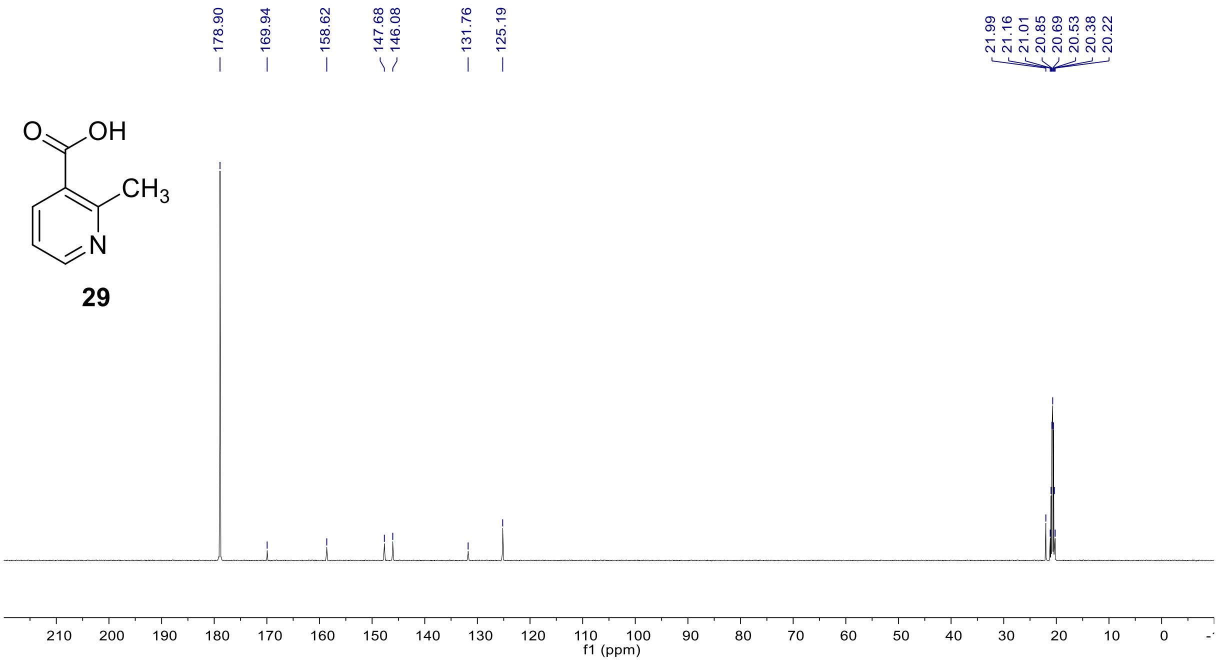

^{13}C NMR spectrum of compound **29** (AcOH-d_4, 126 MHz)

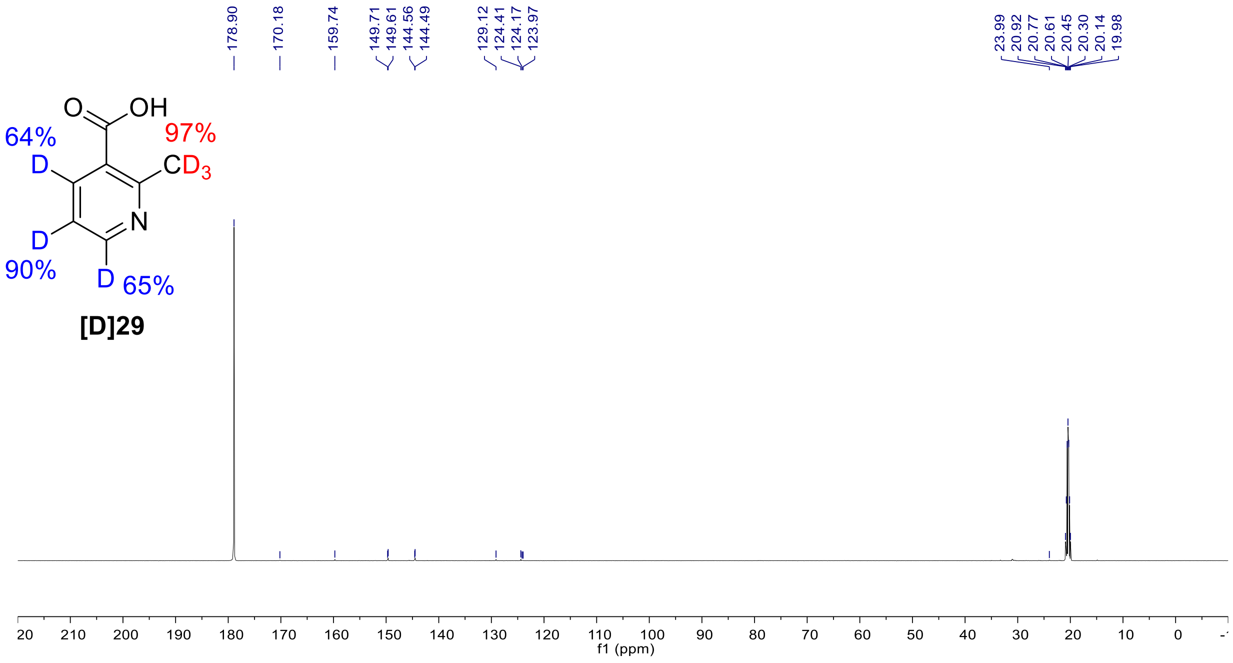

^{13}C NMR spectrum of compound **[D]29** (Procedure A, AcOH-d_4, 126 MHz)

2-ethylbenzoic acid (30)

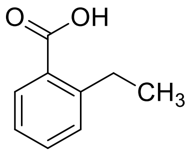

Following the general procedure A, the reaction was set up with 2-ethylbenzoic acid (30.0 mg, 0.20 mmol). Purification by flash column chromatography (hexanes/EtOAc = 16:1) provided product **[D]30** as a white solid (25 mg, 81% yield).

Deuterium Incorporation

General procedure A: [LCMS (ESI)] calcd for $C_9D_9O_2$ $[M-H]^-$ 5.11 D/molecule, [^{1}H NMR] 5.07 D/molecule.

NMR Data of the Starting Material

^{1}H NMR (500 MHz, CD_2Cl_2) δ 8.01 (dd, *J* = 7.8, 1.5 Hz, 1H), 7.51 (td, *J* = 7.5, 1.5 Hz, 1H), 7.36-7.32 (m, 1H), 7.30 (td, *J* = 7.6, 1.3 Hz, 1H), 3.06 (q, *J* = 7.5 Hz, 2H), 1.26 (t, *J* = 7.5 Hz, 3H).

NMR Data of the Product

General procedure A: ^{1}H NMR (500 MHz, CD_2Cl_2) δ 7.97 (s, 0.05H, 95% D), 7.49 (s, 0.06H, 94% D), 7.31 (s, 0.37H, 63% D), 7.27 (s, 0.04H, 96% D), 3.01 (q, *J* = 7.5 Hz, 1.34H, 33% D), 1.22 (t, *J* = 7.5 Hz, 2.06H, 31% D); ^{13}C NMR (126 MHz, CD_2Cl_2) δ 172.9, 147.3-147.2 (1C), 132.9, 131.4-130.9 (1C), 130.5, 127.8, 125.2, 27.7, 15.8.

Mass Data

LabelChecker Results

Formula: C9 H10 O2
Mass (monoisotopic): 150.07

Difference Value: 0.000097
Error Sum: 0.010
Error (%): 0.053

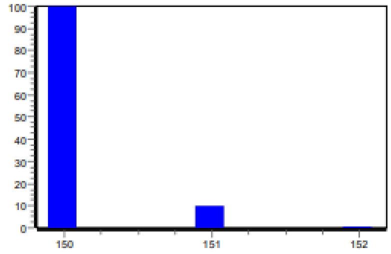

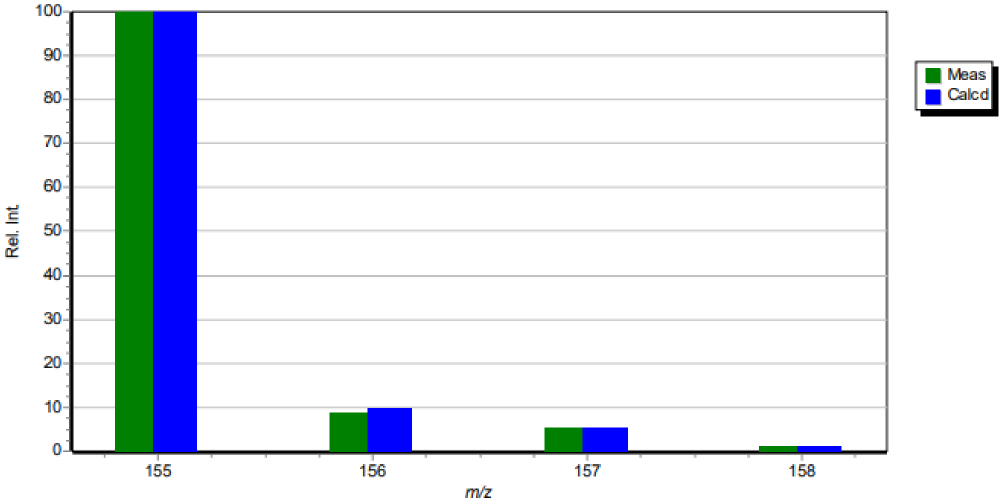

Deuterium: 0-fold (%): 0.00 0.00
Deuterium: 1-fold (%): 0.00 0.00
Deuterium: 2-fold (%): 0.00 0.00
Deuterium: 3-fold (%): 0.00 0.00
Deuterium: 4-fold (%): 0.00 0.00
Deuterium: 5-fold (%): 100.00 94.90
Deuterium: 6-fold (%): 0.00 0.00
Deuterium: 7-fold (%): 4.76 4.51
Deuterium: 8-fold (%): 0.62 0.59
Deuterium: 9-fold (%): 0.00 0.00
Label Atom Sum: 5.11 (51.08%)

Isotope List used for fitting data:

m/z	intensity
155.15	29222648
156.16	2597827
157.17	1636818
158.10	336345

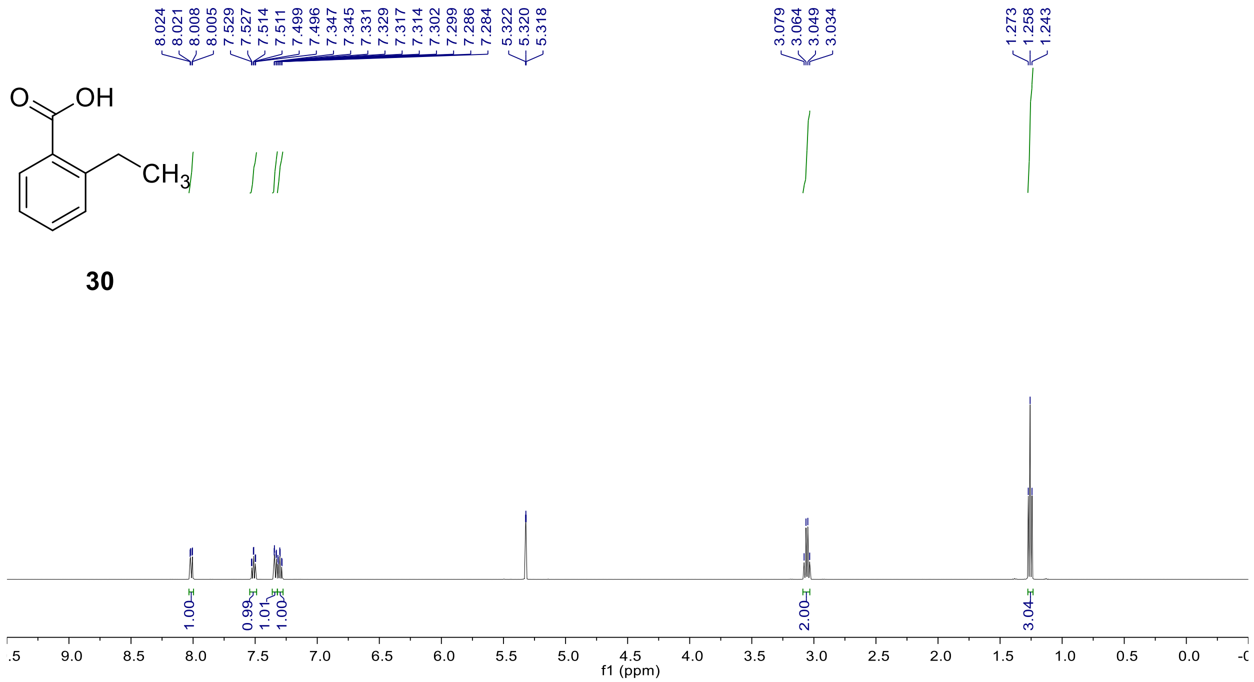

${}^{1}H$ NMR spectrum of compound **30** (CD_2Cl_2, 500 MHz)

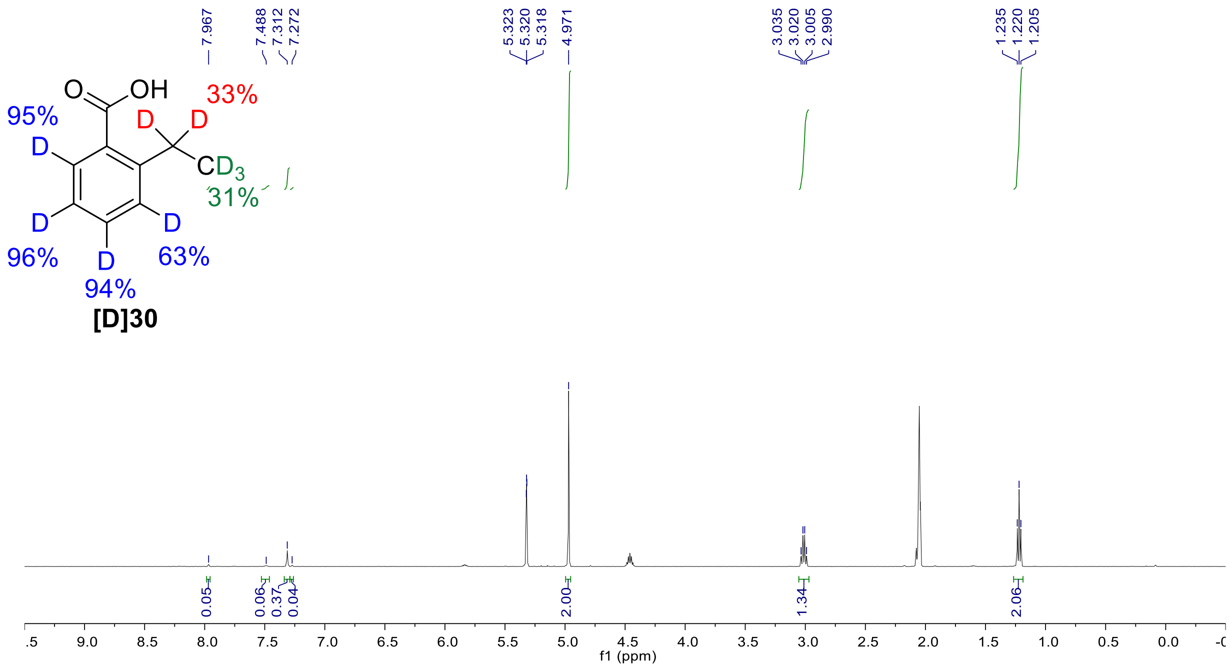

${}^{1}H$ NMR spectrum of compound **[D]30** (Procedure A, CD_2Cl_2, 500 MHz)

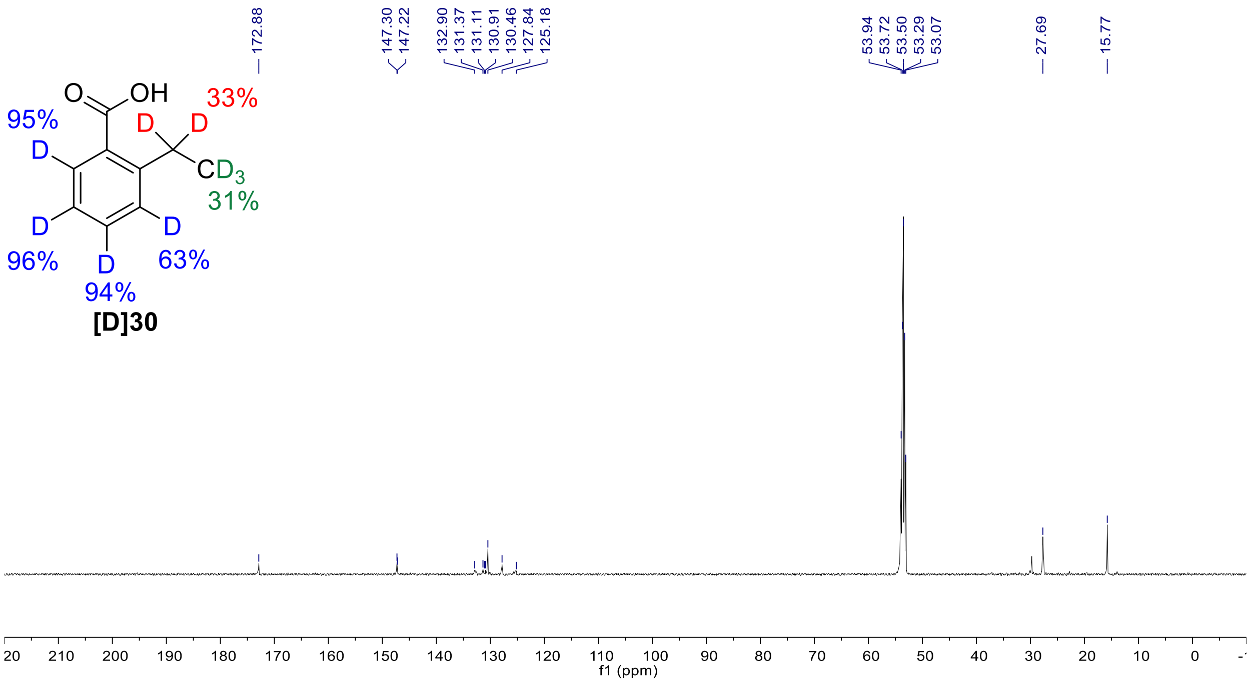

^{13}C NMR spectrum of compound **[D]30** (Procedure A, CD_2Cl_2, 126 MHz)

2-(4-chlorophenyl)-3-methylbutanoic acid (31)

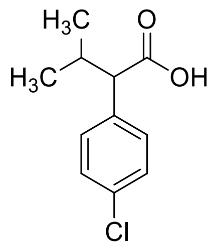

Following the general procedure A, the reaction was set up with 2-(4-chlorophenyl)-3-methylbutanoic acid (42.5 mg, 0.20 mmol). The crude product **[D]31** was filtered and solvent was removed. Subsequently, the reaction mixture was dissolved in acetone (5 mL), and Cs_2CO_3 (78.2 mg, 0.24 mmol) and methyl iodide (0.10 mL, 1.6 mmol) were added. The mixture was stirred overnight at room temperature and all volatiles were removed under reduced pressure. Purification by flash column chromatography (Hexane/EtOAc = 24:1) provided product **[D]31-Me** as a yellow liquid (36 mg, 78% yield).

Deuterium Incorporation ([D]31)

General procedure A: [LCMS (ESI)] calcd for $C_{11}H_9D_4ClO_2$ $[M+H]^+$ 4.00 D/molecule, [^{1}H NMR] 3.84 D/molecule.

NMR Data of the Product ([D]31-Me)

General procedure A: ^{1}H NMR (500 MHz, CD_3OD) δ 7.32 (s, 0.18H, 96% D), 3.64 (s, 3H), 3.20 (d, J = 10.6 Hz, 0.94H, 6% D), 2.36-2.23 (m, 0.88H, 12% D), 1.02 (d, J = 6.5 Hz, 2.79H, 0.70 (d, J = 6.7 Hz, 2.89H, 4% D); ^{13}C NMR (126 MHz, $CDCl_3$) δ 174.3, 136.8, 133.1, 129.8-129.3 (2C), 128.7-128.2 (2C), 59.3, 52.0, 32.2, 21.5, 20.3.

Mass Data

General procedure A

LabelChecker Results

Formula: C11 H13 O2 Cl
Mass (monoisotopic): 212.06

Difference Value: 1.313002
Error Sum: 1.146
Error (%): 0.149

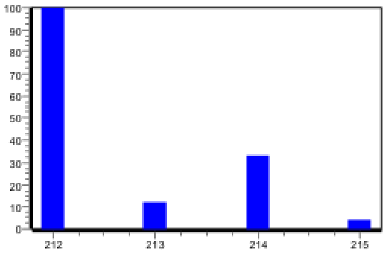

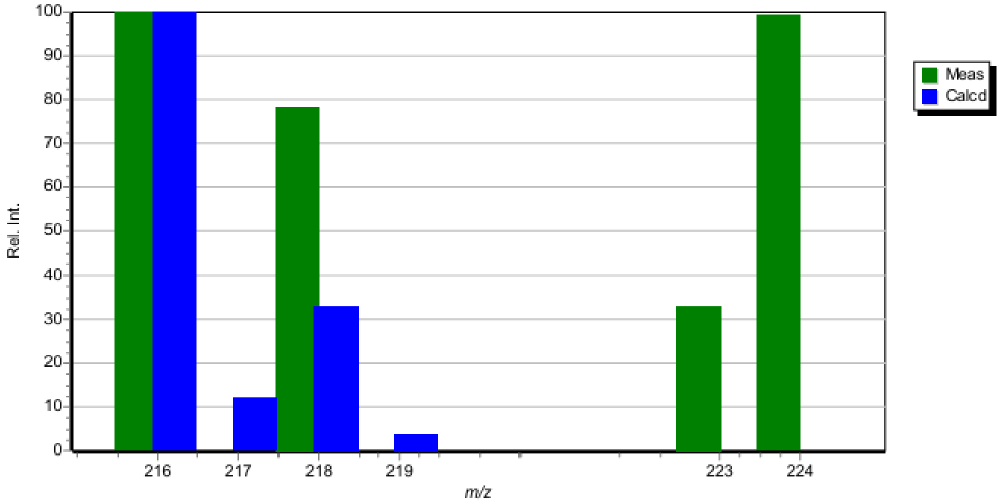

No Convergence! - Max Cycles (20) reached!
Deuterium: 0-fold (%): 0.00 0.00
Deuterium: 1-fold (%): 0.00 0.00
Deuterium: 2-fold (%): 0.00 0.00
Deuterium: 3-fold (%): 0.00 0.00
Deuterium: 4-fold (%): 100.00 100.00
Label Atom Sum: 4.00 (30.77%)

Isotope List used for fitting data:

m/z	intensity
216.13	3239
218.21	2539
223.09	1065
224.13	3212

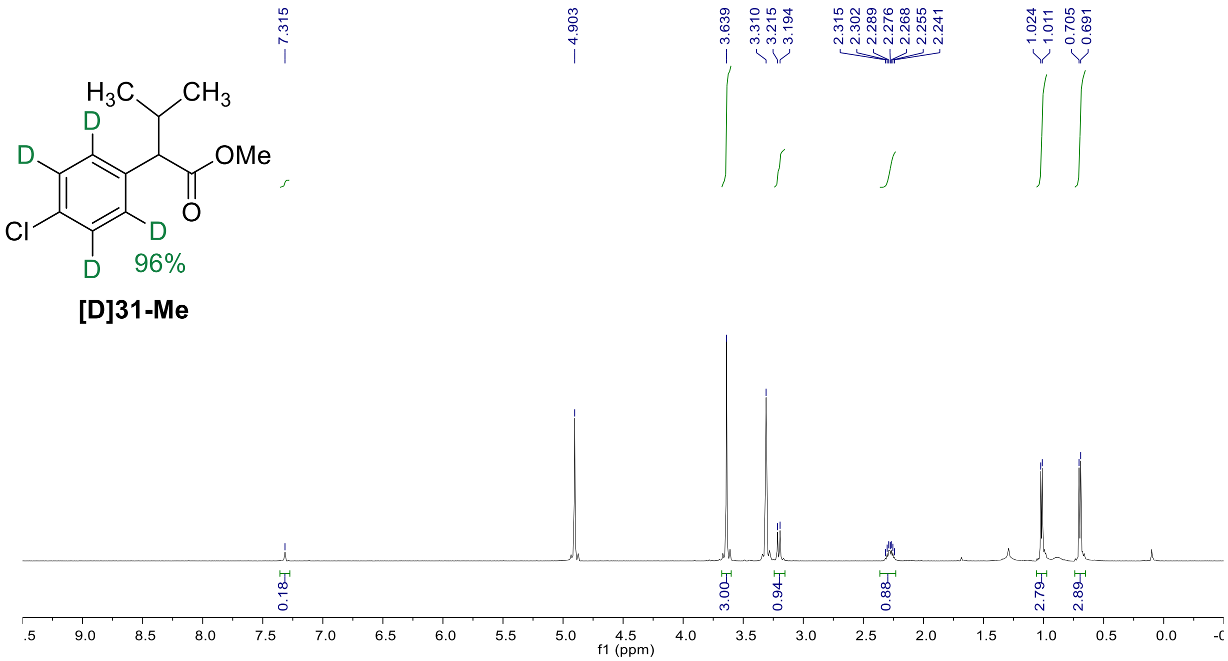

^{1}H NMR spectrum of compound **[D]31-Me** (Procedure A, CD_3OD, 500 MHz)

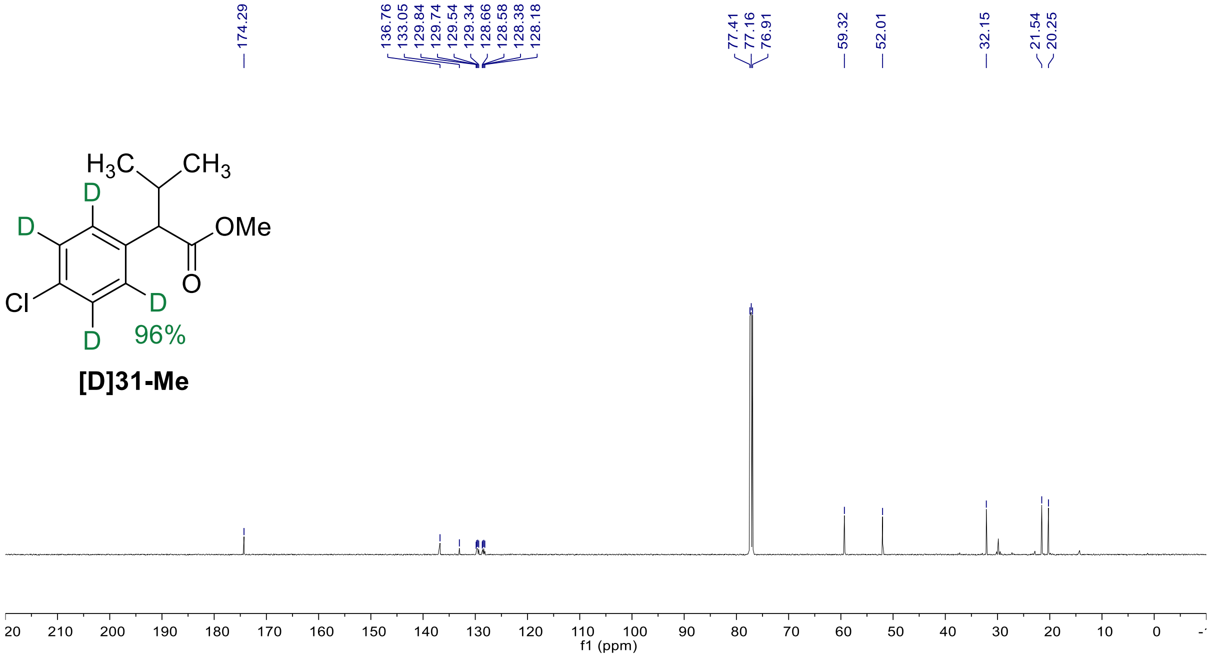

^{13}C NMR spectrum of compound **[D]31-Me** (Procedure A, $CDCl_3$, 126 MHz)

2-(4-isobutylphenyl)propanoic acid (ibuprofen, 32)

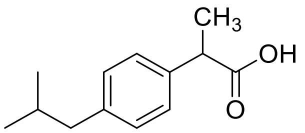

Following the general procedure A, the reaction was set up with 2-(4-isobutylphenyl)propanoic acid (41.2 mg, 0.20 mmol). Purification by flash column chromatography (hexanes/EtOAc = 15:1) provided product **[D]32** as a yellow liquid (34 mg, 80% yield).

Following the general procedure D, **[D]32** was provided as a yellow liquid (40 mg, 95% yield).

Deuterium Incorporation

General procedure A: [LCMS (ESI)] calcd for $C_{13}H_9D_8O_2$ $[M-H]^-$ 5.90 D/molecule, [^{1}H NMR] 5.62 D/molecule.

General procedure D: [GCMS (EI)] calcd for $C_{13}H_9D_8O_2$ $[M]^+$ 5.41 D/molecule, [^{1}H NMR] 5.87 D/molecule.

NMR Data of the Starting Material

^{1}H NMR (500 MHz, $CDCl_3$) δ 7.22 (d, *J* = 8.1 Hz, 2H), 7.10 (d, *J* = 8.1 Hz, 2H), 3.71 (q, *J* = 7.2 Hz, 1H), 2.44 (d, *J* = 7.1 Hz, 2H), 1.84 (hept, *J* = 6.7 Hz, 1H), 1.50 (d, *J* = 7.2 Hz, 3H), 0.89 (d, *J* = 6.6 Hz, 6H).

NMR Data of the Product

General procedure A: ^{1}H NMR (500 MHz, $CDCl_3$) δ 7.24-7.21 (m, 0.08H, 96% D), 7.12 (d, *J* = 14.4 Hz, 1.08H, 46% D), 3.75-3.68 (m, 0.62H, 38% D), 2.50-2.41 (m, 1.94H, 3% D), 1.93-1.80 (m, 0.98H, 2% D), 1.53-1.46 (m, 0.61H, 80% D), 0.90 (t, *J* = 6.9 Hz, 6H); ^{13}C NMR (126 MHz, $CDCl_3$) δ 181.2, 141.0-140.9 (1C), 136.9, 129.4-128.9 (2C), 127.4- 126.8 (2C), 45.2-44.9 (2C), 30.3, 22.5, 18.0-17.5 (1C).

General procedure D: ^{1}H NMR (500 MHz, $CDCl_3$) δ 7.23-7.21 (m, 0.07H, 97% D), 7.12 (d, *J* = 14.4 Hz, 1.28H, 36% D), 3.73-3.69 (m, 0.63H, 37% D), 2.49-2.43 (m, 1.74H, 13% D), 1.91-1.79 (m, 0.85H, 15% D), 1.51-1.46 (m, 0.20H, 93% D), 0.95-0.82 (m, 6.03H).

Mass Data

General procedure A

LabelChecker Results

Formula: C13 H17 O2

Mass (monoisotopic): 205.12

Difference Value: 0.000822

Error Sum: 0.029

Error (%): 0.351

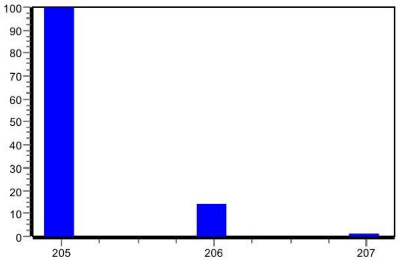

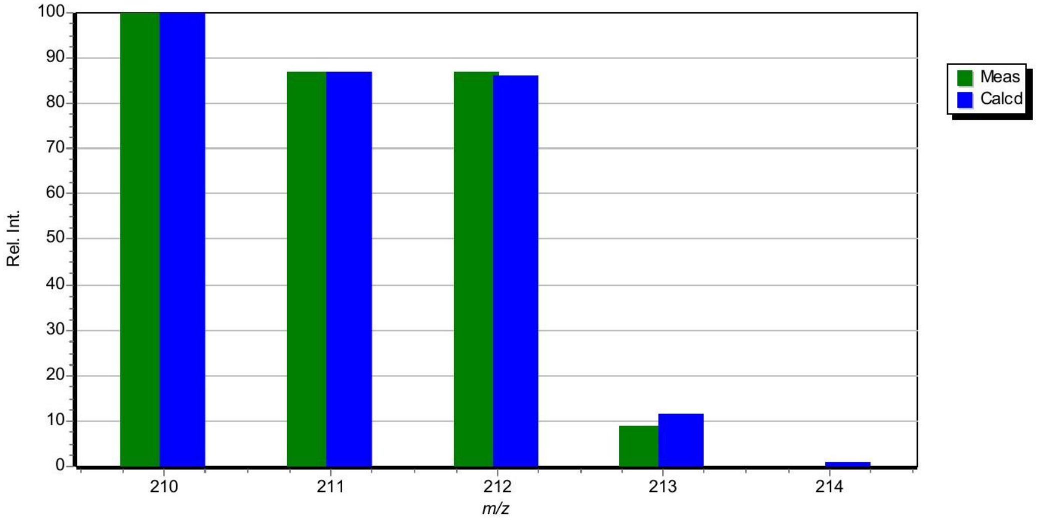

Deuterium: 0-fold (%): 0.00 0.00
Deuterium: 1-fold (%): 0.00 0.00
Deuterium: 2-fold (%): 0.00 0.00
Deuterium: 3-fold (%): 0.00 0.00
Deuterium: 4-fold (%): 0.00 0.00
Deuterium: 5-fold (%): 100.00 40.45
Deuterium: 6-fold (%): 72.54 29.34
Deuterium: 7-fold (%): 74.69 30.21
Deuterium: 8-fold (%): 0.00 0.00
Label Atom Sum: 5.90 (34.69%)

Isotope List used for fitting data:

m/z	intensity
210.15	3155349
211.16	2738895
212.17	2738895
213.17	287112

General procedure D

	M+5	M+6	M+7	M+8
m/z	211	212	213	214
Abound	146577	87364	21984	3014
Theoretical exact mass of start material: 206.1307				
Weighted average of deuterated product: 211.5421				
Average %D: 68%				

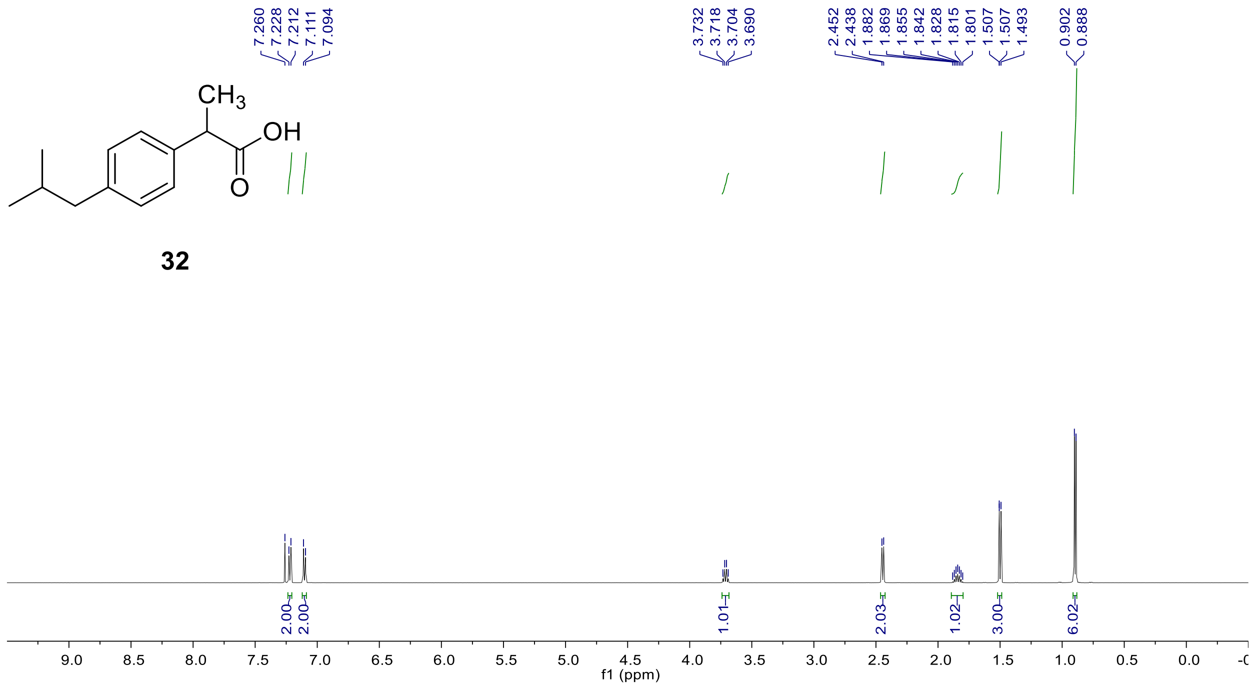

^{1}H NMR spectrum of compound **32** ($CDCl_3$, 500 MHz)

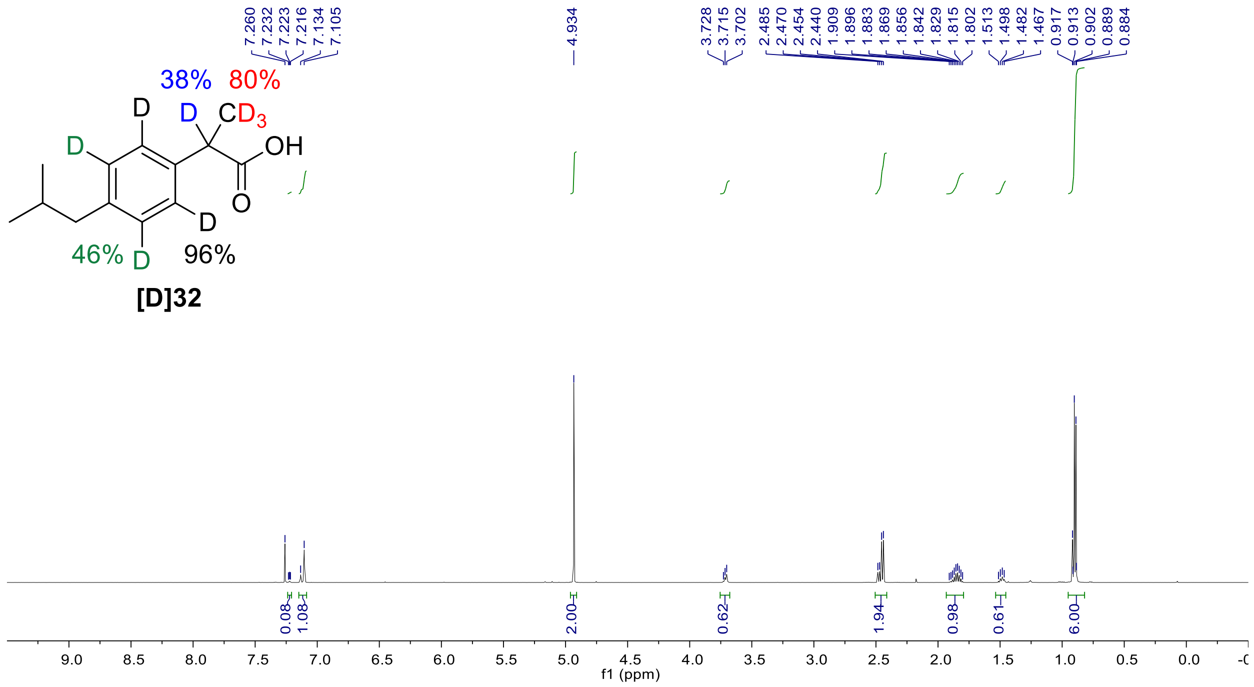

^{1}H NMR spectrum of compound **[D]32** (Procedure A, $CDCl_3$, 500 MHz)

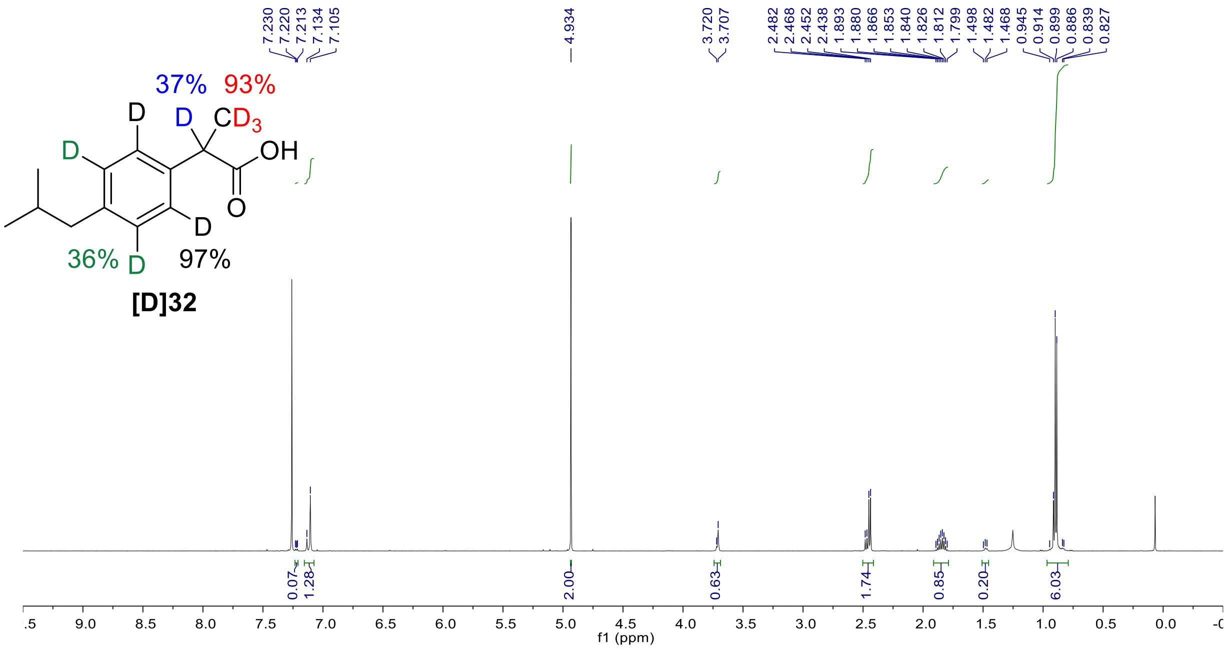

^{1}H NMR spectrum of compound **[D]32** (Procedure D, $CDCl_3$, 500 MHz)

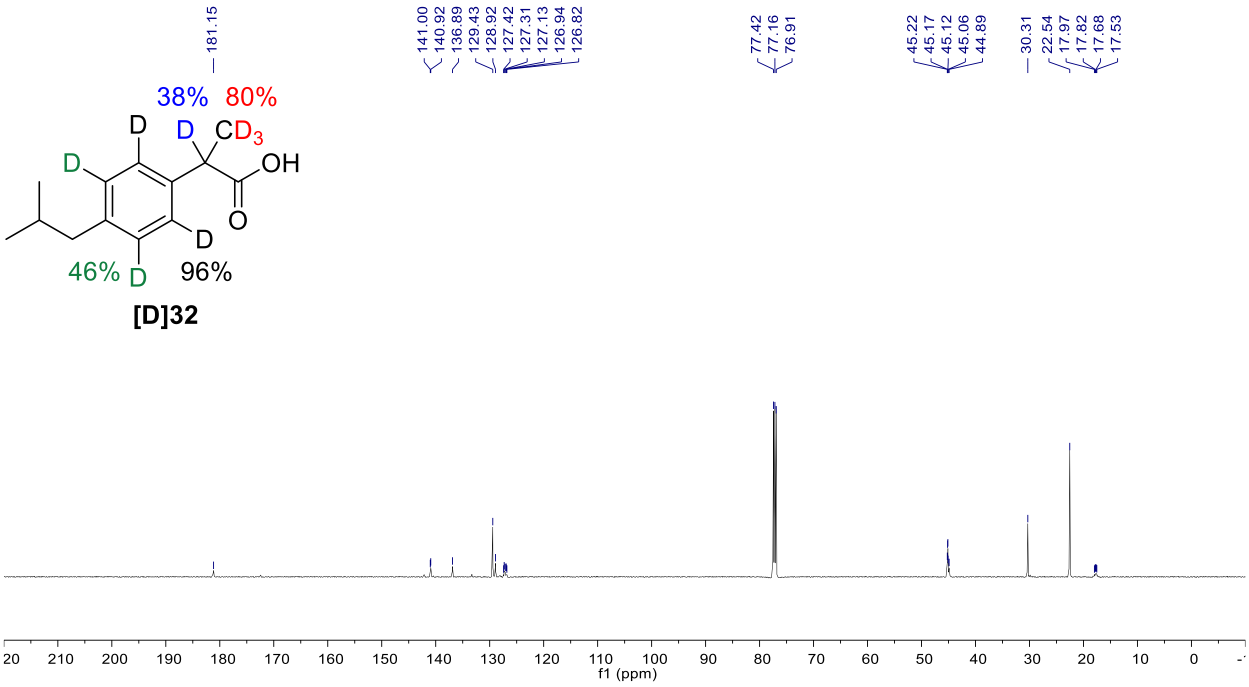

^{13}C NMR spectrum of compound **[D]32** (Procedure A, $CDCl_3$, 126 MHz)

5-(2,5-dimethylphenoxy)-2,2-dimethylpentanoic acid (gemfibrozil, 33)

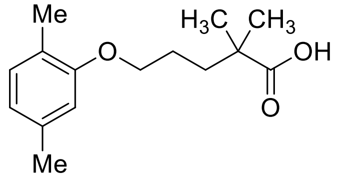

Following the general procedure A, the reaction was set up with 5-(2,5-dimethylphenoxy)-2,2-dimethylpentanoic acid (50.0 mg, 0.20 mmol). Purification by flash column chromatography (hexanes/EtOAc/HCO_2H = 4:1:0.04) provided product **[D]33** as a white solid (44 mg, 85% yield).

Deuterium Incorporation

General procedure A: [LCMS (ESI)] calcd for $C_{15}H_{10}D_{11}O_3$ $[M-H]^-$ 7.86 D/molecule, [^{1}H NMR] 7.95 D/molecule.

NMR Data of the Starting Material

^{1}H NMR (500 MHz, $CDCl_3$) δ 7.00 (d, J = 7.5 Hz, 1H), 6.66 (d, J = 7.5 Hz, 1H), 6.61 (s, 1H), 3.93 (t, J = 6.0 Hz, 2H), 2.30 (s, 3H), 2.17 (s, 3H), 1.84-1.72 (m, 4H), 1.25 (s, 6H).

NMR Data of the Product

General procedure A: ^{1}H NMR (500 MHz, $CDCl_3$) δ 7.01 (s, 0.21H, 79% D), 6.66 (s, 0.14H, 86% D), 6.61 (s, 0.46H, 54% D), 3.93 (t, J = 6.0 Hz, 1.93H, 4% D), 2.31 (s, 2.91H, 3% D), 2.18 (s, 2.93H, 2% D), 1.85-1.71 (m, 3.54H, 12% D), 1.24-1.19 (m, 0.51H, 92% D); ^{13}C NMR (126 MHz, $CDCl_3$) δ 185.0, 157.1-157.0 (1C), 136.6-136.4 (1C), 130.4-129.8 (1C), 123.7-123.6 (1C), 120.8-120.2 (1C), 112.0-111.5 (1C), 68.0, 41.8-41.7 (1C), 36.9, 25.2-25.1 (1C), 21.6-21.4 (2C), 15.9-15.8 (2C).

Mass Data

LabelChecker Results

Formula:	C15 H21 O3
Mass (monoisotopic):	249.15
Difference Value:	0.000268
Error Sum:	0.016
Error (%):	0.097

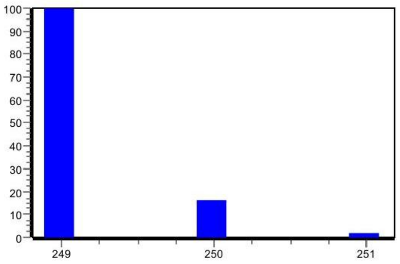

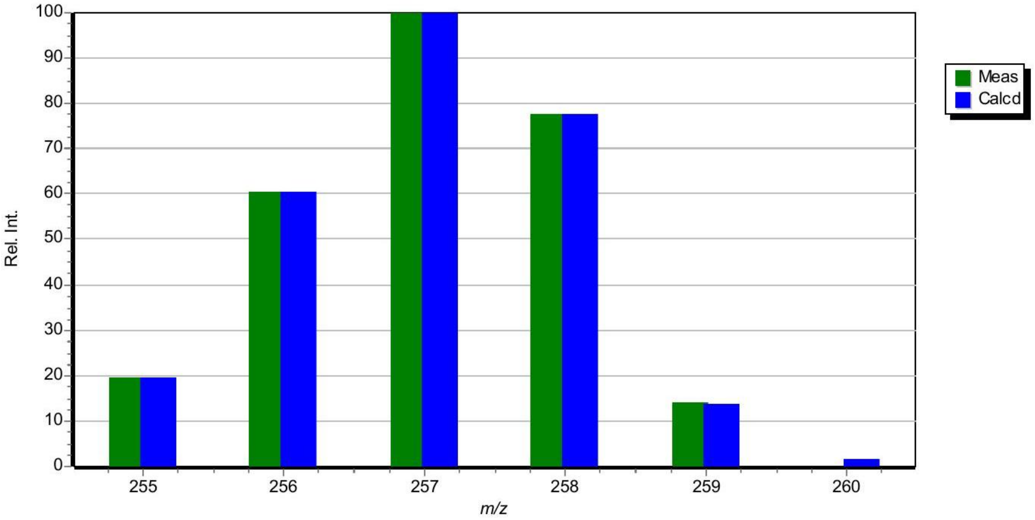

Deuterium: 0-fold (%): 0.01 0.01
Deuterium: 1-fold (%): 0.00 0.00
Deuterium: 2-fold (%): 0.00 0.00
Deuterium: 3-fold (%): 0.00 0.00
Deuterium: 4-fold (%): 0.00 0.00
Deuterium: 5-fold (%): 0.00 0.00
Deuterium: 6-fold (%): 21.85 8.54
Deuterium: 7-fold (%): 63.65 24.87
Deuterium: 8-fold (%): 100.00 39.08
Deuterium: 9-fold (%): 68.40 26.73
Deuterium: 10-fold (%): 2.00 0.78
Deuterium: 11-fold (%): 0.00 0.00
Label Atom Sum: 7.86 (37.44%)

Isotope List used for fitting data:

m/z	intensity
255.19	5229800
256.19	16102509
257.20	26556618
258.21	20619804
259.21	3725283

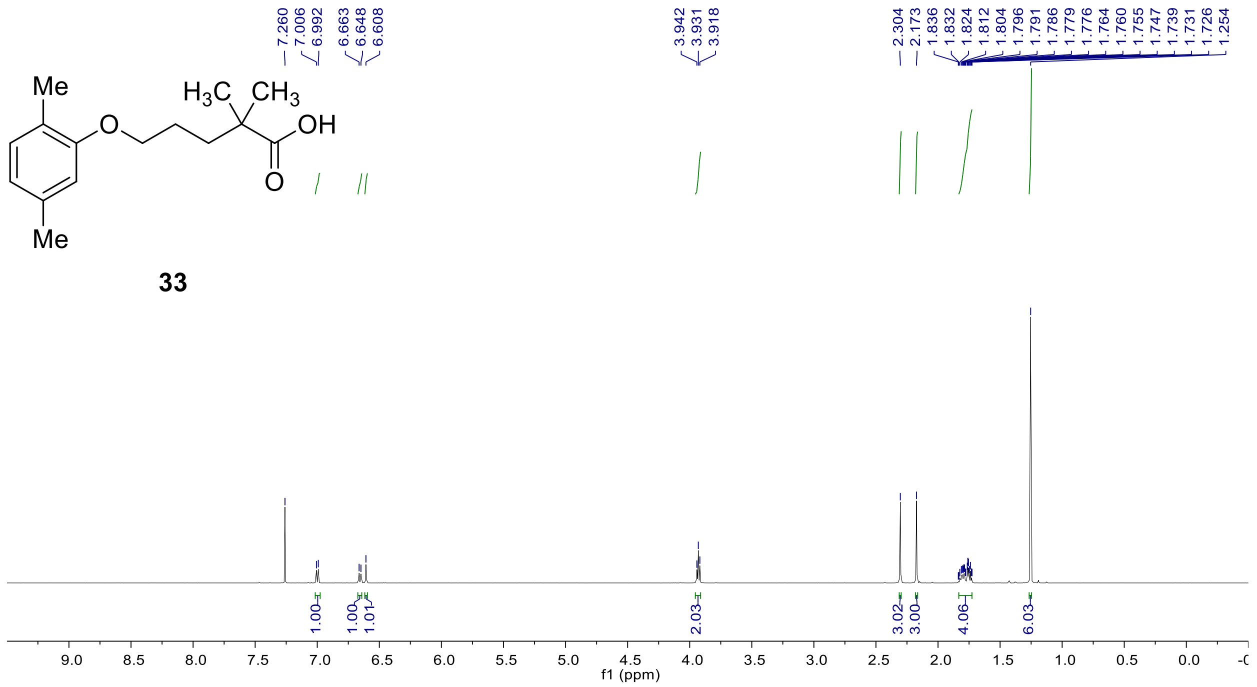

^{1}H NMR spectrum of compound **33** ($CDCl_3$, 500 MHz)

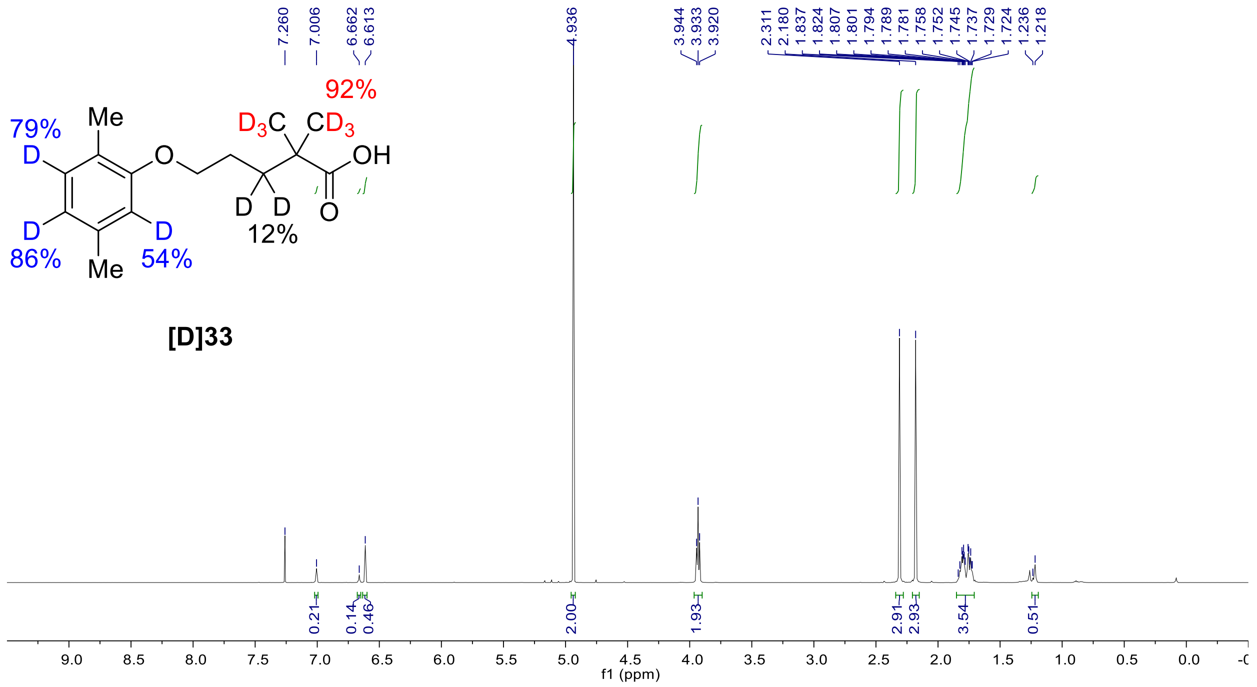

^{1}H NMR spectrum of compound **[D]33** (Procedure A, $CDCl_3$, 500 MHz)

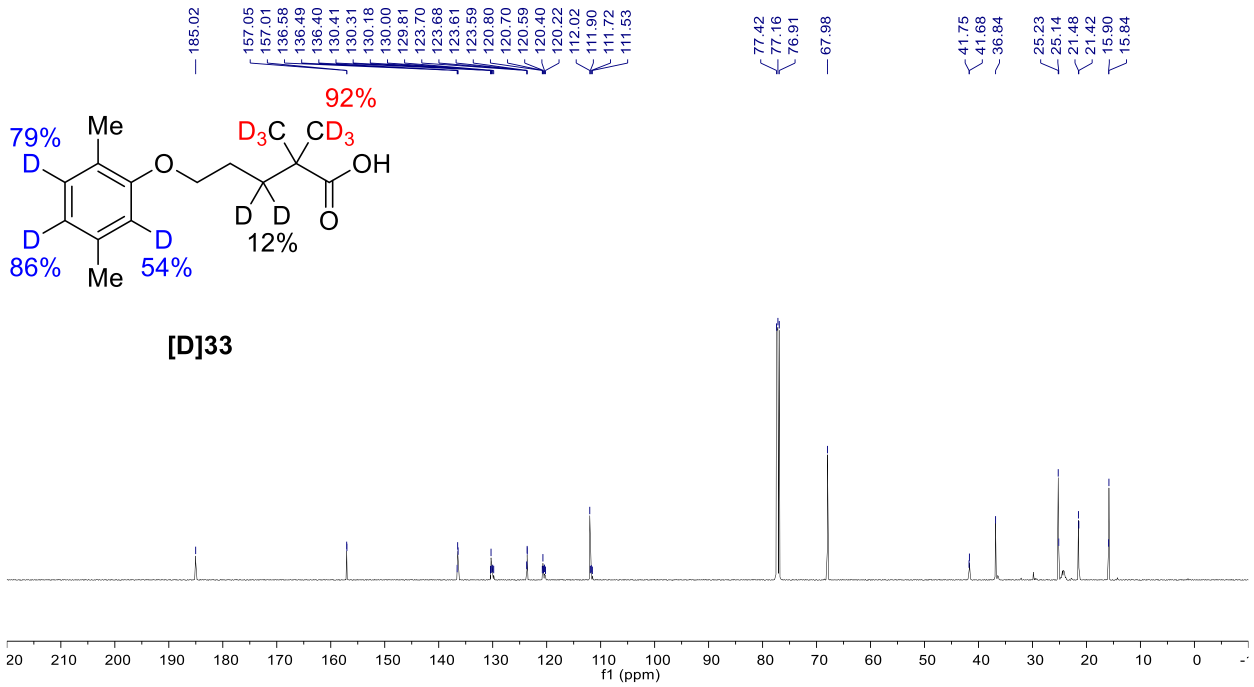

^{13}C NMR spectrum of compound **[D]33** (Procedure A, $CDCl_3$, 126 MHz)

2-(2-fluoro-[1,1'-biphenyl]-4-yl)propanoic acid (flurbiprofen, 34)

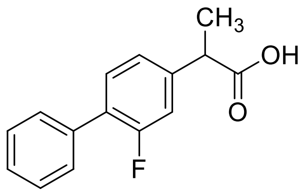

Following the general procedure A, the reaction was set up with 2-(2-fluoro-[1,1'-biphenyl]-4-yl)propanoic acid (48.9 mg, 0.20 mmol). Purification by flash column chromatography (hexanes/EtOAc/HCO_2H = 5:1:0.05) provided product **[D]34** as a yellow solid (49 mg, 96% yield).

Following the general procedure B, **[D]34** was provided as a yellow solid (43 mg, 84% yield).

Deuterium Incorporation

General procedure B: [LCMS (ESI)] calcd for $C_{15}D_{12}FO_2$ $[M-H]^-$ 9.87 D/molecule, [^{1}H NMR] 10.11 D/molecule.

NMR Data of the Starting Material

^{1}H NMR (500 MHz, $CDCl_3$) δ 7.55-7.51 (m, 2H), 7.46-7.39 (m, 3H), 7.39-7.35 (m, 1H), 7.19-7.13 (m, 2H), 3.80 (q, J = 7.2 Hz, 1H), 1.57 (d, J = 7.2 Hz, 3H).

NMR Data of the Product

General procedure A: ^{1}H NMR (500 MHz, $CDCl_3$) δ 7.58-7.52 (m, 0.97H, 34% D), 7.47-7.39 (m, 0.49H, 84% D), 7.37 (s, 0.12H, 88% D), 7.20-7.14 (m, 0.13H, 94% D), 3.82-3.77 (m, 0.74H, 26% D), 1.59-1.52 (m, 1.03H, 66% D).

General procedure B: ^{1}H NMR (500 MHz, $CDCl_3$) δ 7.55 (d, J = 14.2 Hz, 0.50H, 75% D), 7.47-7.39 (m, 0.23H, 92% D), 7.37 (d, J = 6.1 Hz, 0.07H, 93% D), 7.19-7.14 (m, 0.11H, 95% D), 3.82-3.76 (m, 0.56H, 44% D), 1.59-1.51 (m, 0.43H, 86% D); ^{13}C NMR (126 MHz, $CDCl_3$) δ 180.5, 160.8, 158.8, 140.9-140.8 (1C), 135.4-135.3 (1C), 130.9-130.4 (1C), 129.1-128.8 (2C), 128.6-127.9 (2C), 1276-127.1 (1C), 123.7-123.2 (1C), 115.6-115.0 (1C), 44.8-44.7 (1C), 18.1-17.0 (1C).

Mass Data

LabelChecker Results

Formula:	C15 H12 O2 F
Mass (monoisotopic):	243.08
Difference Value:	0.000431
Error Sum:	0.021
Error (%):	0.098

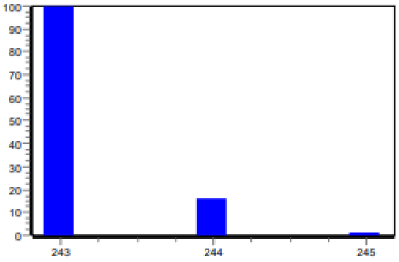

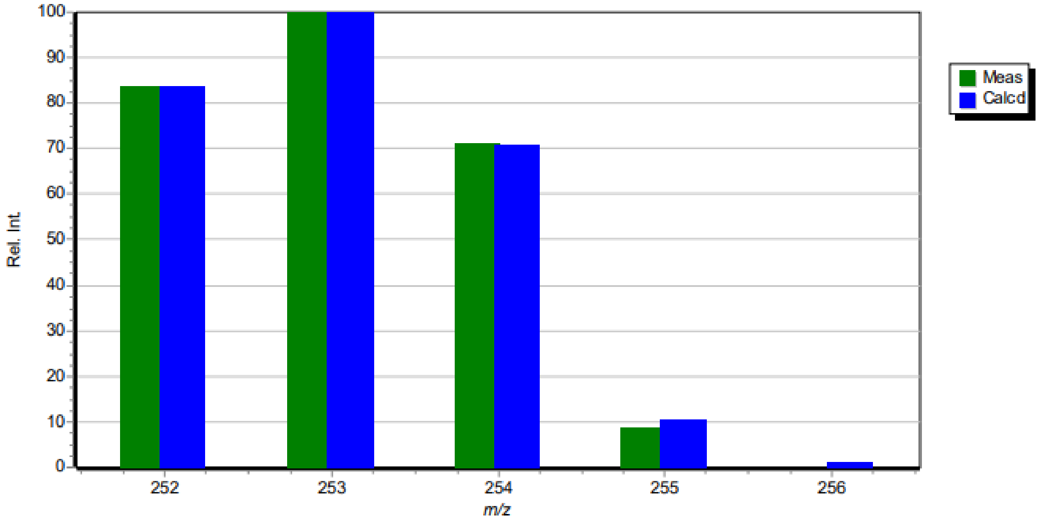

Deuterium: 0-fold (%): 0.00 0.00
Deuterium: 1-fold (%): 0.00 0.00
Deuterium: 2-fold (%): 0.01 0.01
Deuterium: 3-fold (%): 0.00 0.00
Deuterium: 4-fold (%): 0.00 0.00
Deuterium: 5-fold (%): 0.00 0.00
Deuterium: 6-fold (%): 0.00 0.00
Deuterium: 7-fold (%): 0.00 0.00
Deuterium: 8-fold (%): 0.00 0.00
Deuterium: 9-fold (%): 96.95 37.13
Deuterium: 10-fold (%): 100.00 38.30
Deuterium: 11-fold (%): 64.14 24.56
Deuterium: 12-fold (%): 0.00 0.00
Label Atom Sum: 9.87 (82.28%)

Isotope List used for fitting data:

m/z	intensity
252.14	1494155
253.15	1785442
254.15	1270651
255.16	156979

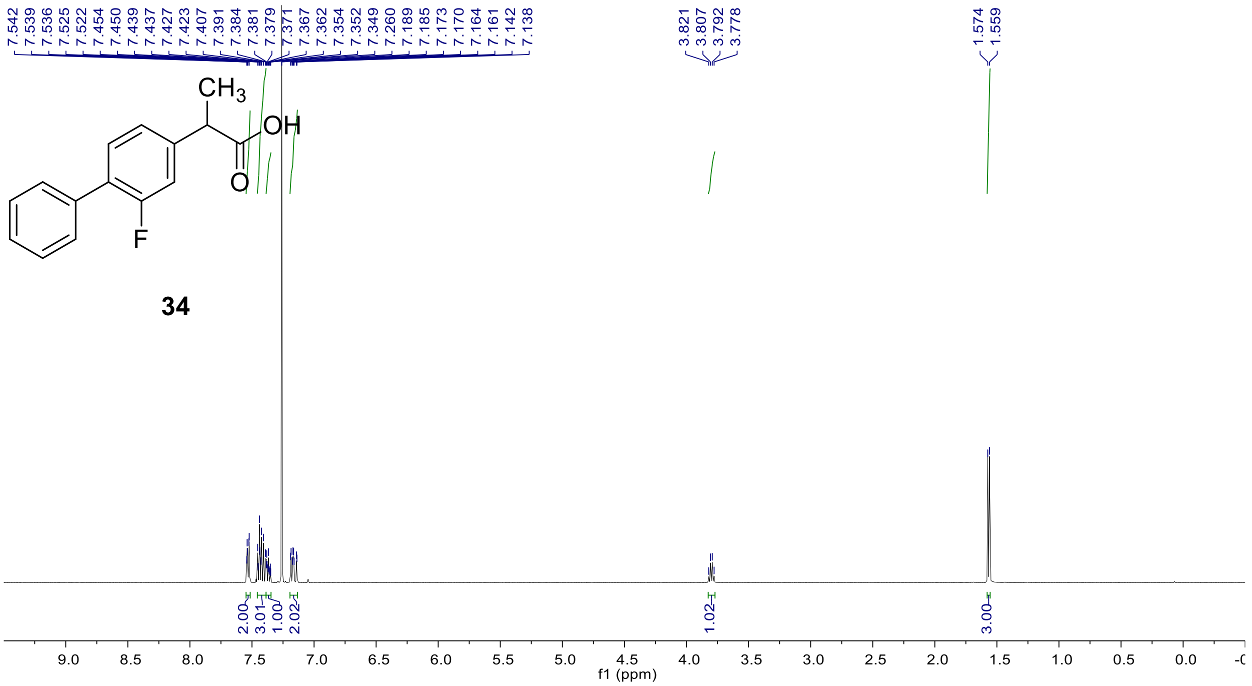

^{1}H NMR spectrum of compound **34** ($CDCl_3$, 500 MHz)

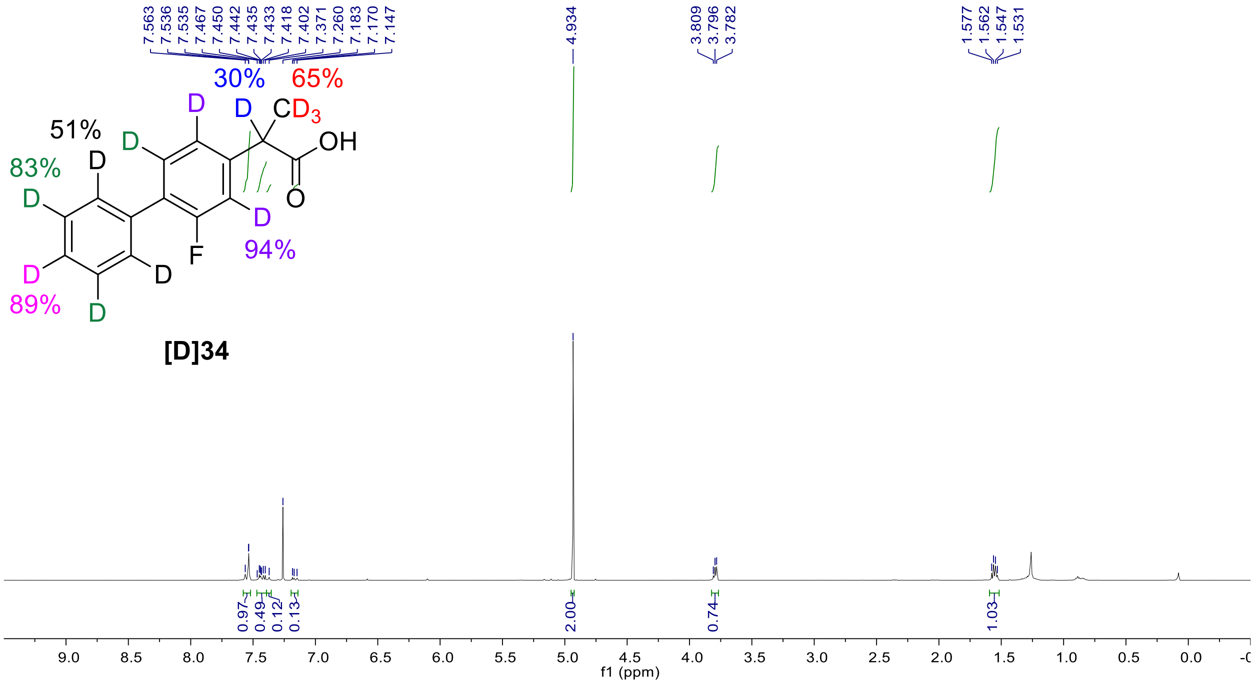

^{1}H NMR spectrum of compound **[D]34** (Procedure A, $CDCl_3$, 500 MHz)

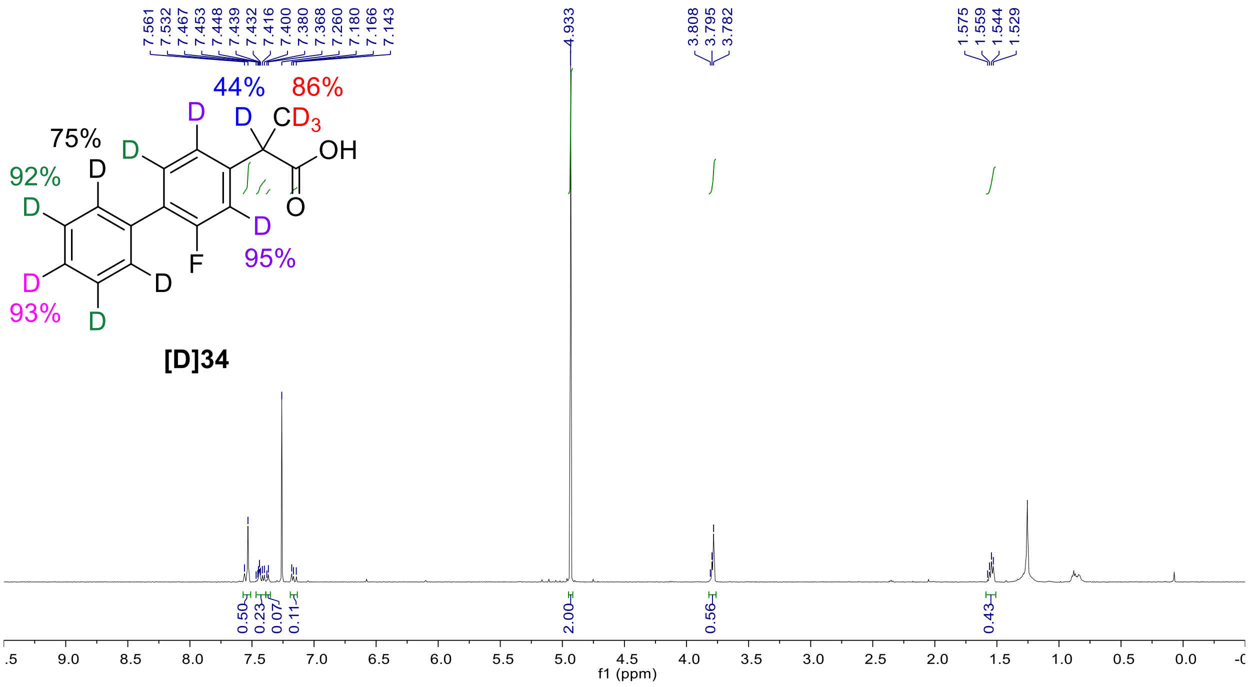

^{1}H NMR spectrum of compound **[D]34** (Procedure B, $CDCl_3$, 500 MHz)

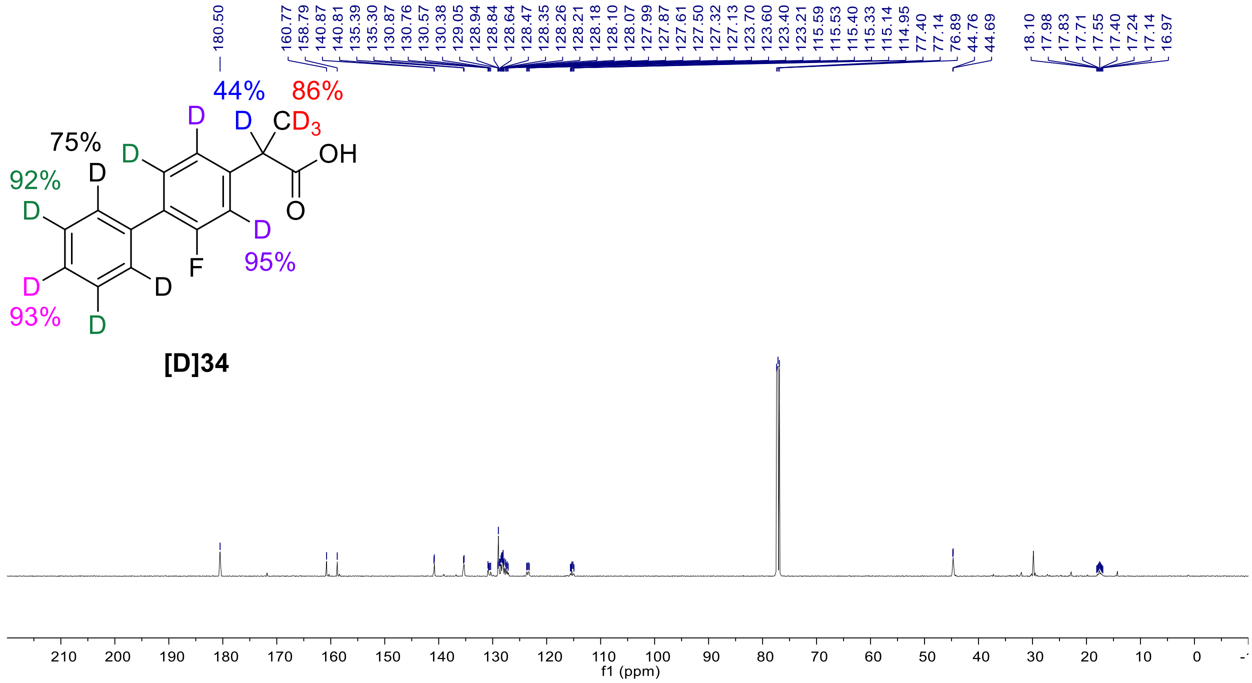

^{13}C NMR spectrum of compound **[D]34** (Procedure B, $CDCl_3$, 126 MHz)

2-(3-benzoylphenyl)propanoic acid (ketoprofen, 35)

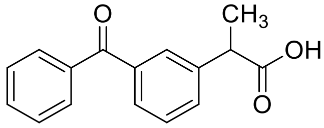

Following the general procedure A, the reaction was set up with 2-(3-benzoylphenyl)propanoic acid (50.8 mg, 0.20 mmol). Purification by flash column chromatography (hexanes/EtOAc/HCO_2H = 1:2:0.05) provided product **[D]35** as a brown liquid (50 mg, 94% yield).

Following the general procedure B, **[D]35** was provided as a yellow solid (43 mg, 80% yield).

Deuterium Incorporation

General procedure A: [LCMS (ESI)] calcd for $C_{16}HD_{13}O_3$ $[M]^+$ 11.57 D/molecule, [^{1}H NMR] 11.03 D/molecule.

NMR Data of the Starting Material

^{1}H NMR (500 MHz, $CDCl_3$) δ 7.83-7.76 (m, 3H), 7.71-7.67 (m, 1H), 7.62-7.55 (m, 2H), 7.51-7.43 (m, 3H), 3.83 (q, J = 7.2 Hz, 1H), 1.56 (d, J = 7.2 Hz, 3H).

NMR Data of the Product

General procedure A: ^{1}H NMR (500 MHz, $CDCl_3$) δ 7.87-7.77 (m, 0.37H, 88% D), 7.70-7.65 (m, 0.09H, 91% D), 7.60-7.55 (m, 0.16H, 92% D), 7.50-7.43 (m, 0.26H, 91% D), 3.88-3.79 (m, 0.49H, 51% D), 1.59-1.50 (m, 0.60H, 80% D); ^{13}C NMR (126 MHz, $CDCl_3$) δ 196.7, 180.2, 140.0, 137.9-137.8 (1C), 137.4-137.3 (1C), 132.5-132.2 (1C), 131.7-131.4 (1C), 130.1-129.6 (2C), 129.5, 129.3-129.0 (1C), 128.5, 128.3-127.7 (2C), 45.1, 17.7.

General procedure B: ^{1}H NMR (500 MHz, $CDCl_3$) δ 7.88-7.77 (m, 0.24H, 92% D), 7.71-7.65 (m, 0.06H, 94% D), 7.61-7.56 (m, 0.12H, 94% D), 7.50-7.43 (m, 0.20H, 93% D), 3.82 (s, 0.31H, 69% D), 1.53 (d, J = 7.8 Hz, 0.43H, 86% D).

Mass Data

LabelChecker Results

Formula: C16 H14 O3
Mass (monoisotopic): 254.09

Difference Value: 0.002399
Error Sum: 0.049
Error (%): 0.489

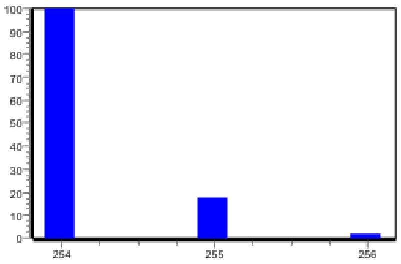

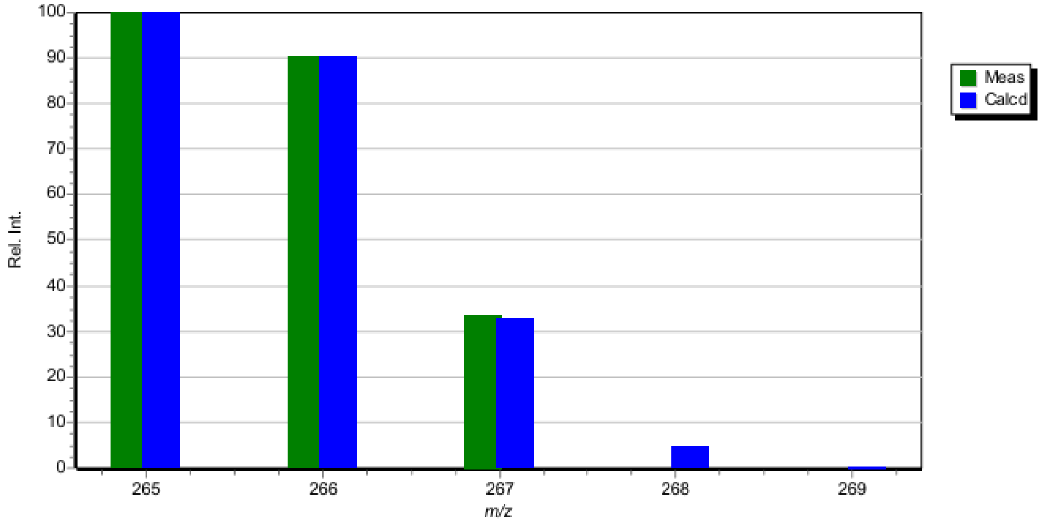

Deuterium: 0-fold (%): 0.00 0.00
Deuterium: 1-fold (%): 0.00 0.00
Deuterium: 2-fold (%): 0.00 0.00
Deuterium: 3-fold (%): 0.00 0.00
Deuterium: 4-fold (%): 0.00 0.00
Deuterium: 5-fold (%): 0.00 0.00
Deuterium: 6-fold (%): 0.00 0.00
Deuterium: 7-fold (%): 0.00 0.00
Deuterium: 8-fold (%): 0.00 0.00
Deuterium: 9-fold (%): 0.00 0.00
Deuterium: 10-fold (%): 0.00 0.00
Deuterium: 11-fold (%): 100.00 52.41
Deuterium: 12-fold (%): 72.88 38.19
Deuterium: 13-fold (%): 17.94 9.40
Label Atom Sum: 11.57 (82.64%)

Isotope List used for fitting data:

m/z intensity
265.14 152313
266.13 137532
267.12 51098

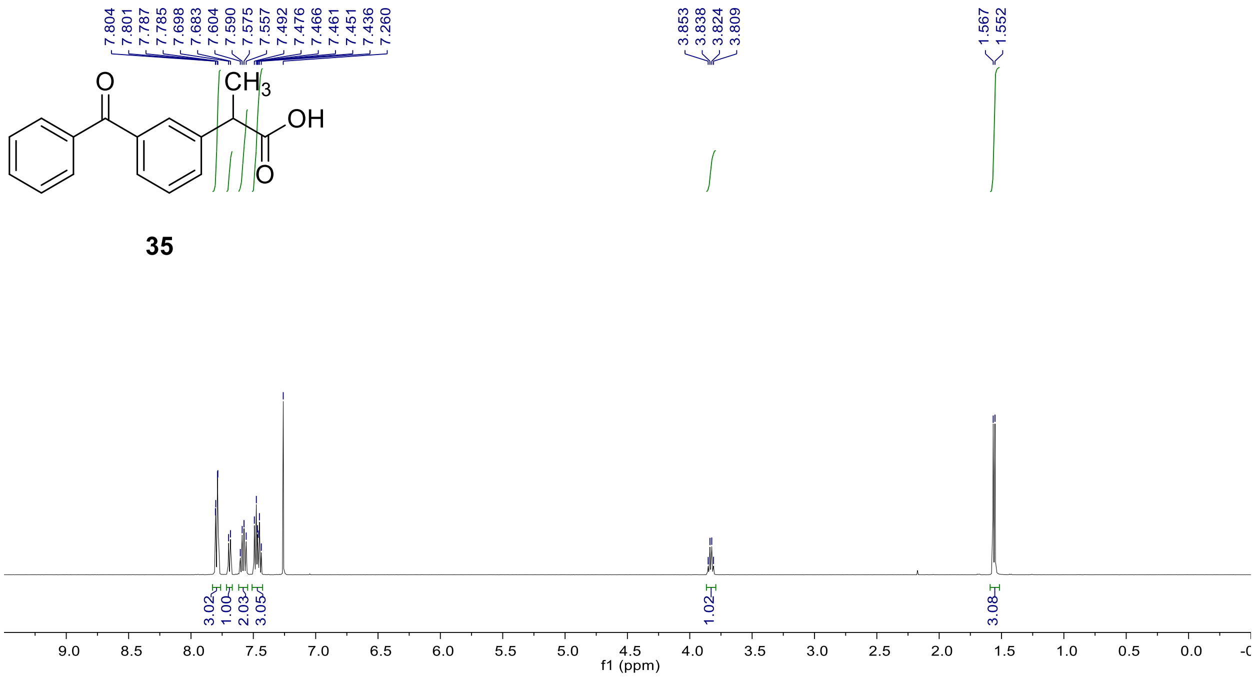

^{1}H NMR spectrum of compound **35** ($CDCl_3$, 500 MHz)

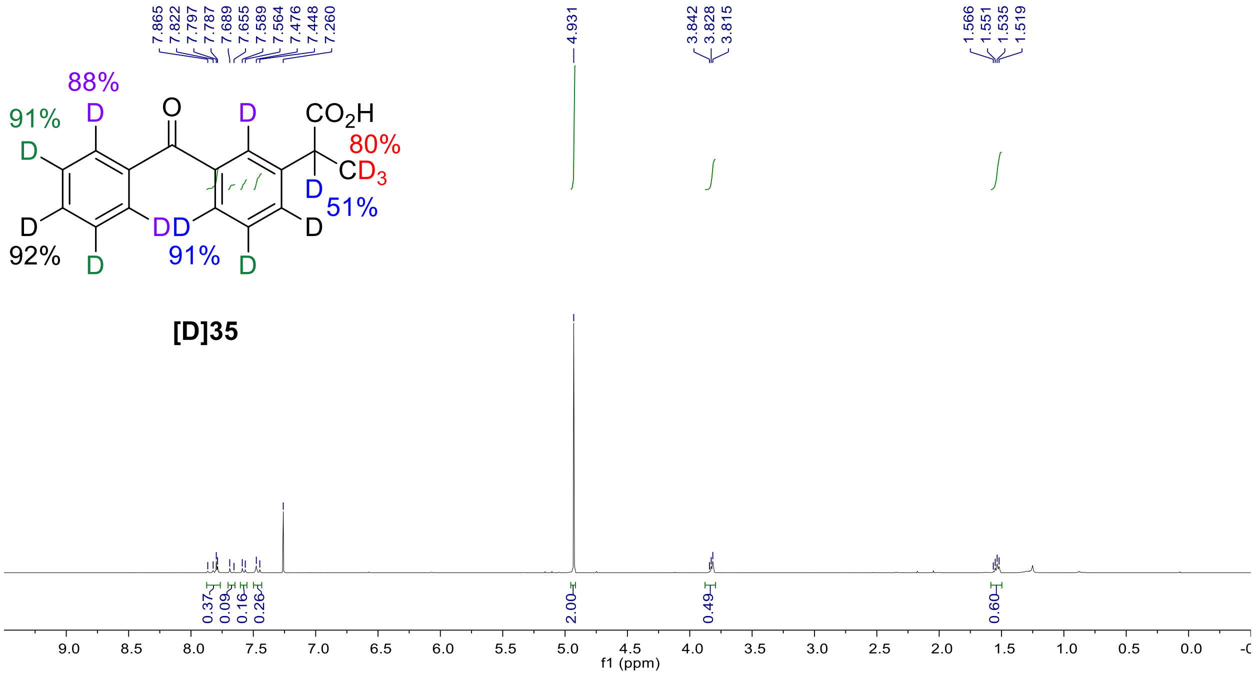

^{1}H NMR spectrum of compound **[D]35** (Procedure A, $CDCl_3$, 500 MHz)

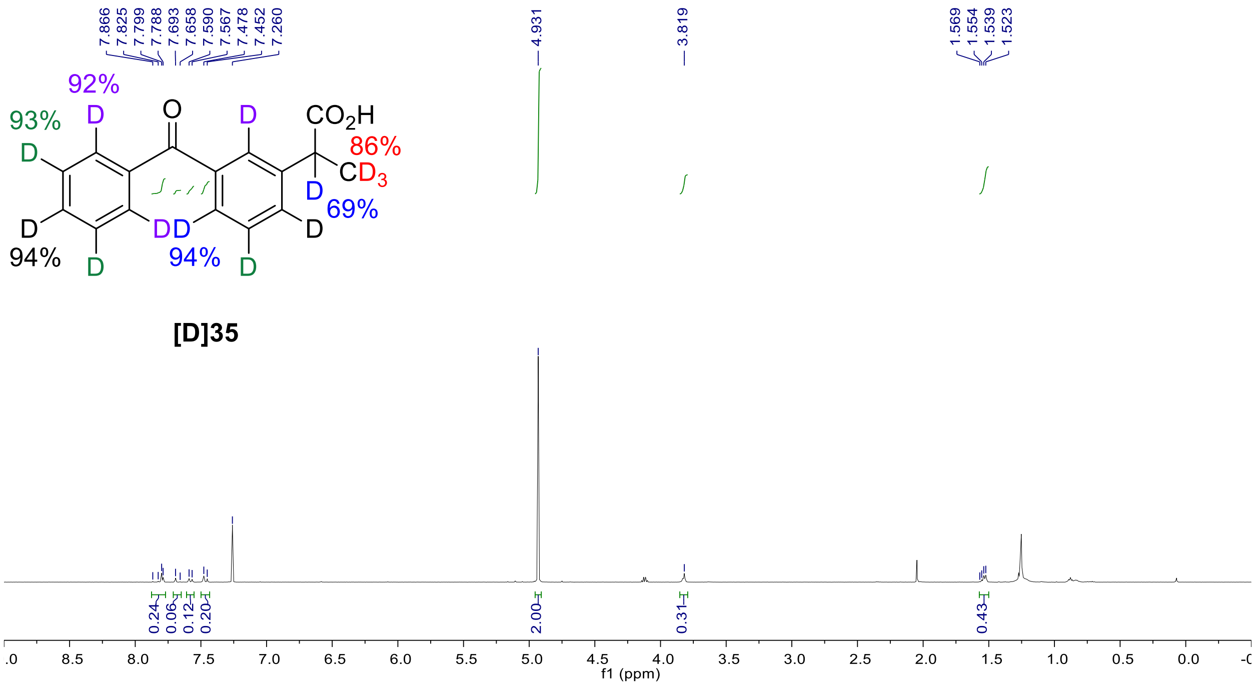

^{1}H NMR spectrum of compound **[D]35** (Procedure B, $CDCl_3$, 500 MHz)

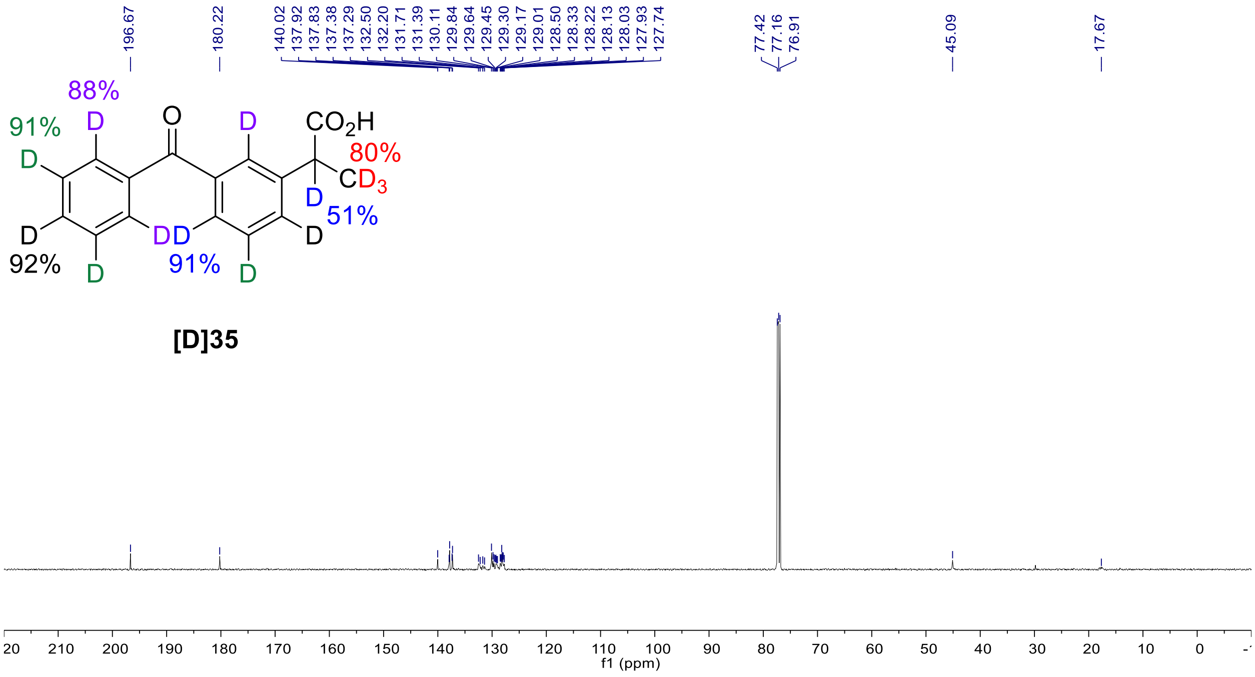

^{13}C NMR spectrum of compound **[D]35** (Procedure A, $CDCl_3$, 126 MHz)

2-(11-oxo-6,11-dihydrodibenzo[*b,e*]oxepin-2-yl)acetic acid (isoxepac, 36)

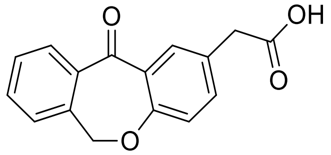

Following the general procedure B, the reaction was set up with 2-(11-oxo-6,11-dihydrodibenzo[*b,e*]oxepin-2-yl)acetic acid (53.7 mg, 0.20 mmol). Purification by flash column chromatography (hexanes/EtOAc/HCO_2H = 2:1:0.03) provided product **[D]36** as a yellow solid (55 mg, 99% yield).

Deuterium Incorporation

General procedure B: [LCMS (ESI)] calcd for $C_{16}H_4D_9O_4$ $[M+H]^+$ 8.57 D/molecule, [^{1}H NMR] 8.52 D/molecule.

NMR Data of the Starting Material

^{1}H NMR (500 MHz, CD_2Cl_2) δ 8.10 (d, *J* = 2.4 Hz, 1H), 7.86 (dd, *J* = 7.7, 1.4 Hz, 1H), 7.59 (td, *J* = 7.5, 1.4 Hz, 1H), 7.48 (td, *J* = 7.6, 1.3 Hz, 1H), 7.43 (dd, *J* = 8.4, 2.4 Hz, 1H), 7.41-7.38 (m, 1H), 7.04 (d, *J* = 8.4 Hz, 1H), 5.20 (s, 2H), 3.70 (s, 2H).

NMR Data of the Product

General procedure B: ^{1}H NMR (500 MHz, CD_2Cl_2) δ 8.08 (s, 0.05H, 95% D), 7.85 (s, 0.06H, 94% D), 7.57 (s, 0.05H, 95% D), 7.47 (s, 0.05H, 95% D), 7.41 (s, 0.05H, 95% D), 7.38 (s, 0.06H, 94% D), 7.02 (s, 0.06H, 94% D), 5.18 (s, 2H), 3.66 (s, 0.11H, 95% D); ^{13}C NMR (126 MHz, CD_2Cl_2) δ 190.6, 177.4, 160.5, 140.3, 136.4-135.9 (1C), 135.8, 132.6-132.4 (1C), 132.3-131.9 (1C), 129.3-128.5 (2C), 127.8-127.4 (1C), 127.3, 125.1, 120.9-120.5 (1C), 73.6, 39.4.

Mass Data

LabelChecker Results

Formula: C16 H13 O4
Mass (monoisotopic): 269.08

Difference Value: 0.000567
Error Sum: 0.024
Error (%): 0.188

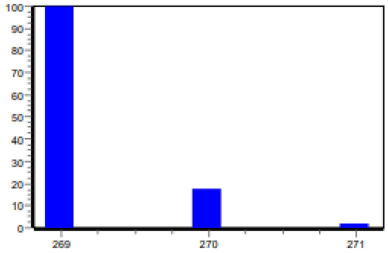

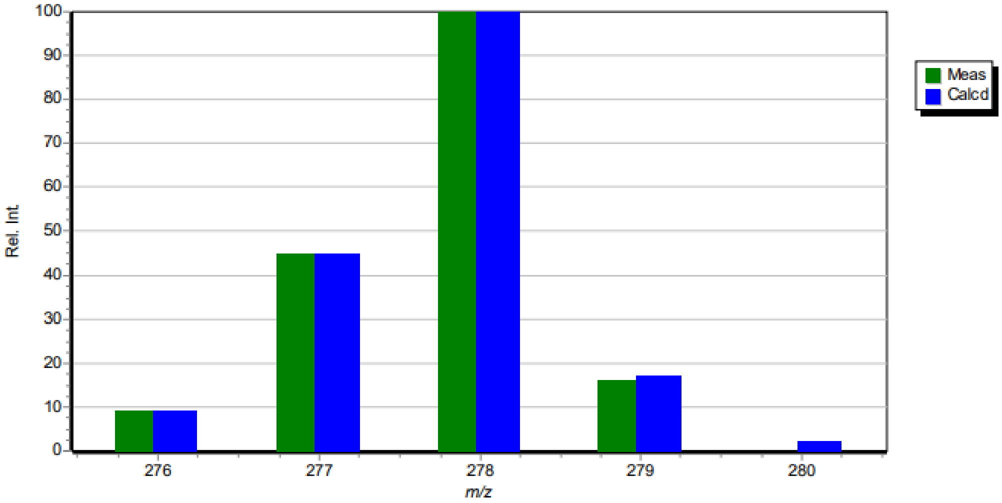

Deuterium: 0-fold (%): 0.00 0.00
Deuterium: 1-fold (%): 0.00 0.00
Deuterium: 2-fold (%): 0.00 0.00
Deuterium: 3-fold (%): 0.00 0.00
Deuterium: 4-fold (%): 0.00 0.00
Deuterium: 5-fold (%): 0.21 0.14
Deuterium: 6-fold (%): 0.00 0.00
Deuterium: 7-fold (%): 10.02 6.36
Deuterium: 8-fold (%): 47.21 29.98
Deuterium: 9-fold (%): 100.00 63.52
Label Atom Sum: 8.57 (65.90%)

Isotope List used for fitting data:

m/z	intensity
276.12	2964155
277.13	14482921
278.14	32103828
279.14	5207237

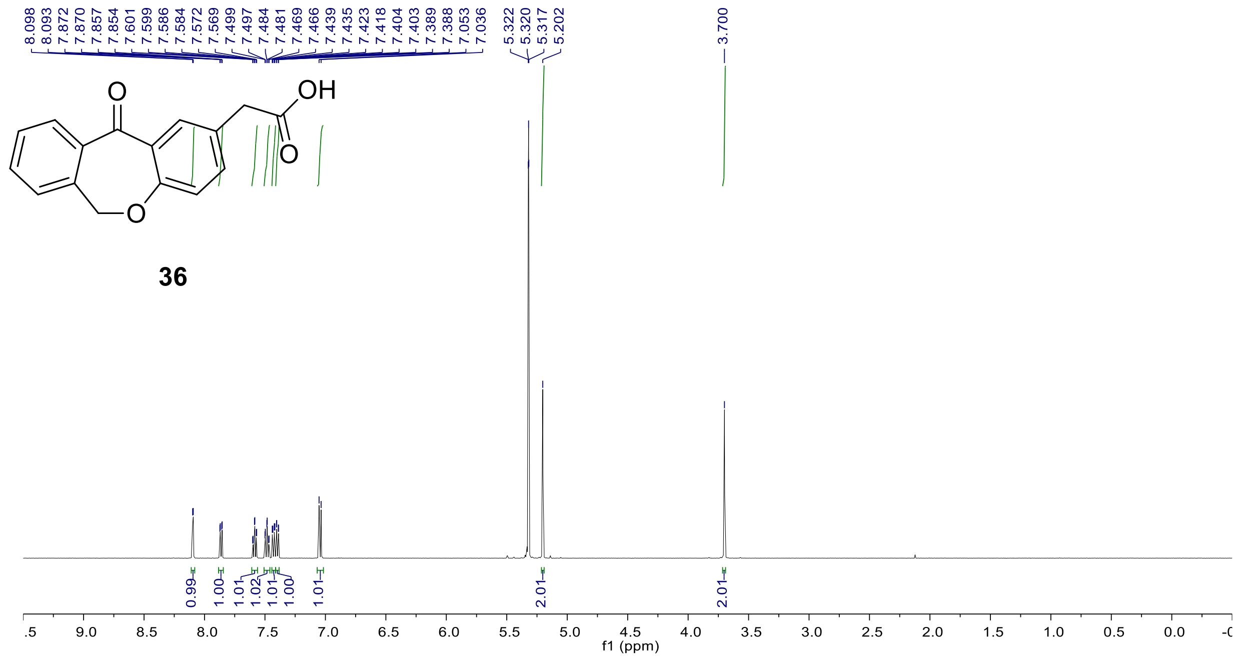

^{1}H NMR spectrum of compound **36** (CD_2Cl_2, 500 MHz)

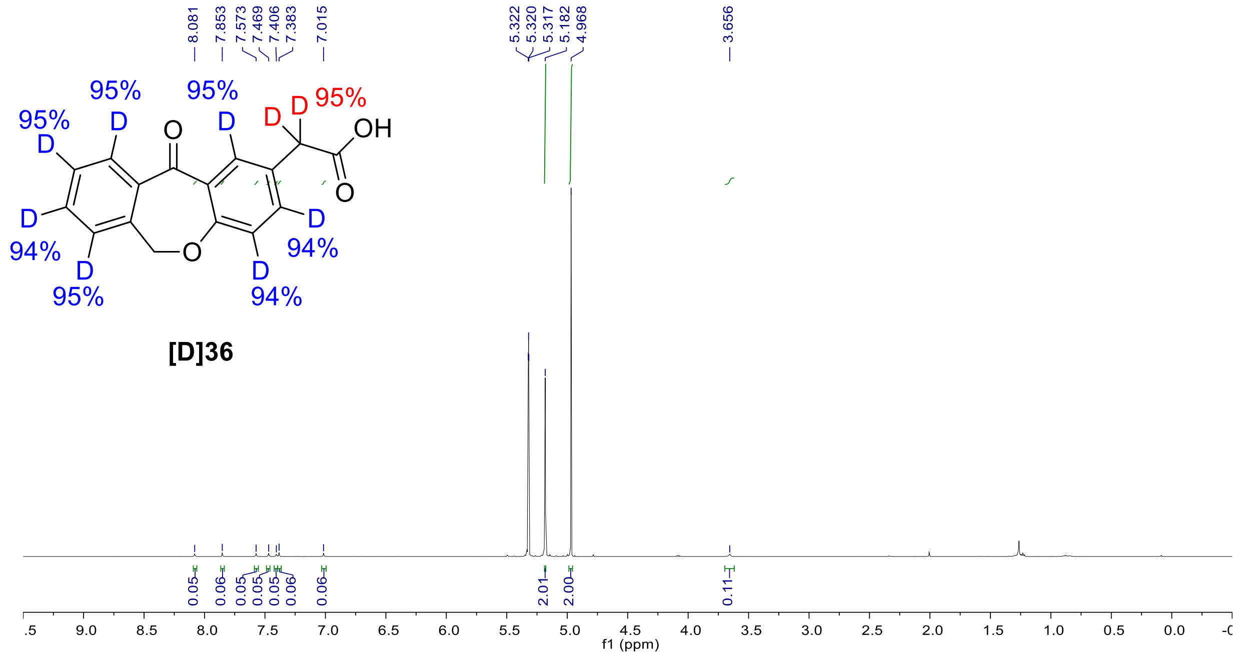

^{1}H NMR spectrum of compound **[D]36** (Procedure B, CD_2Cl_2, 500 MHz)

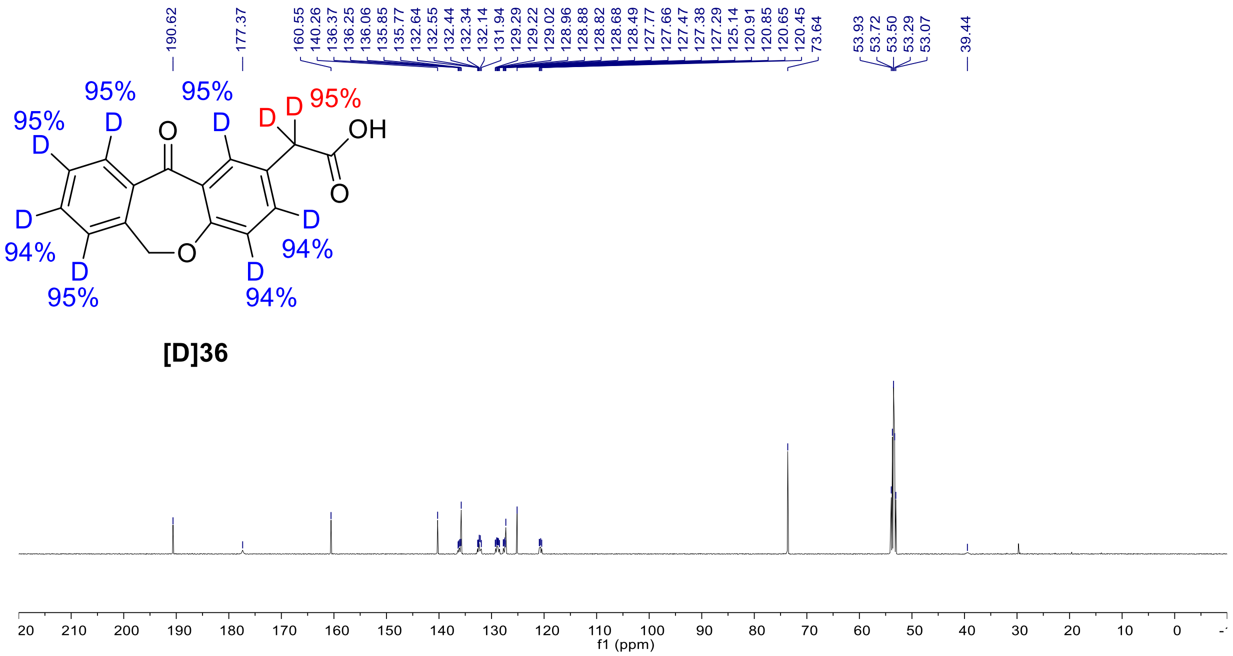

^{13}C NMR spectrum of compound **[D]36** (Procedure B, CD_2Cl_2, 126 MHz)

4-oxo-4-(4-phenylphenyl)butanoic acid (fenbufen, 37)

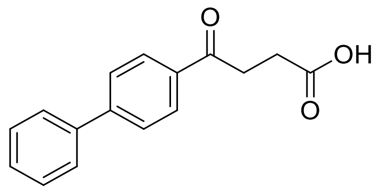

Following the general procedure B, the reaction was set up with 4-oxo-4-(4-phenylphenyl)butanoic acid (50.9 mg, 0.20 mmol). Purification by flash column chromatography (hexanes/EtOAc = 10:1) provided product **[D]37** as a yellow solid (35 mg, 65% yield).

Deuterium Incorporation

General procedure B: [LCMS (ESI)] calcd for $C_{16}HD_{13}O_3Na$ $[M+Na]^+$ 11.01 D/molecule, [^1H NMR] 11.42 D/molecule.

NMR Data of the Starting Material

^1H NMR (500 MHz, $CDCl_3$) δ 8.08 – 8.05 (m, 2H), 7.73-7.67 (m, 2H), 7.66-7.61 (m, 2H), 7.50-7.45 (m, 2H), 7.43-7.39 (m, 1H), 3.36 (t, J = 6.6 Hz, 2H), 2.85 (t, J = 6.6 Hz, 2H).

NMR Data of the Product

General procedure B: ^1H NMR (500 MHz, $CDCl_3$) δ 8.06 (s, 0.13H, 94% D), 7.70 (s, 0.21H, 90% D), 7.63 (s, 0.32H, 84% D), 7.49-7.46 (m, 0.15H, 93% D), 7.42-7.38 (m, 0.06H, 94% D), 3.33 (s, 0.09H, 96% D), 2.83 (d, J = 9.2 Hz, 0.66H, 67% D); ^{13}C NMR (126 MHz, $CDCl_3$) 197.7, 178.2, 146.0, 139.7, 135.1, 128.9-128.2 (5C), 127.3-126.8 (4C), 29.9, 28.0.

Mass Data

LabelChecker Results

Formula: C16 H14 O3 Na
Mass (monoisotopic): 277.08

Difference Value: 0.000040
Error Sum: 0.006
Error (%): 0.035

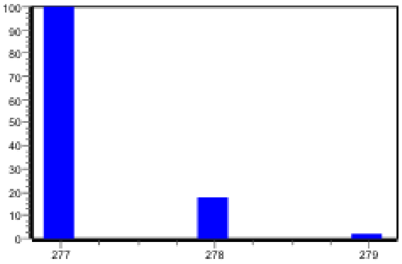

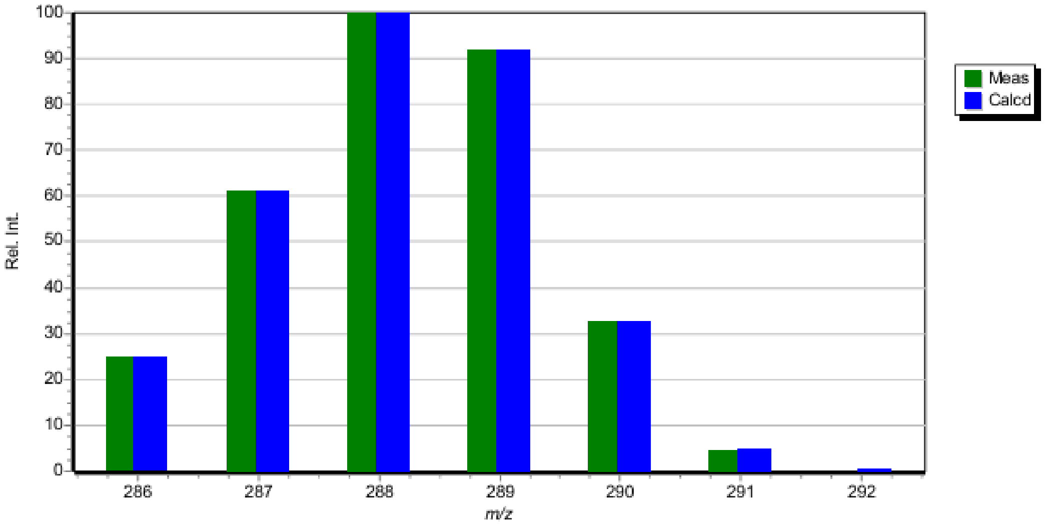

Deuterium: 0-fold (%): 0.00 0.00
Deuterium: 1-fold (%): 0.00 0.00
Deuterium: 2-fold (%): 0.00 0.00
Deuterium: 3-fold (%): 0.00 0.00
Deuterium: 4-fold (%): 0.00 0.00
Deuterium: 5-fold (%): 0.00 0.00
Deuterium: 6-fold (%): 0.00 0.00
Deuterium: 7-fold (%): 0.00 0.00
Deuterium: 8-fold (%): 0.00 0.00
Deuterium: 9-fold (%): 27.60 9.38
Deuterium: 10-fold (%): 63.59 21.60
Deuterium: 11-fold (%): 100.00 33.96
Deuterium: 12-fold (%): 83.62 28.40
Deuterium: 13-fold (%): 19.61 6.66
Deuterium: 14-fold (%): 0.00 0.00
Label Atom Sum: 11.01 (78.67%)

Isotope List used for fitting data:

m/z	intensity
286.14	2423652
287.15	6006945
288.15	9806750
289.16	8995272
290.16	3201181
291.17	430895

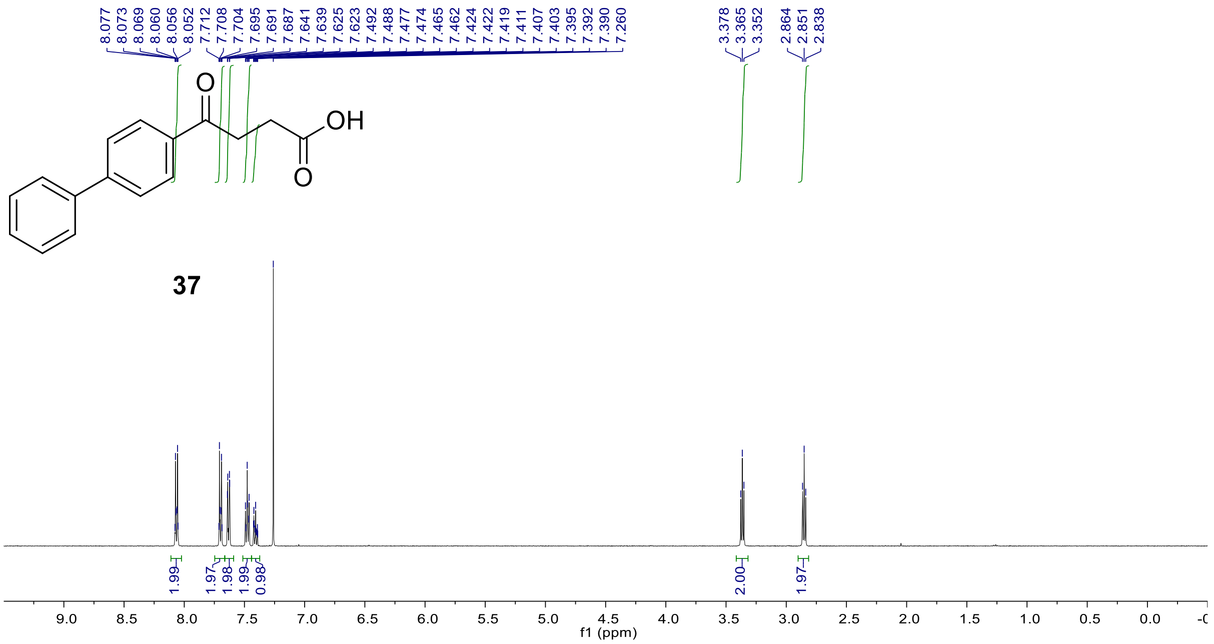

^{1}H NMR spectrum of compound **37** ($CDCl_3$, 500 MHz)

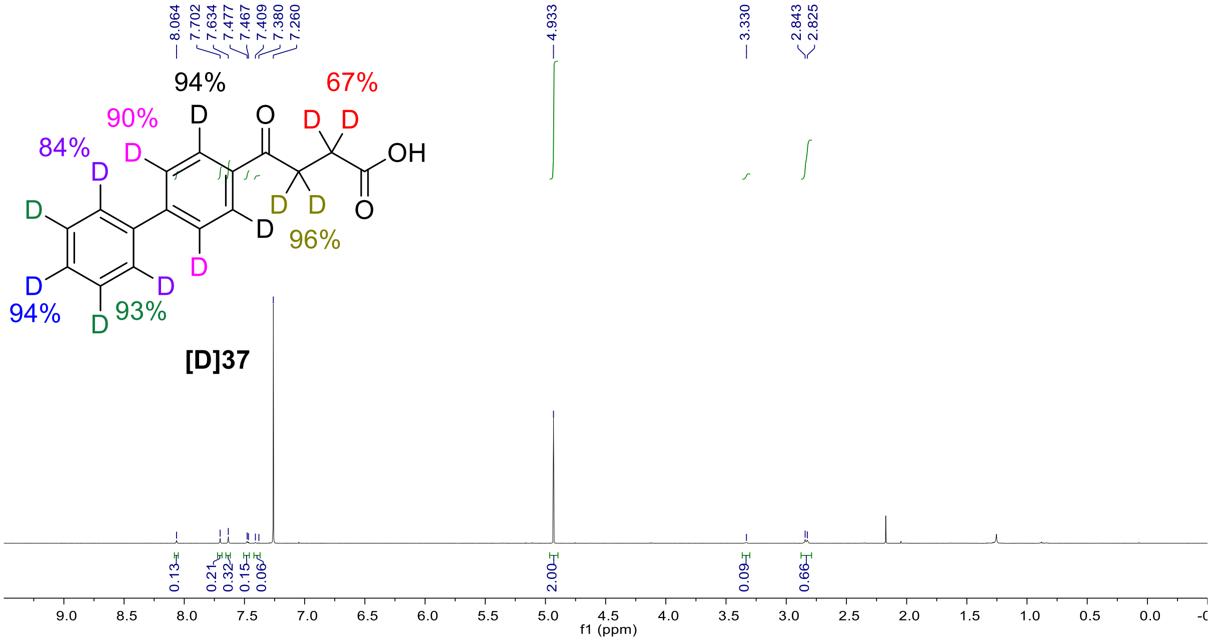

^{1}H NMR spectrum of compound **[D]37** (Procedure B, $CDCl_3$, 500 MHz)

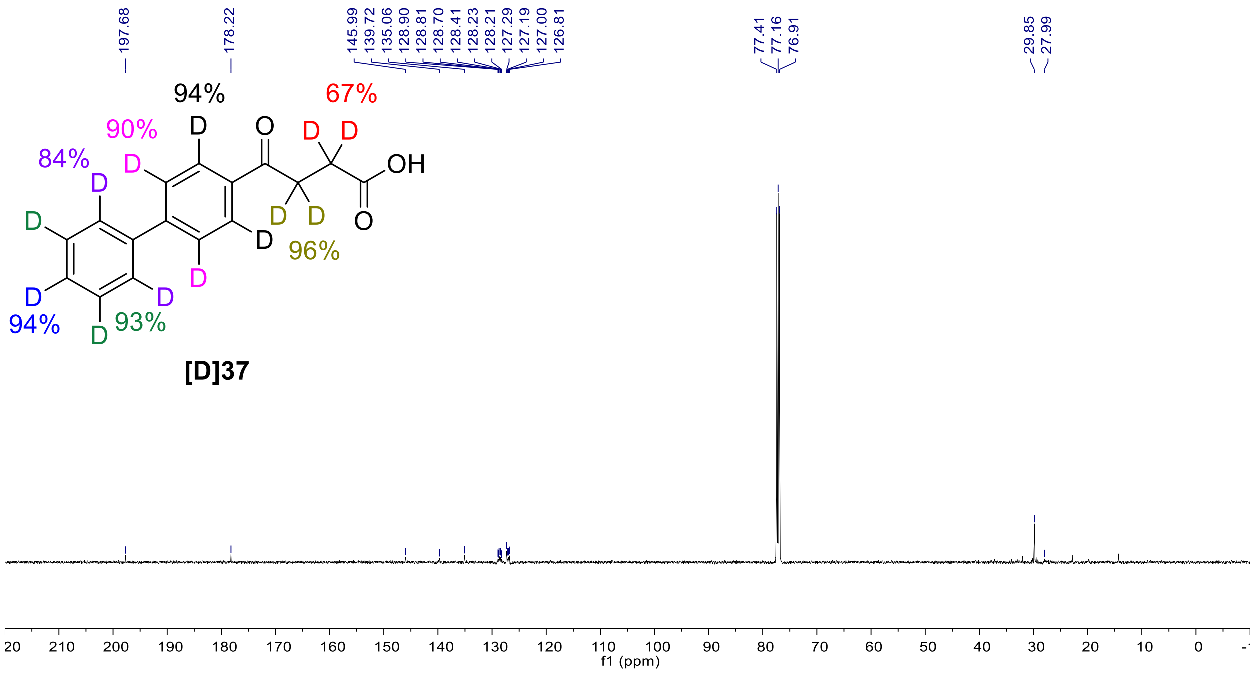

^{13}C NMR spectrum of compound **[D]37** (Procedure B, $CDCl_3$, 126 MHz)

benzamide (38a)

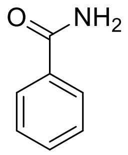

Following the general procedure B, the reaction was set up with benzamide (34.6 mg, 0.20 mmol). Purification by flash column chromatography (hexanes/EtOAc = 1:2) provided product **[D]38a** as a white solid (20 mg, 79% yield).

Deuterium Incorporation

General procedure B: [LCMS (ESI)] calcd for $C_7H_3D_5NO$ $[M+H]^+$ 4.80 D/molecule, [^{1}H NMR] 4.85 D/molecule.

NMR Data of the Starting Material

^{1}H NMR (500 MHz, $CDCl_3$) δ 7.86-7.79 (m, 2H), 7.57-7.51 (m, 1H), 7.46 (t, J = 7.6 Hz, 2H).

NMR Data of the Product

General procedure B: ^{1}H NMR (500 MHz, $CDCl_3$) δ 7.82 (s, 0.07H, 97% D), 7.54 (s, 0.03H, 97% D), 7.46 (s, 0.07H, 97% D); ^{13}C NMR (126 MHz, $CDCl_3$) δ 169.8, 133.3, 131.9-131.4 (2C), 128.5-128.0 (1C), 127.3-126.9 (2C).

Mass Data

LabelChecker Results

Formula: C7 H8 N O
Mass (monoisotopic): 122.06

Difference Value: 0.000024
Error Sum: 0.005
Error (%): 0.098

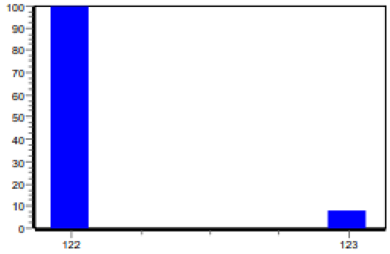

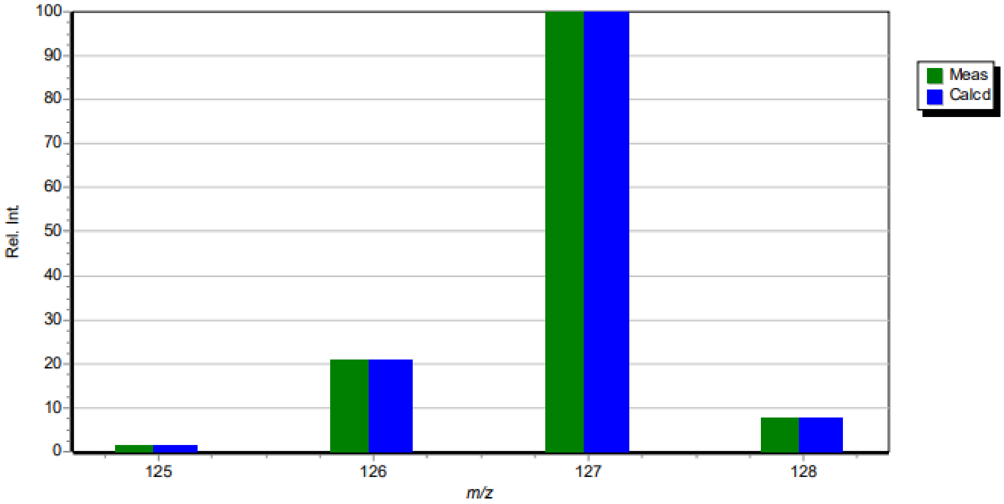

Deuterium: 0-fold (%): 0.00 0.00
Deuterium: 1-fold (%): 0.00 0.00
Deuterium: 2-fold (%): 0.00 0.00
Deuterium: 3-fold (%): 1.73 1.41
Deuterium: 4-fold (%): 21.32 17.32
Deuterium: 5-fold (%): 100.00 81.27
Label Atom Sum: 4.80 (59.98%)

Isotope List used for fitting data:

m/z	intensity
125.08	1430615
126.09	17727826
127.09	84053664
128.09	6609318

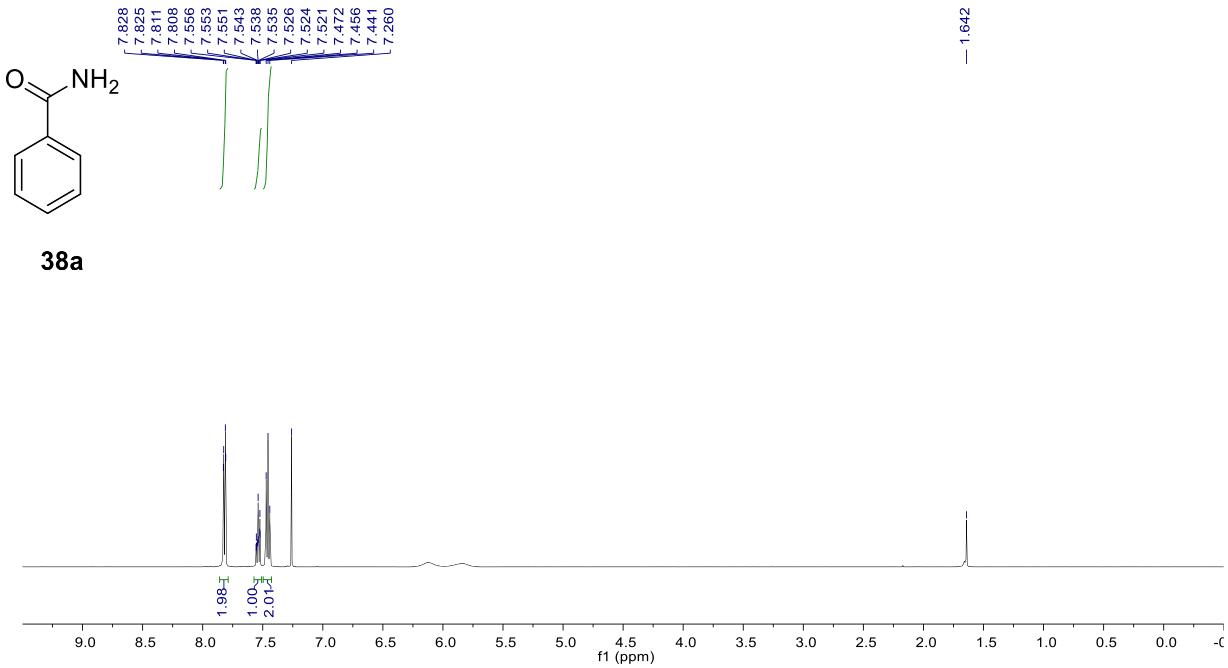

^{1}H NMR spectrum of compound **38a** ($CDCl_3$, 500 MHz)

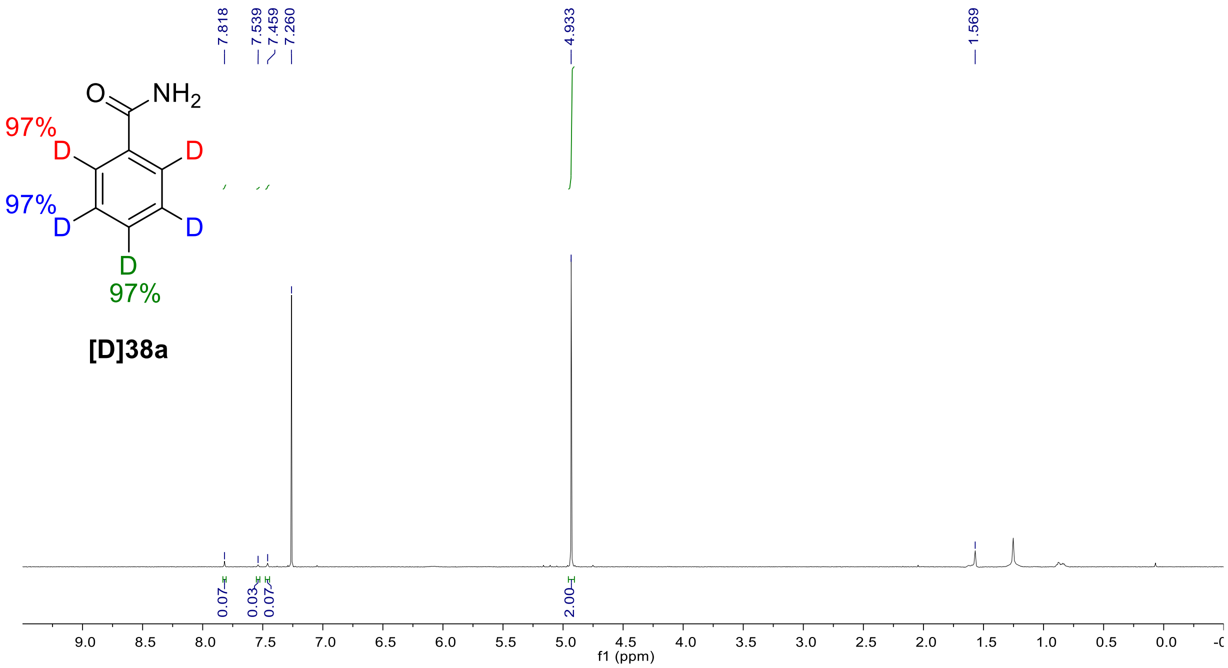

^{1}H NMR spectrum of compound **[D]38a** (Procedure B, $CDCl_3$, 500 MHz)

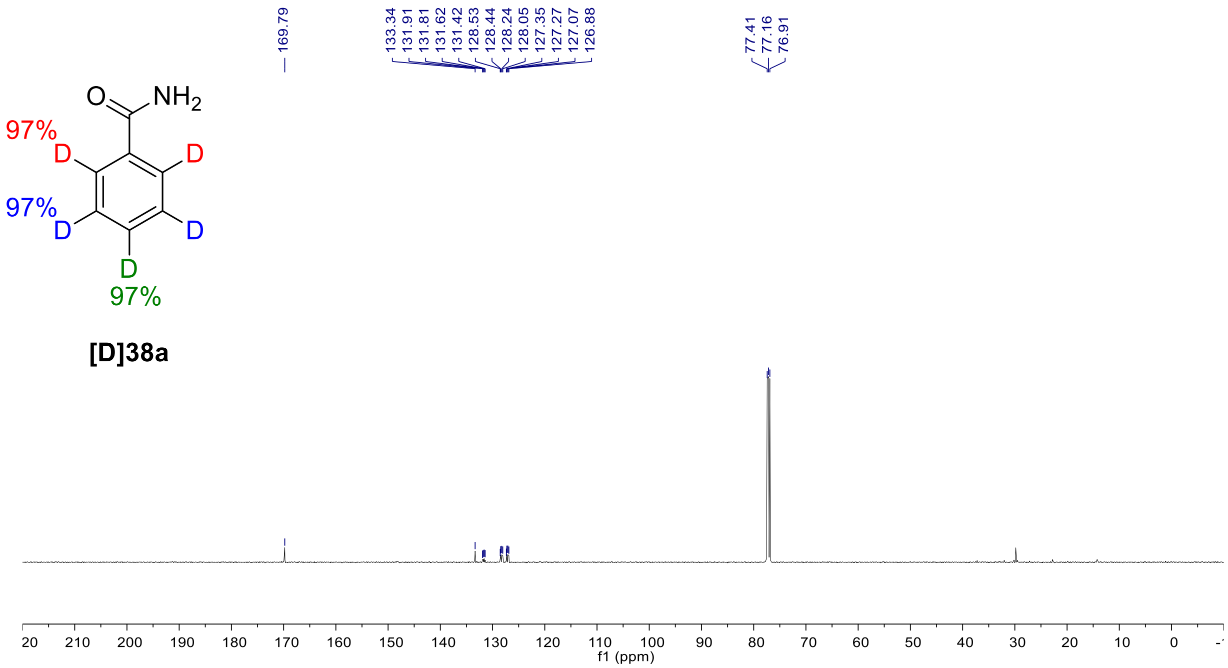

^{13}C NMR spectrum of compound **[D]38a** (Procedure B, $CDCl_3$, 126 MHz)

2-methylbenzamide (38b)

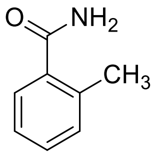

Following the general procedure B, the reaction was set up with 2-methylbenzamide (27.0 mg, 0.20 mmol). Purification by flash column chromatography (hexanes/EtOAc = 1:1) provided product **[D]38b** as a white solid (27 mg, 95% yield).

Deuterium Incorporation

General procedure B: [LCMS (ESI)] calcd for $C_8H_3D_7NO$ $[M+H]^+$ 5.68 D/molecule, [^{1}H NMR] 5.88 D/molecule.

NMR Data of the Starting Material

^{1}H NMR (500 MHz, CD_2Cl_2) δ 7.44 (dd, J = 7.5, 1.5 Hz, 1H), 7.34 (td, J = 7.5, 1.4 Hz, 1H), 7.26-7.20 (m, 2H), 2.46 (s, 3H).

NMR Data of the Product

General procedure B: ^{1}H NMR (500 MHz, CD_2Cl_2) δ 7.45 (s, 0.04H, 96% D), 7.34 (s, 0.04H, 96% D), 7.23 (d, J = 14.2 Hz, 0.08H, 96% D), 2.47-2.41 (m, 0.95H, 68% D); ^{13}C NMR (126 MHz, CD_2Cl_2) δ 171.9, 136.2, 135.4, 131.0-130.5 (1C), 129.9-129.5 (1C), 126.8-126.4 (1C), 125.5-125.0 (1C), 19.7-18.8 (1C).

Mass Data

LabelChecker Results

Formula: C8 H10 N O
Mass (monoisotopic): 136.08

Difference Value: 0.003007
Error Sum: 0.055
Error (%): 0.556

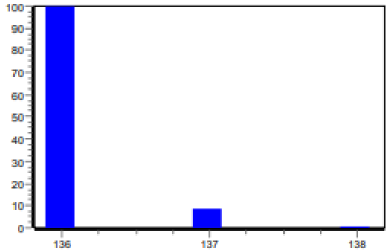

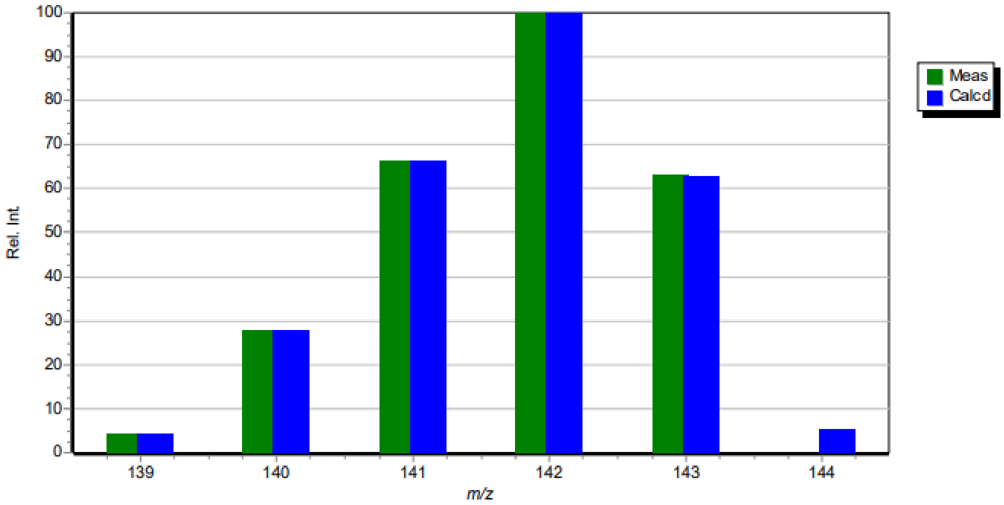

Deuterium: 0-fold (%): 0.02 0.01
Deuterium: 1-fold (%): 0.00 0.00
Deuterium: 2-fold (%): 0.13 0.05
Deuterium: 3-fold (%): 4.58 1.77
Deuterium: 4-fold (%): 29.34 11.32
Deuterium: 5-fold (%): 67.93 26.20
Deuterium: 6-fold (%): 100.00 38.57
Deuterium: 7-fold (%): 57.24 22.08
Label Atom Sum: 5.68 (56.77%)

Isotope List used for fitting data:

m/z	intensity
138.09	266605
139.09	6432719
140.10	41657912
141.11	98976376
142.11	148960224
143.12	94246584

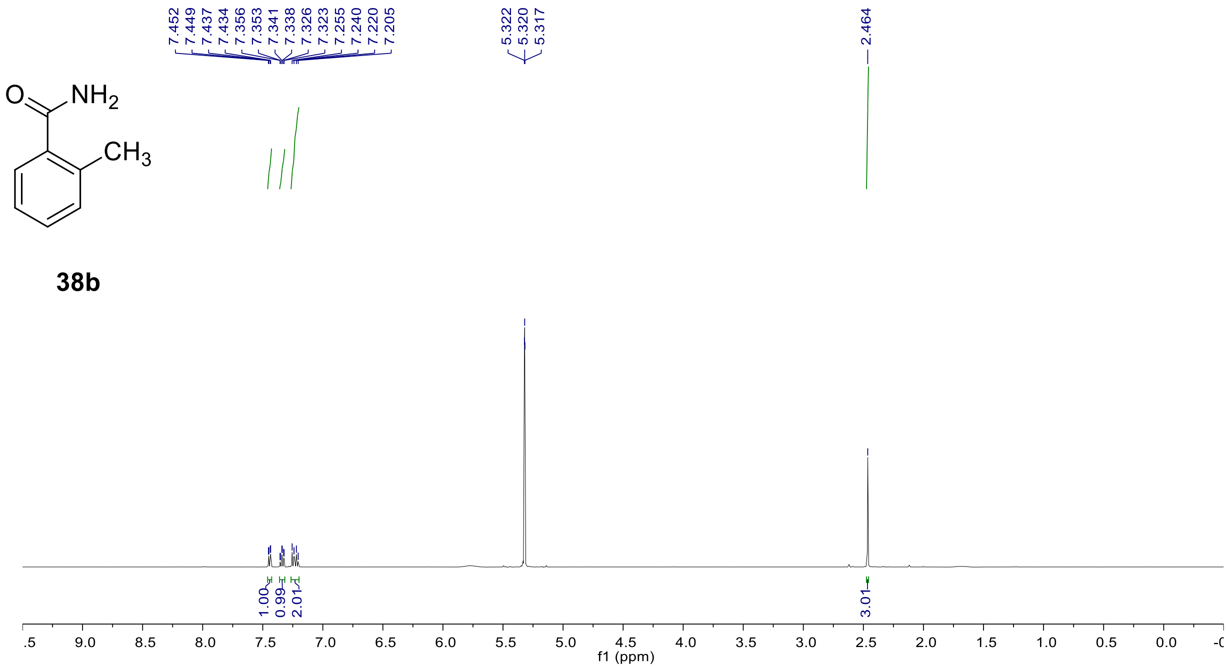

^{1}H NMR spectrum of compound **38b** (CD_2Cl_2, 500 MHz)

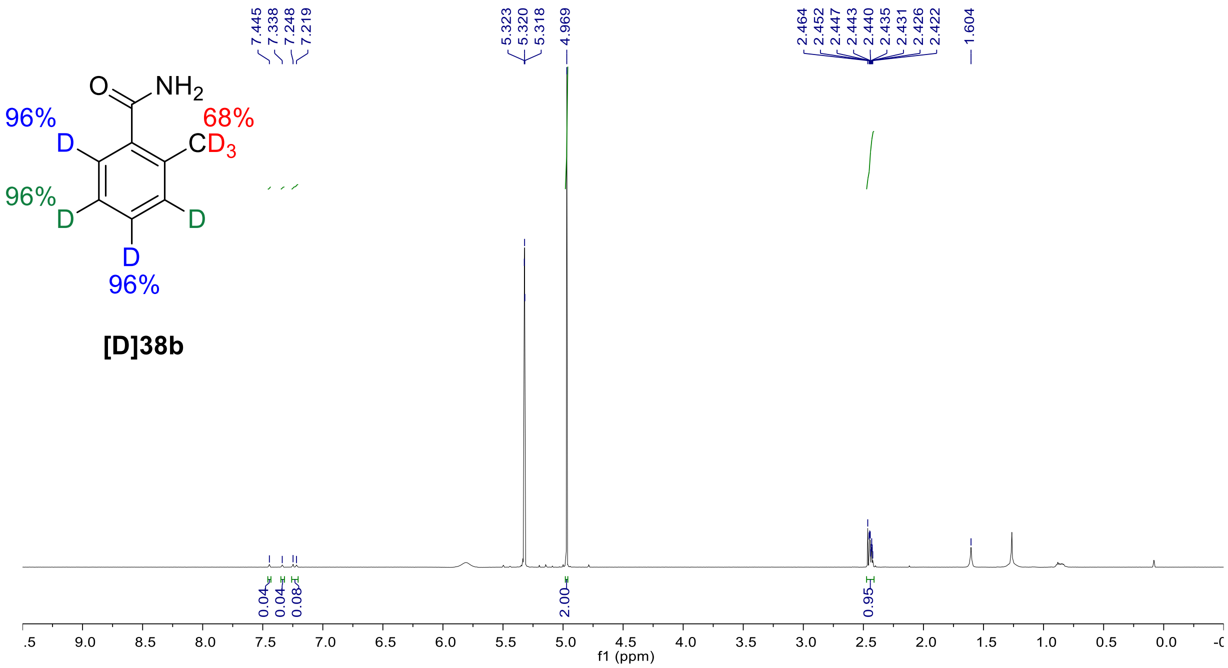

^{1}H NMR spectrum of compound **[D]38b** (Procedure B, CD_2Cl_2, 500 MHz)

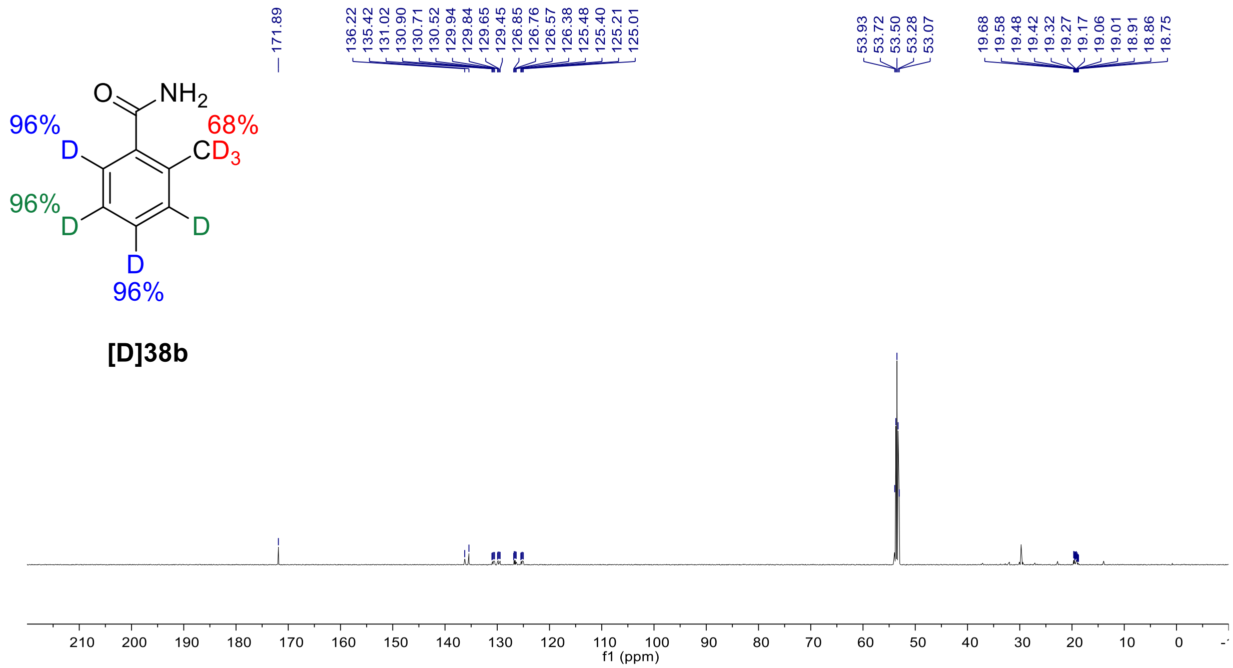

^{13}C NMR spectrum of compound **[D]38b** (Procedure B, CD_2Cl_2, 126 MHz)

2-phenylbenzoxazole (39a)

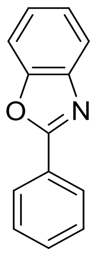

Following the general procedure A, the reaction was set up with 2-phenylbenzoxazole (39.0 mg, 0.20 mmol). Purification by flash column chromatography (hexanes:EtOAc= 20:1) provided product **[D]39a** as a white solid (32 mg, 78% yield).

Deuterium Incorporation

General procedure A: [LCMS (ESI)] calcd for $C_{13}D_8NO$ $[M-H]^-$ 8.00 D/molecule, [^1H NMR] 8.54 D/molecule.

NMR Data of the Starting Material

^1H NMR (500 MHz, $CDCl_3$) δ 8.30-8.24 (m, 2H), 7.83-7.74 (m, 1H), 7.64-7.48 (m, 4H), 7.39 - 7.34 (m, 2H).

NMR Data of the Product

General procedure A: ^1H NMR (500 MHz, $CDCl_3$) δ 8.27 (s, 0.10H, 95% D), 7.78 (s, 0.04H, 96% D), 7.63-7.50 (m, 0.26H, 94% D), 7.36 (s, 0.09H, 96% D); ^{13}C NMR (126 MHz, $CDCl_3$) δ 163.2, 150.9, 142.2, 131.5-131.0 (1C), 129.0-128.4 (2C), 127.6-127.4 (2C), 127.2, 125.2-124.8 (1C), 124.6-124.0 (1C), 120.0-119.6 (1C), 110.6-110.2 (1C).

Mass Data

LabelChecker Results

Formula: C13 H8 N O

Mass (monoisotopic): 194.06

Difference Value: 0.308102

Error Sum: 0.555

Error (%): 0.047

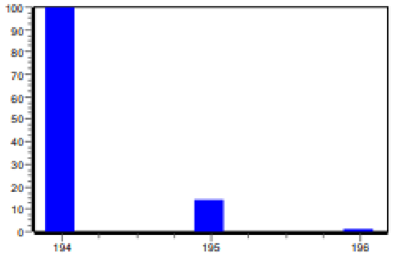

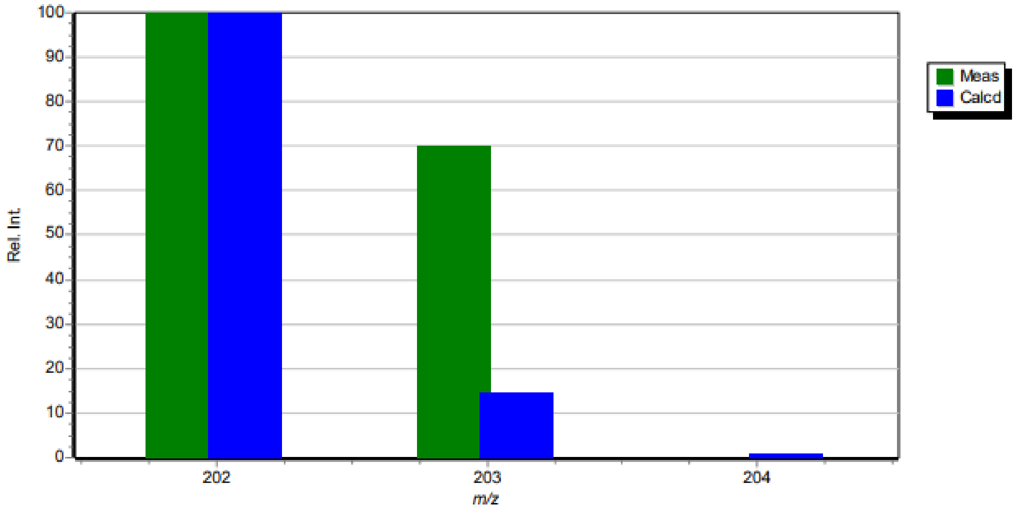

No Convergence! - Max Cycles (20) reached!
Deuterium: 0-fold (%): 0.00 0.00
Deuterium: 1-fold (%): 0.00 0.00
Deuterium: 2-fold (%): 0.00 0.00
Deuterium: 3-fold (%): 0.00 0.00
Deuterium: 4-fold (%): 0.00 0.00
Deuterium: 5-fold (%): 0.00 0.00
Deuterium: 6-fold (%): 0.00 0.00
Deuterium: 7-fold (%): 0.00 0.00
Deuterium: 8-fold (%): 100.00 100.00
Label Atom Sum: 8.00 (100.00%)

Isotope List used for fitting data:

m/z intensity
201.85 4215
202.95 2949

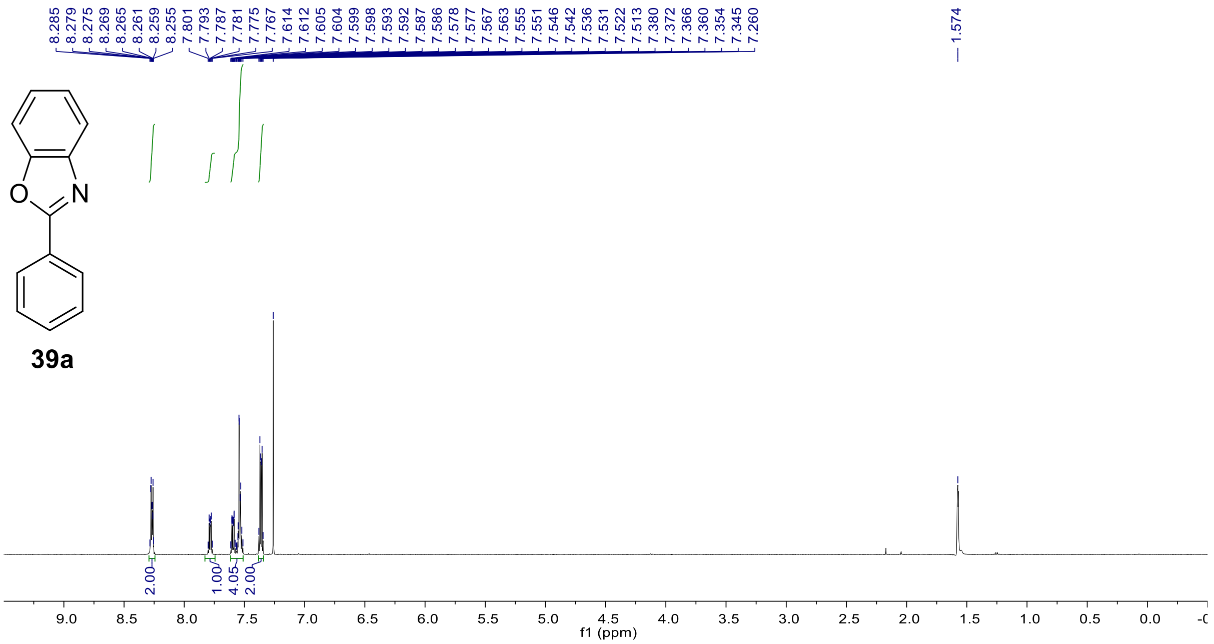

^{1}H NMR spectrum of compound **39a** ($CDCl_3$, 500 MHz)

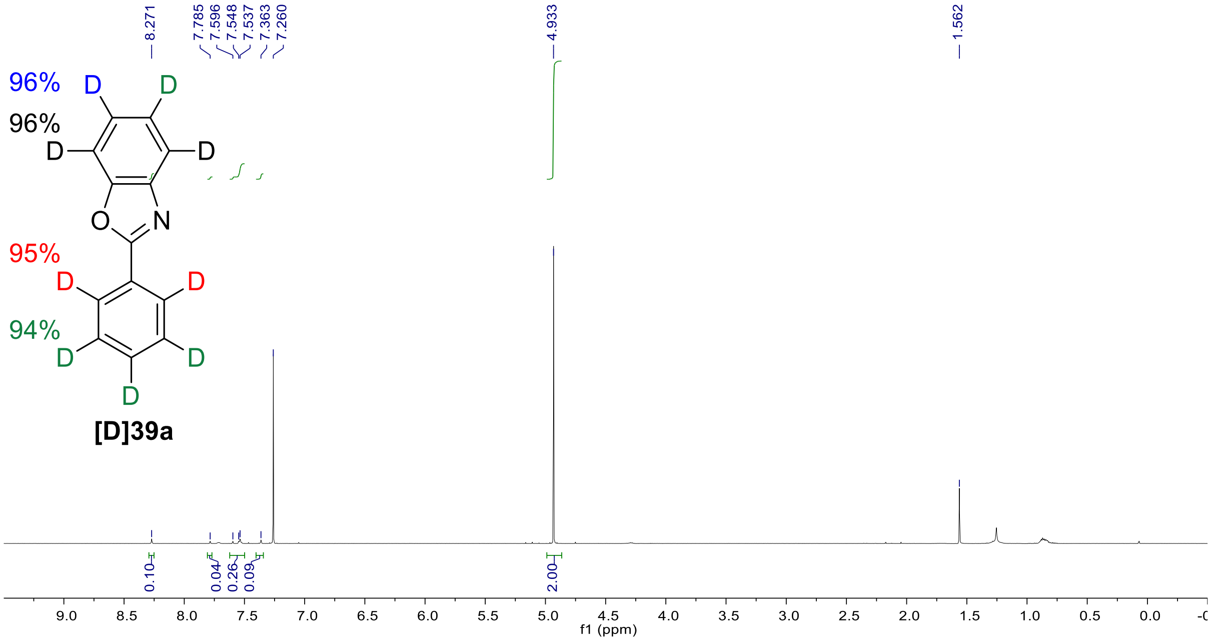

^{1}H NMR spectrum of compound **[D]39a** (Procedure A, $CDCl_3$, 500 MHz)

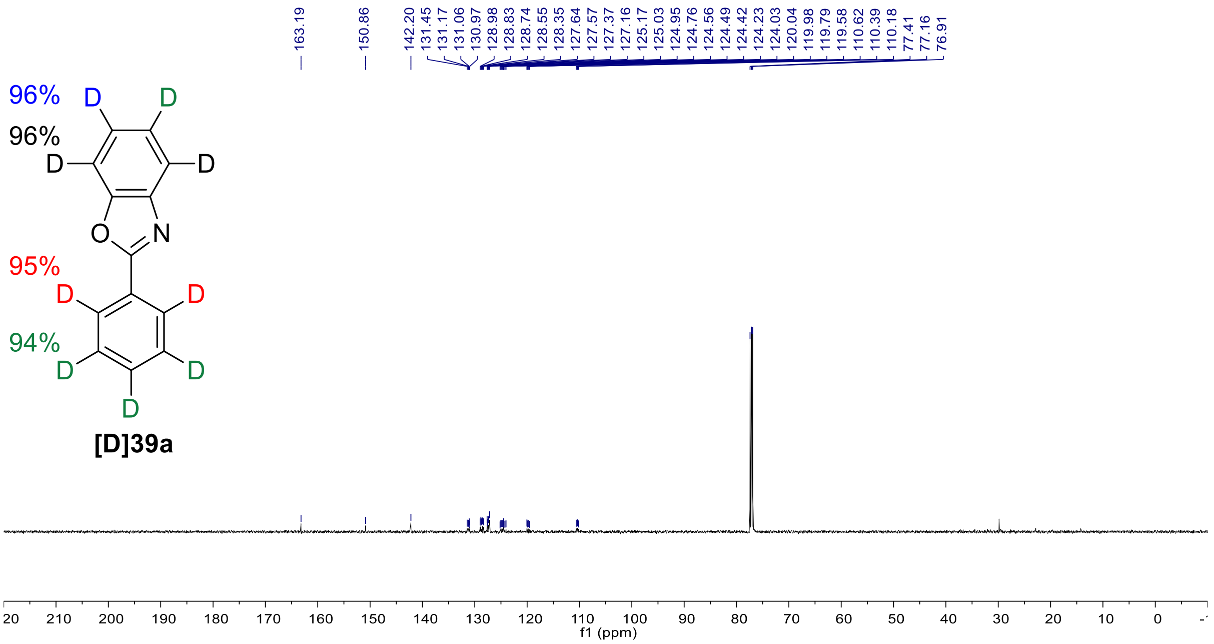

^{13}C NMR spectrum of compound **[D]39a** (Procedure A, $CDCl_3$, 126 MHz)

2-(2-methylphenyl)benzoxazole (39b)

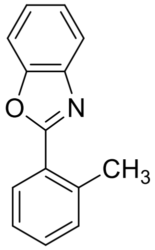

Following the general procedure B, the reaction was set up with 2-(2- methylhenyl)benzoxa zole (41.8 mg, 0.20 mmol). Purification by flash column chromatography (hexanes/EtOAc= 20:1) provided product **[D]39b** as a yellow solid (43 mg, 98% yield).

Deuterium Incorporation

General procedure B: [LCMS (ESI)] calcd for $C_{14}HD_{11}NO$ $[M+H]^+$ 9.11 D/molecule, [^{1}H NMR] 9.46 D/molecule.

NMR Data of the Starting Material

^{1}H NMR (500 MHz, CD_2Cl_2) δ 8.17 (d, J = 7.9 Hz, 1H), 7.80-7.75 (m, 1H), 7.64-7.59 (m, 1H), 7.45-7.34 (m, 5H), 2.81 (s, 3H).

NMR Data of the Product

General procedure B: ^{1}H NMR (500 MHz, CD_2Cl_2) δ 8.18 (s, 0.06H, 94% D), 7.79-7.73 (m, 0.05H, 95% D), 7.62 (s, 0.04H, 96% D), 7.45-7.35 (m, 0.24H, 95% D), 2.83-2.76 (m, 1.15H, 62% D); ^{13}C NMR (126 MHz, CD_2Cl_2) δ 163.3, 150.3, 142.3, 139.0-138.8 (1C), 131.8-131.2 (1C), 130.8-130.2 (1C), 129.7-129.2 (1C), 126.2, 126.0-125.4 (1C), 124.9-124.4 (1C), 124.2-123.7 (1C), 119.9-119.4 (1C), 110.4-109.9 (1C), 22.0-21.2 (1C).

Mass Data

LabelChecker Results

Formula: C14 H12 N O
Mass (monoisotopic): 210.09

Difference Value: 0.000024
Error Sum: 0.005
Error (%): 0.042

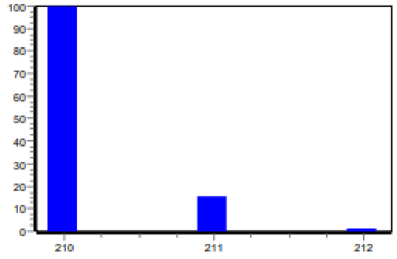

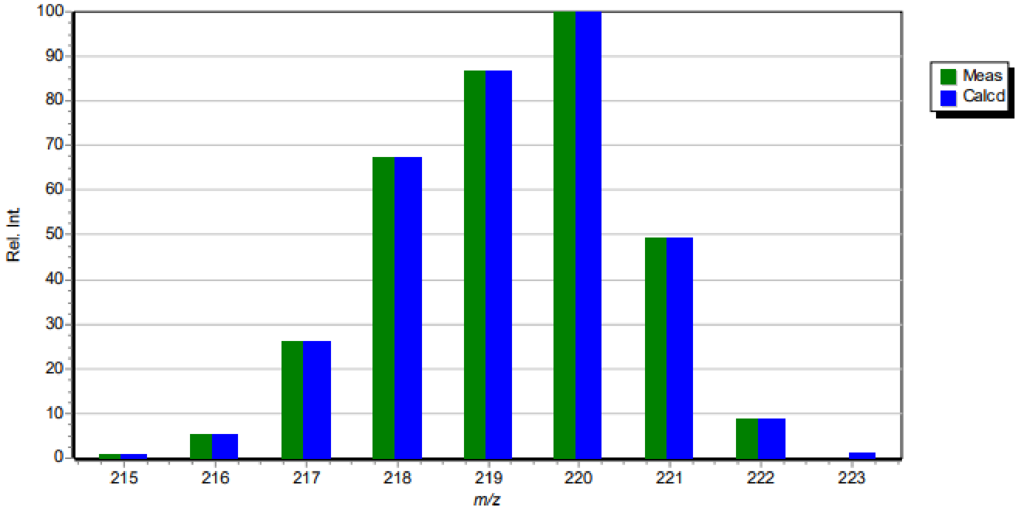

Deuterium: 0-fold (%): 0.00 0.00
Deuterium: 1-fold (%): 0.00 0.00
Deuterium: 2-fold (%): 0.00 0.00
Deuterium: 3-fold (%): 0.00 0.00
Deuterium: 4-fold (%): 0.00 0.00
Deuterium: 5-fold (%): 0.65 0.19
Deuterium: 6-fold (%): 6.23 1.84
Deuterium: 7-fold (%): 29.14 8.59
Deuterium: 8-fold (%): 72.43 21.37
Deuterium: 9-fold (%): 87.83 25.91
Deuterium: 10-fold (%): 100.00 29.50
Deuterium: 11-fold (%): 40.07 11.82
Deuterium: 12-fold (%): 2.64 0.78
Label Atom Sum: 9.11 (75.89%)

Isotope List used for fitting data:

m/z	intensity
215.12	88425
216.13	864094
217.14	4111278
218.14	10521403
219.15	13588779
220.15	15660026
221.16	7766173
222.16	1416747
223.17	65882

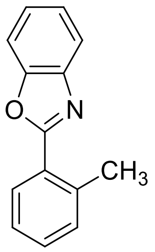

39b

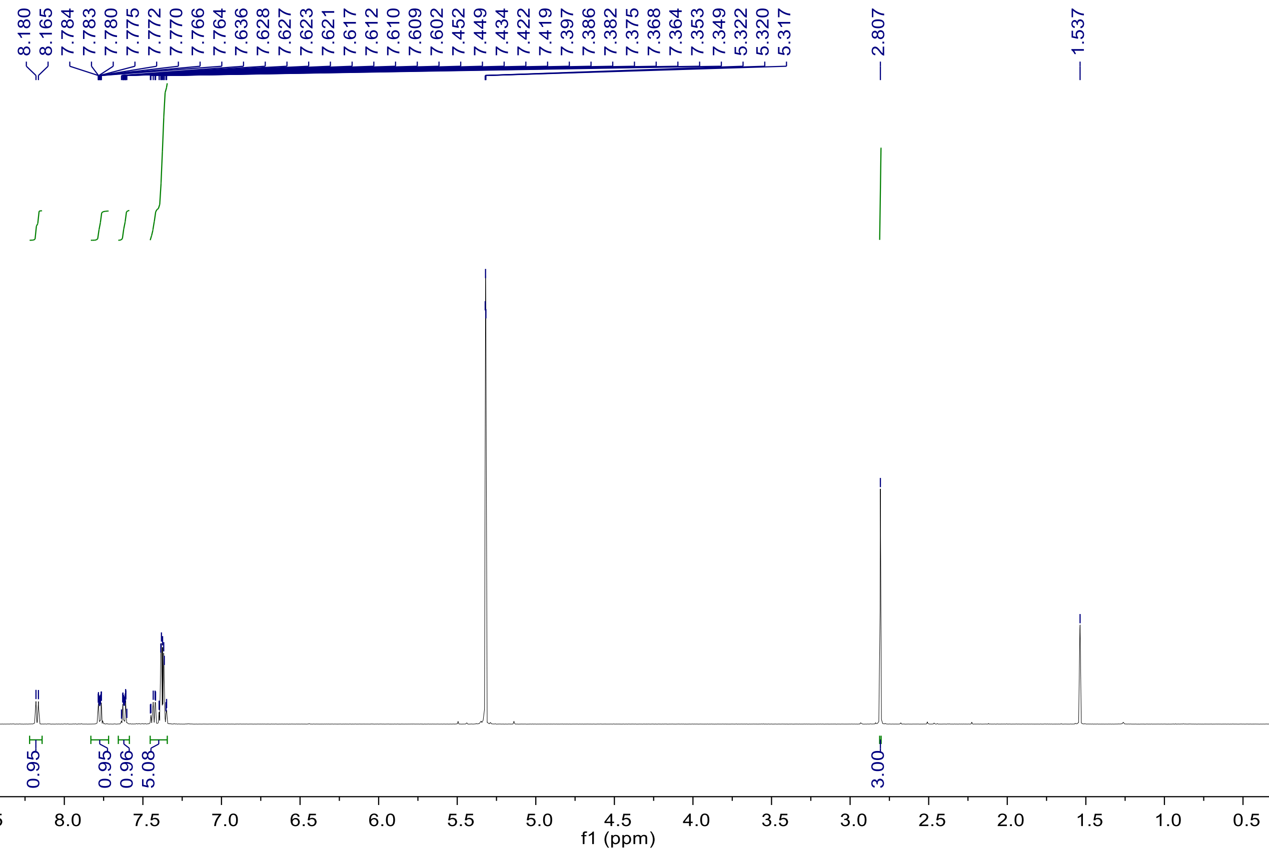

^{1}H NMR spectrum of compound **39b** (CD_2Cl_2, 500 MHz)

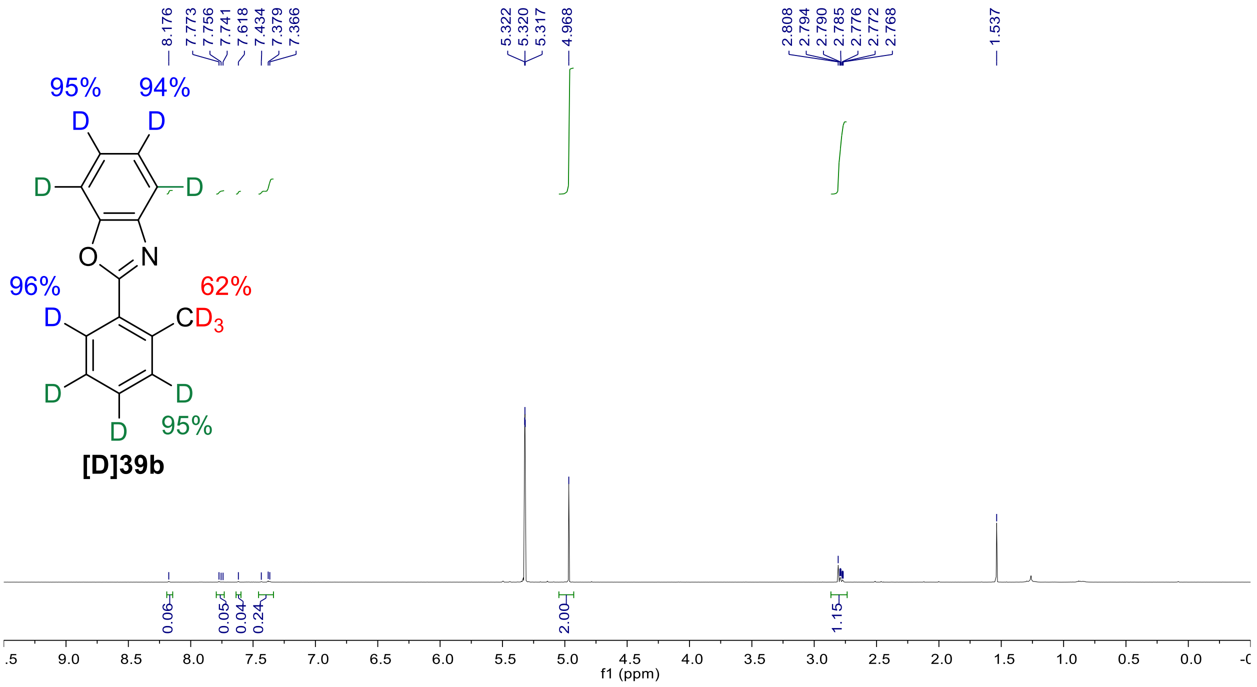

^{1}H NMR spectrum of compound **[D]39b** (Procedure B, CD_2Cl_2, 500 MHz)

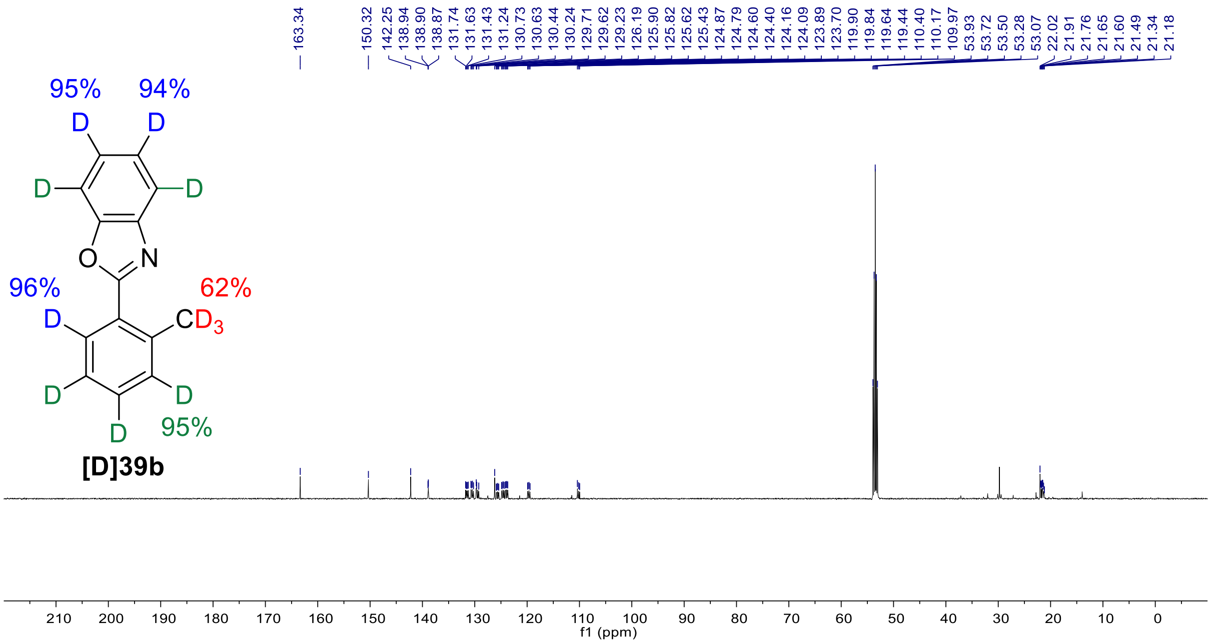

^{13}C NMR spectrum of compound **[D]39b** (Procedure B, CD_2Cl_2, 126 MHz)

2-phenylbenzothiazole (40a)

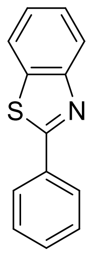

Following the general procedure A, the reaction was set up with 2- phenylbenzothiazole (42.3 mg, 0.20 mmol). Purification by flash column chromatography (hexanes/EtOAc= 20:1) provided product **[D]40a** as a white solid (32 mg, 73% yield).

Deuterium Incorporation

General procedure A: [LCMS (ESI)] calcd for $C_{13}HD_9NS$ $[M+H]^+$ 8.49 D/molecule, [^{1}H NMR] 8.64 D/molecule.

NMR Data of the Starting Material

^{1}H NMR (500 MHz, $CDCl_3$) δ 8.14-8.09 (m, 3H), 7.92 (d, J = 8.0 Hz, 1H), 7.55-7.47 (m, 4H), 7.40 (td, J = 7.7, 7.3, 1.1 Hz, 1H).

NMR Data of the Product

General procedure A: ^{1}H NMR (500 MHz, $CDCl_3$) δ 8.09 (d, J = 8.9 Hz, 0.12H, 96% D), 7.92 (s, 0.04H, 96% D), 7.49 (d, J = 18.5 Hz, 0.18H, 96% D), 7.39 (d, J = 7.5 Hz, 0.04H, 96% D); ^{13}C NMR (126 MHz, $CDCl_3$) δ 168.2, 154.2, 135.1, 133.6, 130.9-130.4 (2C), 128.9-128.5 (2C), 127.6-127.1 (1C), 126.2-125.8 (1C), 125.1-124.6 (1C), 123.3-122.8 (1C), 121.6-121.2 (1C).

Mass Data

LabelChecker Results

Formula: C13 H10 N S
Mass (monoisotopic): 212.05

Difference Value: 0.001813
Error Sum: 0.043
Error (%): 0.331

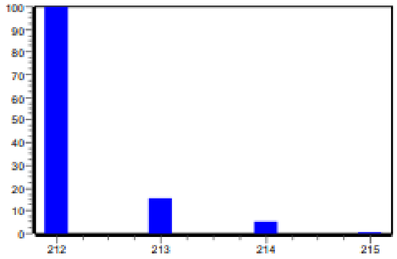

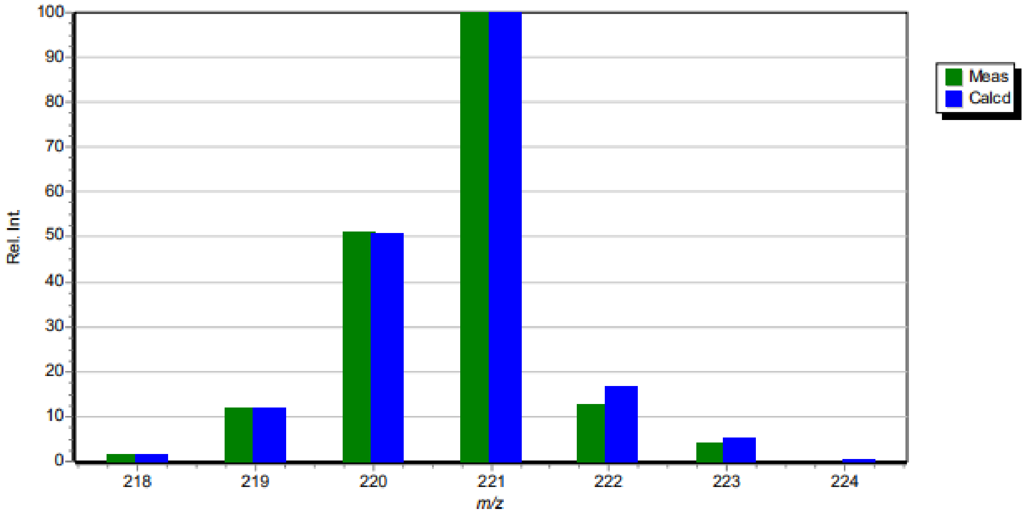

Deuterium: 0-fold (%): 0.00 0.00
Deuterium: 1-fold (%): 0.00 0.00
Deuterium: 2-fold (%): 0.00 0.00
Deuterium: 3-fold (%): 0.01 0.01
Deuterium: 4-fold (%): 0.05 0.03
Deuterium: 5-fold (%): 0.00 0.00
Deuterium: 6-fold (%): 1.88 1.12
Deuterium: 7-fold (%): 12.79 7.61
Deuterium: 8-fold (%): 53.37 31.75
Deuterium: 9-fold (%): 100.00 59.49
Deuterium: 10-fold (%): 0.00 0.00
Label Atom Sum: 8.49 (84.95%)

Isotope List used for fitting data:

m/z	intensity
217.10	266245
218.09	1463670
219.10	10179277
220.10	43360640
221.11	84960232
222.11	10894461
223.10	3487077

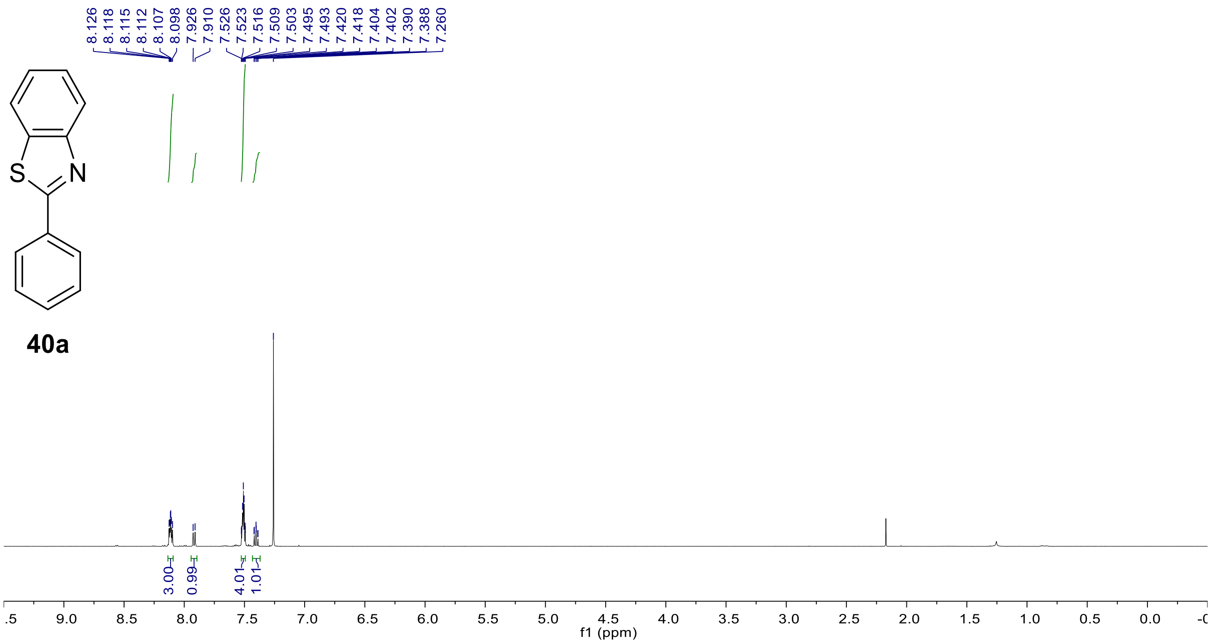

^{1}H NMR spectrum of compound **40a** (CDCl$_3$, 500 MHz)

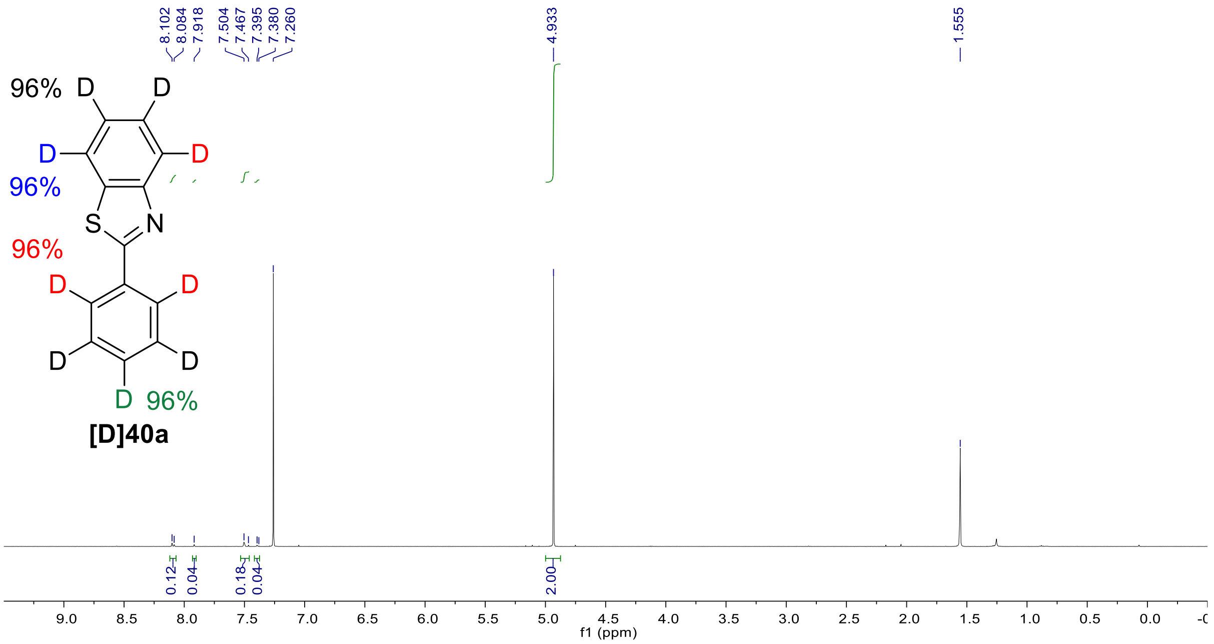

^{1}H NMR spectrum of compound **[D]40a** (Procedure A, CDCl$_3$, 500 MHz)

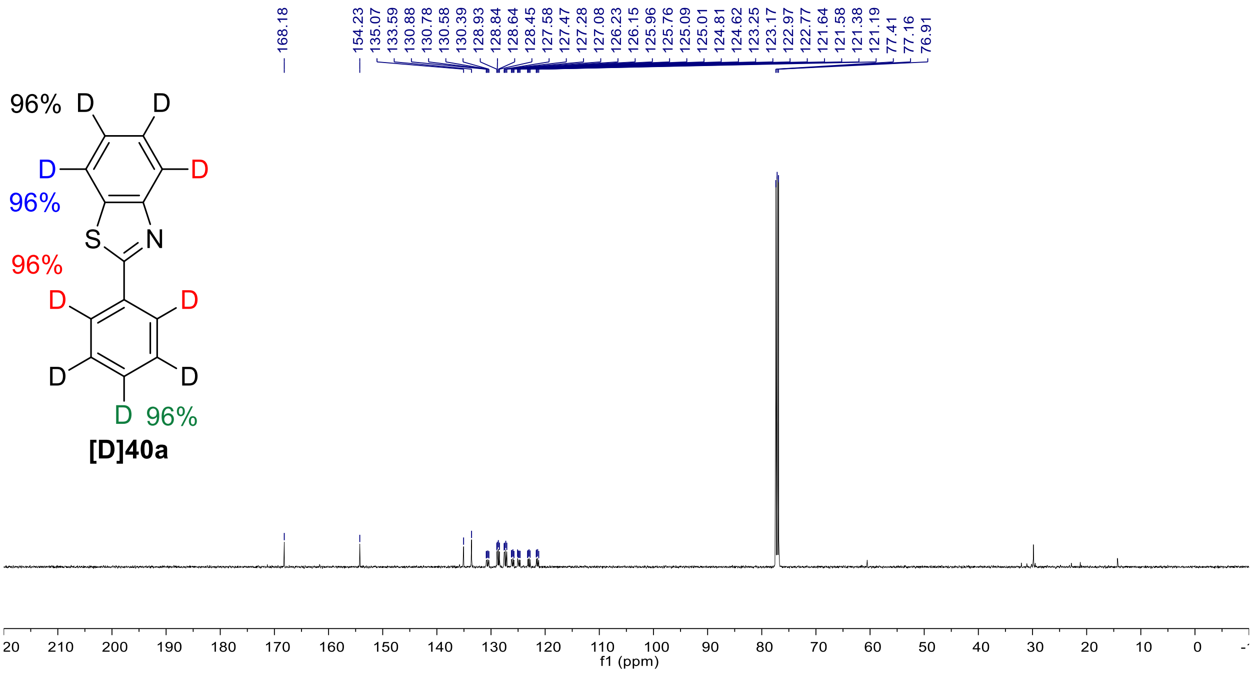

^{13}C NMR spectrum of compound **[D]40a** (Procedure A, $CDCl_3$, 126 MHz)

2-(2-methylphenyl)benzothiazole (40b)

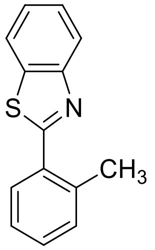

Following the general procedure B, the reaction was set up with 2-(2-methylphenyl)benzothiazole (45.1 mg, 0.20 mmol). Purification by flash column chromatography (hexanes/EtOAc=10:1) provided product **[D]40b** as a white solid (43 mg, 91% yield).

Deuterium Incorporation

General procedure B: [LCMS (ESI)] calcd for $C_{14}HD_{11}NS$ $[M+H]^+$ 9.98 D/molecule, [^{1}H NMR] 10.4 D/molecule.

NMR Data of the Starting Material

^{1}H NMR (500 MHz, $CDCl_3$) δ 8.11 (d, *J* = 8.1 Hz, 1H), 7.94 (dd, *J* = 8.0, 0.6 Hz, 1H), 7.76 (dd, *J* = 7.6, 1.2 Hz, 1H), 7.54-7.50 (m, 1H), 7.44-7.30 (m, 4H), 2.66 (s, 3H).

NMR Data of the Product

General procedure B: ^{1}H NMR (500 MHz, $CDCl_3$) δ 8.11 (s, 0.05H, 95% D), 7.94 (s, 0.05H, 95% D), 7.76 (s, 0.05H, 95% D), 7.51 (m, 0.06H, 94% D), 7.43-7.31 (m, 0.20H, 95% D), 2.66-2.62 (m, 0.17H, 94% D); ^{13}C NMR (126 MHz, $CDCl_3$) δ 168.1, 153.9, 137.2, 135.6, 133.1, 131.5-131.0 (1C), 130.5-130.0 (1C), 129.9-129.4 (1C), 126.0-125.5 (1C), 125.0-124.5 (1C), 123.4-122.9 (1C), 121.4-121.3 (1C), 121.1-120.9 (1C), 21.4-20.5 (1C).

Mass Data

LabelChecker Results

Formula: C14 H12 N S
Mass (monoisotopic): 226.07

Difference Value: 0.003630
Error Sum: 0.060
Error (%): 0.000

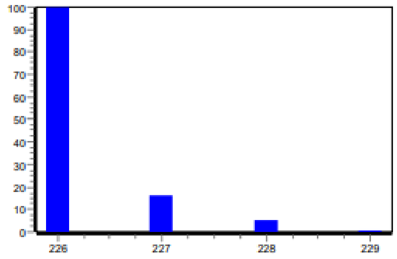

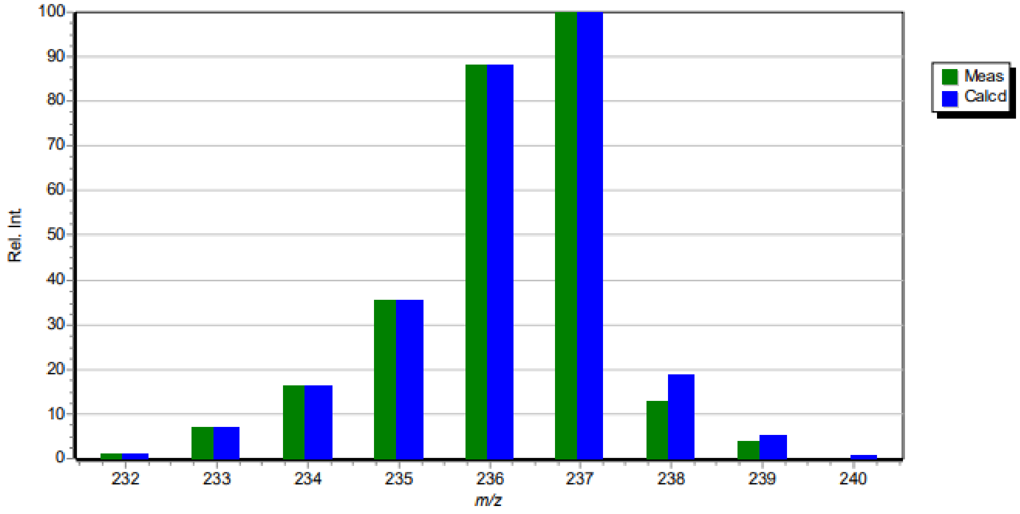

Deuterium: 0-fold (%): 0.00 0.00
Deuterium: 1-fold (%): 0.00 0.00
Deuterium: 2-fold (%): 0.00 0.00
Deuterium: 3-fold (%): 0.00 0.00
Deuterium: 4-fold (%): 0.00 0.00
Deuterium: 5-fold (%): 0.00 0.00
Deuterium: 6-fold (%): 1.72 0.65
Deuterium: 7-fold (%): 8.18 3.11
Deuterium: 8-fold (%): 18.08 6.87
Deuterium: 9-fold (%): 38.25 14.54
Deuterium: 10-fold (%): 96.81 36.81
Deuterium: 11-fold (%): 100.00 38.02
Deuterium: 12-fold (%): 0.00 0.00
Label Atom Sum: 9.98 (83.15%)

Isotope List used for fitting data:

m/z intensity
230.25 228669
231.12 585056
232.11 4776482
233.11 23562688
234.12 54283244
235.12 115895896
236.13 290532544
237.14 328891584
238.14 42607180
239.13 13143490

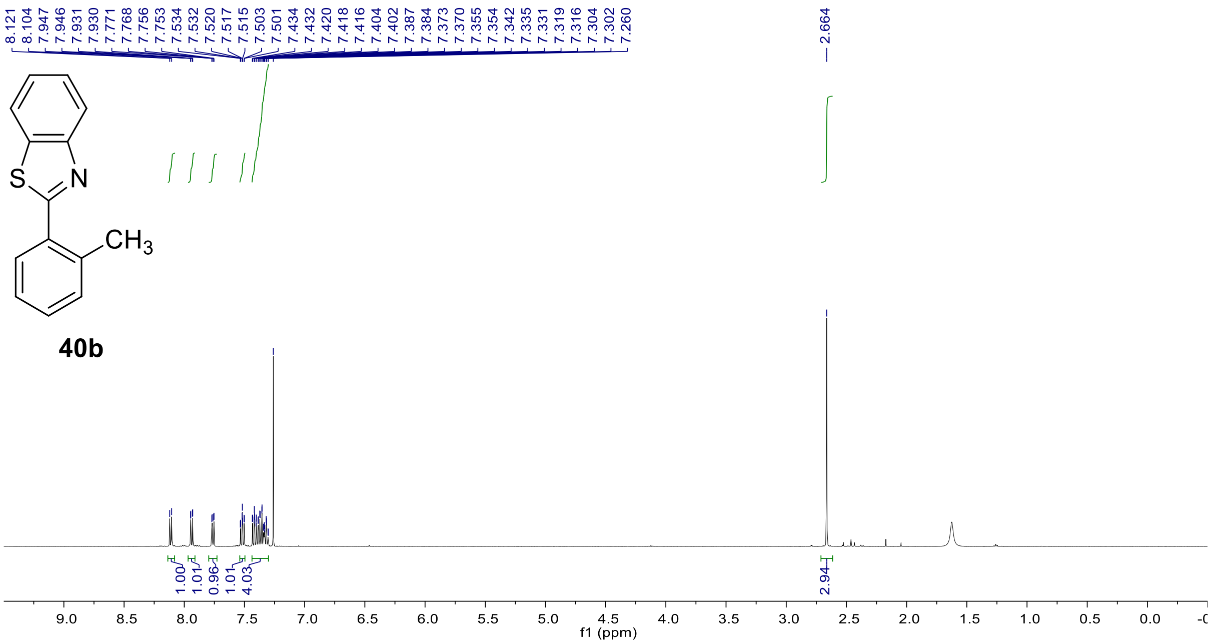

^{1}H NMR spectrum of compound **40b** ($CDCl_3$, 500 MHz)

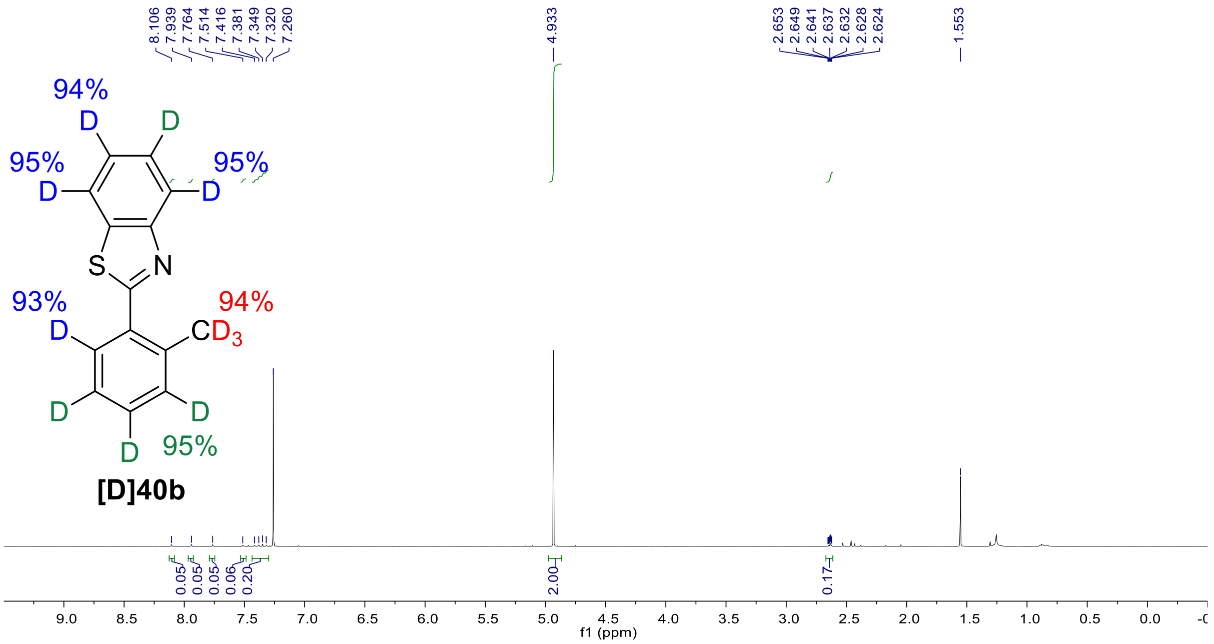

^{1}H NMR spectrum of compound **[D]40b** (Procedure B, $CDCl_3$, 500 MHz)

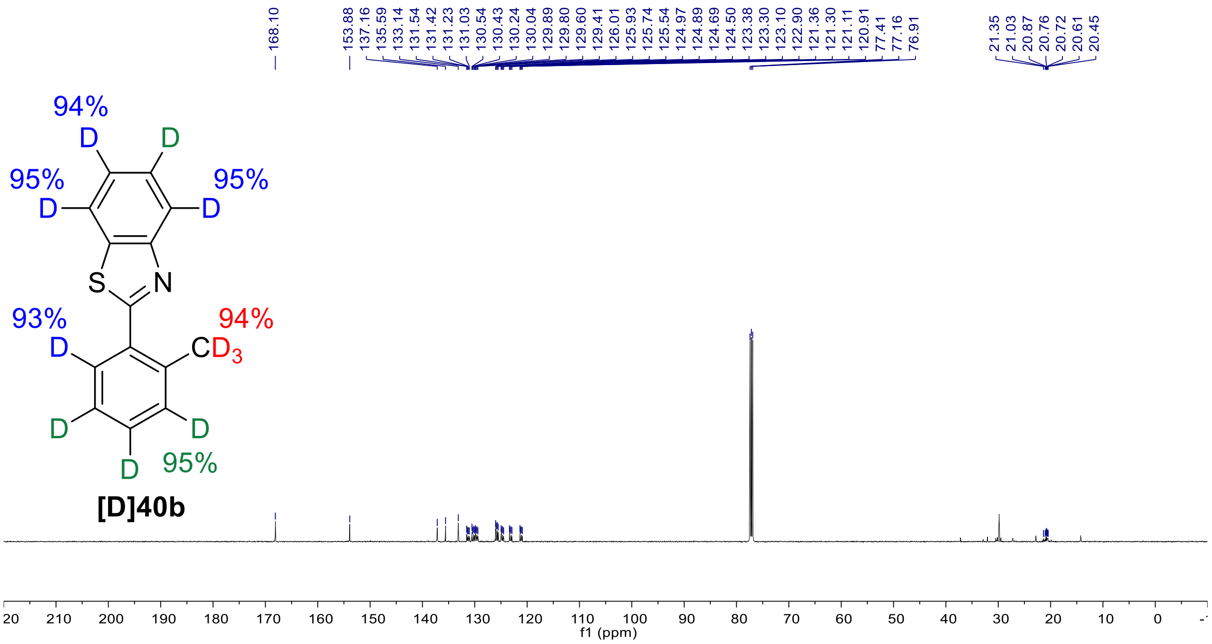

^{13}C NMR spectrum of compound **[D]40b** (Procedure B, $CDCl_3$, 126 MHz)

2-phenylthiazole (41a)

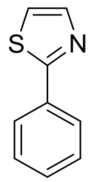

Following the general procedure A, the reaction was set up with 2-phenylthiazole (32.2 mg, 0.20 mmol). Purification by flash column chromatography (hexanes/EtOAc=10:1) provided product **[D]41a** as a yellow oil (29 mg, 86% yield).

Deuterium Incorporation

General procedure A: [LCMS (ESI)] calcd for C_9HD_7NS $[M+H]^+$ 6.66 D/molecule, [^{1}H NMR] 6.83 D/molecule.

NMR Data of the Starting Material

^{1}H NMR (500 MHz, $CDCl_3$) δ 7.99-7.96 (m, 2H), 7.87 (d, J = 3.3 Hz, 1H), 7.47-7.40 (m, 3H), 7.32 (d, J = 3.3 Hz, 1H).

NMR Data of the Product

General procedure A: ^{1}H NMR (500 MHz, $CDCl_3$) δ 7.97 (s, 0.05H, 98% D), 7.87 (s, 0.02H, 98% D), 7.47-7.41 (m, 0.10H, 97% D), 7.33 (s, 0.02H, 98% D); ^{13}C NMR (126 MHz, $CDCl_3$) δ 168.5, 143.7-143.3 (1C), 133.7-133.6 (2C), 129.9-129.4 (2C), 128.9-128.4 (1C), 126.6-126.1 (1C), 118.8-118.4 (1C).

Mass Data

LabelChecker Results

Formula: C9 H8 N S
Mass (monoisotopic): 162.04

Difference Value: 0.001576
Error Sum: 0.040
Error (%): 0.269

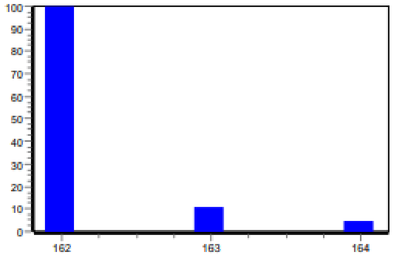

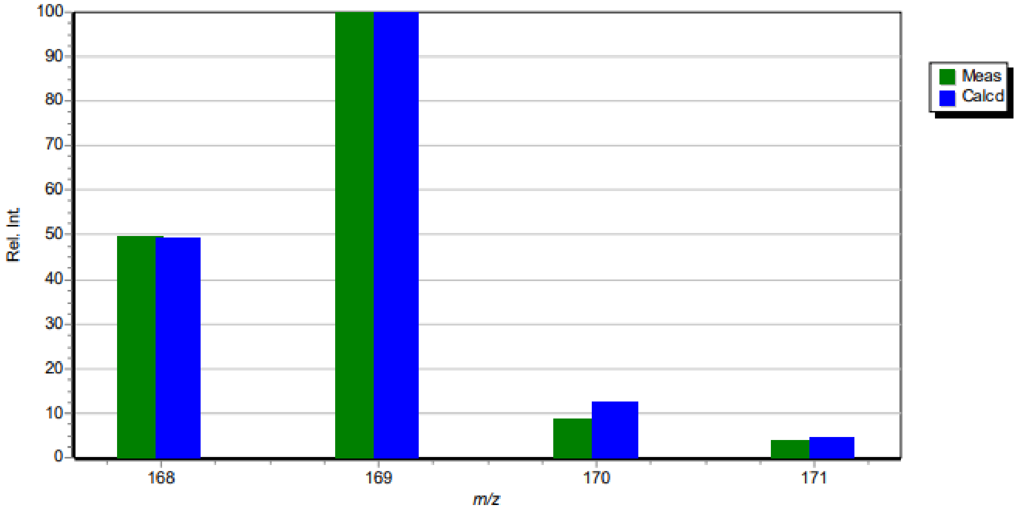

Deuterium: 0-fold (%): 0.00 0.00
Deuterium: 1-fold (%): 0.00 0.00
Deuterium: 2-fold (%): 0.00 0.00
Deuterium: 3-fold (%): 0.00 0.00
Deuterium: 4-fold (%): 0.01 0.00
Deuterium: 5-fold (%): 0.02 0.01
Deuterium: 6-fold (%): 52.49 34.42
Deuterium: 7-fold (%): 100.00 65.57
Deuterium: 8-fold (%): 0.00 0.00
Label Atom Sum: 6.66 (83.19%)

Isotope List used for fitting data:

m/z	intensity
168.07	174676432
169.08	350801344
170.08	31252642
171.08	14752667
172.08	1214486

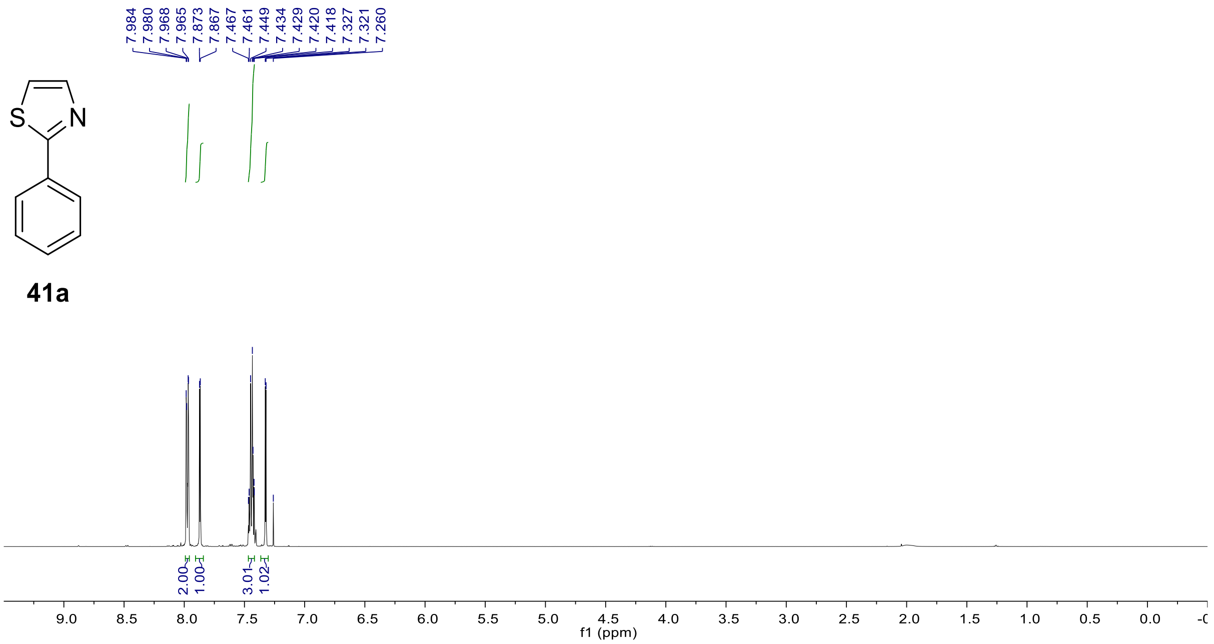

^{1}H NMR spectrum of compound **41a** ($CDCl_3$, 500 MHz)

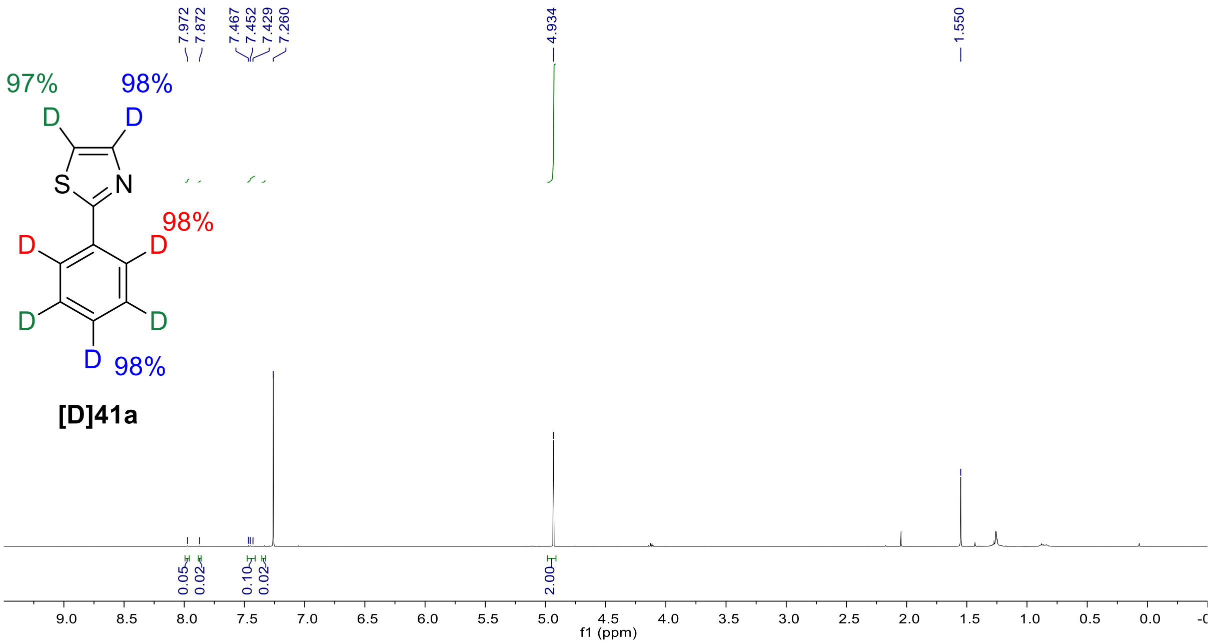

^{1}H NMR spectrum of compound **[D]41a** (Procedure A, $CDCl_3$, 500 MHz)

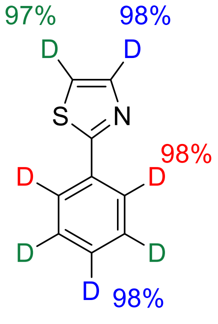

[D]41a

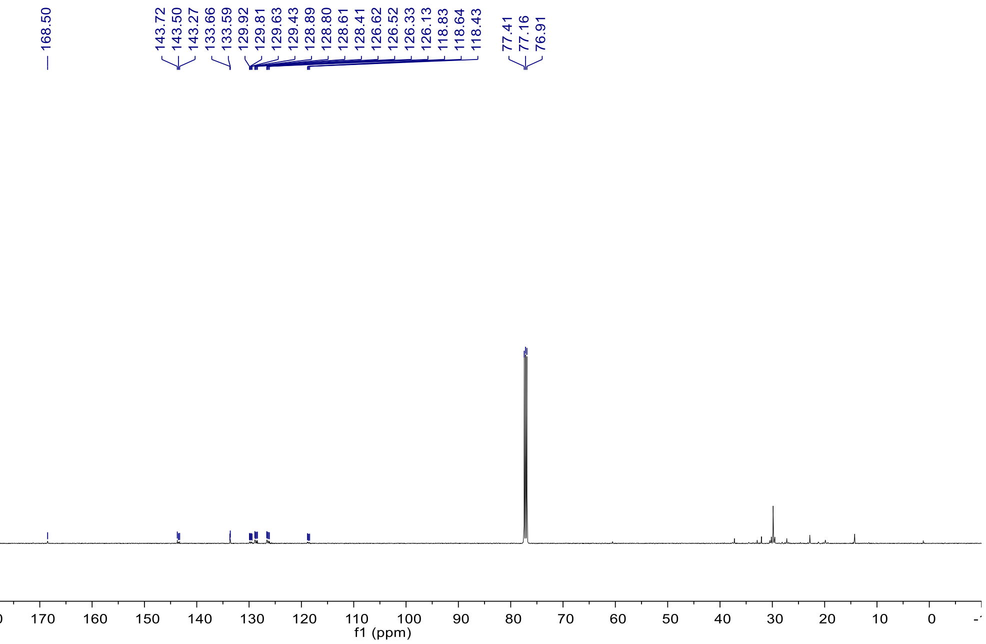

^{13}C NMR spectrum of compound **[D]41a** (Procedure A, $CDCl_3$, 126 MHz)

2-(2-methylphenyl)thiazole (41b)

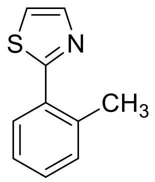

Following the general procedure B, the reaction was set up with 2-(2-methylphenyl)thiazole (35.1 mg, 0.20 mmol). Purification by flash column chromatography (hexanes/EtOAc=10:1) provided product **[D]41b** as a yellow oil (25 mg, 68% yield).

Deuterium Incorporation

General procedure B: [LCMS (ESI)] calcd for $C_{10}HD_9NS$ $[M+H]^+$ 7.53 D/molecule, [^{1}H NMR] 8.01 D/molecule.

NMR Data of the Starting Material

^{1}H NMR (500 MHz, CD_2Cl_2) δ 7.90 (d, *J* = 3.3 Hz, 1H), 7.73 (d, *J* = 7.4 Hz, 1H), 7.43 (d, *J* = 3.3 Hz, 1H), 7.39-7.23 (m, 3H), 2.58 (s, 3H).

NMR Data of the Product

General procedure B: ^{1}H NMR (500 MHz, CD_2Cl_2) δ 7.90 (s, 0.05H, 95% D), 7.72 (s, 0.08H, 92% D), 7.44 (d, *J* = 9.8 Hz, 0.08H, 92% D), 7.36-7.25 (m, 0.45H, 85% D), 2.59-2.52 (m, 0.32H, 89% D); ^{13}C NMR (126 MHz, CD_2Cl_2) δ 167.7, 142.9-142.5 (1C), 136.5, 131.5-130.9 (1C), 130.0-129.6 (1C), 129.3-129.0 (1C), 128.9-128.7 (1C), 126.0-125.4 (1C), 119.6-119.0 (1C), 21.2-20.4 (1C).

Mass Data

LabelChecker Results

Formula: C10 H10 N S
Mass (monoisotopic): 176.05

Difference Value: 0.000170
Error Sum: 0.013
Error (%): 0.000

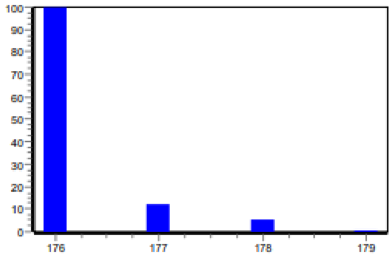

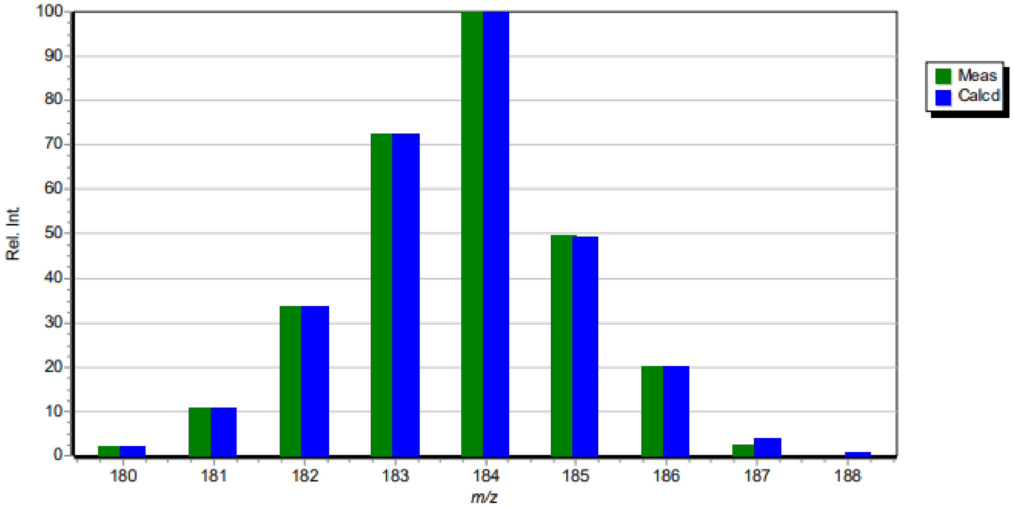

No Convergence! - Max Iterations (3000) reached!
Deuterium: 0-fold (%): 0.00 0.00
Deuterium: 1-fold (%): 0.00 0.00
Deuterium: 2-fold (%): 0.00 0.00
Deuterium: 3-fold (%): 0.29 0.11
Deuterium: 4-fold (%): 2.39 0.86
Deuterium: 5-fold (%): 11.72 4.23
Deuterium: 6-fold (%): 35.66 12.87
Deuterium: 7-fold (%): 75.72 27.33
Deuterium: 8-fold (%): 100.00 36.10
Deuterium: 9-fold (%): 39.02 14.09
Deuterium: 10-fold (%): 12.21 4.41
Label Atom Sum: 7.53 (75.31%)

Isotope List used for fitting data:

m/z	intensity
179.07	327561
180.08	2708152
181.08	13417730
182.09	41502768
183.10	89965016
184.10	123866792
185.11	61542808
186.09	25235678
187.09	3211105

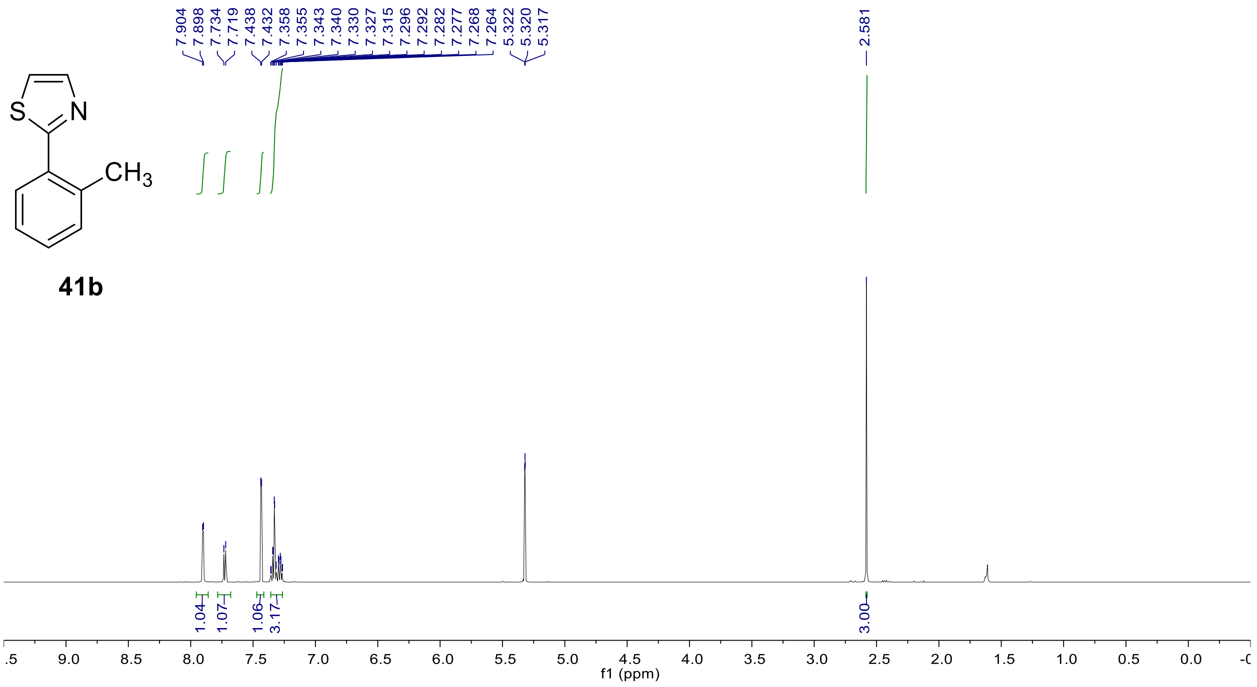

^{1}H NMR spectrum of compound **41b** (CD_2Cl_2, 500 MHz)

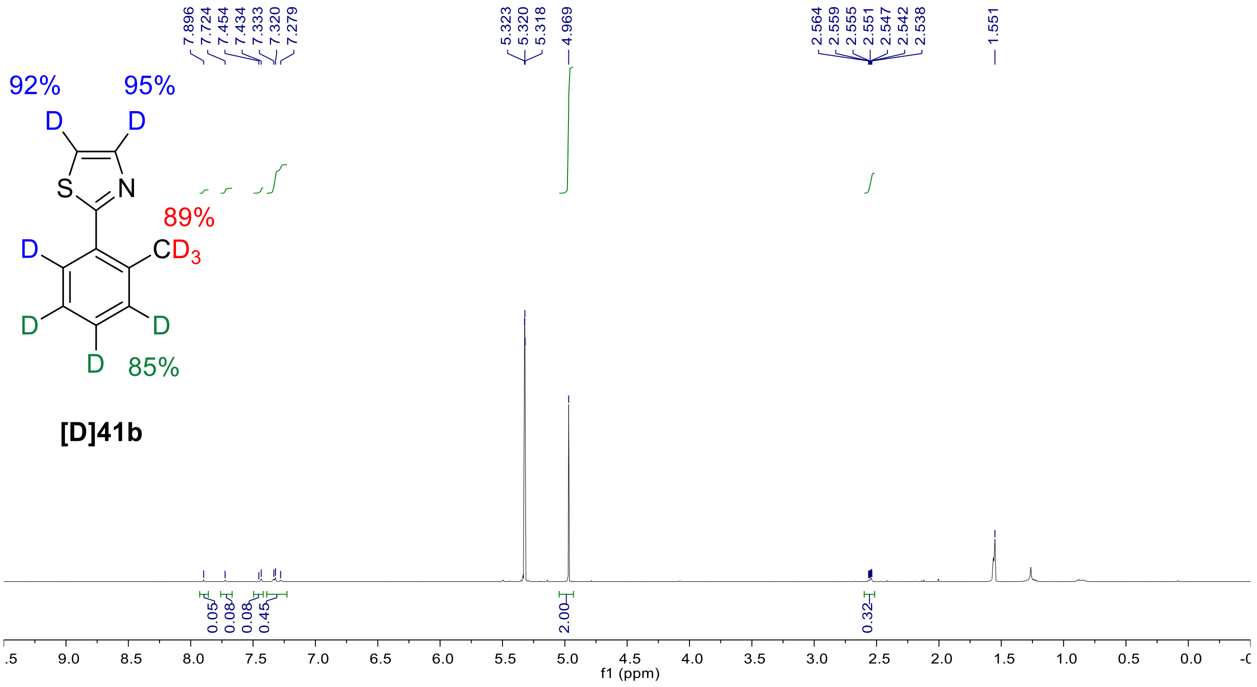

^{1}H NMR spectrum of compound **[D]41b** (Procedure B, CD_2Cl_2, 500 MHz)

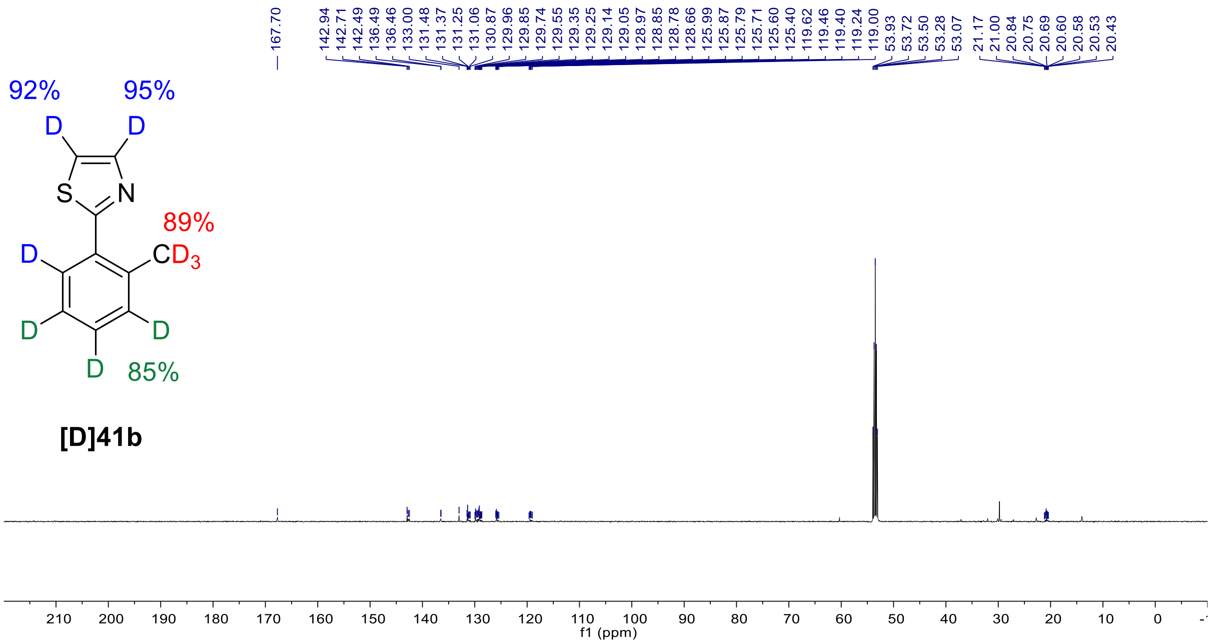

^{13}C NMR spectrum of compound **[D]41b** (Procedure B, CD_2Cl_2, 126 MHz)

1-phenylpyrazole (42a)

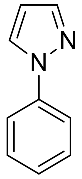

Following the general procedure A, the reaction was set up with 1-phenylpyrazole (28.8 mg, 0.20 mmol). Purification by flash column chromatography (hexanes/EtOAc=10:1) provided product **[D]42a** as a yellow oil (18 mg, 59% yield).

Deuterium Incorporation

General procedure A: [LCMS (ESI)] calcd for $C_9D_7N_2$ $[M-H]^-$ 7.00 D/molecule, [^{1}H NMR] 7.57 D/molecule.

NMR Data of the Starting Material

General procedure A: ^{1}H NMR (500 MHz, CD_2Cl_2) δ 7.98 (dd, J = 2.5, 0.5 Hz, 1H), 7.76-7.67 (m, 3H), 7.52-7.42 (m, 2H), 7.35-7.25 (m, 1H), 6.48 (dd, J = 2.4, 1.8 Hz, 1H).

NMR Data of the Product

General procedure A: ^{1}H NMR (500 MHz, CD_2Cl_2) δ 7.97 (s, 0.04H, 96% D), 7.72-7.68 (m, 0.15H, 95% D), 7.46 (s, 0.14H, 93% D), 7.29 (s, 0.05H, 95% D), 6.47 (s, 0.05H, 95% D); ^{13}C NMR (126 MHz, CD_2Cl_2) δ 140.9-140.4 (1C), 140.2, 129.2-128.8 (2C), 126.7-126.2 (2C), 126.1-125.6 (1C), 118.9-118.4 (1C), 107.4-106.9 (1C).

Mass Data

LabelChecker Results

Formula: C9 H7 N2
Mass (monoisotopic): 143.06

Difference Value: 0.265780
Error Sum: 0.516
Error (%): 0.517

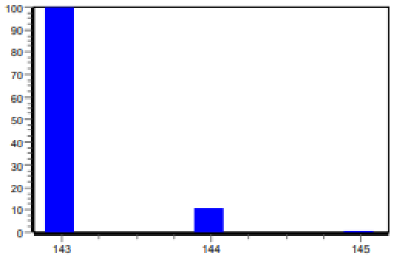

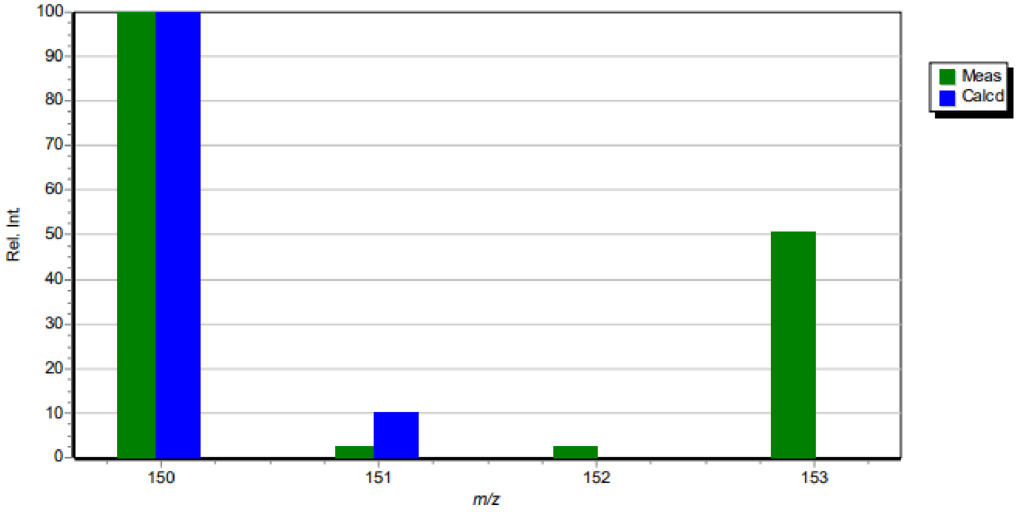

No Convergence! - Max Iterations (3000) reached!
Deuterium: 0-fold (%): 0.00 0.00
Deuterium: 1-fold (%): 0.00 0.00
Deuterium: 2-fold (%): 0.00 0.00
Deuterium: 3-fold (%): 0.00 0.00
Deuterium: 4-fold (%): 0.00 0.00
Deuterium: 5-fold (%): 0.00 0.00
Deuterium: 6-fold (%): 0.04 0.04
Deuterium: 7-fold (%): 100.00 99.96
Label Atom Sum: 7.00 (99.99%)

Isotope List used for fitting data:

m/z	intensity
149.99	9224642
151.00	261164
152.00	216207
153.02	4701403

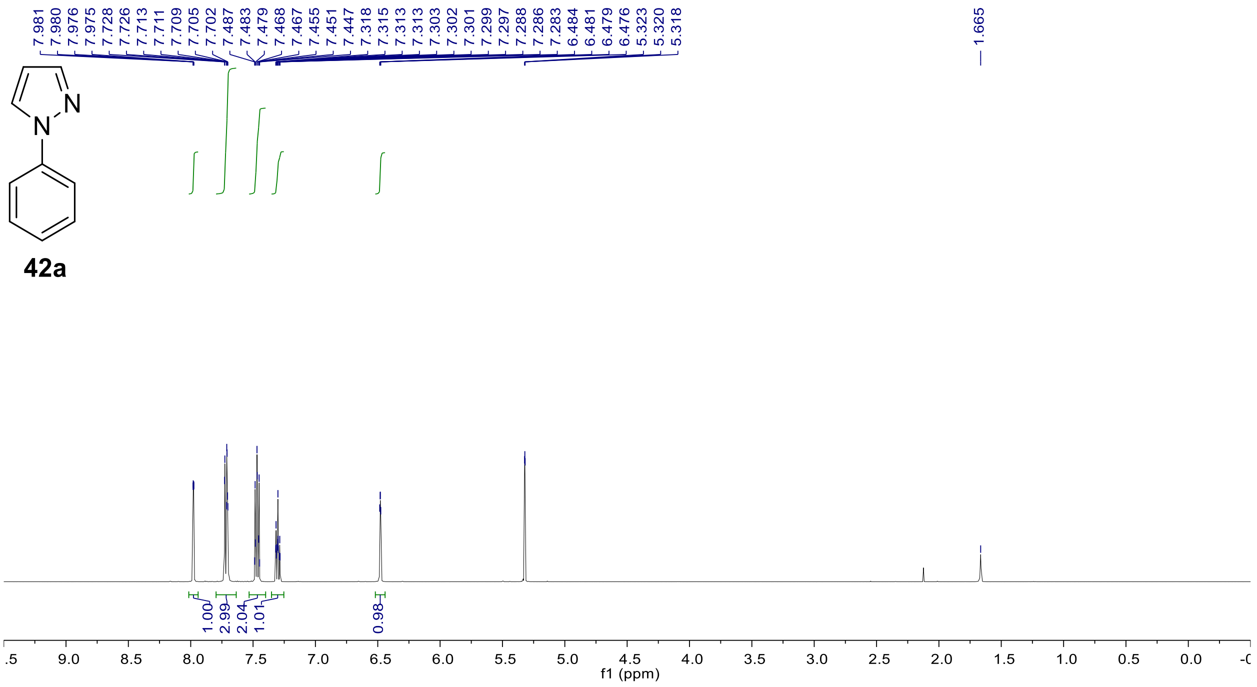

^{1}H NMR spectrum of compound **42a** (CD_2Cl_2, 500 MHz)

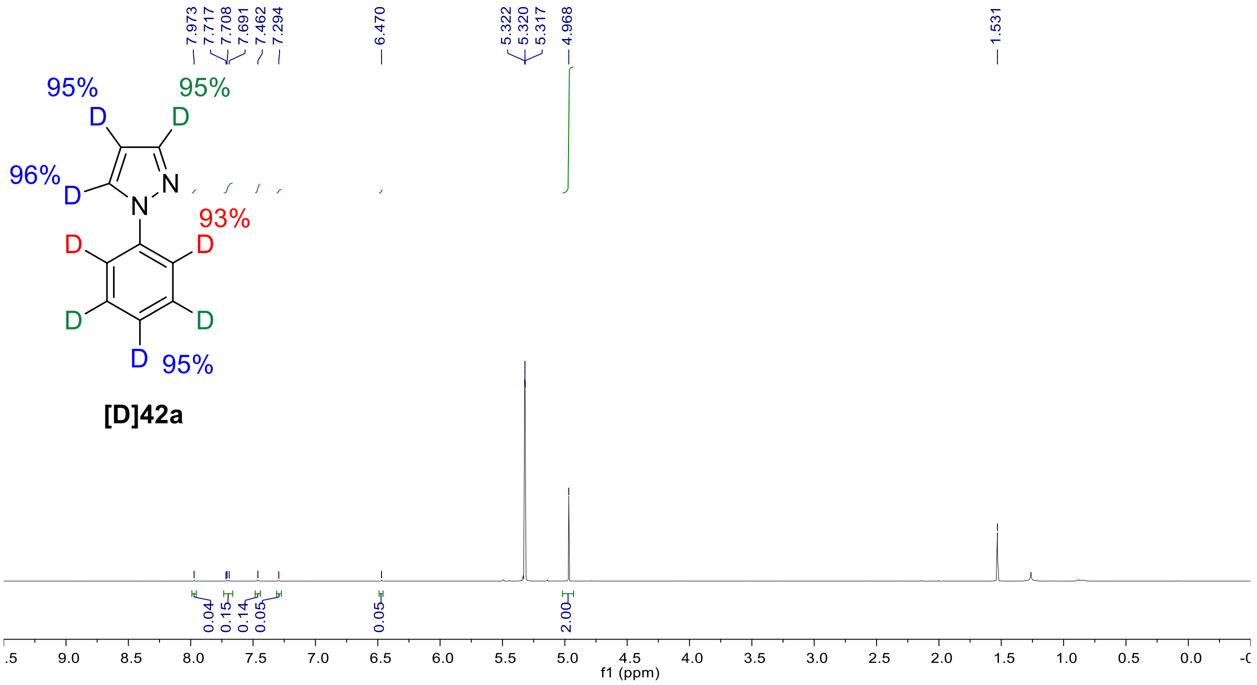

^{1}H NMR spectrum of compound **[D]42a** (Procedure A, CD_2Cl_2, 500 MHz)

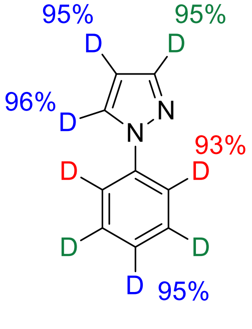

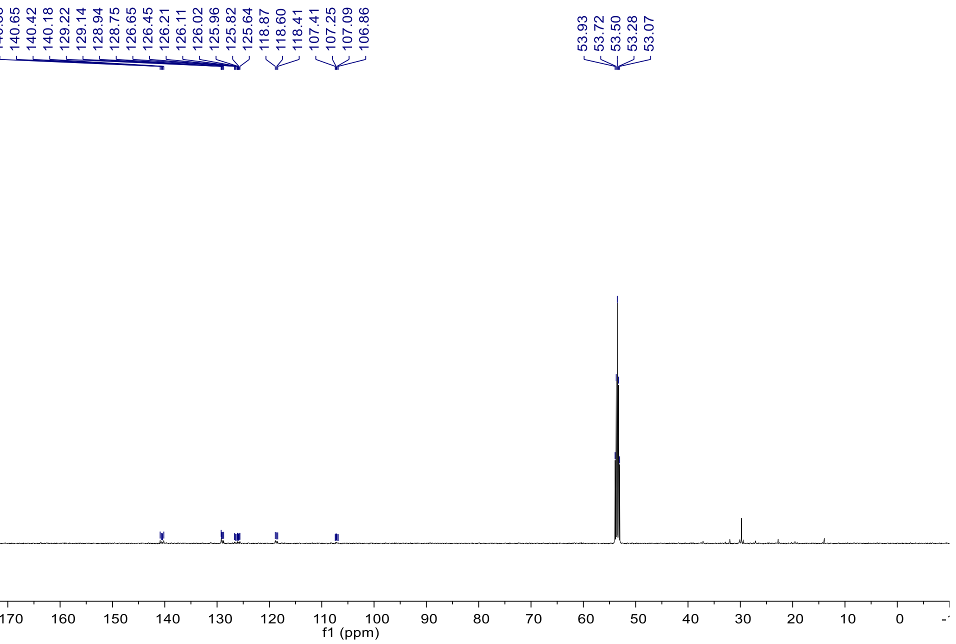

^{13}C NMR spectrum of compound **[D]42a** (Procedure A, CD_2Cl_2, 126 MHz)

1-(2-methylphenyl)-1*H*-pyrazole (42b)

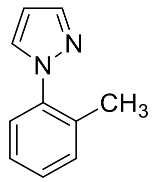

Following the general procedure B, the reaction was set up with 1-(2-methylphenyl)-1*H*-pyrazole (31.6 mg, 0.20 mmol). Purification by flash column chromatography (hexanes/EtOAc=10:1) provided product **[D]42b** as a yellow oil (21 mg, 62% yield).

Deuterium Incorporation

General procedure B: [LCMS (ESI)] calcd for $C_{10}HD_{10}N_2$ $[M+H]^+$ 9.44 D/molecule, [^{1}H NMR] 9.67 D/molecule.

NMR Data of the Starting Material

^{1}H NMR (500 MHz, CD_2Cl_2) δ 7.69 (d, *J* = 1.6 Hz, 1H), 7.64 (d, *J* = 2.3 Hz, 1H), 7.34-7.29 (m, 4H), 6.45 (t, *J* = 2.1 Hz, 1H), 2.24 (s, 3H).

NMR Data of the Product

General procedure B: ^{1}H NMR (500 MHz, CD_2Cl_2) δ 7.68 (s, 0.03H, 97% D), 7.63 (s, 0.03H, 97% D), 7.35-7.27 (m, 0.14H, 97% D), 6.44 (s, 0.03H, 97% D), 2.22-2.18 (m, 0.11H, 96% D); ^{13}C NMR (126 MHz, CD_2Cl_2) δ 140.1-140.0 (1C), 139.8-139.6 (1C), 133.5, 131.2-130.7 (1C), 130.5-130.1 (1C), 128.0-127.6 (1C), 126.3-126.1 (1C), 125.9-125.4 (1C), 105.9-105.4 (1C), 17.5-16.9 (1C).

Mass Data

LabelChecker Results

Formula: C10 H11 N2
Mass (monoisotopic): 159.09

Difference Value: 0.000118
Error Sum: 0.011
Error (%): 0.313

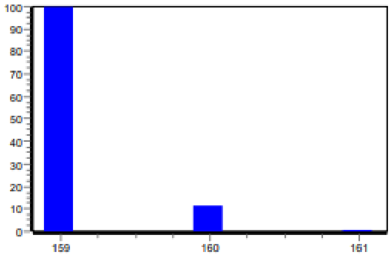

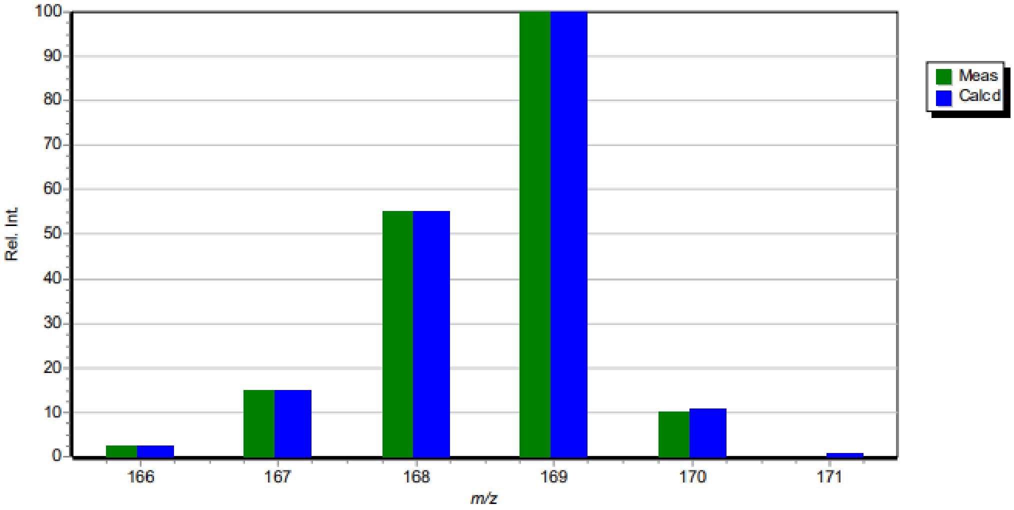

Deuterium: 0-fold (%): 0.00 0.00
Deuterium: 1-fold (%): 0.00 0.00
Deuterium: 2-fold (%): 0.00 0.00
Deuterium: 3-fold (%): 0.00 0.00
Deuterium: 4-fold (%): 0.00 0.00
Deuterium: 5-fold (%): 0.00 0.00
Deuterium: 6-fold (%): 0.22 0.13
Deuterium: 7-fold (%): 2.63 1.50
Deuterium: 8-fold (%): 15.80 8.99
Deuterium: 9-fold (%): 57.23 32.54
Deuterium: 10-fold (%): 100.00 56.85
Deuterium: 11-fold (%): 0.00 0.00
Label Atom Sum: 9.44 (85.86%)

Isotope List used for fitting data:

m/z	intensity
165.13	459050
166.14	5523153
167.14	33456876
168.15	122679528
169.15	221665072
170.16	22702290

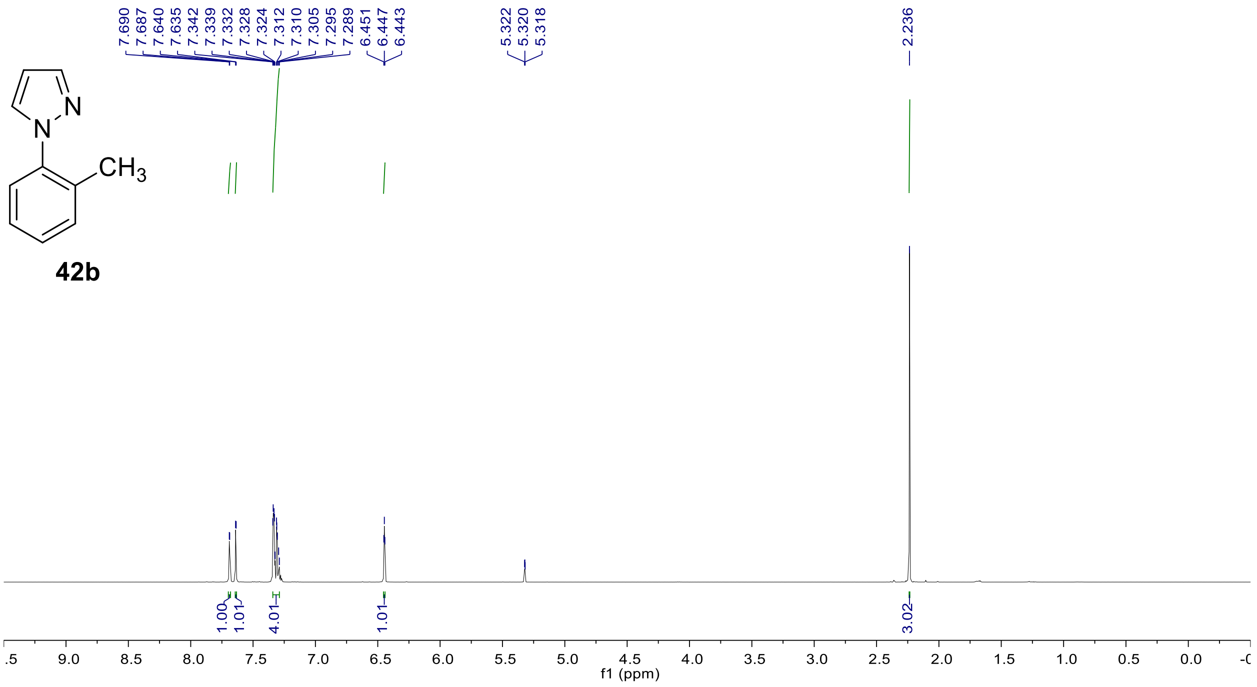

^{1}H NMR spectrum of compound **42b** (CD_2Cl_2, 500 MHz)

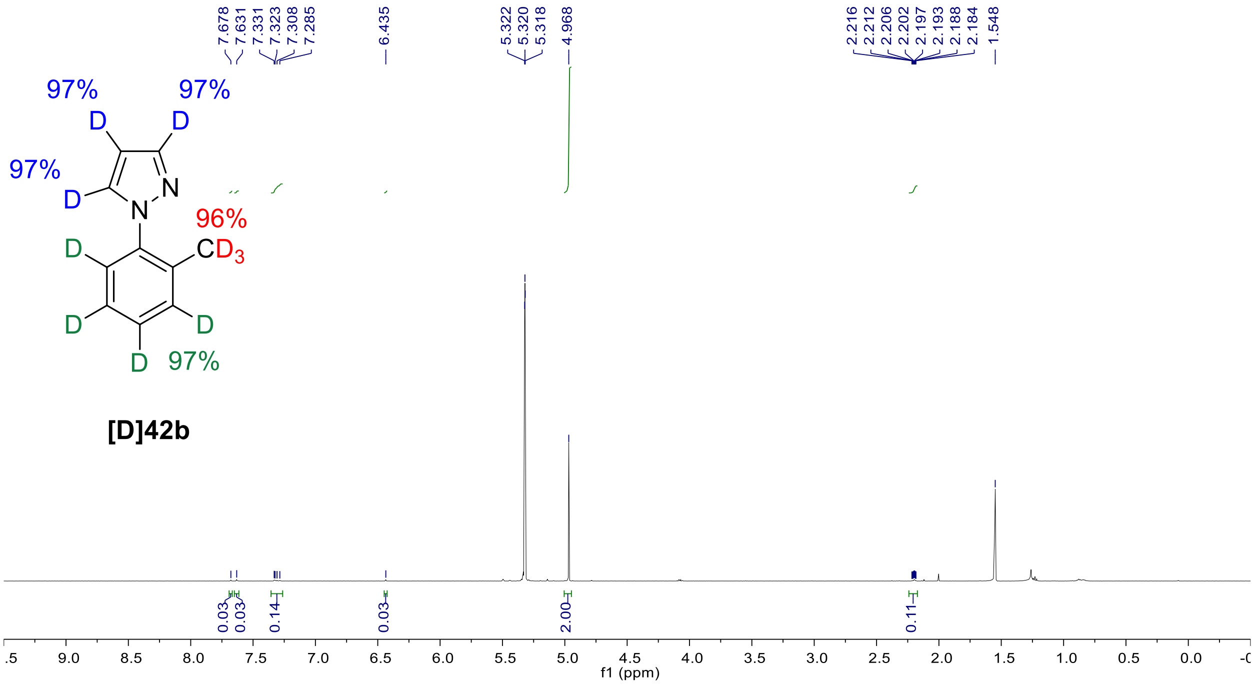

^{1}H NMR spectrum of compound **[D]42b** (Procedure B, CD_2Cl_2, 500 MHz)

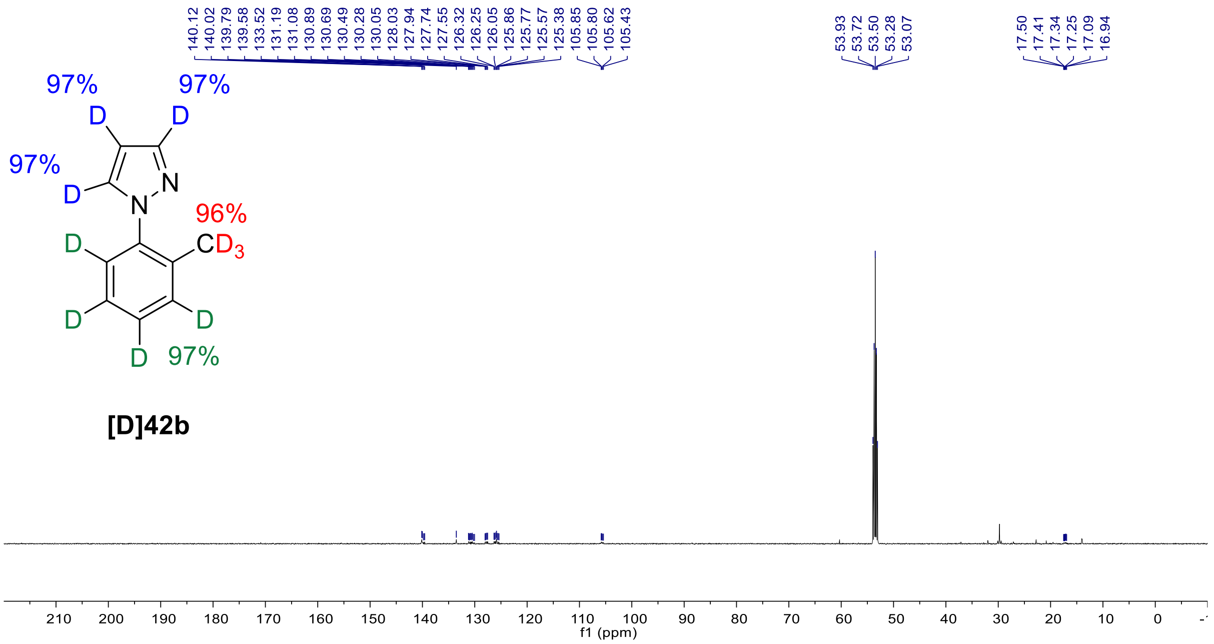

^{13}C NMR spectrum of compound **[D]42b** (Procedure B, CD_2Cl_2, 126 MHz)

2,6-dimethyl-3-(1*H*-pyrazol-1-yl)pyridine (43a)

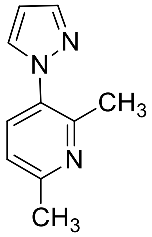

The compound **43a** was prepared following the reported procedure.[1] 3-Bromo-2,3-dimethylpyridine (0.500 mL, 3.85 mmol), CuI (49.5 mg, 0.260 mmol), and Cs_2CO_3 (1.25 g, 3.85 mmol) were added to a solution of 1*H*-pyrazole (175 mg, 2.56 mmol) and DMF (2.60 mL, 1.0 M) in a 40 mL-vial under argon atmosphere. The reaction mixture was stirred in a preheated oil bath at 120 °C. After stirring for 24 h, the reaction mixture was cooled to 25 °C and then treated with ammonium hydroxide solution (25% NH_3 in H_2O, 25.0 mL) and EtOAc (25.0 mL). The solution was transferred to a 125 mL-separatory funnel. The organic layer was collected, and the aqueous layer was extracted with EtOAc (15.0 mL × 3). The combined organic layers were washed with brine (15.0 mL), dried over Na_2SO_4, and filtered. Purification by flash column chromatography (hexanes/EtOAc = 1:1) provided **43a** as a colorless liquid (428 mg, 92% yield). IR (film) 3104, 2925, 1582, 1517, 1478, 753 cm^{-1}; ^1H NMR (300 MHz, $CDCl_3$) δ 7.74 (d, *J* = 1.8 Hz, 1H), 7.60 (d, *J* = 2.4 Hz, 1H), 7.54 (d, *J* = 8.1 Hz, 1H), 7.10 (d, *J* = 8.1 Hz, 1H), 6.47 (t, *J* = 2.1 Hz, 1H), 2.59 (s, 3H), 2.44 (s, 3H); ^{13}C NMR (100 MHz, $CDCl_3$) δ 157.9, 153.2, 140.9, 133.94, 133.87, 130.7, 121.1, 106.9, 24.3, 21.2; HRMS (ESI) calcd for $C_{10}H_{12}N_3$ $[M+H]^+$ 174.1026, found 174.1026.

Following the general procedure B, the reaction was set up with 2,6-dimethyl-3-(1H-pyrazol-1-yl)pyridine (34.6 mg, 0.20 mmol). Purification by flash column chromatography (hexanes/EtOAc=1:1.5) provided product **[D]43a** as a white oil (23 mg, 62% yield).

Deuterium Incorporation

General procedure B: [HRMS (ESI)] calcd for $C_{10}HD_{11}N_3$ $[M+H]^+$ 9.51 D/molecule, [^1H NMR] 9.96 D/molecule.

NMR Data of the Product

General procedure B: ^{1}H NMR (500 MHz, $CDCl_3$) δ 7.75 (d, 0.05H, 95% D), 7.60 (s, 0.08H, 92% D), 7.57 (s, 0.45H, 55% D), 7.13-7.11 (m, 0.07H, 93% D), 6.47 (s, 0.06H, 94% D), 2.58 (s, 0.17H, 94% D), 2.44 (s, 0.16H, 95% D) ;^{13}C NMR (126 MHz, $CDCl_3$) δ 157.8, 153.2, 141.0-140.6 (1C), 134.0 (2C), 130.7-130.3 (1C), 121.3-120.8 (1C), 106.8-106.4 (1C), 24.0-23.2 (1C), 20.9-20.2 (1C).

Mass Data

LabelChecker Results

Formula: C10 H12 N3
Mass (monoisotopic): 174.10

Difference Value: 0.017381
Error Sum: 0.132
Error (%): 0.830

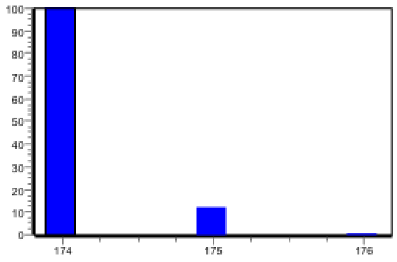

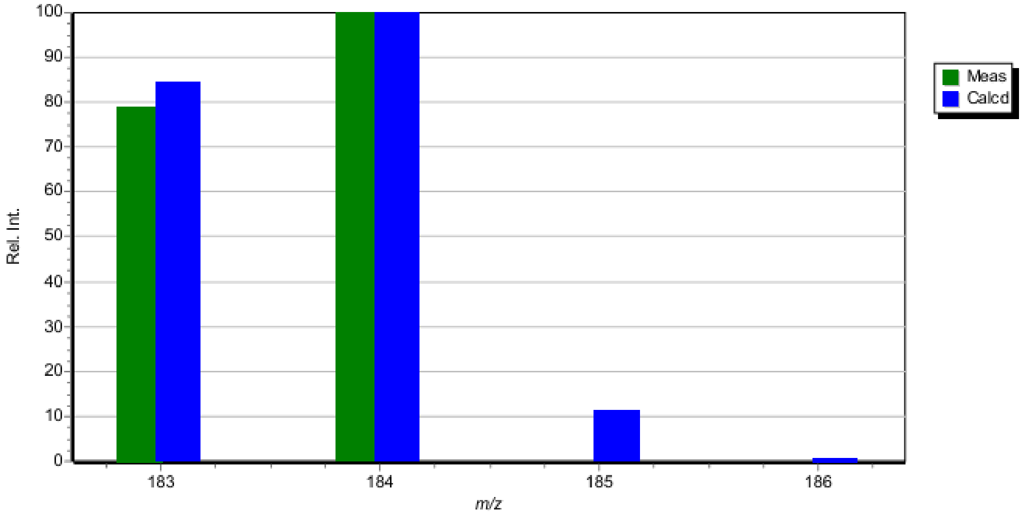

No Convergence! - Max Iterations (3000) reached!
Deuterium: 0-fold (%): 0.00 0.00
Deuterium: 1-fold (%): 0.00 0.00
Deuterium: 2-fold (%): 0.00 0.00
Deuterium: 3-fold (%): 0.00 0.00
Deuterium: 4-fold (%): 0.00 0.00
Deuterium: 5-fold (%): 0.00 0.00
Deuterium: 6-fold (%): 0.00 0.00
Deuterium: 7-fold (%): 0.00 0.00
Deuterium: 8-fold (%): 0.00 0.00
Deuterium: 9-fold (%): 94.25 48.52
Deuterium: 10-fold (%): 100.00 51.48
Deuterium: 11-fold (%): 0.00 0.00
Label Atom Sum: 9.51 (79.29%)

Isotope List used for fitting data:

m/z	intensity
183.16	1714368
183.28	117255
184.16	2164582
184.29	147122

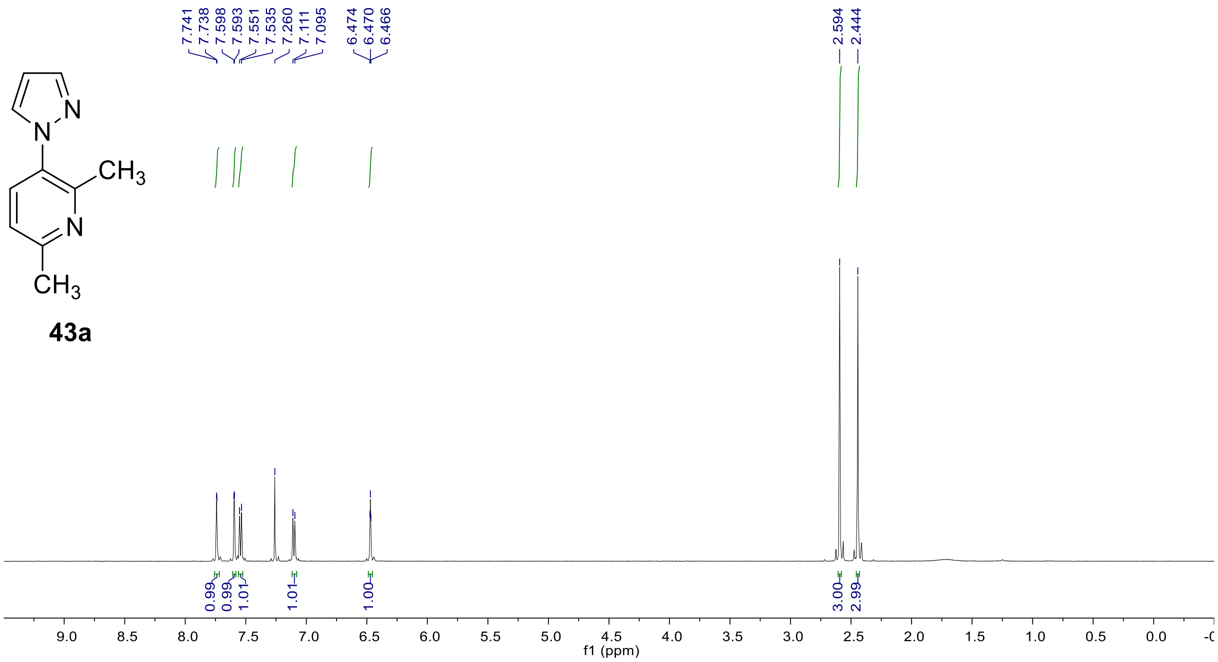

^{1}H NMR spectrum of compound **43a** ($CDCl_3$, 500 MHz)

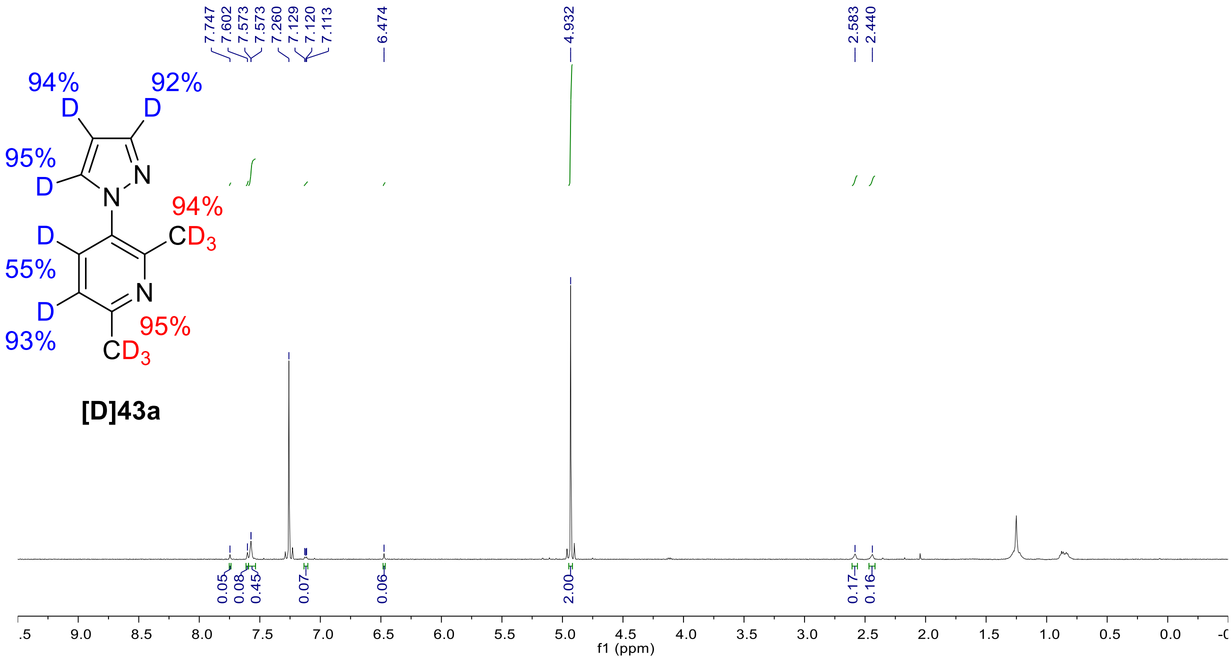

^{1}H NMR spectrum of compound **[D]43a** (Procedure B, $CDCl_3$, 500 MHz)

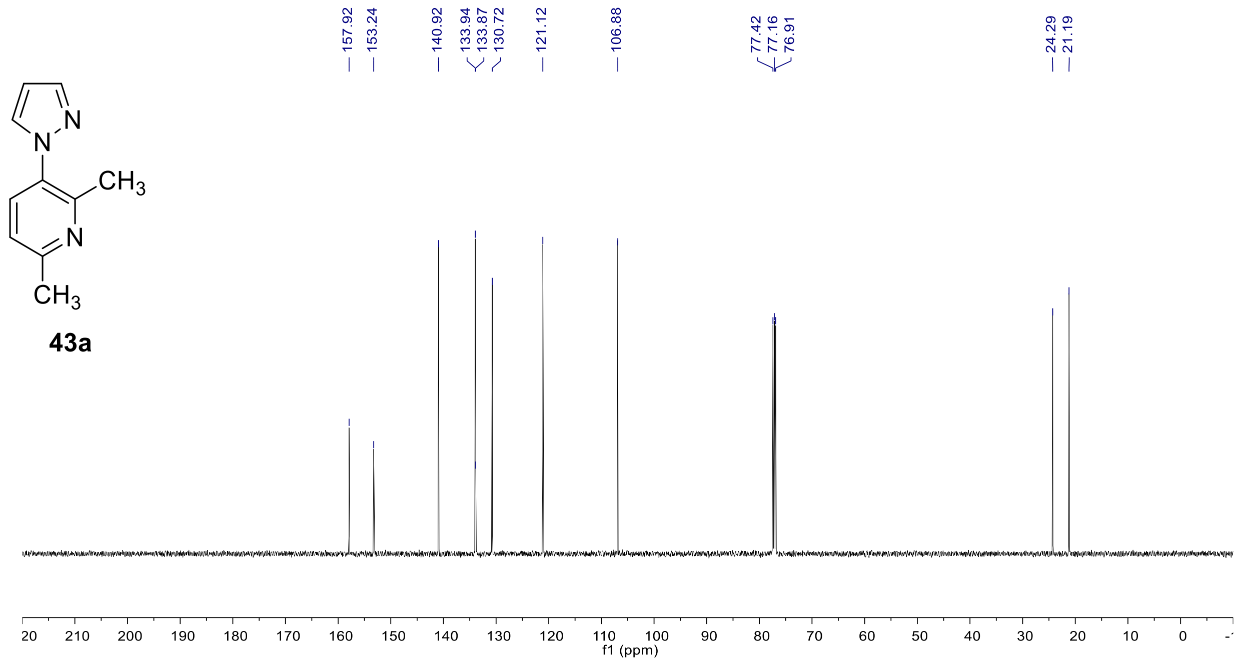

^{13}C NMR spectrum of compound **43a** ($CDCl_3$, 126 MHz)

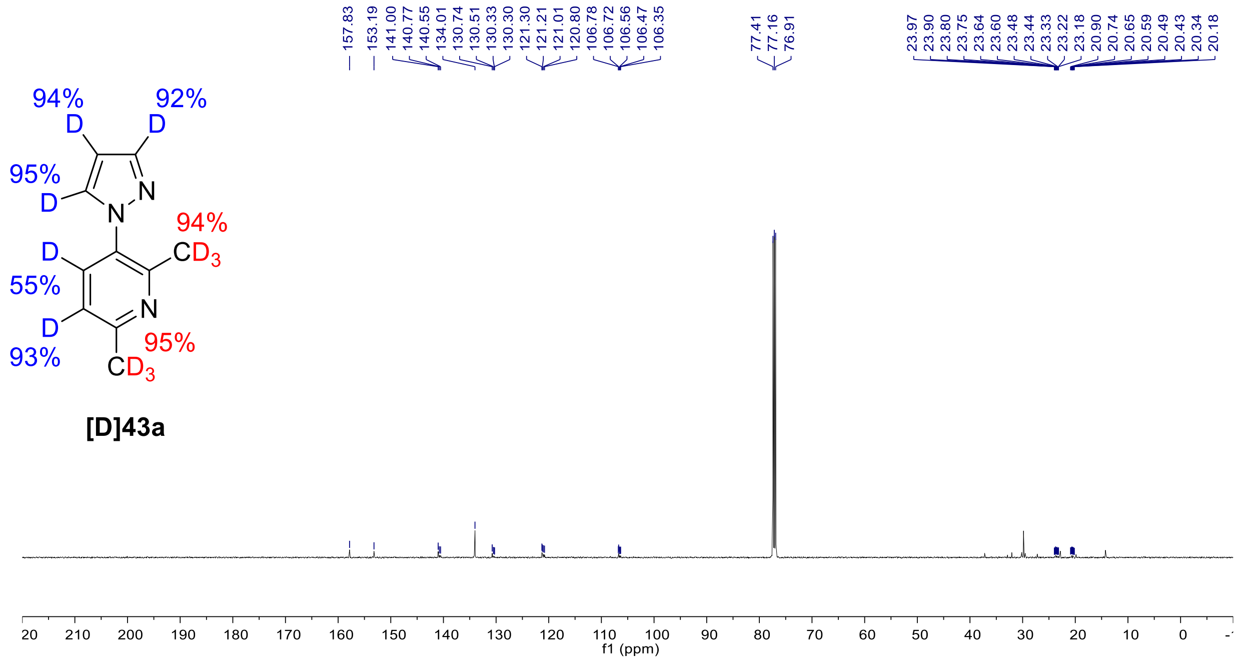

^{13}C NMR spectrum of compound **[D]43a** (Procedure B, $CDCl_3$, 126 MHz)

2,5-dimethyl-3-(1*H*-pyrazol-1-yl)pyrazine (43b)

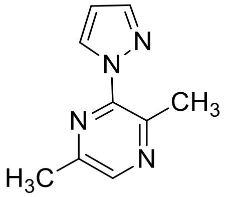

The compound **43b** was prepared following the reported procedure.[1] 3-Chloro-2,5-dimethylpyrazine (0.890 mL, 7.35 mmol), CuI (141 mg, 0.740 mmol), and Cs_2CO_3 (3.58 g, 11.0 mmol) were added to a solution of 1*H*-pyrazole (1.00 g, 14.7 mmol) and DMF (7.50 mL, 1.0 M) in a 40 mL-vial under argon atmosphere. The reaction mixture was stirred in a preheated oil bath at 120 °C. After stirring for 24 h, the reaction mixture was cooled to 25 °C and then treated with ammonium hydroxide solution (25% NH_3 in H_2O, 25.0 mL) and EtOAc (25.0 mL). The solution was transferred to a 125 mL-separatory funnel. The organic layer was collected, and the aqueous layer was extracted with EtOAc (15.0 mL × 3). The combined organic layers were washed with brine (15.0 mL), dried over Na_2SO_4, and filtered. Purification by flash column chromatography (hexanes/EtOAc = 6:1) provided **43b** as a colorless liquid (1.12 g, 87% yield). IR (film) 2926, 2361, 2341, 1518, 1479, 754 cm^{-1}; ^1H NMR (300 MHz, $CDCl_3$) δ 8.33 (s, 1H), 8.27 (d, J = 2.5 Hz, 1H), 7.80-7.76 (m, 1H), 6.50-6.47 (m, 1H), 2.81 (s, 3H), 2.55 (s, 3H). ^{13}C NMR (100 MHz, $CDCl_3$) δ 149.2, 145.7, 143.8, 141.8, 141.6, 129.9, 107.4, 22.7, 20.8; HRMS (ESI) calcd for $C_9H_{11}N_4$ $[M+H]^+$ 175.0978, found 175.0974.

Following the general procedure B, the reaction was set up with 2,5-dimethyl-3-(1H-pyrazol-1-yl)pyrazine (34.8 mg, 0.20 mmol). Purification by flash column chromatography (hexanes/EtOAc=5.5:1) provided product **[D]43b** as a white oil (23 mg, 74% yield).

Deuterium Incorporation

General procedure B: [HRMS (ESI)] calcd for $C_9HD_{10}N_4$ $[M+H]^+$ 7.35 D/molecule, [^1H NMR] 7.66 D/molecule.

NMR Data of the Product

General procedure B: ^1H NMR (500 MHz, $CDCl_3$) δ 8.33 (d, 0.15H, 85% D), 8.28 (s, 0.04H, 96%

D), 7.78 (s, 0.04H, 96% D), 6.49 (s, 0.04H, 96% D), 2.79 (s, 0.12H, 96% D), 2.59-2.52 (m, 1.94H, 35% D); ^{13}C NMR (126 MHz, $CDCl_3$) δ 149.3-149.2 (1C), 145.8, 143.8, 141.8, 141.6-141.3 (1C), 129.8-129.1 (1C), 107.2-106.8 (1C), 22.6-21.9 (1C), 20.8-20.4 (1C).

Mass Data

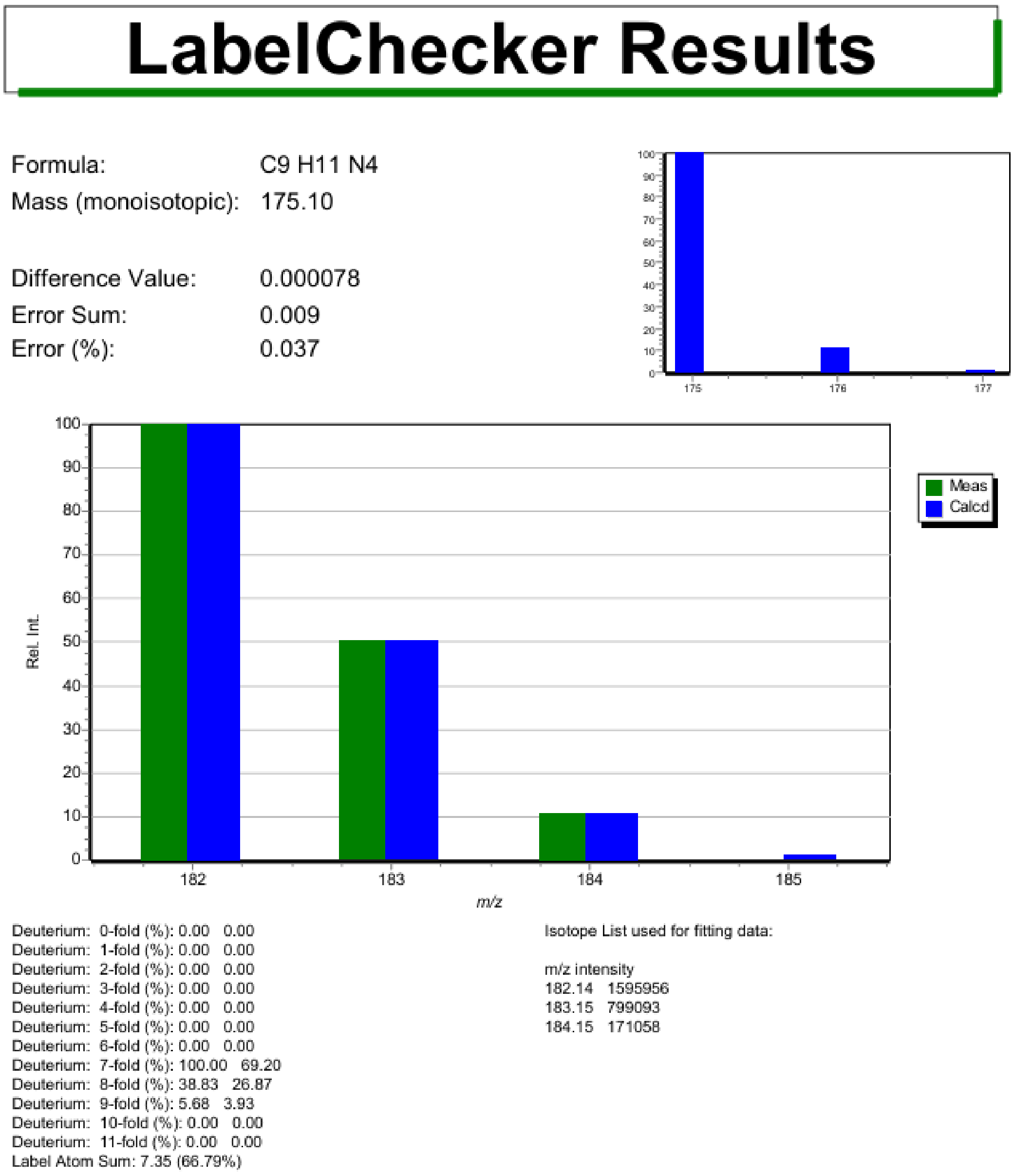

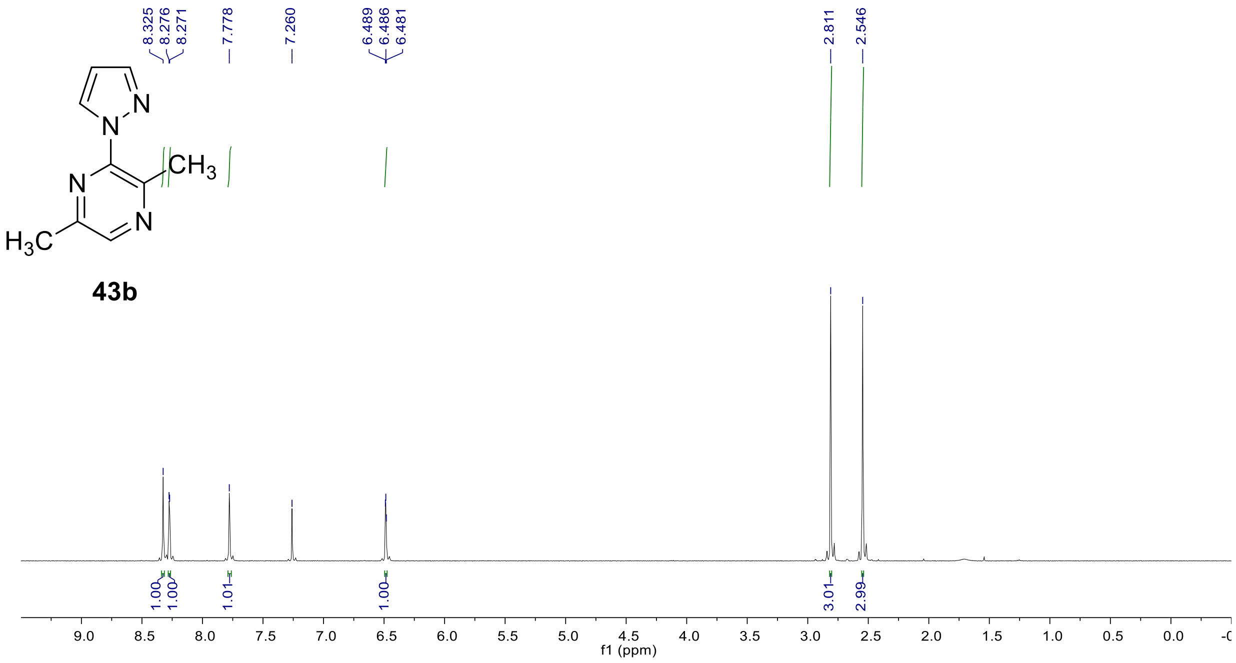

^{1}H NMR spectrum of compound **43b** ($CDCl_3$, 500 MHz)

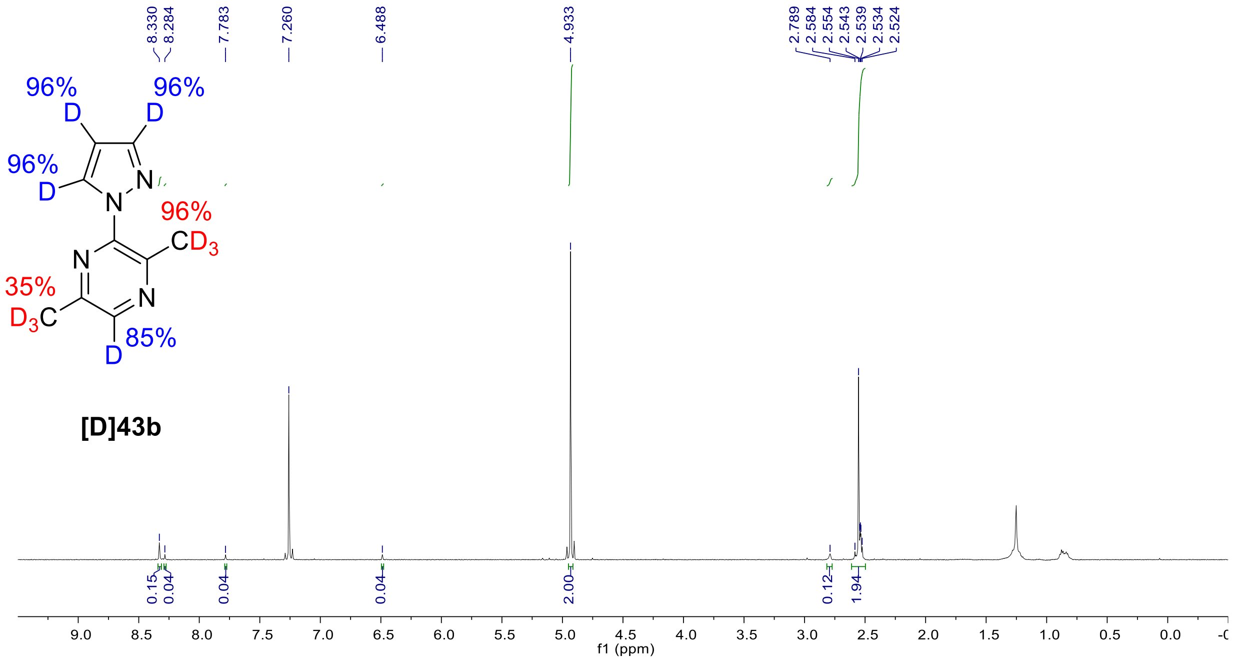

^{1}H NMR spectrum of compound **[D]43b** (Procedure B, $CDCl_3$, 500 MHz)

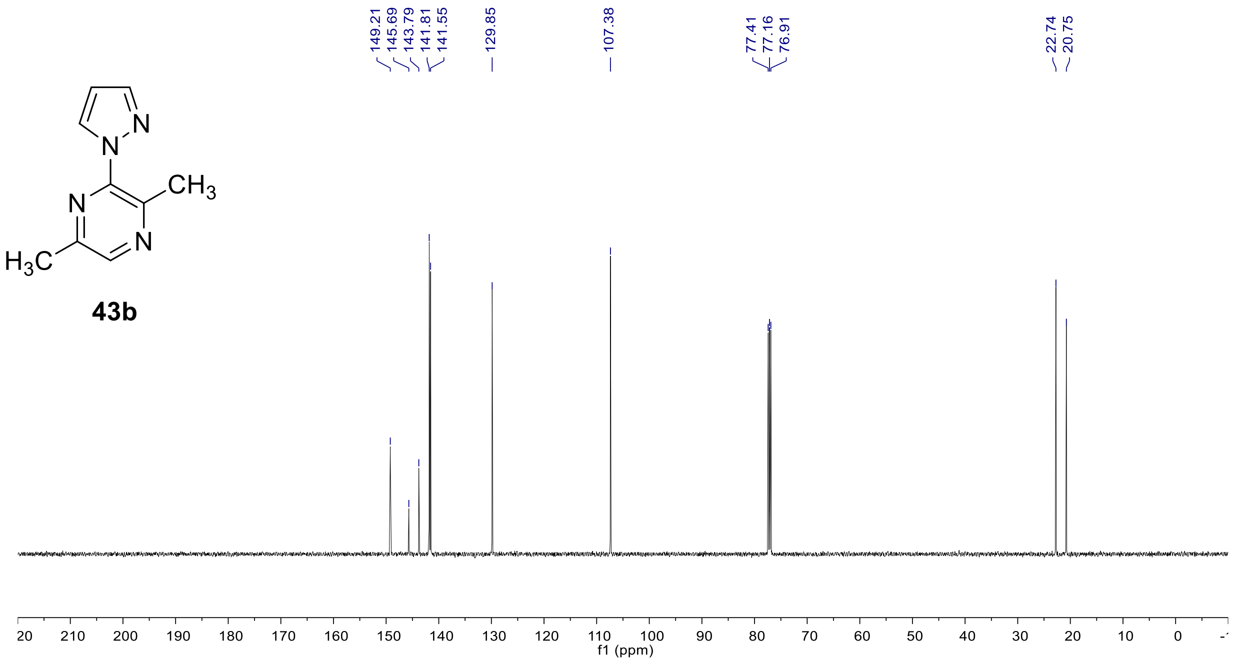

^{13}C NMR spectrum of compound **43b** ($CDCl_3$, 126 MHz)

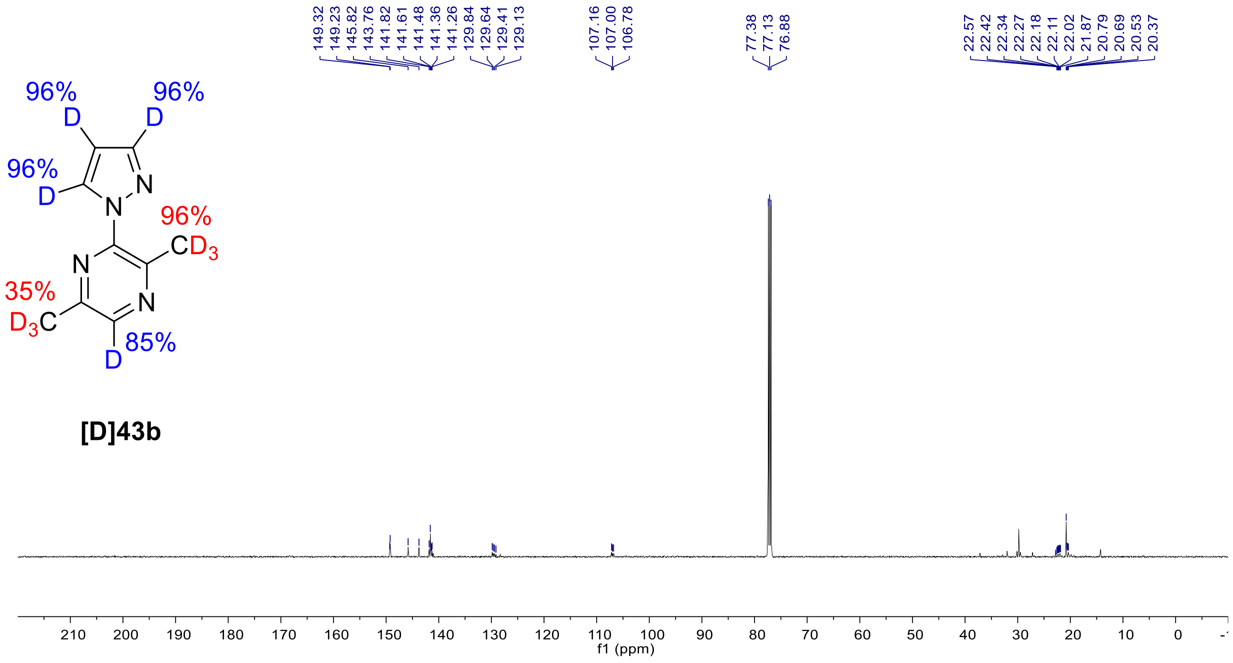

^{13}C NMR spectrum of compound **[D]43b** (Procedure B, $CDCl_3$, 126 MHz)

2-methylbenzenesulfonamide (44)

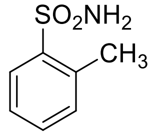

Following the general procedure B, the reaction was set up with 2-methylbenzenesulfonamide (34.2 mg, 0.20 mmol). Purification by flash column chromatography (hexanes/EtOAc = 1:1) provided product **[D]44** as a yellow solid (35 mg, 100% yield).

Deuterium Incorporation

General procedure A: [GCMS (EI)] calcd for $C_7H_2D_7NO_2S$ $[M]^+$ 4.30 D/molecule, [^{1}H NMR] 4.28 D/molecule.

NMR Data of the Starting Material

^{1}H NMR (500 MHz, $(CD_3)_2CO$) δ 7.95 (dd, *J* = 7.9, 1.2 Hz, 1H), 7.48 (t, *J* = 7.5 Hz, 1H), 7.40-7.32 (m, 2H), 6.56 (s, 2H), 2.67 (s, 3H).

NMR Data of the Product

General procedure B: ^{1}H NMR (500 MHz, $(CD_3)_2CO$) δ 7.95 (s, 0.06H, 94% D), 7.48 (s, 0.03H, 97% D), 7.36 (d, *J* = 15.9 Hz, 0.08H, 96% D), 6.56 (s, 2H), 2.69-2.62 (m, 2.56H, 15% D); ^{13}C NMR (126 MHz, $(CD_3)_2CO$) δ 206.4, 142.9, 137.2, 132.9-132.1 (1C), 128.2-127.7 (1C), 126.6-126.1 (1C), 20.2-19.8 (1C).

Mass Data

	M+3	M+4	M+5	M+6	M+7
m/z	174	175	176	177	178
Abound	33755	145479	41695	24795	9339
Theoretical exact mass of start material: 171.0354					
Weighted average of deuterated product: 175.3354					
Average %D: 61%					

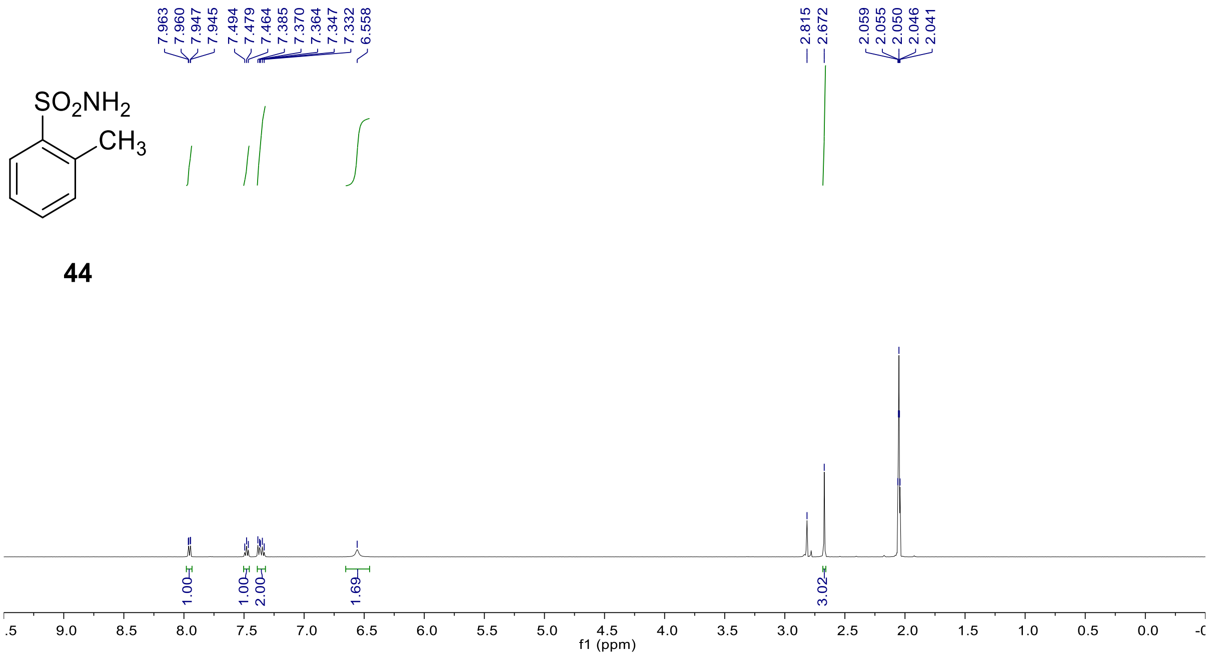

^{1}H NMR spectrum of compound **44** ((CD_3)$_2$CO, 500 MHz)

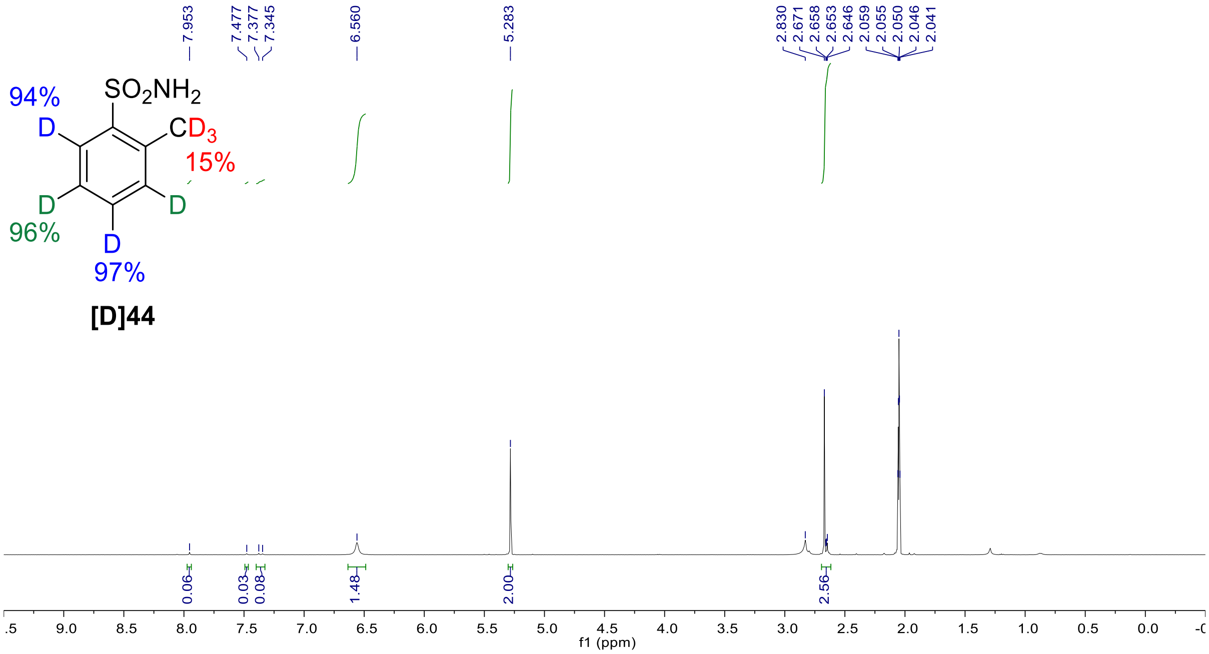

^{1}H NMR spectrum of compound **[D]44** (Procedure B, (CD_3)$_2$CO, 500 MHz)

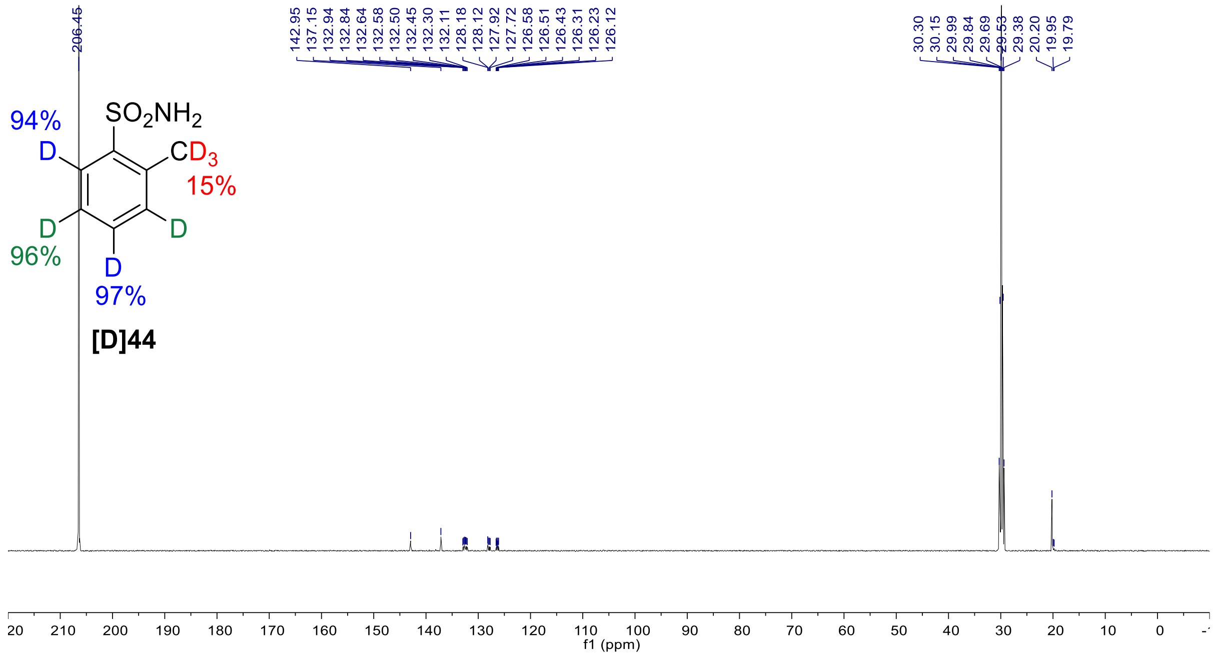

^{13}C NMR spectrum of compound **[D]44** (Procedure B, $(CD_3)_2CO$, 126 MHz)

2-methylbenzophenone (45)

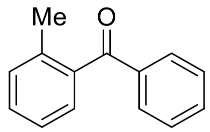

Following the general procedure A, the reaction was set up with 2-methylbenzophenone (39.2 mg, 0.20 mmol). Purification by flash column chromatography (hexanes/EtOAc/AcOH = 10:1:0.08) provided product **[D]45** as a white oil (37 mg, 90% yield).

Deuterium Incorporation

General procedure A: [GCMS (EI)] calcd for $C_{14}D_{12}O$ $[M]^+$ 8.59 D/molecule, [^{1}H NMR] 8.95 D/molecule.

NMR Data of Starting Material

^{1}H NMR (500 MHz, CD_2Cl_2) δ 7.78 (dd, *J* = 8.3, 1.2 Hz, 2H), 7.60 (td, *J* = 7.4, 1.3 Hz, 1H), 7.49-7.45 (m, 2H), 7.49 (td, *J* = 7.4, 1.6 Hz, 1H), 7.32-7.24 (m, 3H), 2.31 (s, 3H).

NMR Data of Product

^{1}H NMR (500 MHz, CD_2Cl_2) δ 7.77 (s, 0.09H, 96% D), 7.60 (s, 0.04H, 96% D), 7.47 (s, 0.09H, 96% D), 7.41 (s, 0.04H, 96% D), 7.30 (d, *J* = 6.1 Hz, 0.11H, 95% D), 7.26 (s, 0.04H, 96% D), 2.30 (s, 2.66H, 11% D).

^{13}C NMR (126 MHz, CD_2Cl_2) δ 198.3, 138.6, 137.7, 136.7, 132.9-132.4 (1C), 130.9-130.3 (1C), 130.0-129.4 (1C), 128.4-127.7 (2C), 125.0-124.5 (2C), 19.7.

Mass Data

	M+8	M+9	M+10	M+11
m/z	204	205	206	207
Abound	520464	612478	96907	14275
Theoretical exact mass of start material: 196.0888				
Weighted average of deuterated product: 204.6825				
Average %D: 72%				

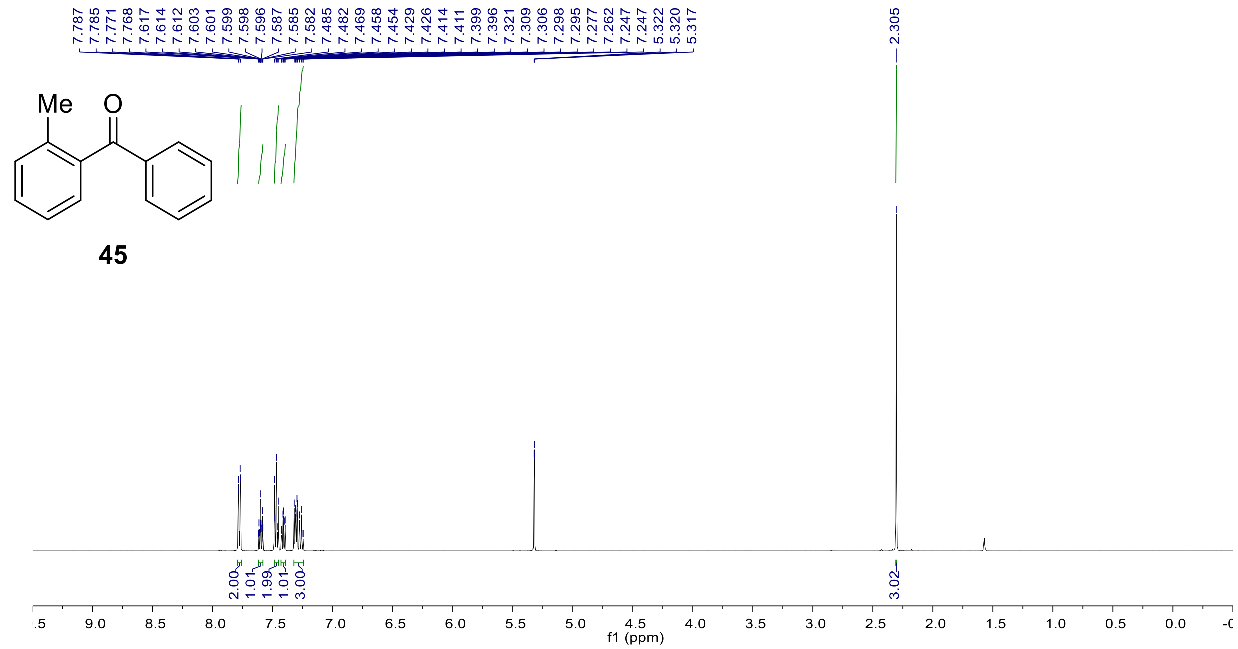

^{1}H NMR spectrum of compound **45** (CD_2Cl_2, 500 MHz)

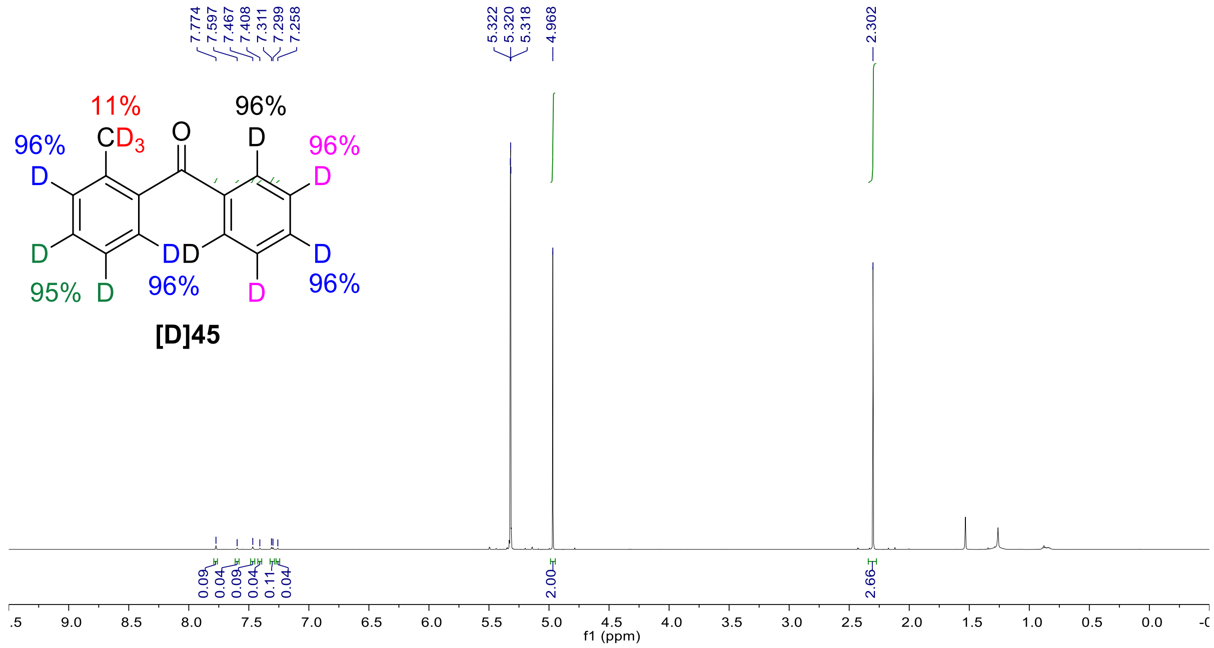

^{1}H NMR spectrum of compound **[D]45** (Procedure A, CD_2Cl_2, 500 MHz)

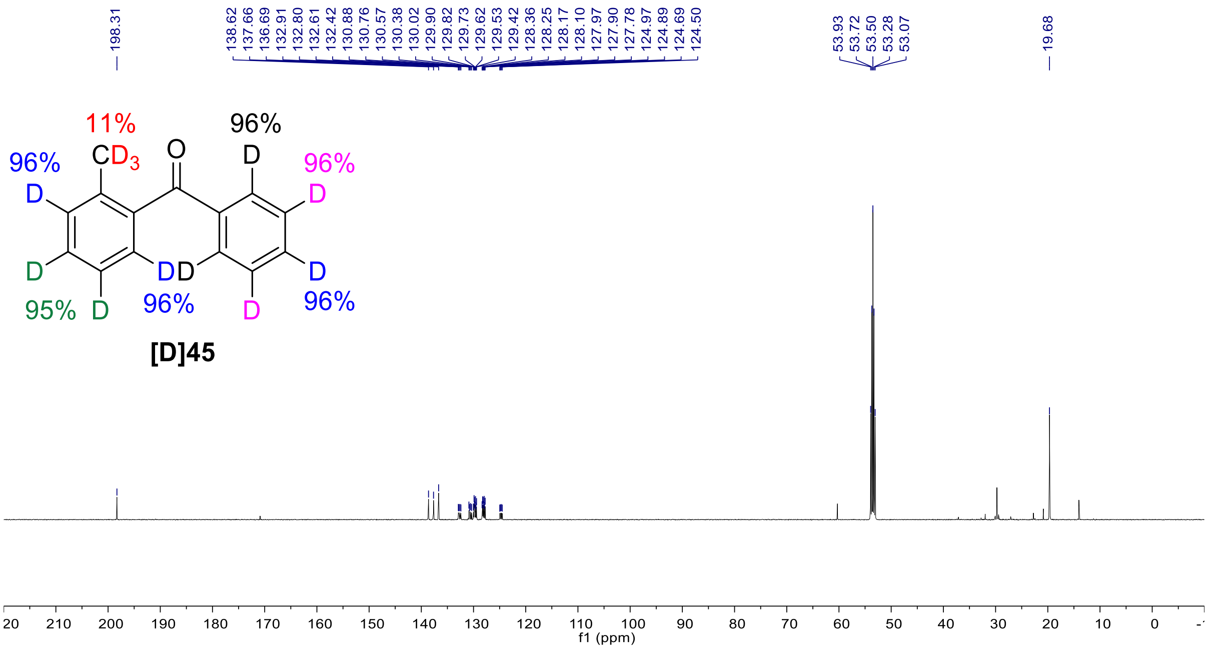

^{13}C NMR spectrum of compound **[D]45** (Procedure A, CD_2Cl_2, 126 MHz)

methyl 4-bromo-2,6-dimethylbenzoate (46)

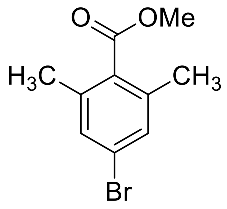

Following the general procedure A, the reaction was set up with methyl 4-bromo-2,6-dimethylbenzoate[10] (48.6 mg, 0.20 mmol). Purification by flash column chromatography (hexanes/EtOAc/HCO_2H = 10:1:0.1) provided product **[D]46** as a white solid (48 mg, 98% yield).

Deuterium Incorporation

General procedure A: [LCMS (ESI)] calcd for $C_{10}H_9D_2O_2Br$ $[M]^+$ 2.00 D/molecule, [^{1}H NMR] 1.94 D/molecule.

NMR Data of the Starting Material

^{1}H NMR (500 MHz, $CDCl_3$) δ 7.20 (s, 2H), 3.90 (s, 3H), 2.28 (s, 6H).

NMR Data of the Product

General procedure A: ^{1}H NMR (500 MHz, $CDCl_3$) δ 7.20 (s, 0.06H, 97% D), 3.90 (s, 2.96H, 1% D), 2.28 (s, 5.94H, 1% D); ^{13}C NMR (126 MHz, $CDCl_3$) δ 169.8, 137.3, 132.8 (2C), 130.6-130.1 (1C), 123.2 (2C), 52.2, 19.7 (2C).

Mass Data

LabelChecker Results

Formula: C10 H11 O2 Br
Mass (monoisotopic): 241.99

Difference Value: 2.834439
Error Sum: 1.684
Error (%): 0.219

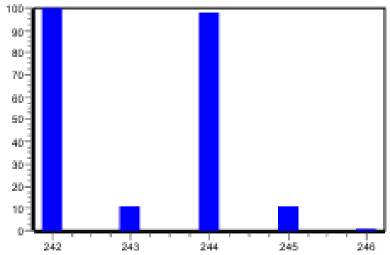

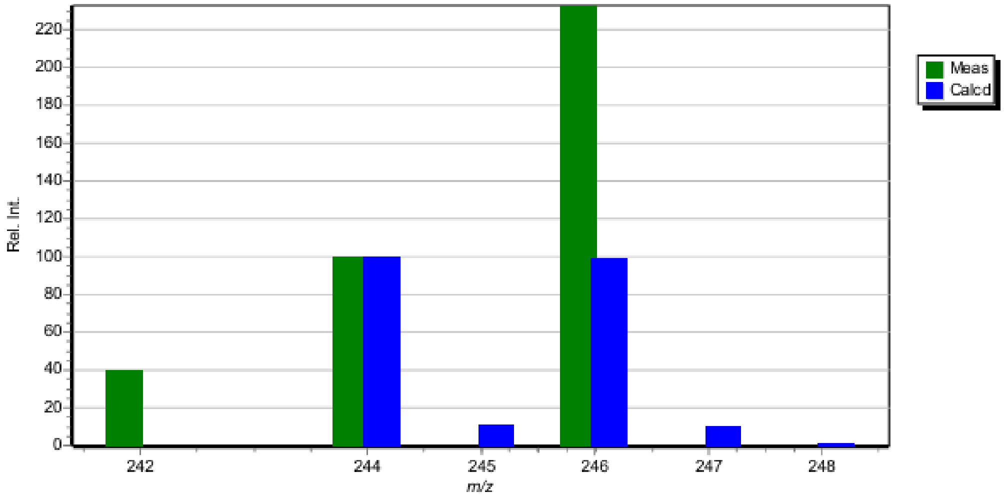

No Convergence! - Max Cycles (20) reached!
Deuterium: 0-fold (%): 0.00 0.00
Deuterium: 1-fold (%): 0.00 0.00
Deuterium: 2-fold (%): 100.00 100.00
Label Atom Sum: 2.00 (18.18%)

Isotope List used for fitting data:

m/z	intensity
242.28	1240899
244.26	3135903
246.24	7300269

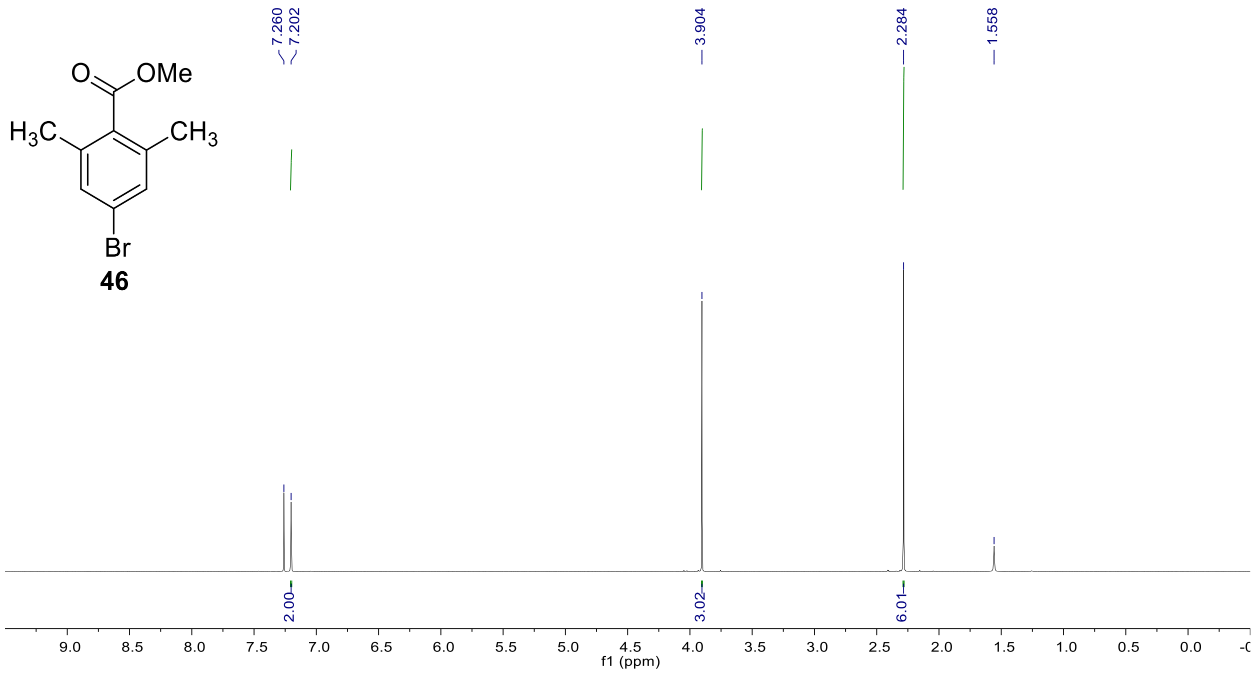

^{1}H NMR spectrum of compound **46** ($CDCl_3$, 500 MHz)

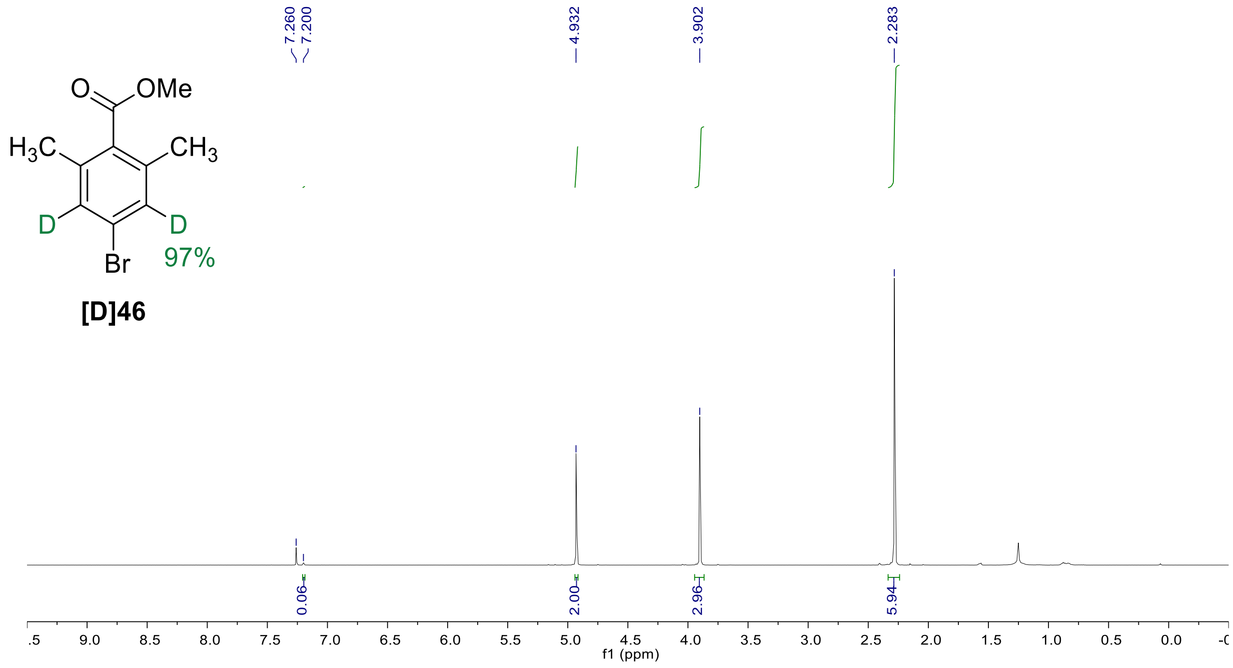

^{1}H NMR spectrum of compound **[D]46** (Procedure A, $CDCl_3$, 500 MHz)

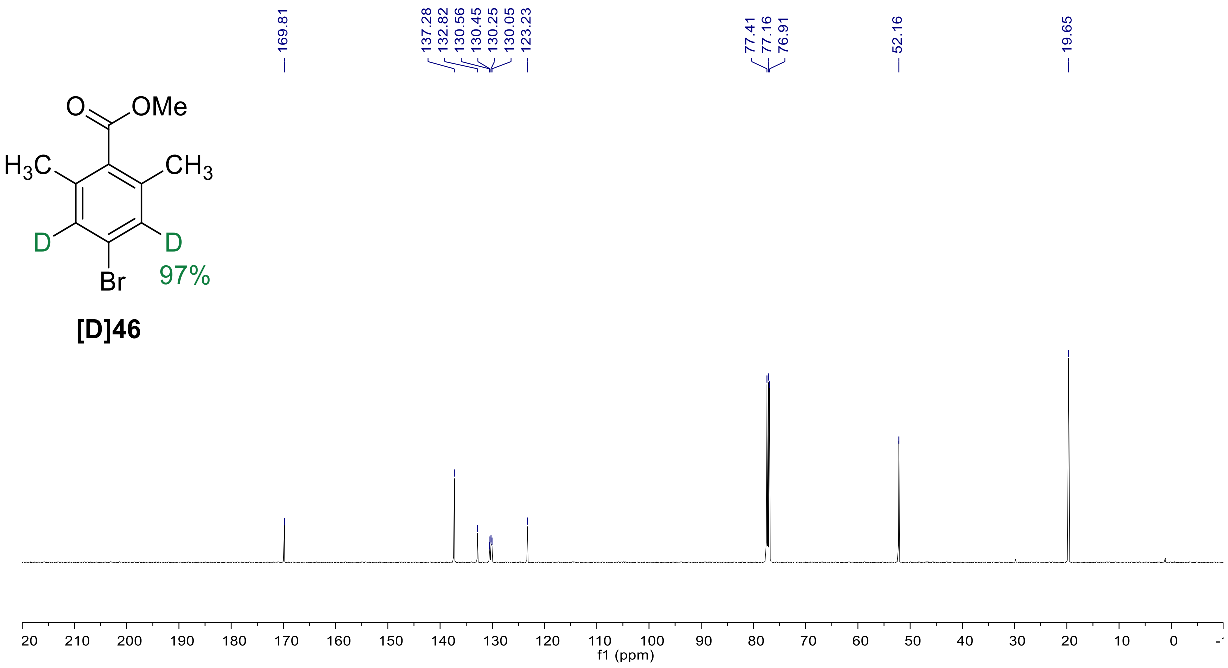

^{13}C NMR spectrum of compound **[D]46** (Procedure A, $CDCl_3$, 126 MHz)

2-methyl-1-nitronaphthalene (47)

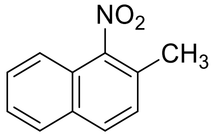

Following the general procedure B, the reaction was set up with 2-methyl-1-nitronaphthalene (37.4 mg, 0.20 mmol). Purification by flash column chromatography (hexanes/EtOAc = 15:1) provided product **[D]47** as an orange solid (38 mg, 98% yield).

Deuterium Incorporation

General procedure B: [GCMS (EI)] calcd for $C_{11}H_3D_6NO_2$ $[M]^+$ 5.28 D/molecule, [^{1}H NMR] 5.42 D/molecule.

NMR Data of the Starting Material

^{1}H NMR (500 MHz, $CDCl_3$) δ 7.88 (dd, *J* = 8.1, 2.4 Hz, 2H), 7.74-7.70 (m, 1H), 7.61 (ddd, *J* = 8.5, 6.9, 1.3 Hz, 1H), 7.54 (ddd, *J* = 8.1, 6.8, 1.2 Hz, 1H), 7.37 (d, *J* = 8.4 Hz, 1H), 2.51 (s, 3H).

NMR Data of the Product

General procedure B: ^{1}H NMR (500 MHz, $CDCl_3$) δ 7.88 (s, 0.07H, 97% D), 7.73 (s, 0.43H, 57% D), 7.62-7.59 (m, 0.03H, 97% D), 7.54 (s, 0.03H, 97% D), 7.36 (s, 0.03H, 97% D), 2.51 (s, 3H); ^{13}C NMR (126 MHz, $CDCl_3$) δ 147.9, 132.2, 130.4-129.9 (1C), 128.4-128.2 (1C), 128.1-127.4 (3C), 126.5-126.0 (1C), 124.8-124.7 (1C), 121.3-120.7 (1C), 17.9.

Mass Data

	M+3	M+4	M+5	M+6	M+7
m/z	190	191	192	193	194
Abound	16379	118851	418788	393243	51122
Theoretical exact mass of start material: 187.0633					
Weighted average of deuterated product: 192.3444					
Average %D: 88%					

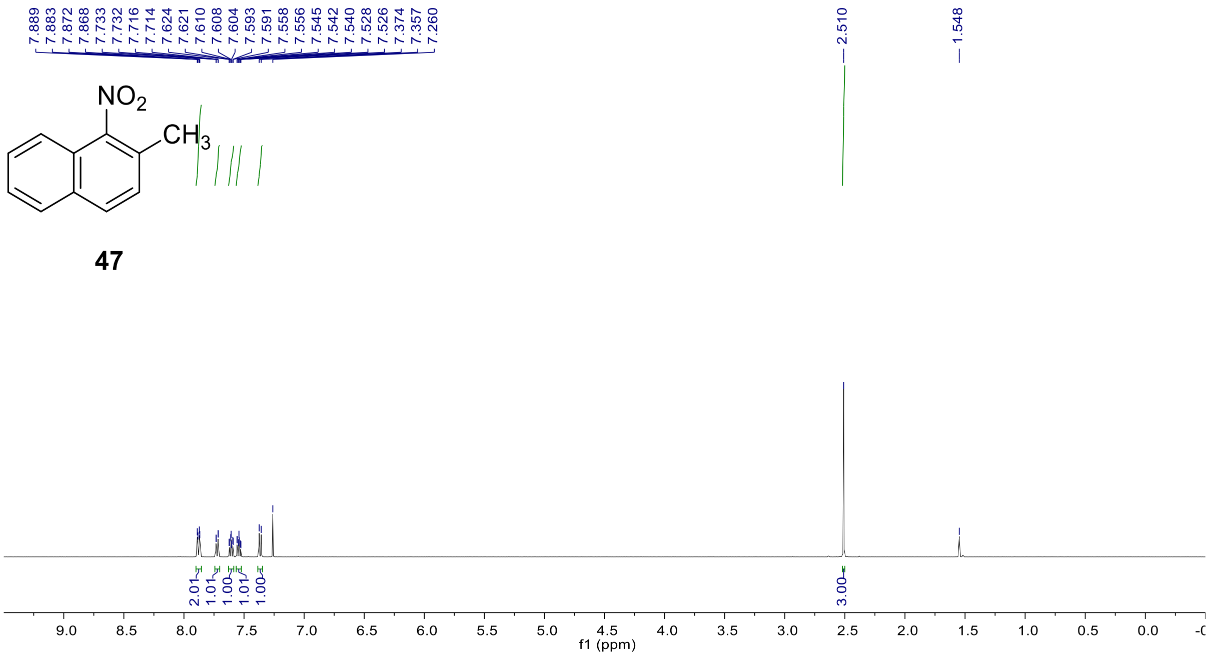

^{1}H NMR spectrum of compound **47** ($CDCl_3$, 500 MHz)

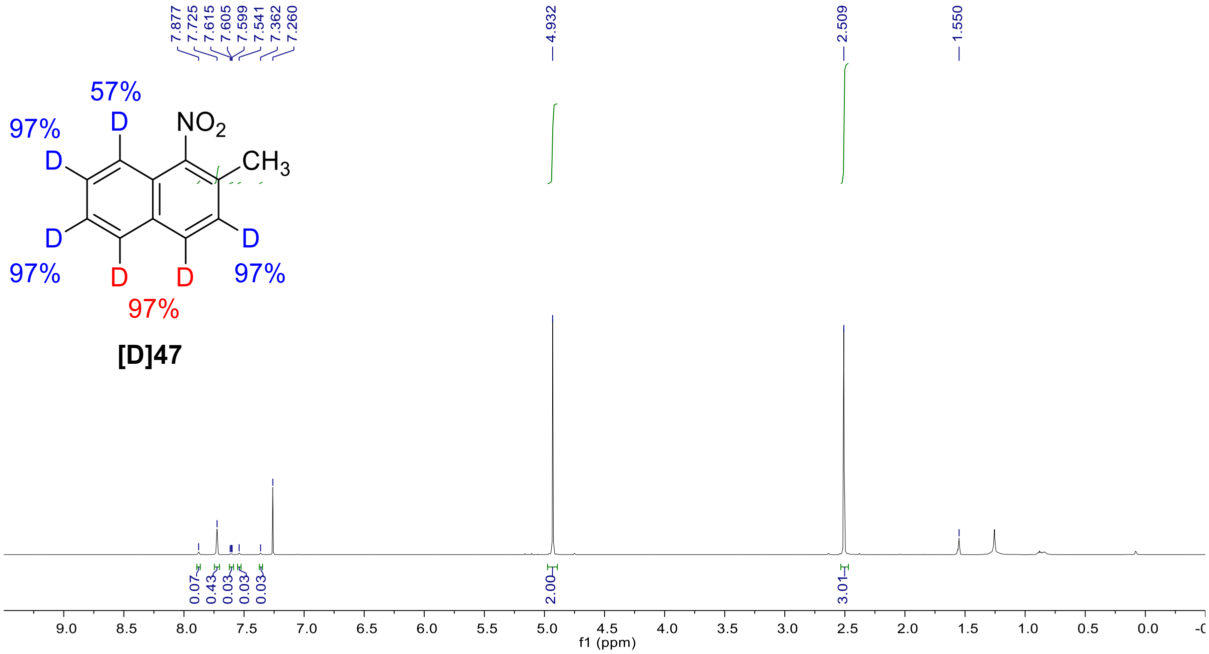

^{1}H NMR spectrum of compound **[D]47** (Procedure B, $CDCl_3$, 500 MHz)

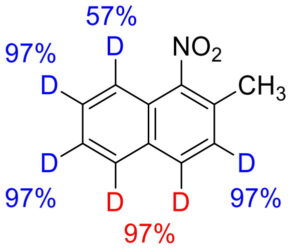

[D]47

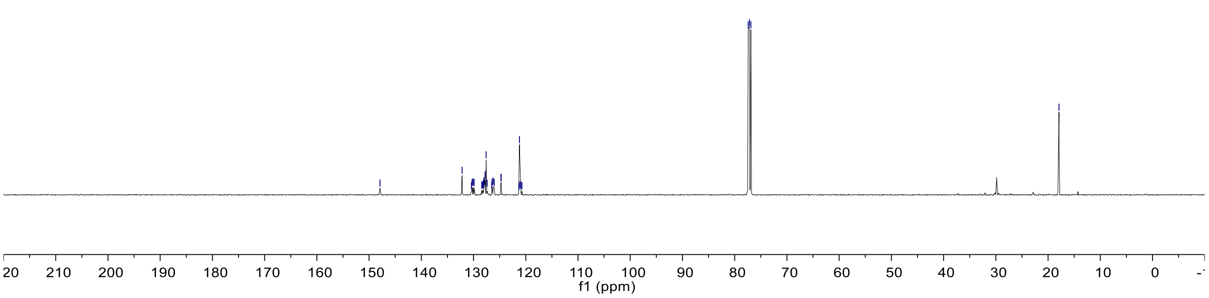

^{13}C NMR spectrum of compound **[D]47** (Procedure B, $CDCl_3$, 126 MHz)

2-nitro-1,1'-biphenyl (48)

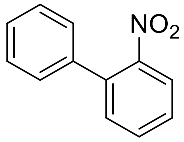

Following the general procedure A, the reaction was set up with 2-nitro-1,1'-biphenyl (39.8 mg, 0.20 mmol). Purification by flash column chromatography (hexanes/EtOAc = 15:1) provided product **[D]48** as a yellow liquid (35 mg, 84% yield).

Deuterium Incorporation

General procedure A: [GCMS (EI)] calcd for $C_{12}D_9NO_2$ $[M]^+$ 8.02 D/molecule, [^{1}H NMR] 8.39 D/molecule.

NMR Data of the Starting Material

^{1}H NMR (500 MHz, $CDCl_3$) δ 7.86 (dd, *J* = 8.1, 1.3 Hz, 1H), 7.62 (td, *J* = 7.6, 1.3 Hz, 1H), 7.51-7.41 (m, 5H), 7.34-7.32 (m, 2H).

NMR Data of the Product

General procedure A: ^{1}H NMR (500 MHz, $CDCl_3$) δ 7.86 (s, 0.05H, 95% D), 7.62 (s, 0.05H, 95% D), 7.49-7.41 (m, 0.26H, 95% D), 7.33(s, 0.26H, 87% D); ^{13}C NMR (126 MHz, $CDCl_3$) δ 149.3, 137.4-137.3 (1C), 136.3, 132.2-131.9 (1C), 131.7-131.5 (1C), 128.7-128.4 (2C), 128.3-128.0 (1C), 127.9-127.8 (1C), 127.7-127.4 (2C), 124.1-123.7 (1C).

Mass Data

	M+5	M+6	M+7	M+8	M+9	M+10
m/z	204	205	206	207	208	209
Abound	7050	28834	73777	136076	188096	25716
Theoretical exact mass of start material: 199.0633						
Weighted average of deuterated product: 207.0818						
Average %D: 89%						

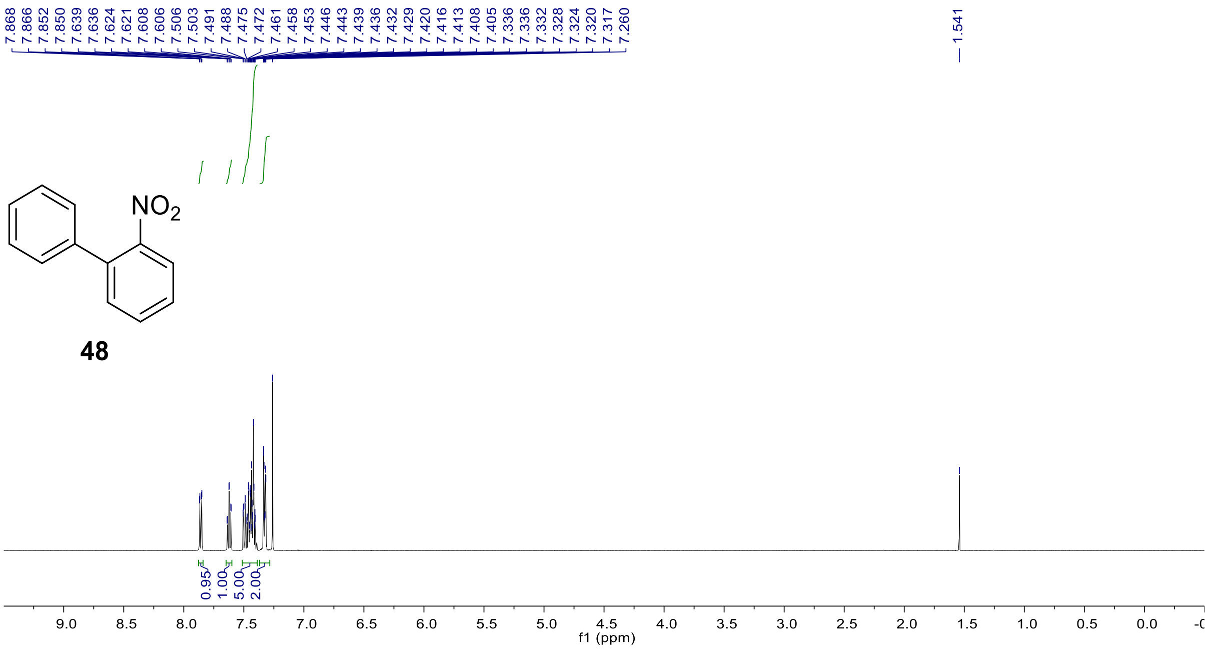

^{1}H NMR spectrum of compound **48** ($CDCl_3$, 500 MHz)

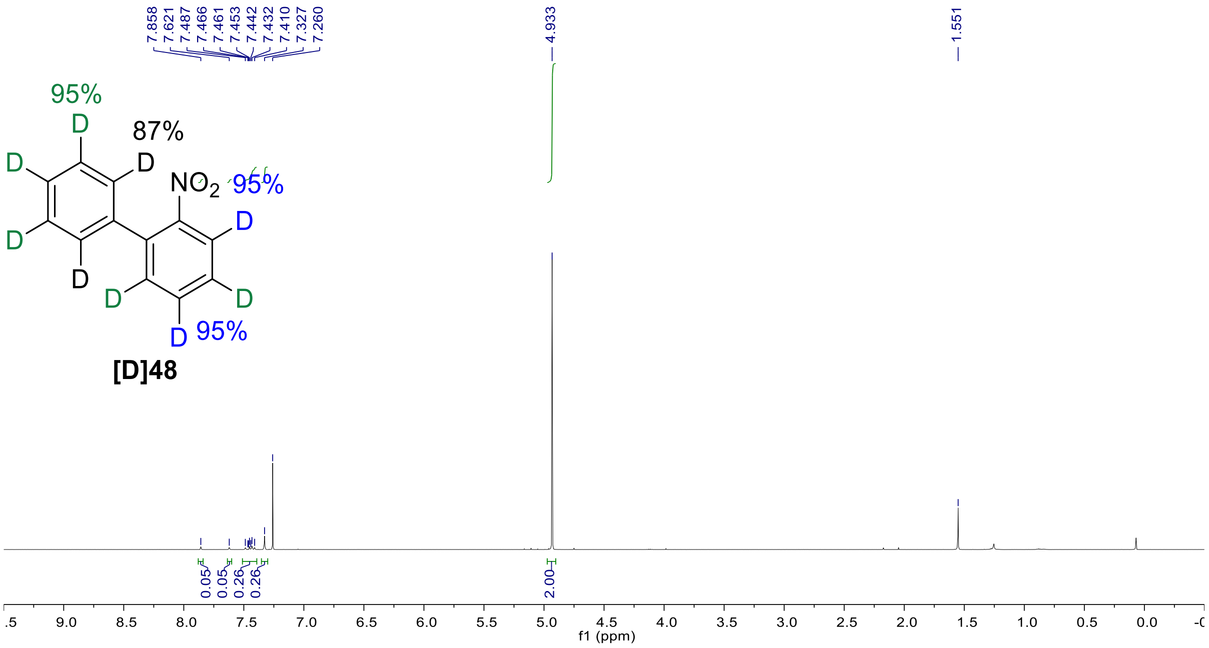

^{1}H NMR spectrum of compound **[D]48** (Procedure A, $CDCl_3$, 500 MHz)

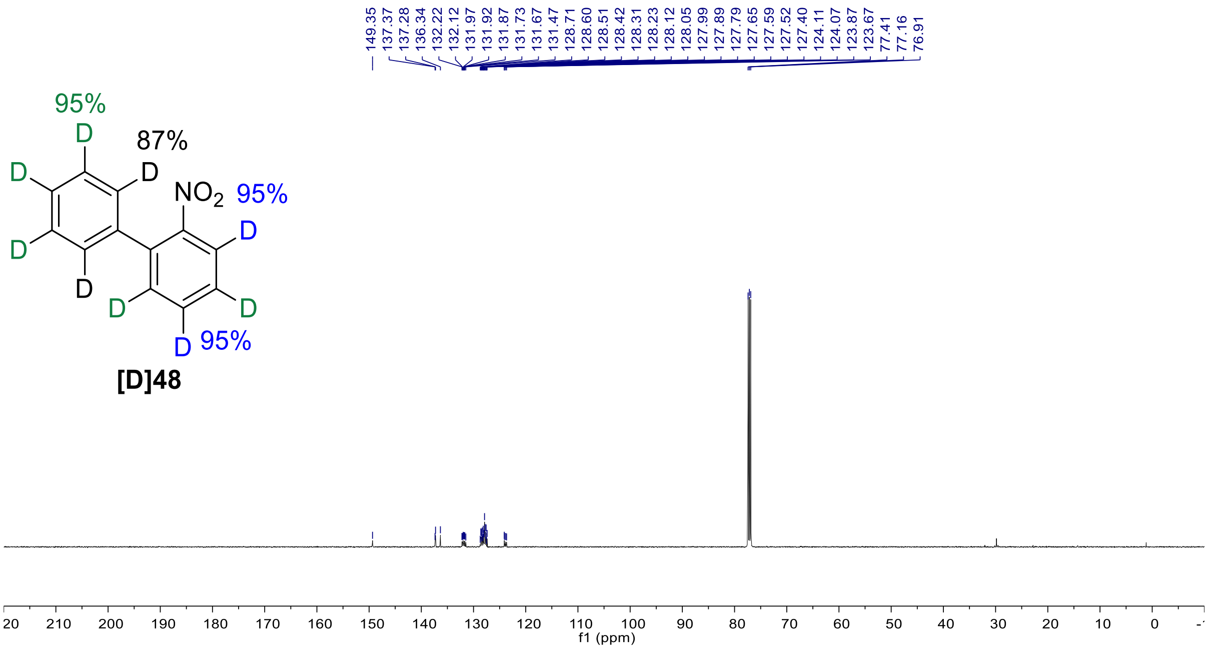

^{13}C NMR spectrum of compound **[D]48** (Procedure A, $CDCl_3$, 126 MHz)

1-nitronaphthalene (49)

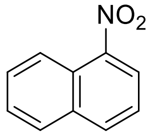

Following the general procedure B, the reaction was set up with 1-nitronaphthalene (34.6 mg, 0.20 mmol). Purification by flash column chromatography (hexanes/EtOAc = 10:1) provided product **[D]49** as a yellow solid (34 mg, 94% yield).

Deuterium Incorporation

General procedure B: [GCMS (EI)] calcd for $C_{10}D_7NO_2$ $[M]^+$ 6.46 D/molecule, [^{1}H NMR] 6.49 D/molecule.

NMR Data of the Starting Material

^{1}H NMR (500 MHz, $CDCl_3$) δ 8.58 (dd, *J* = 8.7, 1.0 Hz, 1H), 8.25 (dd, *J* = 7.6, 1.2 Hz, 1H), 8.14 (d, *J* = 8.2 Hz, 1H), 7.97 (d, *J* = 8.2 Hz, 1H), 7.74 (ddd, *J* = 8.6, 6.9, 1.4 Hz, 1H), 7.64 (ddd, *J* = 8.2, 6.9, 1.1 Hz, 1H), 7.58-7.54 (m, 1H).

NMR Data of the Product

General procedure B: ^{1}H NMR (500 MHz, $CDCl_3$) δ 8.58 (s, 0.33H, 67% D), 8.25 (s, 0.03H, 97% D), 8.14 (s, 0.03H, 97% D), 7.97 (s, 0.03H, 97% D), 7.73 (s, 0.03H, 97% D), 7.63 (s, 0.03H, 97% D), 7.55 (s, 0.03H, 97% D); ^{13}C NMR (126 MHz, $CDCl_3$) δ 146.5, 134.7-134.5 (1C), 134.3-134.1 (1C), 129.5-128.9 (1C), 128.6-128.0 (1C), 127.2-126.7 (1C), 125.2-125.1 (1C), 124.0-123.5 (2C), 123.1-122.6 (1C).

Mass Data

	M+4	M+5	M+6	M+7	M+8
m/z	177	178	179	180	181
Abound	10095	52111	222731	329226	37403
Theoretical exact mass of start material: 173.0477					
Weighted average of deuterated product: 179.5091					
Average %D: 92%					

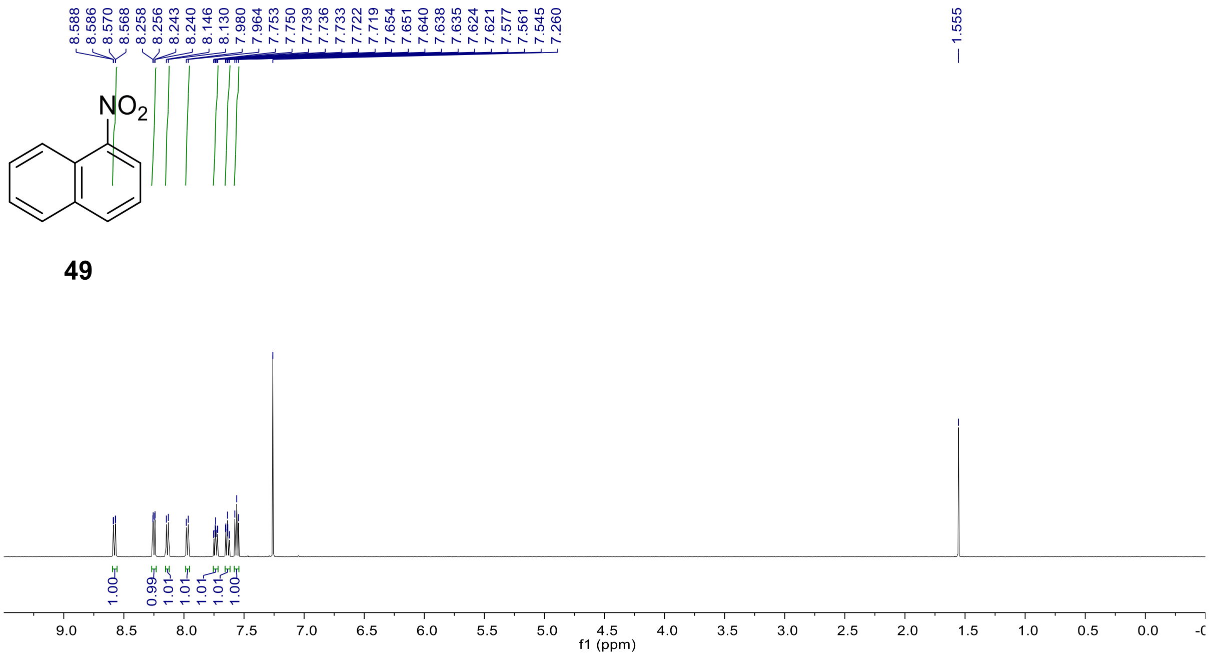

^{1}H NMR spectrum of compound **49** ($CDCl_3$, 500 MHz)

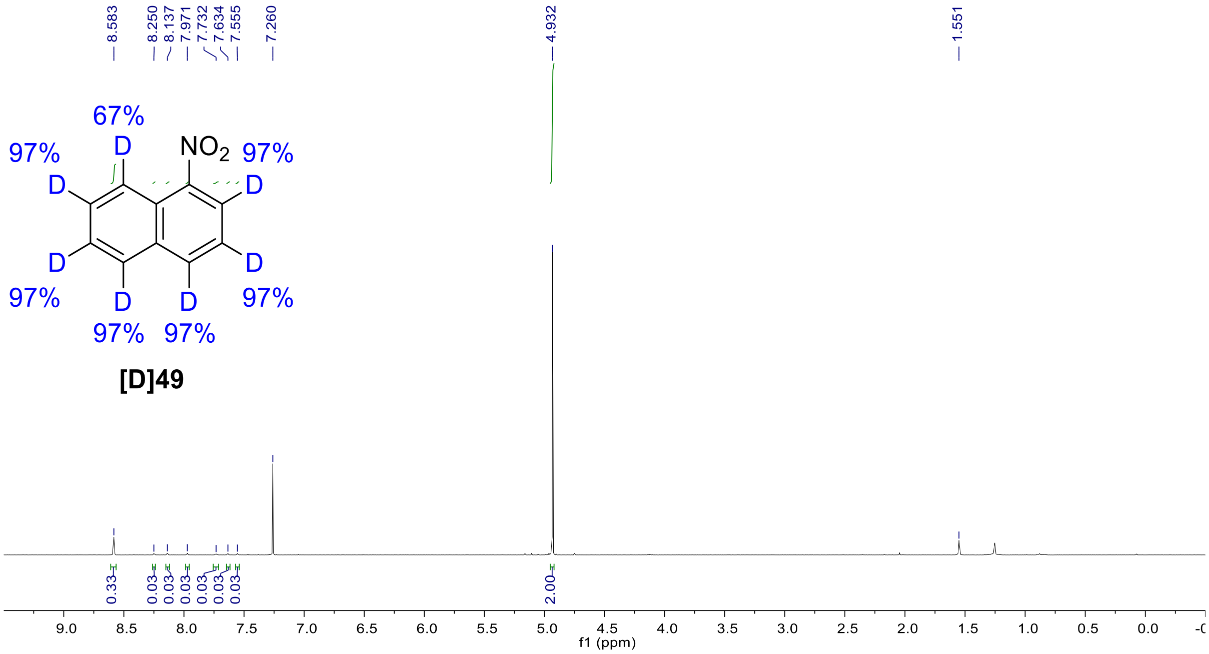

^{1}H NMR spectrum of compound **[D]49** (Procedure B, $CDCl_3$, 500 MHz)

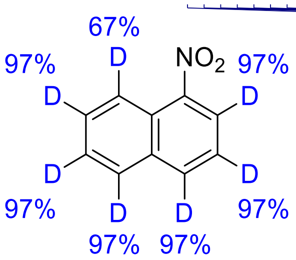

[D]49

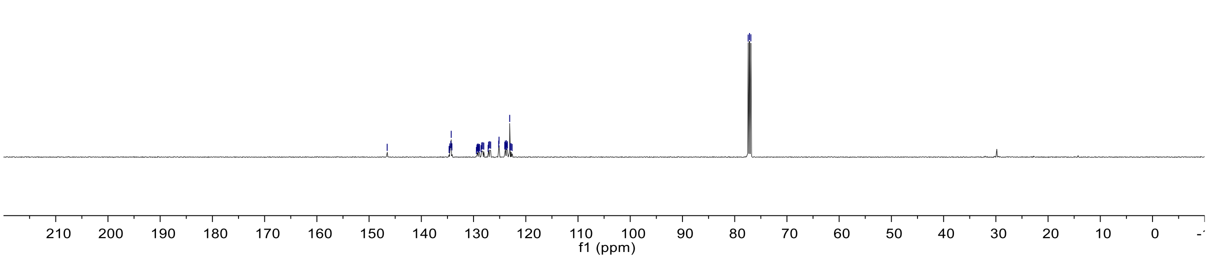

^{13}C NMR spectrum of compound **[D]49** (Procedure B, $CDCl_3$, 126 MHz)

1-methyl-5-nitro-1*H*-indole (50)

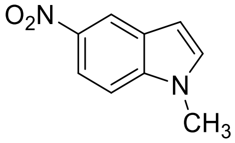

Following the general procedure B, the reaction was set up with 1-methyl-5-nitro-1*H*-indole (35.2 mg, 0.20 mmol). Purification by flash column chromatography (hexanes/EtOAc = 5:1) provided product **[D]50** as a yellow solid (32 mg, 88% yield).

Deuterium Incorporation

General procedure B: [GCMS (EI)] calcd for $C_9H_3D_5N_2O_2$ $[M]^+$ 4.84 D/molecule, [^{1}H NMR] 4.84 D/molecule.

NMR Data of the Starting Material

^{1}H NMR (500 MHz, $CDCl_3$) δ 8.59 (d, *J* = 2.2 Hz, 1H), 8.13 (dd, *J* = 9.1, 2.2 Hz, 1H), 7.34 (d, *J* = 9.1, 1H), 7.21 (d, *J* = 3.2 Hz, 1H), 6.67 (dd, *J* = 3.2, 0.9 Hz, 1H), 3.86 (s, 3H).

NMR Data of the Product

General procedure B: ^{1}H NMR (500 MHz, $CDCl_3$) δ 8.59 (s, 0.03H, 97% D), 8.13 (s, 0.03H, 97% D), 7.34 (s, 0.03H, 97% D), 7.21 (s, 0.03H, 97% D), 6.67 (s, 0.04H, 96% D), 3.87 (s, 2.83H, 6% D); ^{13}C NMR (126 MHz, $CDCl_3$) δ 141.5, 139.5, 132.1-131.8 (1C), 127.5, 118.2-117.8 (1C), 117.2-116.8 (1C), 109.0-108.7 (1C), 103.7-103.3 (1C), 33.4.

Mass Data

	M+3	M+4	M+5	M+6
m/z	179	180	181	182
Abound	15850	167972	820790	90954
Theoretical exact mass of start material: 176.0586				
Weighted average of deuterated product: 180.9008				
Average %D: 97%				

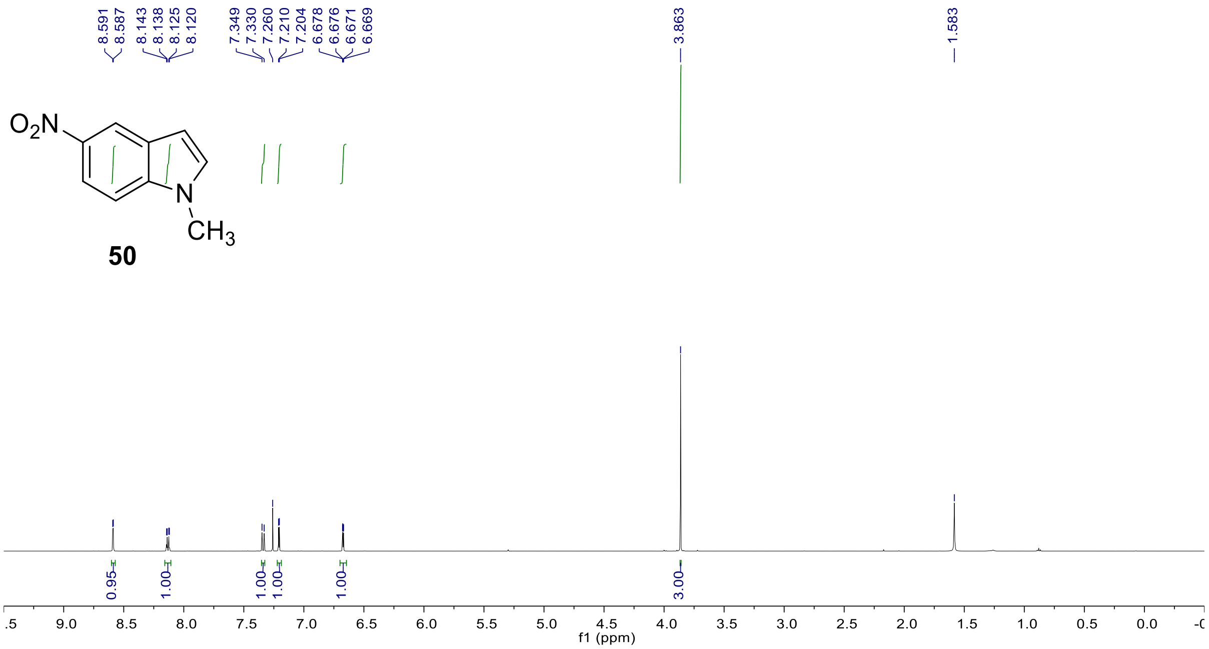

^{1}H NMR spectrum of compound **50** ($CDCl_3$, 500 MHz)

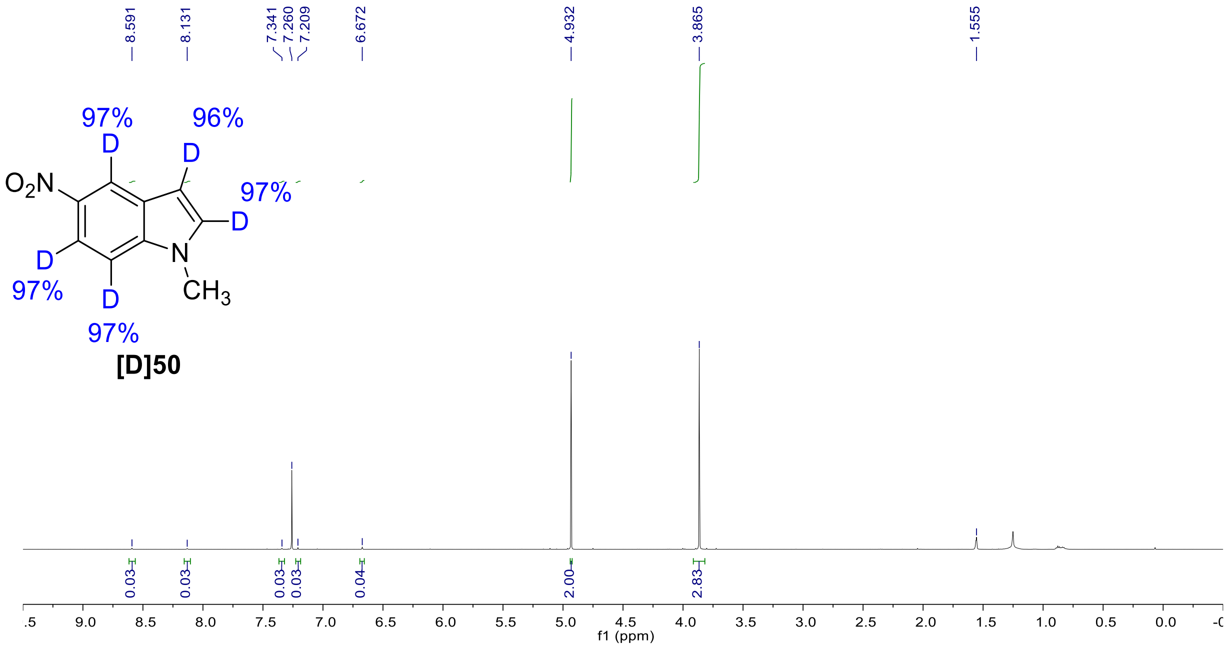

^{1}H NMR spectrum of compound **[D]50** (Procedure B, $CDCl_3$, 500 MHz)

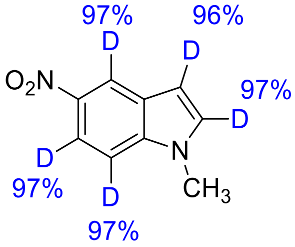

[D]50

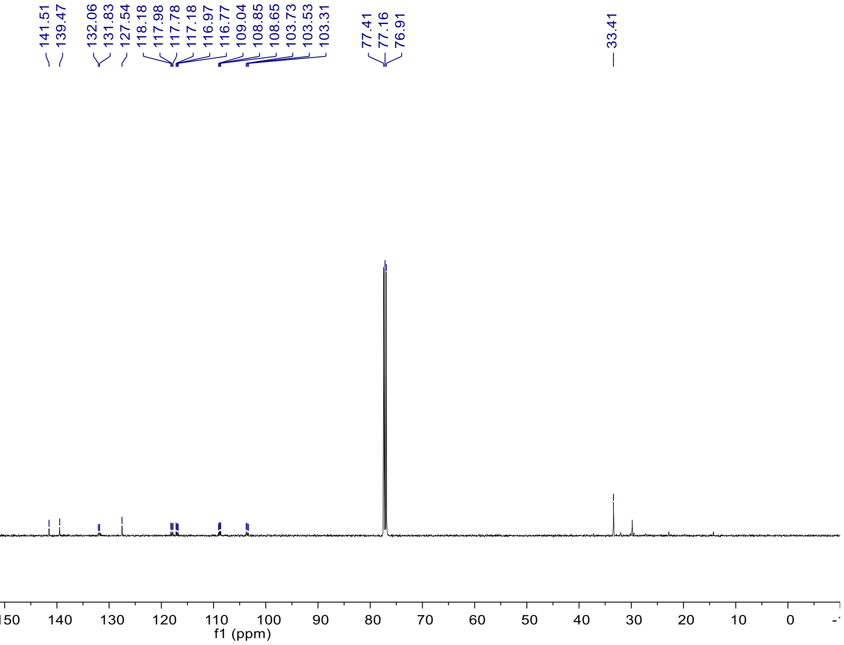

^{13}C NMR spectrum of compound **[D]50** (Procedure B, $CDCl_3$, 126 MHz)

4-nitrobenzo[*c*][1,2,5]thiadiazole (51)

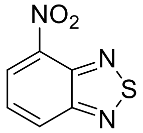

Following the general procedure A, the reaction was set up with 4-nitrobenzo[*c*] [1,2,5]thiadiazole (36.2 mg, 0.20 mmol). Purification by flash column chromatography (hexanes/EtOAc = 5:1) provided product **[D]51** as a yellow solid (36 mg, 98% yield).

Deuterium Incorporation

General procedure A: [GCMS (EI)] calcd for $C_6D_3N_3O_2S$ $[M]^+$ 2.84 D/molecule, [^{1}H NMR] 2.79 D/molecule.

NMR Data of the Starting Material

^{1}H NMR (500 MHz, $CDCl_3$) δ 8.59 (dd, *J* = 7.5, 1.0 Hz, 1H), 8.41 (dd, *J* = 8.8, 1.0 Hz, 1H), 7.80 (dd, *J* = 8.8, 7.5 Hz, 1H).

NMR Data of the Product

General procedure A: ^{1}H NMR (500 MHz, $CDCl_3$) δ 8.60 (s, 0.15H, 85% D), 8.41 (s, 0.03H, 97% D), 7.80 (s, 0.03H, 97% D); ^{13}C NMR (126 MHz, $CDCl_3$) δ 156.3, 146.6, 139.9, 128.3-128.1 (1C), 127.3, 126.9.

Mass Data

	M+1	M+2	M+3	M+4
m/z	182	183	184	185
Abound	9735	165234	555193	56651
Theoretical exact mass of start material: 180.9946				
Weighted average of deuterated product: 183.8373				
Average %D: 95%				

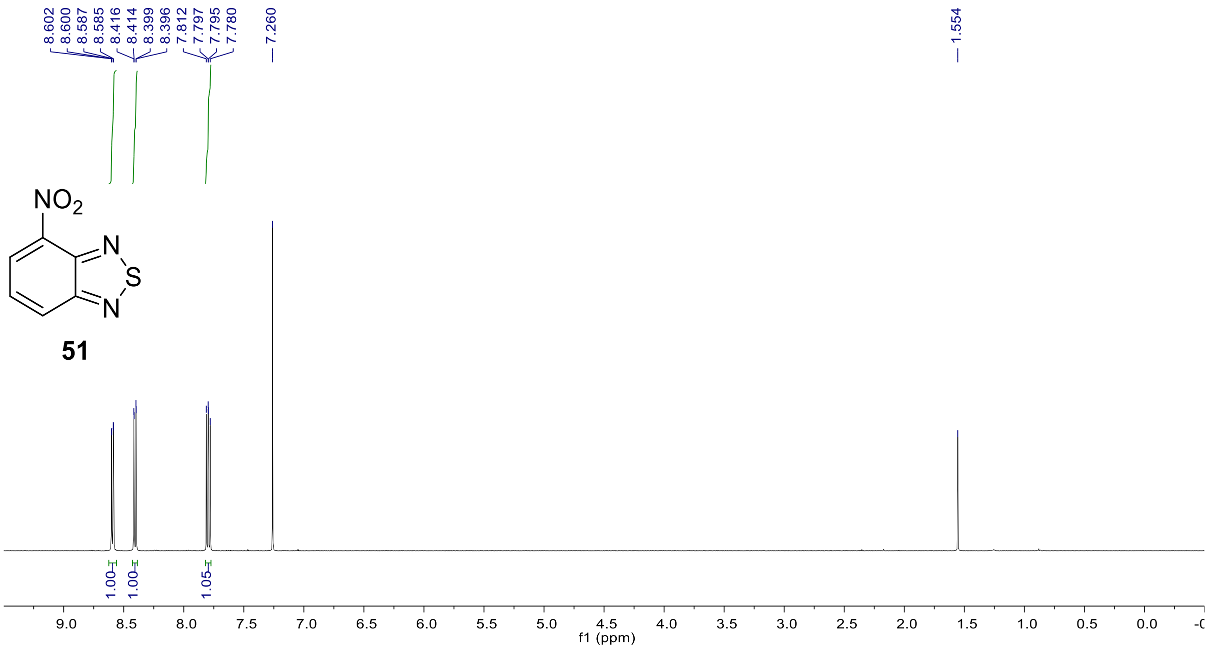

^{1}H NMR spectrum of compound **51** ($CDCl_3$, 500 MHz)

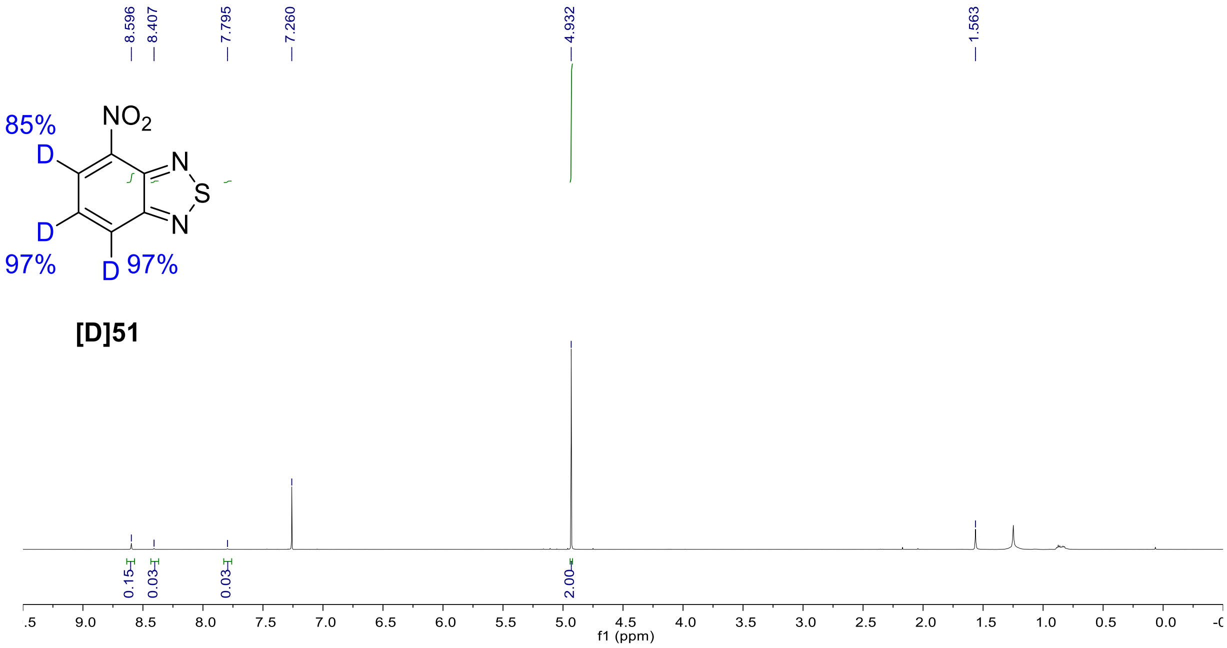

^{1}H NMR spectrum of compound **[D]51** (Procedure A, $CDCl_3$, 500 MHz)

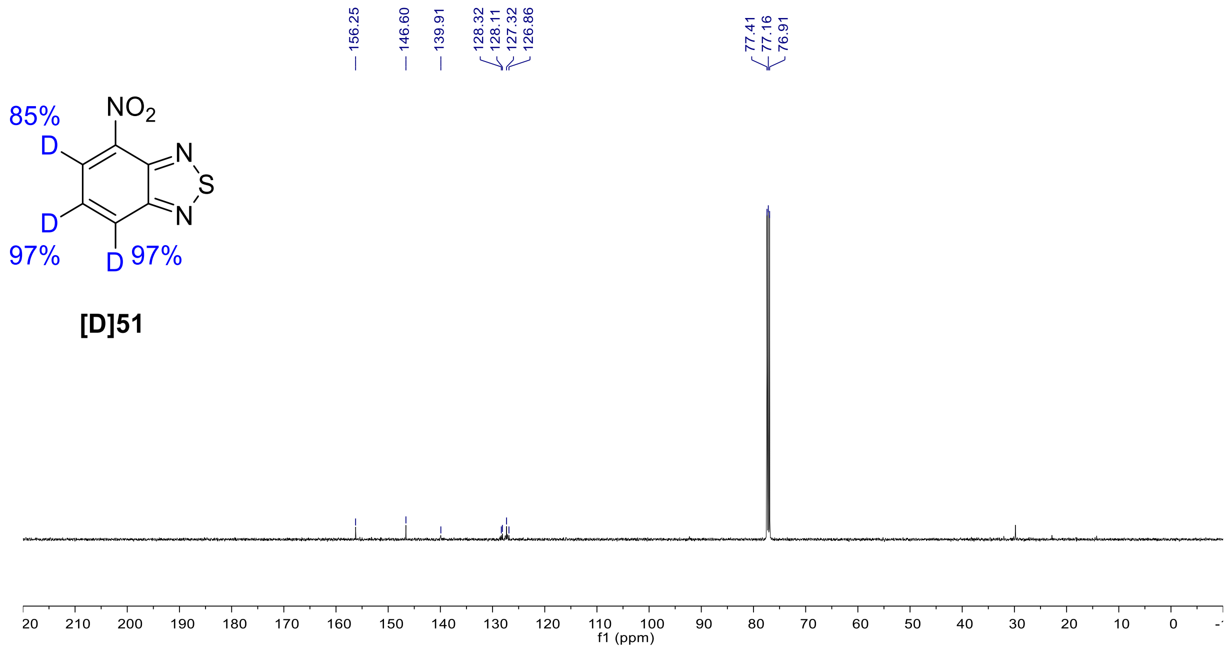

^{13}C NMR spectrum of compound **[D]51** (Procedure A, $CDCl_3$, 126 MHz)

methyl 4-nitrobenzoate (52)

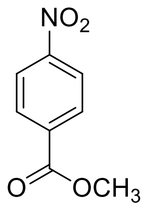

Following the general procedure A, the reaction was set up with methyl 4-nitrobenzoate (36.2 mg, 0.20 mmol). Purification by flash column chromatography (hexanes/EtOAc = 15:1) provided product **[D]52** as a white solid (36 mg, 97% yield).

Deuterium Incorporation

General procedure A: [GCMS (EI)] calcd for $C_8H_3D_4NO_4$ $[M]^+$ 3.51 D/molecule, [^{1}H NMR] 3.66 D/molecule.

NMR Data of the Starting Material

^{1}H NMR (500 MHz, $CDCl_3$) δ 8.31-8.28 (m, 2H), 8.23-8.20 (m, 2H), 3.98 (s, 3H).

NMR Data of the Product

General procedure A: ^{1}H NMR (500 MHz, $CDCl_3$) δ 8.29 (s, 0.10H, 95% D), 8.22 (s, 0.24H, 88% D), 3.98 (s, 2.93H, 2% D); ^{13}C NMR (126 MHz, $CDCl_3$) δ 165.3, 150.6, 135.53-135.46 (1C), 130.9-130.3 (2C), 123.7-123.1 (2C), 53.0.

Mass Data

	M+1	M+2	M+3	M+4	M+5
m/z	182	183	184	185	186
Abound	11178	57147	147302	333203	32011
Theoretical exact mass of start material: 181.0375					
Weighted average of deuterated product: 184.5470					
Average %D: 88%					

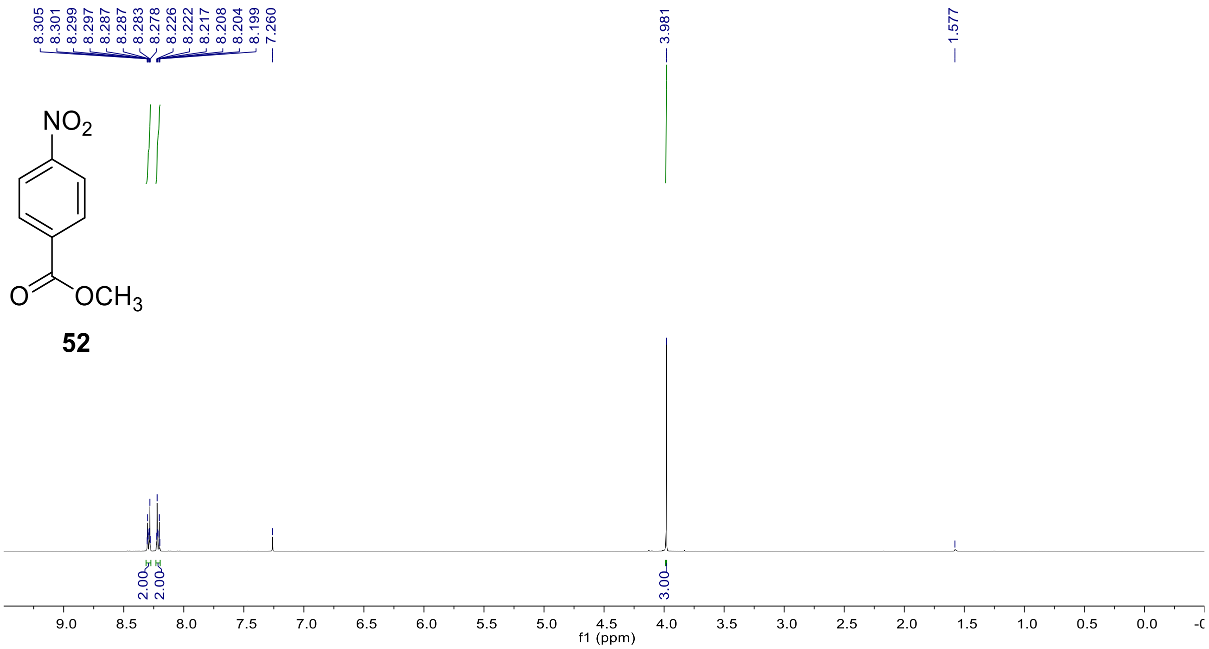

^{1}H NMR spectrum of compound **52** ($CDCl_3$, 500 MHz)

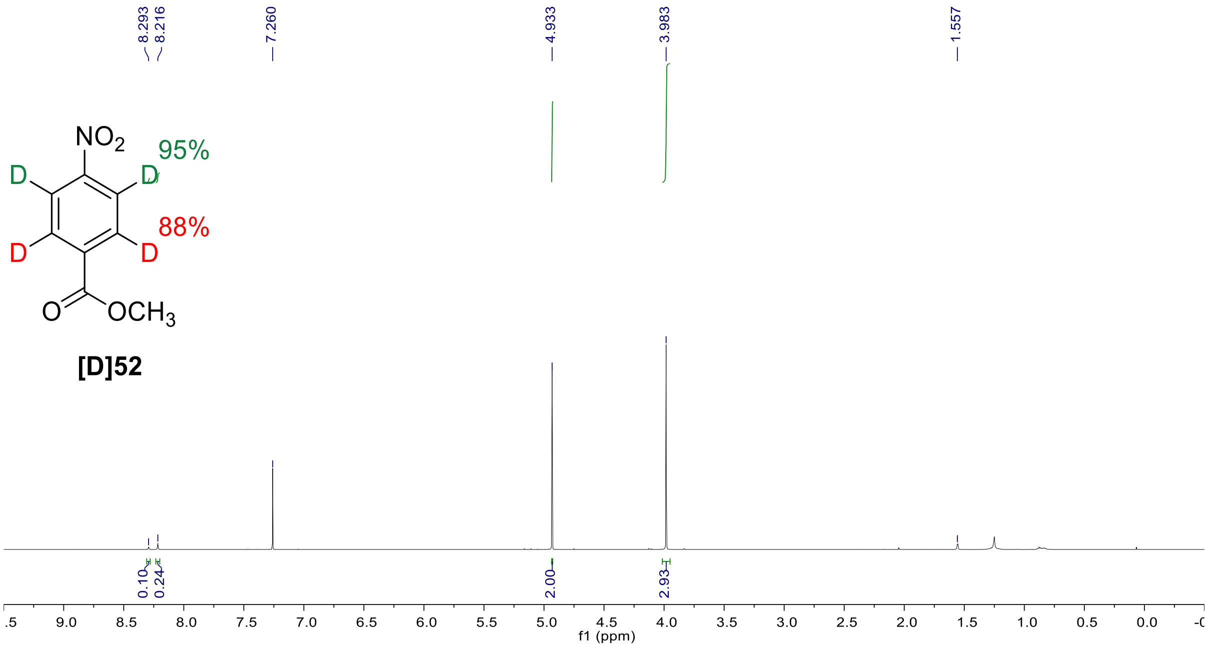

^{1}H NMR spectrum of compound **[D]52** (Procedure A, $CDCl_3$, 500 MHz)

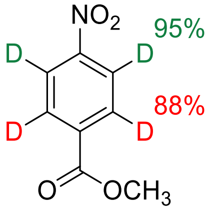

[D]52

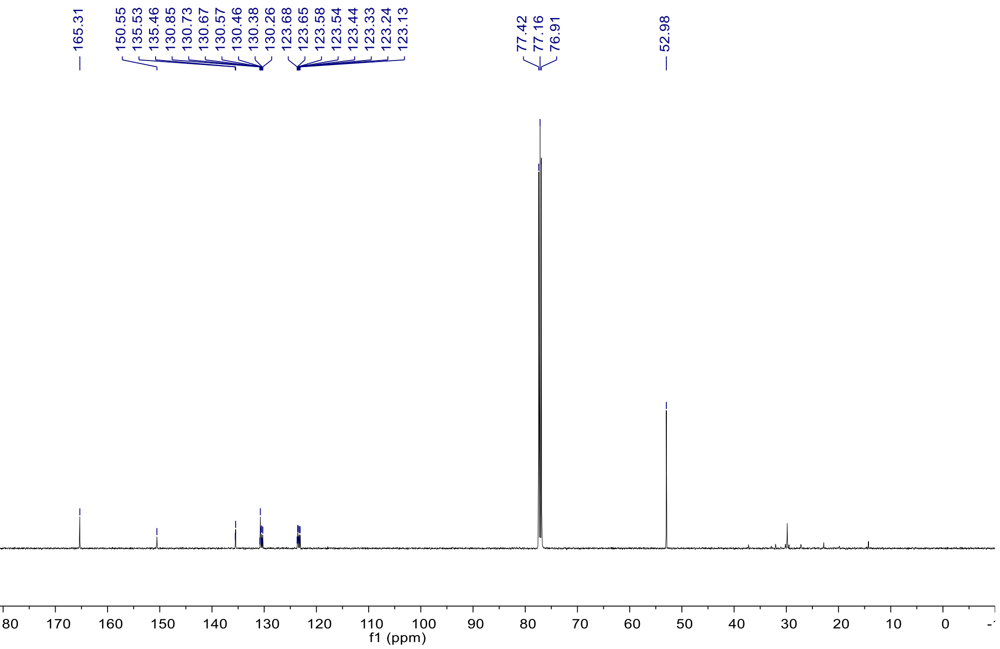

^{13}C NMR spectrum of compound **[D]52** (Procedure A, $CDCl_3$, 126 MHz)

methyl 3-nitrobenzoate (53)

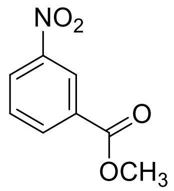

Following the general procedure A, the reaction was set up with methyl 3-nitrobenzoate (36.2 mg, 0.20 mmol). Purification by flash column chromatography (hexanes/EtOAc = 15:1) provided product **[D]53** as a yellow solid (36 mg, 97% yield).

Deuterium Incorporation

General procedure A: [GCMS (EI)] calcd for $C_8H_3D_4NO_4$ $[M]^+$ 3.16 D/molecule, [^{1}H NMR] 3.26 D/molecule.

NMR Data of the Starting Material

^{1}H NMR (500 MHz, $CDCl_3$) δ 8.88 (s, 1H), 8.42 (d, *J* = 8.2 Hz, 1H), 8.37 (d, *J* = 7.7 Hz, 1H), 7.66 (t, *J* = 8.0, 1H), 3.99 (s, 3H).

NMR Data of the Product

General procedure A: ^{1}H NMR (500 MHz, $CDCl_3$) δ 8.88 (s, 0.31H, 69% D), 8.42 (s, 0.13H, 87% D), 8.37 (s, 0.26H, 74% D), 7.66 (s, 0.04H, 96% D), 3.99 (s, 2.99H, 0% D); ^{13}C NMR (126 MHz, $CDCl_3$) δ 165.1, 148.34-148.28 (1C), 135.3-134.8 (1C), 132.0-131.8 (1C), 129.6-129.1 (1C), 127.5-127.0 (1C), 124.8-124.3 (1C), 53.0.

Mass Data

	M+1	M+2	M+3	M+4	M+5
m/z	182	183	184	185	186
Abound	20799	87322	196940	191931	18862
Theoretical exact mass of start material: 181.0375					
Weighted average of deuterated product: 184.1953					
Average %D: 79%					

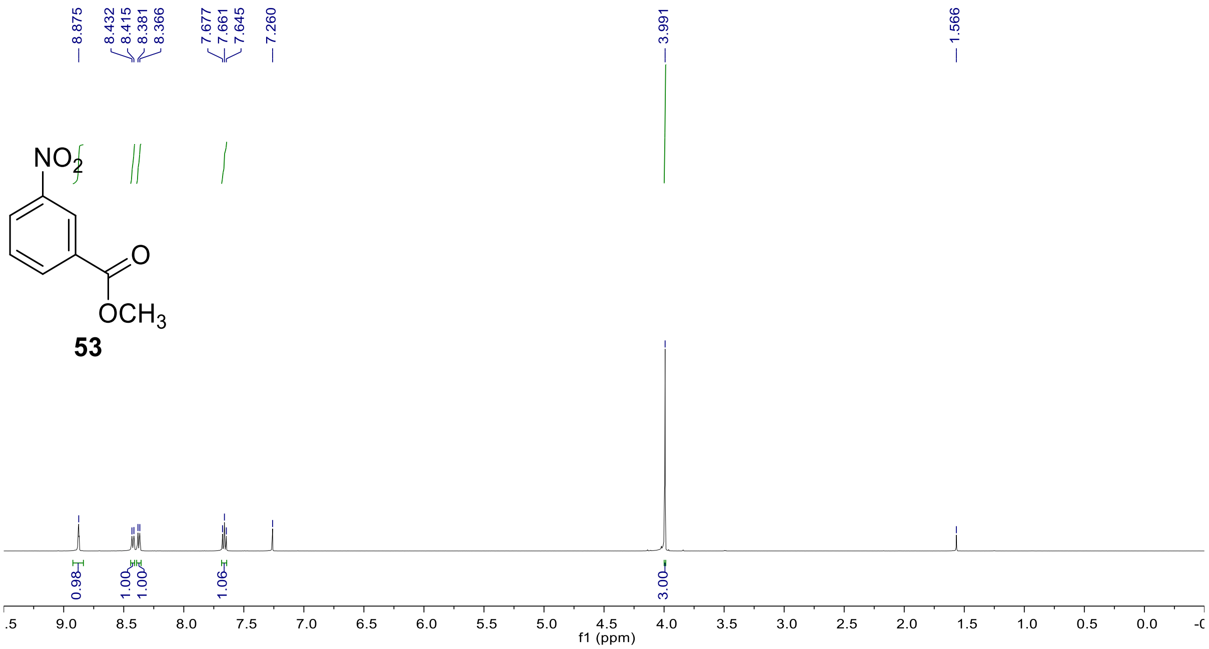

^{1}H NMR spectrum of compound **53** ($CDCl_3$, 500 MHz)

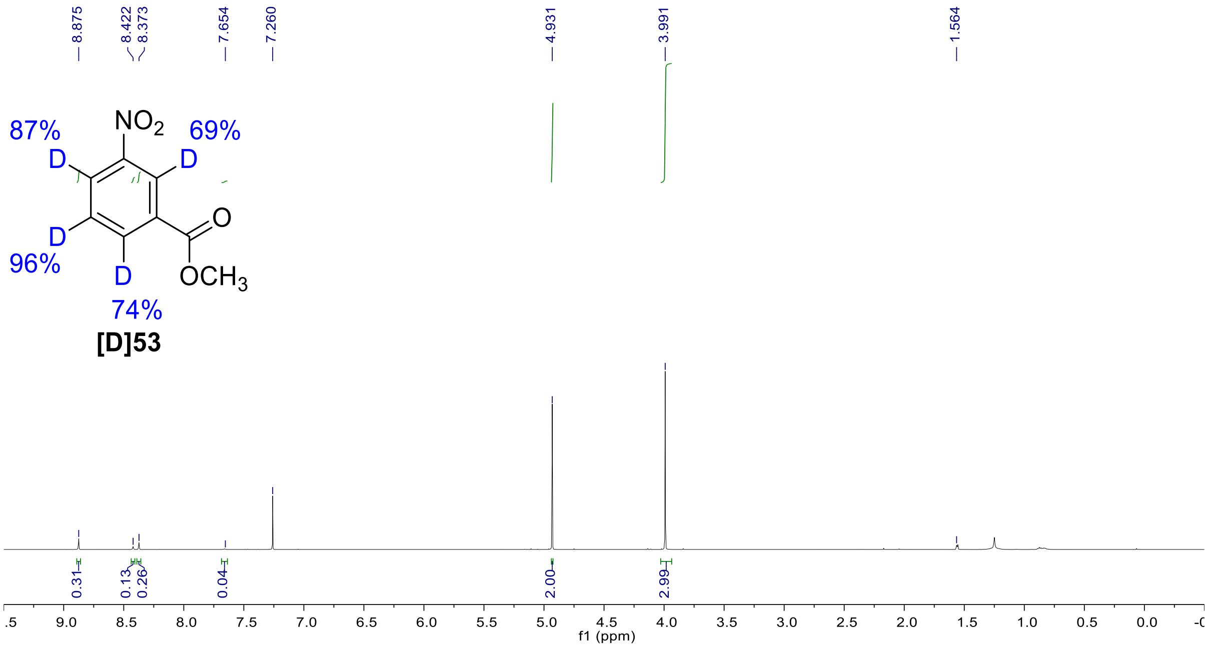

^{1}H NMR spectrum of compound **[D]53** (Procedure A, $CDCl_3$, 500 MHz)

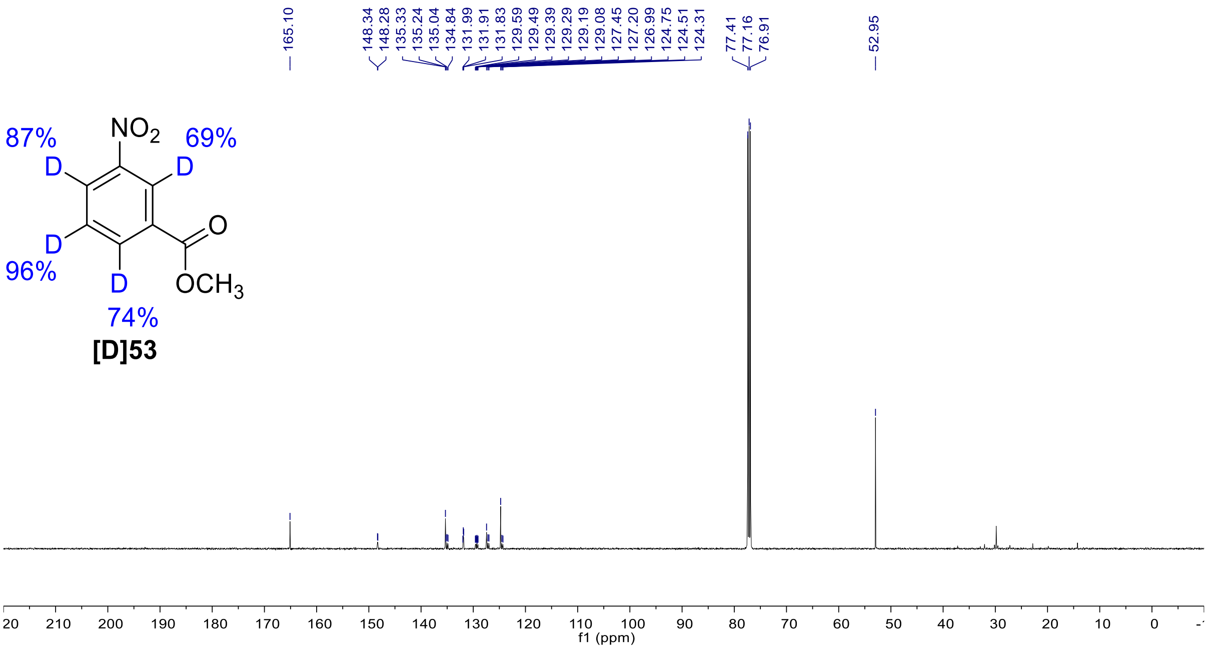

^{13}C NMR spectrum of compound **[D]53** (Procedure A, $CDCl_3$, 126 MHz)

diethyl phthalate (54)

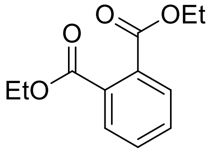

Following the general procedure A, the reaction was set up with diethyl phthalate (44.4 mg, 0.20 mmol). Purification by flash column chromatography (hexanes/EtOAc = 15:1) provided product **[D]54** as a transparent liquid (38 mg, 84% yield).

Deuterium Incorporation

General procedure A: [GCMS (EI)] calcd for $C_{12}H_{10}D_4O_4$ $[M]^+$ 3.73 D/molecule, [^{1}H NMR] 3.78 D/molecule.

NMR Data of the Starting Material

^{1}H NMR (500 MHz, $CDCl_3$) δ 7.73-7.70 (m, 2H), 7.54-7.50 (m, 2H), 4.36 (q, J = 7.1 Hz, 4H), 1.36 (t, J = 7.2 Hz, 6H).

NMR Data of the Product

General procedure A: ^{1}H NMR (500 MHz, $CDCl_3$) δ 7.73 (s, 0.14H, 93% D), 7.53 (s, 0.08H, 96% D), 4.37 (q, J = 7.1 Hz, 4.00H, 0% D), 1.37 (t, J = 7.2 Hz, 6.00H, 0% D); ^{13}C NMR (126 MHz, $CDCl_3$) δ 167.8 (2C), 132.3 (2C), 130.9-130.4 (2C), 128.9-128.4 (2C), 61.8 (2C), 14.2 (2C).

Mass Data

	M+4	M+5
m/z	225	226
Abound	28962	128080
Theoretical exact mass of start material: 222.0892		
Weighted average of deuterated product: 225.8156		
Average %D: 93%		

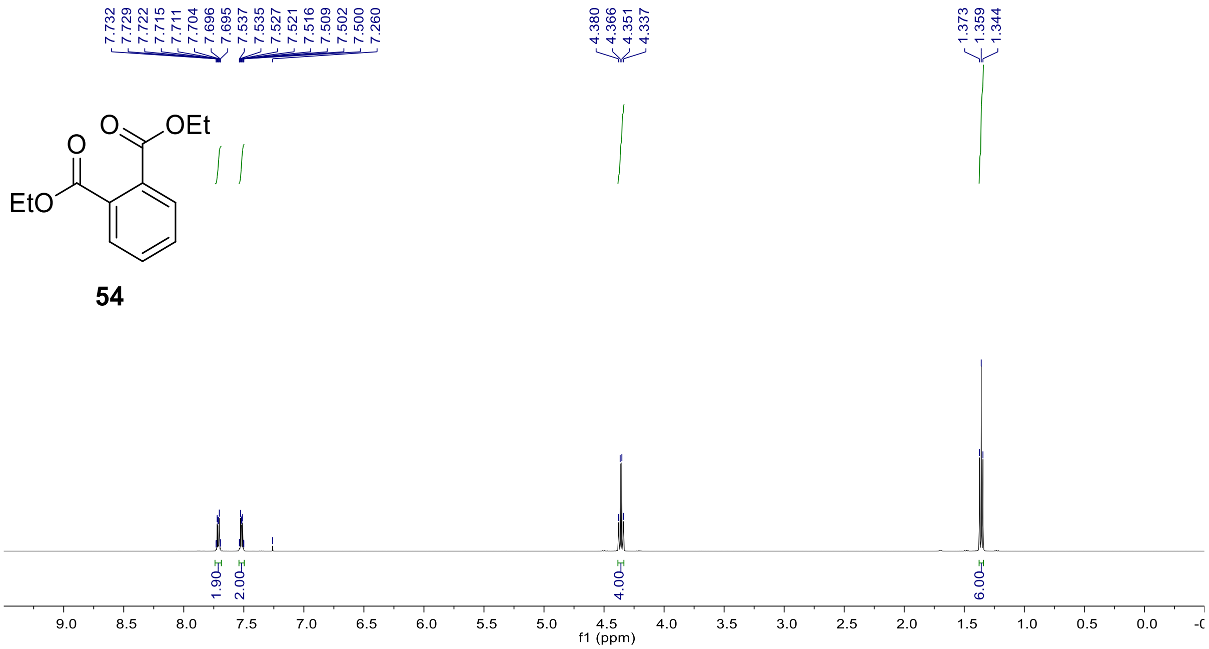

^{1}H NMR spectrum of compound **54** ($CDCl_3$, 500 MHz)

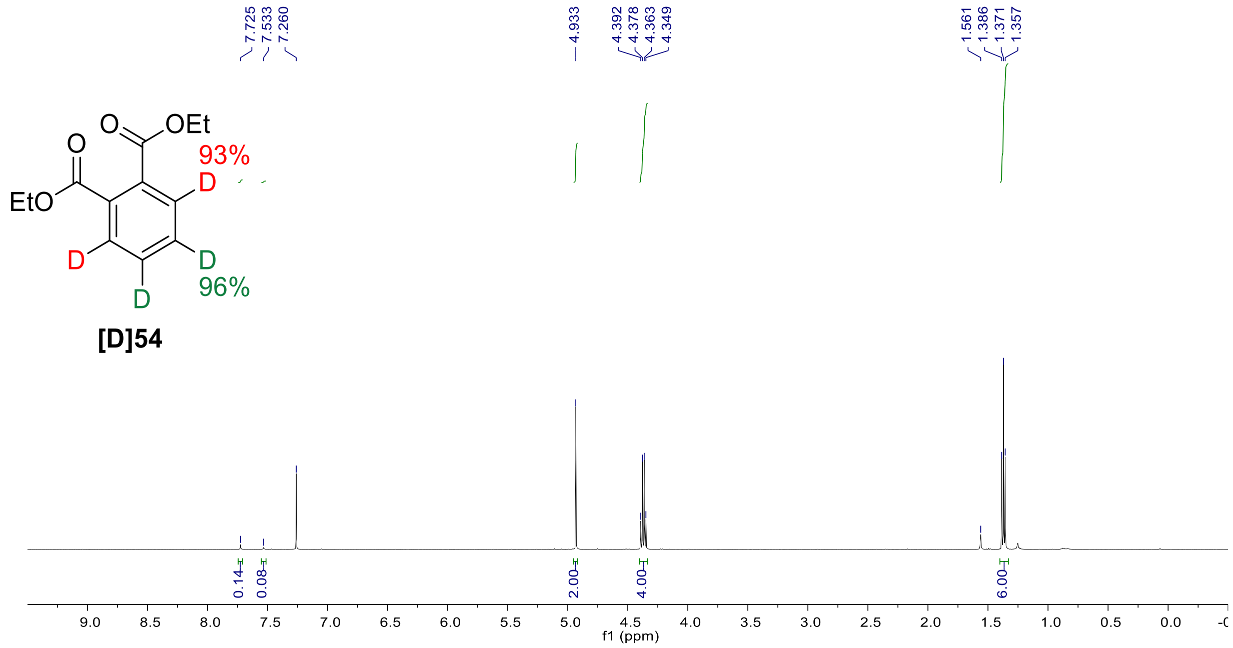

^{1}H NMR spectrum of compound **[D]54** (Procedure A, $CDCl_3$, 500 MHz)

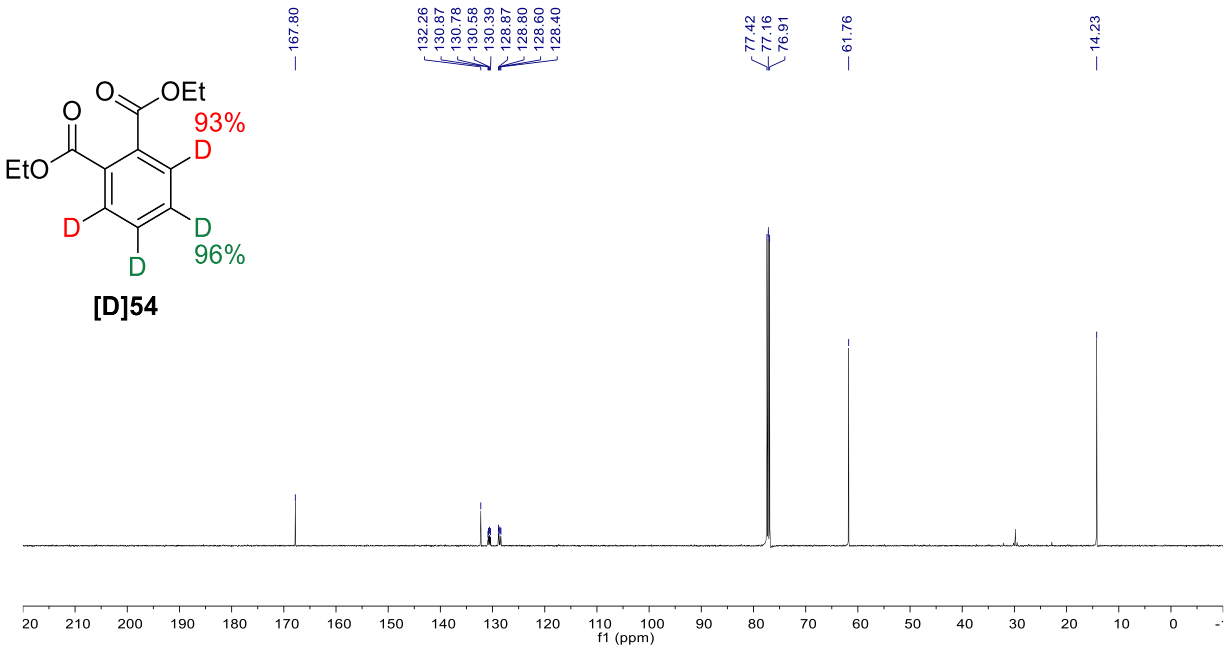

^{13}C NMR spectrum of compound **[D]54** (Procedure A, $CDCl_3$, 126 MHz)

isobenzofuran-1,3-dione (55)

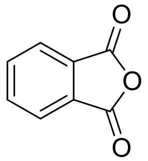

Following the general procedure A, the reaction was set up with isobenzofuran-1,3-dione (29.6 mg, 0.20 mmol). Purification by flash column chromatography (hexanes/EtOAc = 2:1) provided product **[D]55** as a white solid (22 mg, 72% yield).

Deuterium Incorporation

General procedure A: [GCMS (EI)] calcd for $C_8D_4O_3$ $[M]^+$ 3.77 D/molecule, [^{1}H NMR] 3.72 D/molecule.

NMR Data of the Starting Material

^{1}H NMR (500 MHz, $CDCl_3$) δ 8.06-8.01 (m, 2H), 7.94-7.90 (m, 2H).

NMR Data of the Product

General procedure A: ^{1}H NMR (500 MHz, $CDCl_3$) δ 8.04 (s, 0.08H, 96% D), 7.92 (s, 0.19H, 90% D); ^{13}C NMR (126 MHz, $CDCl_3$) δ 162.9 (2C), 136.1-135.5 (2C), 131.4 (2C), 125.8-125.3 (2C).

Mass Data

	M+2	M+3	M+4	M+5
m/z	150	151	152	153
Abound	9840	82694	253510	24409
Theoretical exact mass of start material: 148.0160				
Weighted average of deuterated product: 151.7895				
Average %D: 94%				

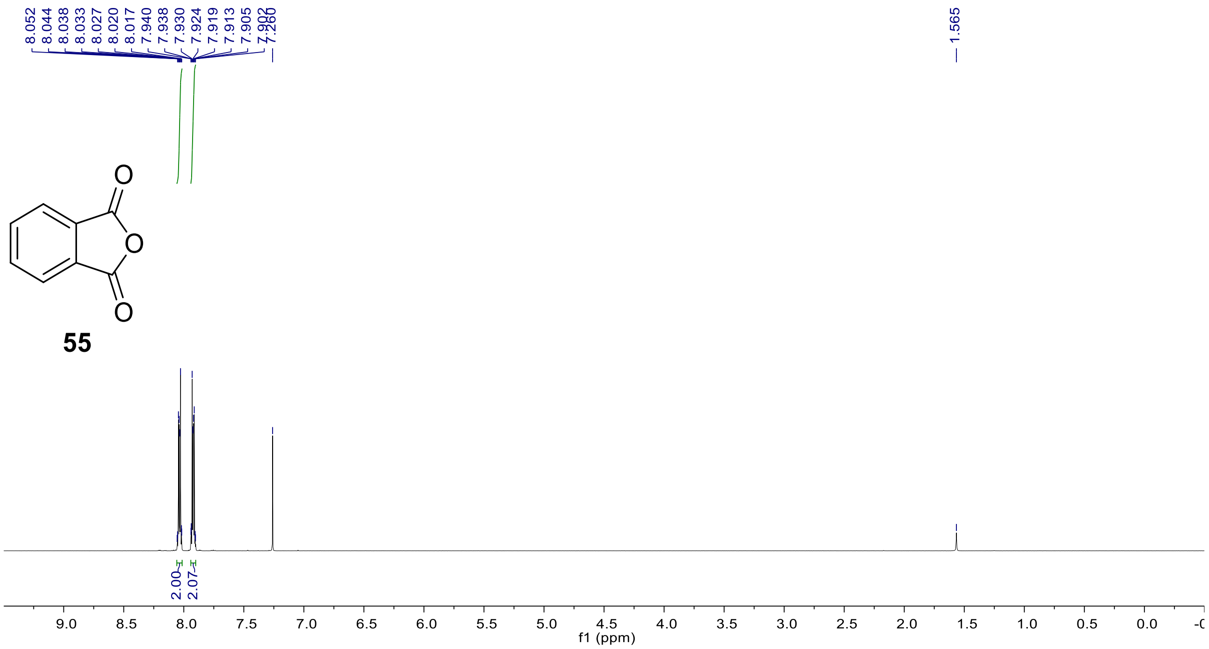

^{1}H NMR spectrum of compound **55** ($CDCl_3$, 500 MHz)

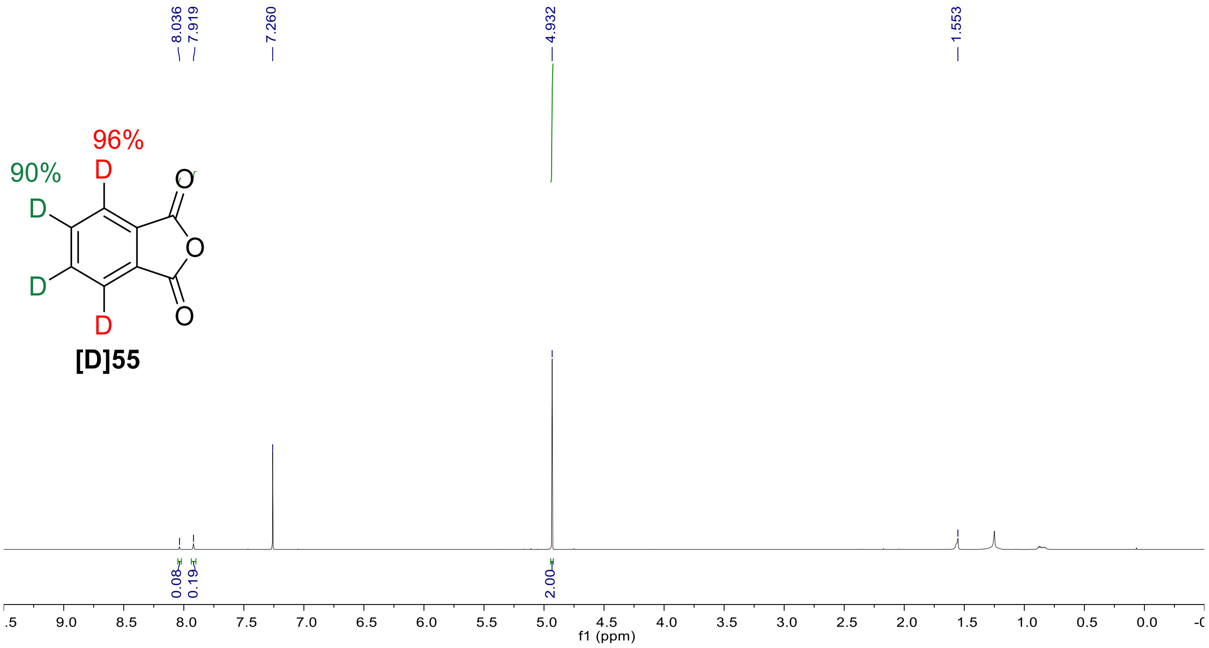

^{1}H NMR spectrum of compound **[D]55** (Procedure A, $CDCl_3$, 500 MHz)

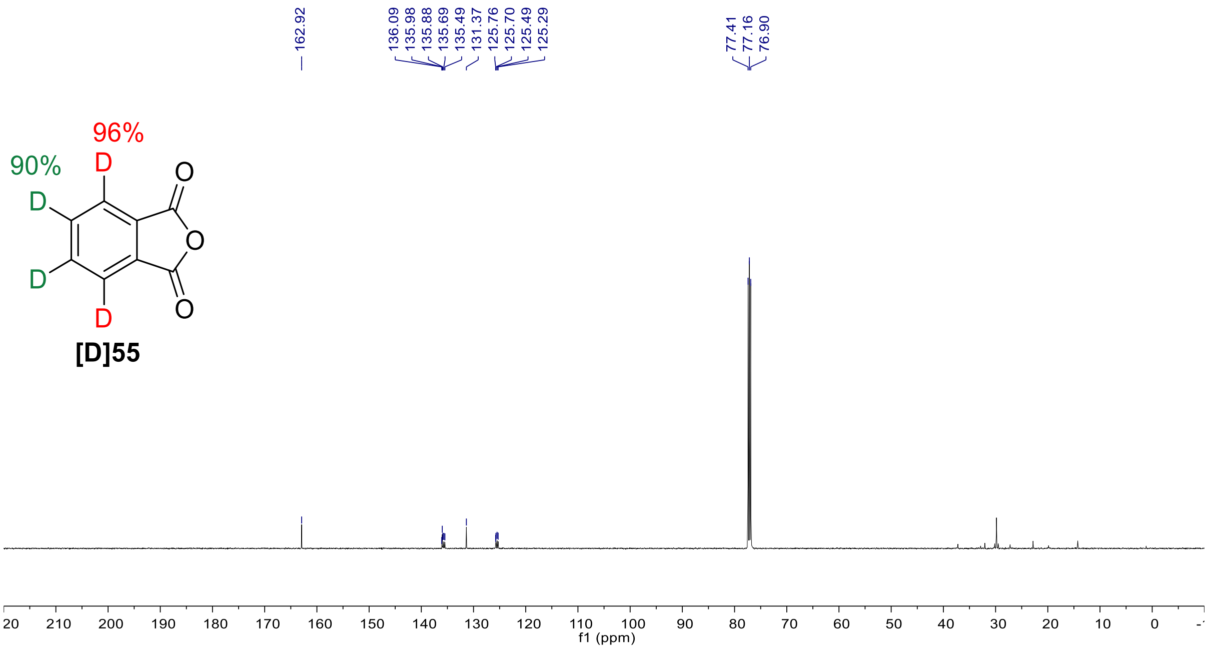

^{13}C NMR spectrum of compound **[D]55** (Procedure A, $CDCl_3$, 126 MHz)

VII. DFT Calculation

VII. A. General Information

All calculations were performed using the Gaussian 16 program package.[11] The geometry optimizations were conducted using the uB3LYP-D3[12] functional with 6-31G(d,p) basis set[13], where the LANL2DZ[14] basis set was used for Pd atom, which includes effective core potentials. Frequency analysis was conducted at the same level as the geometry optimizations, to derive the thermochemistry correction term (G – E) as well as to confirm the stationary points as either minima (no imaginary frequencies) or saddle points (one imaginary frequency) on the potential energy surface. Single point energy calculations based on gas-phase optimized structures were conducted using the uB3LYP-D3 functional with the 6-311+G(d,p) basis set (the SDD[15] basis set was used for Pd atom), together with the application of the SMD continuum solvation model to derive solution phase electronic energies (E_{Sol}). The solvent parameters for acetic acid solvent were taken from the Minnesota Solvent Descriptor Database.[16] Calculated Gibbs free energies in solution were used throughout the article for discussion. The final solution phase Gibbs free energies (G_{Sol}) were computed as follows:

$$G_{Sol} = E_{Sol} + (G - E) \quad (1)$$

$$\Delta G_{Sol} = \Sigma G_{Sol} \text{ for products} - \Sigma G_{Sol} \text{ for reactants} \quad (2)$$

VII. B. Computed Energy Profiles

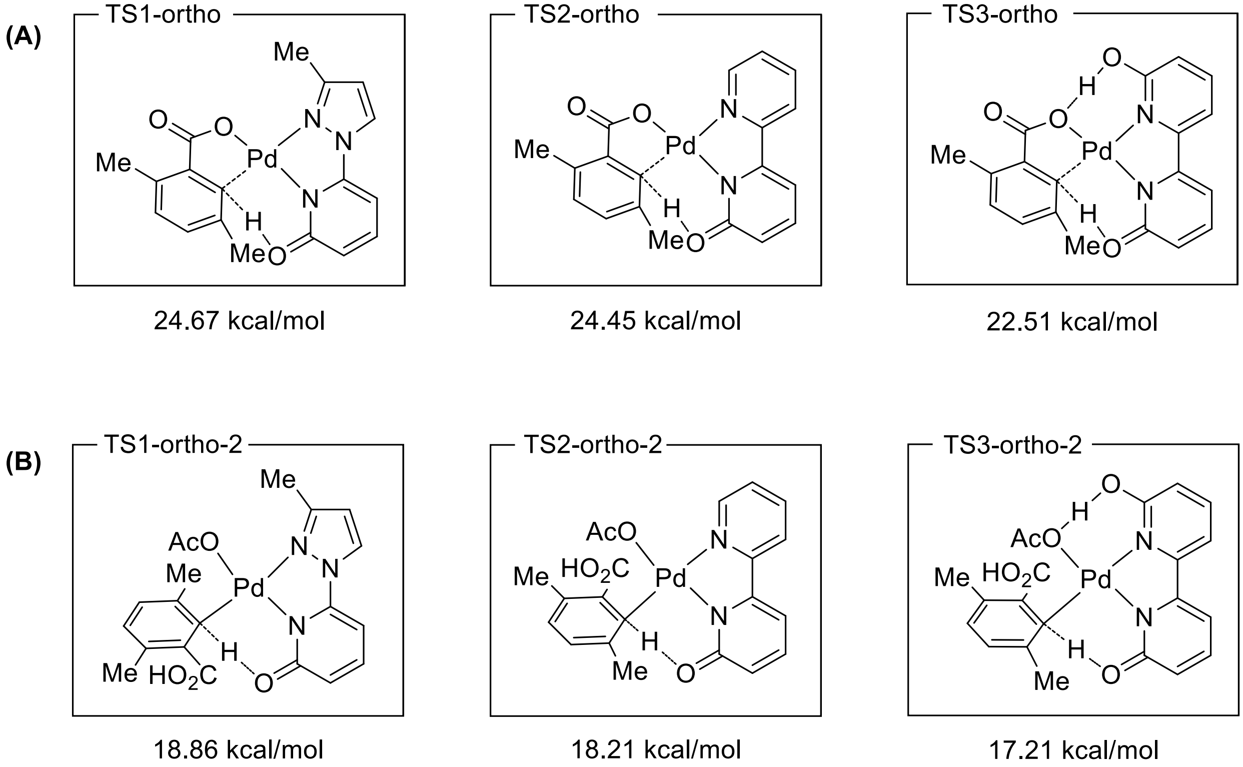

Fig. S2. Transition state energies for (A) directed and (B) nondirected C–H cleavage at the *ortho*-sp^2 position of 2,5-dimethylbenzoic acid. Free energies (kcal/mol) relative to 1/3 $Pd_3(OAc)_6$.

VII. C. Coordinates and Energies of Stationary Points

2,5-dimethylbenzoic acid

G_{sol} = -313434.705

C -0.68467700 -0.49468600 -0.01771000

C 0.70686000 -0.46186000 0.04004000

C 1.42506700 0.74719700 0.03371100

C 0.73921400 1.98247500 -0.03270500

C -0.66021700 1.93367100 -0.09042100

C -1.35946700 0.73108800 -0.08350800

H 1.25907800 -1.39311600 0.09155400

H -1.21226400 2.86813700 -0.14221500

H -2.44598700 0.74360500 -0.12994000

C 1.42816900 3.32491100 -0.04432700

H 2.04131200 3.46647900 0.84944500

H 2.11522100 3.41352100 -0.88985000

H 0.68969300 4.12945300 -0.10019500

C -1.44582300 -1.79871000 -0.00956000

H -2.12300900 -1.85730500 0.85021500

H -2.06017800 -1.90509200 -0.91087600

H -0.77030200 -2.65692600 0.03798000

C 2.91003800 0.70675200 0.09749200

O 3.65650300 1.66858200 0.10008300

O 3.40708800 -0.56016900 0.15642100

H 4.37150600 -0.45483100 0.19387600

3-Me-PzPyOH

G_{sol} = -368915.009

C 0.67051200 0.82218000 -0.02459800

C 1.17673600 -0.45045100 -0.09620000

N -0.66903200 1.02951900 0.07741000

C 0.24576300 -1.52591900 -0.05912200

H 2.23886800 -0.63182200 -0.17729500

C -1.65714000 0.01578800 0.12043300

C -1.10168400 -1.32244000 0.04338800

H 0.63151300 -2.54018500 -0.11492600

H -1.81019700 -2.14091100 0.07162300

O -2.83907300 0.32521100 0.21396300

N 1.45064600 1.99055500 -0.04840300

C 2.81162600 2.11707100 -0.14371700

C 3.08353800 3.46191000 -0.12537700

H 3.46135400 1.25953800 -0.21551300

C 1.81585500 4.10120700 -0.01512500

H 4.05488400 3.92991000 -0.18274900

N 0.83588000 3.20647700 0.03077100

C 1.51944500 5.56620900 0.04791500

H 1.88122400 6.07896600 -0.84970600

H 0.44323900 5.72964500 0.13147600

H 2.01038300 6.02931000 0.91048800

H -0.99252400 1.99075100 0.12742100

BpyOH

G_{sol} = -358105.699

C -4.10718400 -0.77272900 -0.02691900

C -5.00135400 -0.85201500 -1.06683800

N -4.51244200 -0.22033600 1.15484500

C -6.32018100 -0.35304500 -0.87003100

H -4.71617000 -1.28284800 -2.01670700

C -5.78792700 0.29719000 1.44202700

C -6.70765500 0.19623200 0.31870800

H -7.02929100 -0.41488000 -1.69133200

H -7.70804000 0.57937200 0.48105500

O -6.03353900 0.77081700 2.54692700

C -2.70004400 -1.24171800 -0.05862800

C -2.12108800 -1.83237300 -1.19194700

C -0.73632900 -1.45847700 1.12977900

C -0.79341400 -2.24222300 -1.12947000

H -2.69235200 -1.96940700 -2.10223100

C -0.07834300 -2.05471600 0.05388400

H -0.32342800 -2.70196600 -1.99349700

H 0.95849500 -2.36032600 0.14369100

N -2.00971000 -1.06102200 1.08254600

H -3.82067500 -0.18050600 1.89857900

H -0.21919000 -1.29195800 2.07217200

Bpy(OH)$_2$

G_{sol} = -405332.031

C -2.22333000 0.05580000 -0.02237300

C -3.14852400 -0.10034800 -1.02608200

N -2.60410900 0.67418700 1.13514300

C -4.46861100 0.38952300 -0.81805300

H -2.88483900 -0.58417800 -1.95631000

C -3.87885600 1.18892000 1.43121900

C -4.83026100 1.00522600 0.34651300

H -5.20103700 0.26630000 -1.61145300

H -5.83185800 1.38195300 0.51592200

O -4.09511300 1.72525300 2.51372800

C -0.81174400 -0.39353800 -0.07293900

C -0.25876800 -1.04233300 -1.17948800

C 1.18781300 -0.50251300 1.06967400

C 1.08450000 -1.42457100 -1.12286500

H -0.84973000 -1.24734900 -2.06271200

C 1.83641100 -1.15887200 0.01193700

H 1.53957200 -1.93036200 -1.96882700

H 2.87954400 -1.43471400 0.10439500

N -0.08618600 -0.13140500 1.03543200

H -1.89610500 0.77336700 1.85579100

O 1.89386200 -0.23066700 2.18704800

H 1.28863600 0.21836900 2.79826600

AcOH

G_{sol} = -143785.773

C -1.66117700 -0.41221200 -0.00015500

O -0.99959100 0.60118900 0.00007000

O -1.10433400 -1.65085000 -0.00005700

H -0.14185000 -1.51453900 0.00002900

C -3.16665500 -0.49059100 -0.00000600

H -3.50913000 -1.04054200 -0.88137000

H -3.50896100 -1.04079000 0.88127000

H -3.58270100 0.51577600 0.00017600

$Pd_3(OAc)_6$

G_{sol} = -1101358.023

Pd 1.60909300 0.92506300 -0.01356000

C -0.07286000 2.59704400 -1.87016200

O 1.01573700 2.45605300 -1.23913000

O -1.16881600 1.99359900 -1.66552200

C -2.26987000 -1.25420200 1.88606200

O -1.60629100 -2.12085300 1.24478100

O -2.29563100 -0.00136300 1.69280000

C -3.15950800 -1.78221600 2.99145500

H -3.43844900 -0.97835600 3.67308900

H -4.06727200 -2.19122200 2.53565200

H -2.65276700 -2.58945300 3.52302000

C -0.06505800 3.62604100 -2.98077300

H 0.83628200 3.50654400 -3.58540200

H -0.96125400 3.53585000 -3.59450700

H -0.03617200 4.62238800 -2.52815300

Pd -0.00037800 -1.85820000 0.00034600

C -2.23562600 -1.34811500 -1.85612400

O -2.65396200 -0.34407600 -1.20701500

O -1.16686400 -2.00145200 -1.66447400

C 0.07421800 2.59735200 1.87000400

O 1.16995600 1.99349800 1.66539600

O -1.01447300 2.45662800 1.23908100

C 0.06692300 3.62667300 2.98031900

H -0.83472600 3.50816100 3.58467300

H 0.96283800 3.53586700 3.59437500

H 0.03912000 4.62291800 2.52740000

C -3.12089900 -1.81064900 -2.99380600

H -3.16842300 -1.01870700 -3.74674200

H -2.73529400 -2.72844200 -3.43688400

H -4.13456400 -1.96763200 -2.61600400

Pd -1.60860100 0.92579600 0.01368400

C 2.23510200 -1.34867700 1.85671700

O 2.65395700 -0.34504000 1.20731900

O 1.16599900 -2.00149900 1.66523100

C 3.12019300 -1.81143500 2.99444900

H 3.16824500 -1.01932100 3.74716900

H 2.73408400 -2.72888600 3.43779900

H 4.13373600 -1.96911900 2.61660900

C 2.26927700 -1.25553900 -1.88560700

O 2.29564000 -0.00268700 -1.69254100

O 1.60539400 -2.12177500 -1.24407600

C 3.15832500 -1.78416500 -2.99118400

H 4.06542600 -2.19475600 -2.53549300

H 2.65049900 -2.59041200 -3.52322900

H 3.43846200 -0.98035000 -3.67237500

Int1-1

G_{sol} = -761883.084

C -3.26212600 -2.95710700 0.44182100

H -3.38261000 -4.02345300 0.29200400

C -4.15538700 -2.21815300 1.16932200

H -5.01912600 -2.70040800 1.61861600

C -2.09705100 -2.35001600 -0.17171300

C -3.97917300 -0.82310500 1.35365100

H	-4.67888600	-0.23146800	1.92862100
C	-2.86926200	-0.26742400	0.76158200
N	-1.96861900	-0.96807500	0.03613200
Pd	-0.39940900	0.08976000	-0.71273100
O	-1.24916100	-2.95923600	-0.83841800
N	-2.55551300	1.11012100	0.85455000
C	-3.18305300	2.14631300	1.46605300
C	-2.43290200	3.27924900	1.22834100
H	-4.10102100	2.00069500	2.01266500
C	-1.32557900	2.87700500	0.44606100
H	-2.64316700	4.28175200	1.56783400
N	-1.42295100	1.55761700	0.23268200
C	-0.20902800	3.71366100	-0.08413800
H	0.75143800	3.42986500	0.35387600
H	-0.09062000	3.57189200	-1.15952400
H	-0.40556700	4.76515900	0.13548500
C	4.36272000	-0.91978500	1.25184100
C	3.90803700	-2.18606600	0.86653900
C	2.91634700	-2.32493400	-0.10349900
C	2.33046000	-1.20985900	-0.71196300
C	2.76930400	0.07287200	-0.31809800
C	3.78449700	0.19426100	0.63688800
H	4.34009000	-3.07451700	1.32060400
H	2.59256100	-3.31911200	-0.40036800
H	4.11803000	1.19682400	0.88717100
C	2.21484900	1.36462900	-0.88081300
O	2.87393600	2.39738800	-0.83887500
O	1.00442600	1.35029100	-1.41443000
C	1.28015300	-1.42947600	-1.77747100
H	0.21326700	-1.57204200	-1.38693800
H	1.33300000	-0.71438200	-2.59692100
H	1.34427400	-2.44120600	-2.19110700
C	5.42452600	-0.75683200	2.31312100
H	6.03036700	0.13696200	2.13654600
H	4.97383200	-0.65378200	3.30820300
H	6.09420900	-1.62164900	2.34670300

TS1-1

G_{sol} = -761877.307

Imaginary frequency = -1161.42

C	-2.86053800	0.51021300	0.41256400
H	-2.88491700	-0.56792400	0.31655500
C	-3.87287400	1.21958800	1.01915100
H	-4.73376500	0.69183300	1.41900100
C	-1.72268700	1.19879600	-0.11132100
C	-3.81731000	2.62401600	1.14206900
H	-4.60414200	3.18266500	1.63097300
C	-2.70154700	3.24399100	0.61509400
N	-1.70673000	2.56836800	0.00587300
Pd	-0.11539400	3.63188200	-0.69297900
O	-0.73517000	0.63385400	-0.67305900
N	-2.47762200	4.63490200	0.66531900
C	-3.23654800	5.65129900	1.16134500
C	-2.53139900	6.81645200	0.97078500
H	-4.20425400	5.46709800	1.60049800
C	-1.31392500	6.45023200	0.33866100

H	-2.83815200	7.81423700	1.24504500
N	-1.30156600	5.12746600	0.16057500
C	-0.17396900	7.32696200	-0.06337900
H	0.41135300	7.60563200	0.82030000
H	0.49246300	6.81363100	-0.75875400
H	-0.55199400	8.24934600	-0.51352600
C	4.75169500	2.41685400	1.02974600
C	4.12931700	1.19131600	0.76458100
C	3.01961700	1.11899400	-0.07587800
C	2.47969500	2.26621700	-0.67081600
C	3.08322700	3.50995700	-0.38572300
C	4.21431800	3.56197000	0.43383300
H	4.52302700	0.28067100	1.21013100
H	2.56480300	0.15334000	-0.28337400
H	4.66826400	4.53487900	0.59717400
C	2.57435000	4.82733200	-0.93064900
O	3.30606700	5.80903200	-0.97857100
O	1.32074900	4.89460700	-1.34611000
C	1.30018100	2.13271400	-1.59340700
H	0.18932000	1.63410500	-1.06743800
H	1.33391500	2.80080600	-2.45523100
H	1.27215400	1.12279700	-2.02450900
C	5.94575700	2.50855100	1.94991600
H	6.62363600	3.31112800	1.64349200
H	5.63561300	2.72153800	2.98083400
H	6.51241100	1.57234100	1.96701100

Int1-2

G_{sol} = -761894.103

C	-1.71661200	0.86536400	-0.01089700
H	-2.08888900	-0.06706500	-0.41518800
C	-2.04406200	1.32207400	1.25519300
H	-2.71232200	0.74385900	1.88510900
C	-0.83623100	1.63750100	-0.78225600
C	-1.48471700	2.50572700	1.74504700
H	-1.67845500	2.83911600	2.75576200
C	-0.64164600	3.22095100	0.90169900
N	-0.36139900	2.82591600	-0.36391700
Pd	0.84916200	4.16403400	-1.53674600
O	-0.44742400	1.17176500	-1.96705600
C	3.97417100	2.23876400	-5.45104600
C	2.78312100	1.50703900	-5.47029300
C	1.63669100	1.98802600	-4.83394800
C	1.64563000	3.20878400	-4.14563900
C	2.85844000	3.93533000	-4.09045600
C	3.98360000	3.45837500	-4.75908900
H	2.74411700	0.55669500	-5.99776000
H	0.71143300	1.41779600	-4.89355400
H	4.88419100	4.06428600	-4.71637100
C	3.00351900	5.23063600	-3.31085500
O	3.94652700	5.98114700	-3.54109200
O	2.10843200	5.51281300	-2.38611300
C	0.41267900	3.73539100	-3.49709300
H	0.15557100	4.74002900	-3.84451500
H	-0.45725200	3.08547900	-3.63351600
H	0.22130200	1.77589800	-2.34445100

N 0.00247300 4.39523600 1.32100300

C -0.18792400 5.15988800 2.44257800

C 0.72020100 6.18715000 2.38526400

H -0.94935100 4.92509000 3.16932700

C 1.45355700 5.99802900 1.17810800

H 0.84660200 6.98229800 3.10455600

N 1.01112600 4.91146200 0.55475600

C 2.56644800 6.81313500 0.60383500

H 3.51632600 6.55392000 1.08554100

H 2.65938000 6.62961400 -0.47056600

H 2.38654100 7.87762400 0.77927000

C 5.22478200 1.73327400 -6.13013900

H 5.68984500 2.51689400 -6.73805500

H 5.97322800 1.41203200 -5.39525500

H 5.01314600 0.88014400 -6.78170500

Int1-3

G_{sol} = -905673.936

C 0.72701600 -0.38196000 0.34750000

H 0.68626900 -1.40485200 0.70259100

C -0.39397600 0.37824800 0.17272300

H -1.37684900 -0.03329700 0.38533900

C 2.05436600 0.14119900 0.08735900

C -0.28751000 1.71613500 -0.27343900

H -1.16083400 2.34416000 -0.38360900

C 0.98000000 2.18271800 -0.54133700

N 2.11880400 1.45587400 -0.41709100

O 3.08348800 -0.52047100 0.28396100

N 1.18428000 3.51945000 -0.96235000

C 0.29840900 4.52406900 -1.18816800

C 1.02746300 5.65272400 -1.49141800

H -0.76474500 4.36609300 -1.11066800

C 2.38693100 5.27290100 -1.43862100

H 0.64333400 6.63427700 -1.72302300

N 2.46076400 3.97605800 -1.11317300

C 3.58307100 6.12210700 -1.70704000

H 3.88443200 6.01615200 -2.75398500

H 3.34477900 7.17133900 -1.51773800

H 4.42379600 5.81444000 -1.08461800

C 5.94413600 0.73212700 -2.61725900

C 5.15884700 0.51431400 -1.44919000

C 5.63003700 0.84866000 -0.16222700

C 6.89420300 1.45033600 0.00405900

C 7.66241900 1.64288400 -1.13166000

C 7.21490600 1.29104500 -2.43001100

C 5.50076400 3.92225500 -2.82079300

O 4.69797500 3.58219300 -3.68629600

O 5.41517200 3.61856300 -1.54594300

C 6.71738100 4.78358700 -3.12994800

H 6.68483900 5.70146000 -2.53479600

H 7.63297500 4.25182500 -2.85883800

H 6.73045000 5.02935400 -4.19249800

Pd 3.85229700 2.44993300 -1.02425300

C 8.10521200 1.54793200 -3.59439800

O 7.90376400 1.20990200 -4.74397500

O 9.22785300 2.24605900 -3.25607800

H	9.72244100	2.35520300	-4.08449100
C	7.36757400	1.87632400	1.36956200
H	6.90113600	2.82752500	1.65327000
H	7.09725500	1.14017200	2.13339600
H	8.45176400	2.01712100	1.39554000
H	4.27818600	-0.11603200	-1.51110300
C	5.35917200	0.36551300	-3.95265000
H	5.27015400	1.26163000	-4.57268500
H	5.99687700	-0.33698400	-4.49343400
H	4.36444000	-0.06662300	-3.81942400
H	8.64882100	2.08230700	-1.03425200
H	5.03149500	0.53497200	0.68703400

TS1-2

G_{sol} = -905664.514

Imaginary frequency = -1031.31

C	1.01158000	-0.17294500	0.22878100
H	0.98793400	-1.22764200	0.47176100
C	-0.08144000	0.64822600	0.38629300
H	-1.01304800	0.24162800	0.76851100
C	2.23745500	0.36072800	-0.27065300
C	-0.01189900	2.01609100	0.06108500
H	-0.86248300	2.67172700	0.19002700
C	1.19605900	2.48069200	-0.42340500
N	2.28456800	1.69533600	-0.58894600
O	3.27901100	-0.36283600	-0.42670500
N	1.40003800	3.82714900	-0.79087700
C	0.53360900	4.87728100	-0.86251300
C	1.24578600	5.95996200	-1.32594000
H	-0.50380500	4.77369900	-0.58755800
C	2.57236700	5.50280700	-1.53247200
H	0.87148500	6.95649800	-1.50362100
N	2.64661200	4.21584800	-1.19711200
C	3.75491600	6.23518300	-2.07197500
H	3.96025000	5.89472800	-3.09208200
H	3.57025100	7.31132400	-2.07678100
H	4.64383900	6.01110800	-1.47826100
C	6.81460000	0.37402400	-3.34422400
C	5.54757700	0.65331000	-2.82542600
C	5.31532900	0.92922800	-1.46376300
C	6.40304800	0.88201100	-0.55810700
C	7.66442100	0.56217200	-1.04965000
C	7.88725500	0.33218800	-2.41931100
C	5.84424300	3.86583900	-3.04825800
O	4.99658900	3.66834200	-3.91860500
O	5.67985200	3.66096500	-1.76568700
C	7.24226900	4.36858600	-3.38486400
H	7.56943600	5.12831700	-2.67023400
H	7.93732700	3.52535000	-3.30947500
H	7.26368500	4.76251200	-4.40187600
Pd	4.00226300	2.61855500	-1.26698300
H	4.24646100	0.35476300	-0.96050100
C	9.27477100	0.04457300	-2.88365500
O	9.62472300	-0.11827900	-4.03775700
O	10.17409400	-0.02209600	-1.86485000
H	11.02870200	-0.20436500	-2.28774900

C	6.19304500	1.18531100	0.90310200
H	6.19302800	2.26904000	1.06796200
H	5.22421400	0.80116000	1.23851900
H	6.98016300	0.74957300	1.52562800
H	4.70843900	0.68811800	-3.51381400
C	6.97017800	0.15599100	-4.82907100
H	7.63964500	0.90020200	-5.26831200
H	7.41577000	-0.81725700	-5.05088400
H	5.99691600	0.22766800	-5.32102800
H	8.50674700	0.50852100	-0.36924300

Int1-4

G_{sol} = -905676.624

C	0.79638200	-0.04954400	0.50273700
H	0.70490000	-1.08788700	0.79409600
C	-0.23268400	0.86251400	0.63196700
H	-1.18743400	0.55468600	1.04595500
C	2.02005600	0.38908100	-0.03454600
C	-0.04915000	2.18679800	0.22364600
H	-0.84619600	2.91189000	0.31415200
C	1.18788000	2.53872400	-0.30106500
N	2.22353300	1.66835400	-0.41911400
O	2.98802600	-0.50351800	-0.15559500
N	1.43376000	3.84543100	-0.75744500
C	0.58679500	4.91671800	-0.86513800
C	1.30627000	5.94171400	-1.42801400
H	-0.44131000	4.86763100	-0.54546200
C	2.61240600	5.42765800	-1.65329700
H	0.95114300	6.93461400	-1.65854200
N	2.66890100	4.16668200	-1.23853800
C	3.79558200	6.08549400	-2.28216500
H	3.95288400	5.67548700	-3.28526800
H	3.64589200	7.16490900	-2.35173800
H	4.69823900	5.86844500	-1.70663400
C	6.48603600	-0.73743600	-2.52171100
C	5.58146900	0.32586300	-2.39530500
C	5.29518300	0.93568400	-1.16630400
C	5.95226800	0.49267400	0.00122600
C	6.85022300	-0.56955400	-0.11437500
C	7.12620500	-1.19119700	-1.34386500
C	5.84595600	3.62420400	-3.08699400
O	5.00853200	3.25551500	-3.91276900
O	5.73068000	3.52886900	-1.78843300
C	7.16546700	4.25745200	-3.51342100
H	7.37310600	5.15401000	-2.92266200
H	7.97712700	3.54754700	-3.32364500
H	7.13803300	4.49964800	-4.57671800
Pd	4.05696400	2.51684100	-1.17892300
C	8.09035600	-2.31857700	-1.38613500
O	8.41605500	-2.95446900	-2.37321900
O	8.62142800	-2.61601100	-0.16551200
H	9.22683800	-3.35694400	-0.32805300
C	5.68702400	1.13228700	1.34434700
H	5.91909900	2.20196500	1.31820600
H	4.62999500	1.03915400	1.62145500
H	6.28391800	0.66728300	2.13432000

H 5.10928200 0.71568700 -3.29275200
C 6.73226100 -1.32619200 -3.88930300
H 7.78941000 -1.26927200 -4.16224700
H 6.47715000 -2.38917500 -3.91811400
H 6.14099700 -0.79524900 -4.64013700
H 7.35922800 -0.92810800 0.77362800
H 3.79852200 -0.10628400 -0.55581900

Int2-1

G_{sol} = -751076.171

C -3.07416200 -2.47592900 1.97759200
H -3.70370400 -3.31024900 1.68974800
C -2.89646300 -2.11441300 3.28524800
H -3.39403200 -2.66897700 4.07628200
C -2.42303400 -1.76007400 0.89297300
C -2.06484700 -1.01868400 3.62408900
H -1.91968100 -0.73240100 4.65786000
C -1.44798900 -0.33308000 2.59624800
N -1.62413400 -0.68739600 1.28827100
Pd -0.61298000 0.43364500 -0.06131500
O -2.54496800 -2.05262700 -0.30614800
C -0.55859800 0.82079200 2.79188700
C -0.21589700 1.38884200 4.02345800
N -0.04530600 1.35536200 1.64953100
C 0.63932300 2.48518700 4.06129300
H -0.61853100 0.97246800 4.93849300
C 0.77789800 2.41629700 1.66863000
C 1.14538800 3.01047900 2.87002700
H 0.90849900 2.92819500 5.01508300
H 1.11677900 2.75800000 0.69424700
H 1.81270200 3.86440900 2.86232000
C 3.13343300 -1.70985500 -3.24055900
C 2.02691300 -2.54080900 -3.45130600
C 0.73717700 -2.09578800 -3.16840000
C 0.50220900 -0.81655700 -2.65135400
C 1.61162500 0.02408000 -2.41919900
C 2.89837000 -0.42928700 -2.73403300
H 2.17252300 -3.54174900 -3.85036400
H -0.10897100 -2.75148900 -3.35721400
H 3.72258300 0.25887600 -2.57361000
C 1.52103100 1.42716700 -1.85375800
O 2.42564300 2.23167700 -2.04097300
O 0.45164500 1.76310600 -1.15001700
C -0.92487000 -0.39410700 -2.39107700
H -1.11666900 0.65514900 -2.61374600
H -1.63090800 -0.99795600 -2.97050500
H -1.33767300 -0.73086200 -1.37869100
C 4.53727800 -2.18706200 -3.52697100
H 4.97426300 -2.67581400 -2.64708800
H 4.55464200 -2.91404600 -4.34493300
H 5.19394800 -1.35464100 -3.79667400

TS2-1

G_{sol} = -751069.488

Imaginary frequency = -1229.13

C -3.99087000 -0.30938600 0.19131600

H -4.56584600 -1.16015800 -0.15361100
C -3.90757600 0.03257100 1.52203200
H -4.43318500 -0.56123000 2.26432600
C -3.29617500 0.46714300 -0.79100700
C -3.14251100 1.14213100 1.94047700
H -3.06892300 1.40017800 2.98927200
C -2.48598200 1.88021300 0.97071700
N -2.58403800 1.54535900 -0.34473000
Pd -1.54565000 2.67051900 -1.66830400
O -3.30253500 0.21427800 -2.03735200
C -1.62681600 3.05424900 1.22395200
C -1.37496300 3.61391400 2.47881900
N -1.05277600 3.59465300 0.11568200
C -0.53361800 4.72043200 2.57587300
H -1.83155100 3.18989900 3.36509100
C -0.24450900 4.65976100 0.19590000
C 0.04215000 5.25398400 1.42221500
H -0.33093700 5.16228700 3.54650300
H 0.15209700 5.00774100 -0.75505700
H 0.70077900 6.11401000 1.46247000
C 2.01095700 0.39806200 -4.90839300
C 0.88600400 -0.43455500 -4.93797300
C -0.36347800 0.03874600 -4.54283000
C -0.53874900 1.35264000 -4.08817000
C 0.59647400 2.19057200 -4.02610000
C 1.83702900 1.70948200 -4.45739900
H 0.98437700 -1.46046000 -5.28557200
H -1.22746300 -0.61952600 -4.59518200
H 2.67582500 2.39839800 -4.42743000
C 0.57103500 3.61621900 -3.51042100
O 1.46266900 4.40119400 -3.81103100
O -0.41918900 3.98349900 -2.71511400
C -1.91723500 1.81669100 -3.70641400
H -2.13350300 2.84342900 -4.00914200
H -2.67280800 1.21072800 -4.22385700
H -2.51608600 1.17634800 -2.68901600
C 3.37298900 -0.10782300 -5.32085400
H 3.92118700 -0.51564200 -4.46198000
H 3.29601900 -0.90591200 -6.06605800
H 3.98450700 0.69488600 -5.74409100

Int2-2

G_{sol} = -751084.715

C -3.77547900 -0.37841900 0.07963400
H -4.24525100 -1.27317300 -0.30923500
C -3.91828400 0.03958900 1.38852900
H -4.52907900 -0.53132300 2.08060400
C -2.96962500 0.39073400 -0.77868400
C -3.25387400 1.19181400 1.82622100
H -3.33245800 1.50399100 2.85927300
C -2.48047300 1.91469100 0.92462400
N -2.37026500 1.52436900 -0.38222500
Pd -1.17457800 2.78255600 -1.61951700
O -2.78833100 -0.03279400 -2.02496200
C -1.69879800 3.11378800 1.31478700
C -1.73254200 3.69846500 2.58565900
N -0.90721900 3.62464200 0.34874000

C	-0.92829500	4.80798200	2.84067700
H	-2.37568300	3.30518200	3.36358900
C	-0.13748800	4.69390600	0.58085800
C	-0.11416800	5.31564100	1.82821400
H	-0.94279700	5.27235900	3.82185100
H	0.44284400	5.03382600	-0.27430100
H	0.52201700	6.17821400	1.99254400
C	2.33419300	0.90144900	-5.23175900
C	1.18206900	0.11321500	-5.32344600
C	-0.02944500	0.55122100	-4.78811200
C	-0.13533000	1.78554900	-4.13162800
C	1.03522300	2.56918000	-4.00335200
C	2.23176100	2.13228300	-4.57184300
H	1.22759500	-0.84892900	-5.82891800
H	-0.91812800	-0.06691900	-4.90106400
H	3.09634100	2.78267200	-4.47538300
C	1.06642800	3.88565800	-3.25148200
O	1.99758900	4.66810400	-3.41463700
O	0.08463600	4.15534700	-2.41345500
C	-1.44632000	2.26105500	-3.60565000
H	-1.71307300	3.25036100	-3.98949700
H	-2.26681700	1.58563300	-3.87689300
H	-2.19030300	0.59545400	-2.48181900
C	3.65610000	0.43506900	-5.79409100
H	4.20286400	1.26096700	-6.26072300
H	4.30156400	0.02668100	-5.00603500
H	3.51986100	-0.34917500	-6.54522500

Int2-3

G_{sol} = -894864.993

C	-1.04198500	3.59776700	-4.37852700
C	-0.17934800	2.70012300	-3.69862400
C	-0.51926400	2.15330200	-2.44502600
C	-1.75073500	2.47460700	-1.82563600
C	-2.60196300	3.32467000	-2.51002300
C	-2.26179400	3.90029800	-3.75887900
H	0.69581600	2.29748700	-4.19819000
C	-0.88313100	5.44463300	-0.87975900
O	-0.59715400	4.79989400	0.12677900
O	-0.27016000	5.34716600	-2.03969200
C	-2.01395100	6.46301500	-0.90357100
H	-1.66192900	7.41633300	-1.30918700
H	-2.80965400	6.10580900	-1.56441500
H	-2.40575100	6.60408600	0.10450700
Pd	1.18815200	3.93226100	-2.10113200
C	3.74469400	4.69928700	-0.96966300
C	4.70728200	5.48894100	-0.32461100
C	2.26535800	6.52824800	-1.01277400
C	4.42647900	6.81596600	-0.02762000
H	5.66796900	5.06443600	-0.06333600
C	3.18760300	7.35338600	-0.38545600
H	1.28761900	6.86992900	-1.32665700
H	5.16996200	7.42883800	0.47238700
H	2.93473900	8.38770200	-0.18286200
N	2.53986800	5.24088900	-1.28119600
C	-2.08316900	1.94284400	-0.45810200
H	-1.62351100	2.59402200	0.29366600
H	-3.16283800	1.92849500	-0.28300200

H -1.69438400 0.92958000 -0.31712500
C -0.59364300 4.16451600 -5.69943000
H -1.28780600 3.90687500 -6.50326000
H -0.56768300 5.25741200 -5.66453300
H 0.40338400 3.79282500 -5.94893700
C -3.20691800 4.86129000 -4.38764900
O -3.11803200 5.33406700 -5.50456900
O -4.23483600 5.20862300 -3.56109000
H -4.77766200 5.83188500 -4.07065700
C 3.93632700 3.30031900 -1.37888700
C 5.12082900 2.62791100 -1.14435100
C 5.24324100 1.29617900 -1.60198700
H 5.94175300 3.10551600 -0.62626700
C 4.20466300 0.71262400 -2.26807600
H 6.16475700 0.74822000 -1.42449300
H 4.25214200 -0.30286900 -2.64468100
C 2.95804400 1.41770000 -2.52266900
O 2.02089000 0.88646100 -3.13765600
N 2.87389200 2.72584000 -2.03116300
H -3.55993100 3.57914300 -2.07094600
H 0.12378200 1.37774200 -2.04165800

TS2-2

G_{sol} = -894855.296

Imaginary frequency = -1083.32

C 0.58081800 1.14801300 -0.03996700
H 0.53582900 0.07779500 0.12044900
C -0.50649200 1.96988200 0.13869900
H -1.45817300 1.55085900 0.45230500
C 1.83466600 1.69934400 -0.45256500
C -0.39555400 3.35650100 -0.08235500
H -1.24985000 4.00554900 0.05956700
C 0.82998200 3.86269600 -0.48162500
N 1.91107800 3.04756400 -0.65968400
O 2.86544300 0.96301800 -0.62880300
C 6.46544400 1.57352500 -3.27879500
C 5.16418600 1.87163600 -2.87844700
C 4.94913800 2.27845200 -1.53821700
C 6.04894200 2.34137700 -0.66044400
C 7.35987000 2.05889700 -1.04756400
C 7.55617000 1.67754200 -2.39867200
C 5.37792700 5.26642000 -3.12605000
O 4.53398000 4.98421800 -3.97650300
O 5.24427300 5.09472700 -1.83300400
C 6.73608900 5.84227400 -3.50520500
H 7.05926300 6.59958100 -2.78673000
H 7.46910600 5.02854600 -3.47828200
H 6.70098300 6.25602400 -4.51414900
Pd 3.63107000 3.96555800 -1.28769800
H 3.87285800 1.70439300 -1.03296700
C 1.08452900 5.29535100 -0.74350800
C 0.12172200 6.30550600 -0.64977300
C 2.71592100 6.86901800 -1.35152300
C 0.48475500 7.62284800 -0.91721900
H -0.89787100 6.06525700 -0.37460400
C 1.80214800 7.91542900 -1.27147700

H	3.75852500	7.00773600	-1.61568700
H	-0.25610400	8.41333100	-0.84900100
H	2.12060900	8.92936500	-1.48522900
N	2.35826400	5.60458800	-1.09565600
C	4.01682200	1.77577200	-3.84982100
H	3.11688900	1.41425600	-3.34172700
H	3.80411500	2.76965600	-4.25667800
H	4.24747400	1.09989800	-4.67870800
H	5.87137300	2.63190000	0.37214400
H	6.64928600	1.26341700	-4.30115800
C	8.92355800	1.37131300	-2.90686500
O	9.95564800	1.42148500	-2.26304900
O	8.93705700	1.01492800	-4.21960000
H	9.86945600	0.84668800	-4.43084500
C	8.47332000	2.17807200	-0.03567100
H	9.01601700	1.23608300	0.07939200
H	9.21817700	2.91487100	-0.34796100
H	8.06718600	2.47595700	0.93508000

Int2-4

G_{sol} = -894867.180

C	-0.65444900	-0.07203500	-0.02608600
H	-0.73358700	-1.12149400	0.22870400
C	-1.71899200	0.80094900	0.04221900
H	-2.69541500	0.45116700	0.36197800
C	0.59889800	0.42128100	-0.44438100
C	-1.53129100	2.14691600	-0.29700200
H	-2.35757200	2.84184800	-0.22971900
C	-0.27493300	2.57610600	-0.70617400
N	0.78396600	1.70955600	-0.78593200
O	1.60292400	-0.43918500	-0.48811900
C	5.80308800	-0.20973100	-0.89055200
C	4.85323600	0.81411700	-0.77613600
C	3.81092600	0.99748200	-1.69492800
C	3.70534800	0.13049600	-2.80503000
C	4.64718800	-0.89250500	-2.92805800
C	5.68573800	-1.07959700	-2.00019400
C	4.34741300	3.86778800	-3.33019400
O	3.56150000	3.47344900	-4.19097600
O	4.20939500	3.71824700	-2.03574700
C	5.63430800	4.59756000	-3.70123900
H	5.82899000	5.42521800	-3.01421400
H	6.47113300	3.89639900	-3.61398700
H	5.57746400	4.95675400	-4.72986200
Pd	2.59775500	2.59192200	-1.47180100
C	0.00260300	3.99139600	-1.05657400
C	-0.97477000	4.99304500	-1.10046200
C	1.65775900	5.53232700	-1.64417900
C	-0.59992400	6.29512700	-1.42309300
H	-2.01363800	4.76639000	-0.89555900
C	0.73852600	6.57696100	-1.69517500
H	2.71557900	5.66118900	-1.84978200
H	-1.34881000	7.07997000	-1.46285300
H	1.06674900	7.57870600	-1.94883000
N	1.29067400	4.28367300	-1.33550100
C	6.64583700	-2.19325600	-2.19876600

O	7.58537300	-2.47544200	-1.47515600
O	6.39013200	-2.92941500	-3.31763700
H	7.07993900	-3.61190700	-3.33325100
C	6.88616400	-0.32555900	0.15429700
H	6.85924000	-1.29852000	0.65261200
H	7.88091000	-0.25087900	-0.29311300
H	6.77375800	0.46263000	0.90430400
C	2.61106400	0.29708000	-3.83217800
H	1.62168800	0.21937600	-3.36420300
H	2.68017300	1.28774800	-4.29165300
H	2.67502000	-0.46868200	-4.61083300
H	4.58047300	-1.56806300	-3.77375900
H	4.94094200	1.49841300	0.06382700
H	2.42756200	-0.00760100	-0.81944000

Int3-1

G_{sol} = -798303.307

C	-2.95382900	-2.50259700	1.96043200
H	-3.53863700	-3.35942700	1.64621600
C	-2.83745700	-2.13680800	3.27347100
H	-3.34201500	-2.71162000	4.04536800
C	-2.28897800	-1.76355200	0.90412500
C	-2.06211100	-1.01221900	3.64372600
H	-1.96686800	-0.72546500	4.68259300
C	-1.43279400	-0.29523400	2.64561100
N	-1.54991500	-0.65720000	1.33173400
Pd	-0.50934500	0.45343100	0.02237100
O	-2.34874800	-2.06455200	-0.29829700
C	-0.60819300	0.89824100	2.87892700
C	-0.38451700	1.46463700	4.12390700
N	-0.06032800	1.45833100	1.74724400
C	0.40005300	2.62238900	4.21696500
H	-0.81824200	1.01947400	5.00921000
C	0.68277400	2.59345500	1.82086300
C	0.92963500	3.19100600	3.07959000
H	0.58393500	3.07390900	5.18694300
H	1.53429900	4.08917100	3.09340900
C	3.12109000	-1.82637300	-3.41079100
C	1.98093400	-2.61949000	-3.58843000
C	0.71869000	-2.14205300	-3.24393000
C	0.54186100	-0.86509800	-2.69771500
C	1.68559300	-0.06347600	-2.50001000
C	2.94629400	-0.54967000	-2.87358000
H	2.07916200	-3.61748500	-4.00865700
H	-0.15354000	-2.77036500	-3.40372500
H	3.79865300	0.10742000	-2.73277800
C	1.67241300	1.32213500	-1.90959000
O	2.59585900	2.09903800	-2.08317200
O	0.61335700	1.69544400	-1.17367000
C	-0.85818700	-0.41465800	-2.36558500
H	-1.03784100	0.64241300	-2.55721200
H	-1.60538600	-0.99308700	-2.91813100
H	-1.21079400	-0.76304100	-1.33691700
C	4.49520300	-2.34235900	-3.76487600
H	4.92134400	-2.92412100	-2.93803700
H	4.46278800	-2.99832600	-4.64049900

H 5.18681300 -1.52244200 -3.97847500

O 1.17906000 3.16788700 0.75868200

H 0.98895700 2.66061800 -0.11768700

TS3-1

G_{sol} = -798298.432

Imaginary frequency = -1101.30

C -3.86261600 -0.29747000 0.17457100

H -4.40736100 -1.16828000 -0.16897400

C -3.80637200 0.06406700 1.50044500

H -4.32425700 -0.53398400 2.24481900

C -3.17629300 0.48219800 -0.81019000

C -3.07825500 1.19909400 1.91142500

H -3.02449200 1.47086100 2.95734400

C -2.42872000 1.94742100 0.94449200

N -2.50105600 1.59197100 -0.36914500

Pd -1.45589200 2.70663100 -1.67662000

O -3.15955400 0.20265900 -2.04807900

C -1.61692900 3.15044600 1.20708200

C -1.44798000 3.71083000 2.46298700

N -1.02313100 3.70321300 0.09983300

C -0.65578400 4.86241100 2.59012200

H -1.92326800 3.27045200 3.32956000

C -0.27018900 4.82289100 0.20956200

C -0.06827400 5.42217700 1.47727800

H -0.51091800 5.31330900 3.56700700

H 0.54698400 6.31237300 1.51994800

C 2.01611800 0.28028600 -5.00792600

C 0.86549000 -0.51810000 -4.99653700

C -0.35917000 -0.00225700 -4.57994300

C -0.48555900 1.32290600 -4.14128400

C 0.67671600 2.12375400 -4.11462500

C 1.89485900 1.59885000 -4.56360700

H 0.92618300 -1.55348400 -5.32362500

H -1.24124200 -0.63761700 -4.59067900

H 2.75900200 2.25573200 -4.54728900

C 0.71805200 3.53878900 -3.59842800

O 1.64210800 4.29084900 -3.85958300

O -0.28796800 3.95266600 -2.81644600

C -1.84114700 1.82259400 -3.73078400

H -2.03657000 2.85918100 -4.00979700

H -2.62338800 1.24539500 -4.24076400

H -2.38524800 1.20160600 -2.71107500

C 3.33631600 -0.25703700 -5.50641500

H 3.44400500 -1.32384100 -5.28577200

H 3.42373700 -0.13904600 -6.59380300

H 4.17984700 0.27155200 -5.05297200

O 0.28198900 5.38373800 -0.83474100

H 0.10240300 4.88671900 -1.71709000

Int3-2

G_{sol} = -798314.742

C -1.08378800 0.20742200 0.03586300

H -0.39092400 0.97212300 0.36520000

C -2.10219500 -0.27203300 0.83205600

H -2.23607500 0.11592000 1.83724400

C	-0.94397600	-0.30454300	-1.27757400
C	-2.97436500	-1.25750300	0.34377000
H	-3.78308300	-1.62096800	0.96374100
C	-2.77370000	-1.74061100	-0.94408500
N	-1.76813300	-1.27040900	-1.72768500
Pd	-1.87254900	-1.95382700	-3.79364600
O	0.00475900	0.18598600	-2.04067800
C	-3.60490000	-2.81289800	-1.54667300
C	-4.59228200	-3.49022500	-0.83648200
N	-3.32604700	-3.15040500	-2.84373700
C	-5.28741900	-4.53677400	-1.45062400
H	-4.81107800	-3.22451400	0.18875400
C	-3.94341800	-4.20745600	-3.39741500
C	-4.95996600	-4.91703200	-2.73874800
H	-6.06413600	-5.06494600	-0.90674300
H	-5.43611300	-5.74350900	-3.25108600
C	1.52358100	-4.05268700	-7.45234700
C	0.26167900	-4.61179400	-7.67884400
C	-0.88711400	-4.02819000	-7.14408200
C	-0.82228300	-2.87052100	-6.35523000
C	0.45624300	-2.32103800	-6.09430900
C	1.59321600	-2.90130900	-6.65855700
H	0.17218000	-5.50747600	-8.28924000
H	-1.85925600	-4.46609100	-7.36169500
H	2.55017500	-2.43185700	-6.45047600
C	0.68112000	-1.13202400	-5.19006600
O	1.74914100	-0.53893600	-5.17469700
O	-0.31771400	-0.76048300	-4.38998100
C	-2.06690300	-2.24305600	-5.82975100
H	-2.15044200	-1.18880300	-6.11149500
H	-2.97239900	-2.75174700	-6.17495600
H	-0.02495100	-0.15153000	-3.00427100
C	2.77780700	-4.67904300	-8.01339100
H	3.47503800	-3.91641500	-8.37509500
H	3.30617200	-5.26122600	-7.24802400
H	2.55304400	-5.35520500	-8.84378100
O	-3.57276300	-4.61667900	-4.60939900
H	-2.82133400	-4.06695300	-4.90620200

Int3-3

G_{sol} = -942090.557

C	0.12448900	1.10448100	0.06386300
H	0.17735400	0.04704500	0.29658400
C	-0.91973600	1.89150000	0.45558300
H	-1.74860300	1.46956200	1.01763200
C	1.25109500	1.63858000	-0.68209100
C	-0.91571600	3.27040500	0.14587400
H	-1.71832100	3.90958400	0.48802100
C	0.13845200	3.78276700	-0.58497500
N	1.16664800	2.99122800	-1.03817500
O	2.22495200	0.93722100	-0.99599100
C	3.27824700	1.98186800	-3.46369600
C	4.29729600	2.29990900	-2.54173100
C	5.49157400	2.91440000	-2.97492000
C	5.63343100	3.14455300	-4.33280800
C	4.63268300	2.80142800	-5.27619000

C	3.42946800	2.21573700	-4.85885400
H	4.16897500	1.96170200	-1.51834400
C	2.84921600	5.36343200	-4.83517800
O	1.76647500	4.88815600	-5.13793200
O	3.46251400	5.14116300	-3.66937500
C	3.64523900	6.28211600	-5.74141200
H	3.11319300	6.42039700	-6.68286700
H	3.78641800	7.24966600	-5.24903500
H	4.63519200	5.85628500	-5.92551700
Pd	2.40165200	3.94927700	-2.36511100
C	0.27370700	5.22199500	-0.86119200
C	-0.59742700	6.19712900	-0.38608900
C	1.69383900	6.87929100	-1.76820700
C	-0.30630000	7.54431100	-0.61805400
H	-1.47832100	5.91746000	0.17509800
C	0.84531300	7.89723700	-1.29515700
H	-0.97914400	8.31361600	-0.25197400
H	1.12887800	8.92502100	-1.48382700
N	1.38570000	5.57656600	-1.57511000
C	4.86916900	3.09211300	-6.71629500
O	4.17675300	2.73081400	-7.64702900
O	5.98066100	3.85630800	-6.92410800
H	6.03458100	3.97705800	-7.88613000
C	2.30773300	1.82135500	-5.77890700
H	1.94145000	2.69518500	-6.32237900
H	2.64259900	1.09811200	-6.52631200
H	1.48241400	1.39734000	-5.20221100
C	6.55296600	3.31029800	-1.98099200
H	7.51149300	3.50443200	-2.47009400
H	6.25878200	4.22345800	-1.44963300
H	6.70282100	2.53068800	-1.22704200
H	2.46360600	1.35780900	-3.11255000
H	6.54504300	3.60268700	-4.69977900
O	2.80826700	7.22922500	-2.38420400
H	3.23737500	6.43524100	-2.83430700

TS3-2

G_{sol} = -942082.679

Imaginary frequency = -871.56

C	0.58298500	1.04976500	-0.06297800
H	0.55074300	-0.01286300	0.14348900
C	-0.50808500	1.86989100	0.09171400
H	-1.45332500	1.45881500	0.43415200
C	1.82676300	1.58868800	-0.51931700
C	-0.40847300	3.24511700	-0.18943100
H	-1.26528400	3.89309500	-0.06283200
C	0.80671100	3.74768800	-0.62406000
N	1.89241400	2.93133900	-0.78503400
O	2.85295400	0.84561700	-0.67725900
C	6.48978400	1.55624500	-3.48146800
C	5.19557800	1.80788100	-3.01726600
C	4.90697000	2.12463500	-1.67697800
C	5.96000800	2.13195000	-0.72825700
C	7.24858200	1.84301100	-1.16534600
C	7.52891600	1.58229000	-2.51933800
C	5.38811700	4.84883300	-3.54562400

O	4.57834100	4.40117200	-4.34182800
O	5.19209800	4.91627700	-2.22685900
C	6.75633400	5.36111600	-3.95401900
H	6.97096300	6.32302300	-3.48055400
H	7.50789300	4.64661700	-3.60182400
H	6.81473900	5.44800200	-5.03926600
Pd	3.57270200	3.82785800	-1.51677900
H	3.84079300	1.56992300	-1.20937400
C	1.04029900	5.17736700	-0.91550600
C	0.06595200	6.16023500	-0.79783800
C	2.66095600	6.77948200	-1.51842800
C	0.41262600	7.49265400	-1.05936700
H	-0.94379900	5.90582700	-0.50487600
C	1.70688300	7.81467900	-1.41309200
H	-0.33819900	8.27225600	-0.97530000
H	2.02810900	8.82917500	-1.61380100
N	2.31468000	5.49606200	-1.29297500
C	8.94203700	1.33388400	-2.92640700
O	9.34060900	1.17055500	-4.06441400
O	9.80228200	1.30776000	-1.87301000
H	10.67862500	1.14873500	-2.25933400
C	6.70444700	1.30373900	-4.95329900
H	7.36093800	2.06100000	-5.38982300
H	7.19331100	0.34266400	-5.13307400
H	5.74694500	1.32718800	-5.47902300
C	5.68288300	2.43914500	0.72213300
H	5.64219900	3.52199300	0.88837700
H	4.71454800	2.02453500	1.02029600
H	6.45807300	2.02943800	1.37639500
H	8.06607400	1.83272800	-0.45333000
H	4.38164800	1.79725600	-3.73439400
O	3.90124800	7.09577400	-1.82596200
H	4.48585600	6.27542200	-1.92985400

Int3-4

G_{sol} = -942095.047

C	-1.42442000	1.06735800	0.16978500
H	-1.49780100	0.00698900	0.37606800
C	-2.49538300	1.92965400	0.26112800
H	-3.47415500	1.56147300	0.55182100
C	-0.17299400	1.58401600	-0.22066600
C	-2.31278200	3.28636500	-0.02646800
H	-3.15025900	3.96677100	0.03884700
C	-1.05441500	3.74758000	-0.39692300
N	0.01379000	2.89076100	-0.49417300
C	4.73212000	0.84003500	-0.21469400
C	3.80502600	1.87712400	-0.10571700
C	3.06534500	2.23028400	-1.25598900
C	3.28725800	1.54518000	-2.45762800
C	4.21879400	0.50324900	-2.57573800
C	4.95205300	0.15036400	-1.41904800
C	3.58947500	4.82415200	-3.25663800
O	2.88549400	4.19169400	-4.02817500
O	3.37915400	4.92289600	-1.94442200
C	4.81165300	5.60767500	-3.70377800

H	4.69876100	6.66196200	-3.43130400
H	5.69823400	5.23063100	-3.18556500
H	4.93998800	5.51546500	-4.78246500
Pd	1.79643400	3.79600100	-1.21218800
C	-0.80234200	5.18239000	-0.68468800
C	-1.78273800	6.16302000	-0.56375800
C	0.80542000	6.78032400	-1.28581200
C	-1.43947400	7.49561200	-0.82949400
H	-2.79234600	5.91788200	-0.26396300
C	-0.14675600	7.81860300	-1.18760400
H	-2.19209000	8.27343200	-0.74447400
H	0.17138500	8.83343300	-1.39222300
N	0.46434000	5.49917000	-1.05693600
C	3.59495300	2.57936300	1.21605000
H	3.81053400	3.64973900	1.13043600
H	2.55389200	2.48686200	1.54825500
H	4.23654500	2.16105400	1.99704200
H	2.73920400	1.85193100	-3.34184900
H	5.30659600	0.55341700	0.65935200
C	5.95298800	-0.94560700	-1.45508000
O	6.23614500	-1.63329500	-2.41998800
O	6.57328900	-1.14360600	-0.25704900
H	7.19377700	-1.87381900	-0.41126100
C	4.39003500	-0.17031900	-3.91525800
H	5.42259000	-0.09782900	-4.26714800
H	4.17335500	-1.24045200	-3.85451400
H	3.72877900	0.28848400	-4.65501800
O	0.82666800	0.72057700	-0.30346300
H	1.64656000	1.15172200	-0.64286000
O	2.04800800	7.09547300	-1.59472300
H	2.63781300	6.27615400	-1.67394300

VIII. References

(1) H. T. Kim, E. Kang, M. Kim and J. M. Joo, *Org. Lett.*, 2021, **23**, 3657-3662.

(2) S. J. Yun, J. Kim, E. S. Kang, H. M. Jung, H. T. Kim, M. Kim and J. M. Joo, *ACS Catal.*, 2023, **13**, 4042-4052.

(3) (a) L. Y. Liao, X. R. Kong and X. F. Duan, *J. Org. Chem.*, 2014, **79**, 777-782; (b) T. Tomon, T. Koizumi and K. Tanaka, *Eur. J. Inorg. Chem.*, 2005, **2005**, 285-293.

(4) J. Kong, Z. J. Jiang, J. Xu, Y. Li, H. Cao, Y. Ding, B. Tang, J. Chen and Z. Gao, *J. Org. Chem.*, 2021, **86**, 13350-13359.

(5) (a) A. Uttry, S. Mal and M. van Gemmeren, *J. Am. Chem. Soc.*, 2021, **143**, 10895-10901; (b) C. Teja, S. Kolb, P. Colonna, J. Grover, S. Garcia-Argote, G. K. Lahiri, G. Pieters, D. B. Werz and D. Maiti, *Angew. Chem. Int. Ed.*, 2024, **63**, e202410162.

(6) Z. Wang, L. Hu, N. Chekshin, Z. Zhuang, S. Qian, J. X. Qiao and J. Q. Yu, *Science*, 2021, **374**, 1281-1285.

(7) Y. P. Wang, X. J. Li, F. Q. Leng, H. L. Zhu, J. Y. Li, D. P. Zou, Y. J. Wu and Y. S. Wu, *Adv. Synth. Catal.*, 2014, **356**, 3307-3313.

(8) Y. Li, C. Zheng, Z. J. Jiang, J. Tang, B. Tang, Z. Gao, *Chem. Commun.*, 2022, **58**, 3497-3500.

(9) N. K. Razdan, T. C. Lin and A. Bhan, *Chem. Rev.*, 2023, **123**, 2950-3006.

(10) V. A. Pistritto, S. Liu and D. A. Nicewicz, *J. Am. Chem. Soc.*, 2022, **144**, 15118-15131.

(11) R. C. Gaussian 16, M. J. Frisch, G. W. Trucks, H. B. Schlegel, G. E. Scuseria, M. A. Robb, J. R. Cheeseman, G. Scalmani, V. Barone, G. A. Petersson, H. Nakatsuji, X. Li, M. Caricato, A. V. Marenich, J. Bloino, B. G. Janesko, R. Gomperts, B. Mennucci, H. P. Hratchian, J. V. Ortiz, A. F. Izmaylov, J. L. Sonnenberg, D. Williams-Young, F. Ding, F. Lipparini, F. Egidi, J. Goings, B. Peng, A. Petrone, T. Henderson, D. Ranasinghe, V. G. Zakrzewski, J. Gao, N. Rega, G. Zheng, W. Liang, M. Hada, M. Ehara, K. Toyota, R. Fukuda, J. Hasegawa, M. Ishida, T. Nakajima, Y. Honda, O. Kitao, H. Nakai, T. Vreven, K. Throssell, J. A. Montgomery, Jr., J. E. Peralta, F. Ogliaro, M. J. Bearpark, J. J. Heyd, E. N. Brothers, K. N. Kudin, V. N. Staroverov, T. A. Keith, R. Kobayashi, J. Normand, K. Raghavachari, A. P. Rendell, J. C. Burant, S. S. Iyengar, J. Tomasi, M. Cossi, J. M. Millam, M. Klene, C. Adamo, R. Cammi, J. W. Ochterski, R. L. Martin, K. Morokuma, O. Farkas, J. B. Foresman, and D. J. Fox, Gaussian, Inc., Wallingford CT, 2016.

(12) (a) A. D. Becke, *J. Chem. Phys.*, 1993, **98**, 1372-1377; (b) C. Lee, W. Yang and R. G. Parr, *Phys. Rev. B Condens. Matter*, 1988, **37**, 785-789; (c) S. Grimme, J. Antony, S. Ehrlich and H. Krieg, *J. Chem. Phys.*, 2010, **132**, 154104.

(13) R. Ditchfield, W. J. Hehre and J. A. Pople, *J. Chem. Phys.*, 1971, **54**, 724-728.

(14) (a) W. R. Wadt and P. J. Hay, *J. Chem. Phys.*, 1985, **82**, 284-298; (b) P. J. Hay and W. R. Wadt, *J. Chem. Phys.*, 1985, **82**, 299-310; (c) P. J. Hay and W. R. Wadt, *J. Chem. Phys.*, 1985, **82**, 270-283.

(15) A. Nicklass, M. Dolg, H. Stoll and H. Preuss, *J. Chem. Phys.*, 1995, **102**, 8942-8952.

(16) The Minnesota Solvent Descriptor Database, see https://comp.chem.umn.edu/solvation/mnsddb.pdf.
